# Activation of Saturated Fluorocarbons to Synthesize Spirobiindanes, Monofluoroalkenes, and Indane Derivatives

**DOI:** 10.1016/j.isci.2019.06.018

**Published:** 2019-06-19

**Authors:** Jiandong Wang, Yuta Ogawa, Norio Shibata

**Affiliations:** 1 Department of Nanopharmaceutical Sciences and Department of Life Science and Applied Chemistry, Nagoya Institute of Technology, Gokiso, Showa-ku, Nagoya 466-5888, Japan; 2 Institute of Advanced Fluorine-Containing Materials, Zhejiang Normal University, 688 Yingbin Avenue, 321004 Jinhua, China

**Keywords:** Chemistry, Organic Chemistry, Organic Synthesis

## Abstract

Fluorinated organic compounds are produced in abundance by the pharmaceutical and agrochemical industry, making such compounds attractive as building blocks for further functionalization. Unfortunately, activation of C(sp^3^)-F bond in saturated fluorocarbons, especially for aliphatic *gem*-difluoroalkanes, remains challenging. Here we describe the selective activation of inert C(sp^3^)-F bonds catalyzed by B(C_6_F_5_)_3_. In hexafluoro-2-propanol (HFIP), chemically robust aliphatic *gem*-difluorides are converted in high yields to the corresponding substituted 2,2′,3,3′-tetrahydro-1,1′-spirobiindenes via a B(C_6_F_5_)_3_-catalyzed intramolecular cascade Friedel-Crafts cyclization, not requiring a silicon-based trapping reagent. However, in the absence of a hydrogen-bonding donor solvent such as HFIP, the aliphatic *gem*-difluorides preferentially engage in a defluorination/elimination process that provides monofluorinated alkenes in good yields. Furthermore, a series of substituted 1-alkyl-2,3-dihydro-1*H*-indenes was obtained in high yield from the B(C_6_F_5_)_3_-catalyzed defluorinative cyclization of aliphatic secondary monofluorides in HFIP. The protocol could inspire development of a new class of main-group Lewis acid-catalyzed C(sp^3^)-F bond activation in general unactivated fluorocarbons.

## Highlights

• C(sp^3^)-F bond activation in general unactivated fluorocarbons
• The activation of C(sp^3^)-F bonds in aliphatic *gem*-difluoroalkanes
• The selective activation of inert C(sp^3^)-F bonds catalyzed by B(C_6_F_5_)_3_
• An intramolecular cascade defluorinative Friedel-Crafts cyclization

## Graphical Abstract

**Figure:**
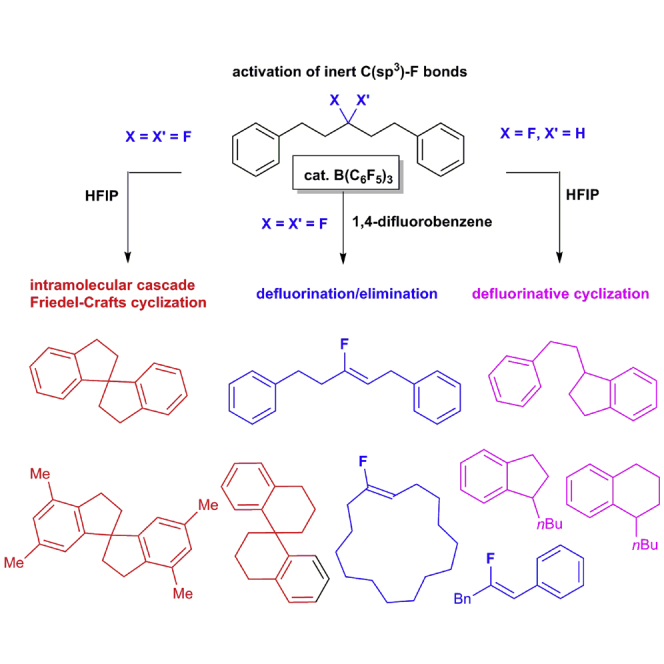

## Introduction

The demand for the selective activation of C-F bonds is growing as a result of the increased availability of fluorinated compounds in the pharmaceutical and agrochemical industries (Amii and Uneyama, 2009, Ahrens et al., 2015, Kuehnel et al., 2013, Hamel and Paquin, 2018, Klare, 2017). Recently, remarkable progress has been made in the transition-metal-mediated heterolysis of C(sp^2^)-F bonds in aromatic and vinylic fluorocarbons (Ahrens et al., 2015, Kuehnel et al., 2013, Pike et al., 2017, Guo et al., 2015, Luo et al., 2018). However, the defluorinative functionalization of C(sp^3^)-F bonds in unactivated aliphatic fluorides is less frequently reported and still a challenging issue in synthetic organic chemistry (Stahl et al., 2013, Shen et al., 2015). Indeed, the notorious chemical robustness of C-F bonds not only stems from their thermodynamic stability—the C-F bond is among the strongest covalent single bonds that carbon can form—but also from kinetic factors because the fluoride moiety is neither a good leaving group nor a good Lewis base (O'Hagan, 2008, Nolte et al., 2012).

The direct abstraction of the fluoride moiety in inert C(sp^3^)-F bonds by *p*-block-based Lewis acids that exhibit high fluoride affinity has emerged as a promising strategy for the degradation of saturated fluorocarbons (Stahl et al., 2013, Shen et al., 2015), because of that the formation of covalent bonds between fluorine and main-group elements (e.g., B, Al, Si, and P), which are more stable than C-F bonds, may offer a thermodynamic driving force for the scission of the C-F bonds (Stahl et al., 2013). In addition, the stronger Lewis acidity of fluorophilic electrophiles is essential for the direct heterolytic cleavage of C(sp^3^)-F bonds, given the high activation barrier. The substitution of the fluoride in C(sp^3^)-F bonds to form C-H, C-C, and C-heteroatom bonds has been initiated by neutral, strong aluminum- and boron-based Lewis acids (Stahl et al., 2013, Greb, 2018, Morgan et al., 2013, Koerte et al., 2017, Ahrens et al., 2013, Jaiswal et al., 2017) or cationic species such as [CPh_3_]^+^, [SiEt_3_]^+^, [*i*Bu_2_Al]^+^, [(C_6_F_5_)_3_FP]^+^, and even P(III) dications such as [(bipy)PPh]^2+^ bearing weakly coordinating counter anions such as [B(C_6_F_5_)_4_]^-^ (Stahl et al., 2013, Klahn et al., 2007, Gu et al., 2009, Forster et al., 2017, Douvris and Ozerov, 2008, Scott et al., 2005, Großekappenberg et al., 2015, Zhu et al., 2016, Chitnis et al., 2018).

In their seminal reports, Olah and co-workers described the cleavage of unactivated C(sp^3^)-F bonds initiated by boron-based Lewis acids, specifically the preferential abstraction of fluorides from aliphatic fluorohaloalkanes by boron trifluoride to generate the stable BF_4_^-^ anion and Friedel-Crafts-type alkylation products with excess arenes (Olah et al., 1957, Olah and Kuhn, 1964). Consistent with the greater stability of tertiary carbocations derived from tertiary aliphatic fluorides, Oshima and co-workers have reported that BF_3_·OEt_2_ catalyzes C-C bond couplings between silicon enolates and tertiary fluorides (Hirano et al., 2004). Subsequently, Stephan et al. have reported the splitting of unactivated C(sp^3^)-F bonds using stoichiometric amounts of B(C_6_F_5_)_3_ and a phosphine as frustrated Lewis pairs (FLPs) to produce [R_3_PR’][FB(C_6_F_5_)_3_] salts (Caputo and Stephan, 2012). Alternatively, using catalytic amounts of B(C_6_F_5_)_3_ with an excess of Et_3_SiH, a C-H bond is formed at the expense of the corresponding C(sp^3^)-F bond (Caputo and Stephan, 2012). Furthermore, HB(C_6_F_5_)_2_ has been used to induce the direct C(sp^3^)-F borylation of secondary and primary aliphatic fluoroalkanes via an initial dehydrofluorination and a subsequent borylation of the resulting olefin intermediates (Bamford et al., 2018). Recently, Moran and co-workers have reported the Friedel-Crafts reactions of tertiary fluoroalkanes with an excess of arenes (3.0–5.0 equiv.) catalyzed by B(C_6_F_5_)_3_ in MeNO_2_ under ambient atmosphere; interestingly, in this case the Lewis acid B(C_6_F_5_)_3_ absorbs water to generate [(C_6_F_5_)_3_B(OH_2_)_n_], which then acts as a Brønsted acid (Dryzhakov and Moran, 2016, Dryzhakov et al., 2017, Beringhelli et al., 2001). Despite the general progress in this area, the development of alternative catalytic methods based on boron-based Lewis acids as fluorophilic electrophiles for the activation of inert C(sp^3^)-F bonds in saturated fluorocarbons remains highly desirable.

**Figure 1:**
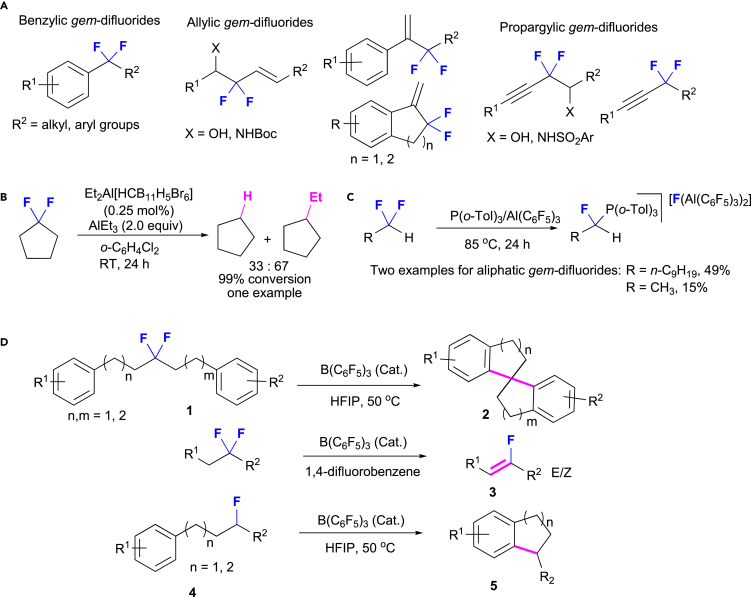
Activation of C(sp^3^)-F Bonds in *gem*-Difluoroalkanes (A) Activated benzylic, allylic, and propargylic *gem*-difluoroalkanes. (B) [Et_2_Al][HCB_11_H_5_Br_6_]-induced C(sp^3^)-F functionalization of 1,1-difluorocyclopentane. (C) Monodefluorination of a *gem*-difluoromethyl group initiated by the FLP Al(C_6_F_5_)_3_/P(*o*-Tol)_3_. (D) Synthesis of spirobiindanes, monofluoroalkenes, and 1-alkyl-2,3-dihydro-1H-indenes via a B(C_6_F_5_)_3_-catalyzed activation of C(sp^3^)-F bonds (this work).

The modification of C(sp^3^)-F bonds in aliphatic *gem*-difluoroalkanes is much more difficult than in the corresponding saturated monofluorocarbons because the strength of C-F bonds increases with the number of geminal fluorine atoms (Hamel and Paquin, 2018, O'Hagan, 2008). Indeed, in most cases, the fluorine moiety in *gem*-difluorides is found at activated benzylic, allylic, or propargylic positions (Figure 1A), as well as at the *α*-position of a carbonyl group or in *gem*-difluorocyclopropanes (Hamel and Paquin, 2018, Song et al., 2017). In a representative example of unactivated aliphatic *gem*-difluoroalkanes from Ozerov and co-workers, the ethylation of 1,1-difluorocyclopentane was observed together with the reduction side product cyclopentane (67:33) by gas chromatography-mass spectrometry analysis as one special case (Figure 1B) by using catalytic amounts of [Et_2_Al][HCB_11_H_5_Br_6_] in the presence of an excess amount of AlEt_3_ (Gu et al., 2009). In 2018, the group of Young reported two examples for the monodefluorination of an acyclic aliphatic *gem*-difluoromethyl group in 1,1-difluoroethane and 1,1-difluorodecane: using FLPs obtained from Al(C_6_F_5_)_3_ and P(*o*-Tol)_3_, the *α*-fluoroalkylphosphonium salts were generated in moderate yield (Figure 1C) (Mandal et al., 2018). Building upon our long-standing interest in the activation of inert C(sp^3^)-F bonds (Haufe et al., 2012, Tanaka et al., 2016, Cui et al., 2018), we discovered in this study that a catalytic amount of the Lewis acid B(C_6_F_5_)_3_ activated the C(sp^3^)-F bond in aliphatic *gem*-difluoroalkanes of type **1** to selectively generate substituted 2,2′,3,3′-tetrahydro-1,1′-spirobiindenes (**2**) and monofluorinated alkenes (**3**) in good yield (Figure 1D). Moreover, this method was also used for the functionalization of the C(sp^3^)-F bond in secondary monofluoroalkanes to C(sp^3^)-C(sp^3^) bonds in good yield. The use of hydrogen-bonding hexafluoro-2-propanol (HFIP) as the solvent was essential to induce the catalyst turnover for the defluorinative Friedel-Crafts alkylation.

## Results and Discussion

### Optimization Study

Initially, based on the pioneering work of Olah and Stephan on the activation of C(sp^3^)-F bonds in saturated monofluoroalkanes initiated by boron-based Lewis acids (Olah et al., 1957, Olah and Kuhn, 1964, Caputo and Stephan, 2012), we attempted to use stoichiometric amounts of BF_3_·OEt_2_ and B(C_6_F_5_)_3_ (2.2 equiv.) to induce cleavage of the C(sp^3^)-F bond in unactivated *gem*-difluoroalkane **1a** (Table 1, entries 1 and 2). Although no reaction was detected upon using BF_3_·OEt_2_, the use of a stoichiometric amount of B(C_6_F_5_)_3_ afforded 2,2′,3,3′-tetrahydro-1,1′-spirobi[indene] **2a** in 85% yield. This result was very encouraging, considering that examples of the activation of C(sp^3^)-F bonds in inert aliphatic *gem*-difluoroalkanes are extremely rare (Figure 1) (Gu et al., 2009, Mandal et al., 2018). Subsequently, we turned our attention to the development of a catalytic B(C_6_F_5_)_3_-induced cascade intramolecular Friedel-Crafts cyclization (Lan et al., 2006, Birman et al., 1999, Li et al., 2016, Zheng et al., 2018).

**Table 1:** Optimization of the Selective Cleavage of C(sp^3^)-F Bonds in Aliphatic *gem*-Difluoroalkanes


Entry	Lewis Acids (Equiv.)	Solvent (0.1M)	T (°C)	t (h)	Yields (%)	
					**2a**	**3a**^a^
1	BF_3_·OEt_2_ (2.2)	CH_2_Cl_2_	RT	30	NR	NR
2	B(C_6_F_5_)_3_ (2.2)	CH_2_Cl_2_	RT	30	85	0
3	B(C_6_F_5_)_3_ (0.2)	CH_2_Cl_2_	RT	30	Trace	0
4	B(C_6_F_5_)_3_ (0.2)	(CF_3_)_2_CHOH	RT	17	28	0
5	B(C_6_F_5_)_3_ (0.2)	(CF_3_)_2_CHOH	50	2	75	0
6	B(C_6_F_5_)_3_ (0.1)	(CF_3_)_2_CHOH	50	20	16	0
7	B(C_6_F_5_)_3_ (0.2)	(CF_3_)_2_CHOH^b^	50	2	27	0
8	B(C_6_F_5_)_3_ (0.2)	(CF_3_)_2_CHOH^c^	50	2	84	0
9	B(C_6_F_5_)_3_ (0.2)	(CF_3_)_2_CHOH^c^ H_2_O (2.2 equiv.)	50	2	0^d^	0^d^
10	B(C_6_F_5_)_3_ (0.2)	iPrOH^c^	50	2	NR	NR
11	B(C_6_F_5_)_3_ (0.2)	CF_3_CH_2_OH^c^	50	2	NR	NR
12	B(C_6_F_5_)_3_ (0.2)	(CF_3_)_2_PhOH^c^	50	2	NR	NR
13	–	(CF_3_)_2_CHOH^c^	50	2	NR	NR
14	B(C_6_F_5_)_3_ (0.2)	*o*-C_6_H_4_Cl_2_	160	3	0	64
15	B(C_6_F_5_)_3_ (0.1)	*o*-C_6_H_4_Cl_2_	160	3	0	30
16	–	*o*-C_6_H_4_Cl_2_	160	3	NR	NR
17	B(C_6_F_5_)_3_ (0.2)	*o*-C_6_H_4_Cl_2_	220	3	0	81^e^
18	B(C_6_F_5_)_3_ (0.2)	*p*-C_6_H_4_F_2_	reflux	3	0	75
19	B(C_6_F_5_)_3_ (0.2)	*p*-C_6_H_4_F_2_	reflux	24	0	87^f^

RT, room temperature; NR, no reaction.

a Determined by^19^F NMR analysis using PhCF_3_ as the internal standard.

b (CF_3_)_2_CHOH was used as purchased without any precaution to exclude moisture. The hydrolysis product 1,5-diphenylpentan-3-one was observed as the major product.

c Concentration: 0.05 M.

d The hydrolysis product 1,5-diphenylpentan-3-one was obtained in quantitative yield after 12 h at 50°C.

e *Z*/*E* = 7.3:1.

f *Z*/*E* = 7.1:1.

Recently, HFIP has attracted considerable attention as a solvent to promote Friedel-Crafts acylations and alkylations (Motiwala et al., 2015, Vekariya and Aube, 2016, Tang et al., 2018) owing to its unique properties, which include reduced nucleophilicity, a strong propensity to engage as a hydrogen-bonding donor, and the ability to stabilize cationic intermediates (Colomer et al., 2017). Indeed, intermolecular Friedel-Crafts alkylations by hydrogen-bonding interactions between activated benzylic C-F bonds and HFIP in the absence of any Lewis or Brønsted acids has been reported by Paquin and co-workers (Champagne et al., 2014, Champagne et al., 2015). Using a combination of the hydrogen-bonding-donor solvent HFIP and B(C_6_F_5_)_3_ (20 mol %) in the absence of any silicon-based trapping reagent, afforded the defluorinative Friedel-Crafts-type product **2a** in 75% yield (Table 1, entry 5). It is worth noting here that moisture was strictly excluded in our method, owing to the hydrolysis of **1a** under acidic conditions. When using “wet” HFIP, i.e., HFIP that was used as purchased under an atmosphere of argon without any precaution to exclude moisture, the corresponding hydrolysis product (1,5-diphenylpentan-3-one) was observed as the major product and **2a** was obtained in only 27% yield (Table 1, entry 7). Upon adding H_2_O (2.2 equiv.), the intramolecular Friedel-Crafts transformation was completely suppressed, and the quantitative hydrolysis into a ketone was confirmed instead when prolonging the reaction time (Table 1, entry 9). Other reaction parameters, such as solvents, concentration, and temperature, were also investigated (for more details, see also Table S1). Finally, we were able to identify the optimal reaction conditions for the synthesis of spirobiindanes **2**: B(C_6_F_5_)_3_ (20 mol%) in HFIP (0.05 M) at 50°C for 2 h (Table 1, entry 8). In the absence of B(C_6_F_5_)_3_, or when using other hydrogen-bonding solvents such as iPrOH, CF_3_CH_2_OH, or (CF_3_)_2_PhOH, the reaction did not proceed (Table 1, entries 10–13). Unexpected results were obtained using 1,2-dichlorobenzene as the solvent. Indeed, the formation of monofluoroalkene **3a**, derived from the defluorination/elimination sequence of aliphatic *gem*-difluoroalkane **1a** (Yanai et al., 2011, Yang et al., 2013, Surmont et al., 2009, Li et al., 2018, Drouin et al., 2018), was observed in good yield when conducting the reaction at very high temperatures (Table 1, entries 14, 17). Specifically, when the temperature was increased from 160°C to 220°C in a sealed tube, the yield of the elimination product **3a** increased from 64% to 81% with good stereoselectivity (*Z*/*E* = 7.3:1), whereas no reaction was detected in the absence of B(C_6_F_5_)_3_ (entry 16). These results indicate that the increased reaction temperature is beneficial for the defluorination/elimination. Furthermore, after screening the reaction conditions to find an acceptable reaction temperature (for further details, see also Table S2) in the presence of B(C_6_F_5_)_3_ (20 mol%), we discovered that stirring the reaction mixture in refluxing 1,4-difluorobenzene (boiling point 88–89°C) instead of using harsher reaction conditions (220°C) afforded **3a** in 87% yield with good stereoselectivity (*Z*/*E* = 7.1:1).

### Substrate Scope

**Figure 2:**
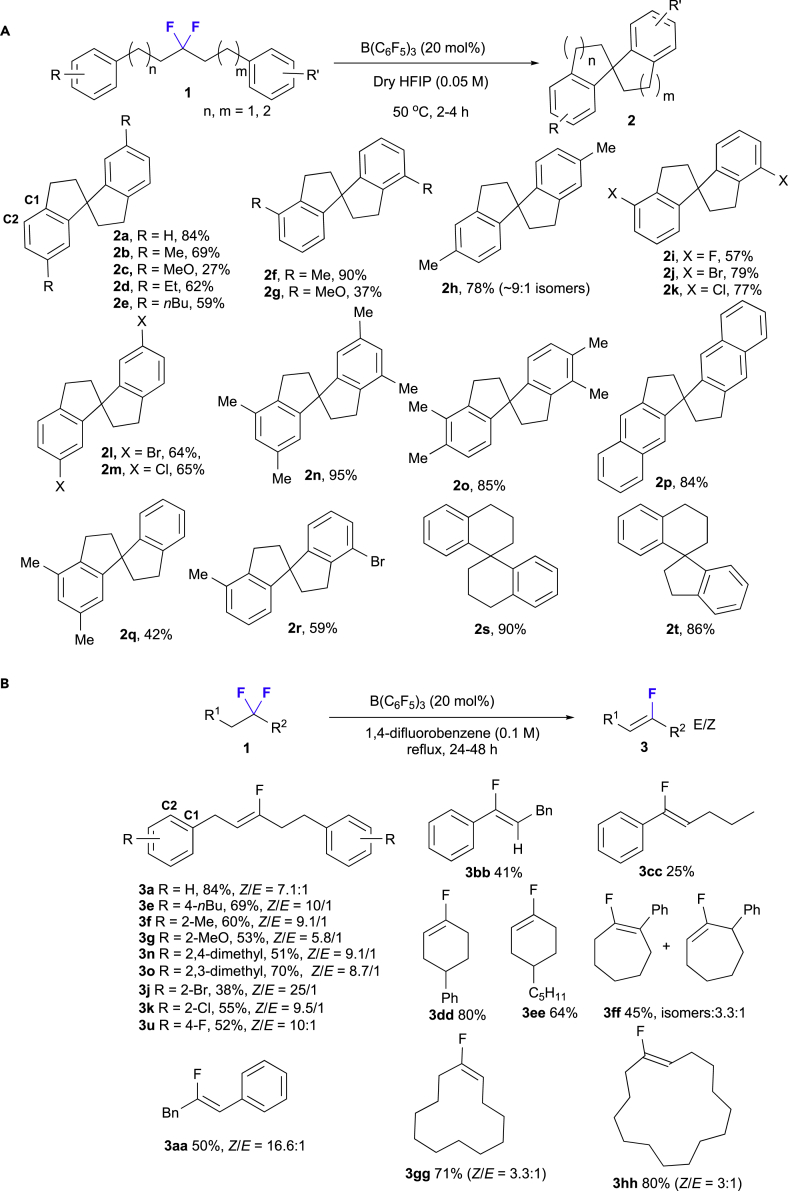
Substrate Scope of the Defluorination of Aliphatic *gem*-Difluoroalkanes to Afford Spirobiindanes **2** and Monofluoroalkenes **3** (A) Intramolecular Friedel-Crafts reactions. (B) Defluorination/elimination reactions.

With the optimized reaction conditions in hand, we explored the substrate scope (Figure 2). First, we examined intramolecular Friedel-Crafts reactions as shown in Figure 2A. For aliphatic *gem*-difluoroalkanes substituted with alkyl groups (**1a**-**h**), good to high yields were observed; in particular, *gem*-difluoroalkane **1f**, bearing a methyl group at the C2 position of the benzene ring, generated the desired product (**2f**) in high yield (up to 90%). However, for methoxy-substituted *gem*-difluorides **1c** and **1g**, substantially lower yields were observed due to the presence of the electron-rich heteroatom acting as a Lewis base that is able to provide lone electron pairs to interact with the Lewis acid catalyst. This unexpected donor-acceptor interaction between the oxygen atom and the electron-deficient boron moiety hampered the fluoride abstraction via the C(sp^3^)-F→B(C_6_F_5_)_3_ interaction, leading to decreased yields. In contrast, *gem*-difluoroalkanes (**1i**-**m**) with a halogen (F, Cl, Br) group at the C2 or C4 position afforded acceptable yields (57–79%), whereas dialkyl-substituted substrates **1n** and **1o** furnished good to excellent product yields (up to 95%). Naphthyl-type 2,2′,3,3′-tetrahydro-1,1′-spirobi[cyclopenta[b]naphthalene] **2p** was also prepared in good yield (84%). Moreover, 4,6-dimethyl-2,2′,3,3′-tetrahydro-1,1′-spirobi[indene] **2q** and 4-bromo-4′-methyl-2,2′,3,3′-tetrahydro-1,1′-spirobi[indene] **2r** were generated in moderate yields (42% and 59%, respectively). Six-membered spiro-compound **2s** and five- or six-membered spiro-compound **2t** were also prepared in high yields (up to 90%). As shown in Figure 2B, the substrate scope for the defluorination/elimination process was explored. When using symmetric substrates, the desired acyclic monofluoroalkenes (**3a**, **3e**-**g**, **3j**-**k**, **3n**-**o**, **3u**, and **3aa**) were prepared in moderate to good yields (up to 84%) with good *Z*/*E* stereocontrol. Specifically, substrates with electron-donating substituents such as methyl, methoxy, or dialkyl groups on the benzene ring gave the desired products (**3e**-**g** and **3n**-**o**) in moderate to good yields (51%–70%) in refluxing 1,4-difluorobenzene. Halogen groups were well tolerated in the elimination transformation, although an unexpected decrease in yield (38%) was observed for the preparation of bromo-substituted **3j**, albeit that the stereocontrol was high (*Z*/*E* = 25:1). In addition, benzylic *gem*-difluoroalkanes afforded **3bb** and **3cc** in merely low to moderate yields (41% and 25%, respectively), and only the *Z*-isomer is formed, even though the fluorinated moiety is located at the activated position and was thus expected to be removed more easily. For cyclic *gem*-difluoroalkanes (Strobach and Boswell, 1971), the formation of six-membered substrates was favored, i.e., **3dd** and **3ee** were prepared in 80% and 64% yield, respectively. Furthermore, the defluorination of large-ring-type *gem*-difluoroalkanes proceeded smoothly to afford the corresponding cyclic monofluoroalkenes **3gg** and **3hh** in good yields, albeit that the *Z*/*E* selectivity was low.

### B(C_6_F_5_)_3_ Catalyzed Friedel-Crafts Reactions of Secondary Aliphatic Fluorides

**Figure 3:**
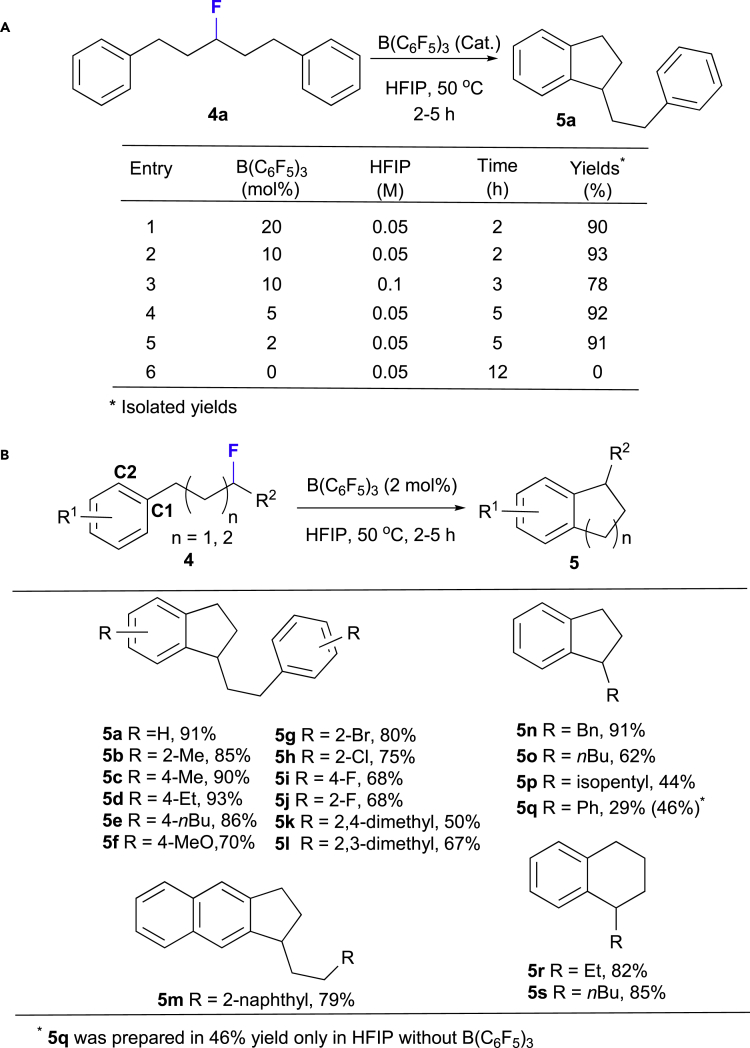
Intramolecular Friedel-Crafts Cyclization of Secondary Monofluoroalkanes **4** (A) Optimization of reaction conditions. (B) Substrate Scope.

Although the cleavage of the C(sp^3^)-F bond in unactivated aliphatic monofluorides was expected to be easier than in the corresponding saturated *gem-*difluoroalkanes, the Friedel-Crafts alkylation of secondary monofluorinated alkanes was less successful (Hamel and Paquin, 2018, Stahl et al., 2013). Under the previously established optimal reaction conditions for the synthesis of spirobiindanes **2**, using 20 mol % B(C_6_F_5_)_3_ in HFIP, an intramolecular defluorinative cyclization of secondary fluorocarbon **4a** was observed in good yield (90%; Figure 3A, entry 1). Subsequently, upon decreasing the catalyst loading to 2 mol %, the desired 1-phenethyl-2,3-dihydro-1H-indene (**5a**) was smoothly prepared (91%; Figure 3A, entry 5). However, in the absence of a Lewis-acidic catalyst, a reaction was not observed (Figure 3A, entry 6), which demonstrates the crucial importance of B(C_6_F_5_)_3_ for abstraction of fluoride. Subsequently, we explored the substrate scope (Figure 3B) of this reaction. With long-chain symmetric substrates with electron-donating groups (**4a**-**f**), good to high yields were observed (70%–93%). Conversely, yields for the intramolecular Friedel-Crafts transformation of halogen-substituted monofluoroalkanes **5g**-**j** were a bit lower (68%–80%). Similarly, 2,3-dimethyl- and 2,4-dimethyl-substituted **4k** and **4l** were converted into the cyclic products **5k** and **5l** in 50% and 67% yield, respectively, whereas the naphthyl-type product **5m** was obtained in 79% yield. Miscellaneous monofluorides **4n**-**p** furnished the desired alkyl-substituted indanes in moderate to good yields (44%–91%). Six-membered ring products **5r** and **5s** were also prepared in 82%–85% yield. However, benzylic secondary monofluoride **4q** furnished 1-phenyl-2,3-dihydro-1H-indene (**5q**) in merely 29% yield. It should be noted that an increased yield (46%) for the synthesis of **5q** was observed in the absence of a Lewis acid catalyst. Although intermolecular Friedel-Crafts alkylations of primary benzylic monofluoride using excess amounts of electrophiles in HFIP in the absence of acids have already been reported (Champagne et al., 2014), we have observed the first example of the functionalization of a secondary benzylic monofluoride such as **4q** in the absence of any catalyst or additive (Figure 3B).

### Mechanistic Investigations

We found that the donor-acceptor interactions between the fluorine moiety of C(sp^3^)-F bonds in unactivated aliphatic *gem*-difluoroalkanes, a weak Lewis base, and the strong Lewis acid B(C_6_F_5_)_3_, is of vital importance; this is emphasized by the overwhelming chemoselectivity for the Friedel-Crafts cyclization of C(sp^3^)-F bonds rather than the cleavage of weaker C-halogen bonds or the removal of other good leaving groups (Table 2). Specifically, when using a stoichiometric amount of B(C_6_F_5_)_3_ (2.2 equiv.) in CH_2_Cl_2_ at room temperature for 30 h, *gem*-difluoroalkane **1a** afforded only the desired 2,2′,3,3′-tetrahydro-1,1′-spirobi[indene] **2a** in 85% yield, whereas the formation of elimination product **3a** was not observed. In contrast, the intramolecular Friedel-Crafts cyclization was not observed when using 1,5-diphenylpentan-3-one (**1v**), (3,3-dimethoxypentane-1,5-diyl)dibenzene (**1w**), (3,3-dichloropentane-1,5-diyl)dibenzene (**1x**), and (3,3-dibromopentane-1,5-diyl)dibenzene (**1y**) (Table 2, entries 1–5). Although the cleavage of C-OMe bonds of substrate **1w** by B(C_6_F_5_)_3_ (2.2 equiv.) was observed, as the electron-rich heteroatom is a good Lewis base, only a complex mixture was found, and the formation of **2a** was not observed (Table 2, entry 3). For the *gem*-dibromoalkane **1y**, the formation of an unexpected elimination product in 29% yield was detected, which was ascribed to the ability of the bromine to act as a good leaving group (Table 2, entry 5). In addition, under optimized conditions of HFIP, the formation of C(sp^3^)-C(sp^3^) bonds was only detected for *gem*-difluoroalkane **1a**, but not for the relatively weaker C(sp^3^)-OMe, C(sp^3^)-Cl, and C(sp^3^)-Br bonds (Table 2, entries 6–12). However, the unexpected elimination products (monochloroalkene **3x** and monobromoalkene **3y**) were formed in of 63% and 81% yield, respectively (Table 2, entry 9 and 11). Interestingly, in the absence of B(C_6_F_5_)_3_ but still using HFIP as solvent, the yields of elimination products **3x** and **3y** remained essentially unchanged (61% and 86%, respectively). In other words, it is the hydrogen-bonding interaction between HFIP and either the C(sp^3^)-Cl or C(sp^3^)-Br bonds rather than the interaction with Lewis acids B(C_6_F_5_)_3_ that governs the elimination process. Similarly, under the standard reaction conditions for the defluorinative elimination of **1a**, i.e., treatment with B(C_6_F_5_)_3_ (20 mol %) in refluxing 1,4-difluorobenzene for 24 h, *gem*-difluoride **1a** afforded the desired monofluorinated olefin **3a** in 87% yield, whereas a reaction was not observed for the corresponding aliphatic halides and ketals, with the exception of (3,3-dibromopentane-1,5-diyl)dibenzene, which afforded the monobromoalkene elimination product in 9% yield (Table 2, entries 13–16). Therefore the synthesis of spirobiindanes and monofluoroalkenes from aliphatic *gem*-difluoroalkanes **1** catalyzed by B(C_6_F_5_)_3_ proceeds from a C-F bond activation process.

**Table 2:** Control Experiments to Probe Reaction Mechanism


Entry	X^a^	Lewis Acids (Equiv.)	Solvent (0.1M)	T (^o^C)	t (h)	Yields (%)	
						**2a**	**3a**^b^
1	F	B(C_6_F_5_)_3_ (2.2)	CH_2_Cl_2_	RT	30	85	0
2	**1v** (C=O)	B(C_6_F_5_)_3_ (2.2)	CH_2_Cl_2_	RT	30	NR	NR
3	MeO	B(C_6_F_5_)_3_ (2.2)	CH_2_Cl_2_	RT	30	ND^c^	ND^c^
4	Cl	B(C_6_F_5_)_3_ (2.2)	CH_2_Cl_2_	RT	30	NR	NR
5	Br	B(C_6_F_5_)_3_ (2.2)	CH_2_Cl_2_	RT	30	0	29
6	F	B(C_6_F_5_)_3_ (0.2)	(CF_3_)_2_CHOH^d^	50	2	84	0
7	**1v** (C=O)	B(C_6_F_5_)_3_ (0.2)	(CF_3_)_2_CHOH^d^	50	2	NR	NR
8	MeO	B(C_6_F_5_)_3_ (0.2)	(CF_3_)_2_CHOH^d^	50	2	NR	NR
9	Cl	B(C_6_F_5_)_3_ (0.2)	(CF_3_)_2_CHOH^d^	50	2	0	63
10	Cl	–	(CF_3_)_2_CHOH^d^	50	2	0	61
11	Br	B(C_6_F_5_)_3_ (0.2)	(CF_3_)_2_CHOH^d^	50	2	0	81
12	Br	–	(CF_3_)_2_CHOH^d^	50	2	0	86
13	F	B(C_6_F_5_)_3_ (0.2)	*p*-C_6_H_4_F_2_	reflux	24	0	87
14	MeO	B(C_6_F_5_)_3_ (0.2)	*p*-C_6_H_4_F_2_	reflux	24	0	0
15	Cl	B(C_6_F_5_)_3_ (0.2)	*p*-C_6_H_4_F_2_	reflux	24	0	Trace
16	Br	B(C_6_F_5_)_3_ (0.2)	*p*-C_6_H_4_F_2_	reflux	24	0	9

NR, no reaction; RT, room temperature; ND, not detected.

a Substrates: 1,5-diphenylpentan-3-one (**1v**), (3,3-dimethoxypentane-1,5-diyl)dibenzene (**1w**), (3,3-dichloropentane-1,5-diyl)dibenzene (**1x**), and (3,3-dibromopentane-1,5-diyl)dibenzene (**1y**).

b NMR yields.

c Complex mixture.

d Concentration (0.05 M).

**Figure 4:**
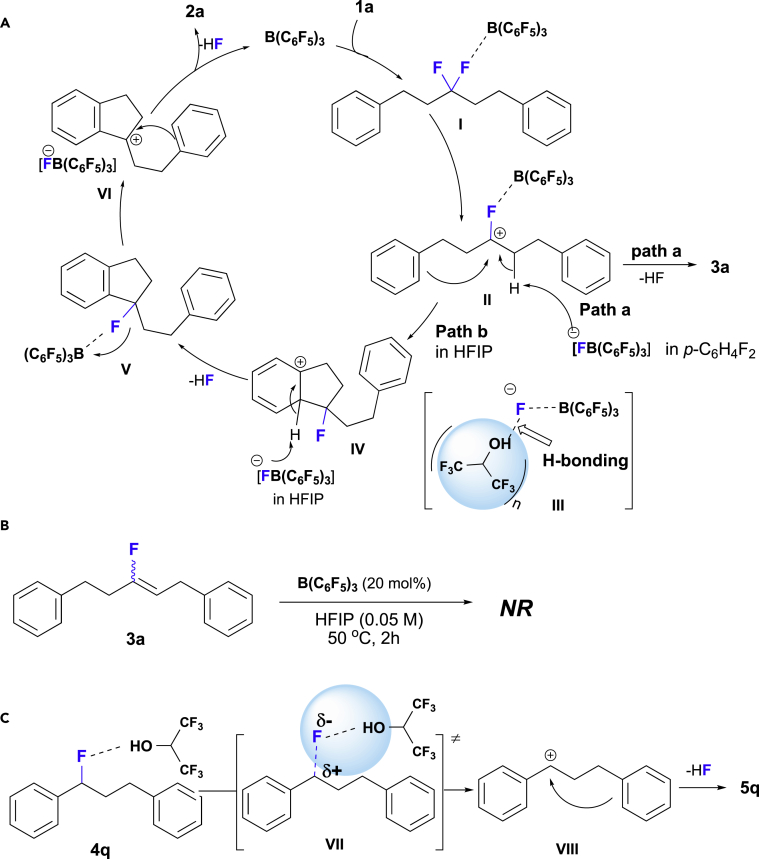
Proposed Reaction Mechanism (A) Plausible reaction pathway for the defluorinative Friedel-Crafts cyclization and defluorinative elimination of *gem*-difluoroalkanes (**1**). (B) To rule out the possibility of affording spirobiindanes **2** via the intermediate of monofluoroalkene **3**. (C) Proposed reaction mechanism for the hydrogen-bonding-induced intramolecular Friedel-Crafts reaction of benzylic monofluoride **4q**.

Based on the results discussed above, a reaction mechanism of C-F bond cleavage induced by the C(sp^3^)-F→B(C_6_F_5_)_3_ interaction is proposed in Figure 4A. In our opinion, two effects of HFIP favor the intramolecular Friedel-Crafts process. (1) The strong hydrogen-bonding interaction between the hydrogen-bonding donor solvent HFIP and the fluoride anion in [FB(C_6_F_5_)_3_]^-^ reduces the Brønsted basicity of the fluoride anion (cf. intermediate **III**) (Lee et al., 2016, Liang et al., 2017), which would result in the suppression of the E1-type elimination. Indeed, it has already been reported that the Lewis basicity of the fluoride anion of CsF or tetrabutylammonium fluoride (TBAF) is decreased in tertiary alcohols or urea (Kim et al., 2006, Kim et al., 2008a, Kim et al., 2008b, Pfeifer et al., 2016). For instance, relative to anhydrous TBAF, the TBAF(*t*-BuOH)_4_ complex significantly favors nucleophilic substitution over elimination pathways (Kim et al., 2008a, Kim et al., 2008b). (2) HFIP, with its high dielectric constant (ε = 15.7) and low nucleophilicity (Colomer et al., 2017), provides additional stabilization for several carbocation intermediates in the intramolecular Friedel-Crafts alkylation (e.g., Figure 4B, **II**, **IV,** and **VI**). In addition, the alternative and probable reaction pathway via further defluorinative cyclization of monofluoroalkene **3** with a C(sp^2^)-F bond was ruled out as shown in Figure 4B. This result also indicates that the selective C-F bond activation by B(C_6_F_5_)_3_ is limited to C(sp^3^)-F bonds. For benzylic secondary monofluoride (1-fluoropropane-1,3-diyl)dibenzene (**4q**), the hydrogen bonding between the benzylic C(sp^3^)-F bond and HFIP could enable the heterolytic cleavage of the C-F bond to generate carbonium ion **VIII** in Figure 4C, followed by the formation of the C(sp^3^)-C(sp^3^) bond. Accordingly, it is reasonable to extrapolate that in the intramolecular cyclization of aliphatic *gem*-difluoroalkanes **1**, the hydrogen-bond interaction enhances the ability of the fluoride to act as a leaving group, thus promoting the generation of carbonium ion **II** via the removal of a fluoride anion at relative low reaction temperatures. Indeed, without HFIP, higher temperatures were beneficial for the defluorinative elimination to generate monofluoroalkene **3**; the yield of **3a** increases from 64% to 81% when the temperature is increased from 160°C to 220°C in 1,2-dichlorobenznene in a sealed tube (Table 1, entries 14 and 17). Therefore, a combination of the hydrogen-bonding donor solvent HFIP and a catalytic amount of B(C_6_F_5_)_3_ promotes the cascade intramolecular Friedel-Crafts reactions of *gem*-difluorides **1**. It also should be pointed out that the HF generated *in situ* from Friedel-Crafts cyclization might enhance the hydrogen-bonding interaction with C(sp^3^)-F bonds to improve further the ability of fluoride moiety to act as a leaving group, which would benefit the heterolytic cleavage of C(sp^3^)-F bonds induced by B(C_6_F_5_)_3_. Indeed, the intermolecular Friedel-Crafts reaction of primary benzylic monofluoride was controlled only by hydrogen-bonding effect initiated by HFIP and HF generated *in situ* (Champagne et al., 2014).

In conclusion, the selective cleavage of C(sp^3^)-F bonds in unactivated aliphatic *gem*-difluoroalkanes **1** afforded substituted spirobiindanes **2** and monofluoroalkenes **3** in good yields. In addition, the intramolecular Friedel-Crafts cyclization of aliphatic secondary monofluoroalkanes **4** was also described. The C(sp^3^)-F→B(C_6_F_5_)_3_ interaction was probed by control experiments by the use of the corresponding ketone, ketal, and other halide-substituted derivatives. Accordingly, the combination of the hydrogen-bonding donor solvent HFIP and a catalytic amount of the Lewis acid B(C_6_F_5_)_3_ enables the selective functionalization of inert C(sp^3^)-F bonds into C(sp^3^)-C(sp^3^) bonds.

### Limitations of the Study

The substrates with electron-withdrawing groups such as CF_3_ and nitro groups are not suitable, which is to support the Friedel-Crafts cyclization mechanism in Figure 4A. We also examined more reactive iodo-substituted substrates, but complex mixtures were obtained. Although the corresponding F-, Cl-, and Br-substituted substrates are acceptable (**2i**, **2j**, **2k**, **2l,** and **2m**, Figure 2A), these results also show some limitation of this method.

## Methods

All methods can be found in the accompanying Transparent Methods supplemental file.

## Supplemental Information

Document S1. Transparent Methods, Figures S1–S253, Tables S1 and S2
