# Supplementary material for: Activation of Saturated Fluorocarbons to Synthesize Spirobiindanes, Monofluoroalkenes, and Indane Derivatives
Source: iScience. 2019 Jun 19;17:132–43. doi: 10.1016/j.isci.2019.06.018 (PMC6612000; doi:10.1016/j.isci.2019.06.018)
Supplement: Document S1. Transparent Methods, Figures S1–S253, Tables S1 and S2 [file mmc1.pdf]

ISCI, Volume *17*

Supplemental Information

Activation of Saturated Fluorocarbons to Synthesize Spirobiindanes, Monofluoroalkenes, and Indane Derivatives

Jiandong Wang, Yuta Ogawa, and Norio Shibata

Supplemental Figures

Figure S1. ^{1}H NMR spectrum of **2a**, related to **Figure 2**

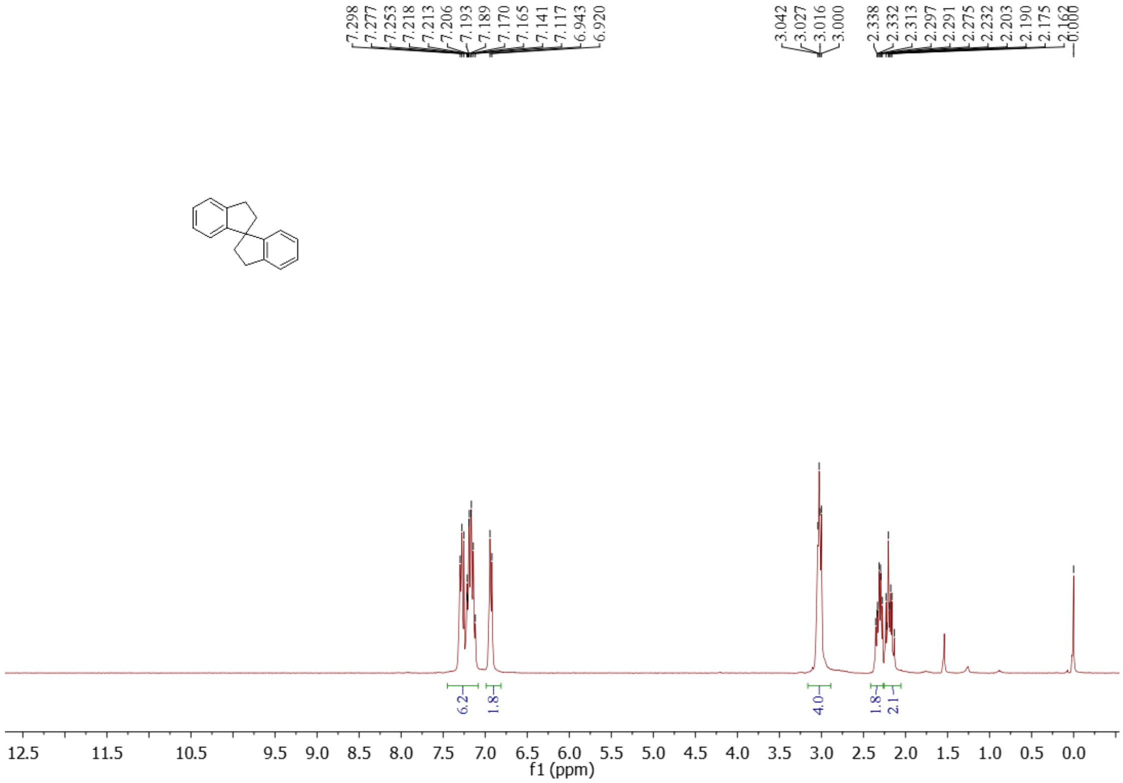

Figure S2. ^{13}C NMR spectrum of **2a**, related to **Figure 2**

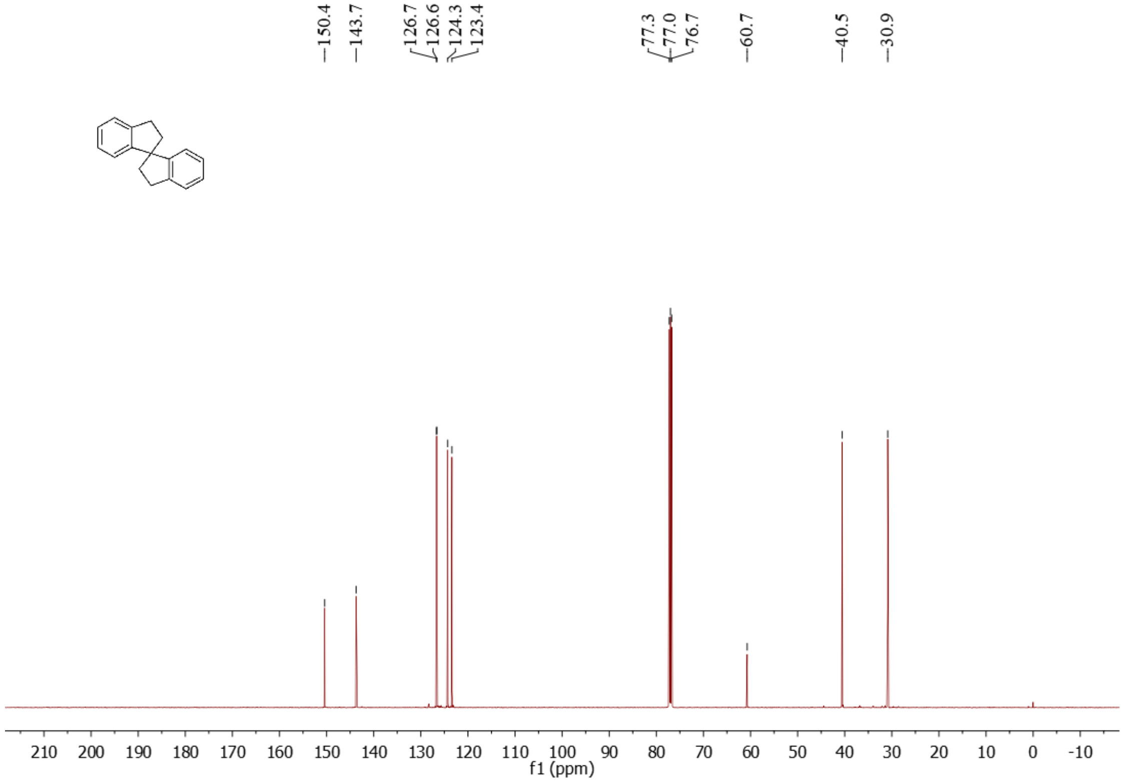

Figure S3. ^{1}H NMR spectrum of **2b**, related to **Figure 2**

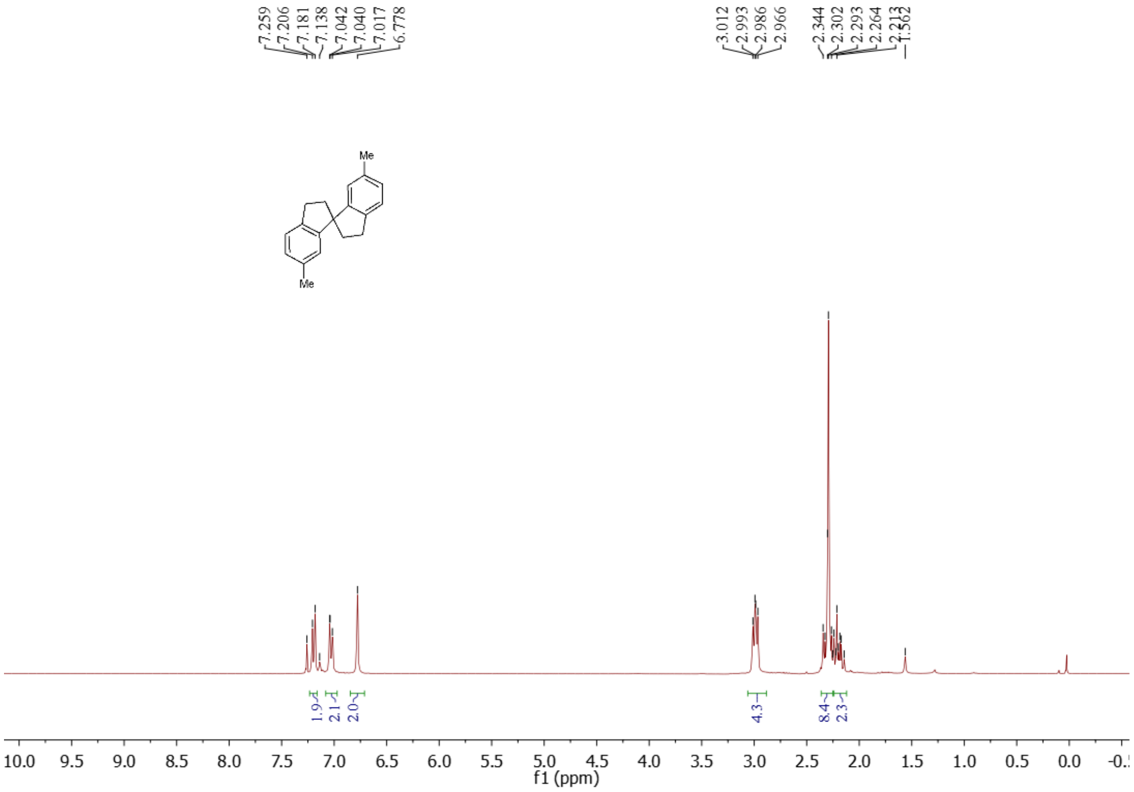

Figure S4. ^{13}C NMR spectrum of **2b**, related to **Figure 2**

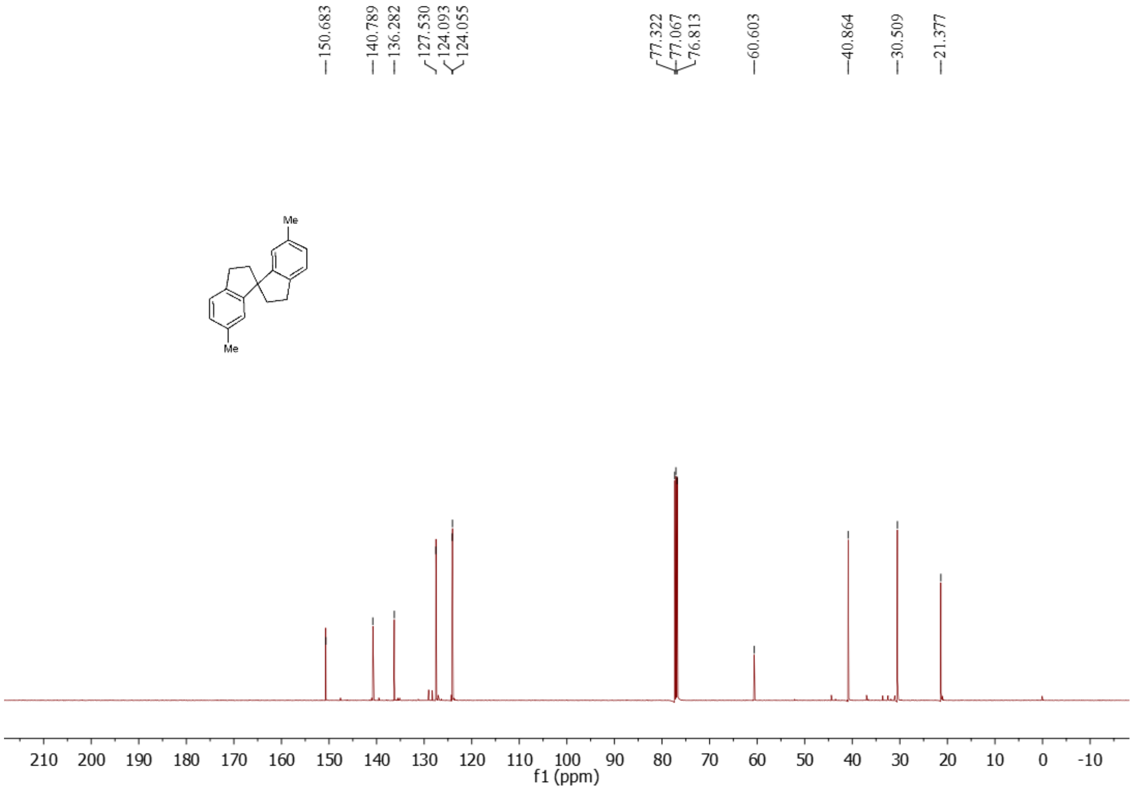

Figure S5. ^{1}H NMR spectrum of **2c**, related to **Figure 2**

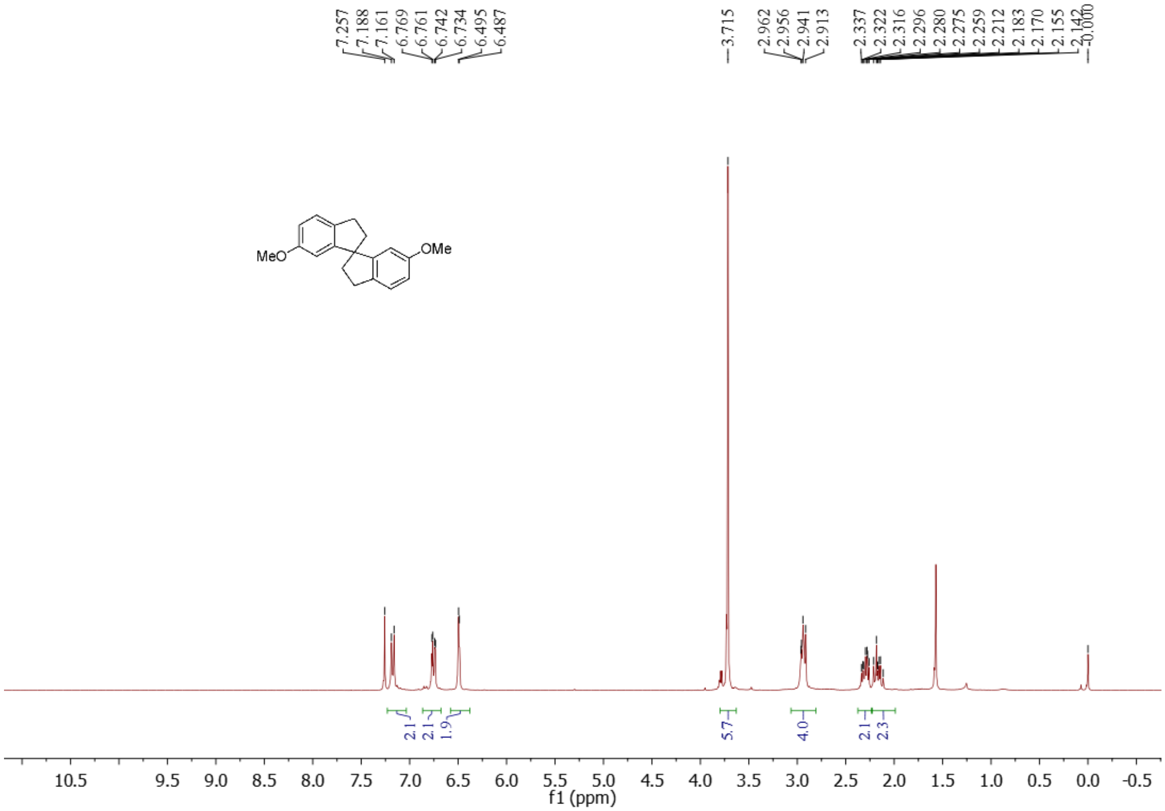

Figure S6. ^{13}C NMR spectrum of **2c**, related to **Figure 2**

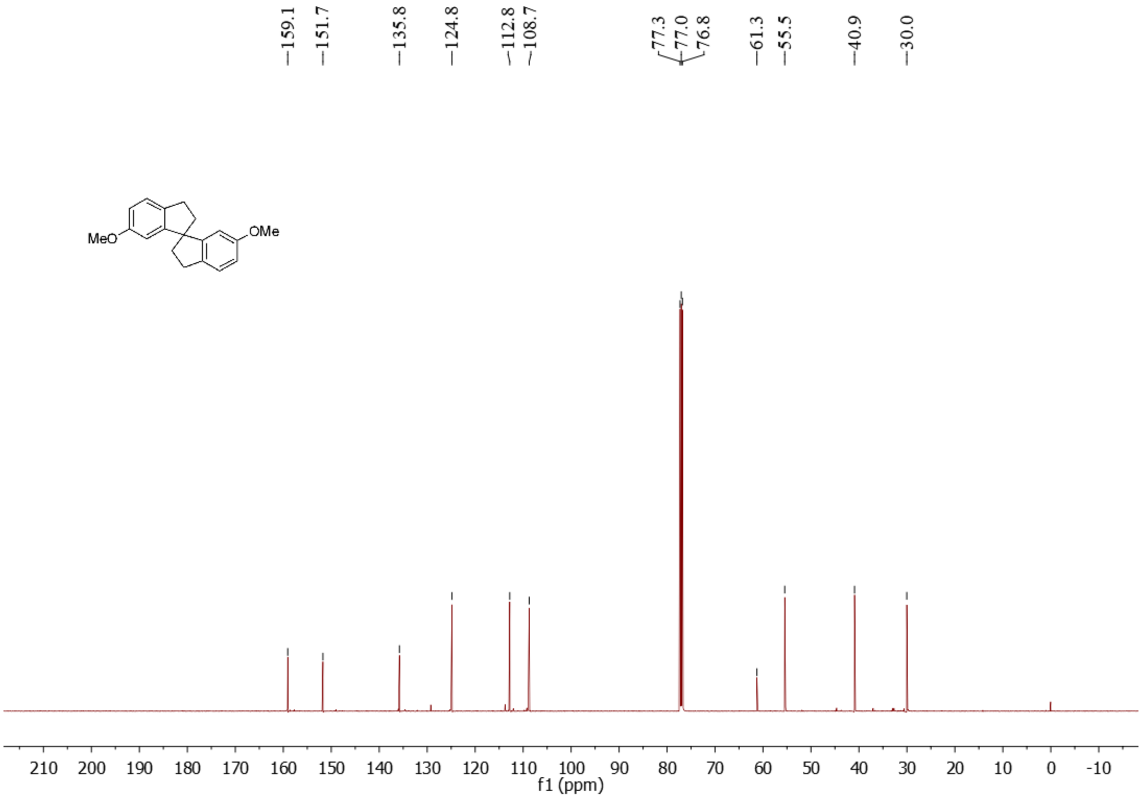

Figure S7. ^{1}H NMR spectrum of **2d**, related to **Figure 2**

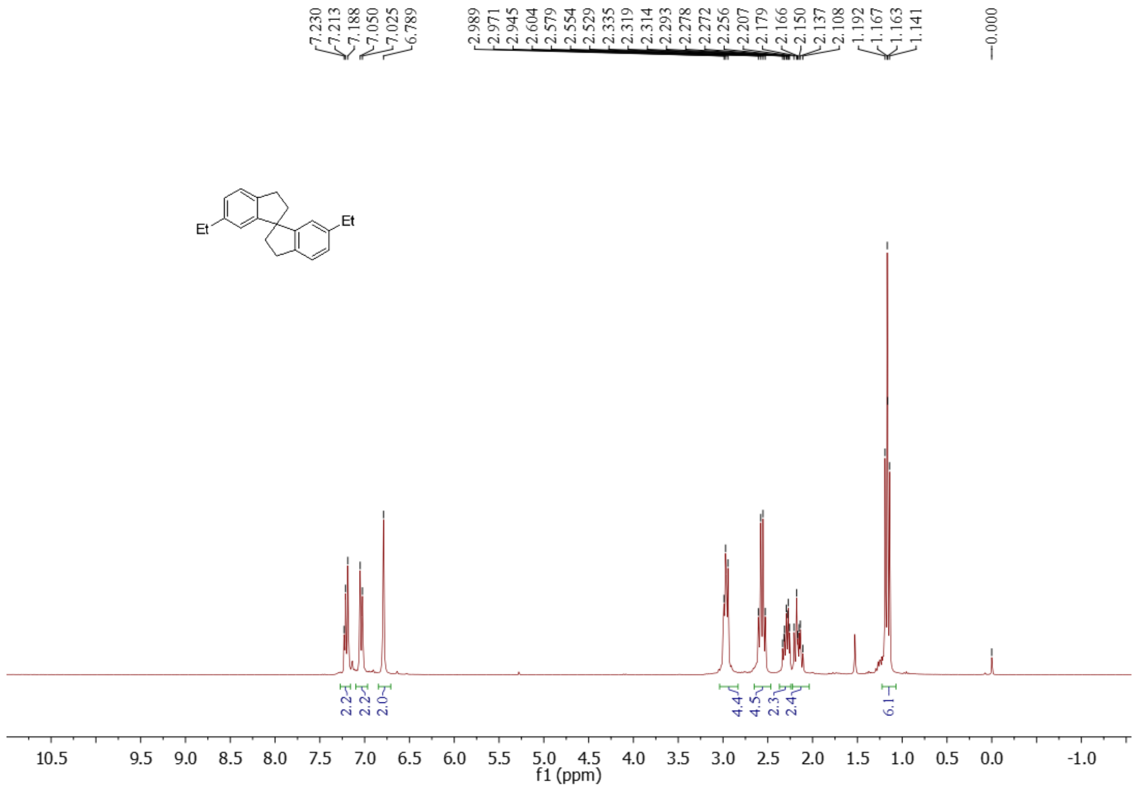

Figure S8. ^{13}C NMR spectrum of **2d**, related to **Figure 2**

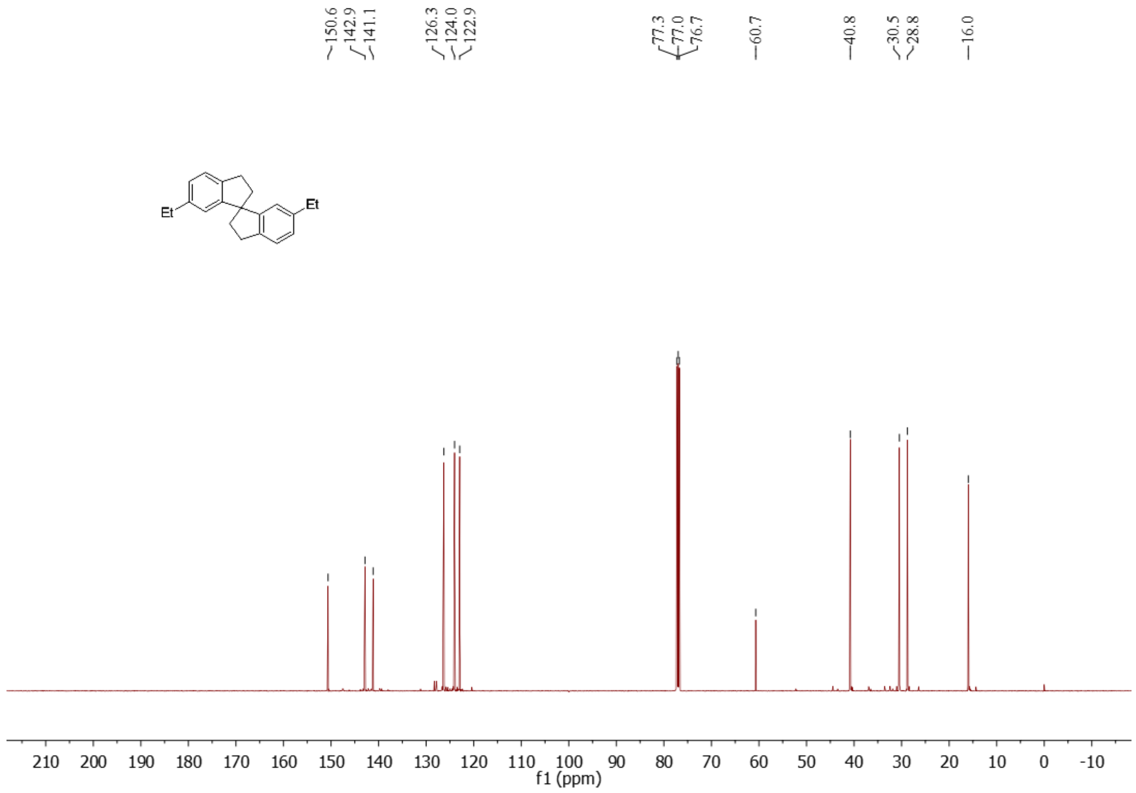

Figure S9. ^{1}H NMR spectrum of **2e**, related to **Figure 2**

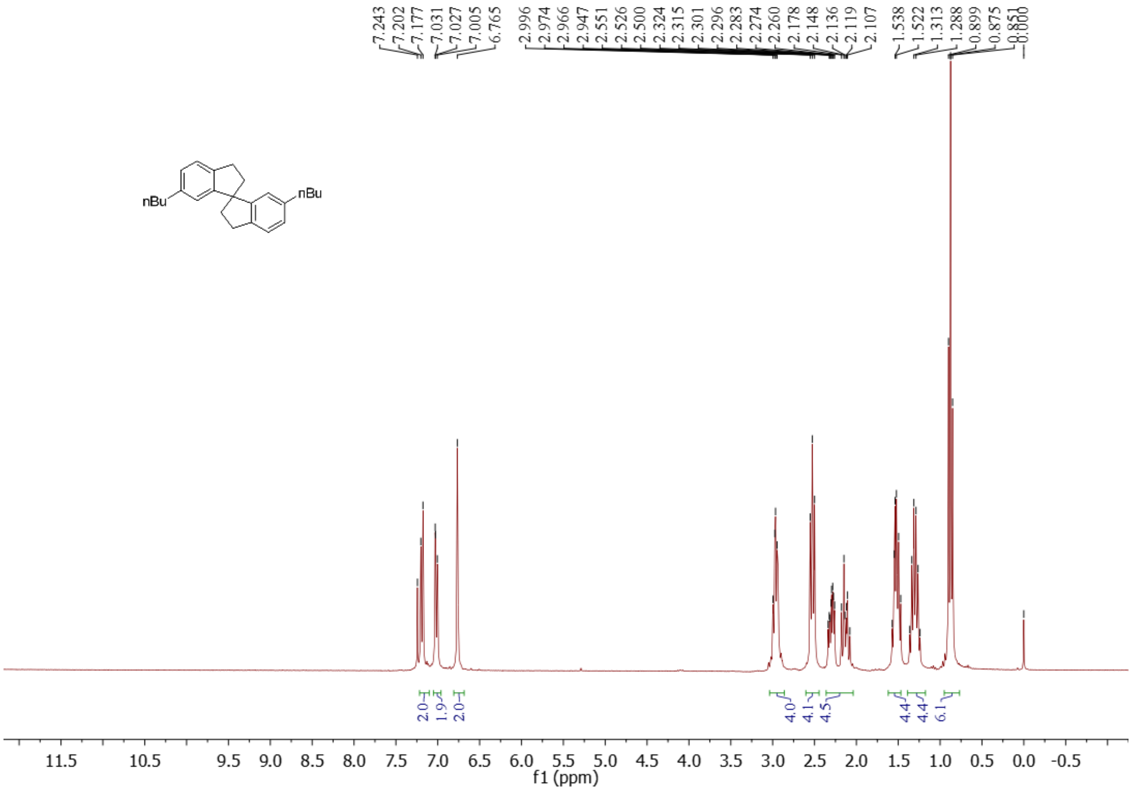

Figure S10. ^{13}C NMR spectrum of **2e**, related to **Figure 2**

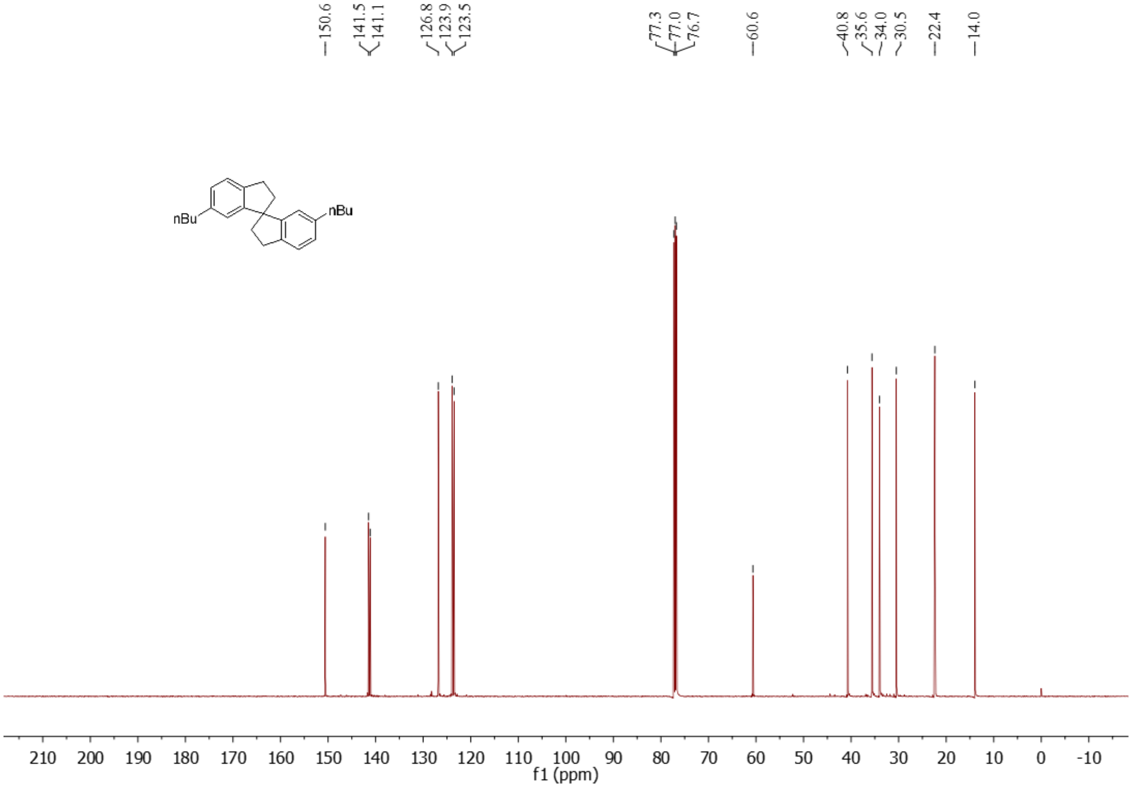

Figure S11. ^{1}H NMR spectrum of **2f**, related to **Figure 2**

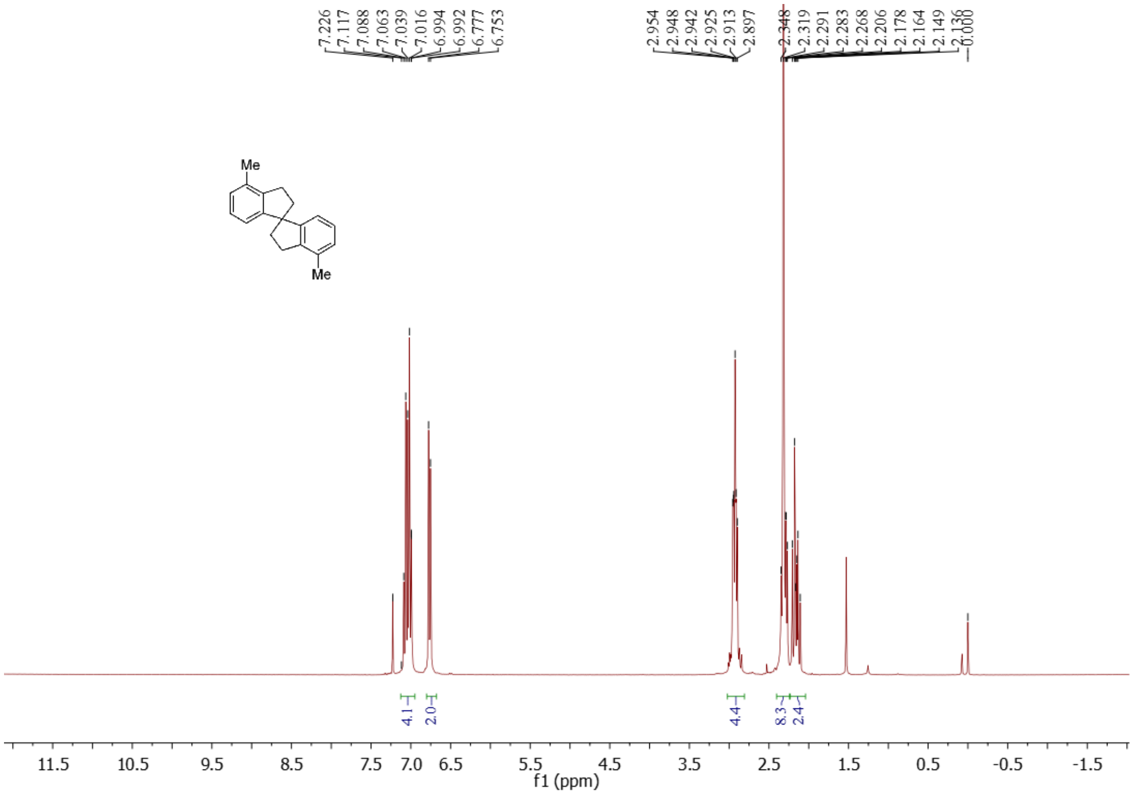

Figure S12. ^{13}C NMR spectrum of **2f**, related to **Figure 2**

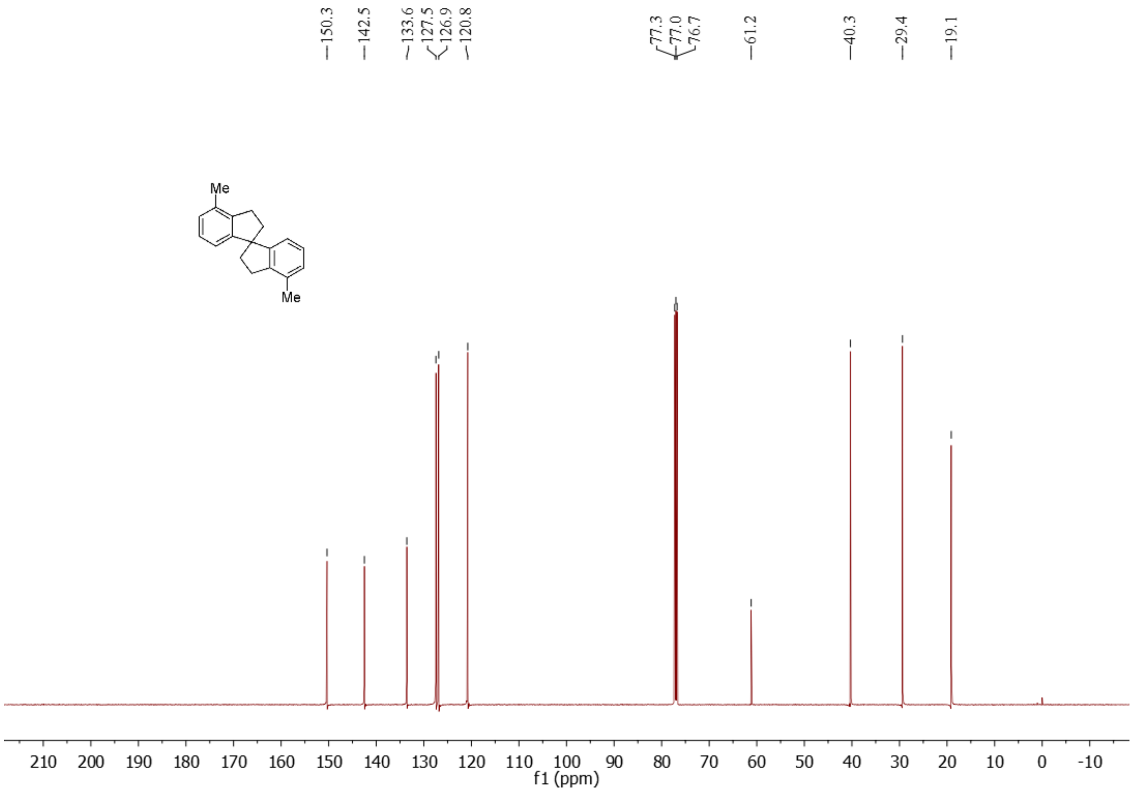

Figure S13. ^{1}H NMR spectrum of **2g**, related to **Figure 2**

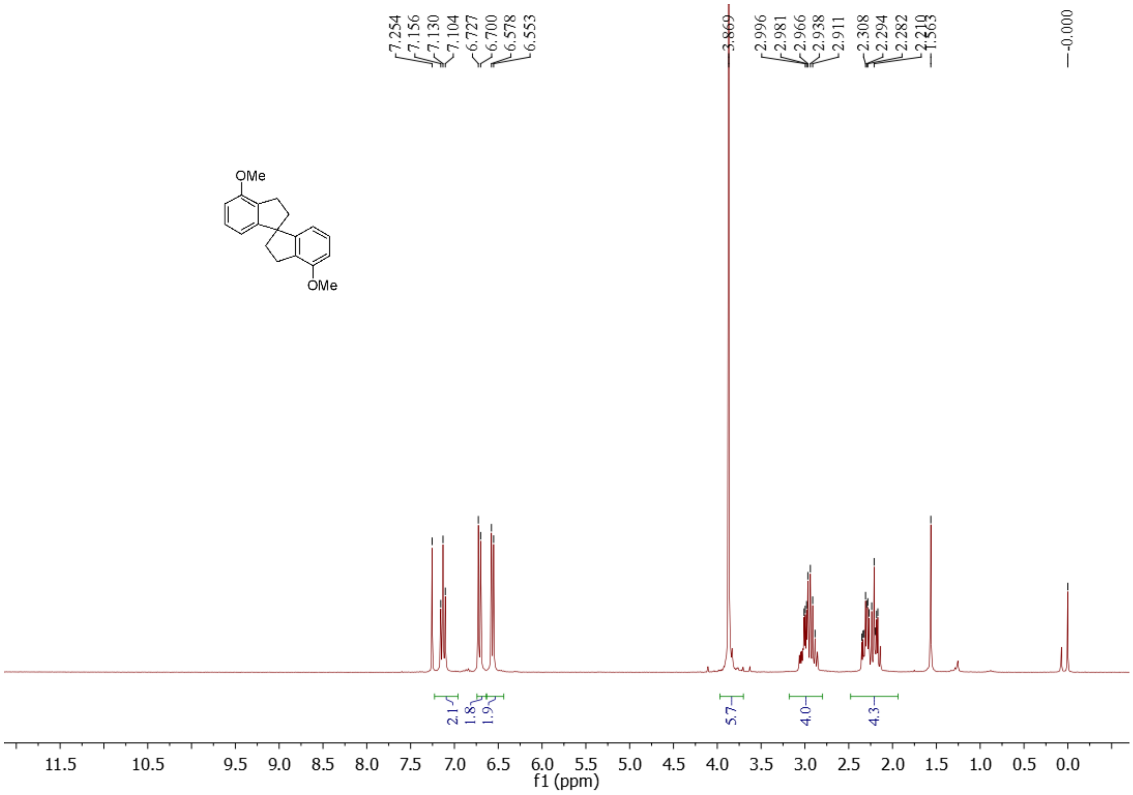

Figure S14. ^{13}C NMR spectrum of **2g**, related to **Figure 2**

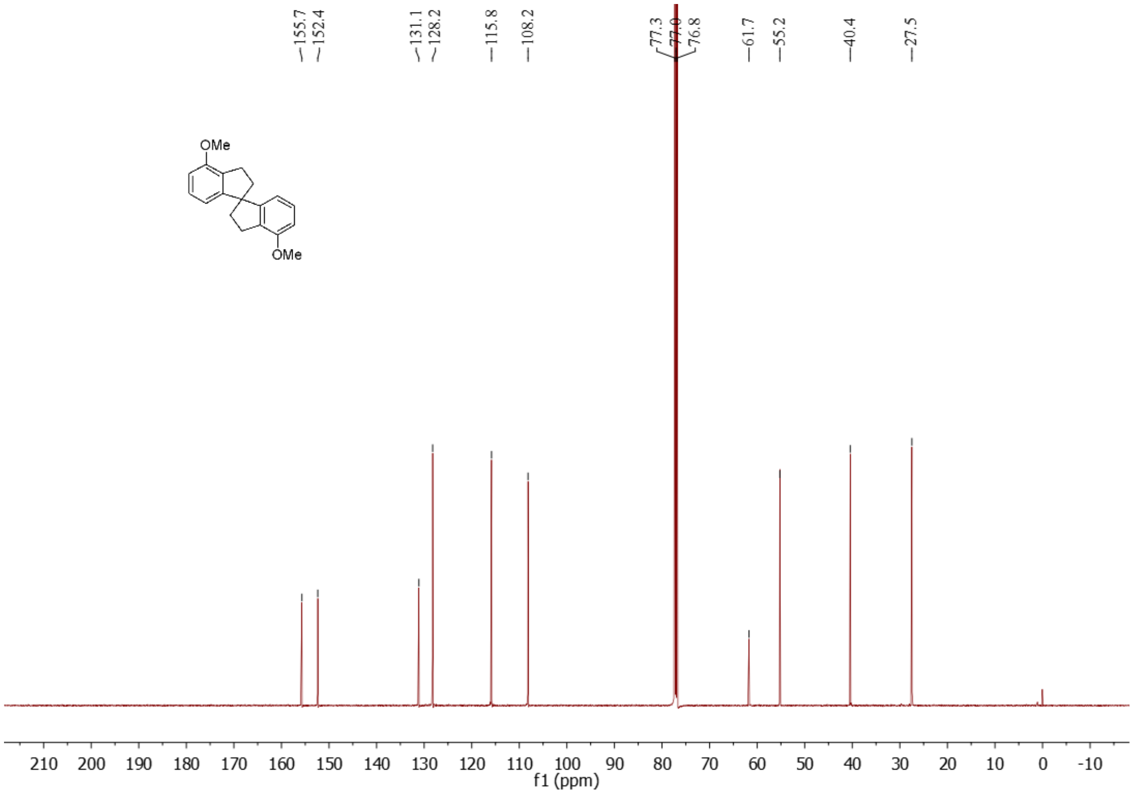

Figure S15. ^{1}H NMR spectrum of **2h**, related to **Figure 2**

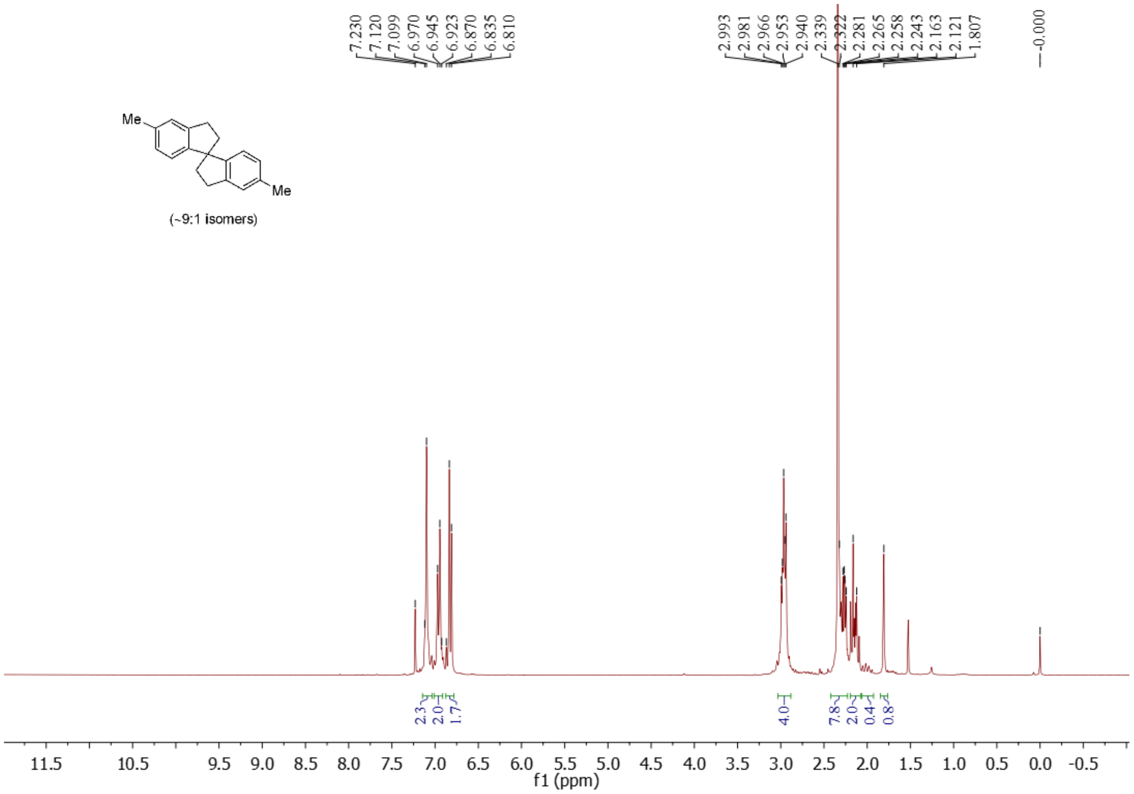

Figure S16. ^{13}C NMR spectrum of **2h**, related to **Figure 2**

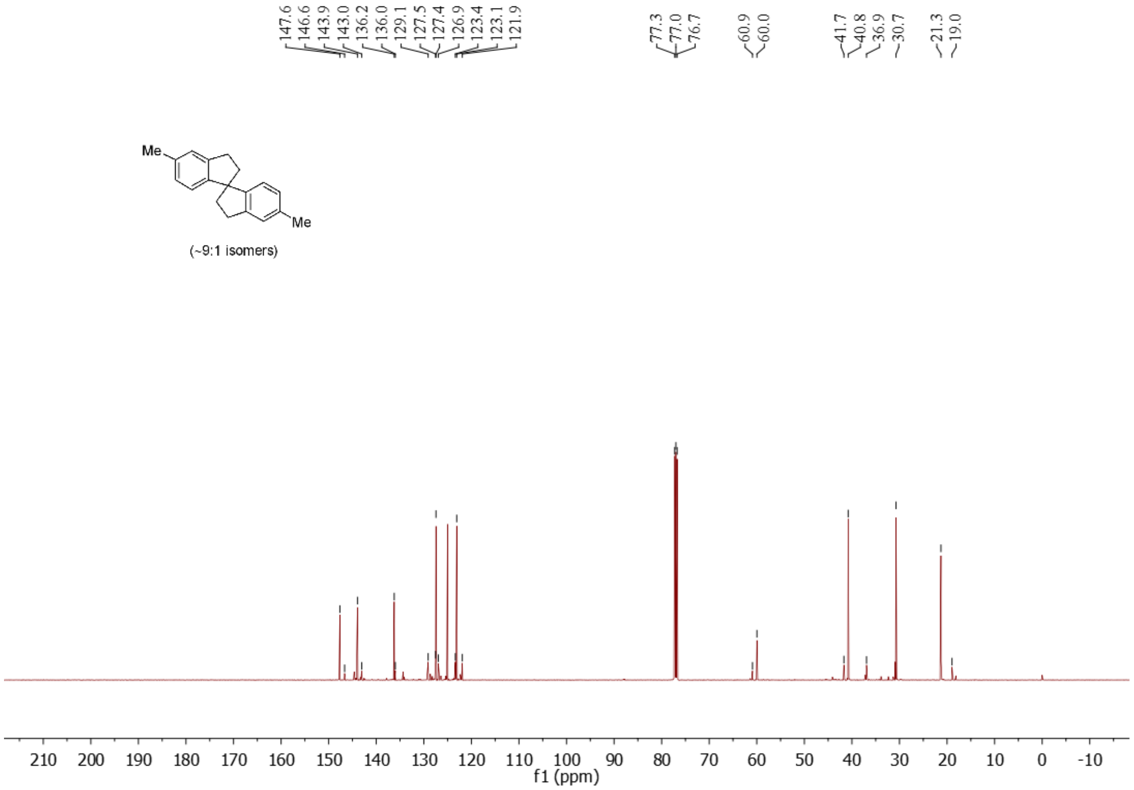

Figure S17. GC-MS analysis of **2h**, related to **Figure 2**

2018/04/13, 14:21:54

C:¥GCMSsolution¥Data¥WANGJIANDONG¥wjd-921.qgd

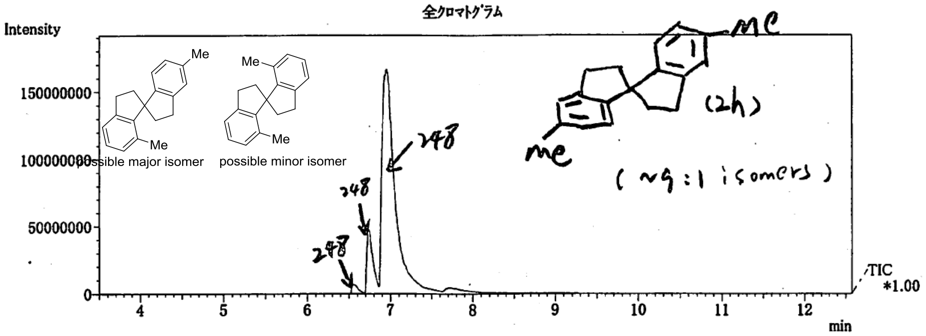

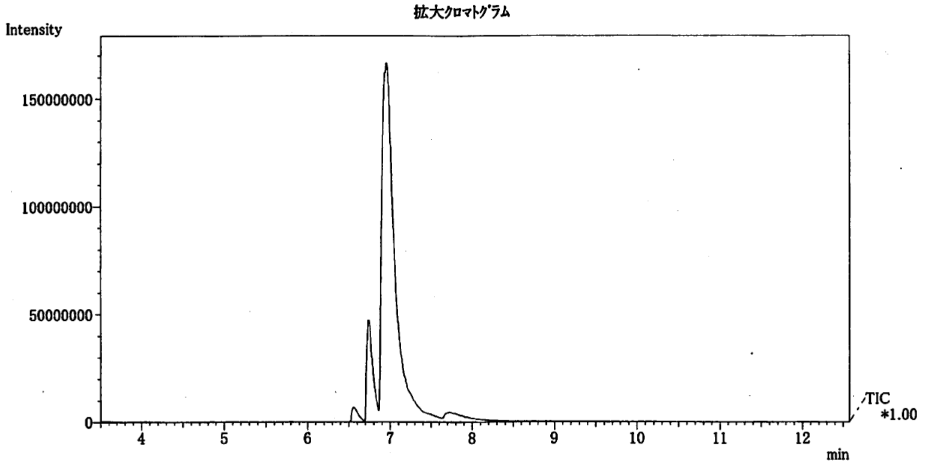

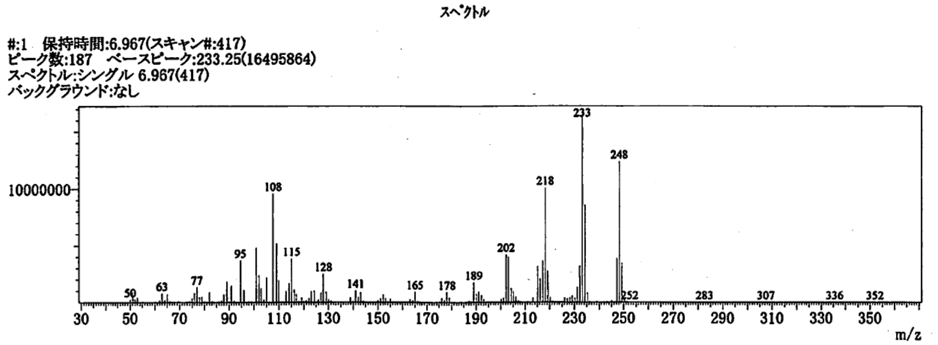

Figure S18. ^{1}H NMR spectrum of **2i**, related to **Figure 2**

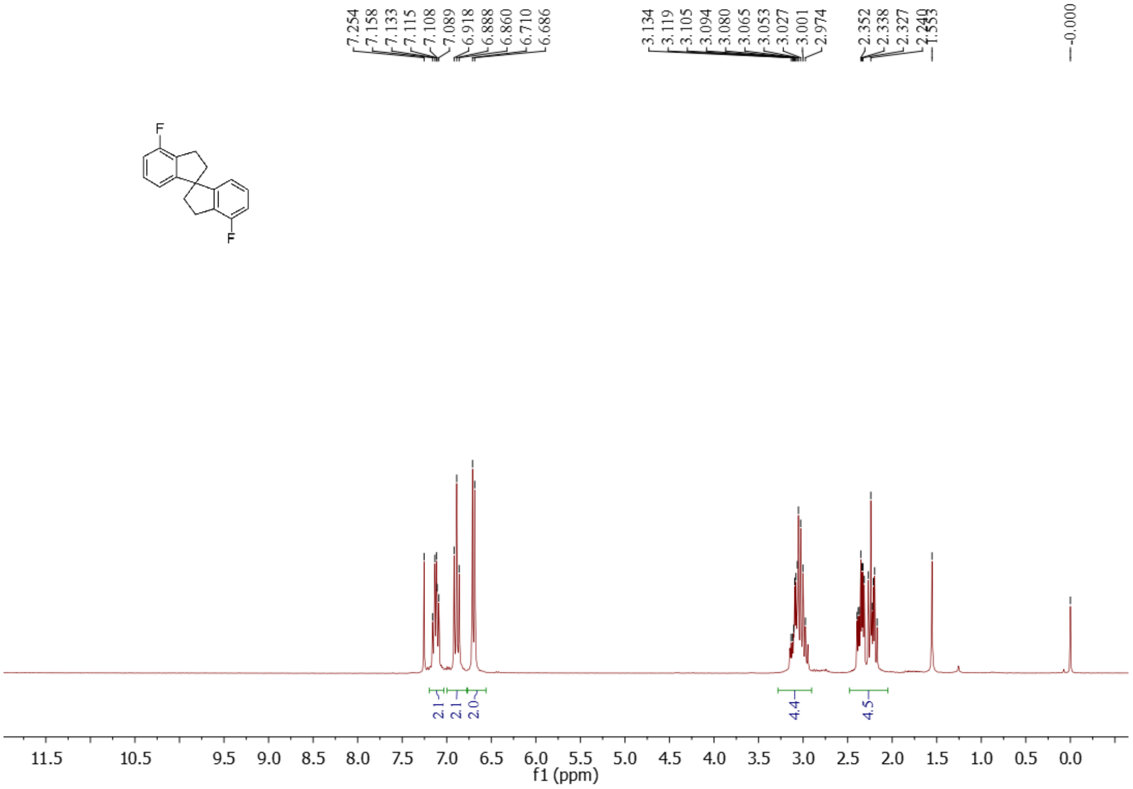

Figure S19. ^{13}C NMR spectrum of **2i**, related to **Figure 2**

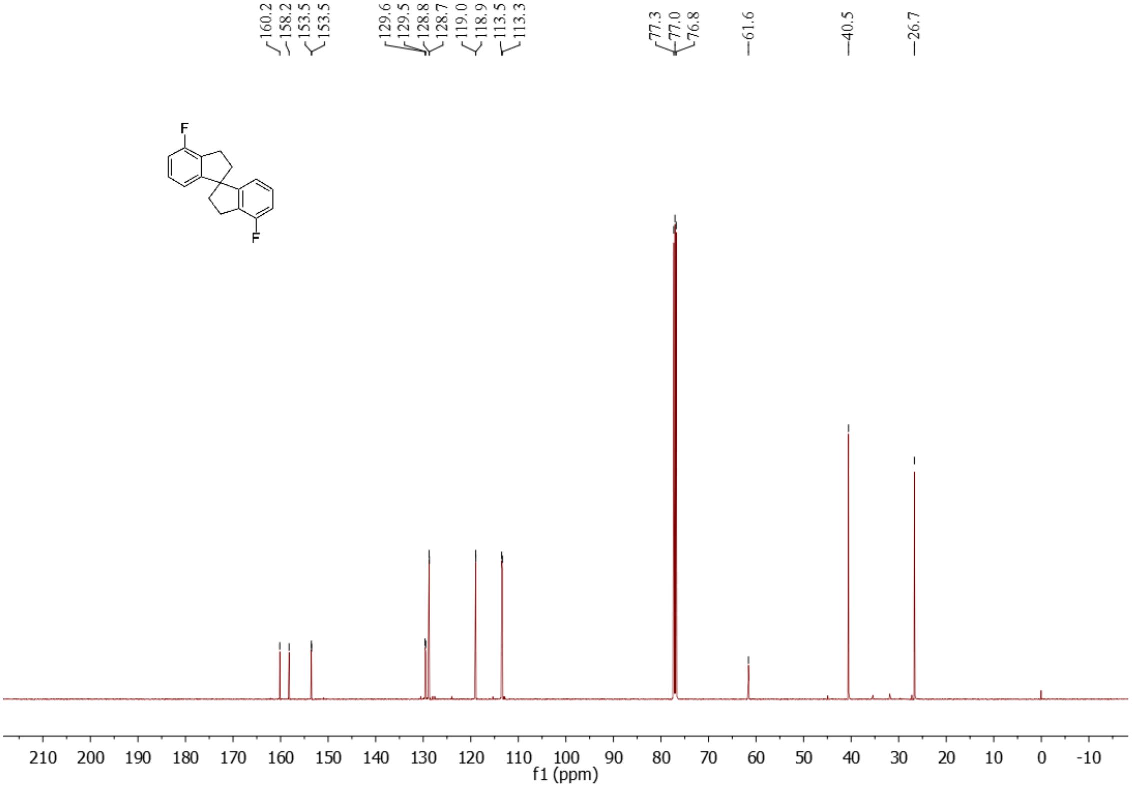

Figure S20. ^{1}H NMR spectrum of **2j**, related to **Figure 2**

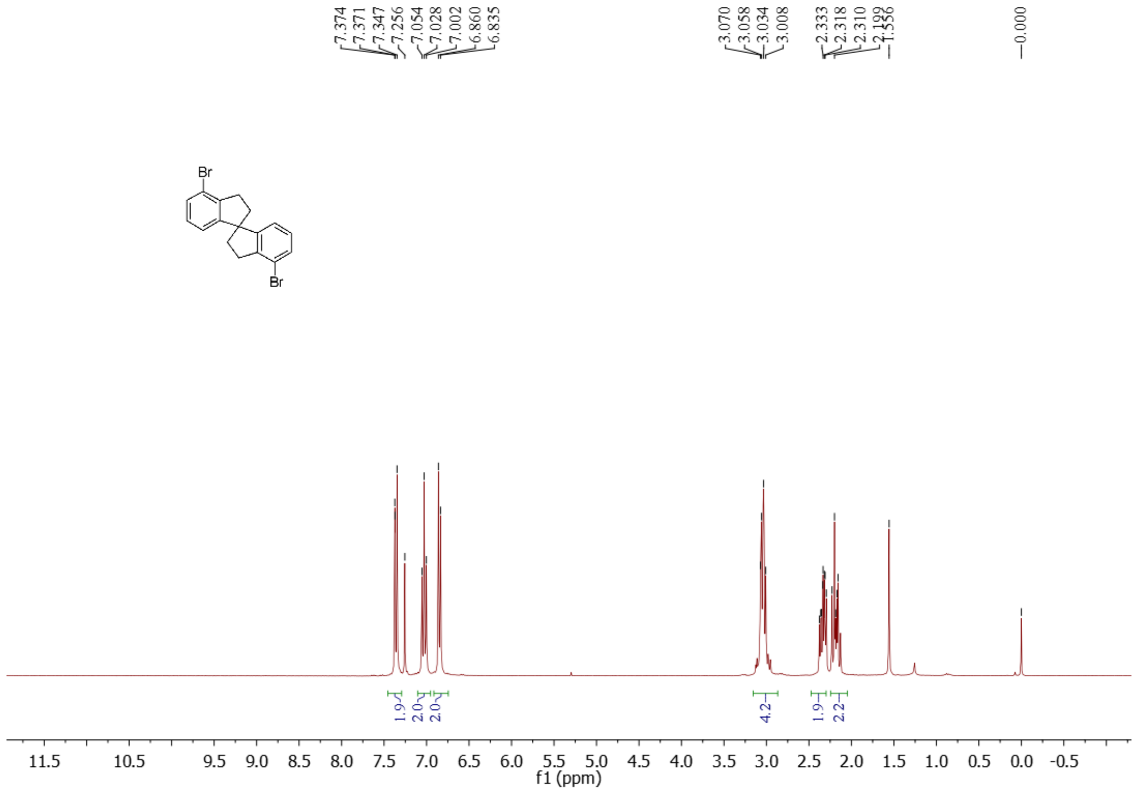

Figure S21. ^{13}C NMR spectrum of **2j**, related to **Figure 2**

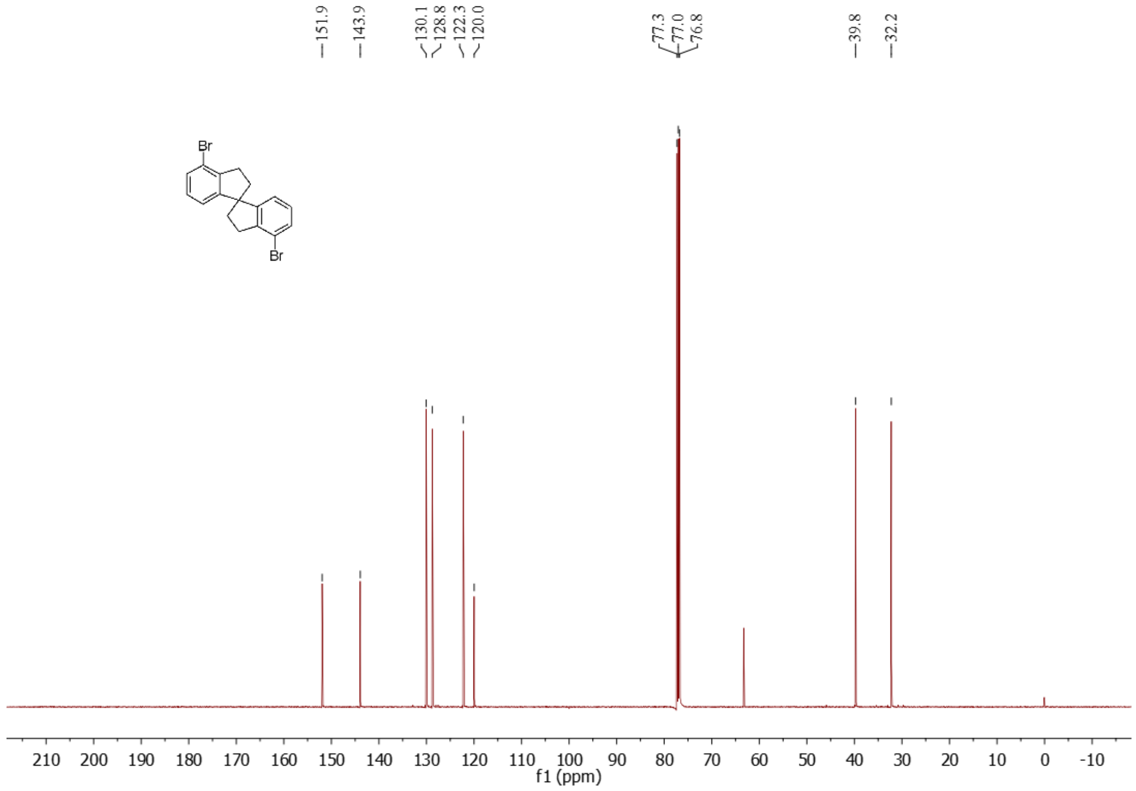

Figure S22. ^{1}H NMR spectrum of **2k**, related to **Figure 2**

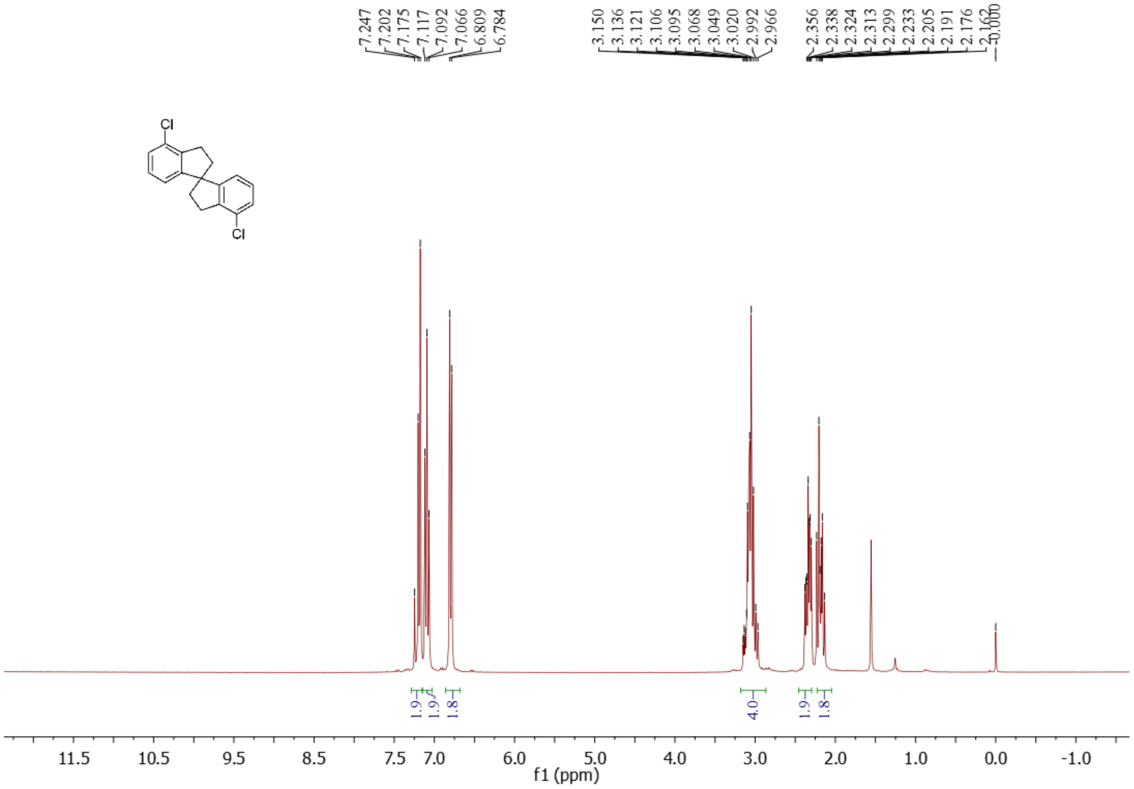

Figure S23. ^{13}C NMR spectrum of **2k**, related to **Figure 2**

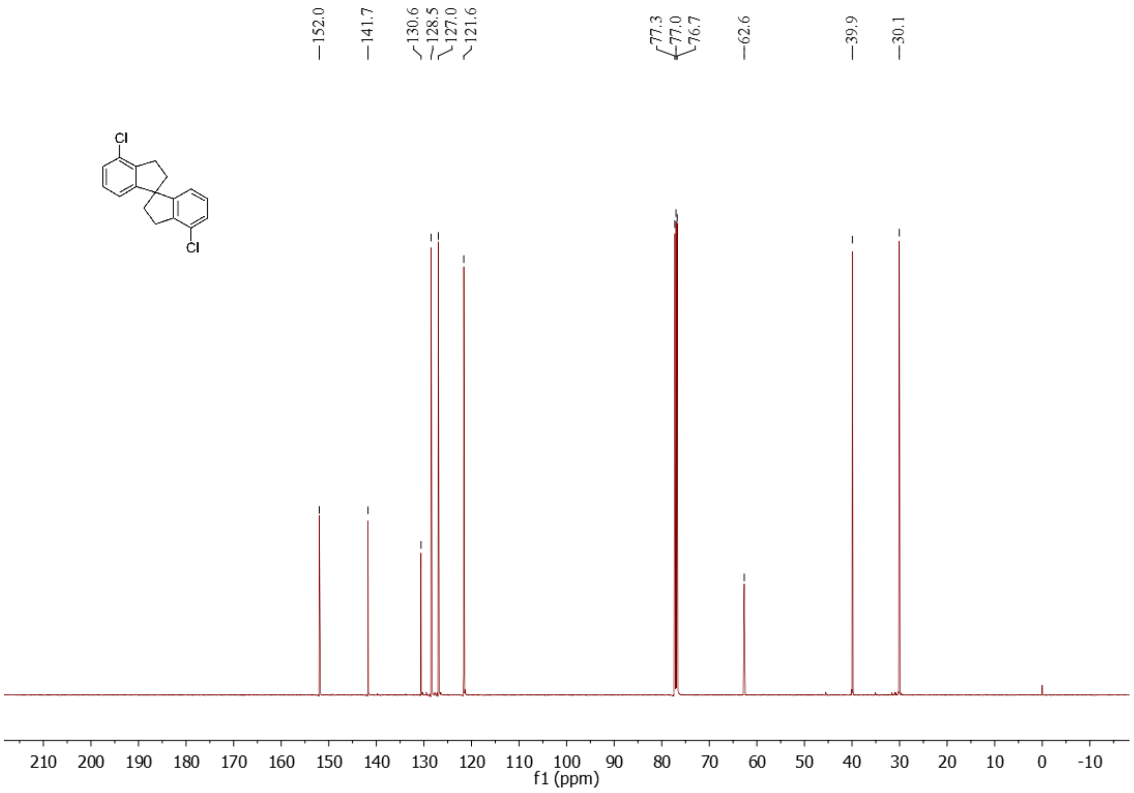

Figure S24. ^{1}H NMR spectrum of **2l**, related to **Figure 2**

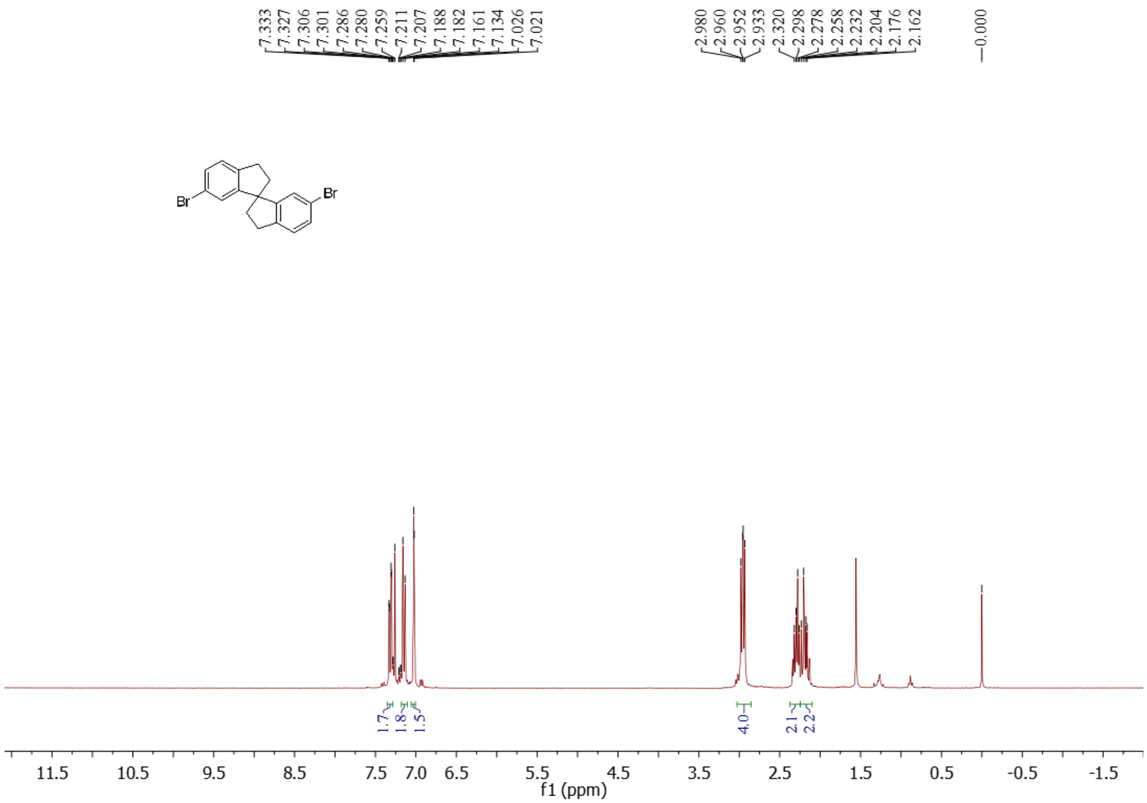

Figure S25. ^{13}C NMR spectrum of **2l**, related to **Figure 2**

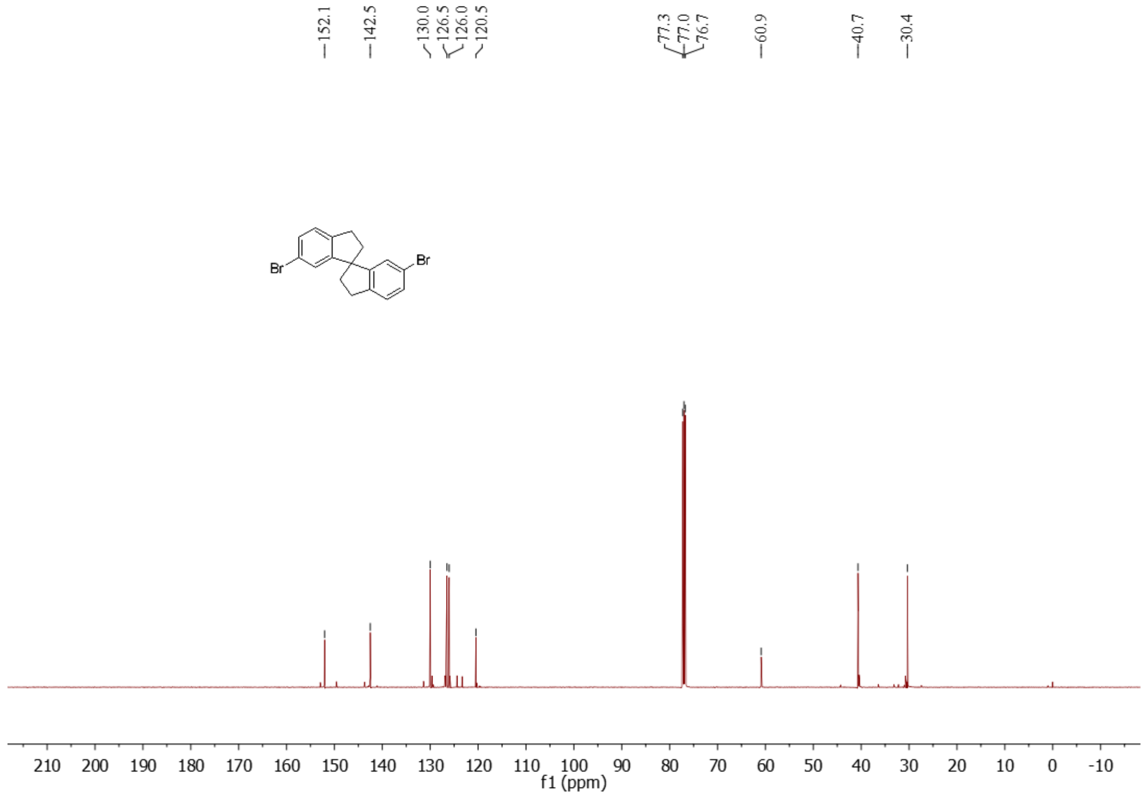

Figure S26. ^{1}H NMR spectrum of **2m**, related to **Figure 2**

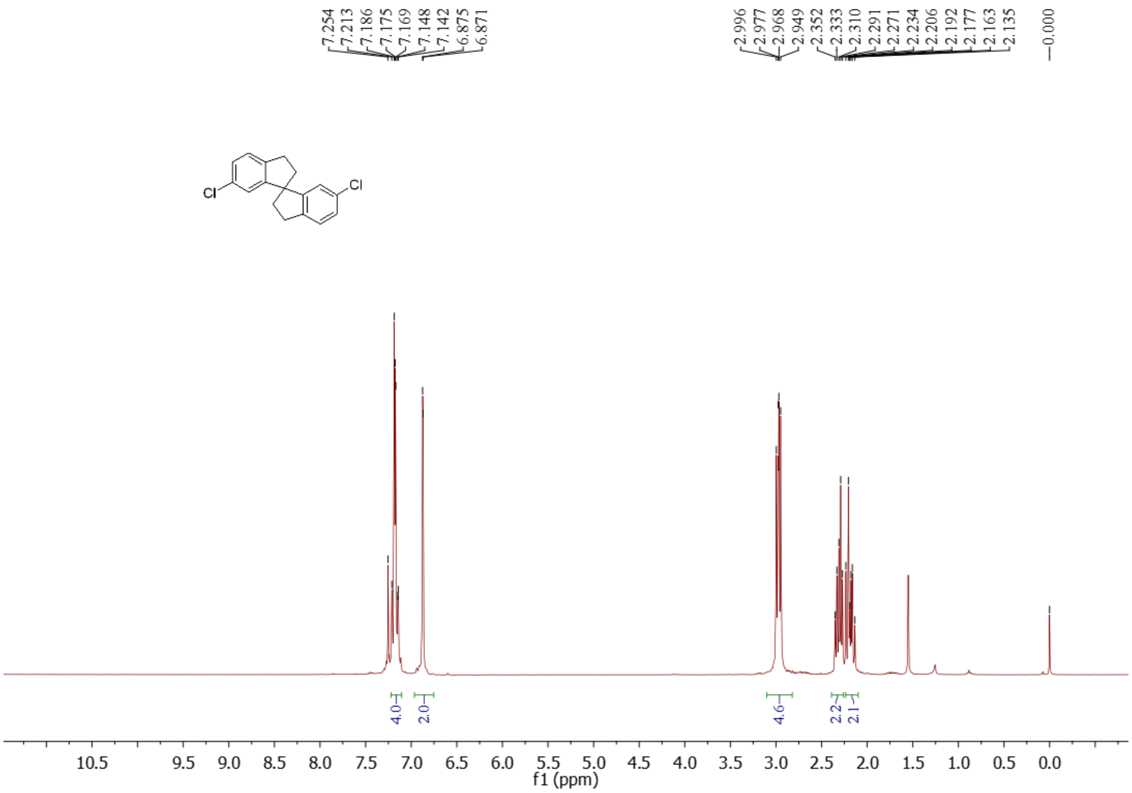

Figure S27. ^{13}C NMR spectrum of **2m**, related to **Figure 2**

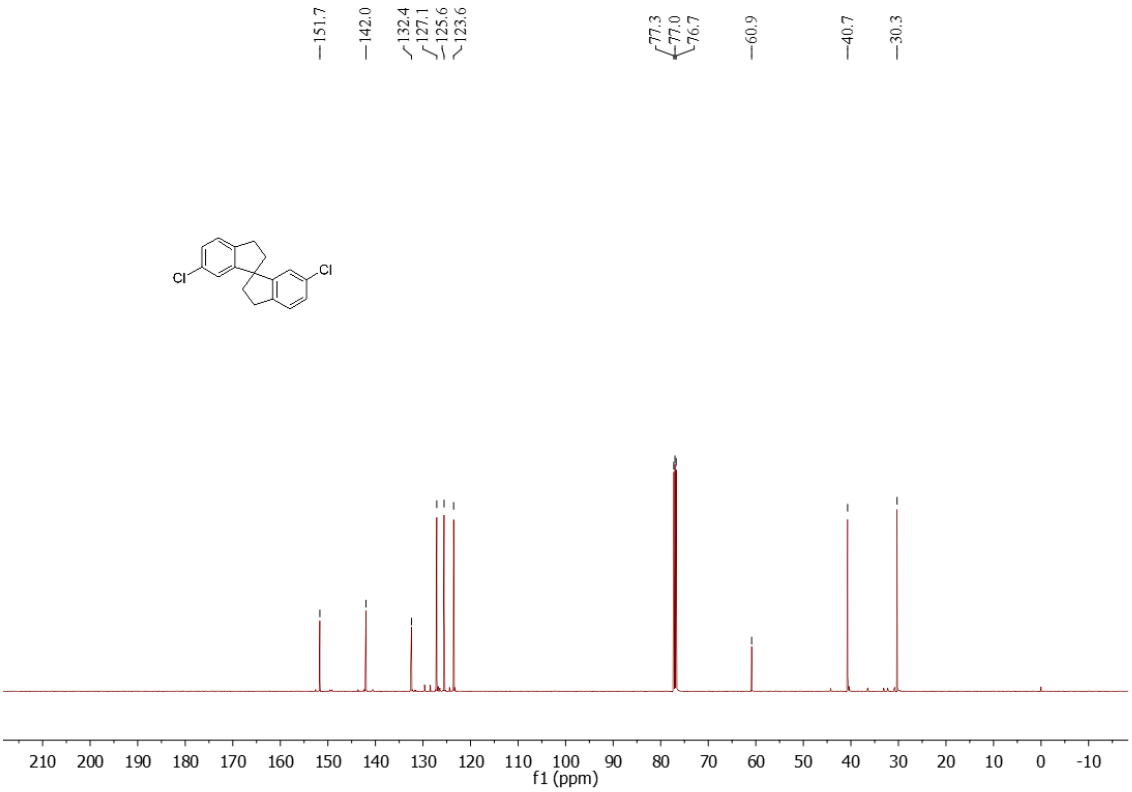

Figure S28. ^{1}H NMR spectrum of **2n**, related to **Figure 2**

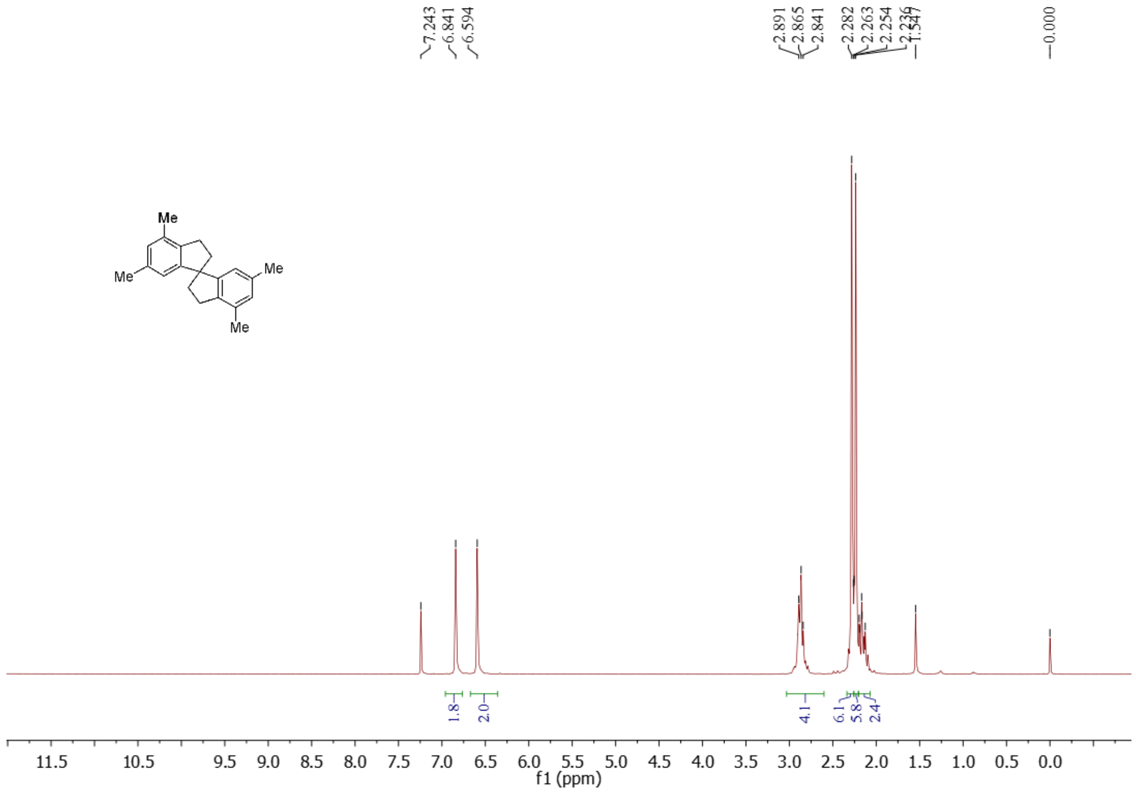

Figure S29. ^{13}C NMR spectrum of **2n**, related to **Figure 2**

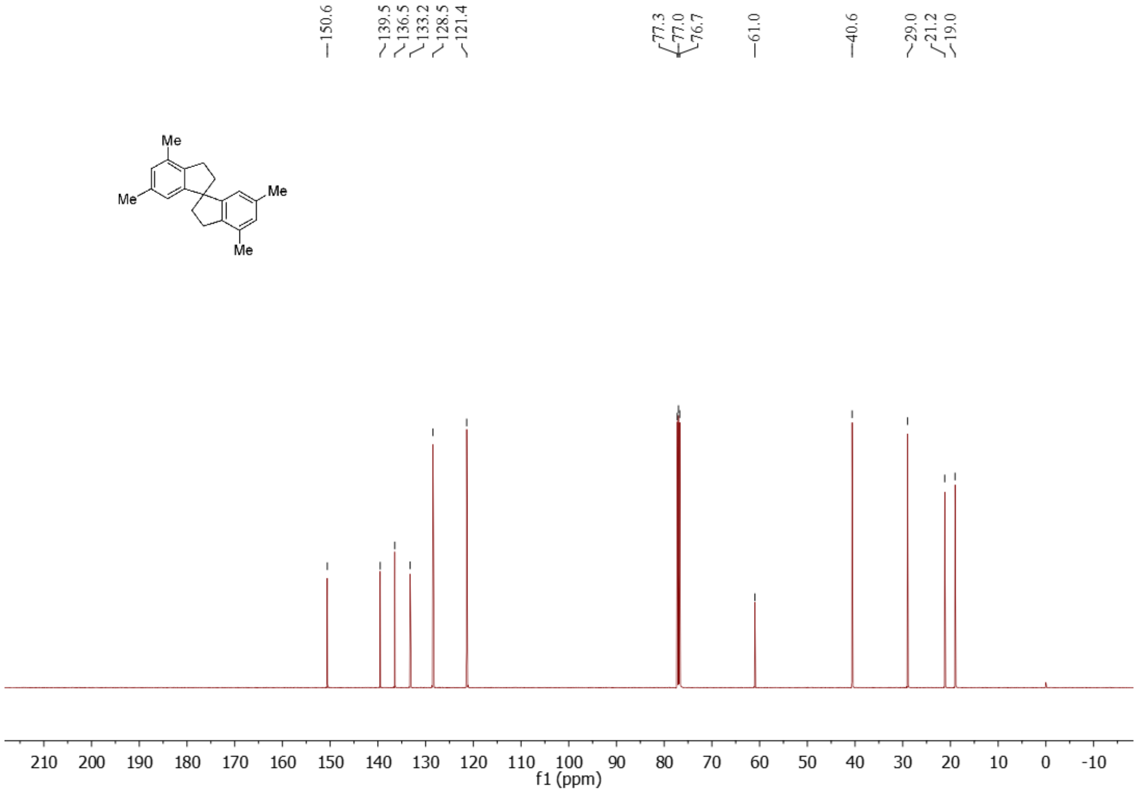

Figure S30. ^{1}H NMR spectrum of **2o**, related to **Figure 2**

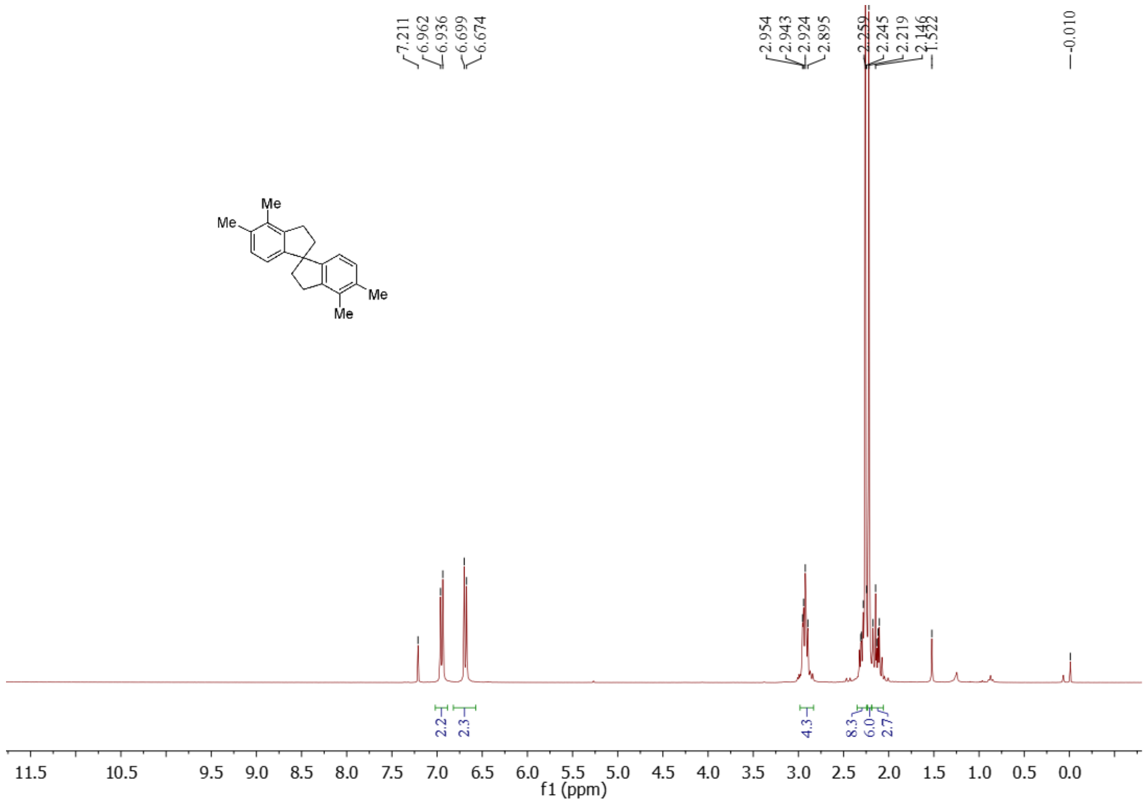

Figure S31. ^{13}C NMR spectrum of **2o**, related to **Figure 2**

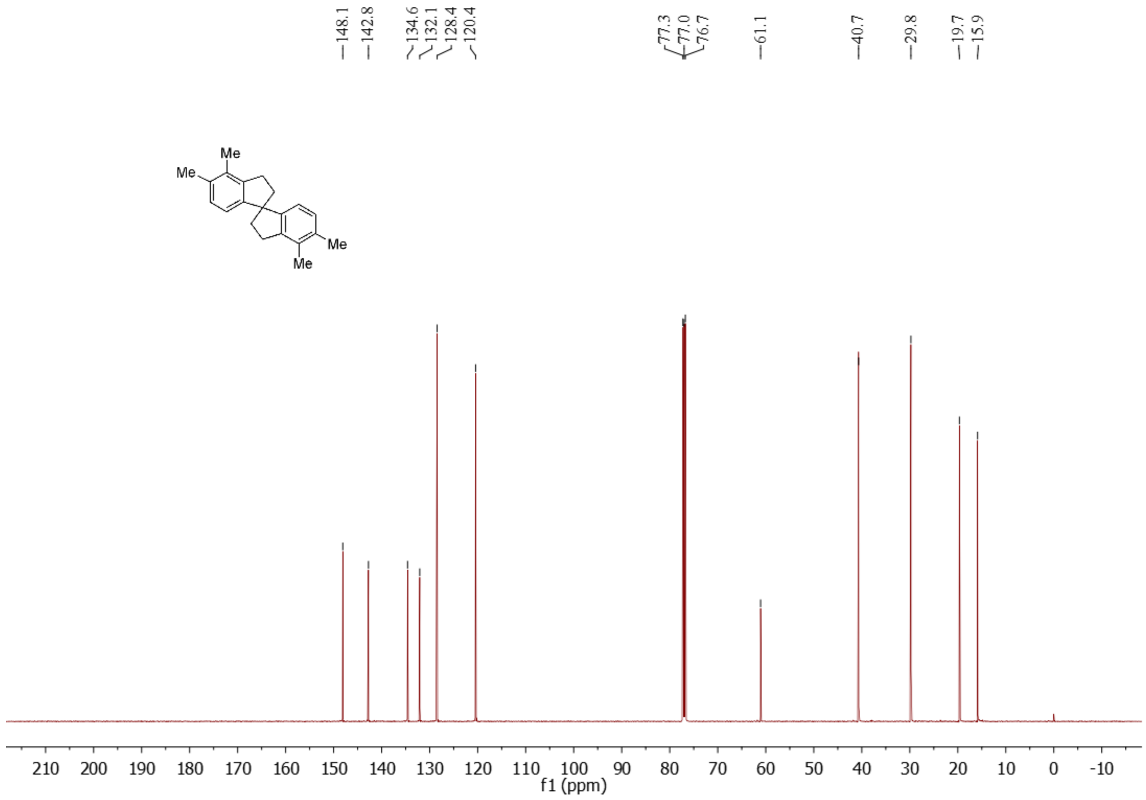

Figure S32. ^{1}H NMR spectrum of **2p**, related to **Figure 2**

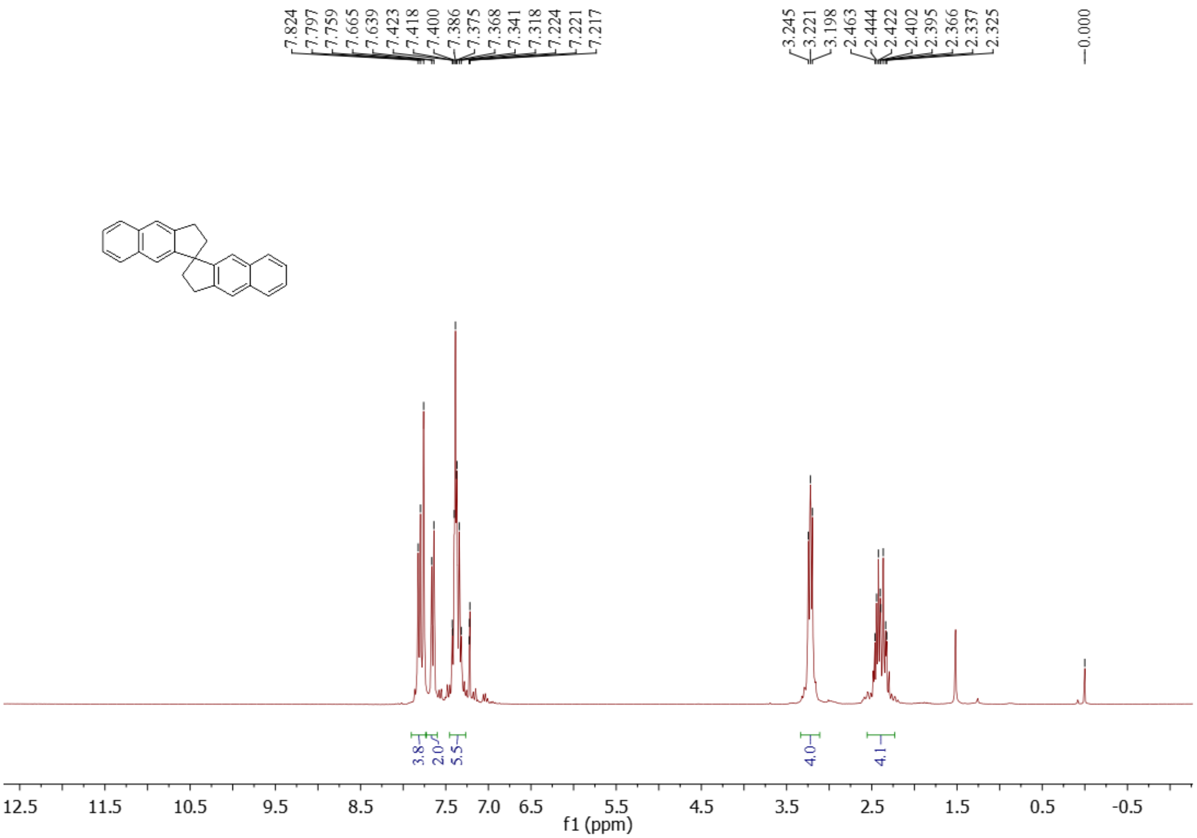

Figure S33. ^{13}C NMR spectrum of **2p**, related to **Figure 2**

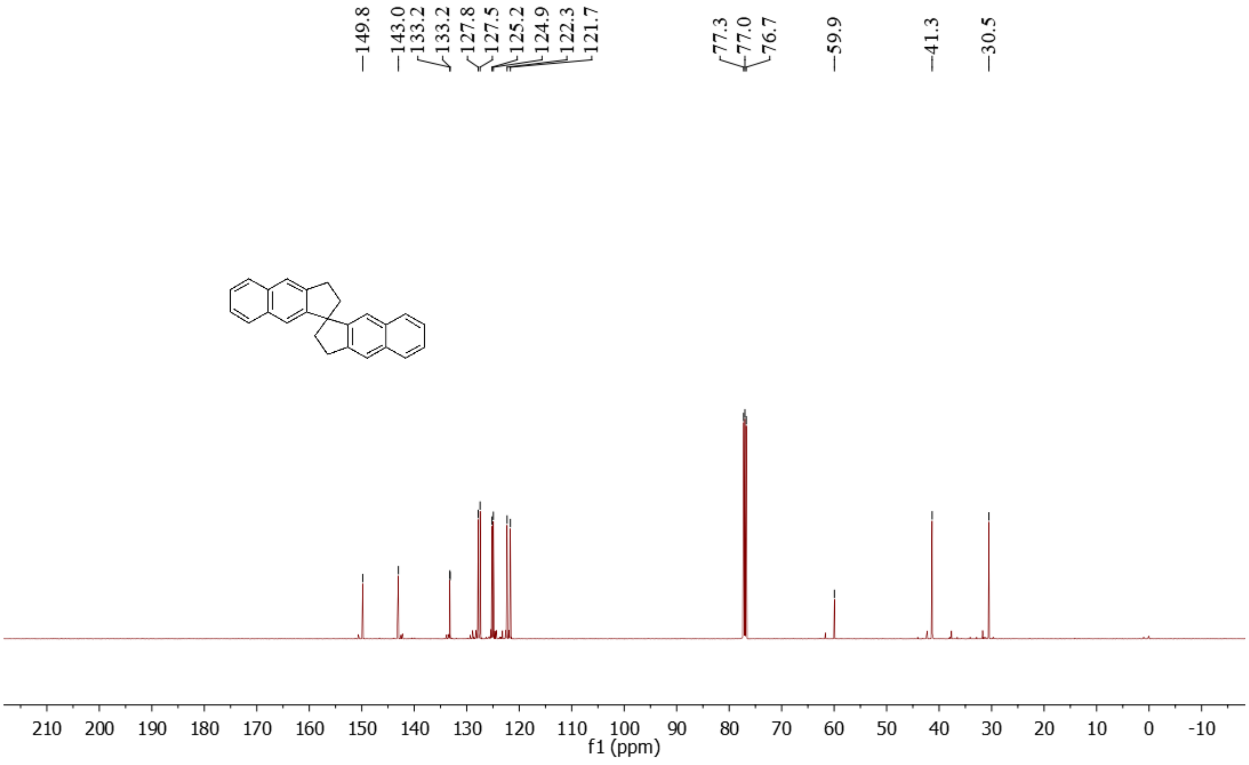

Figure S34. ^{1}H NMR spectrum of **2q**, related to **Figure 2**

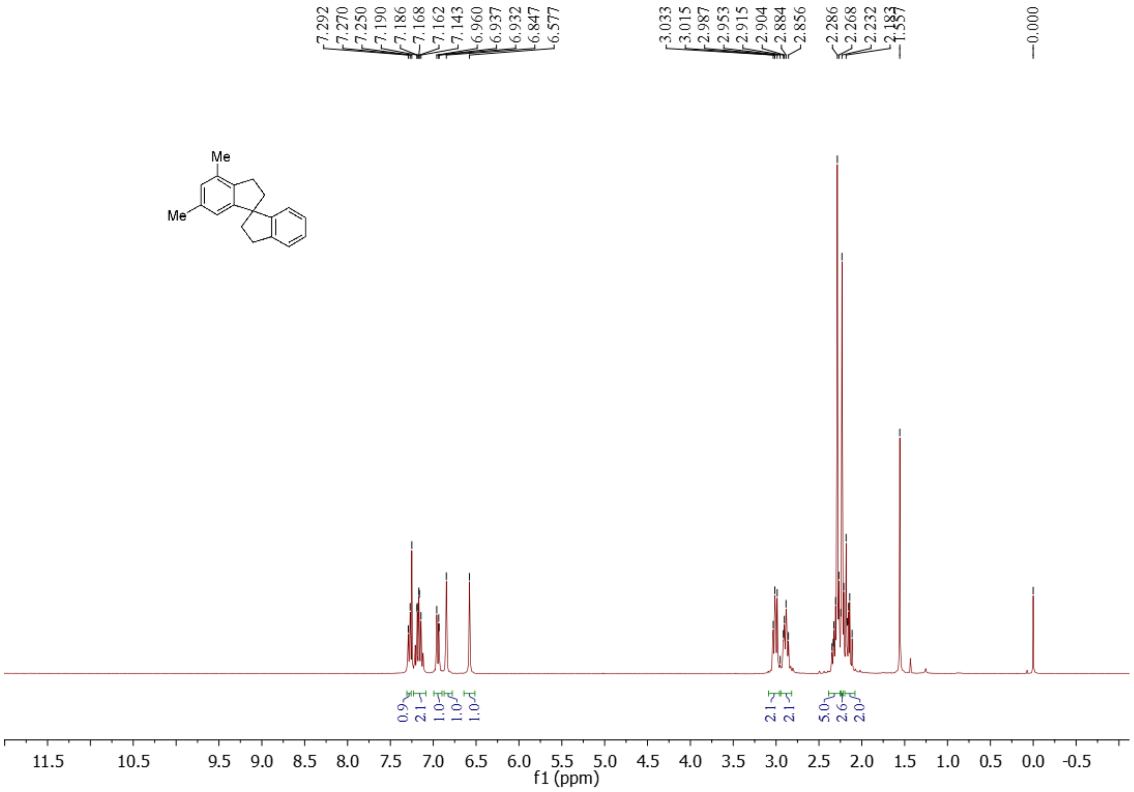

Figure S35. ^{13}C NMR spectrum of **2q**, related to **Figure 2**

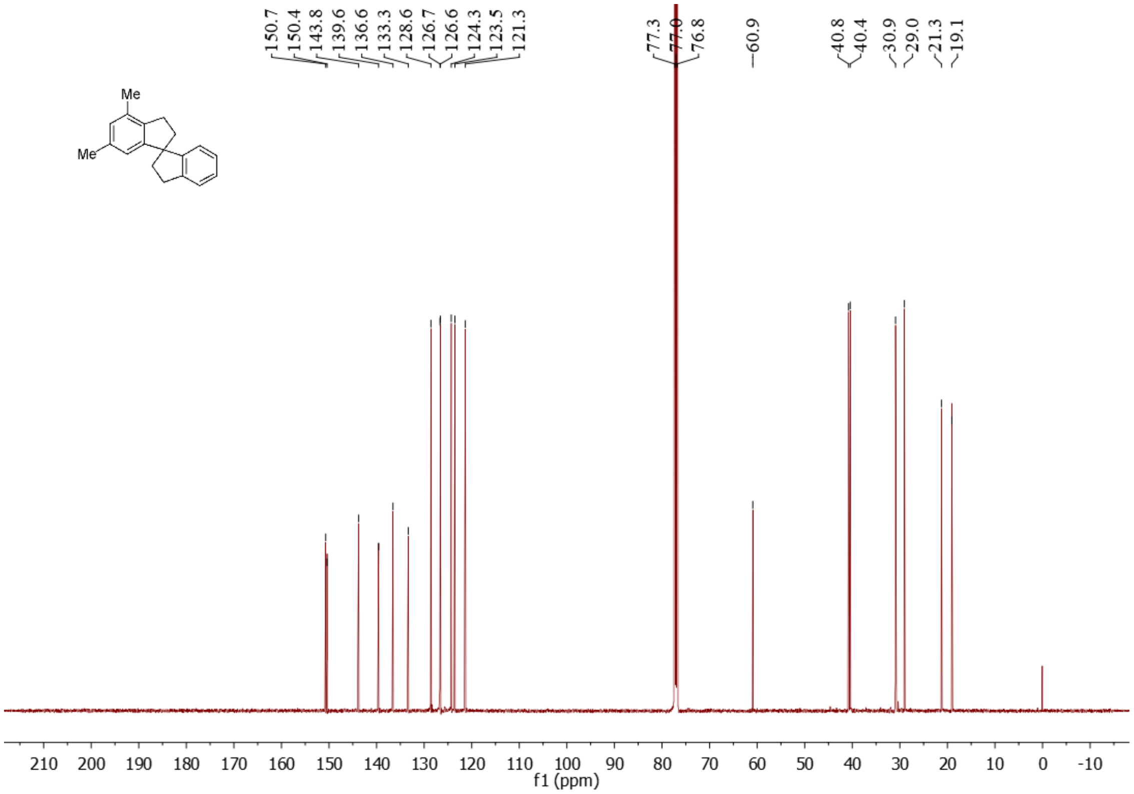

Figure S36. ^{1}H NMR spectrum of **2r**, related to **Figure 2**

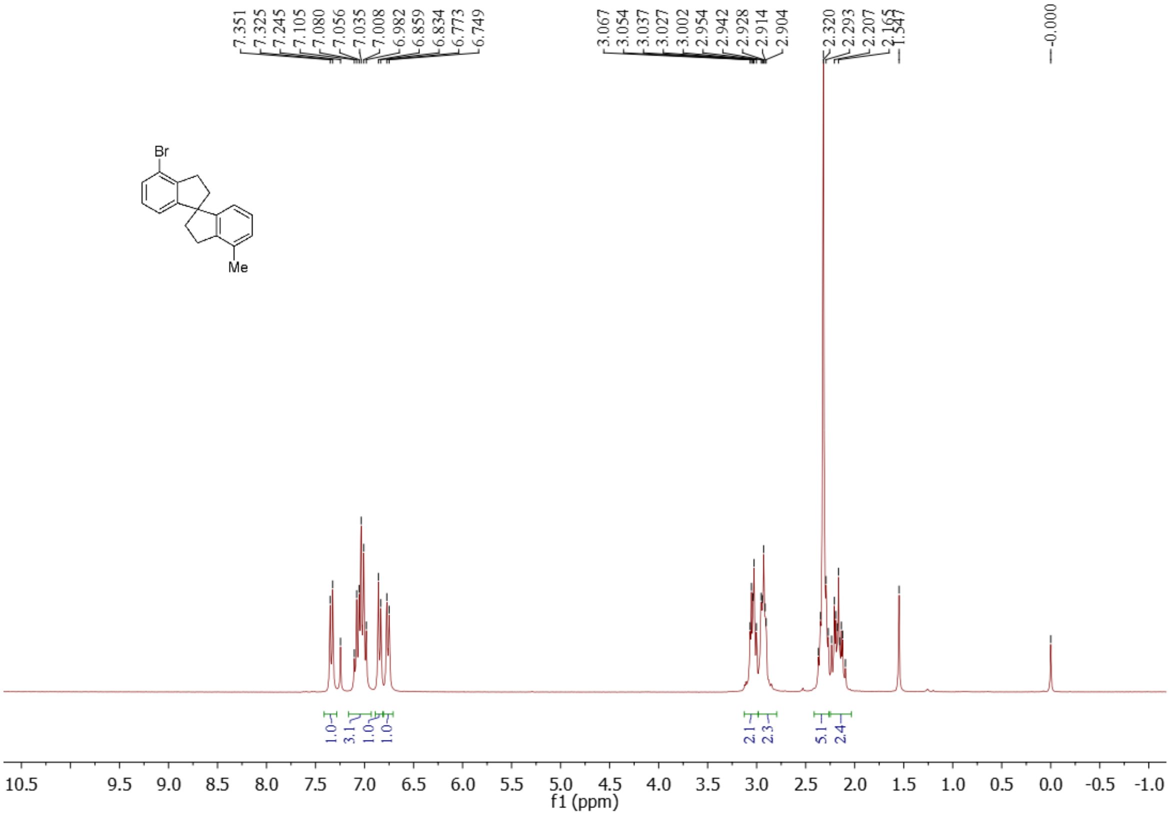

Figure S37. ^{13}C NMR spectrum of **2r**, related to **Figure 2**

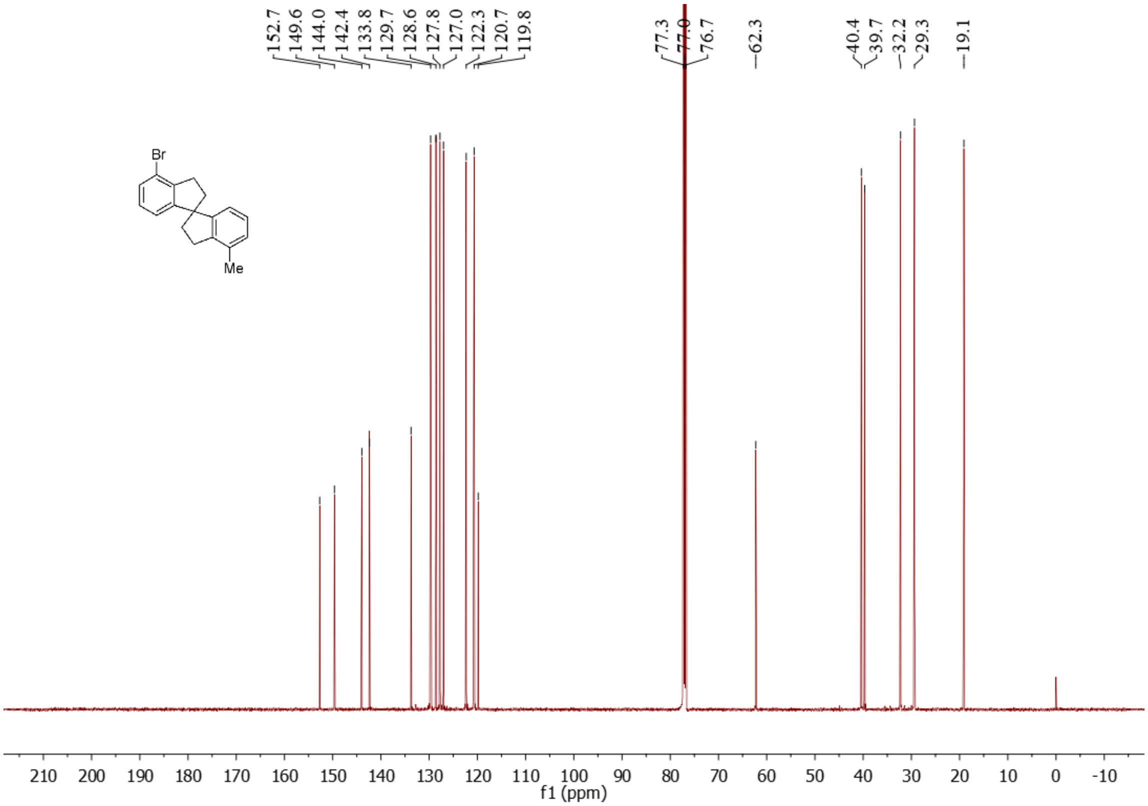

Figure S38. ^{1}H NMR spectrum of **2s**, related to **Figure 2**

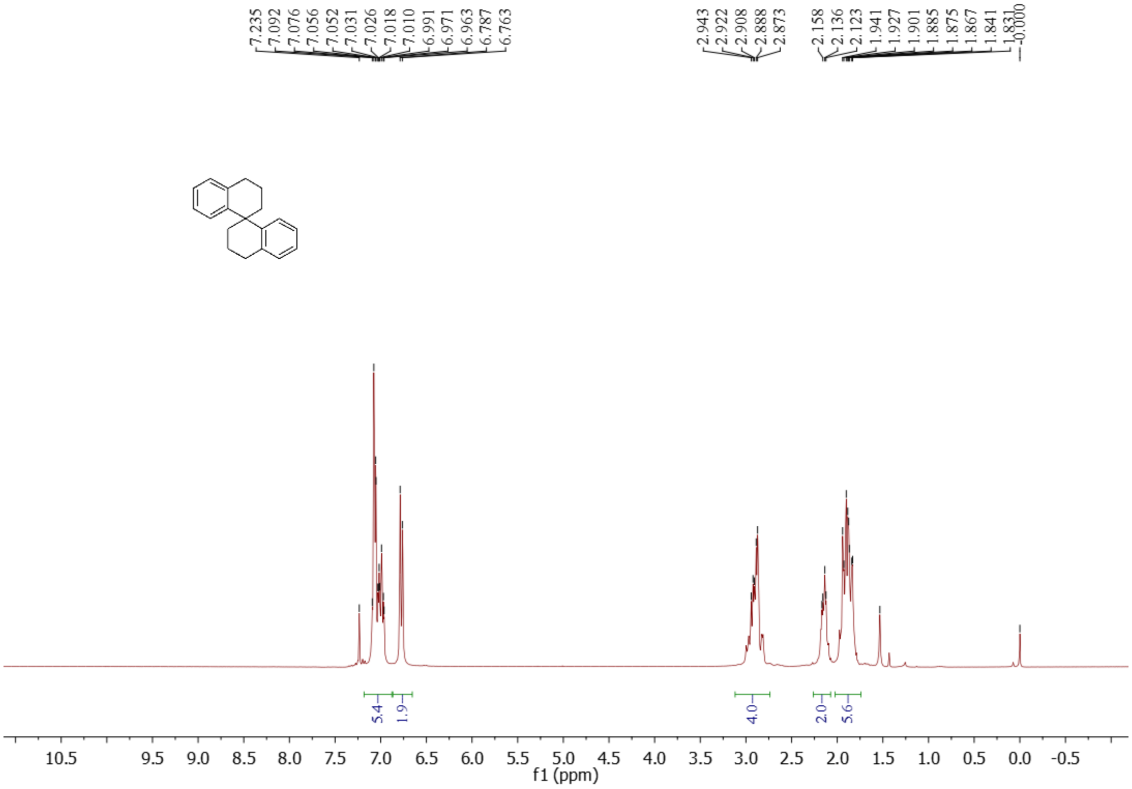

Figure S39. ^{13}C NMR spectrum of **2s**, related to **Figure 2**

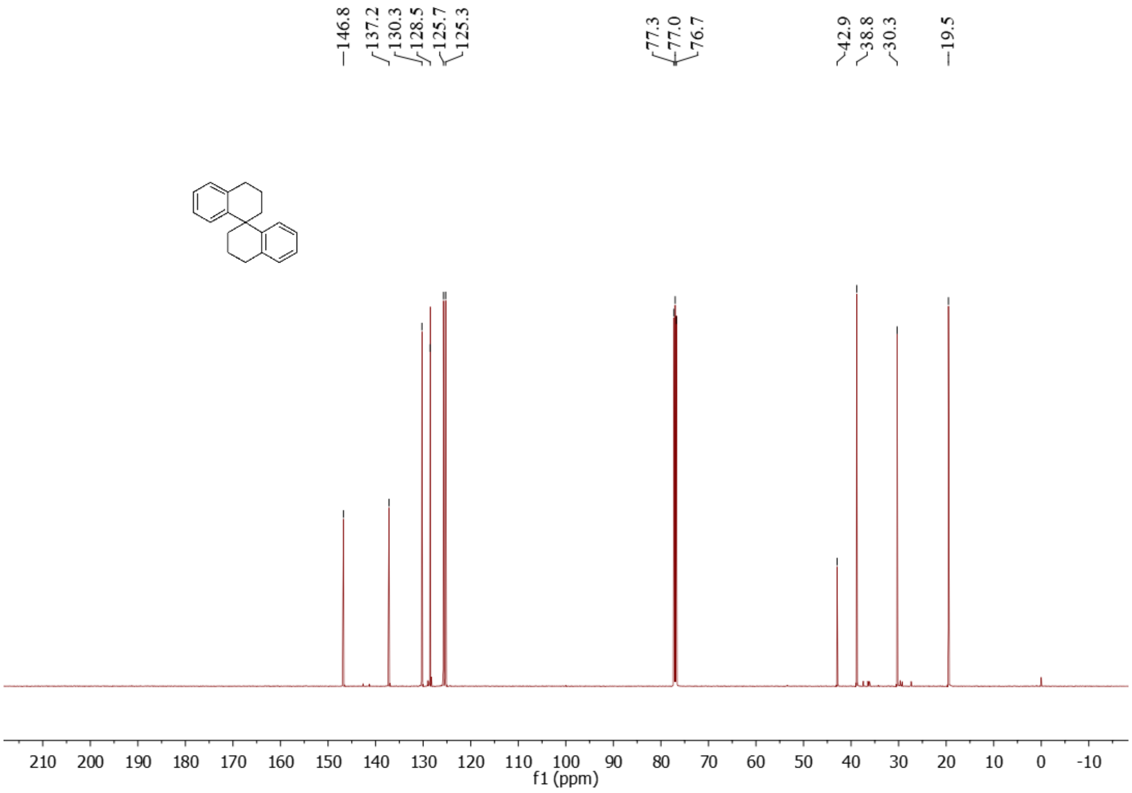

Figure S40. ^{1}H NMR spectrum of **2t**, related to **Figure 2**

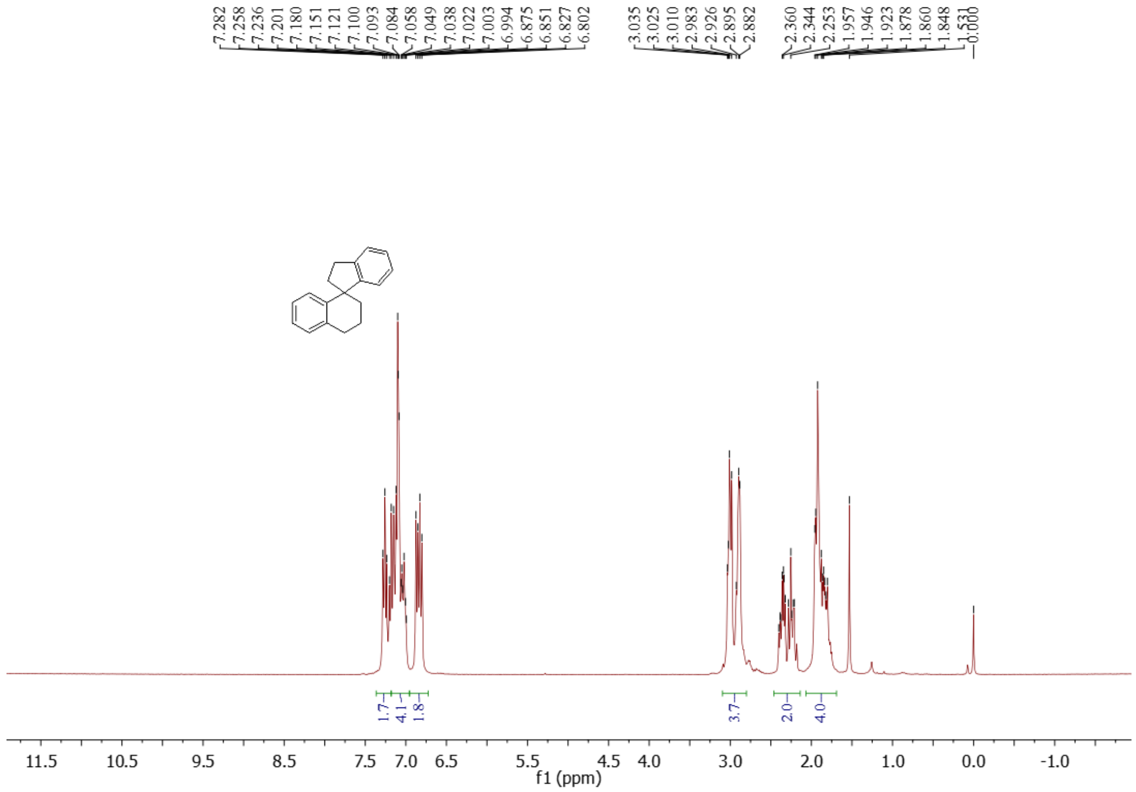

Figure S41. ^{13}C NMR spectrum of **2t**, related to **Figure 2**

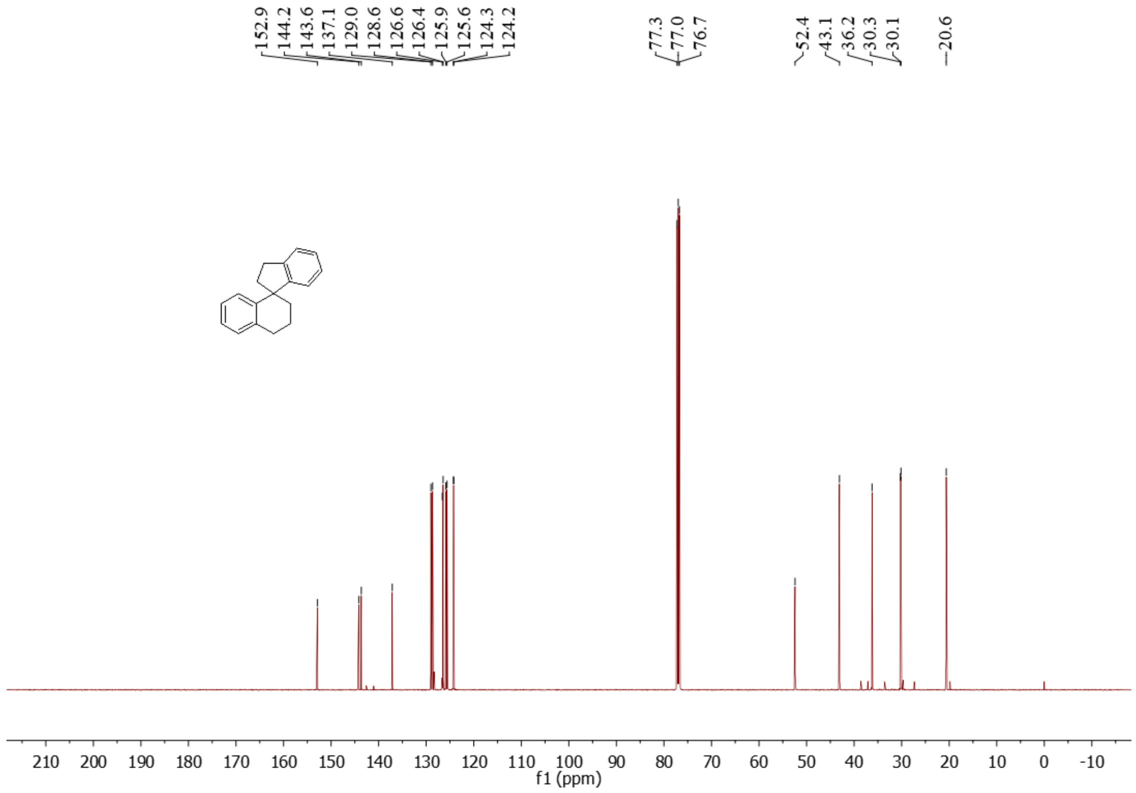

Figure S42. ^{1}H NMR spectrum of **3a**, related to **Figure 2**

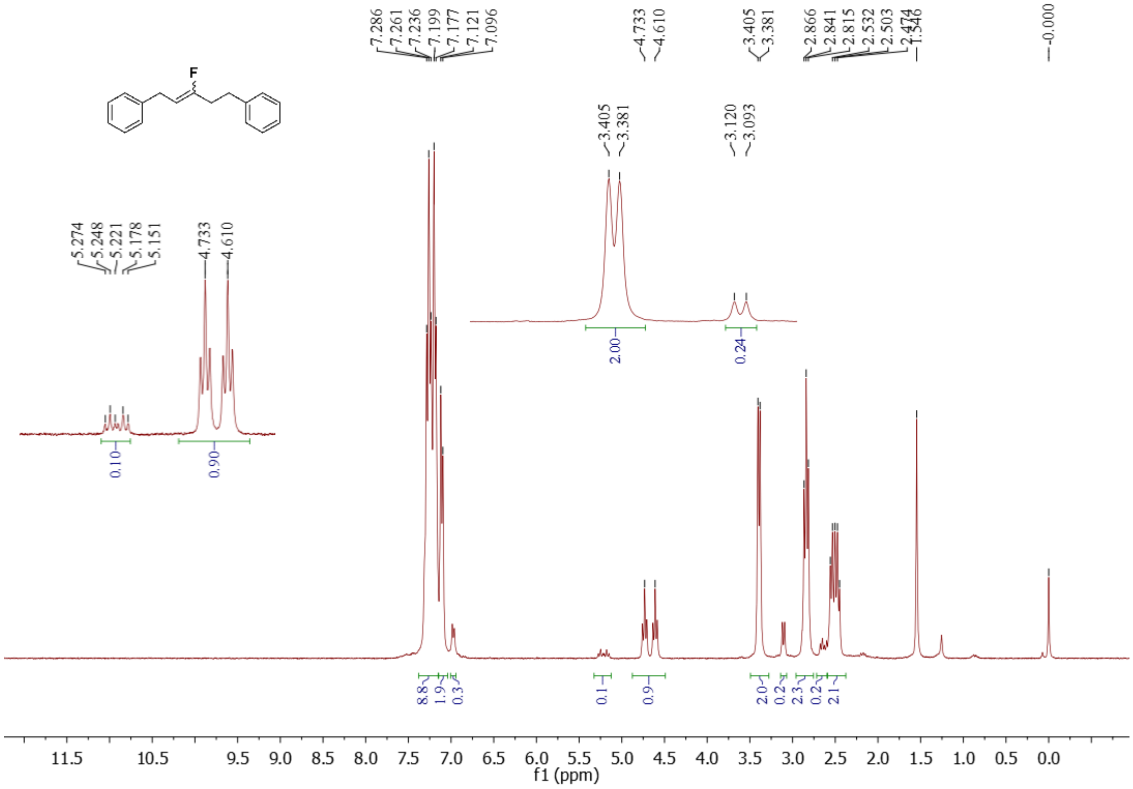

Figure S43. ^{13}C NMR spectrum of **3a**, related to **Figure 2**

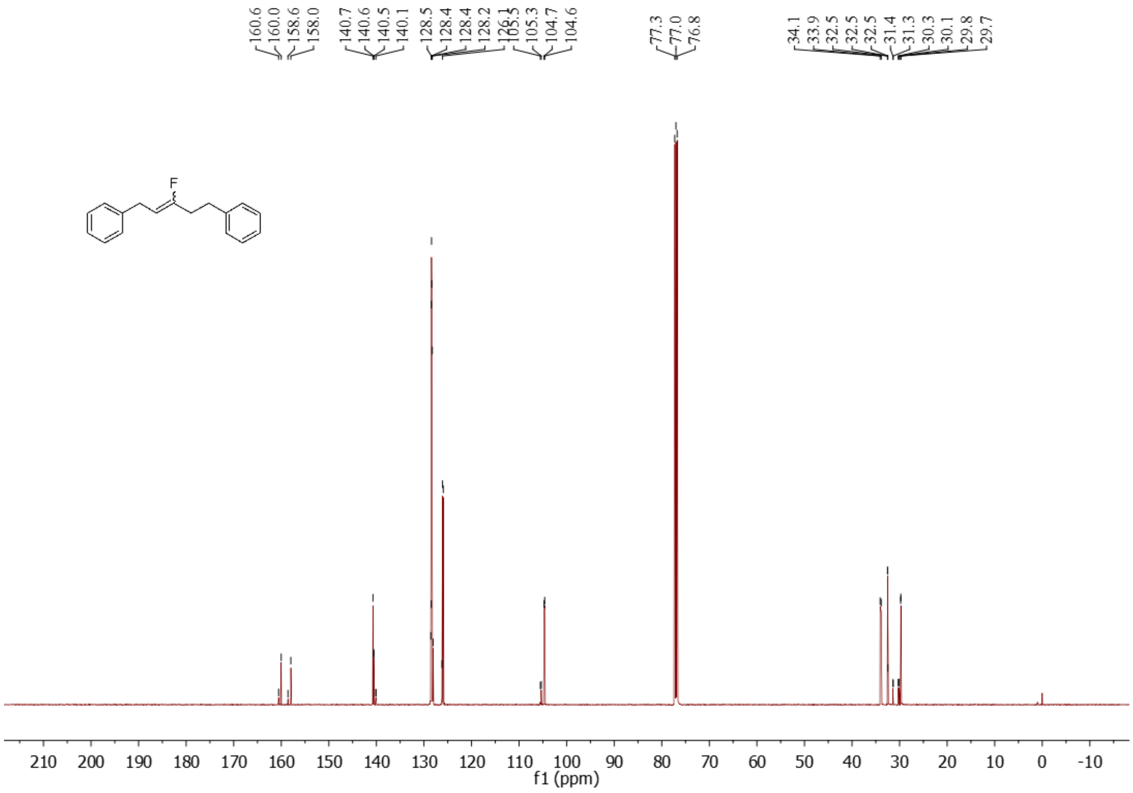

Figure S44. ^{19}F NMR spectrum of **3a**, related to **Figure 2**

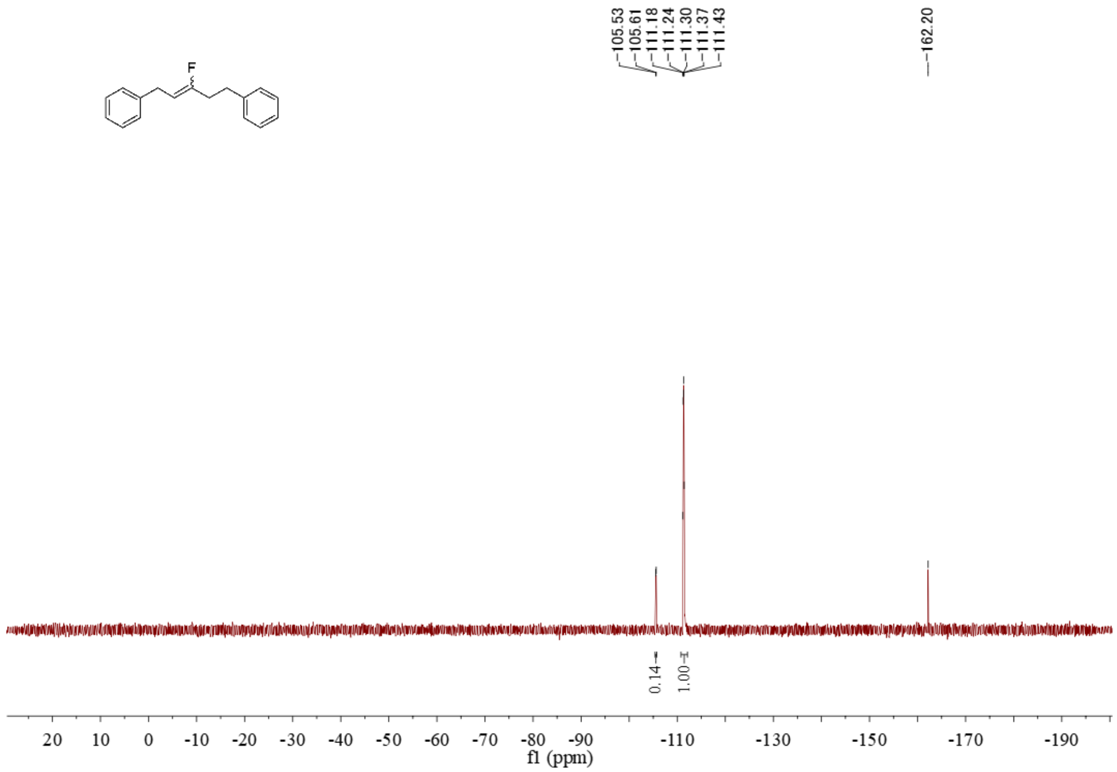

Figure S45. ^{1}H NMR spectrum of **3e**, related to **Figure 2**

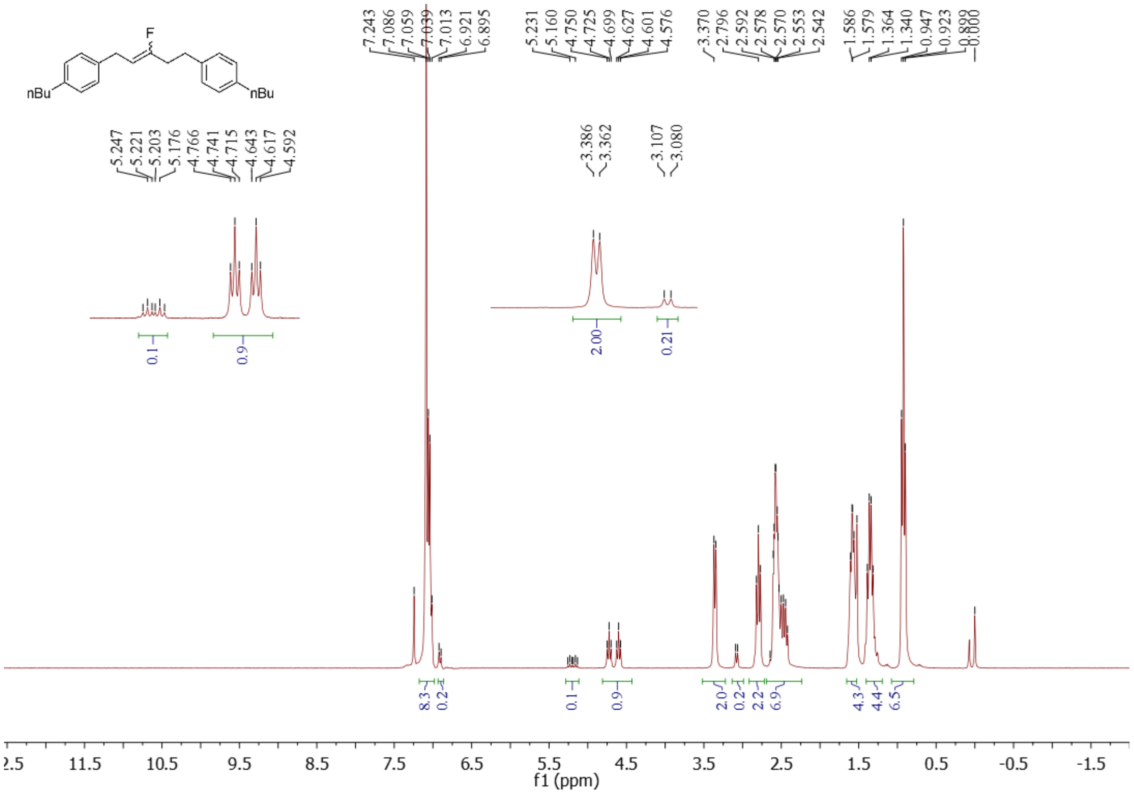

Figure S46. ^{13}C NMR spectrum of **3e**, related to **Figure 2**

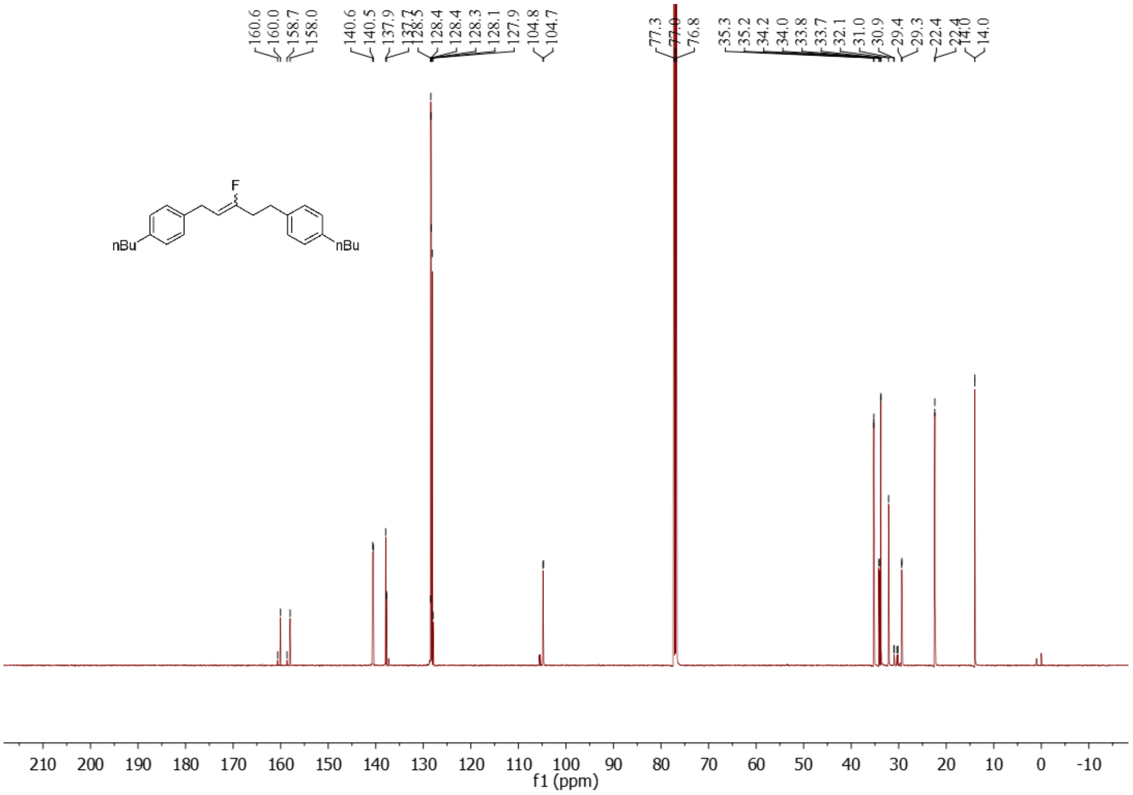

Figure S47. ^{19}F NMR spectrum of **3e**, related to **Figure 2**

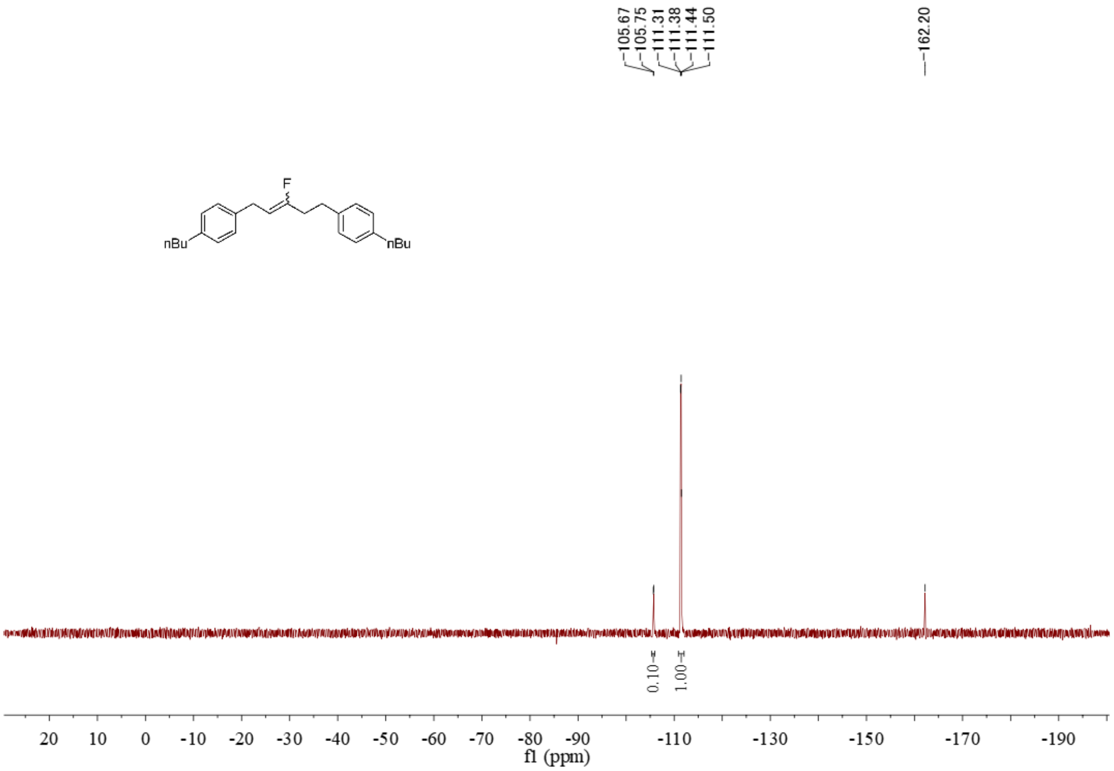

Figure S48. ^{1}H NMR spectrum of **3f**, related to **Figure 2**

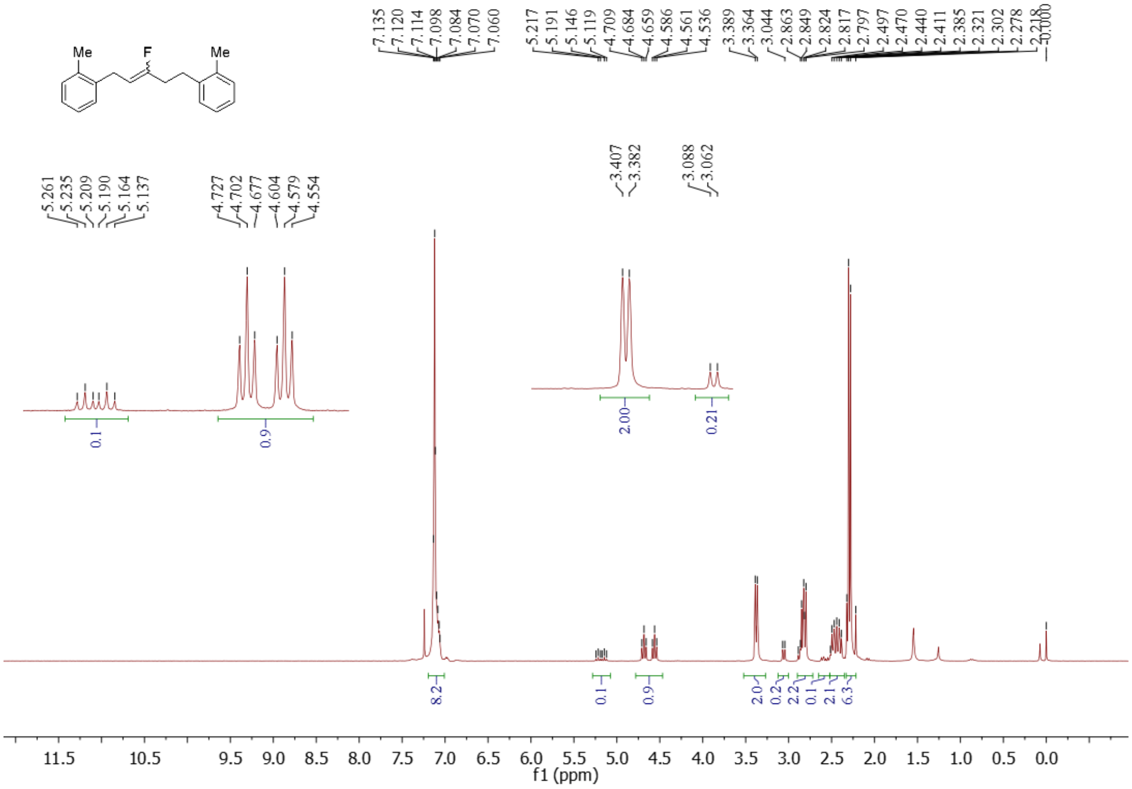

Figure S49. ^{13}C NMR spectrum of **3f**, related to **Figure 2**

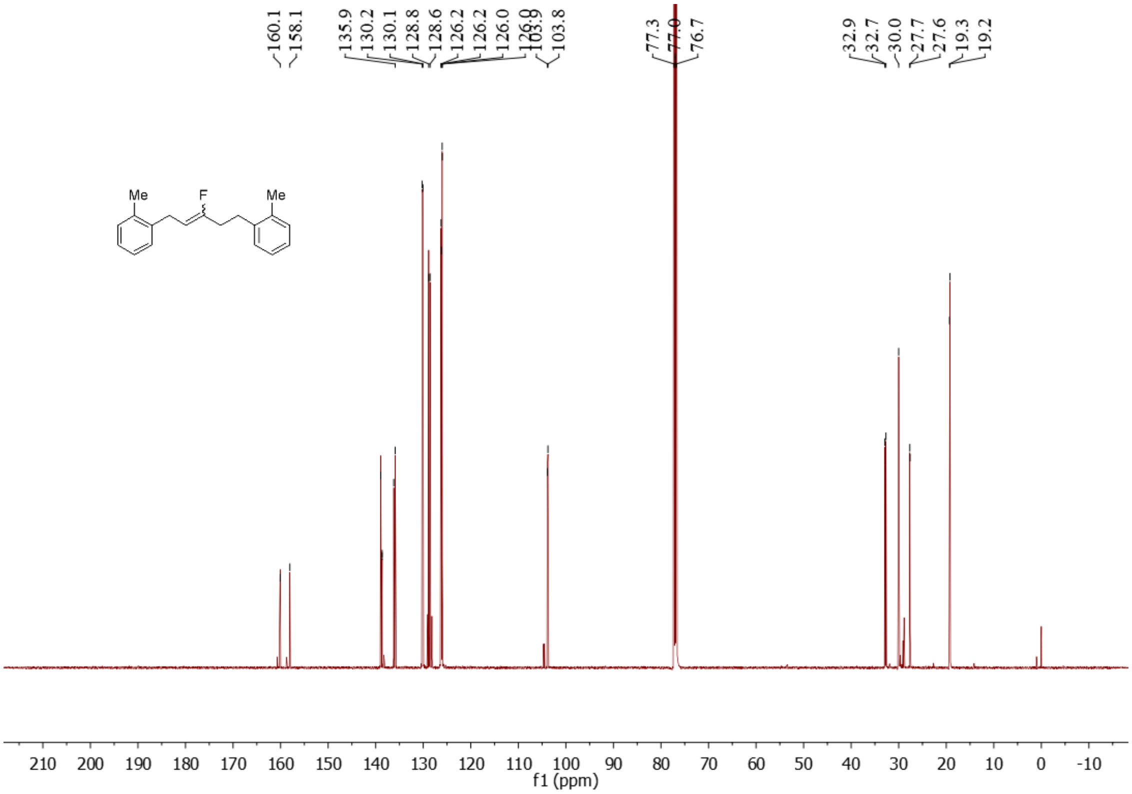

Figure S50. ^{19}F NMR spectrum of **3f**, related to **Figure 2**

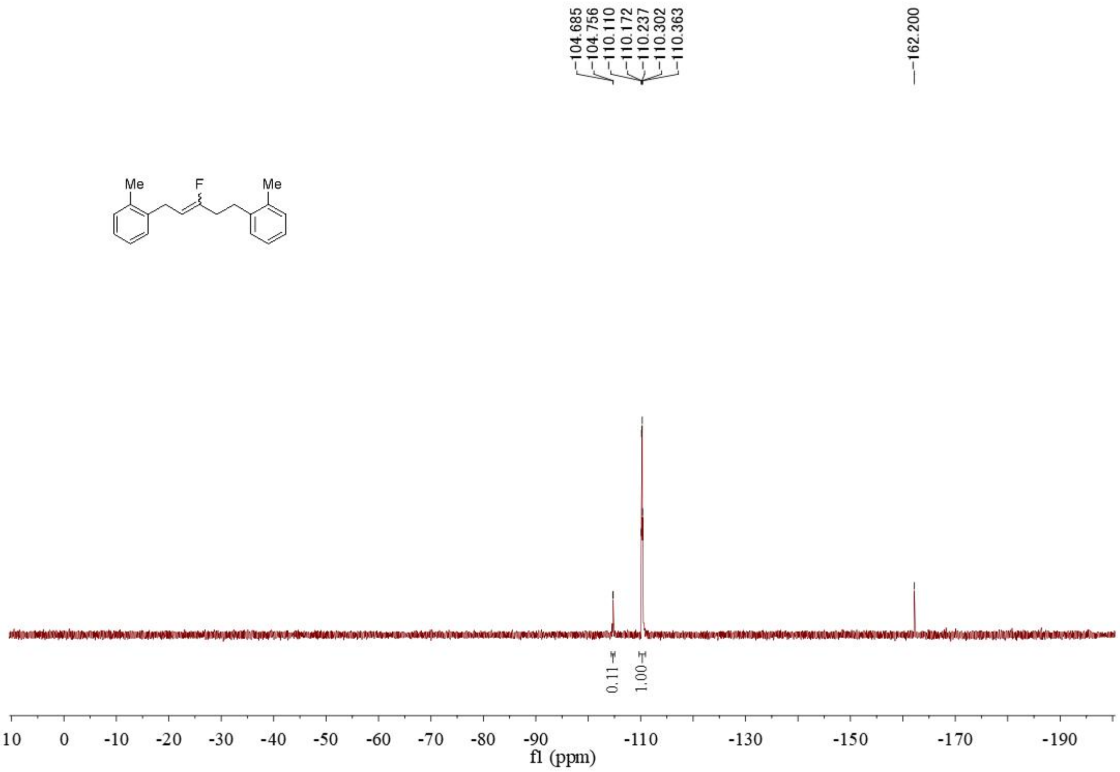

Figure S51. ^{1}H NMR spectrum of **3g**, related to **Figure 2**

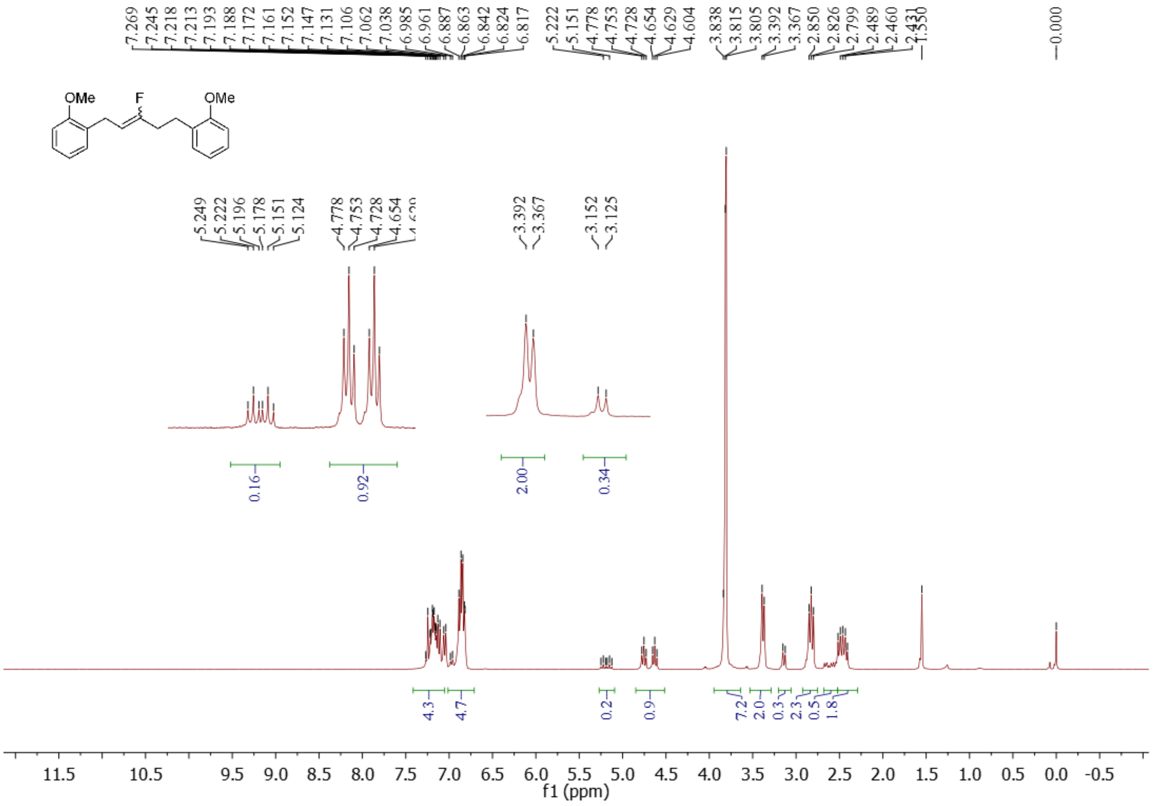

Figure S52. ^{13}C NMR spectrum of **3g**, related to **Figure 2**

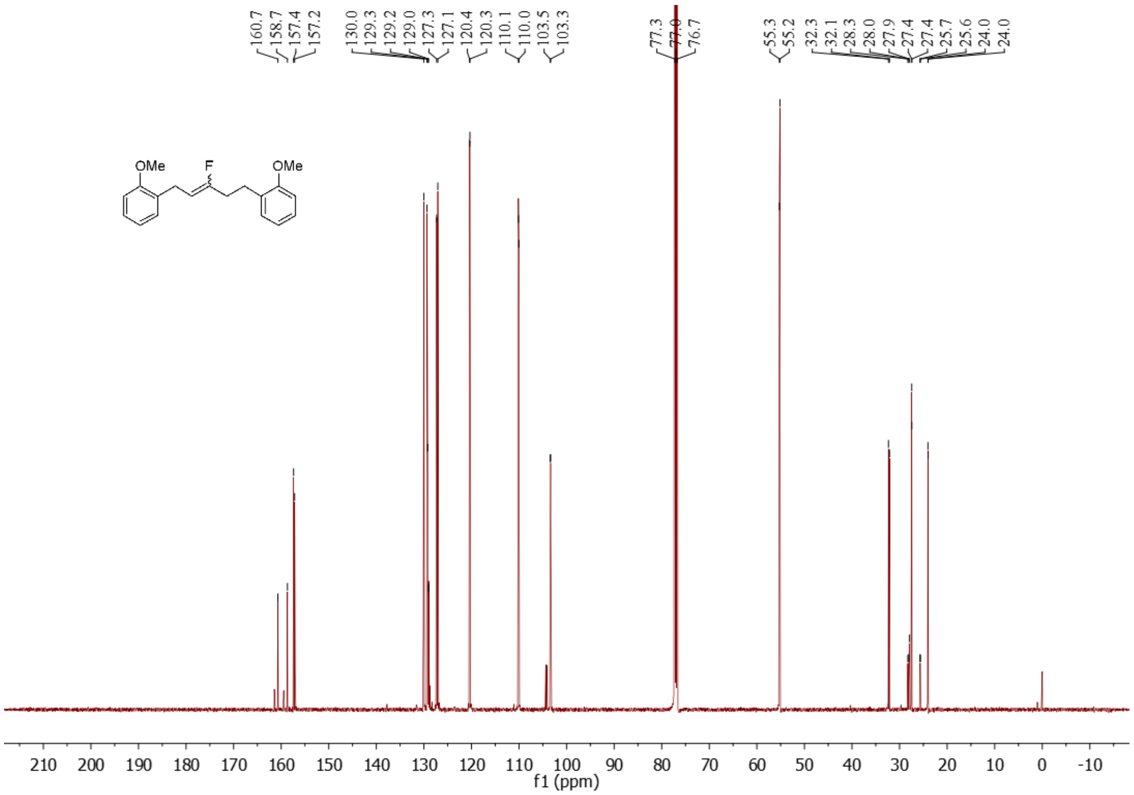

Figure S53. ^{19}F NMR spectrum of **3g**, related to **Figure 2**

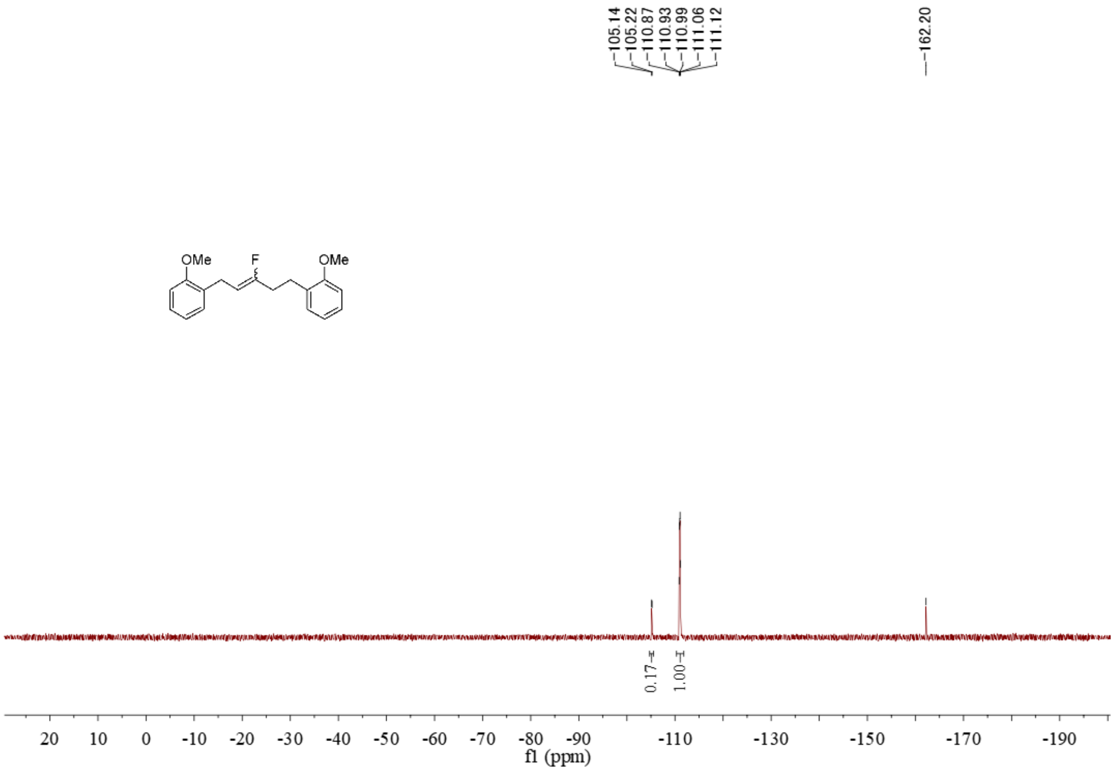

Figure S54. ^{1}H NMR spectrum of **3n**, related to **Figure 2**

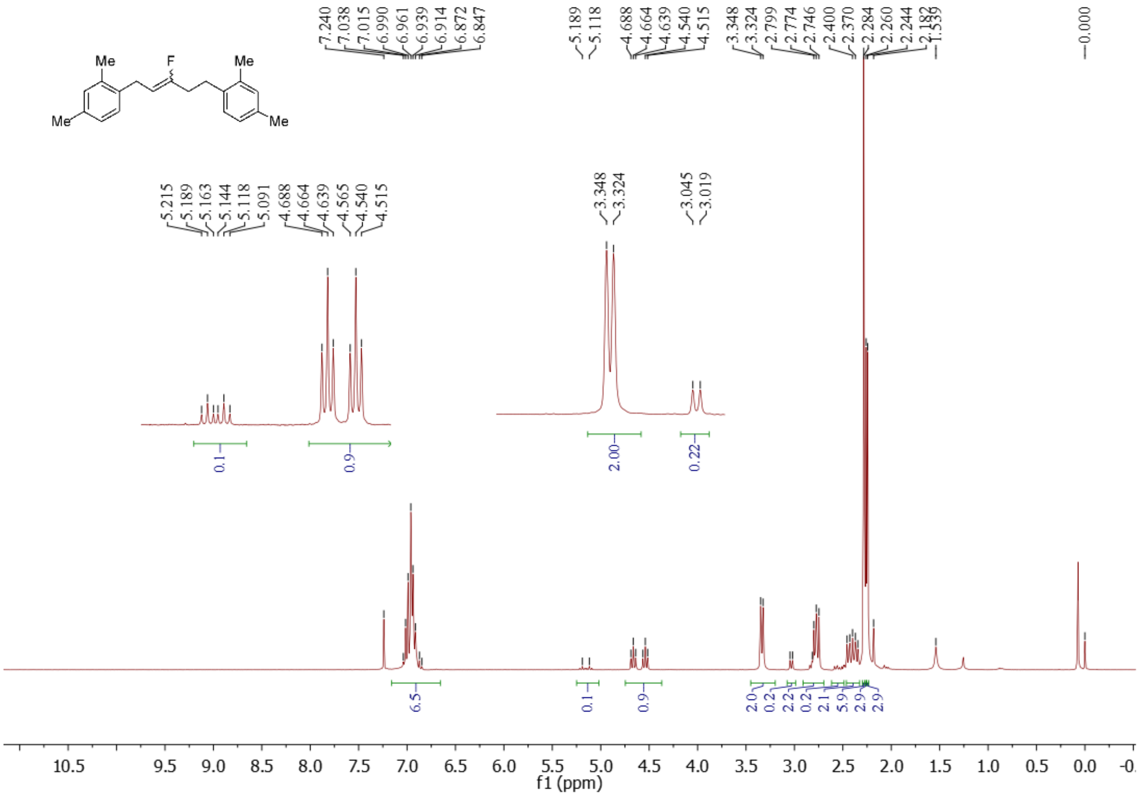

Figure S55. ^{13}C NMR spectrum of **3n**, related to **Figure 2**

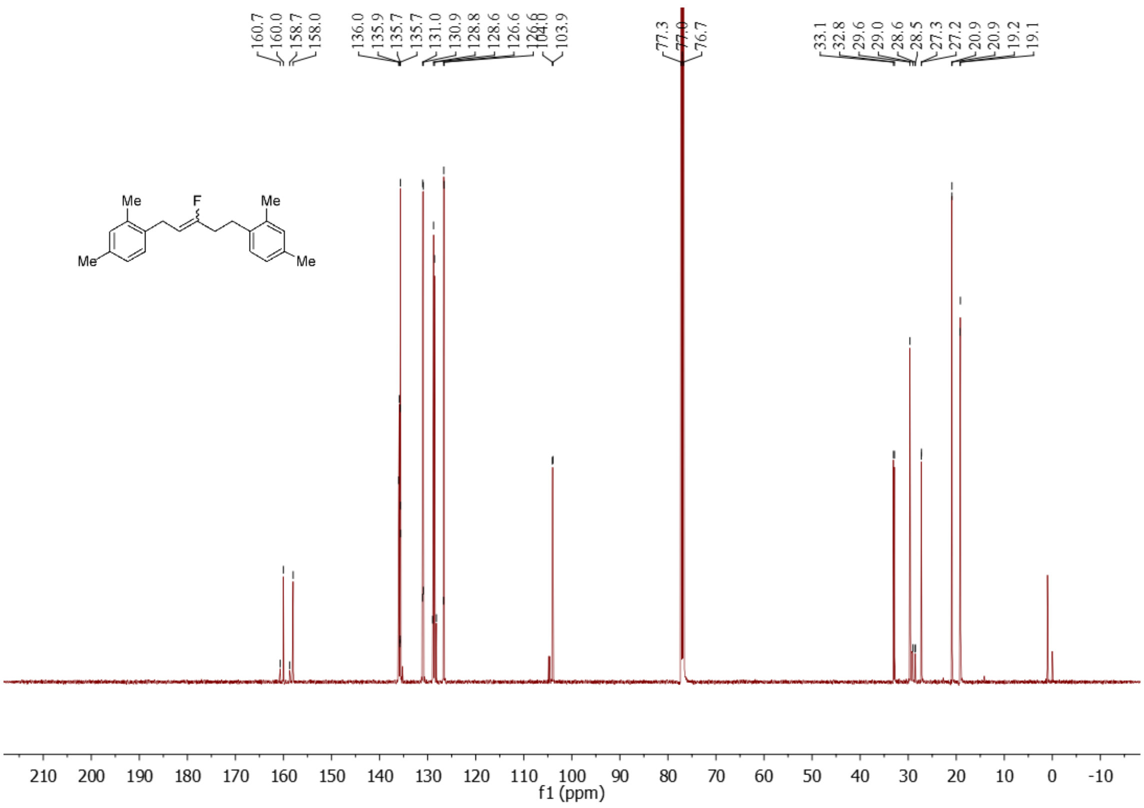

Figure S56. ^{19}F NMR spectrum of **3n**, related to **Figure 2**

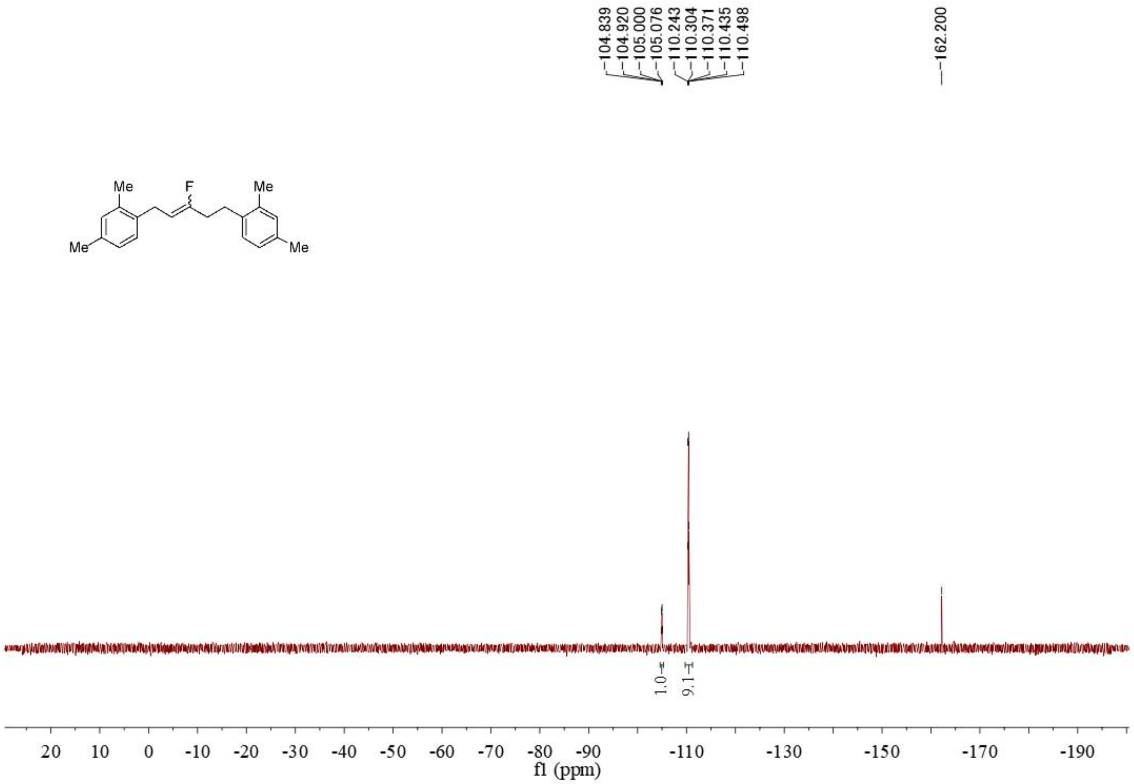

Figure S57. ^{1}H NMR spectrum of **3o**, related to **Figure 2**

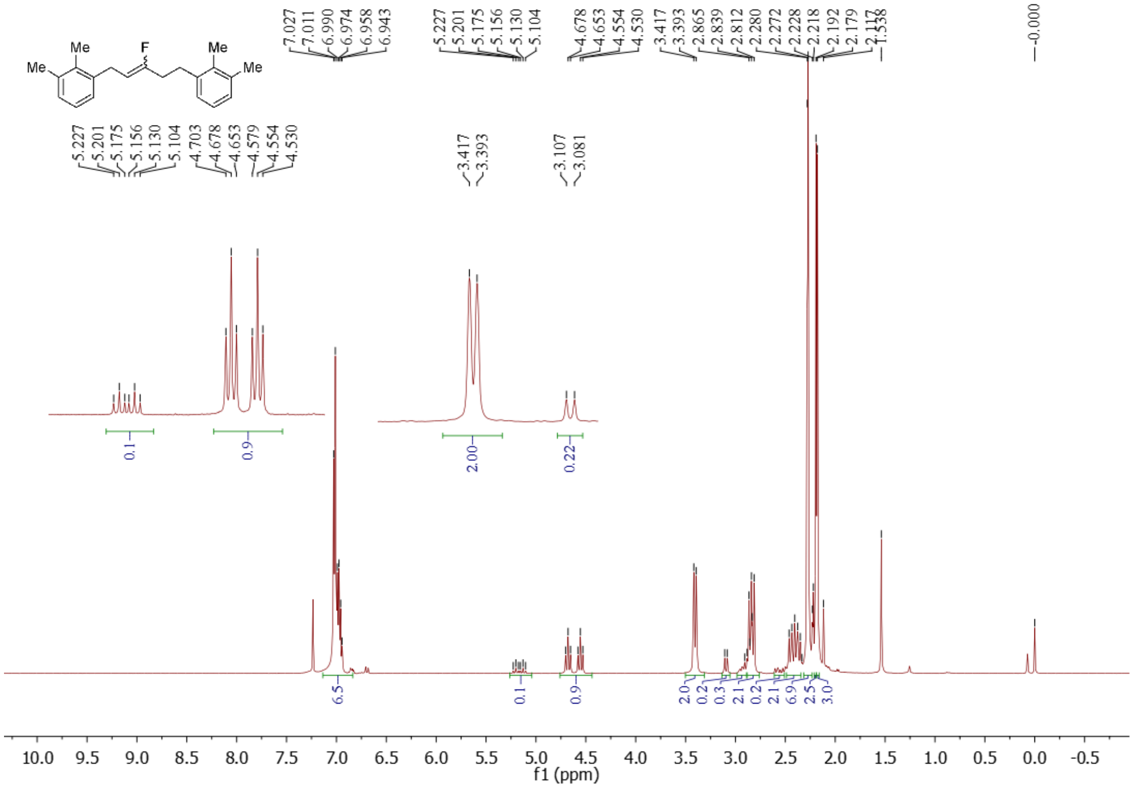

Figure S58. ^{13}C NMR spectrum of **3o**, related to **Figure 2**

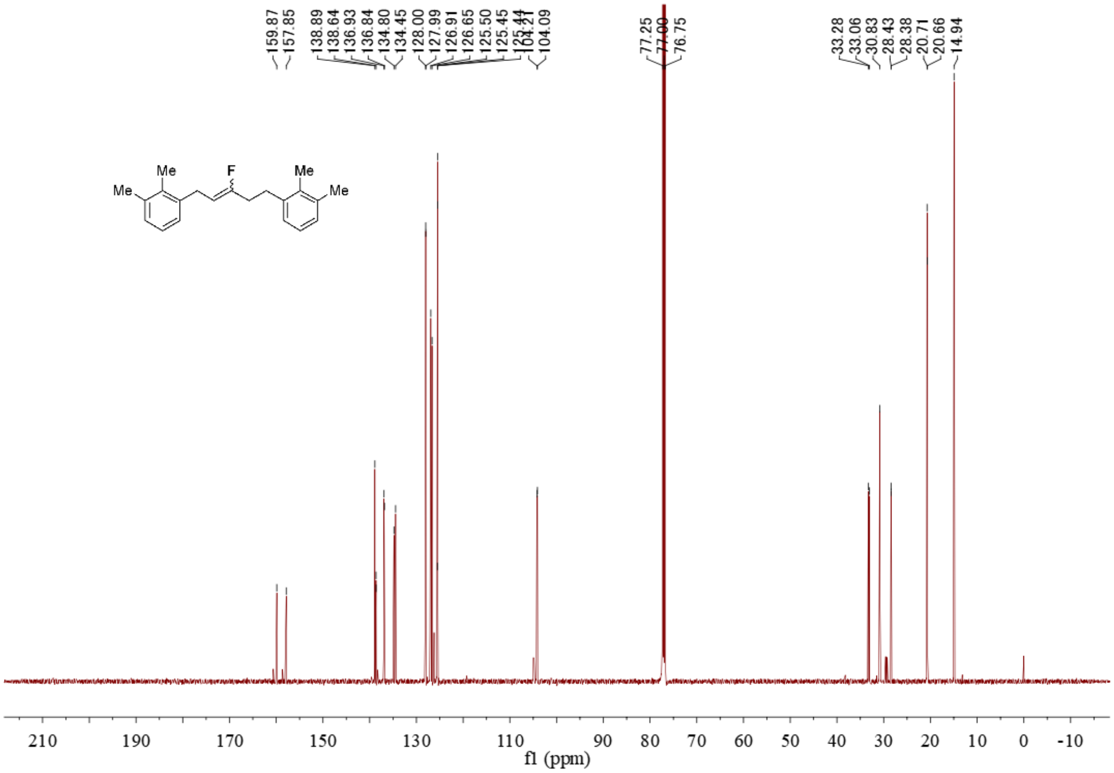

Figure S59. ^{19}F NMR spectrum of **3o**, related to **Figure 2**

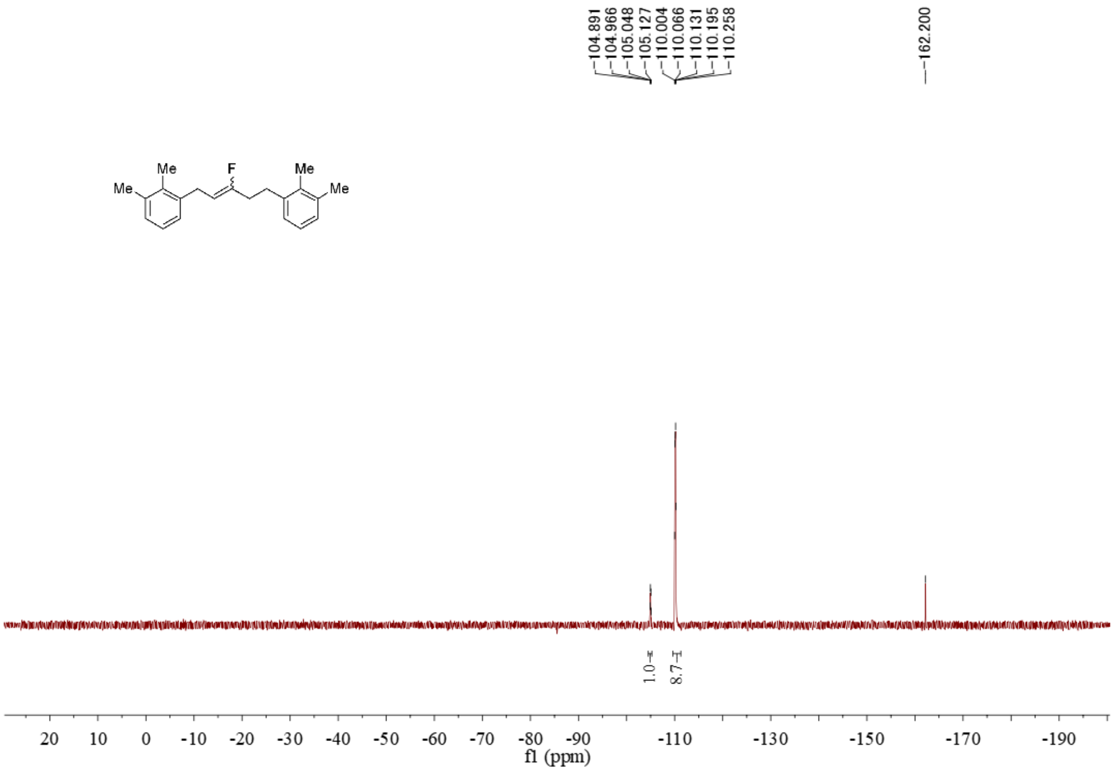

Figure S60. ^{1}H NMR spectrum of **3j**, related to **Figure 2**

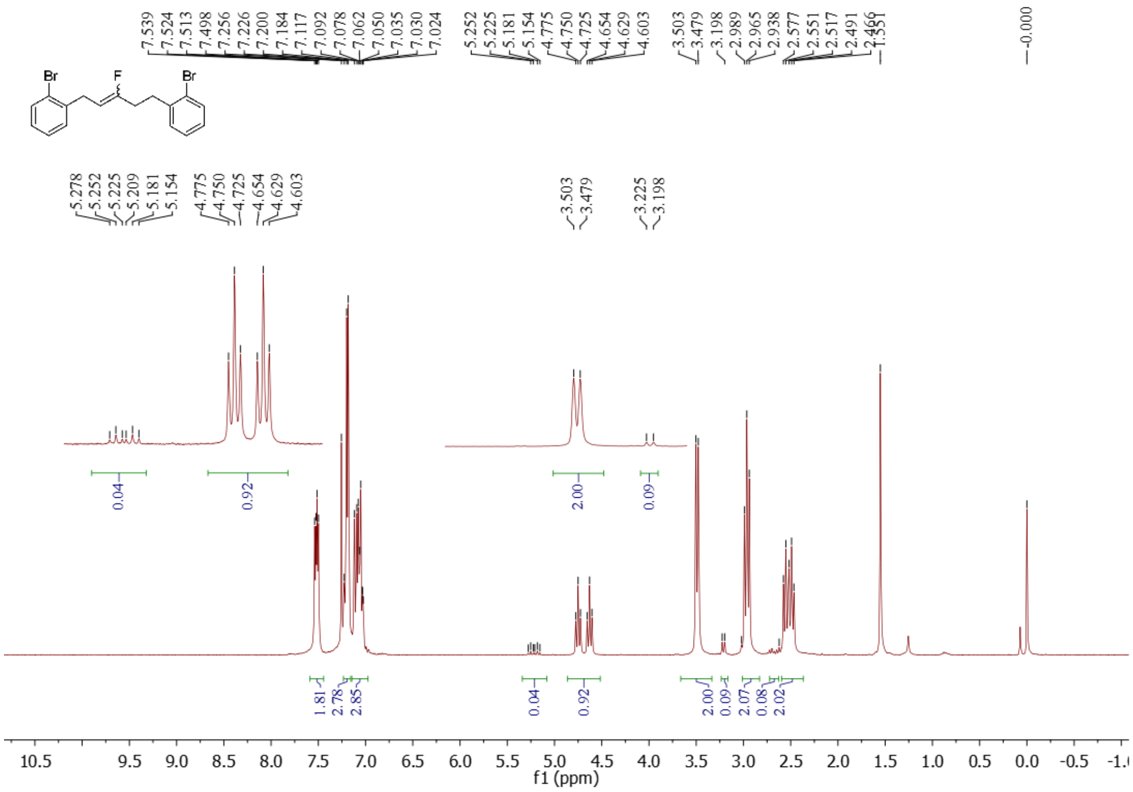

Figure S61. ^{13}C NMR spectrum of **3j**, related to **Figure 2**

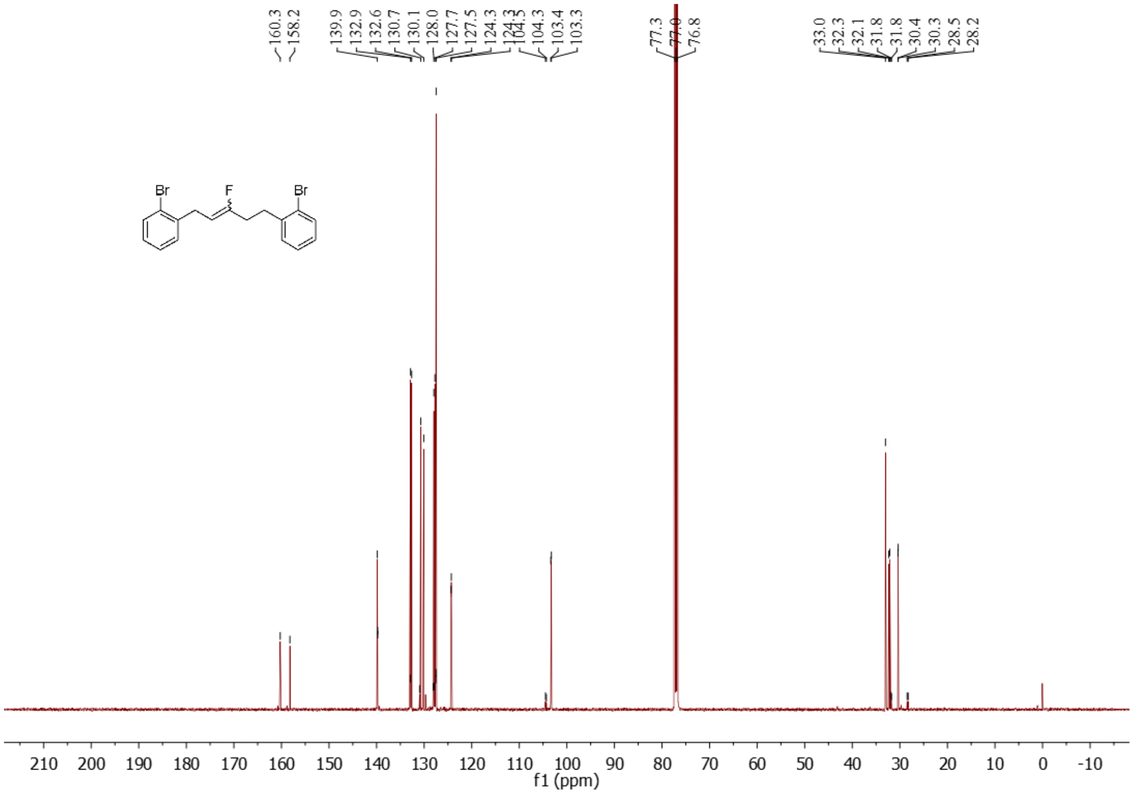

Figure S62. ^{19}F NMR spectrum of **3j**, related to **Figure 2**

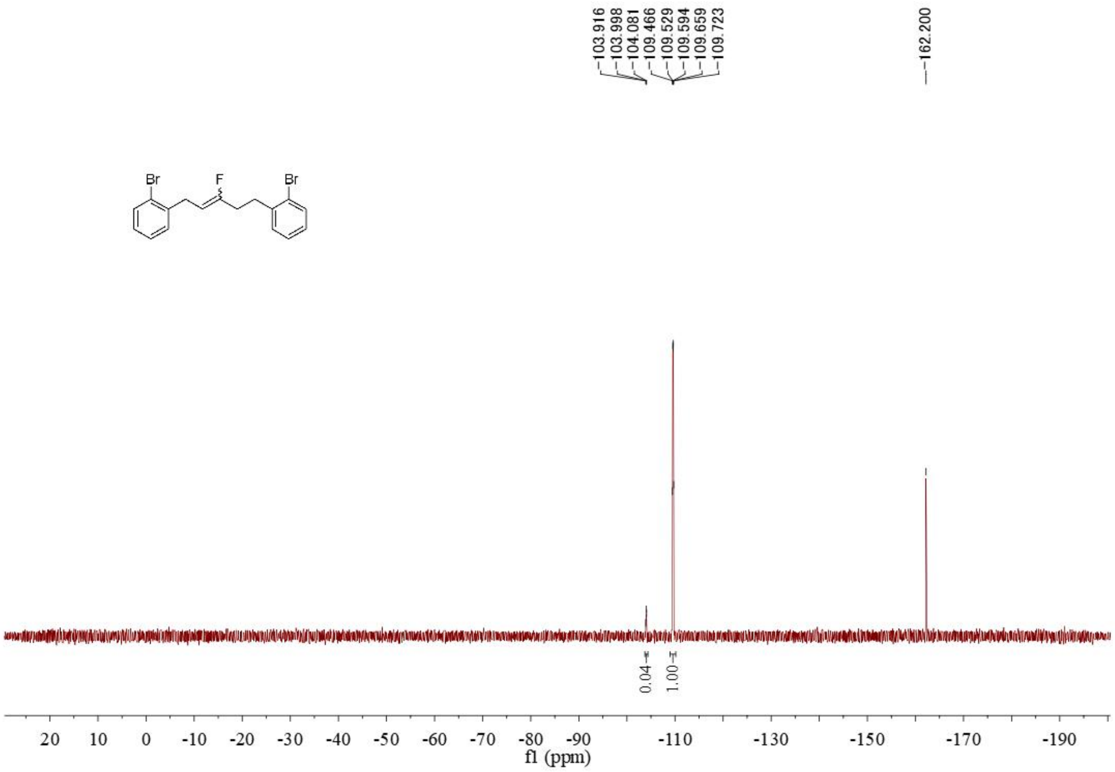

Figure S63. ^{1}H NMR spectrum of **3k**, related to **Figure 2**

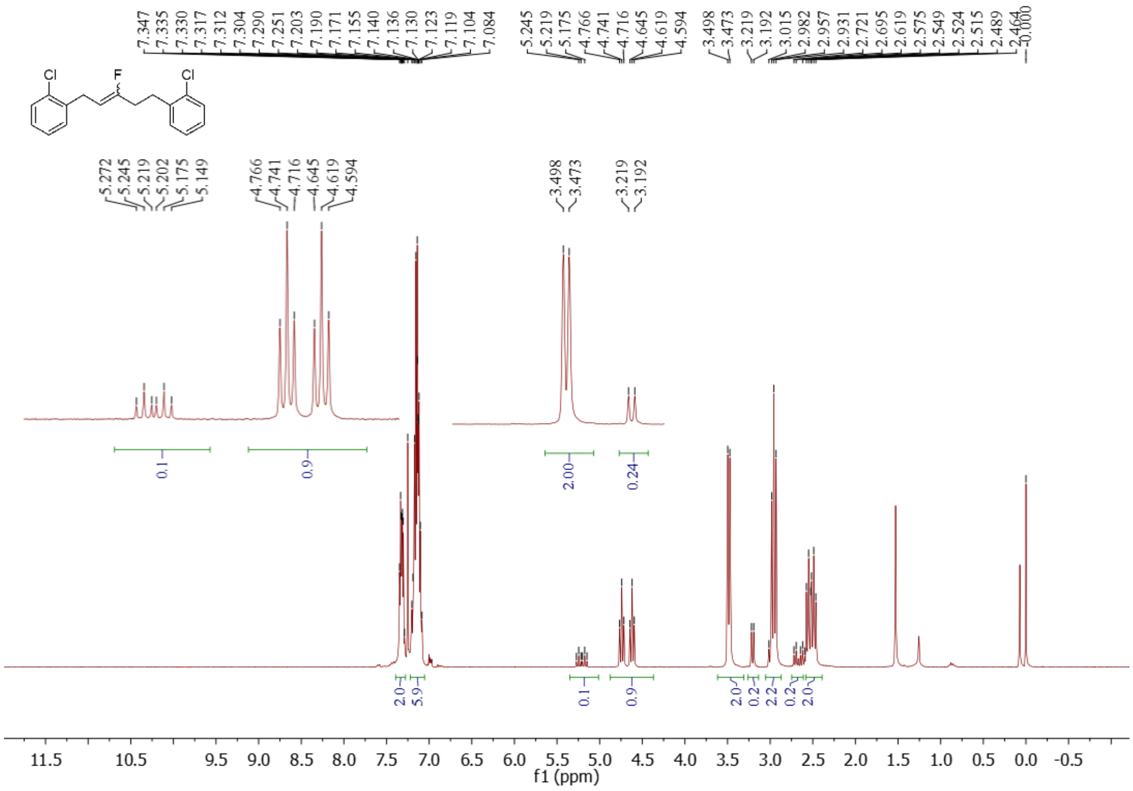

Figure S64. ^{13}C NMR spectrum of **3k**, related to **Figure 2**

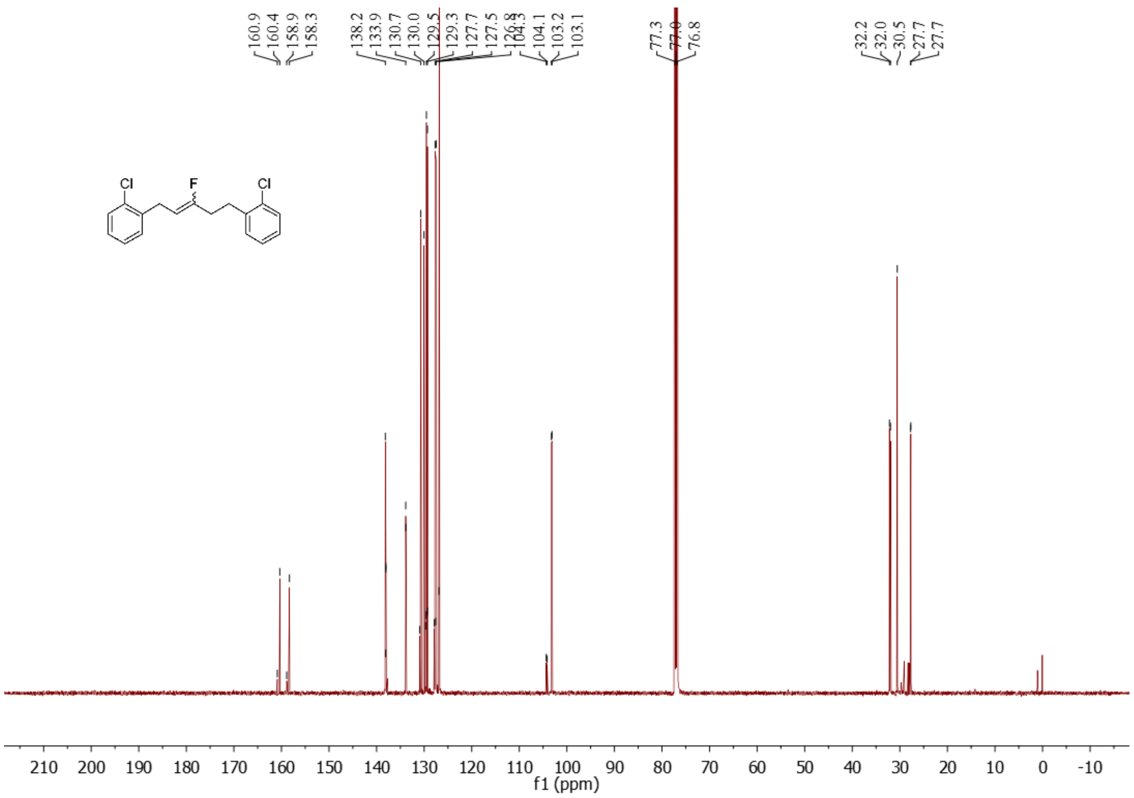

Figure S65. ^{19}F NMR spectrum of **3k**, related to **Figure 2**

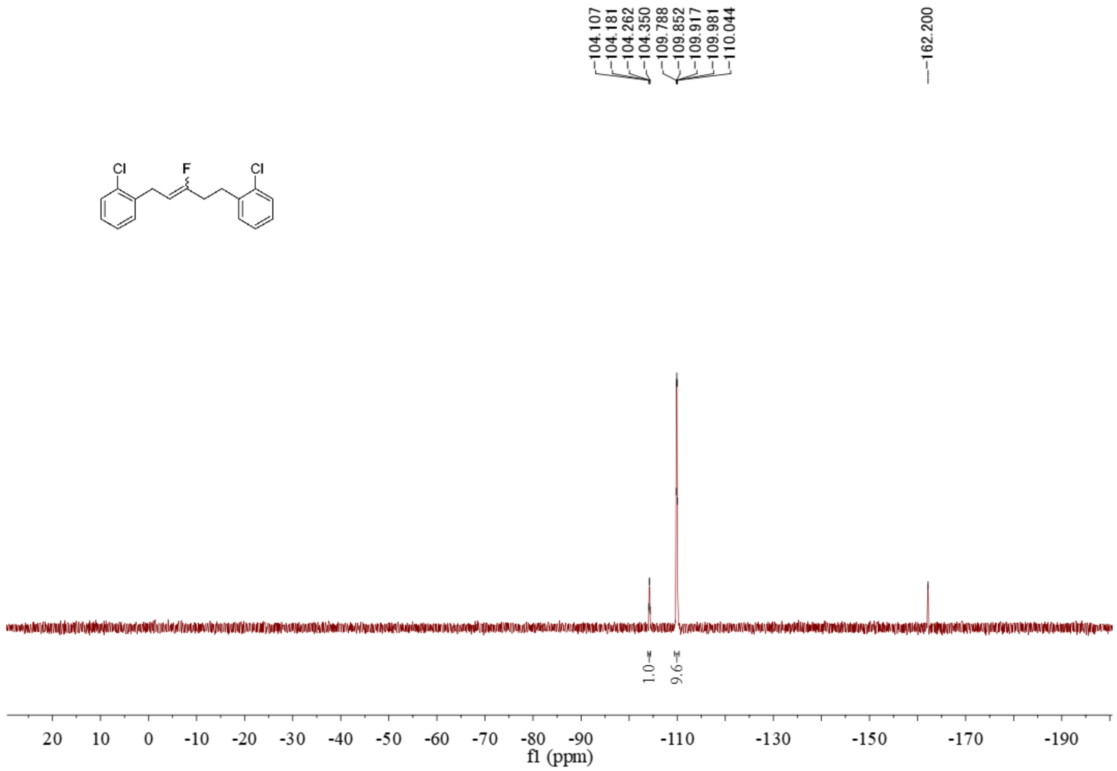

Figure S66. ^{1}H NMR spectrum of **3u**, related to **Figure 2**

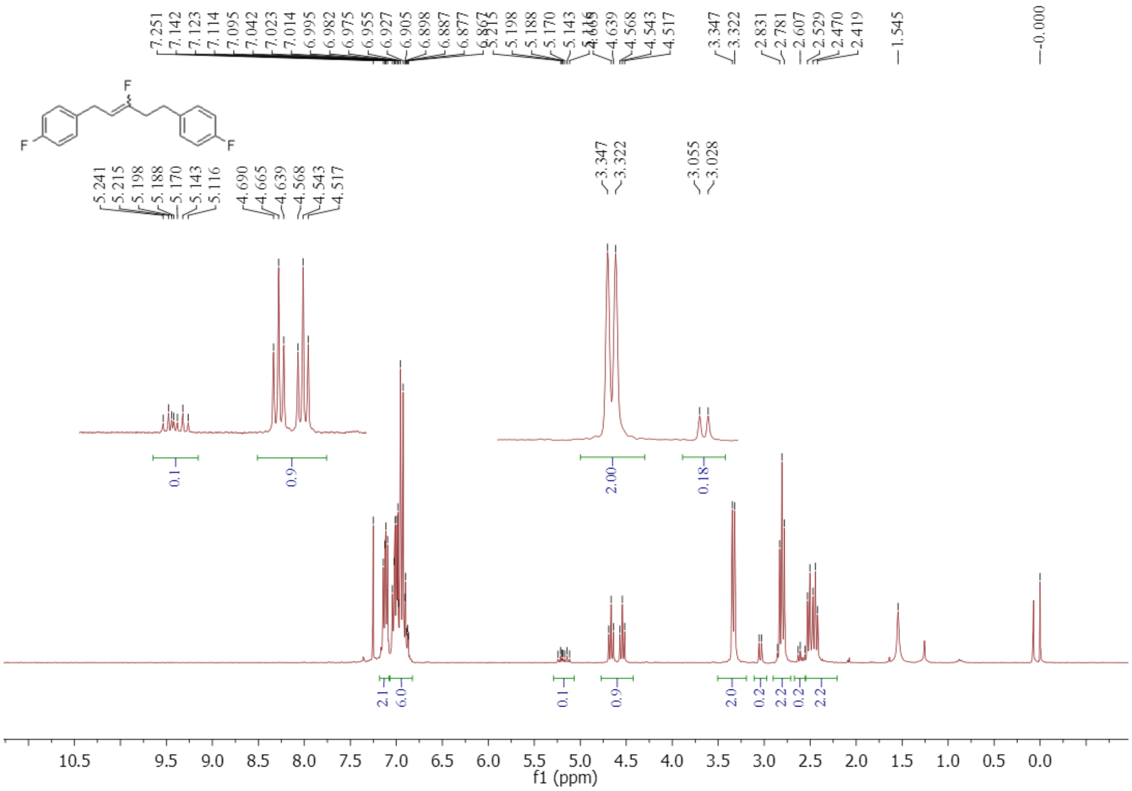

Figure S67. ^{13}C NMR spectrum of **3u**, related to **Figure 2**

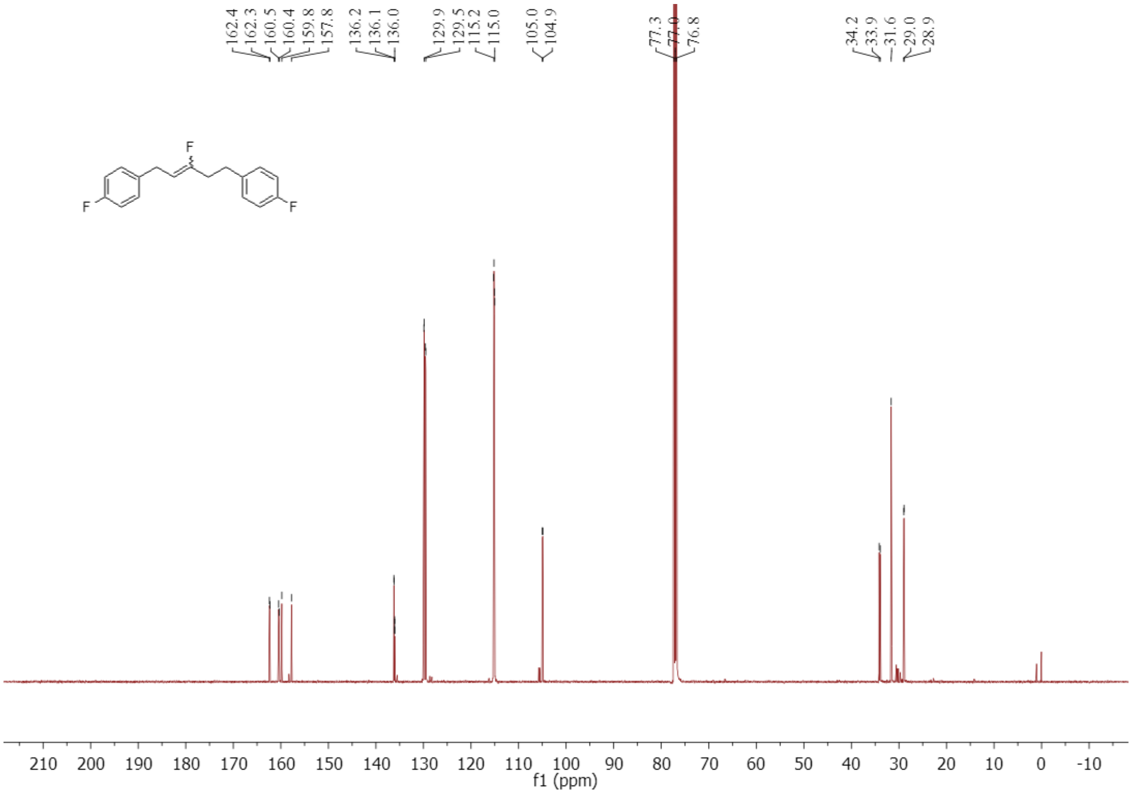

Figure S68. ^{19}F NMR spectrum of **3u**, related to **Figure 2**

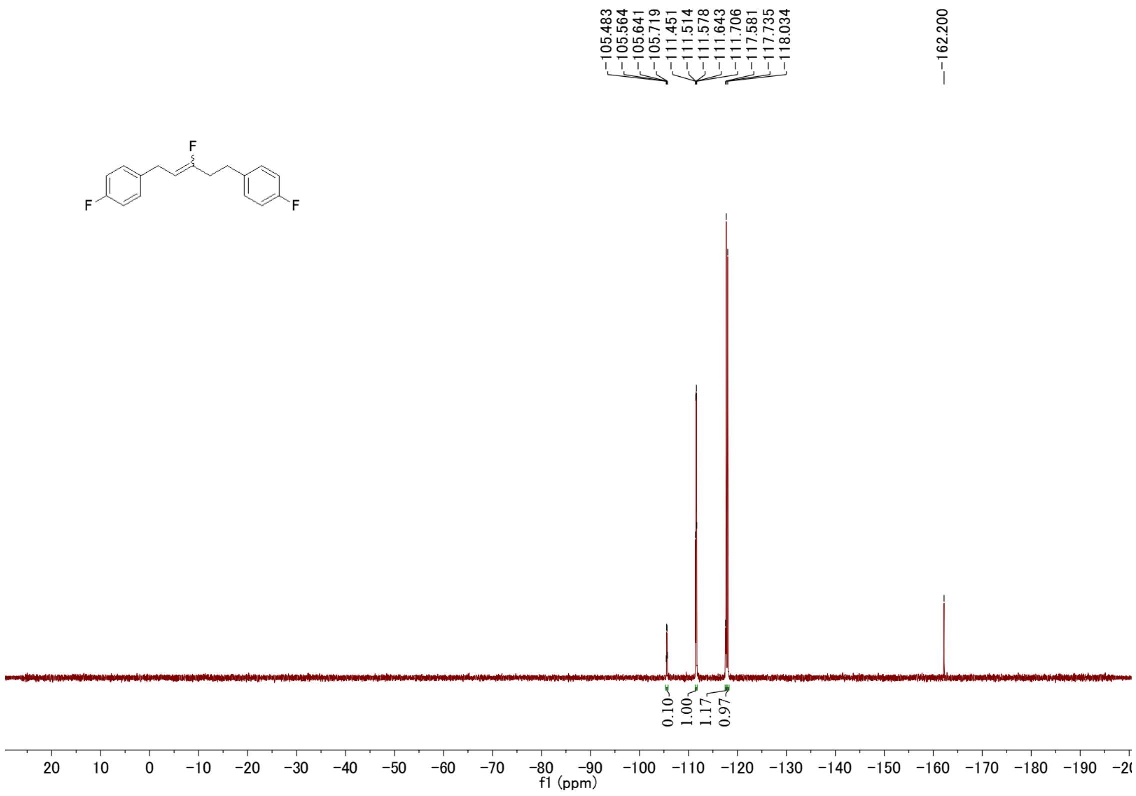

Figure S69. ^{1}H NMR spectrum of **3aa**, related to **Figure 2**

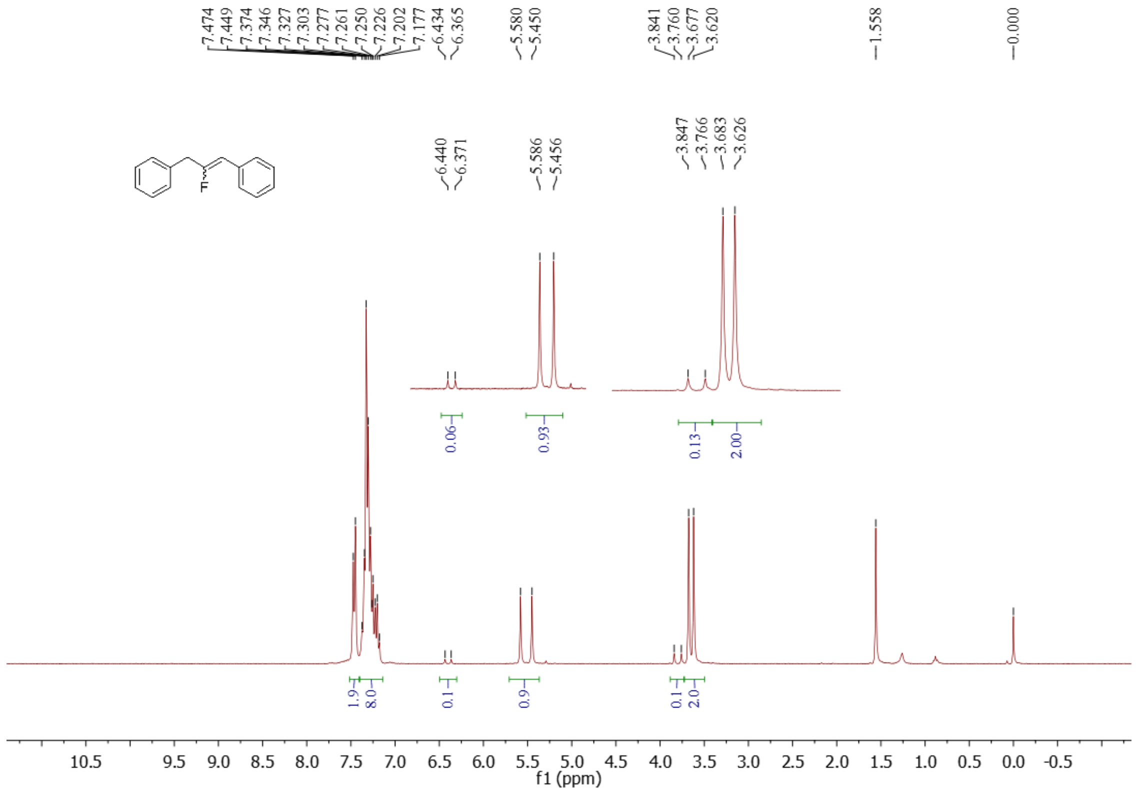

Figure S70. ^{19}F NMR spectrum of **3aa**, related to **Figure 2**

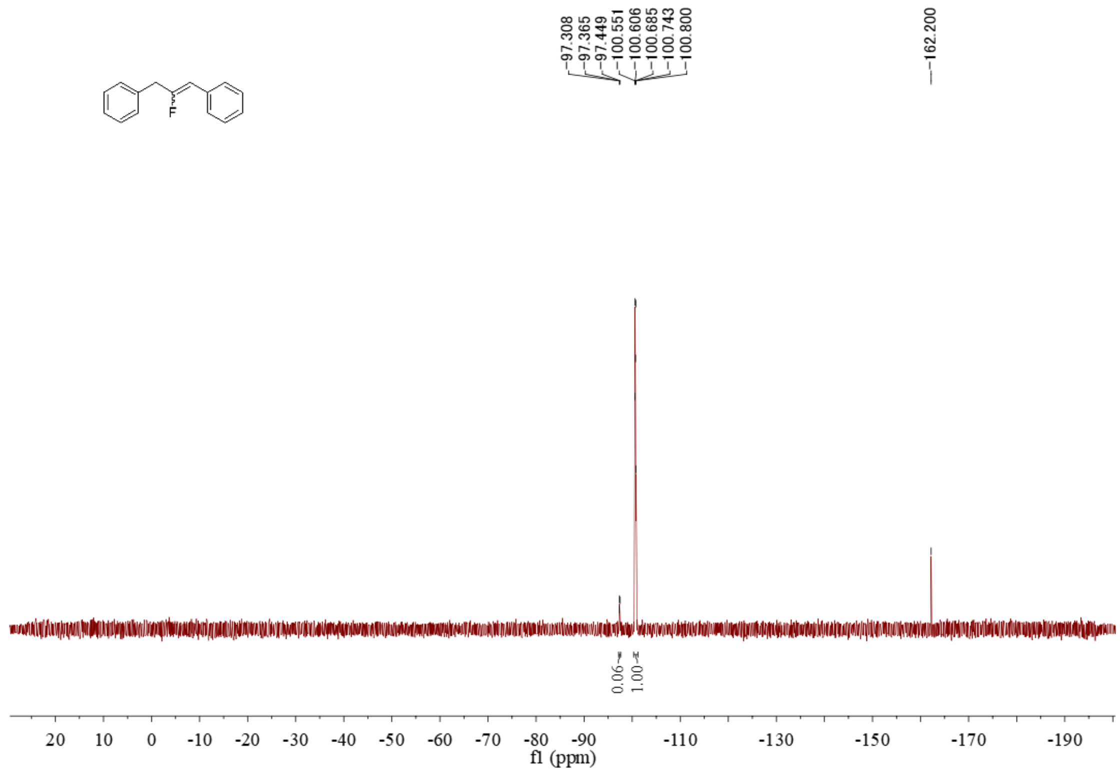

Figure S71. ^{1}H NMR spectrum of **3bb**, related to **Figure 2**

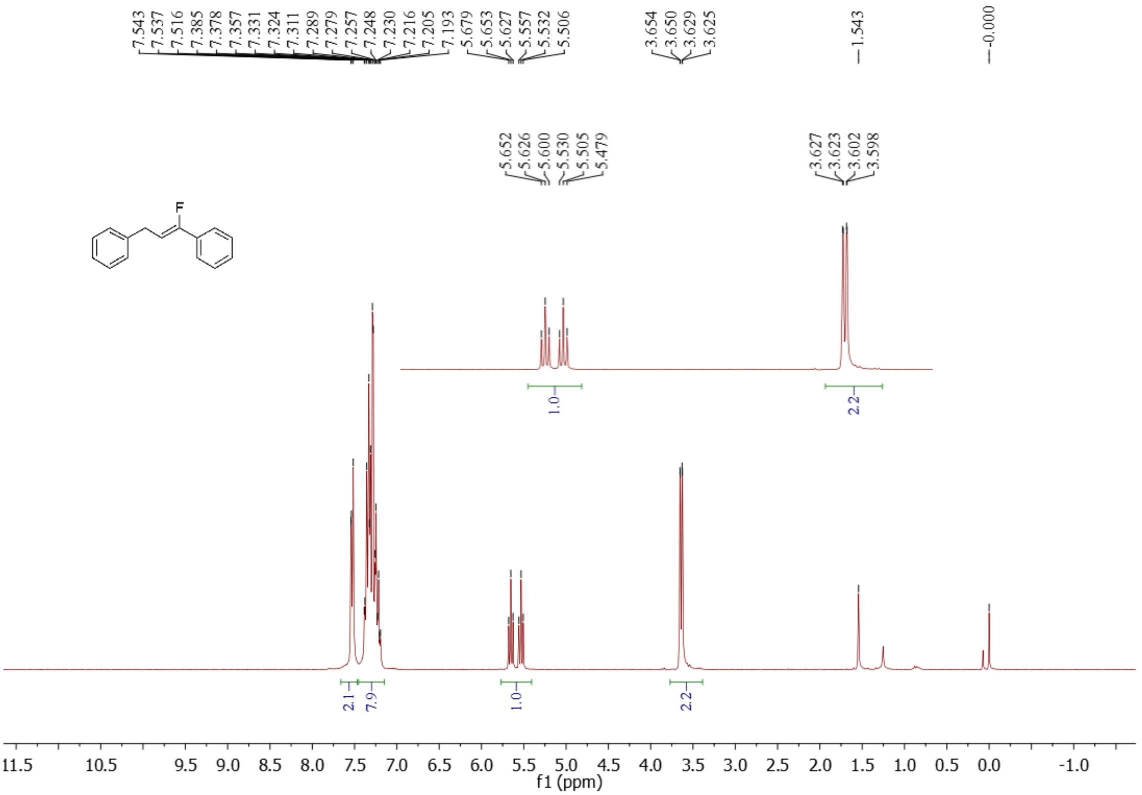

Figure S72. ^{19}F NMR spectrum of **3bb**, related to **Figure 2**

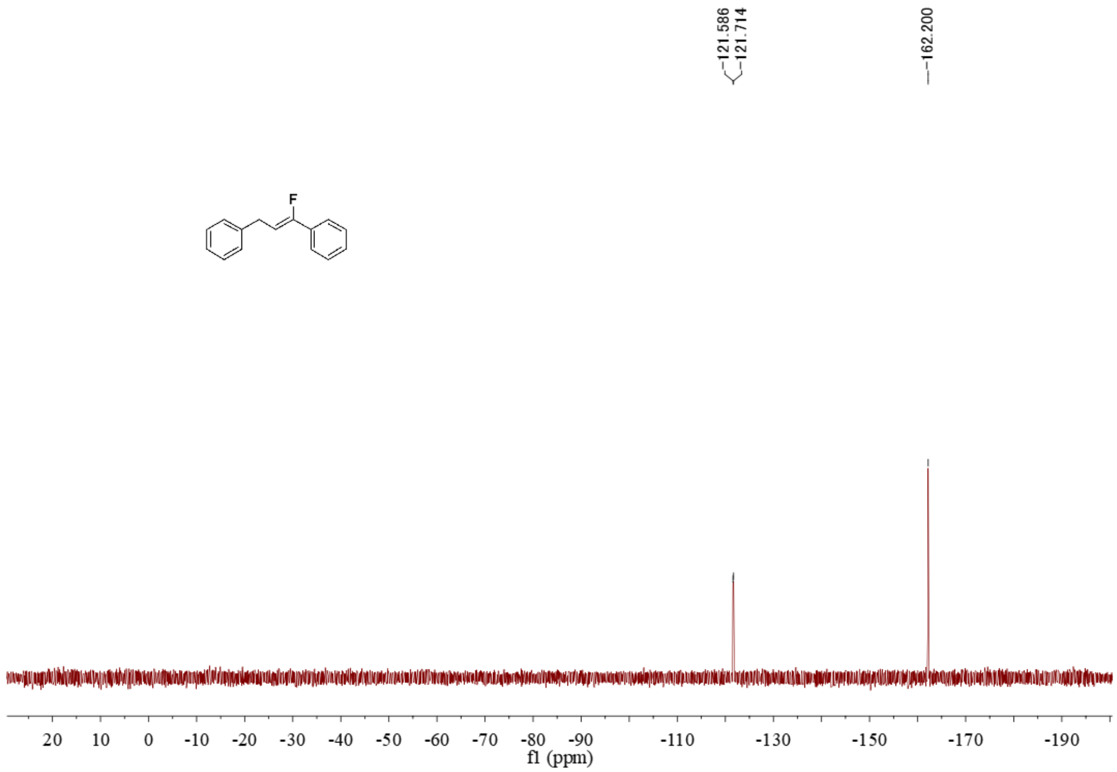

Figure S73. ^{1}H NMR spectrum of **3cc**, related to **Figure 2**

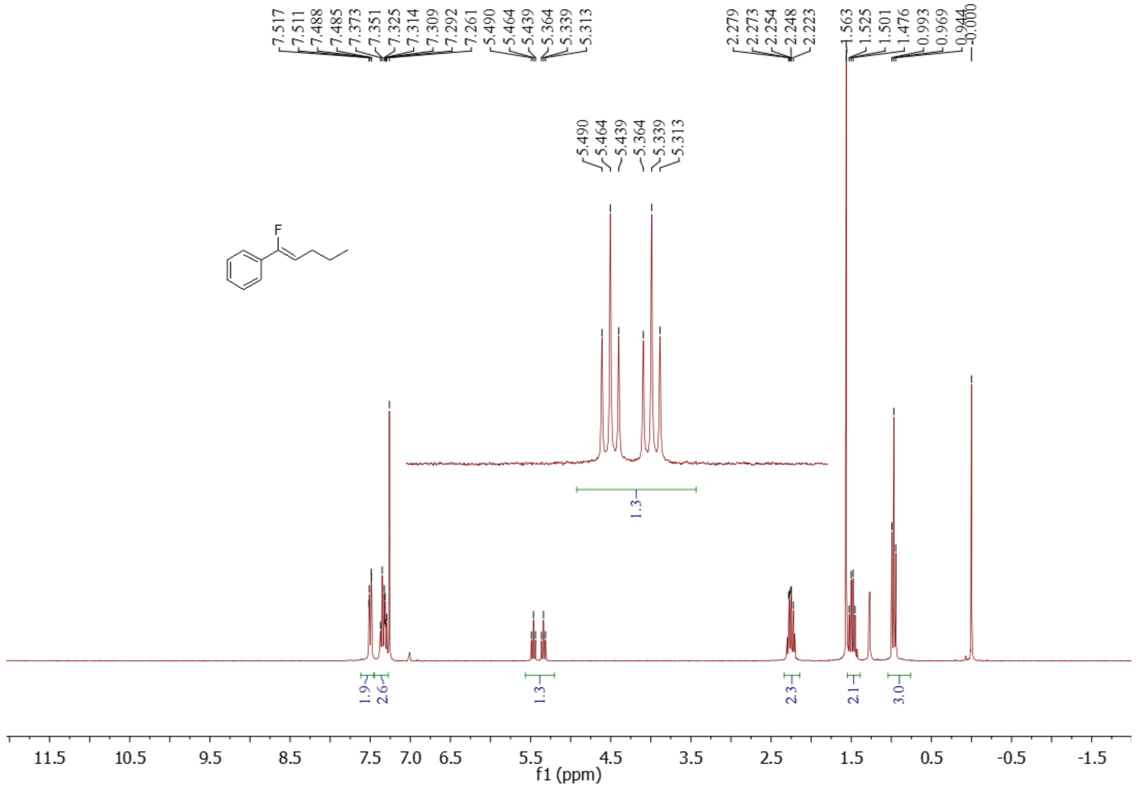

Figure S74. ^{19}F NMR spectrum of **3cc**, related to **Figure 2**

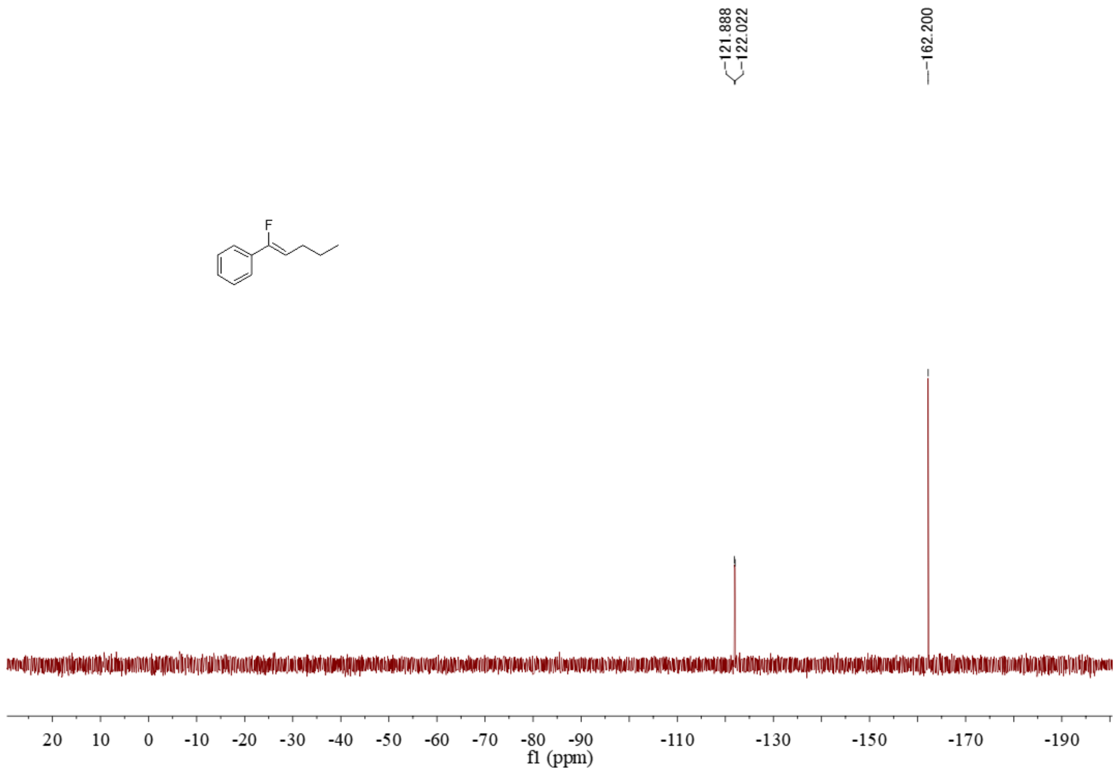

Figure S75. ^{1}H NMR spectrum of **3dd**, related to **Figure 2**

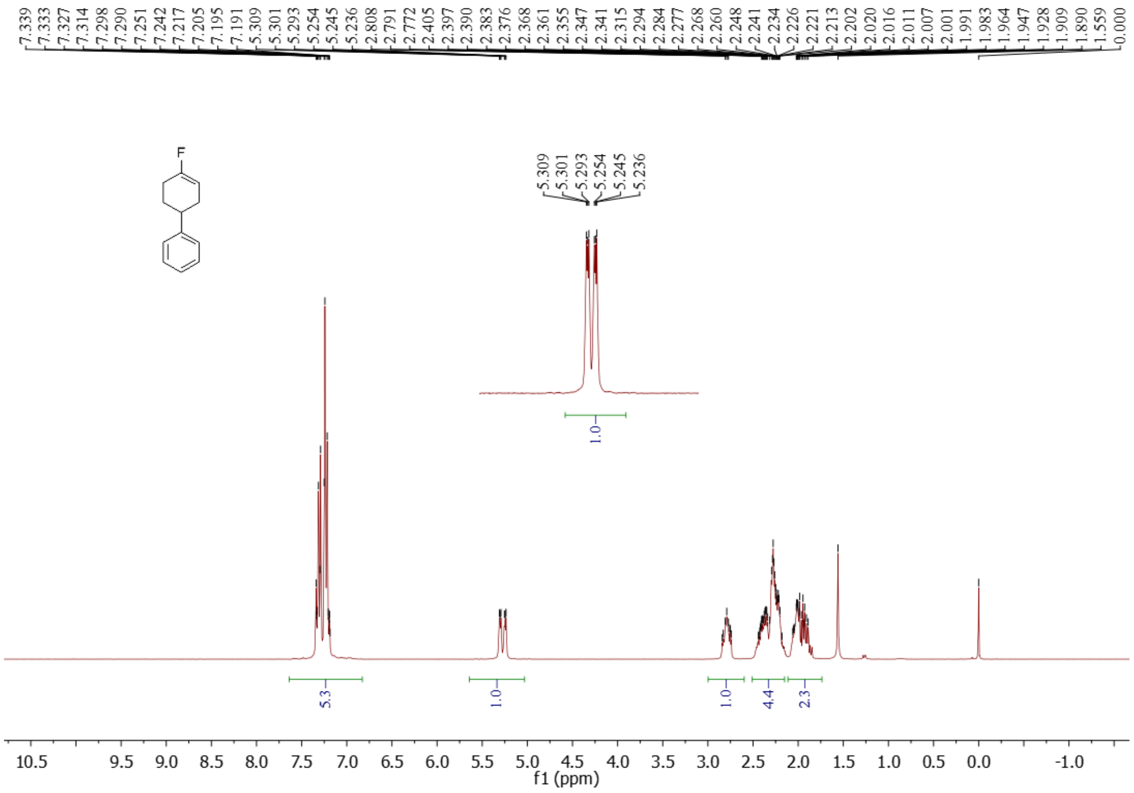

Figure S76. ^{19}F NMR spectrum of **3dd**, related to **Figure 2**

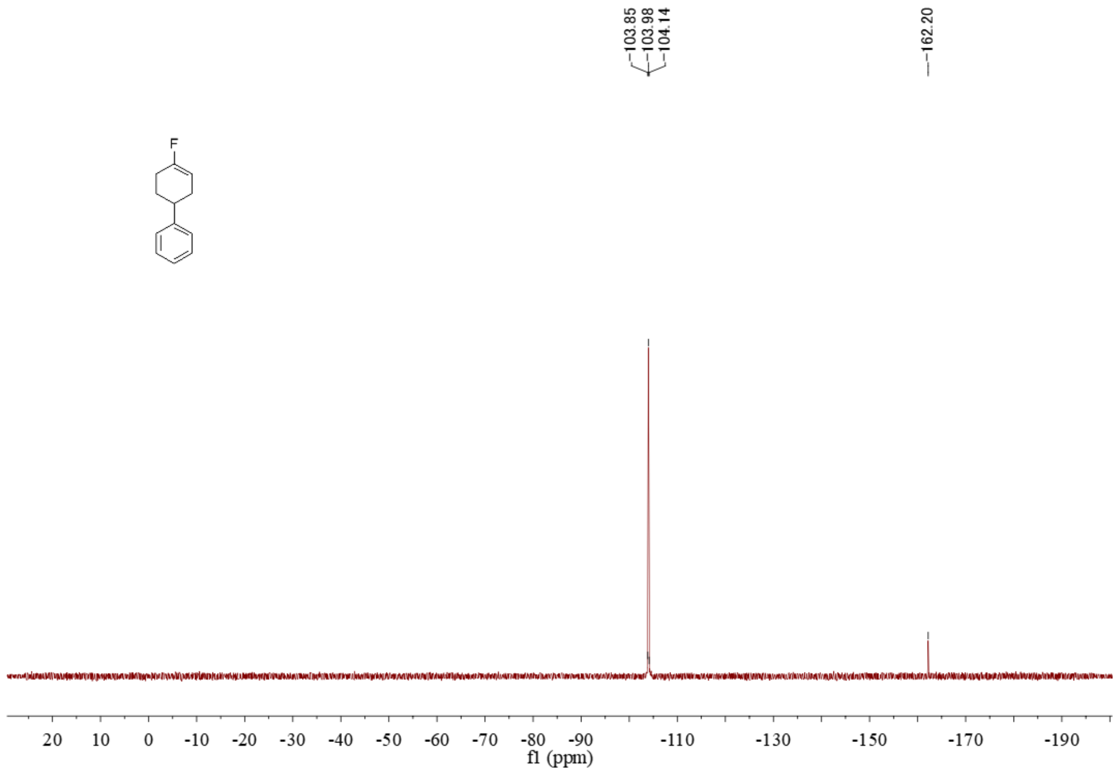

Figure S77. ^{1}H NMR spectrum of **3ee**, related to **Figure 2**

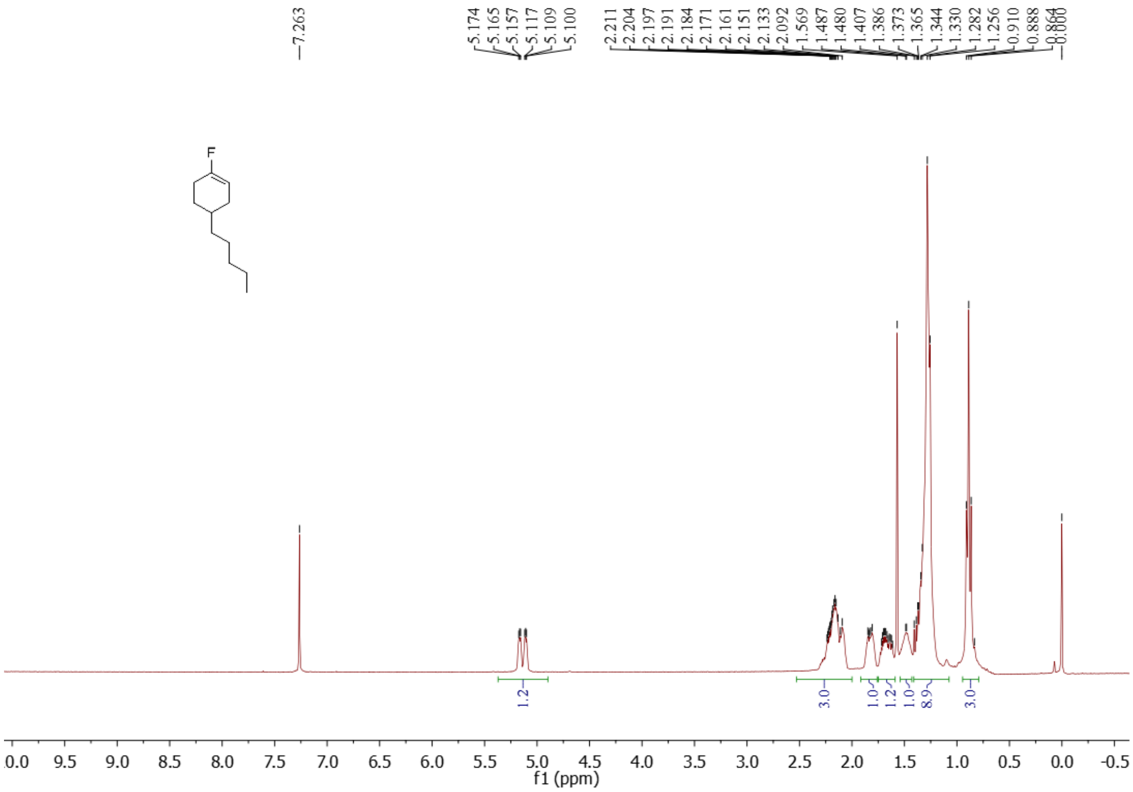

Figure S78. ^{19}F NMR spectrum of **3ee**, related to **Figure 2**

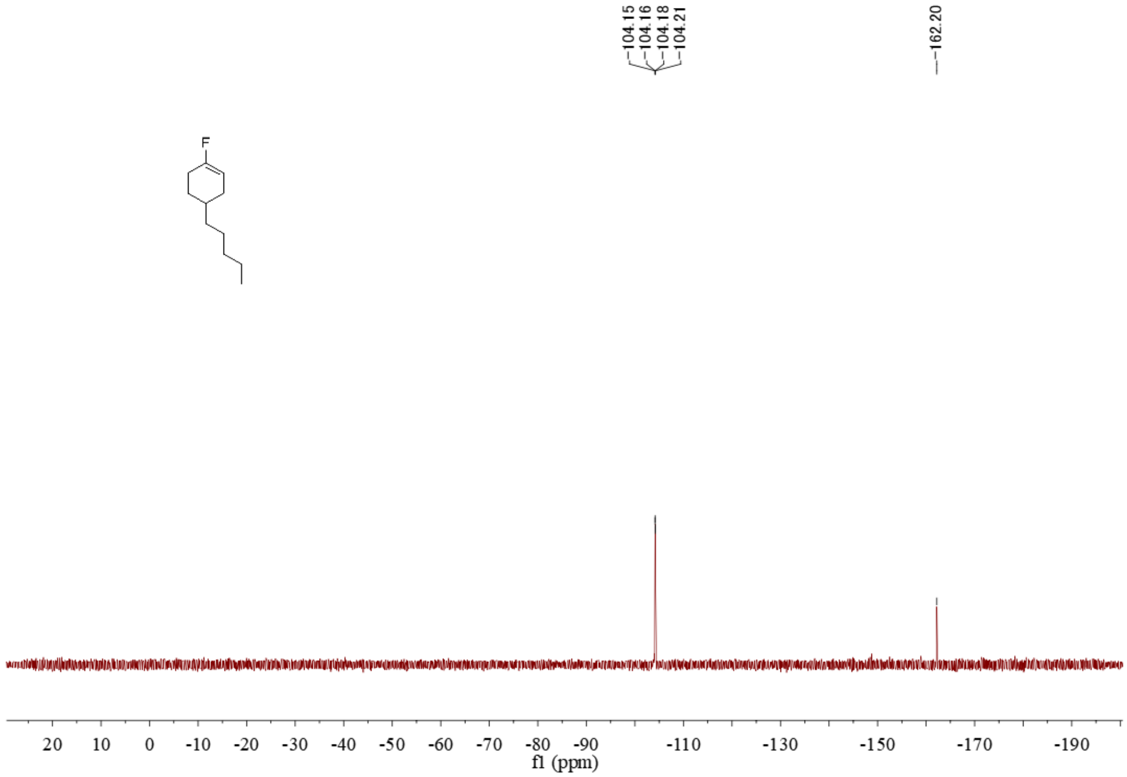

Figure S79. ^{1}H NMR spectrum of **3ff**, related to **Figure 2**

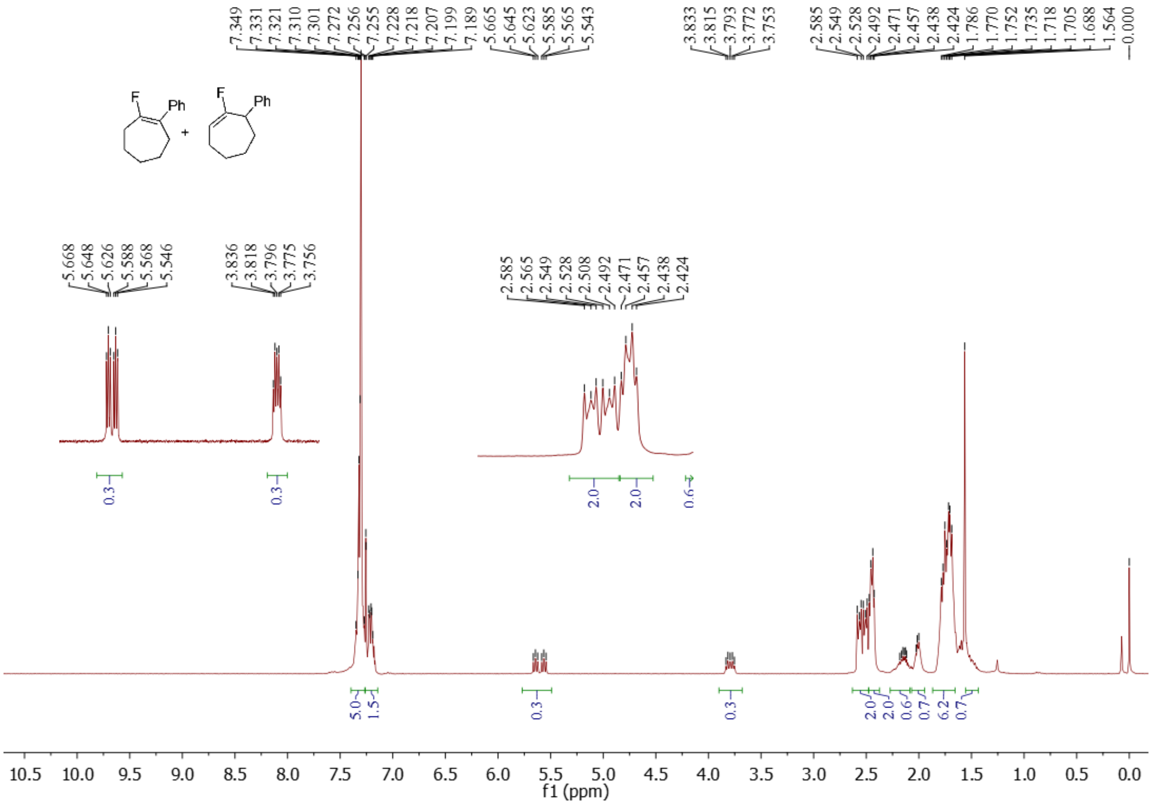

Figure S80. ^{13}C NMR spectrum of **3ff**, related to **Figure 2**

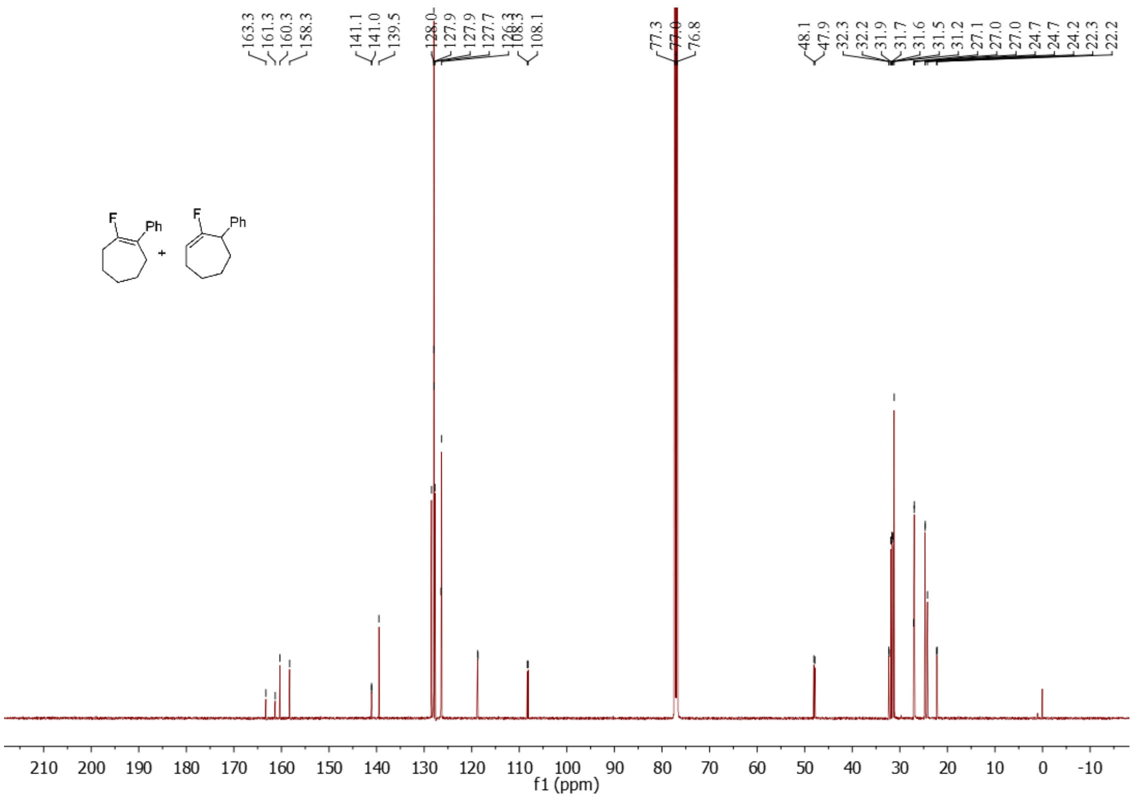

Figure S81. ^{19}F NMR spectrum of **3ff**, related to **Figure 2**

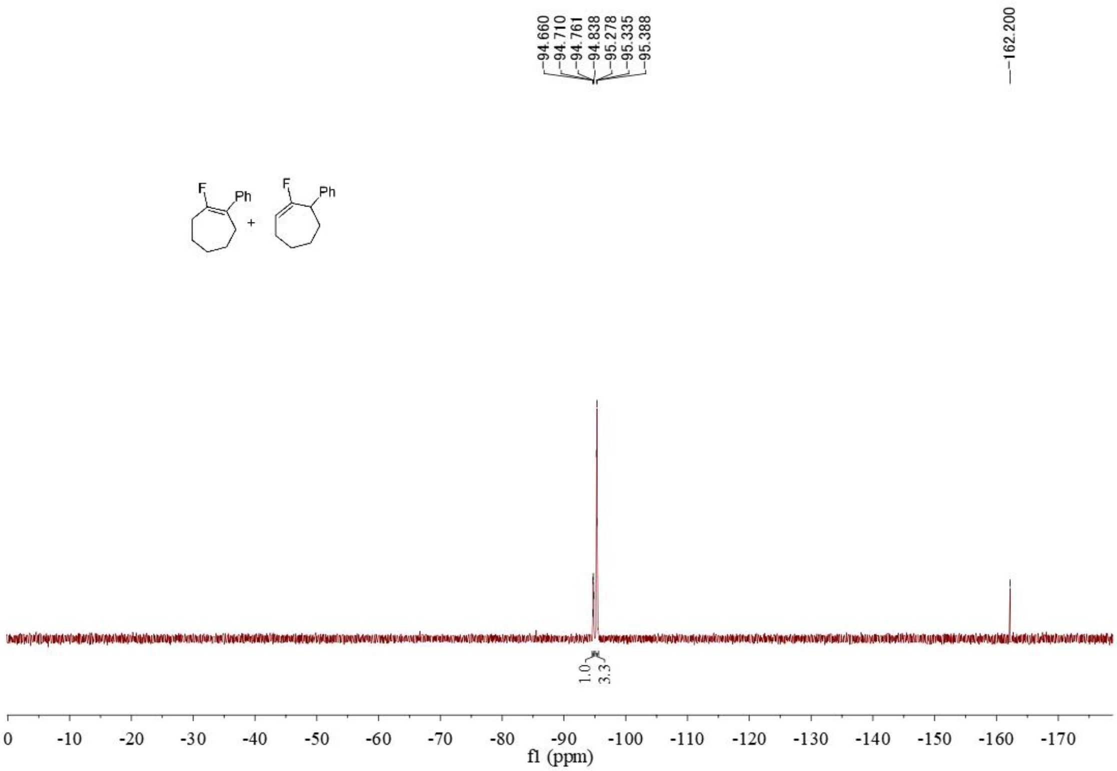

Figure S82. ^{1}H NMR spectrum of **3gg**, related to **Figure 2**

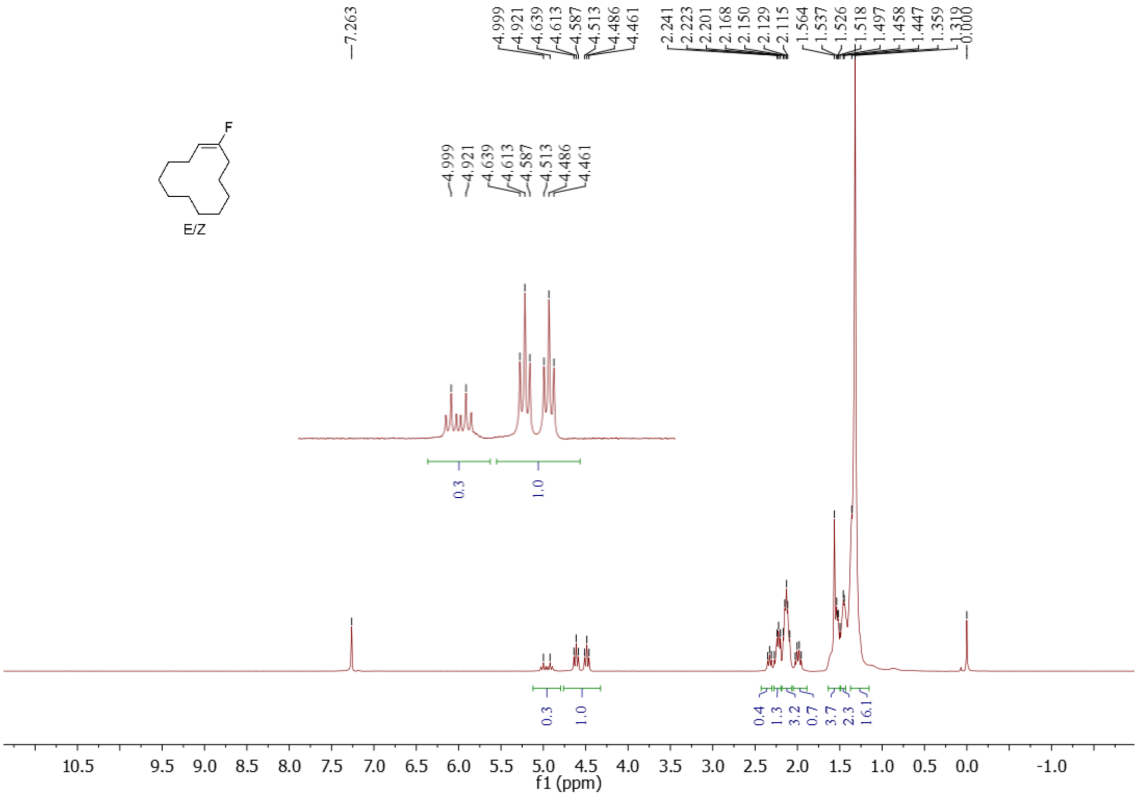

Figure S83. ^{13}C NMR spectrum of **3gg**, related to **Figure 2**

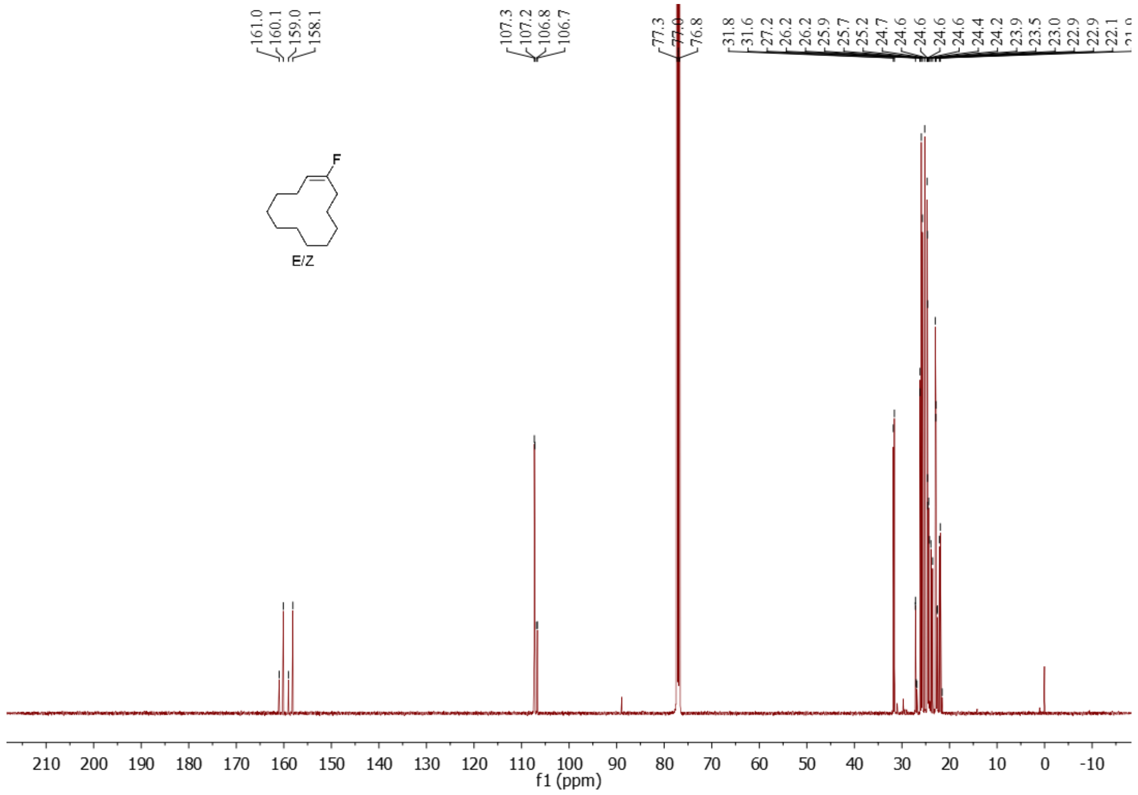

Figure S84. ^{19}F NMR spectrum of **3gg**, related to **Figure 2**

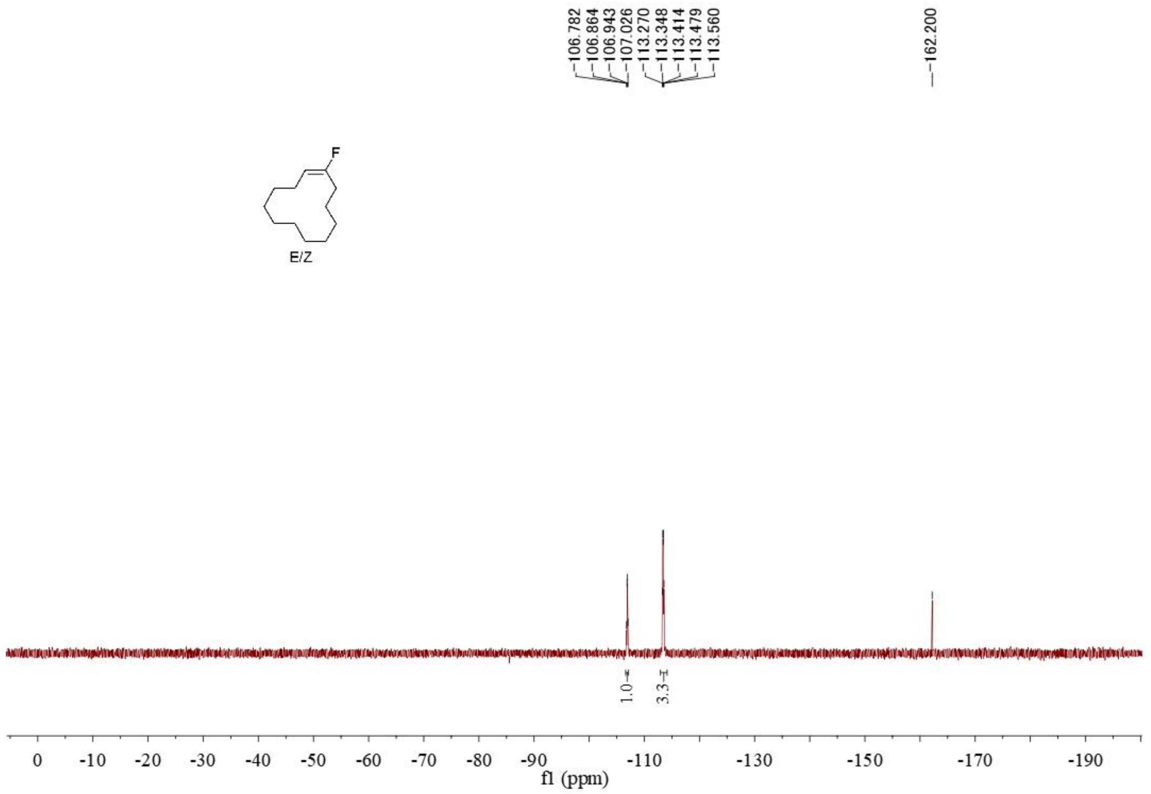

Figure S85. ^{1}H NMR spectrum of (*Z*)-**3hh**, related to **Figure 2**

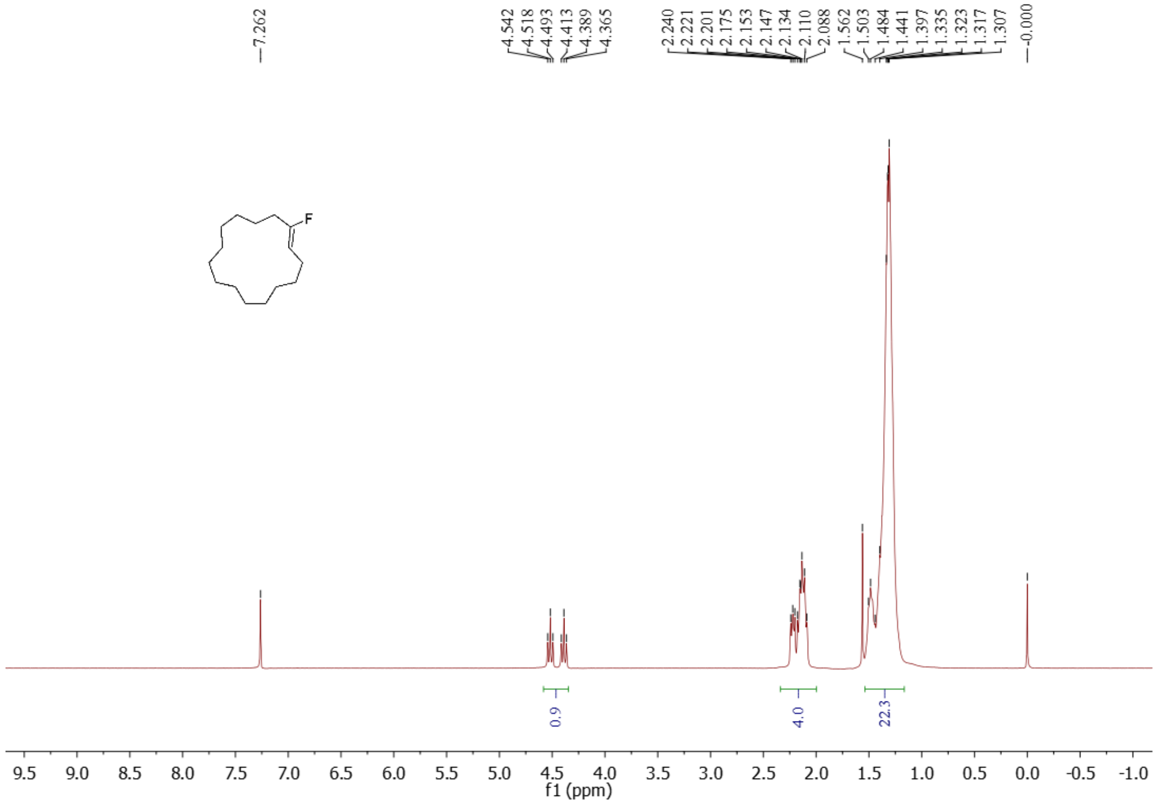

Figure S86. ^{13}C NMR spectrum of (*Z*)-**3hh**, related to **Figure 2**

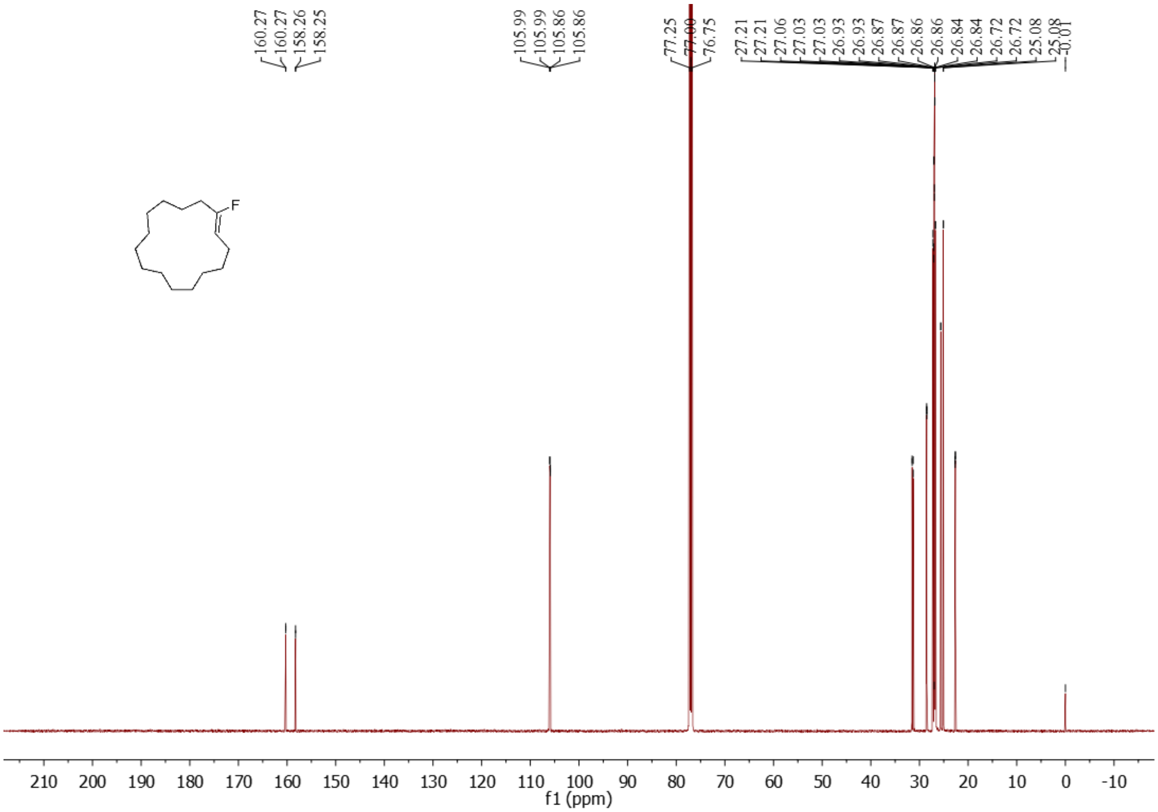

Figure S87. ^{19}F NMR spectrum of (*Z*)-**3hh**, related to **Figure 2**

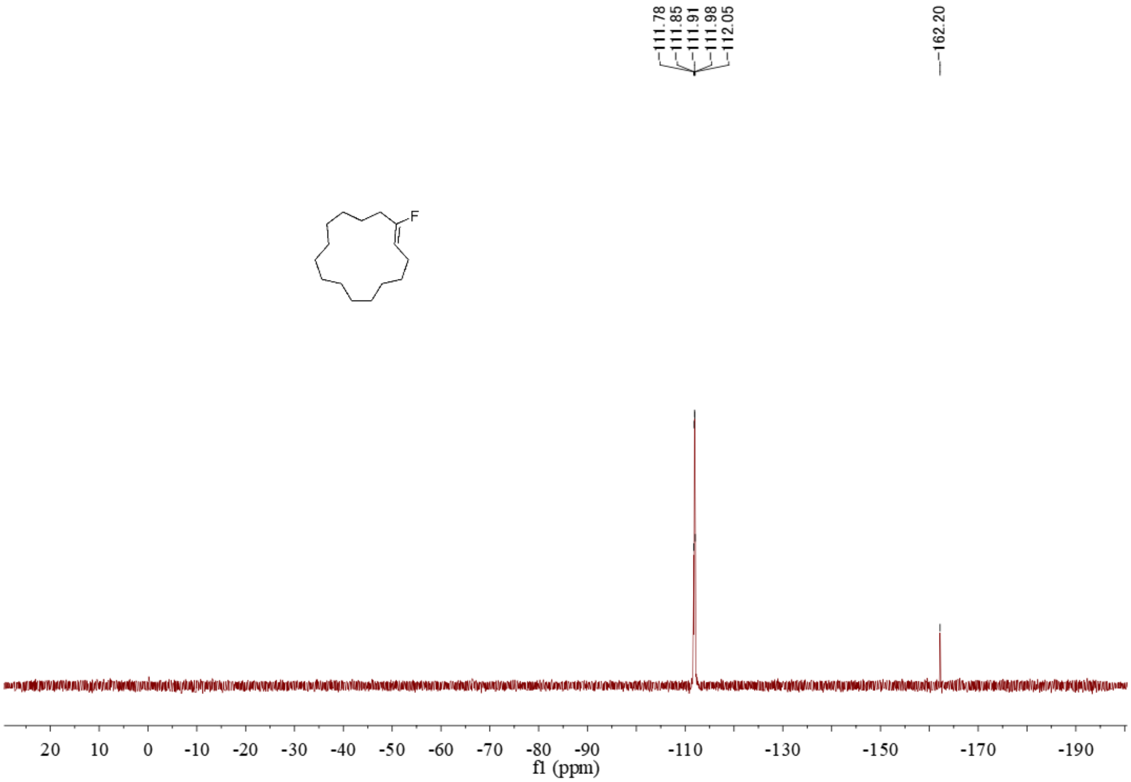

Figure S88. ^{19}F NMR spectrum of **3hh**, related to **Figure 2**

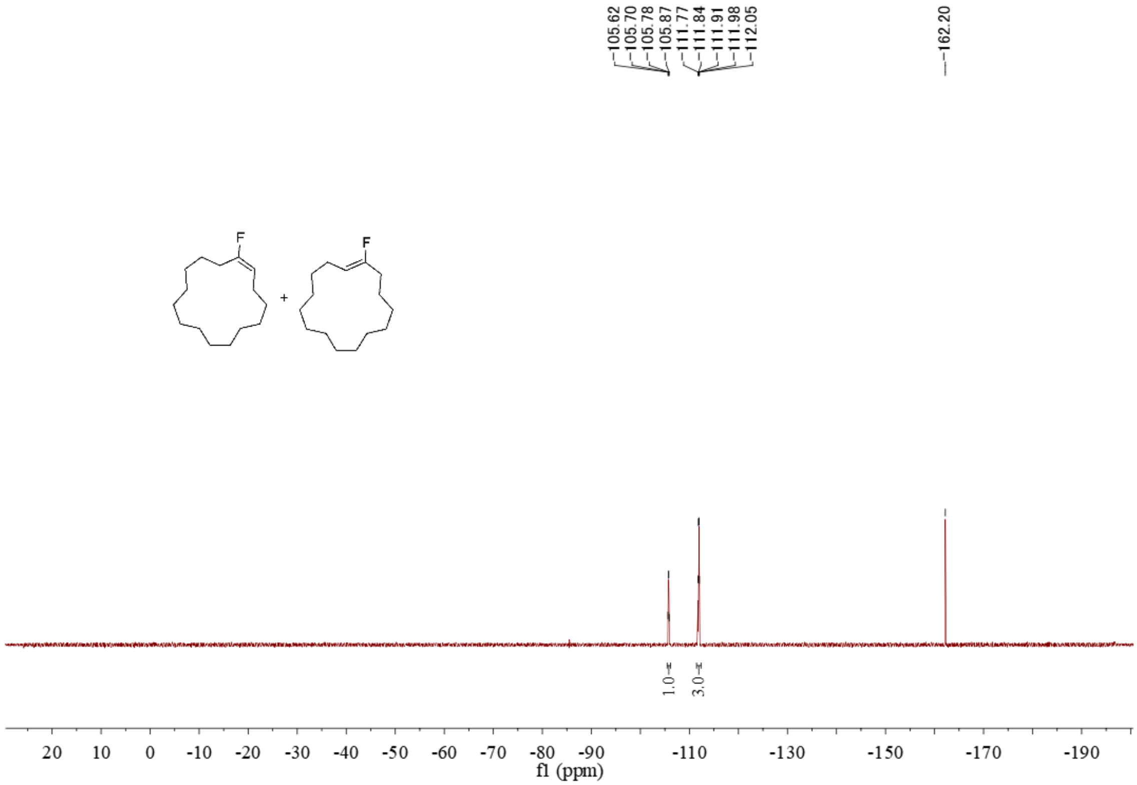

Figure S89. ^{1}H NMR spectrum of **5a**, related to **Figure 3**

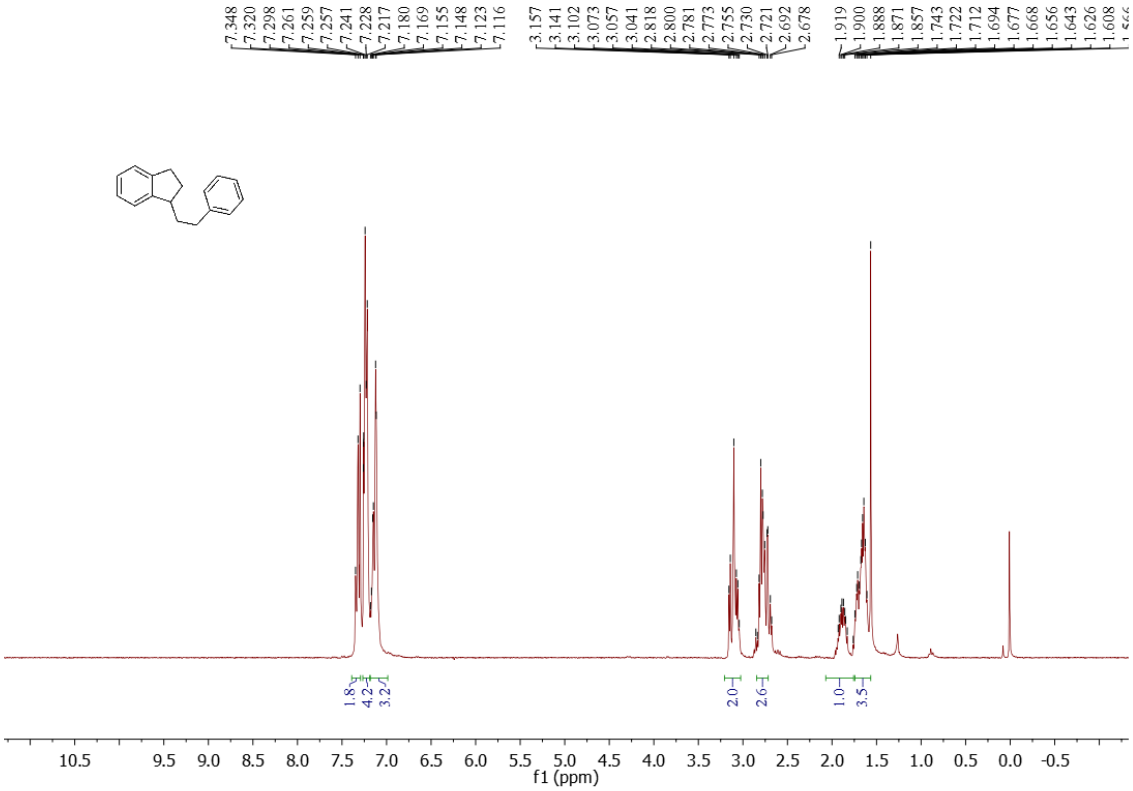

Figure S90. ^{13}C NMR spectrum of **5a**, related to **Figure 3**

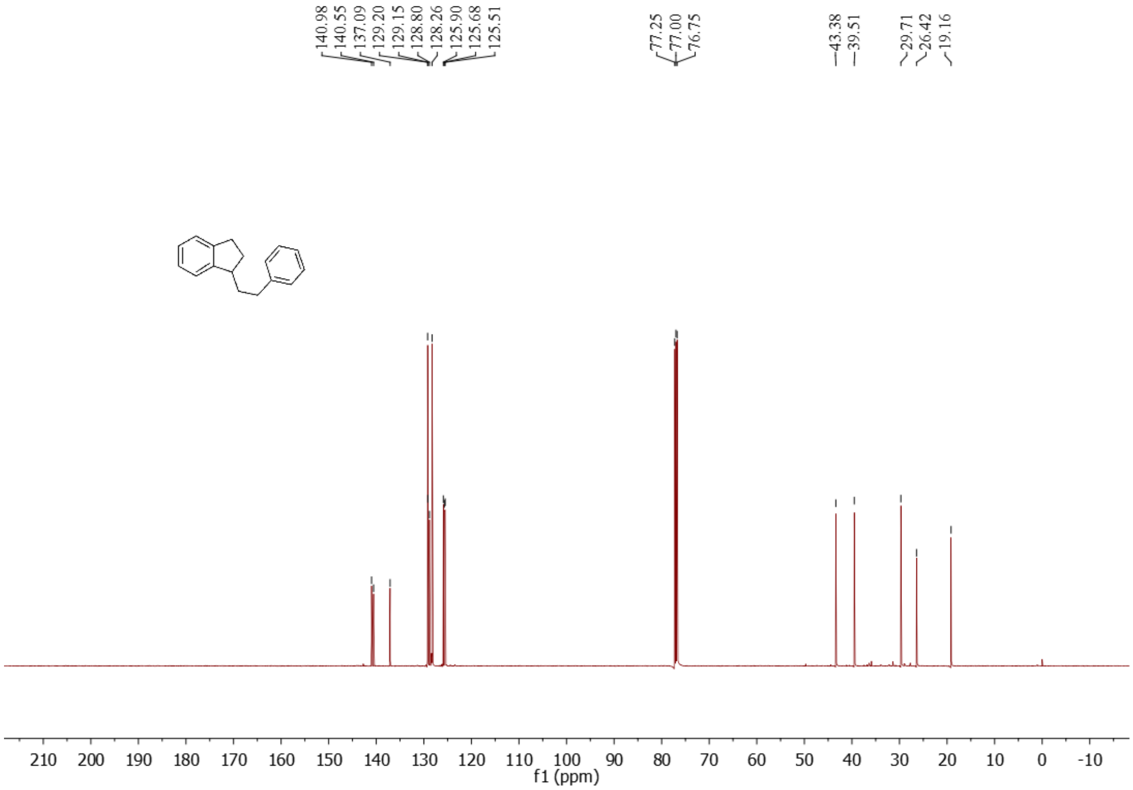

Figure S91. ^{1}H NMR spectrum of **5b**, related to **Figure 3**

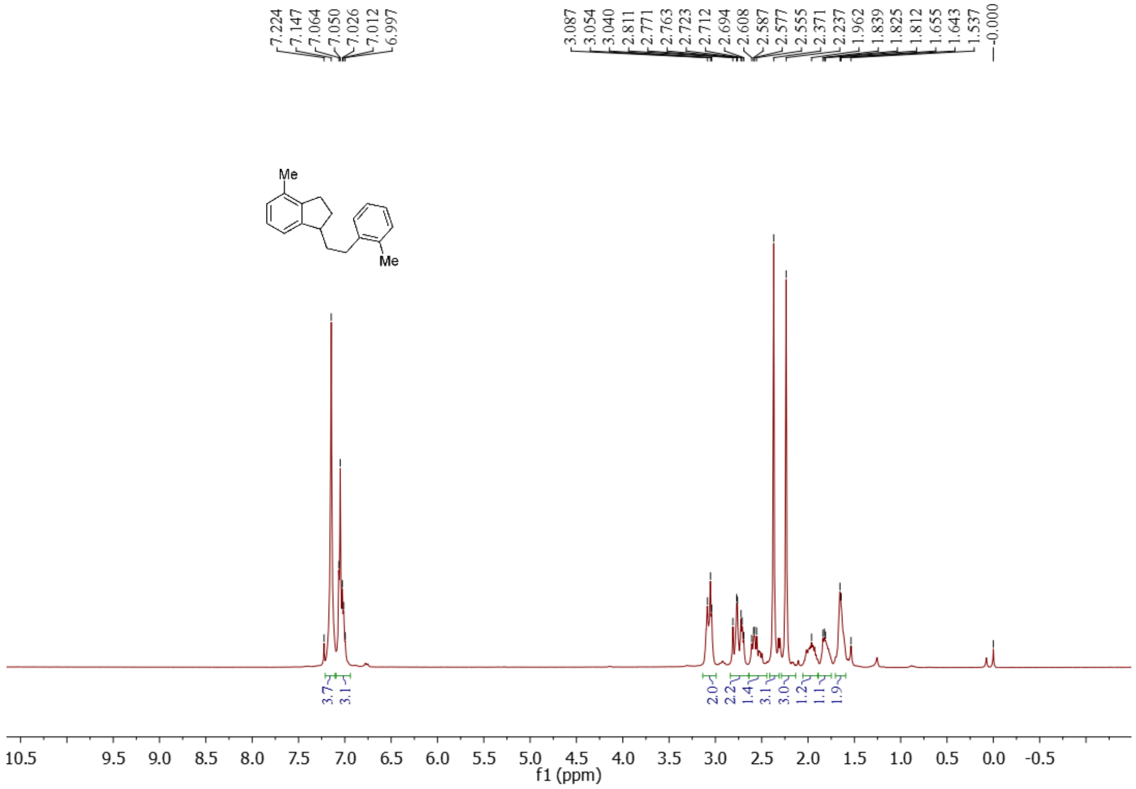

Figure S92. ^{13}C NMR spectrum of **5b**, related to **Figure 3**

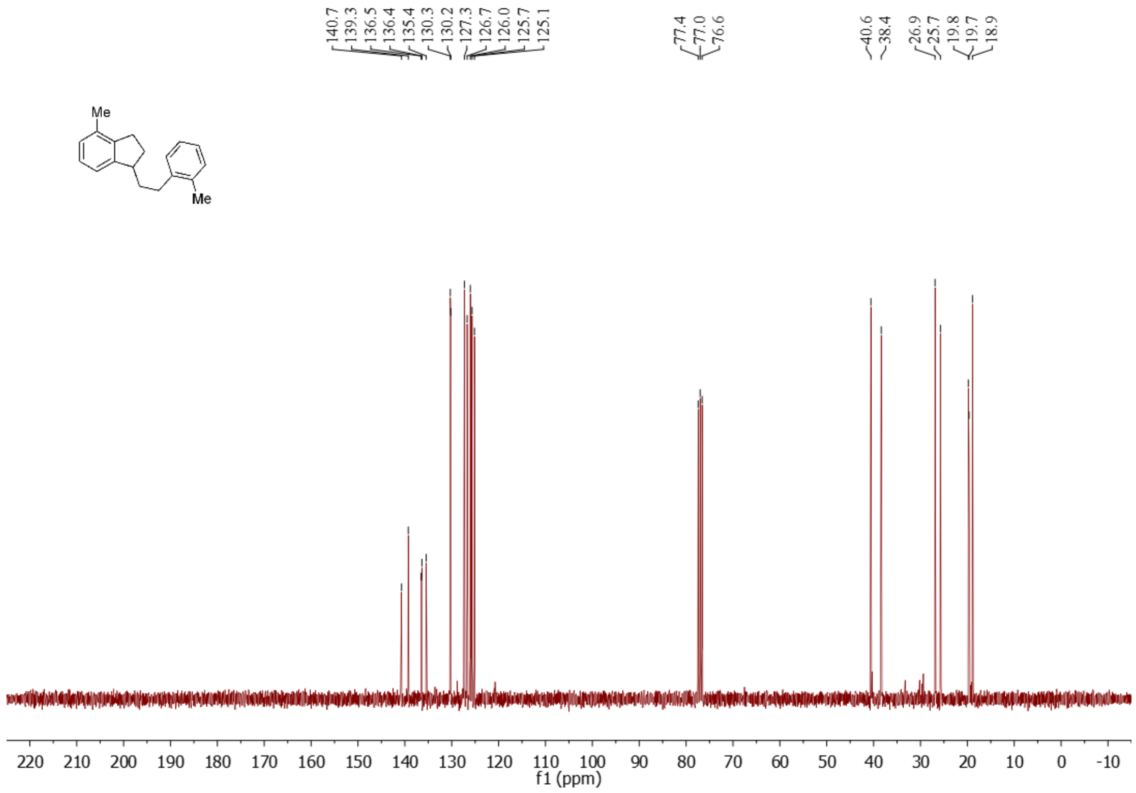

Figure S93. ^{1}H NMR spectrum of **5c**, related to **Figure 3**

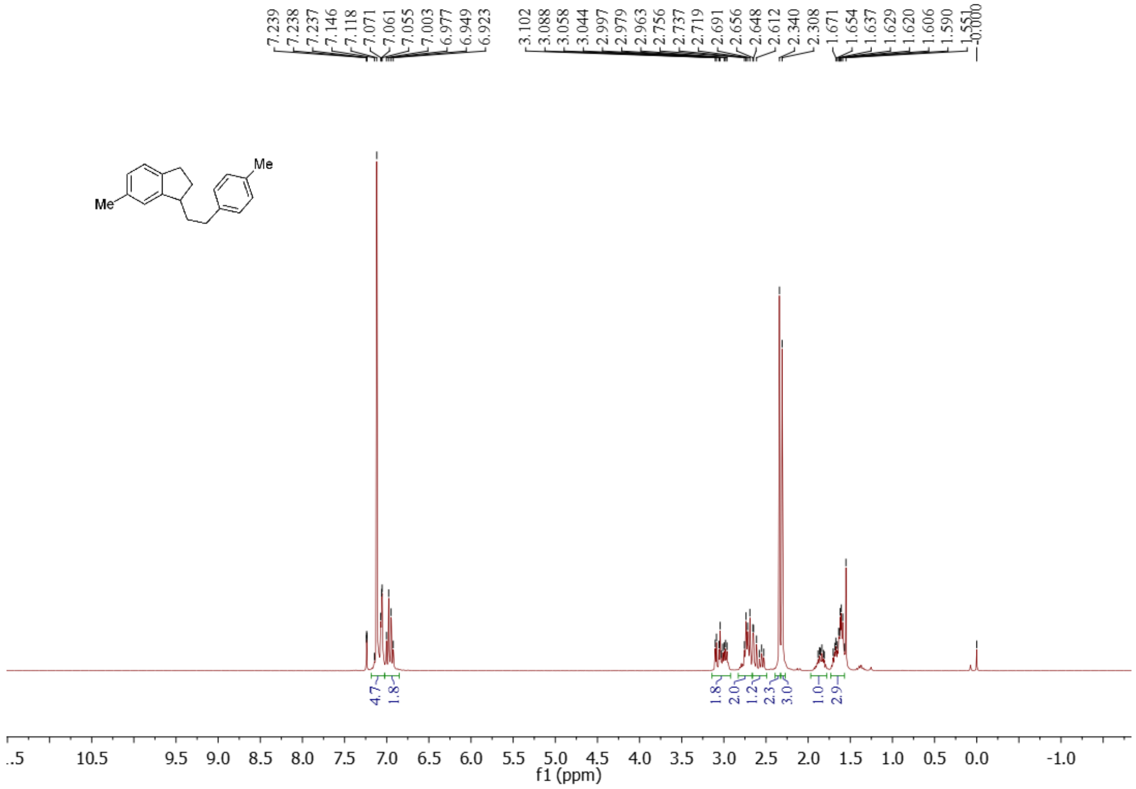

Figure S94. ^{13}C NMR spectrum of **5c**, related to **Figure 3**

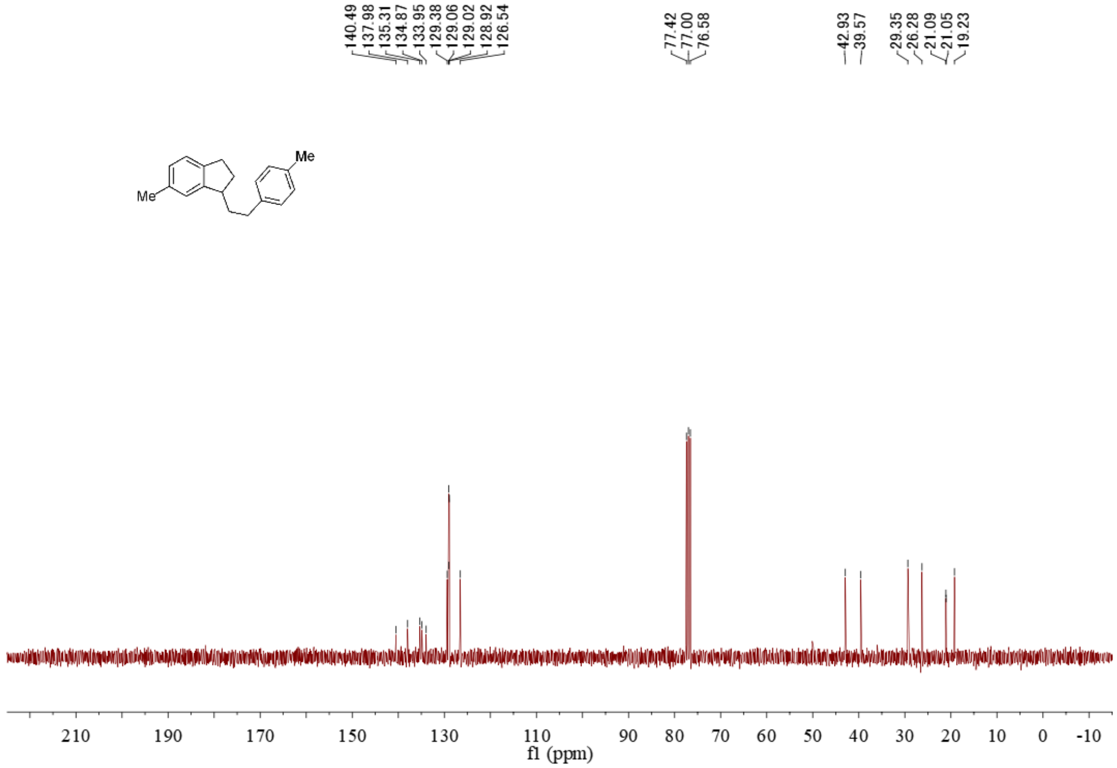

Figure S95. ^{1}H NMR spectrum of **5d**, related to **Figure 3**

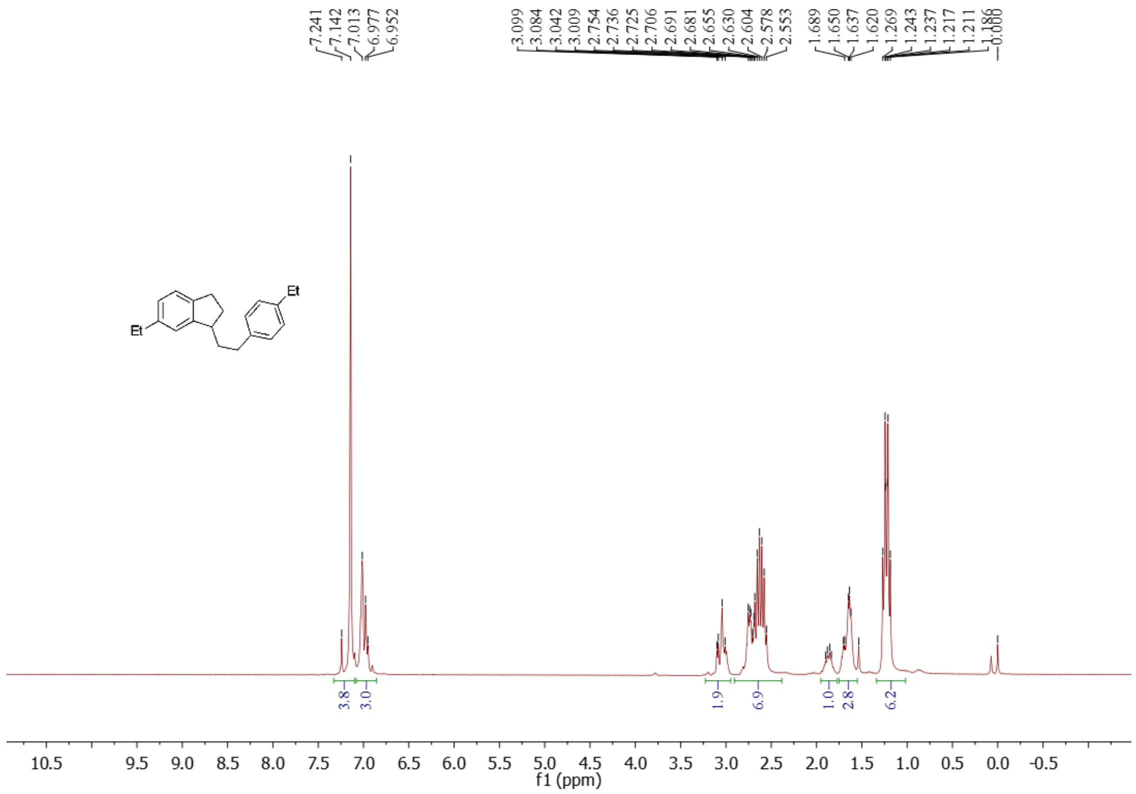

Figure S96. ^{13}C NMR spectrum of **5d**, related to **Figure 3**

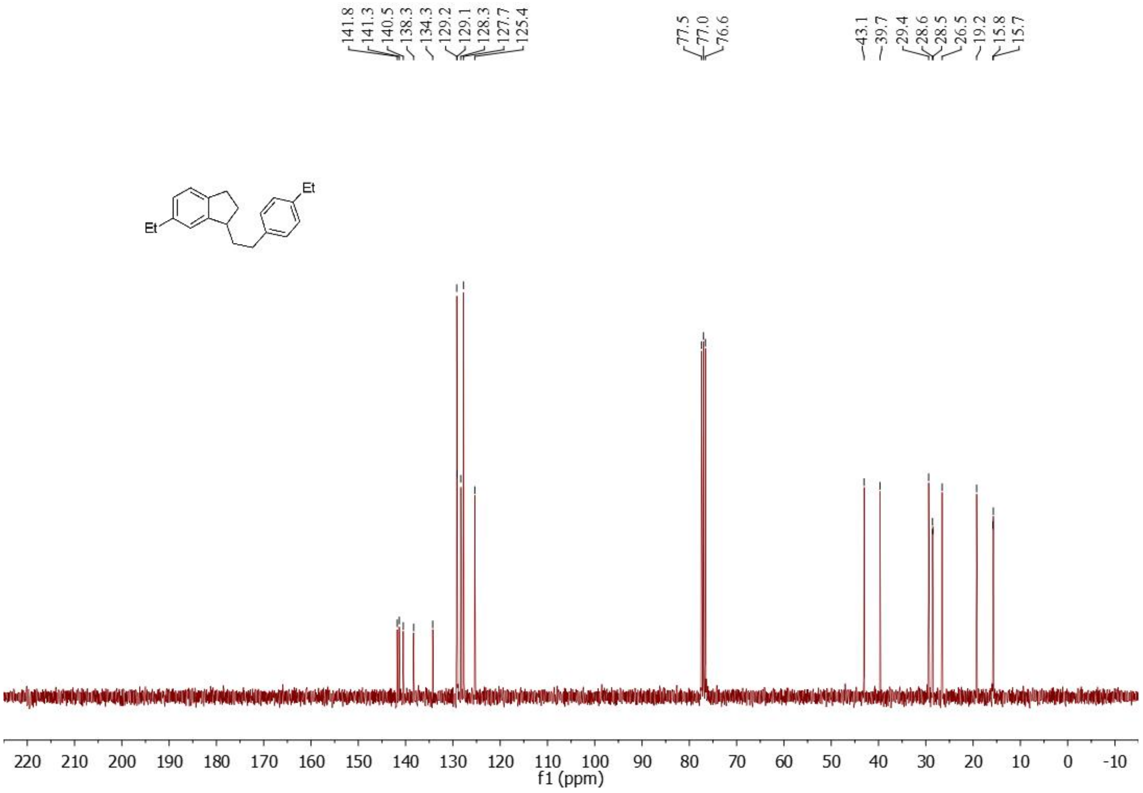

Figure S97. ^{1}H NMR spectrum of **5e**, related to **Figure 3**

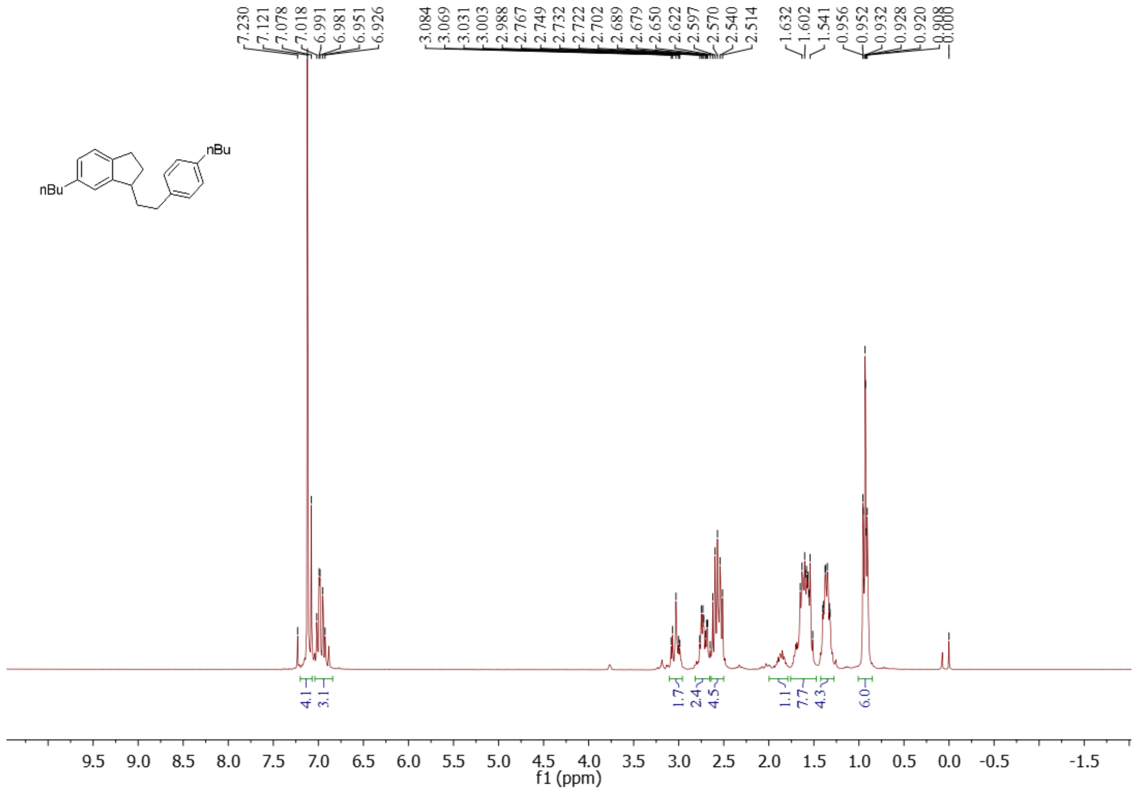

Figure S98. ^{13}C NMR spectrum of **5e**, related to **Figure 3**

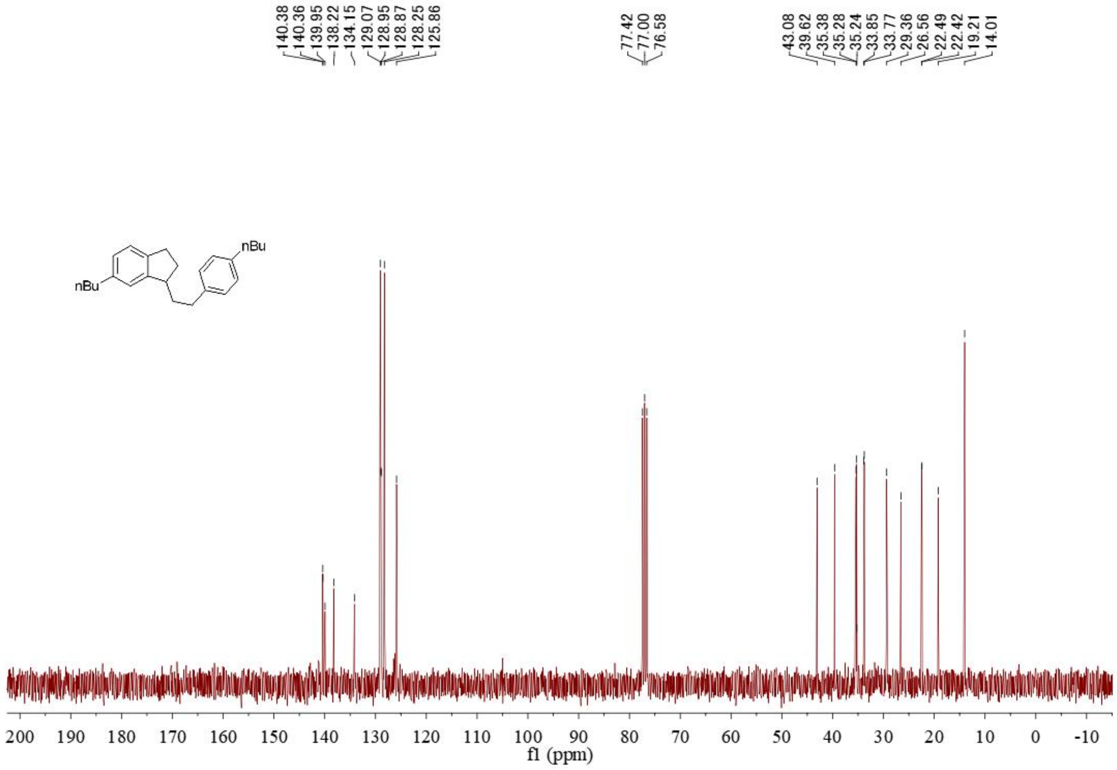

Figure S99. ^{1}H NMR spectrum of **5f**, related to **Figure 3**

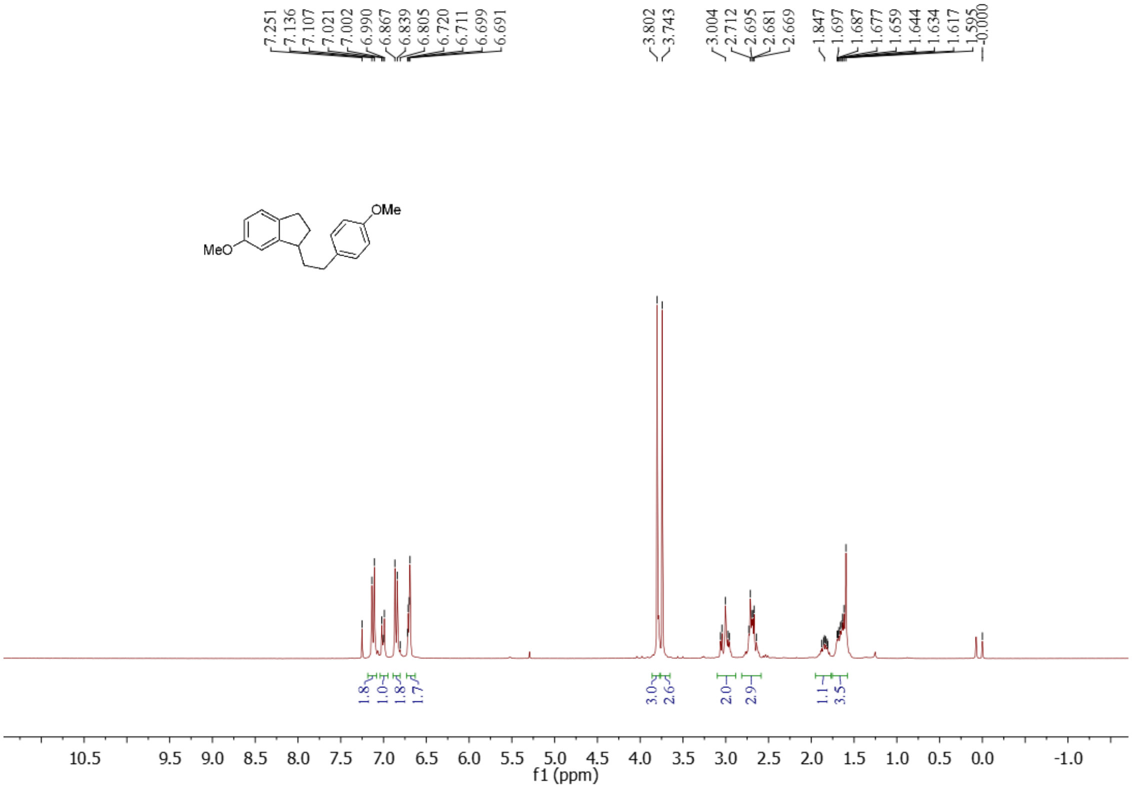

Figure S100. ^{13}C NMR spectrum of **5f**, related to **Figure 3**

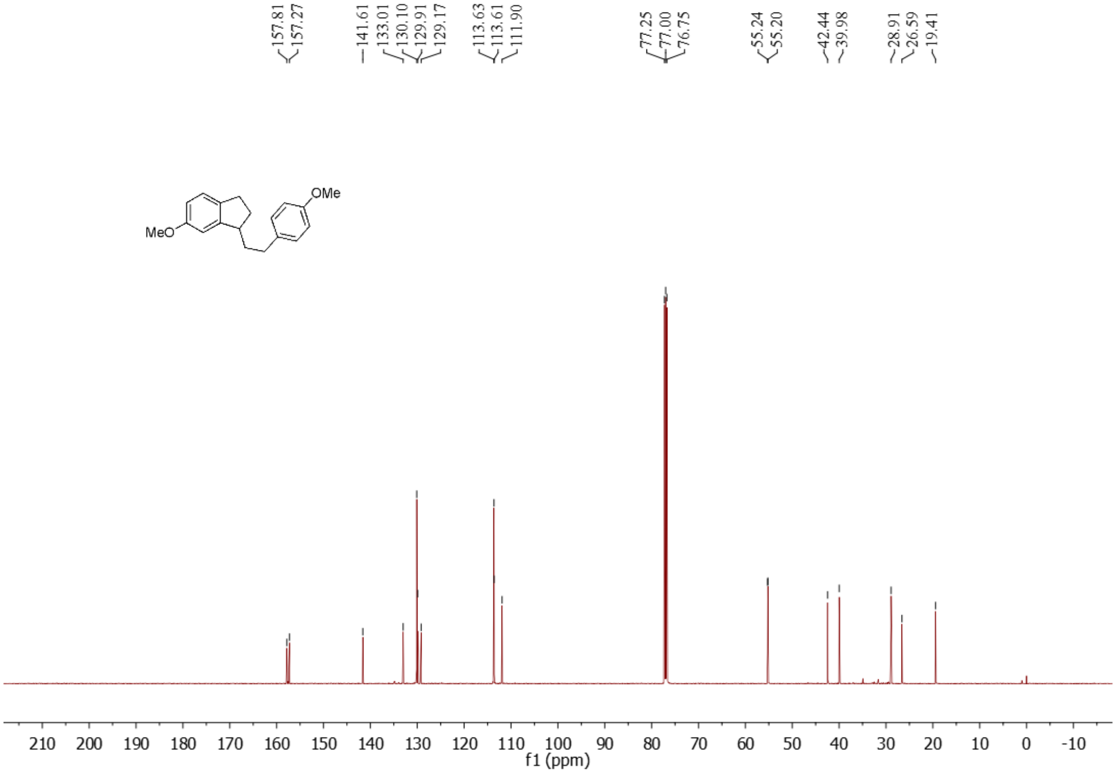

Figure S101. ^{1}H NMR spectrum of **5g**, related to **Figure 3**

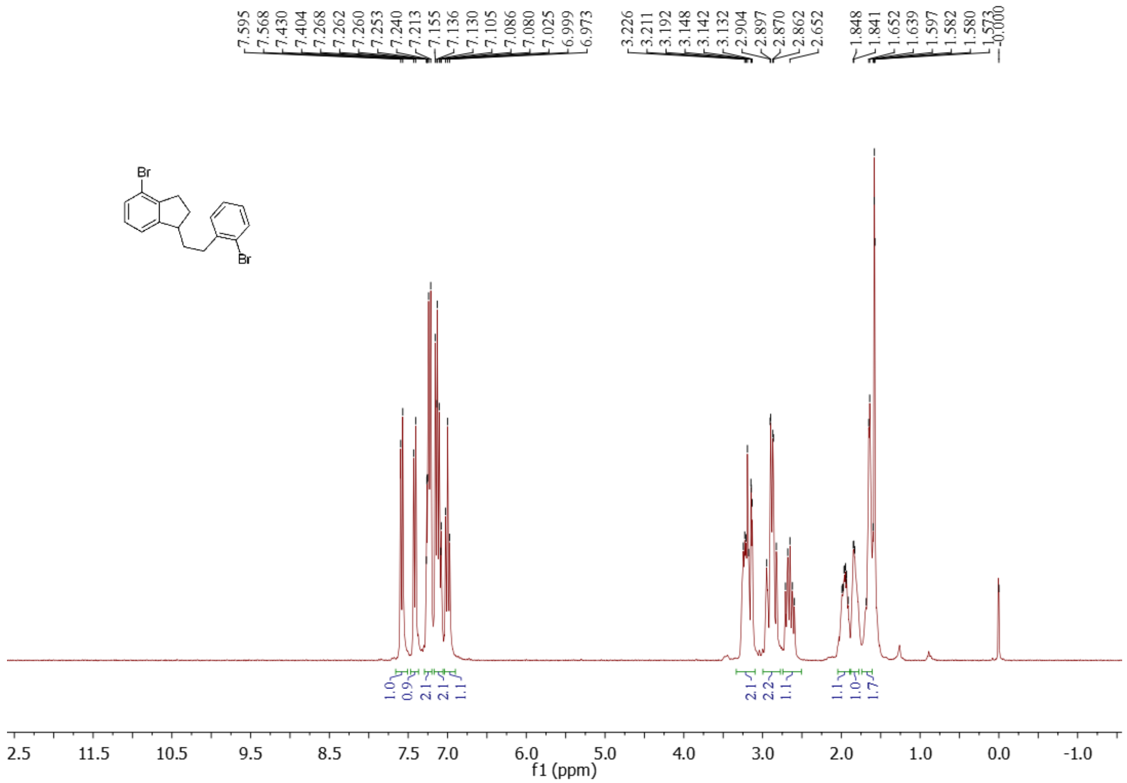

Figure S102. ^{13}C NMR spectrum of **5g**, related to **Figure 3**

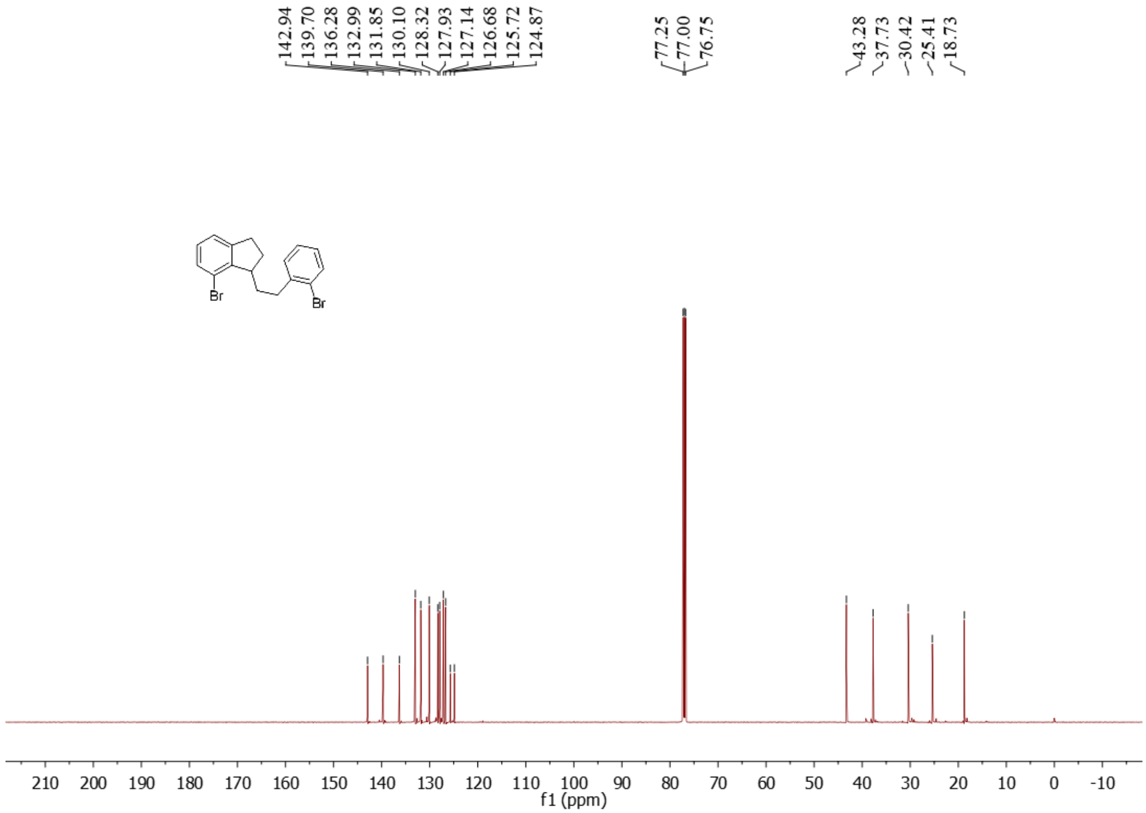

Figure S103. ^{1}H NMR spectrum of **5h**, related to **Figure 3**

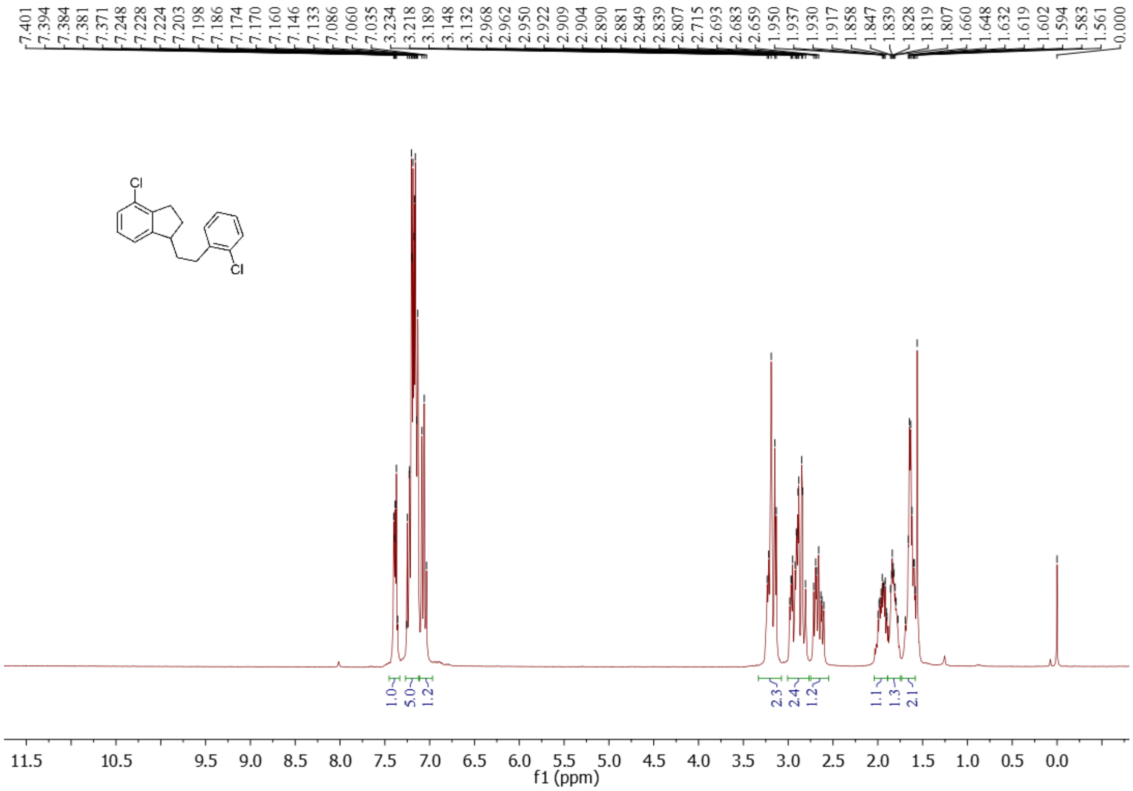

Figure S104. ^{13}C NMR spectrum of **5h**, related to **Figure 3**

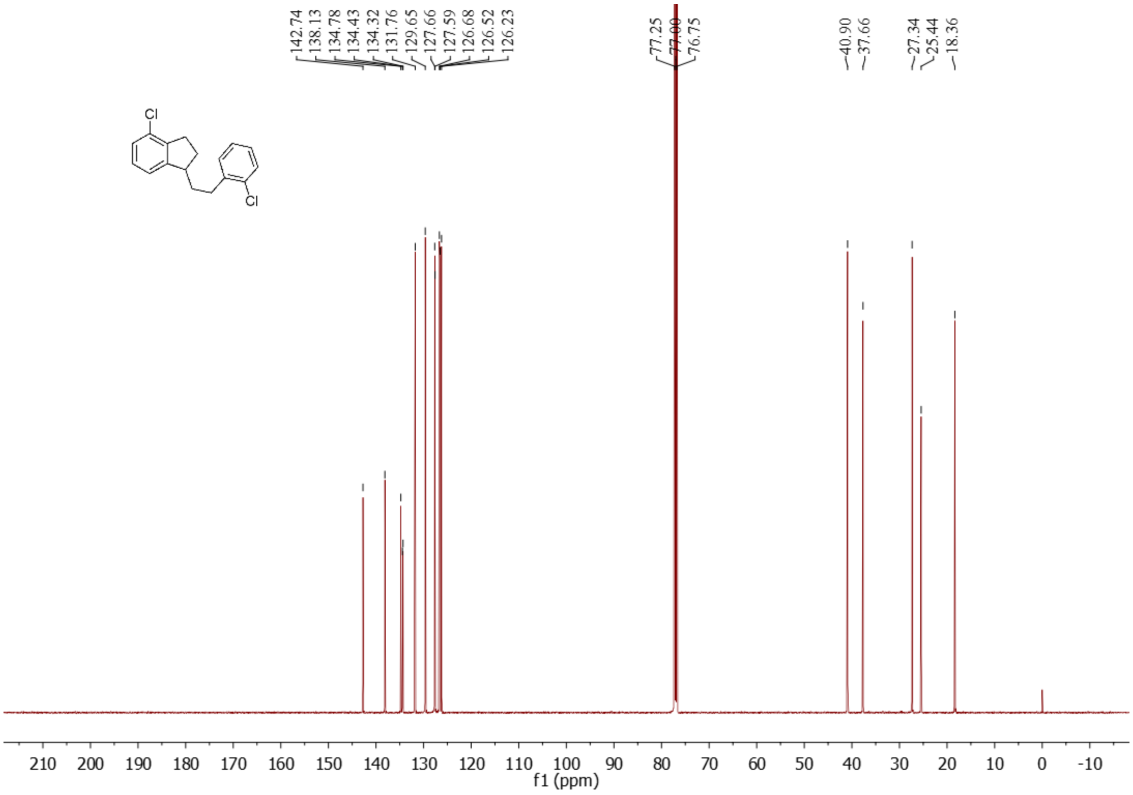

Figure S105. ^{1}H NMR spectrum of **5i**, related to **Figure 3**

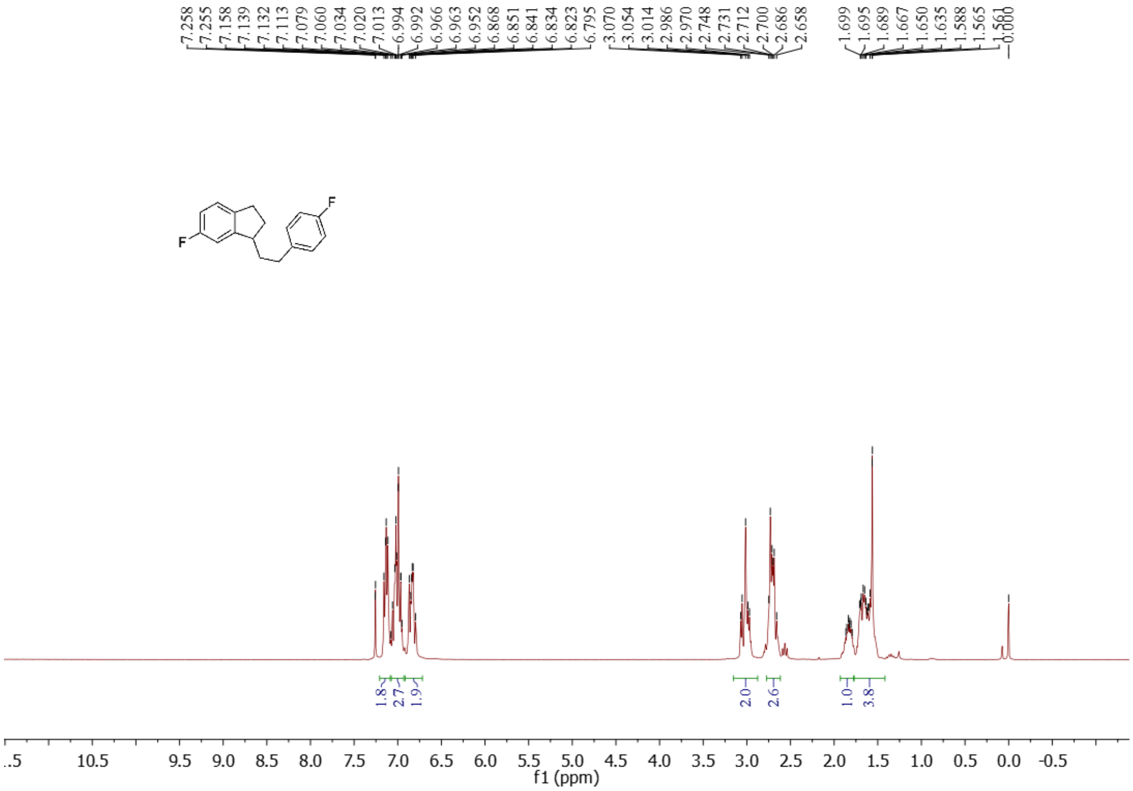

Figure S106. ^{13}C NMR spectrum of **5i**, related to **Figure 3**

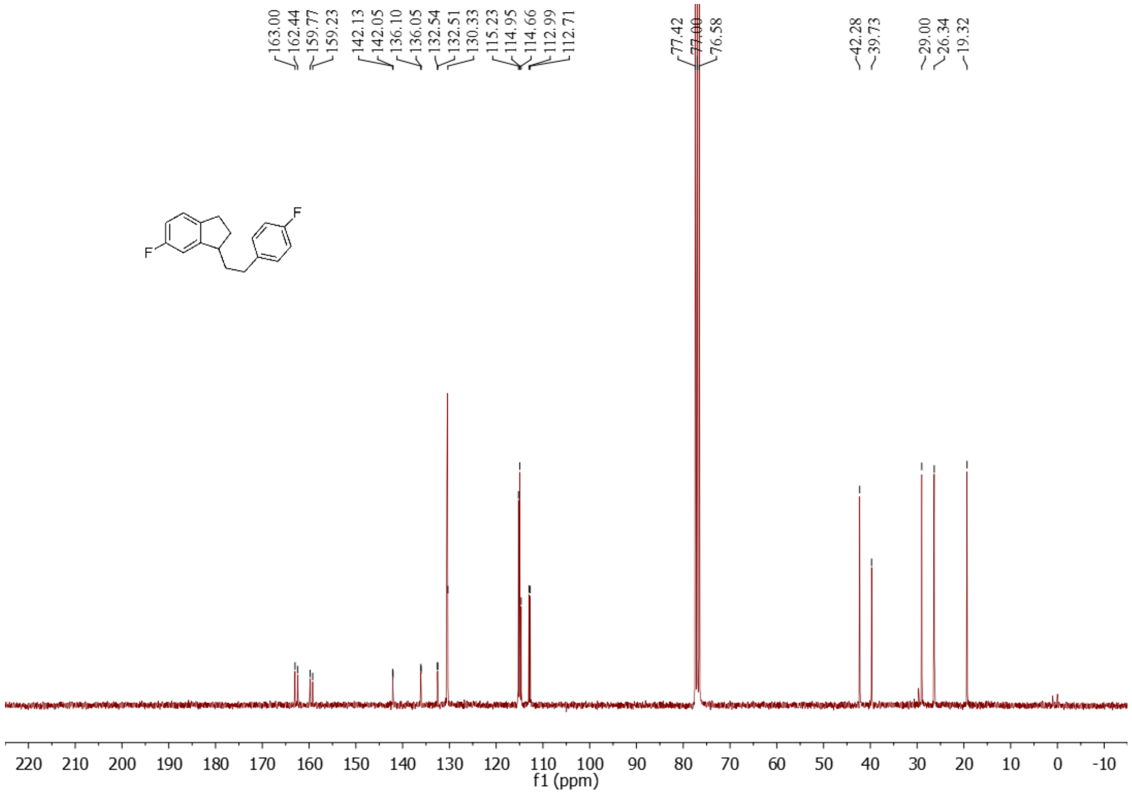

Figure S107. ^{1}H NMR spectrum of **5j**, related to **Figure 3**

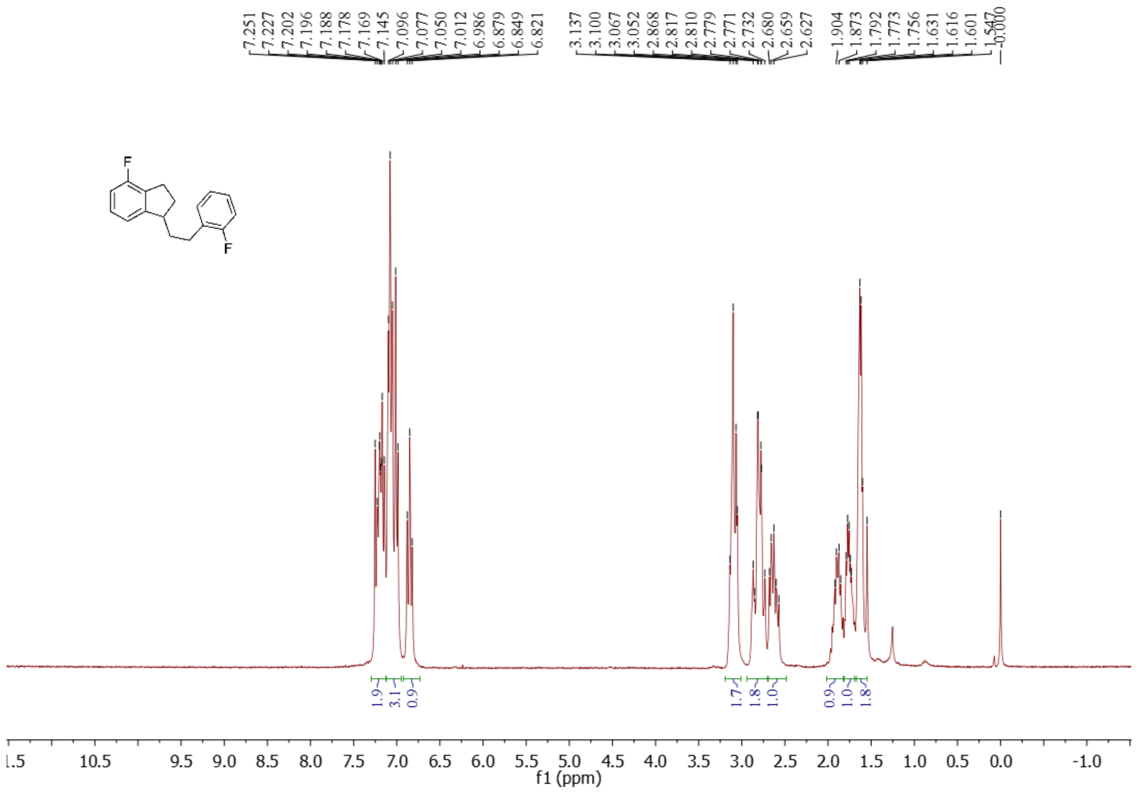

Figure S108. ^{13}C NMR spectrum of **5j**, related to **Figure 3**

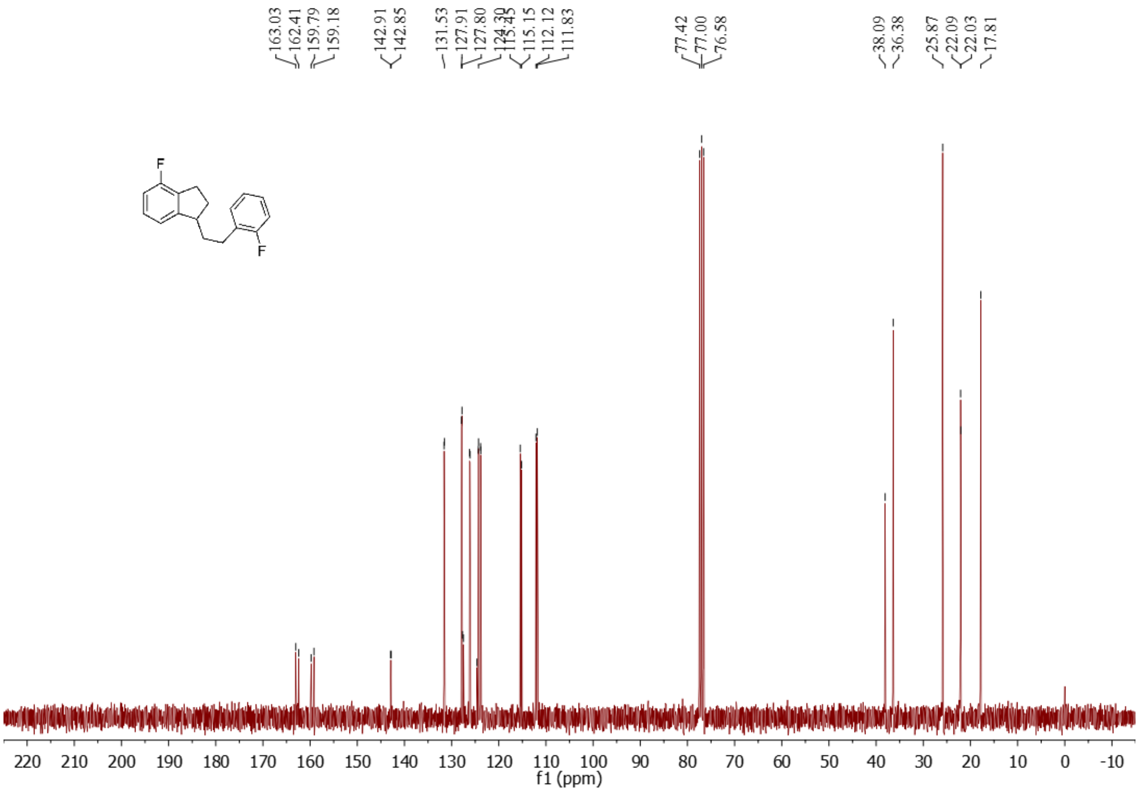

Figure S109. ^{1}H NMR spectrum of **5k**, related to **Figure 3**

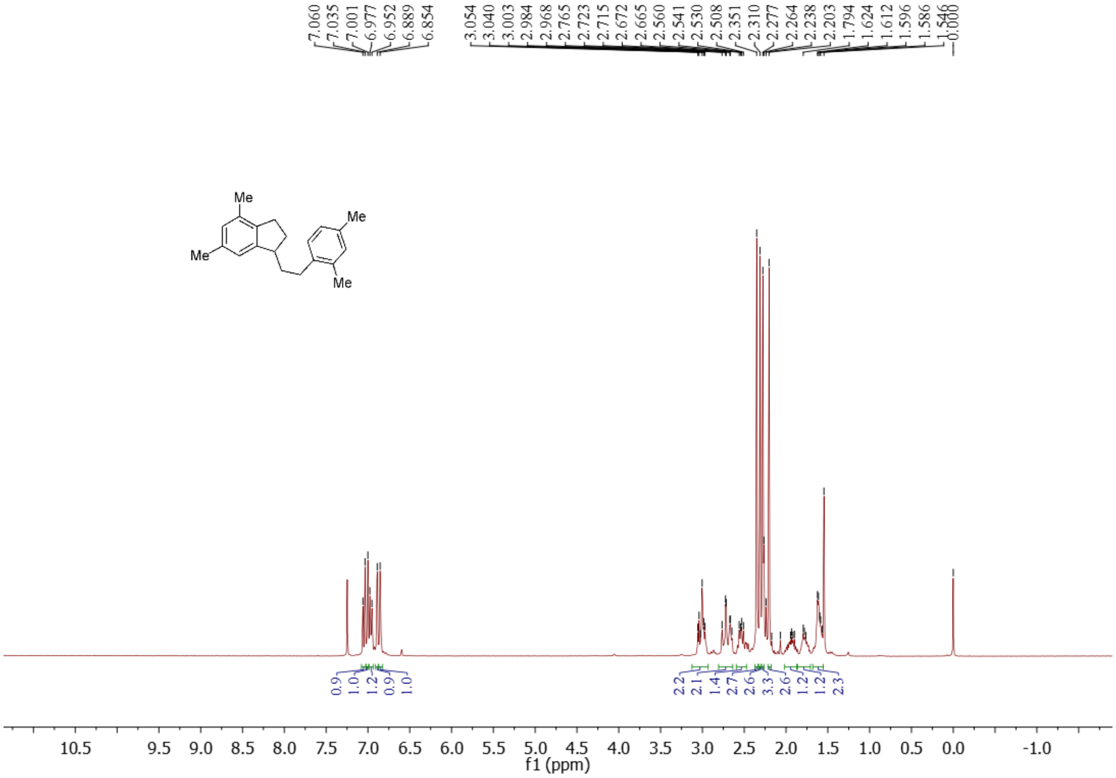

Figure S110. ^{13}C NMR spectrum of **5k**, related to **Figure 3**

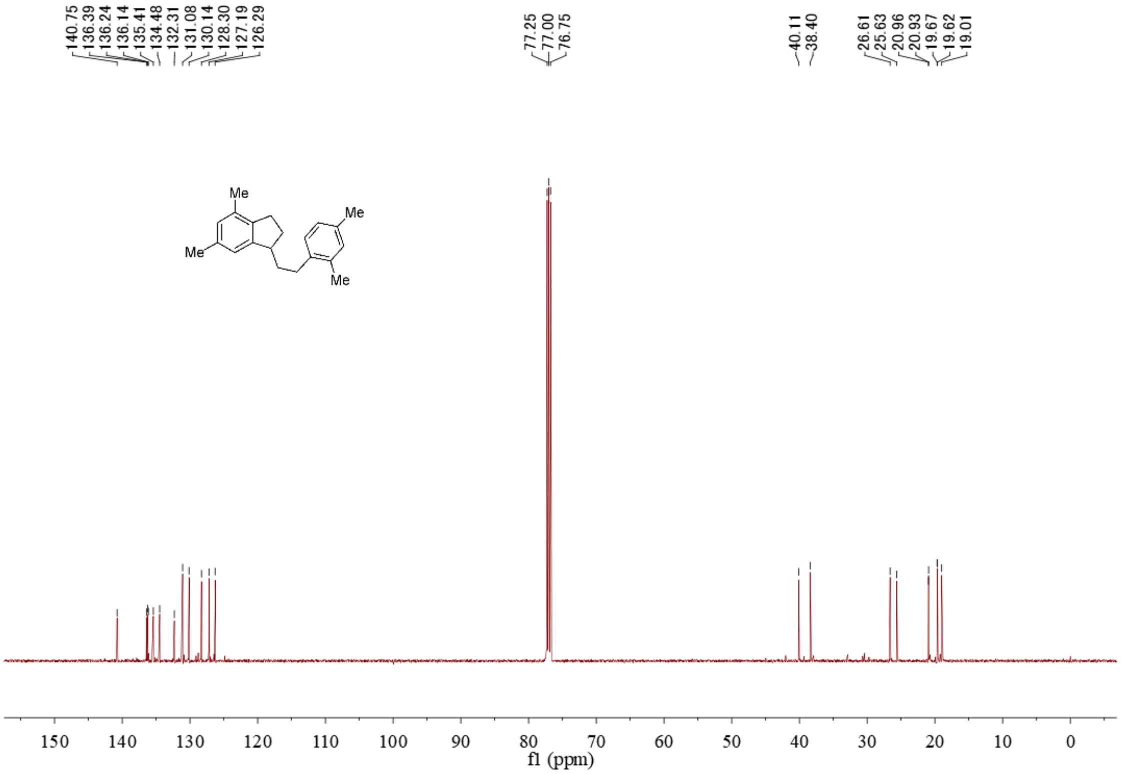

Figure S111. ^{1}H NMR spectrum of **5l**, related to **Figure 3**

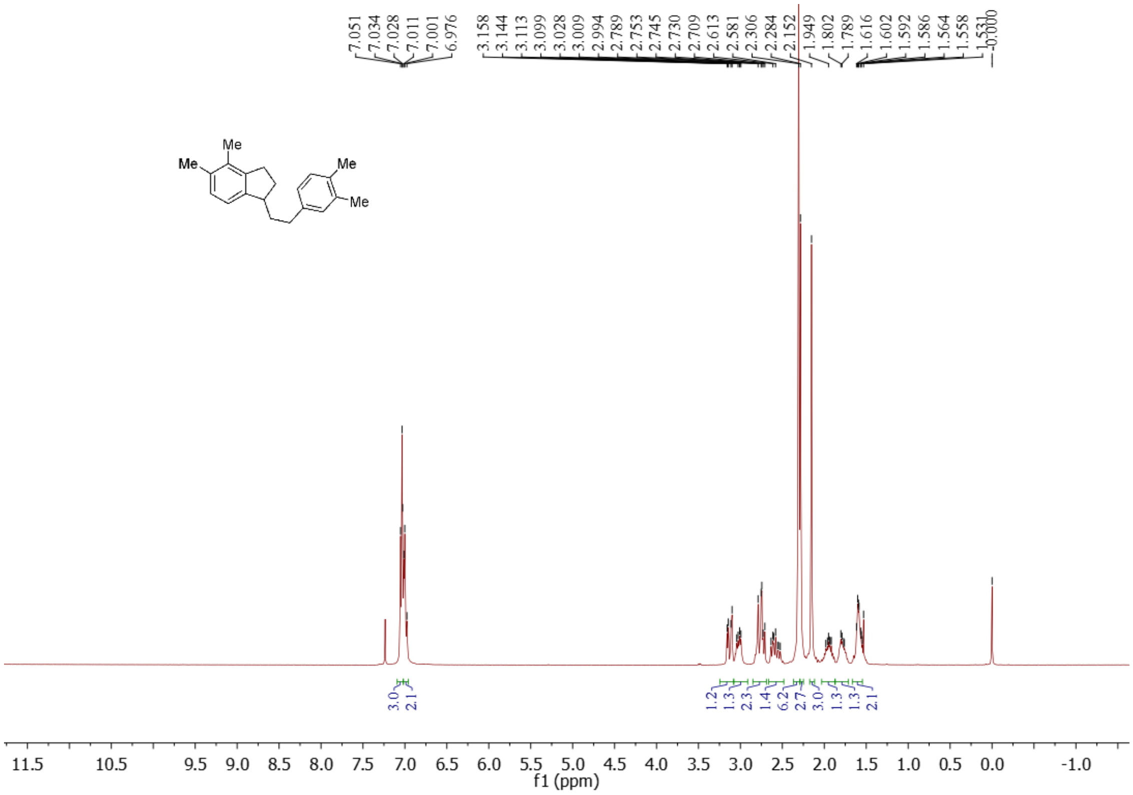

Figure S112. ^{13}C NMR spectrum of **5l**, related to **Figure 3**

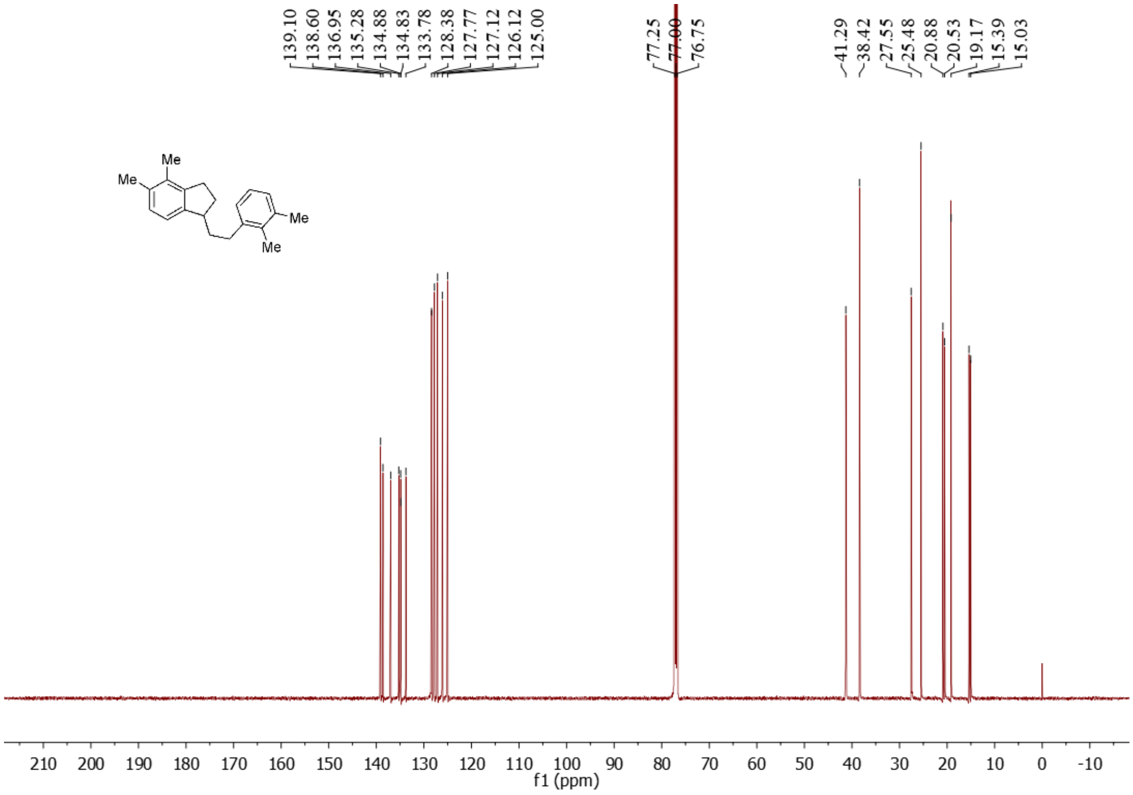

Figure S113. ^{1}H NMR spectrum of **5m**, related to **Figure 3**

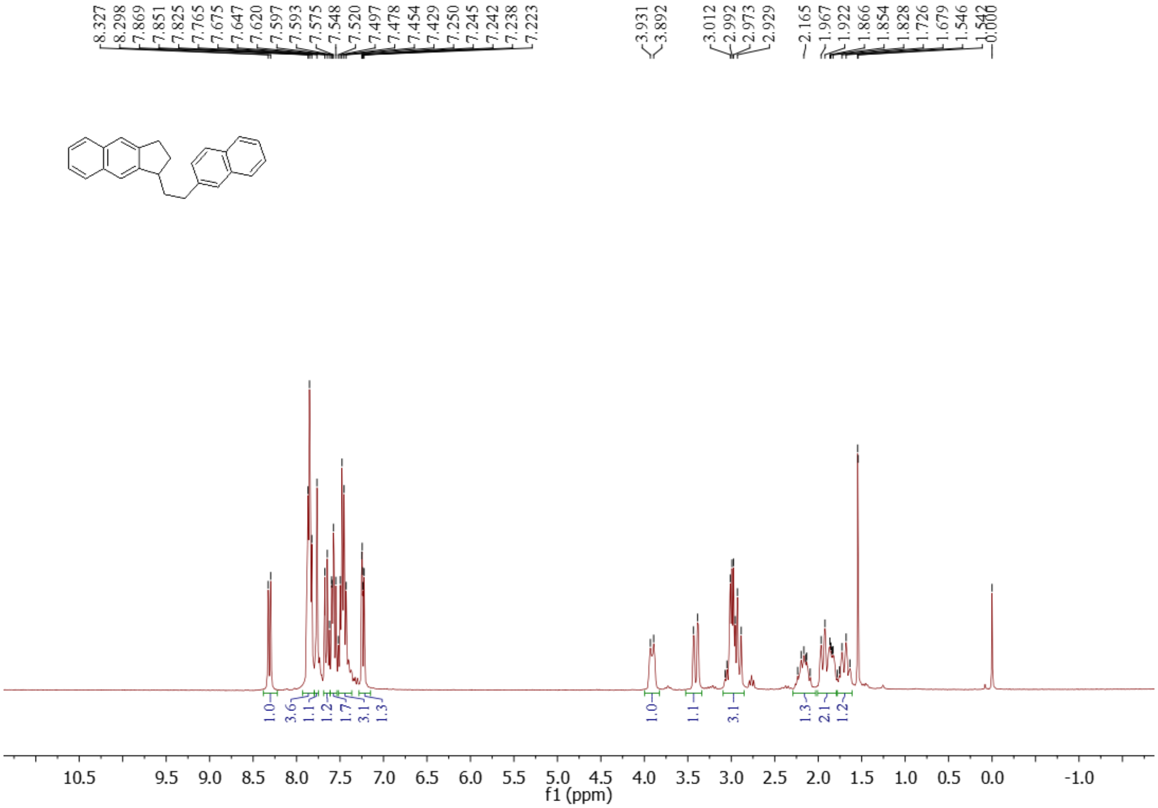

Figure S114. ^{13}C NMR spectrum of **5m**, related to **Figure 3**

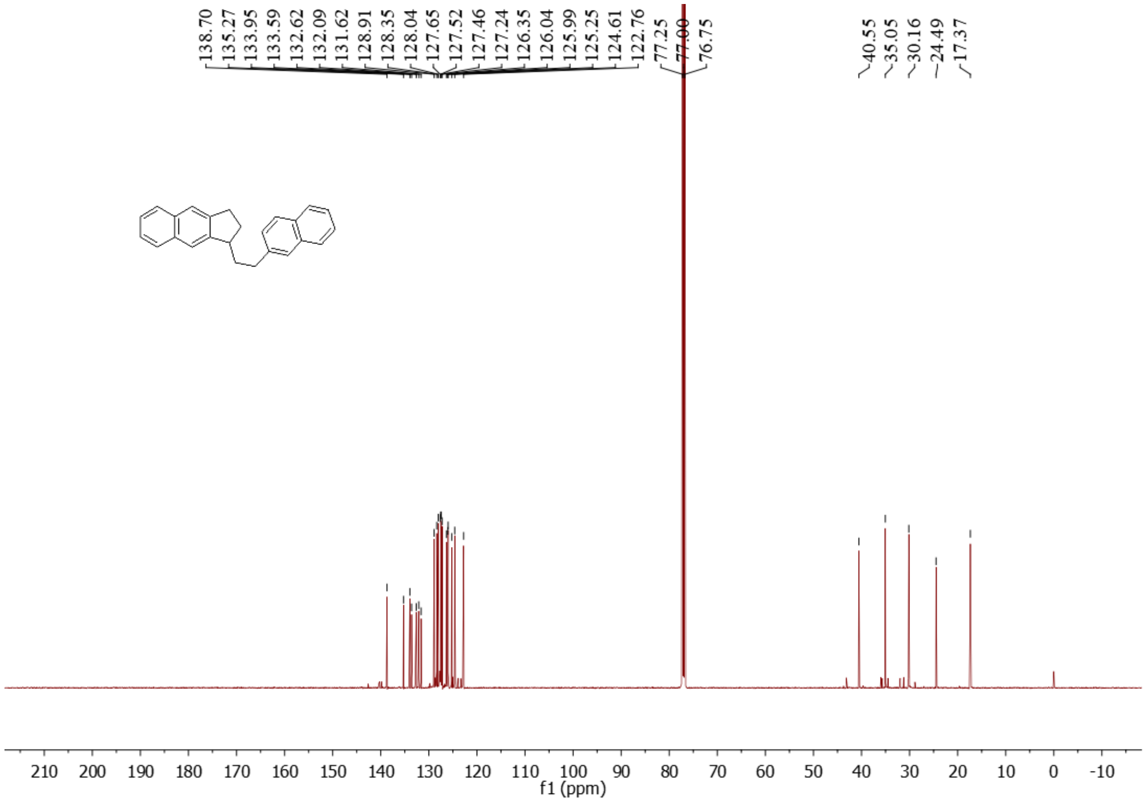

Figure S115. ^{1}H NMR spectrum of **5n**, related to **Figure 3**

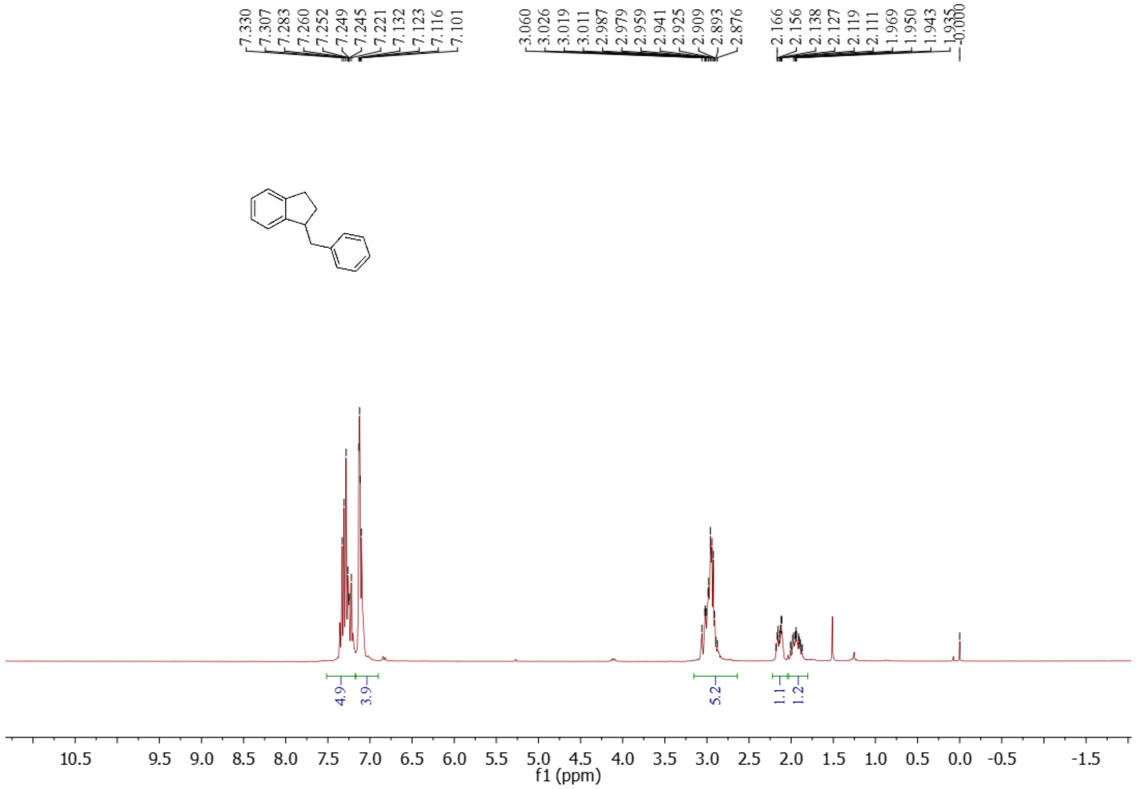

Figure S116. ^{13}C NMR spectrum of **5n**, related to **Figure 3**

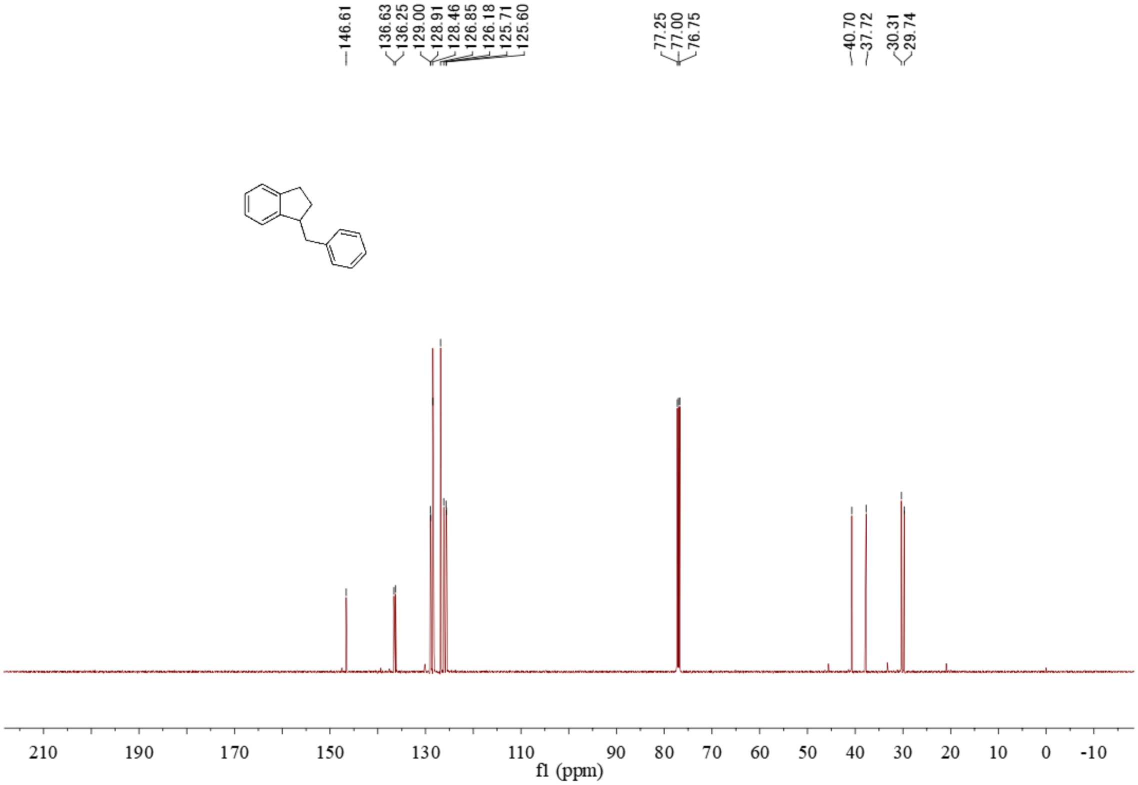

Figure S117. ^{1}H NMR spectrum of **5o**, related to **Figure 3**

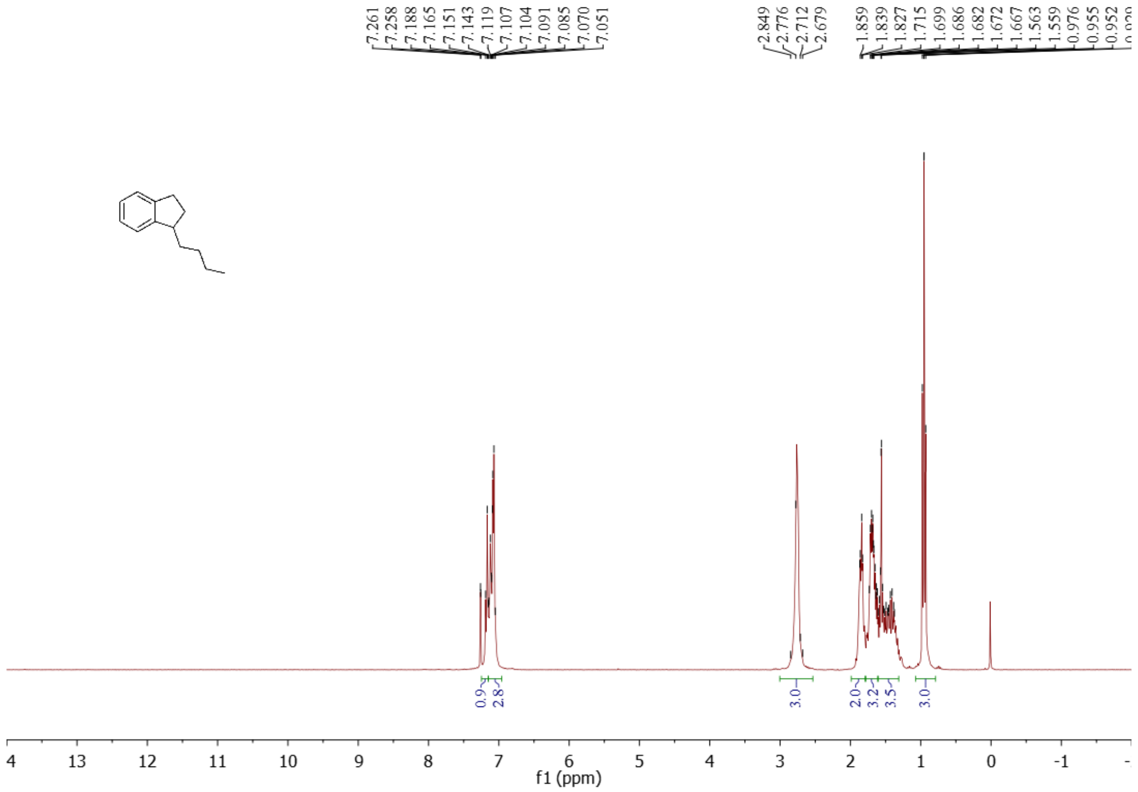

Figure S118. ^{1}H NMR spectrum of **5p**, related to **Figure 3**

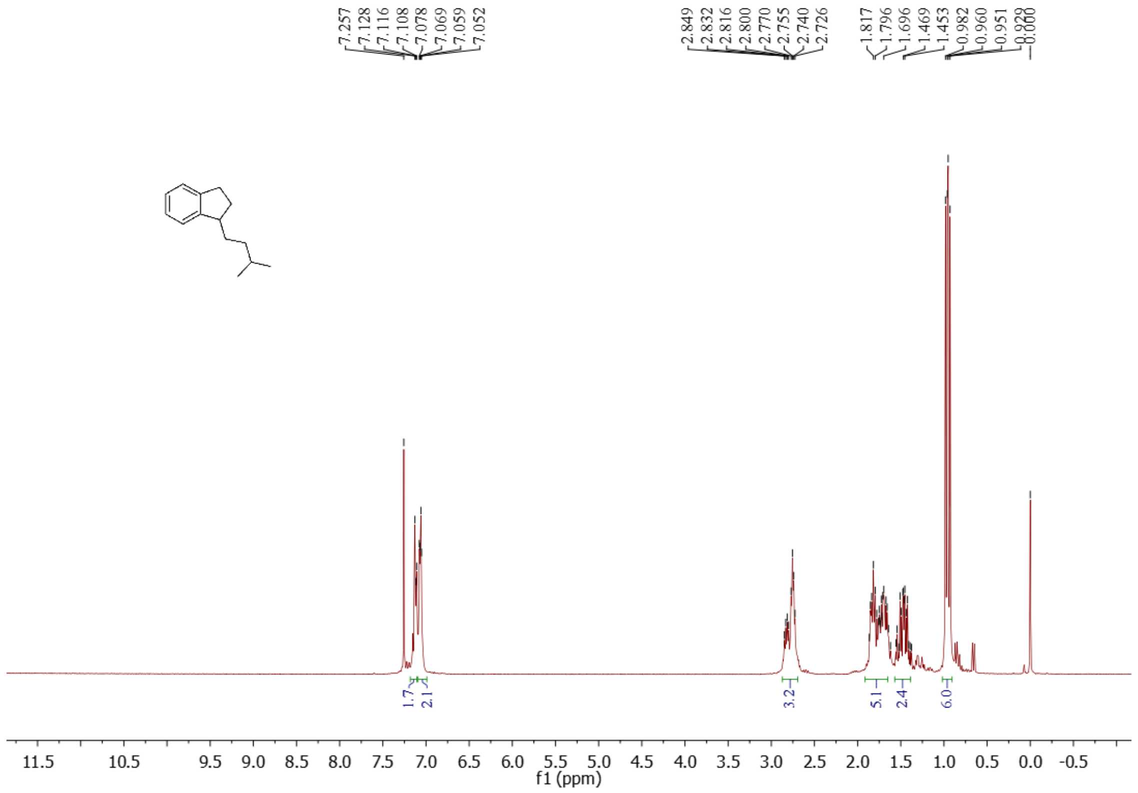

Figure S119. ^{13}C NMR spectrum of **5p**, related to **Figure 3**

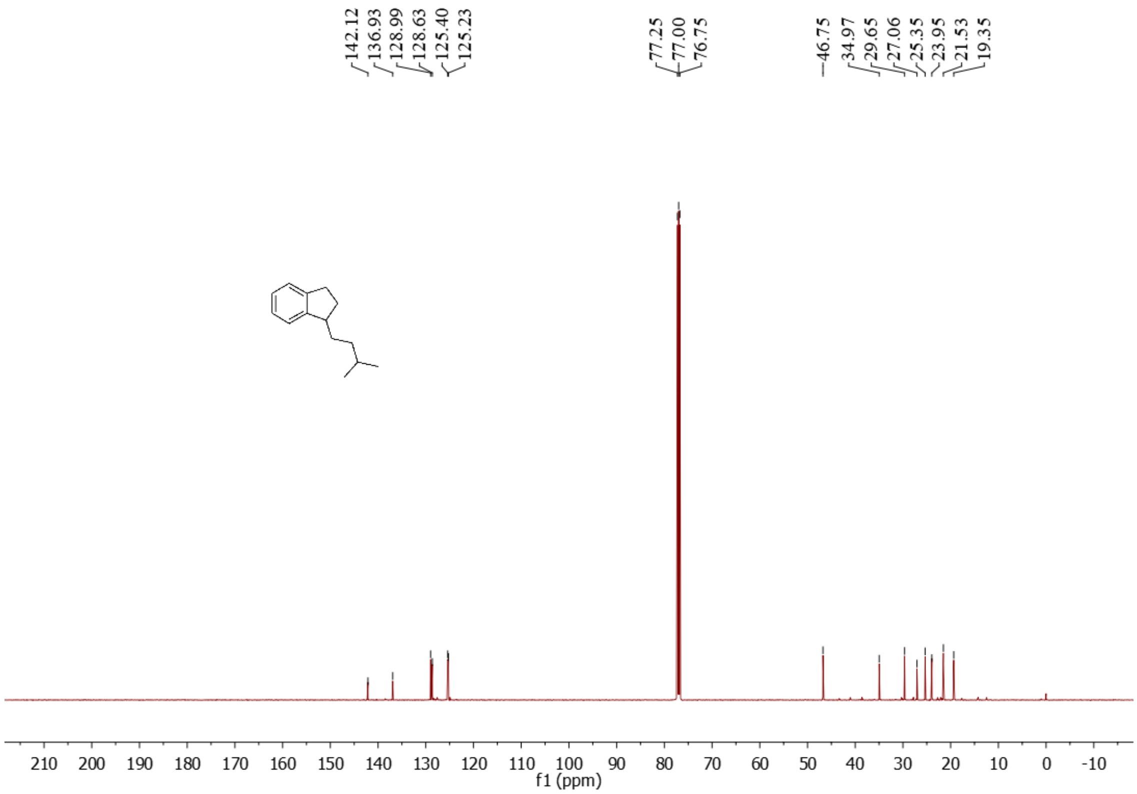

Figure S120. ^{1}H NMR spectrum of **5q**, related to **Figure 3**

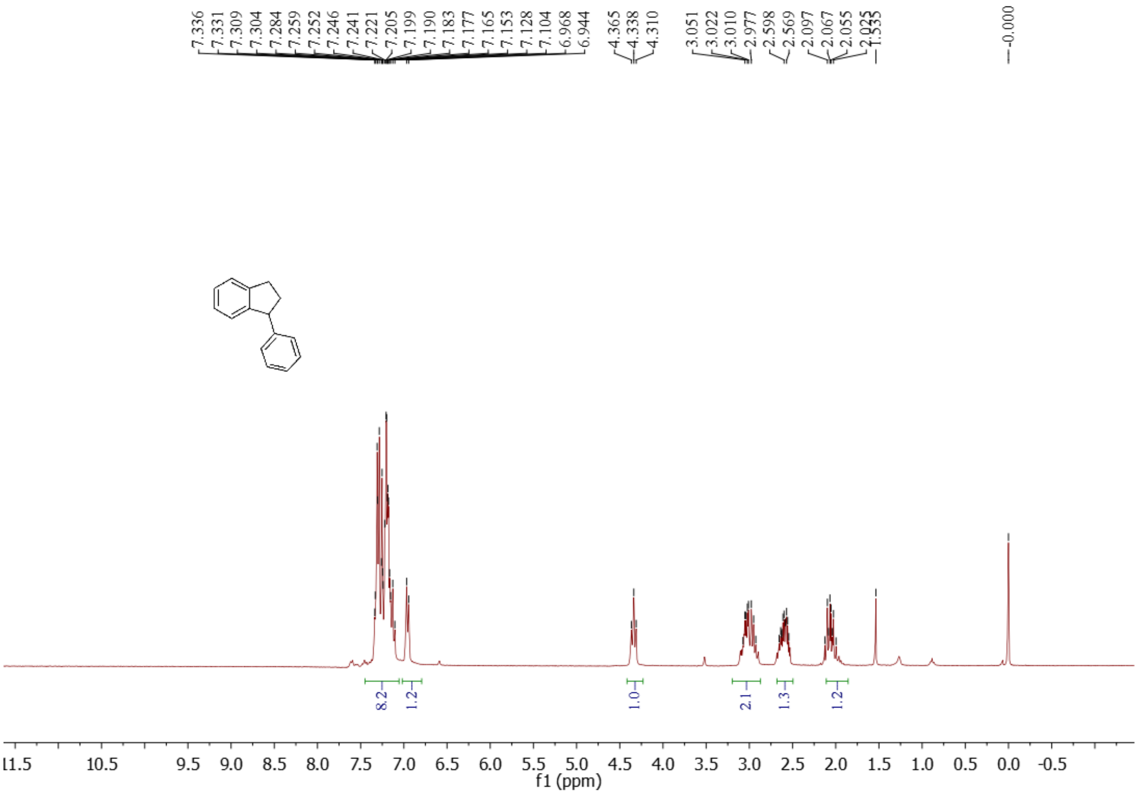

Figure S121. ^{1}H NMR spectrum of **5r**, related to **Figure 3**

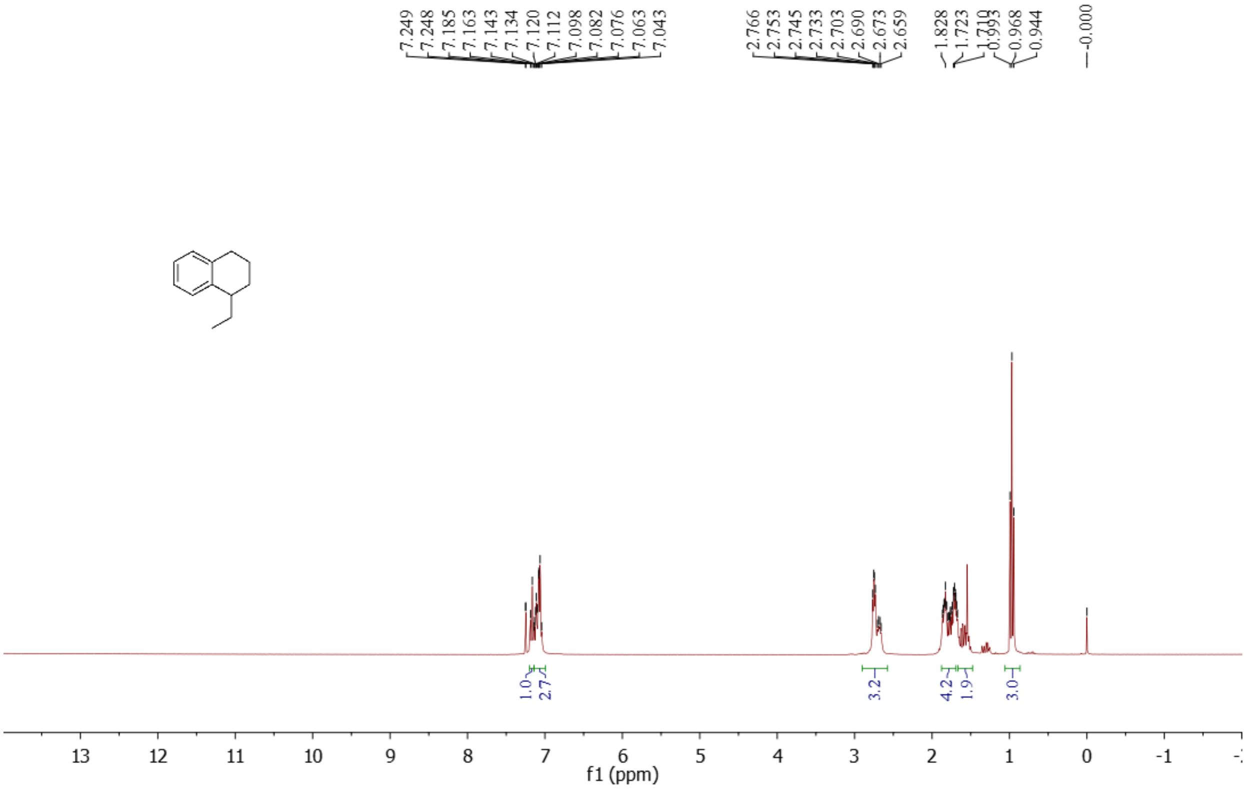

Figure S122. ^{1}H NMR spectrum of **5s**, related to **Figure 3**

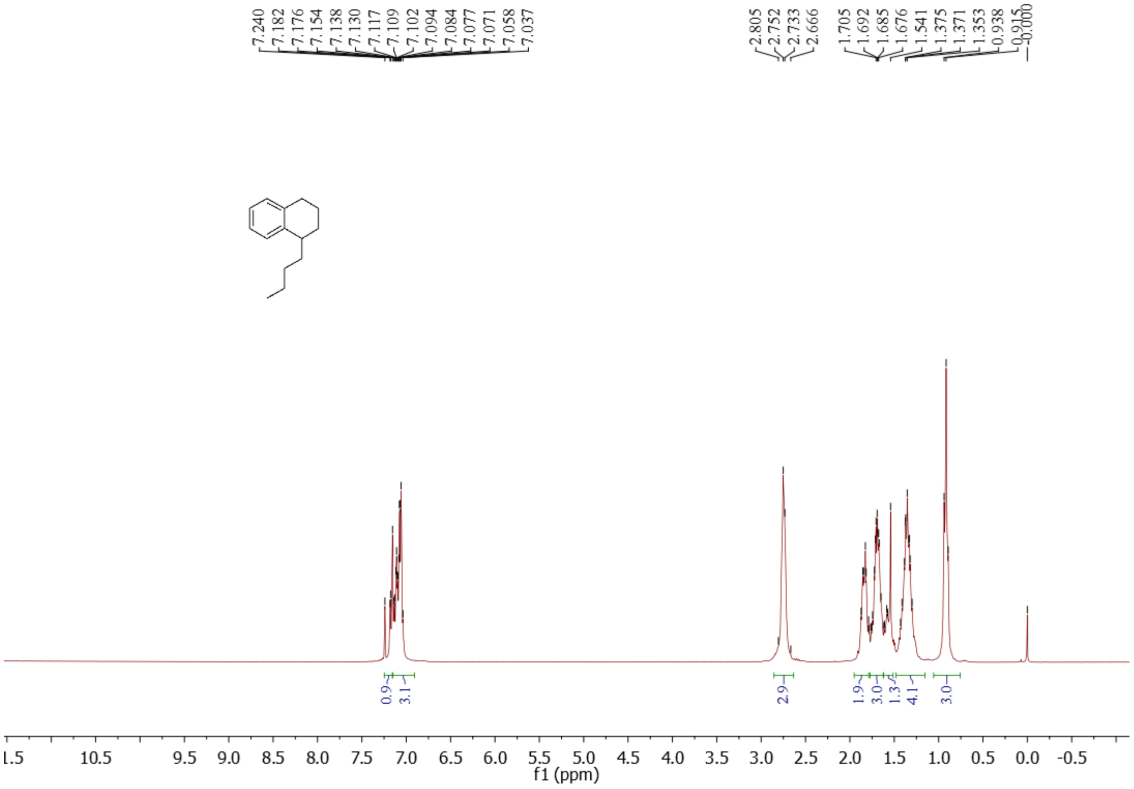

Figure S123. ^{1}H NMR spectrum of unknown *gem*-difluoride **1b**, related to **Figure 2**

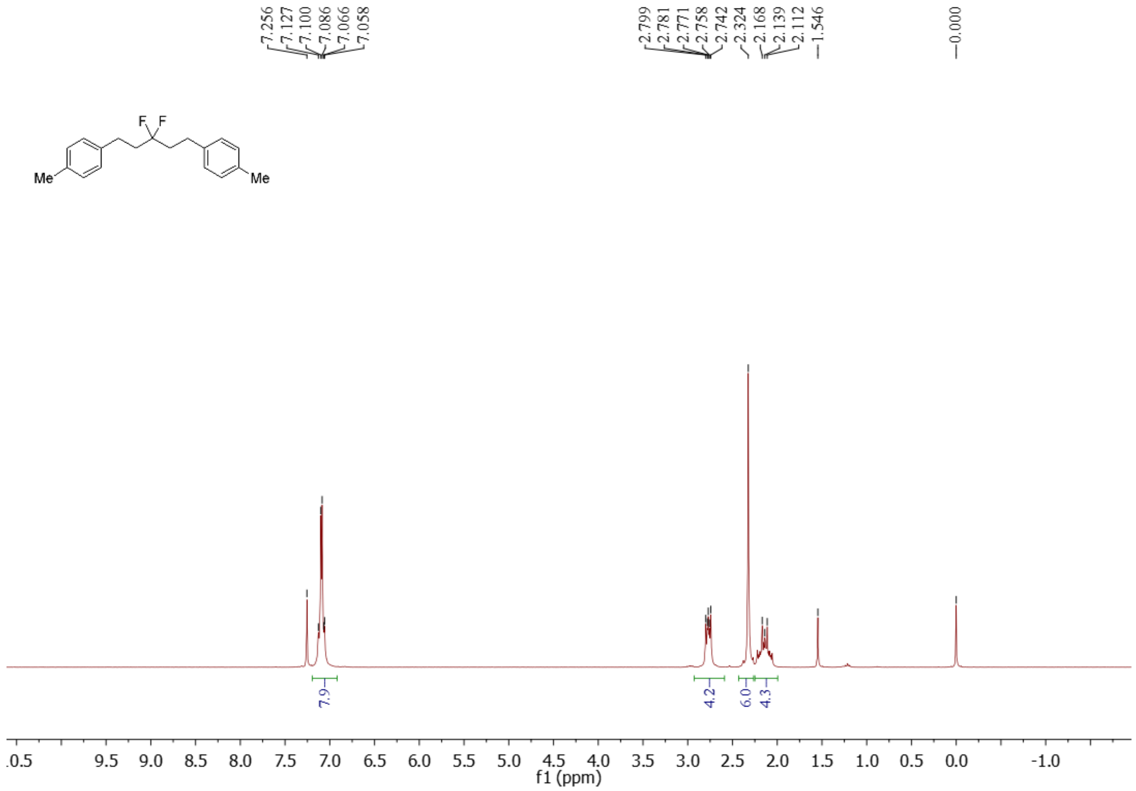

Figure S124. ^{13}C NMR spectrum of unknown *gem*-difluoride **1b**, related to **Figure 2**

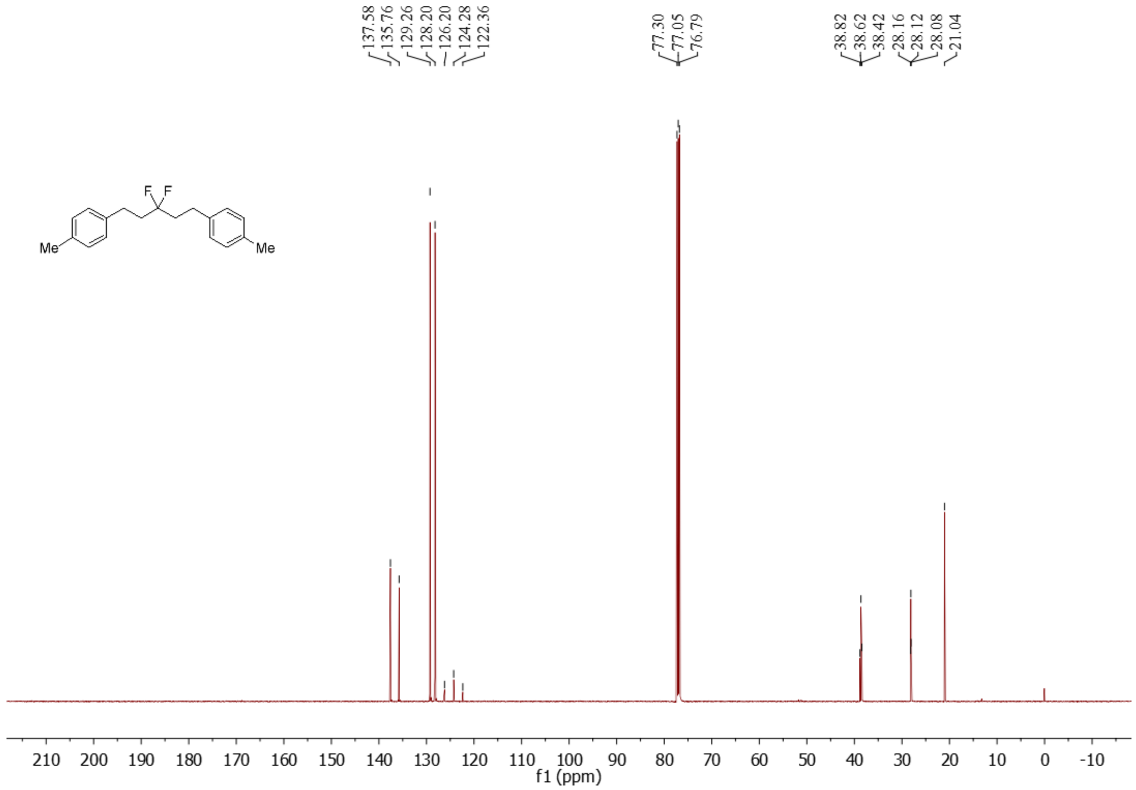

Figure S125. ^{19}F NMR spectrum of unknown *gem*-difluoride **1b**, related to **Figure 2**

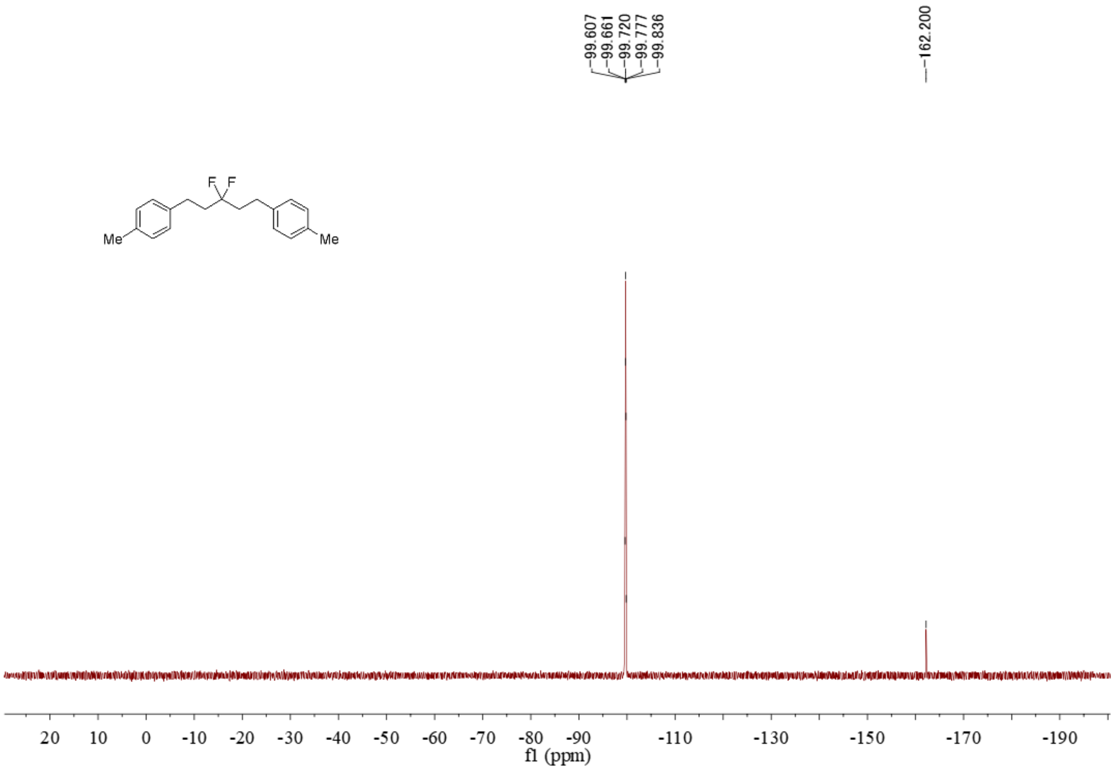

Figure S126. ^{1}H NMR spectrum of unknown *gem*-difluoride **1c**, related to **Figure 2**

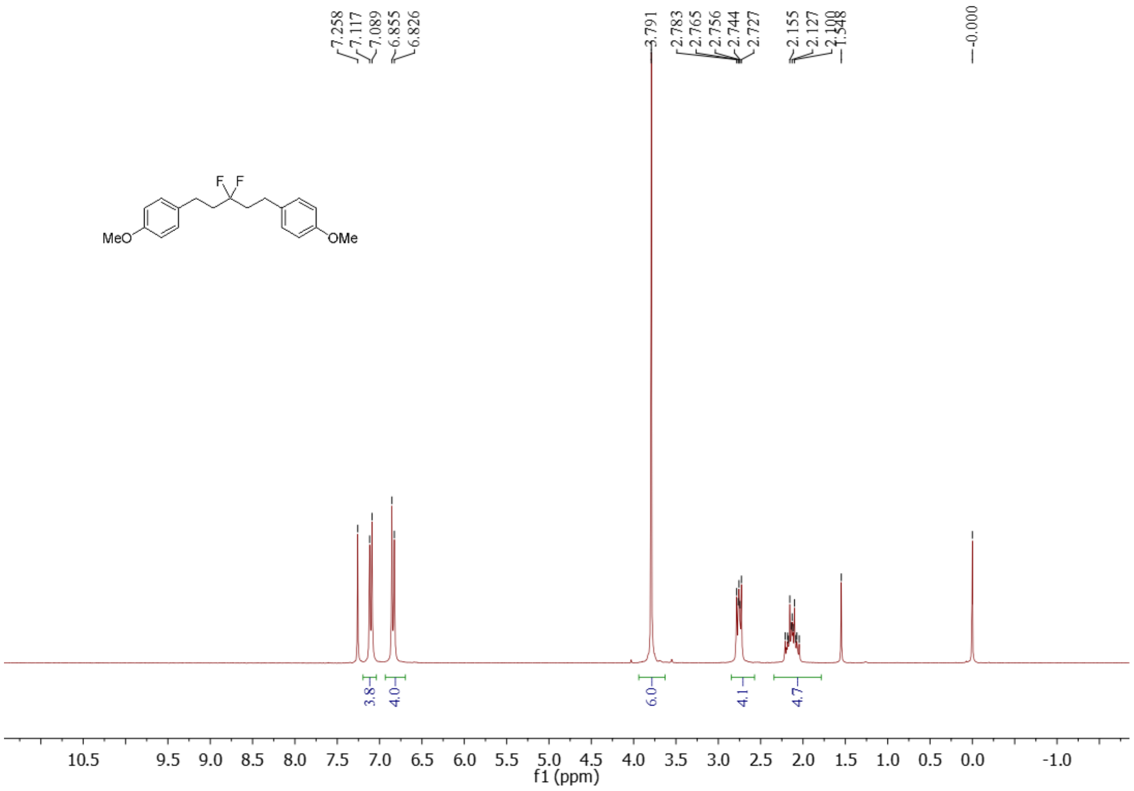

Figure S127. ^{13}C NMR spectrum of unknown *gem*-difluoride **1c**, related to **Figure 2**

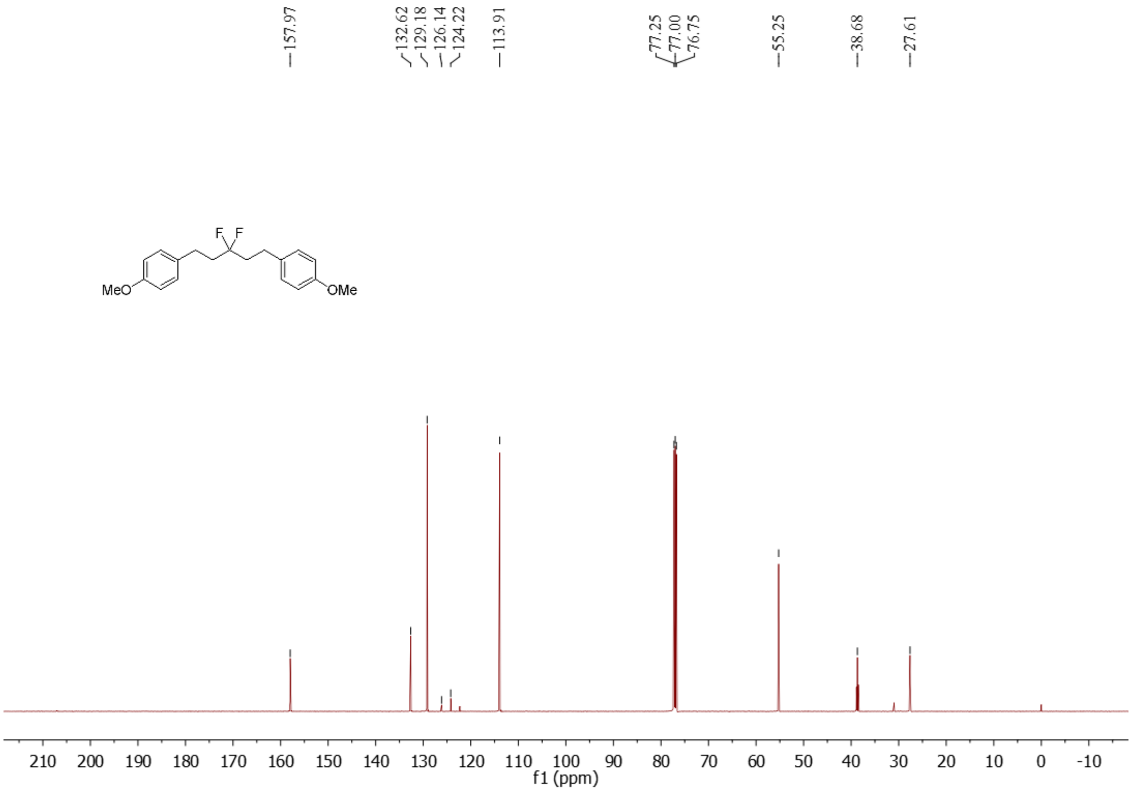

Figure S128. ^{19}F NMR spectrum of unknown *gem*-difluoride **1c**, related to **Figure 2**

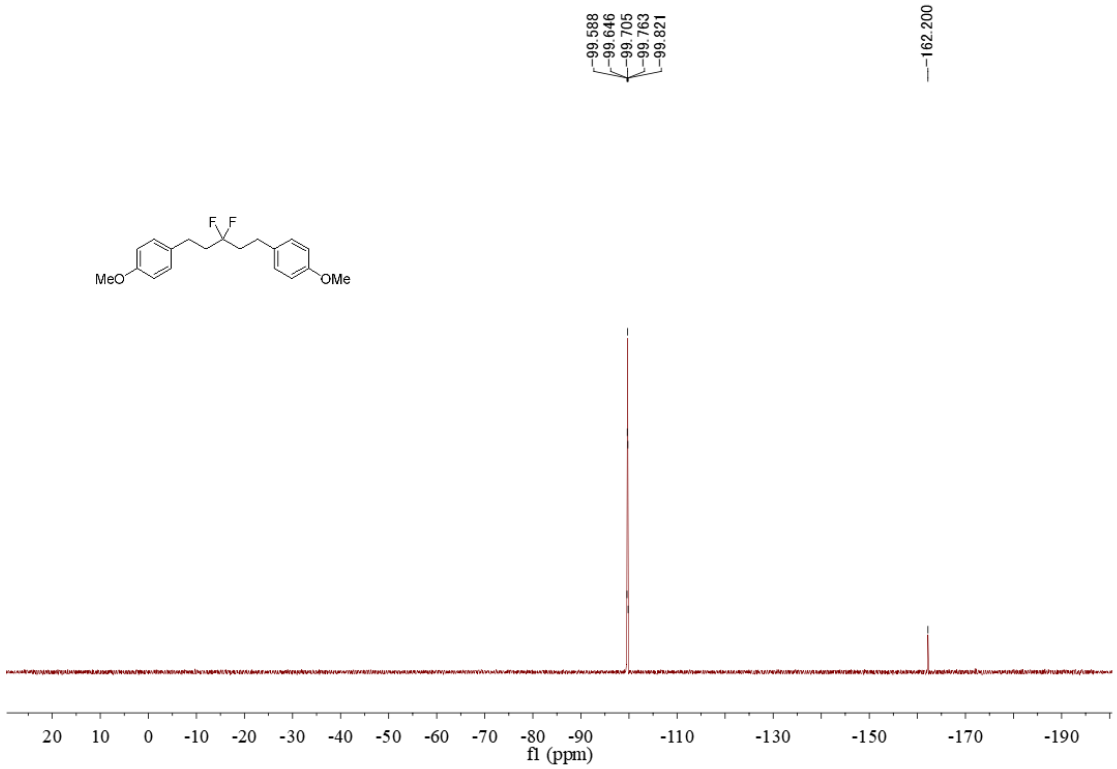

Figure S129. ^{1}H NMR spectrum of unknown *gem*-difluoride **1d**, related to **Figure 2**

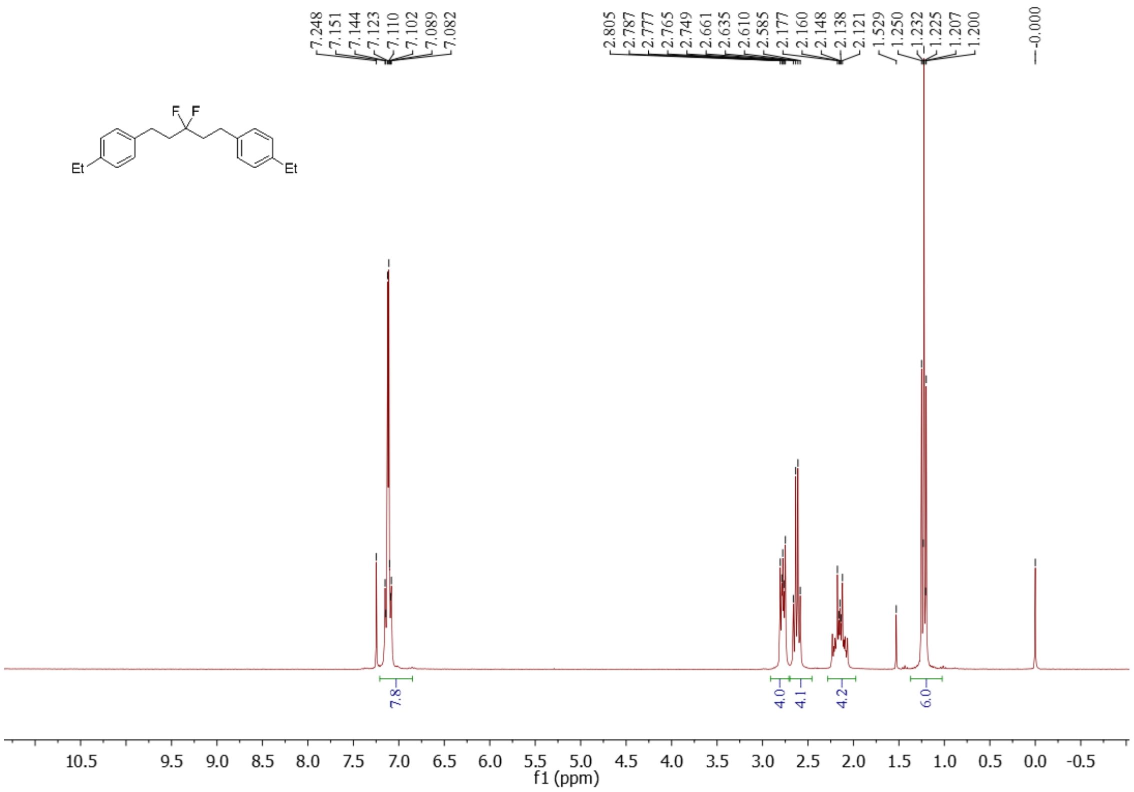

Figure S130. ^{13}C NMR spectrum of unknown *gem*-difluoride **1d**, related to **Figure 2**

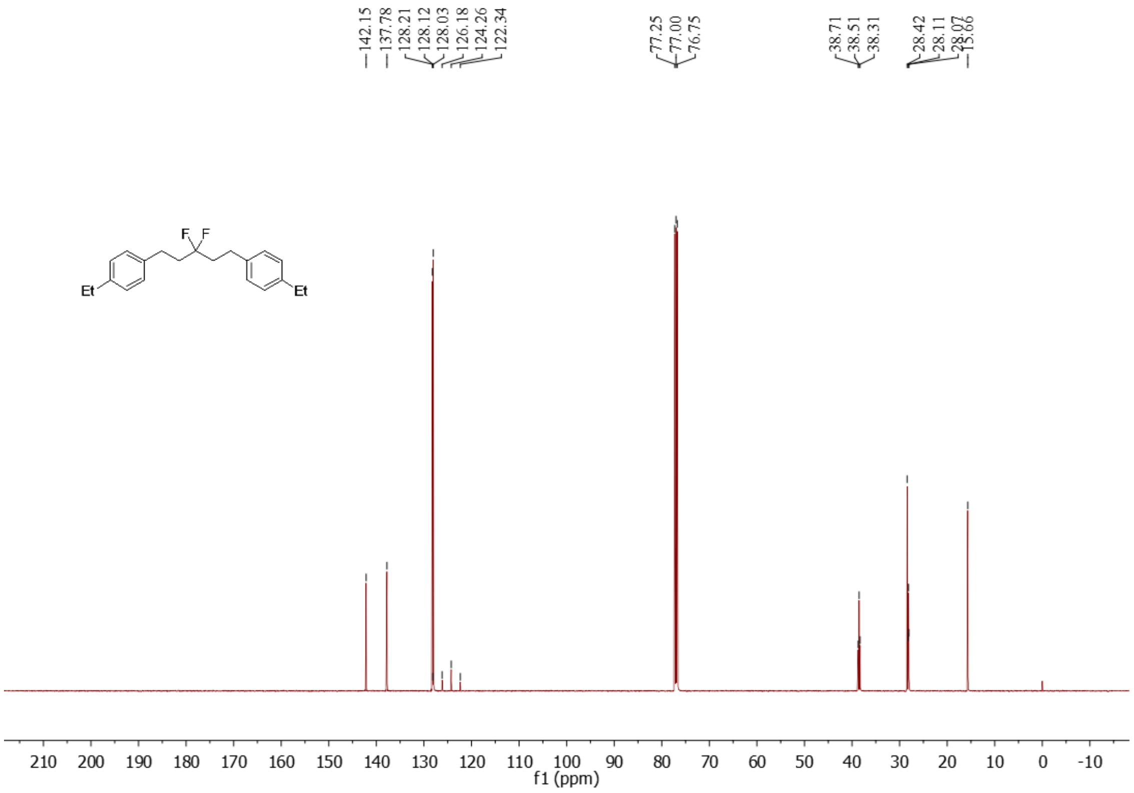

Figure S131. ^{19}F NMR spectrum of unknown *gem*-difluoride **1d**, related to **Figure 2**

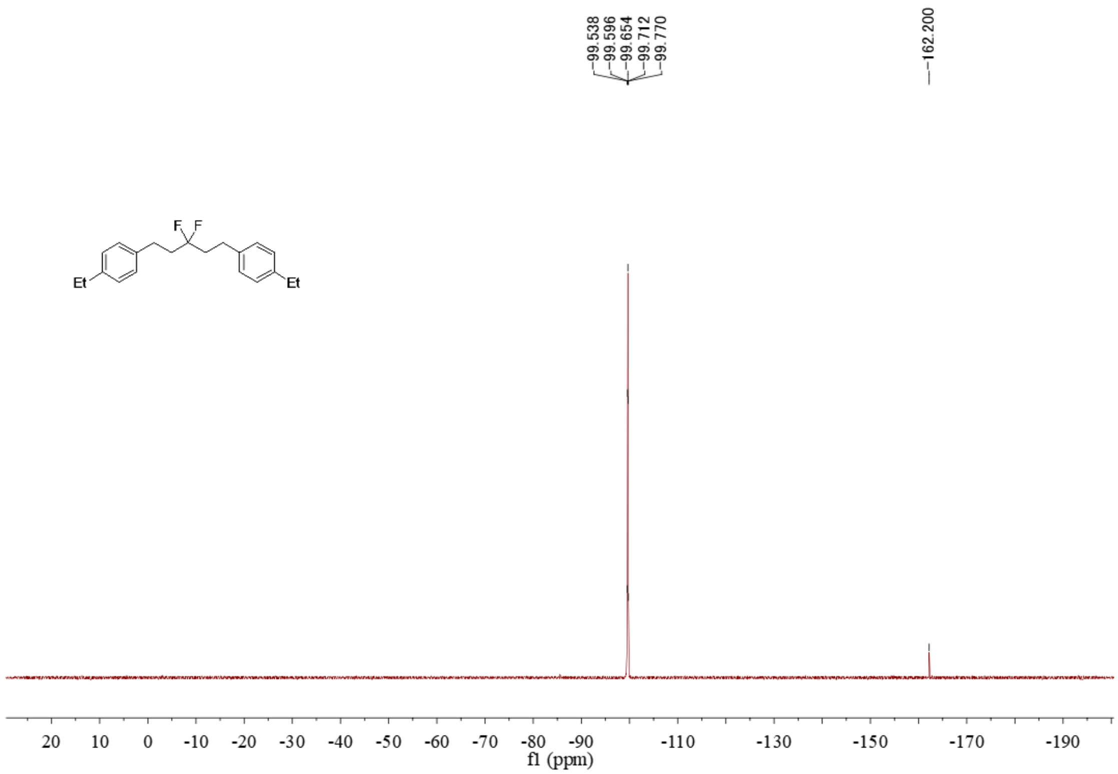

Figure S132. ^{1}H NMR spectrum of unknown *gem*-difluoride **1e**, related to **Figure 2**

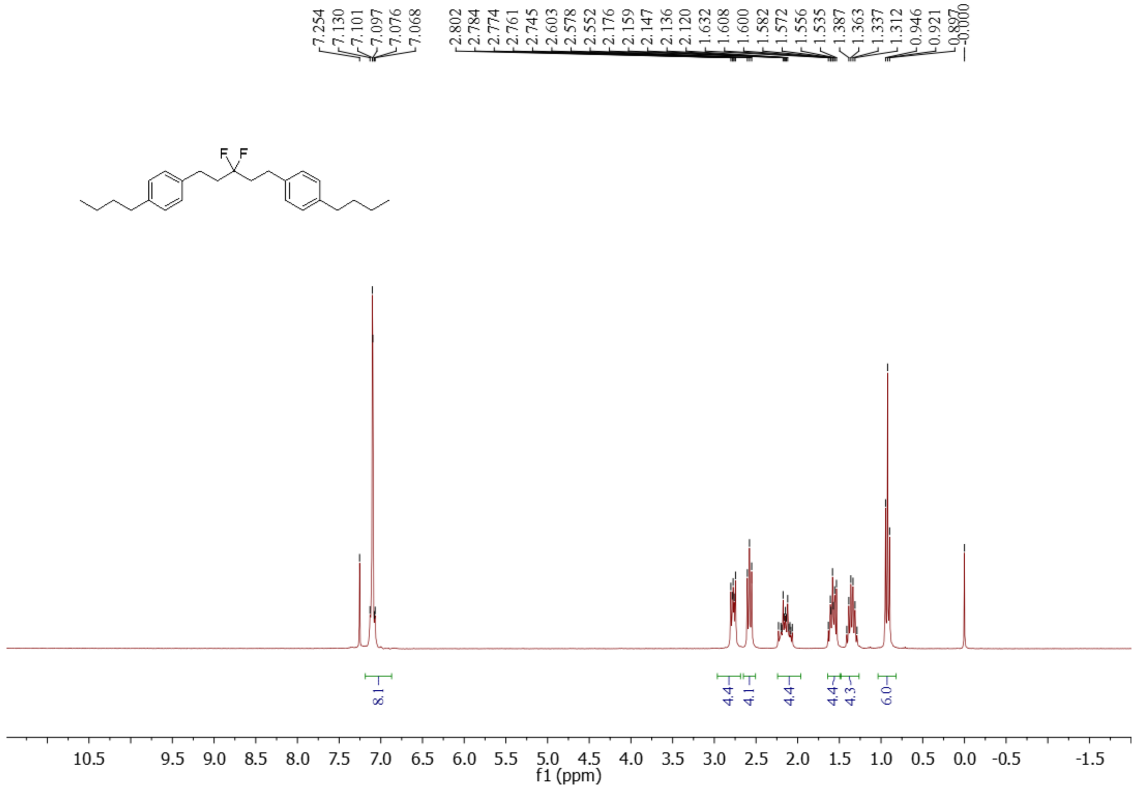

Figure S133. ^{13}C NMR spectrum of unknown *gem*-difluoride **1e**, related to **Figure 2**

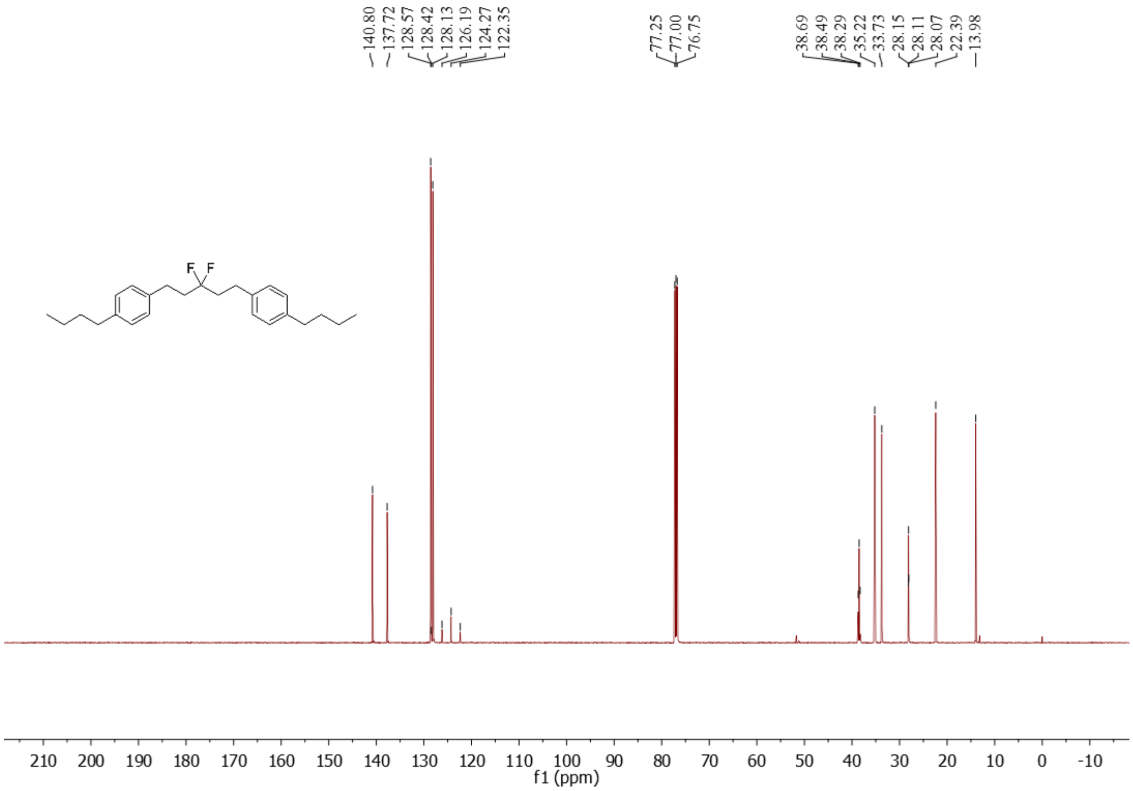

Figure S134. ^{19}F NMR spectrum of unknown *gem*-difluoride **1e**, related to **Figure 2**

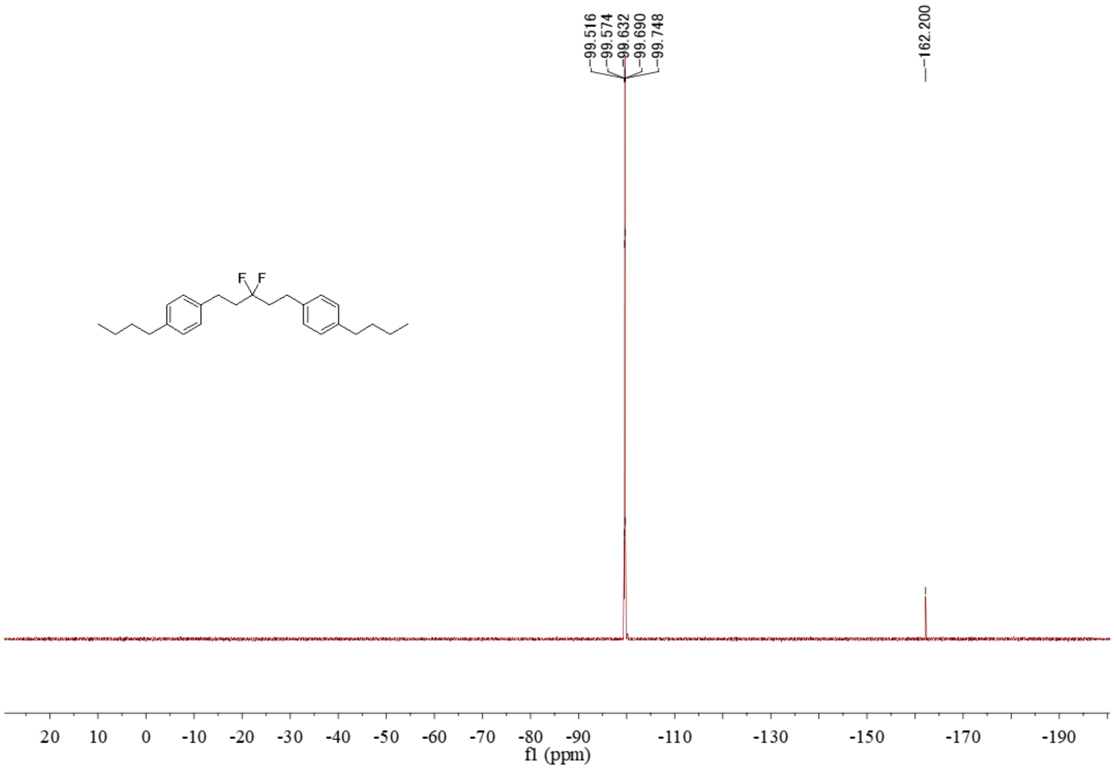

Figure S135. ^{1}H NMR spectrum of unknown *gem*-difluoride **1f**, related to **Figure 2**

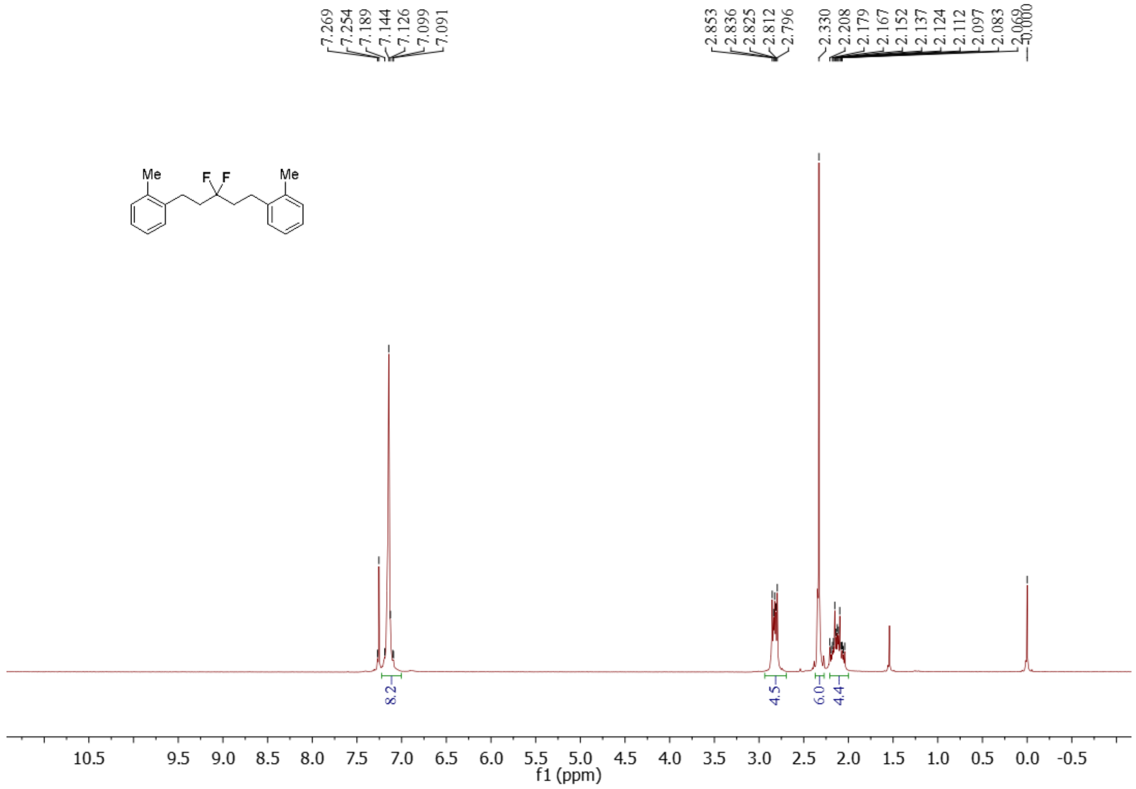

Figure S136. ^{13}C NMR spectrum of unknown *gem*-difluoride **1f**, related to **Figure 2**

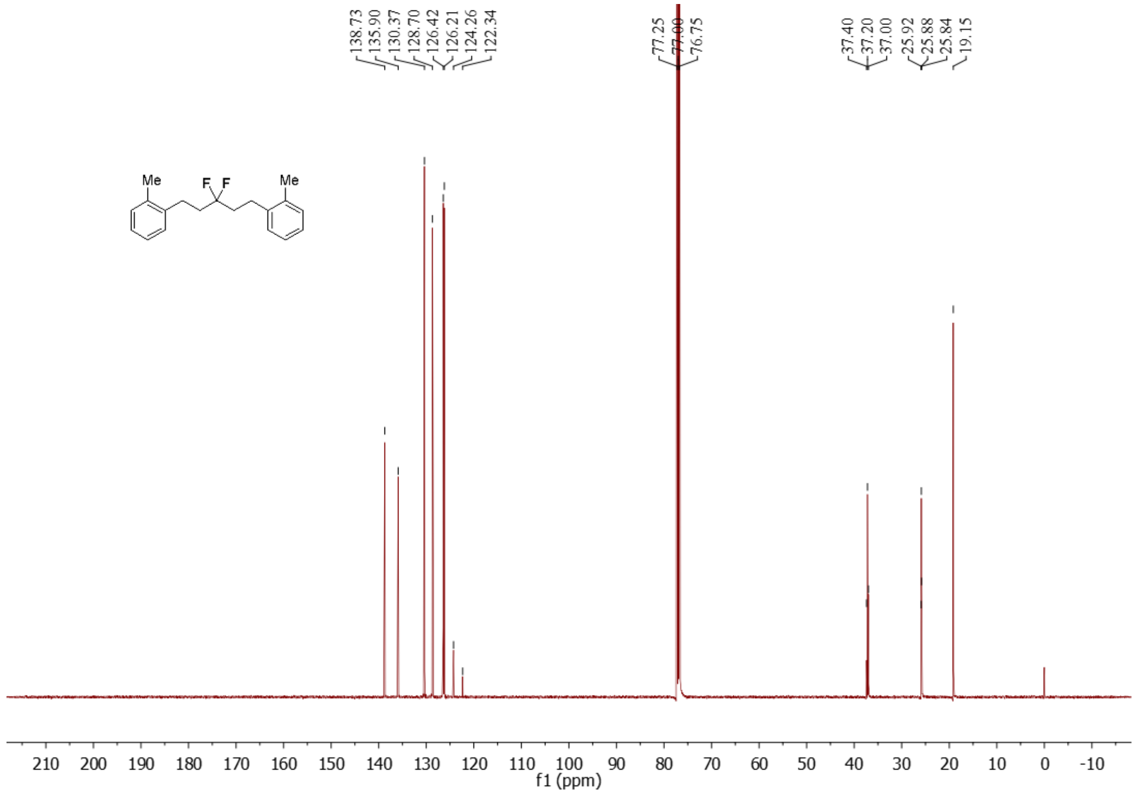

Figure S137. ^{19}F NMR spectrum of unknown *gem*-difluoride **1f**, related to **Figure 2**

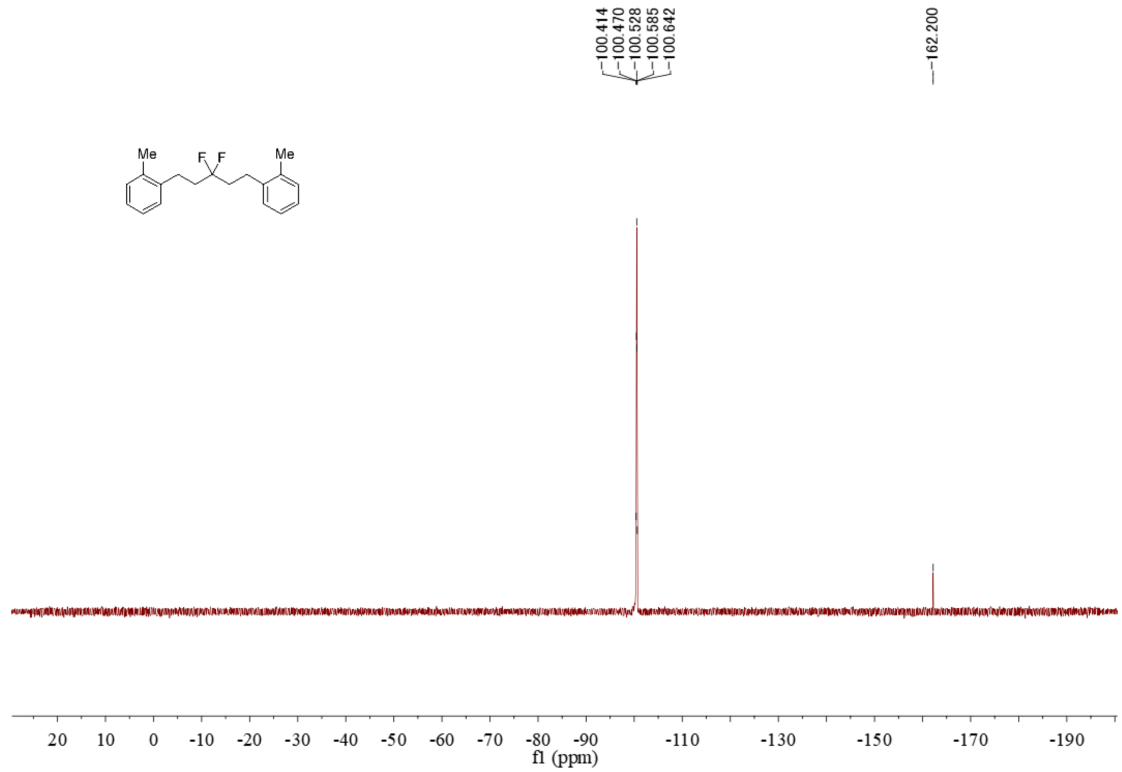

Figure S138. ^{1}H NMR spectrum of unknown *gem*-difluoride **1g**, related to **Figure 2**

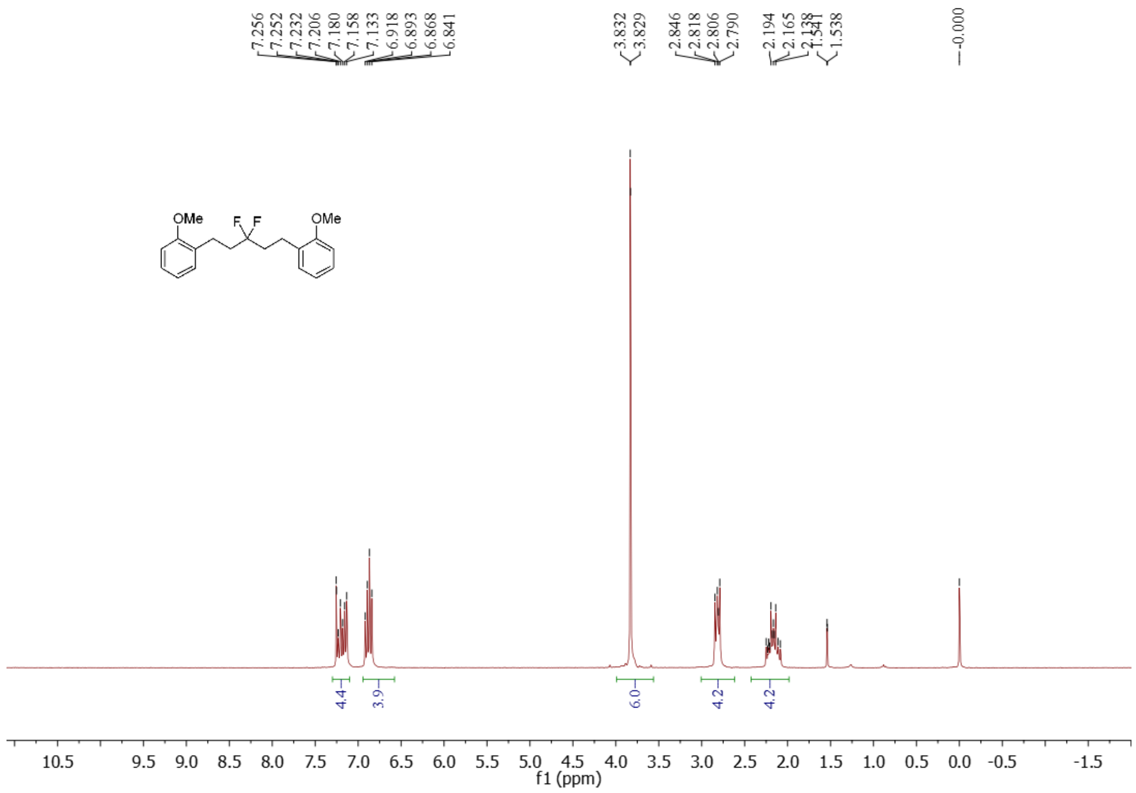

Figure S139. ^{13}C NMR spectrum of unknown *gem*-difluoride **1g**, related to **Figure 2**

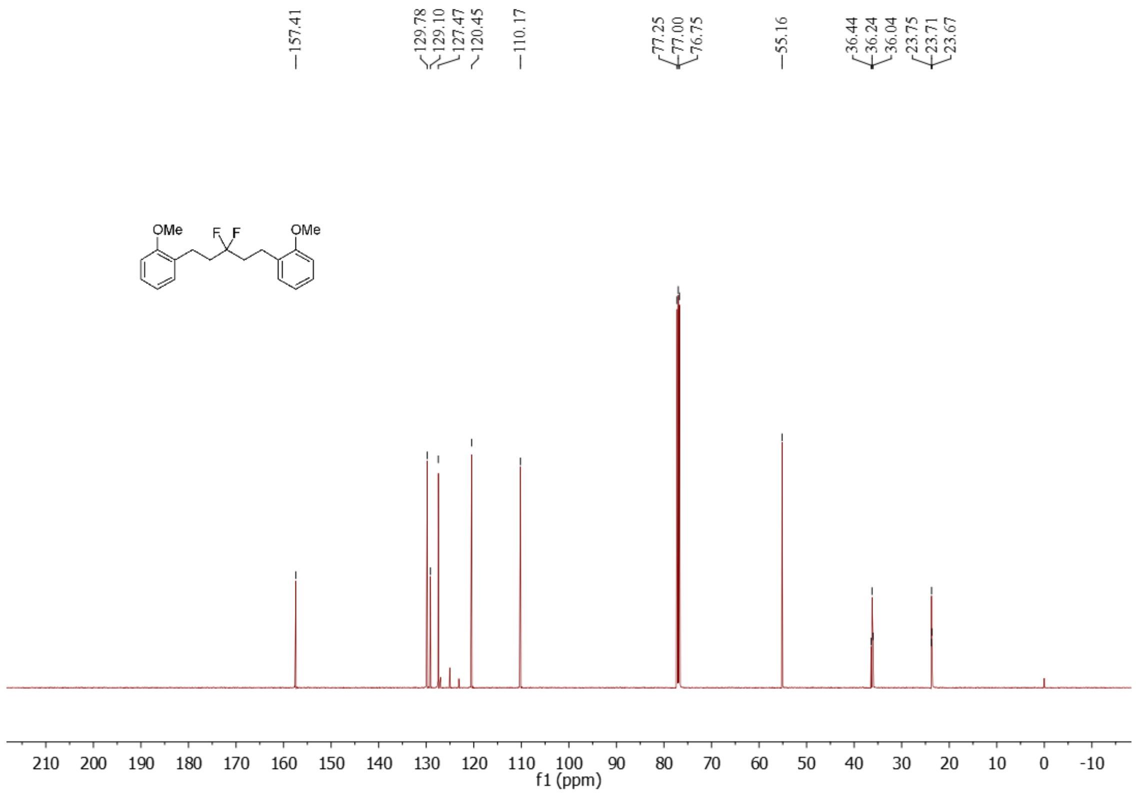

Figure S140. ^{19}F NMR spectrum of unknown *gem*-difluoride **1g**, related to **Figure 2**

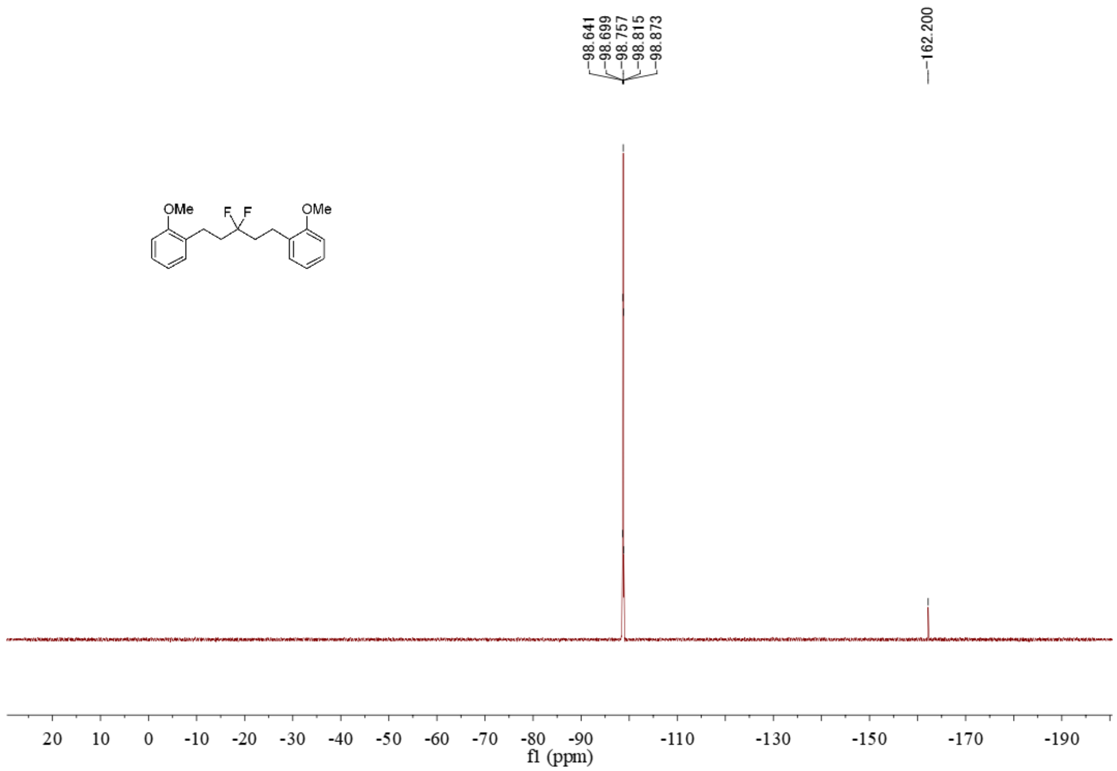

Figure S141. ^{1}H NMR spectrum of unknown *gem*-difluoride **1h**, related to **Figure 2**

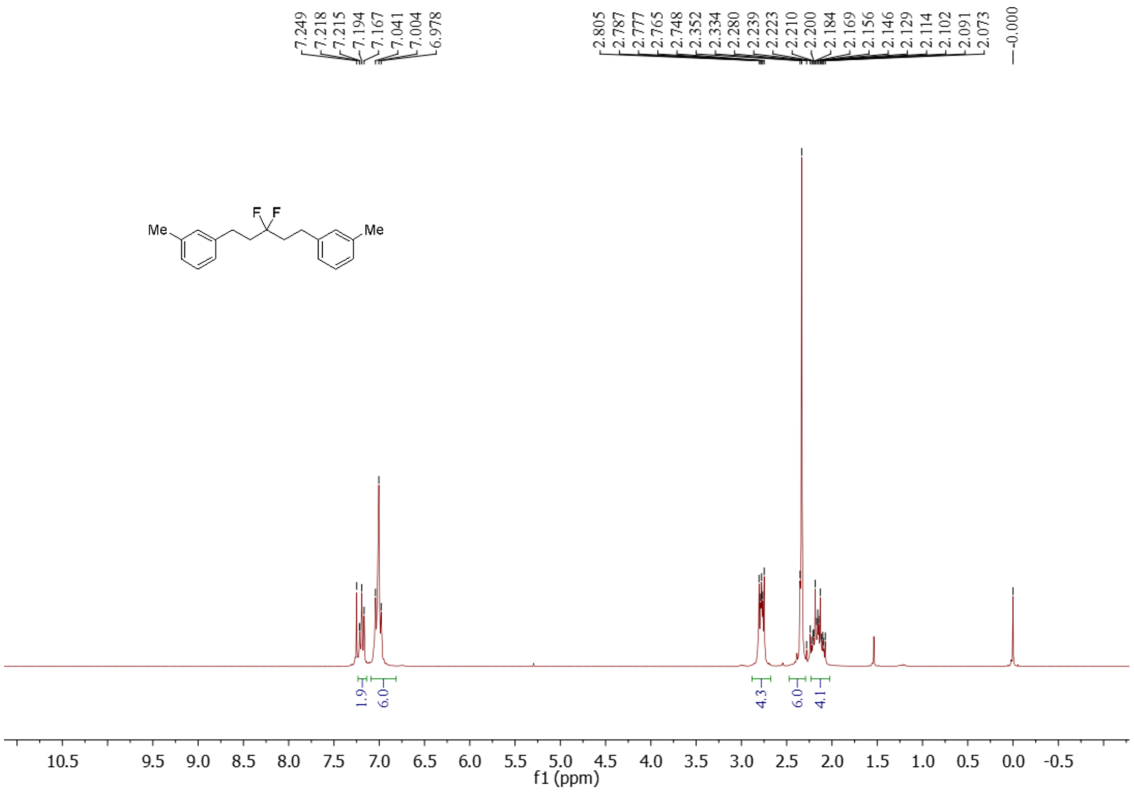

Figure S142. ^{13}C NMR spectrum of unknown *gem*-difluoride **1h**, related to **Figure 2**

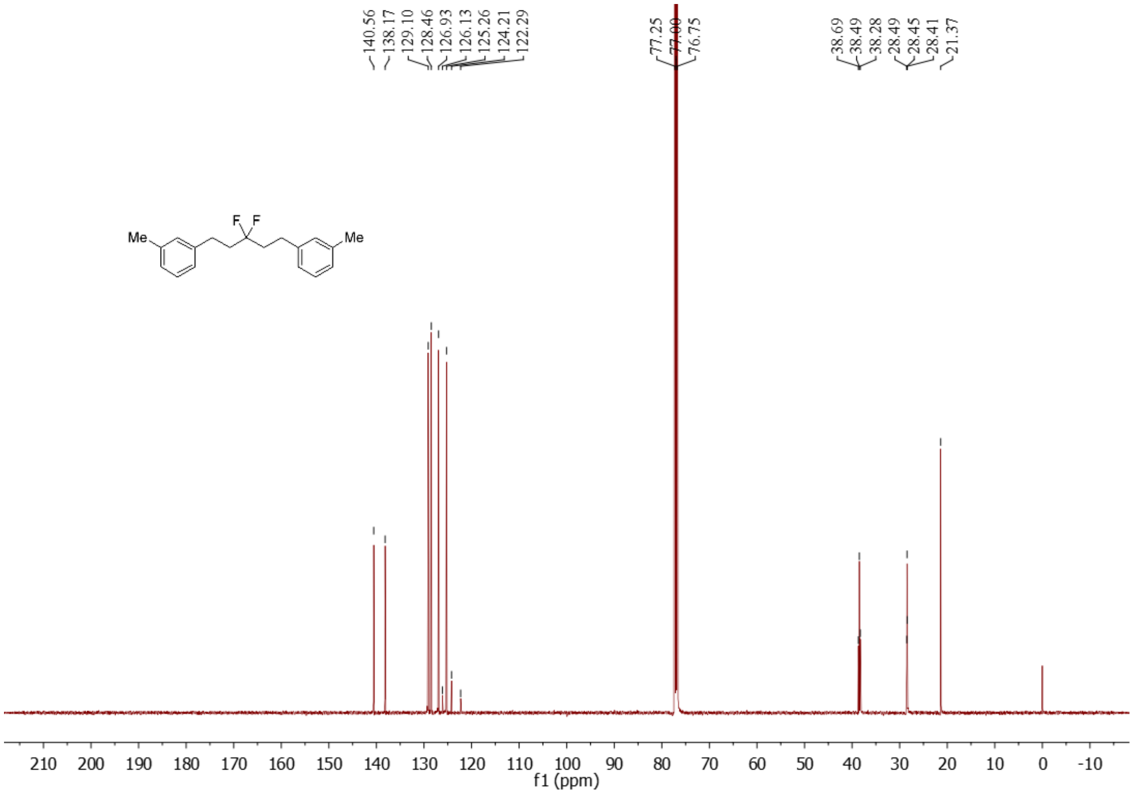

Figure S143. ^{19}F NMR spectrum of unknown *gem*-difluoride **1h**, related to **Figure 2**

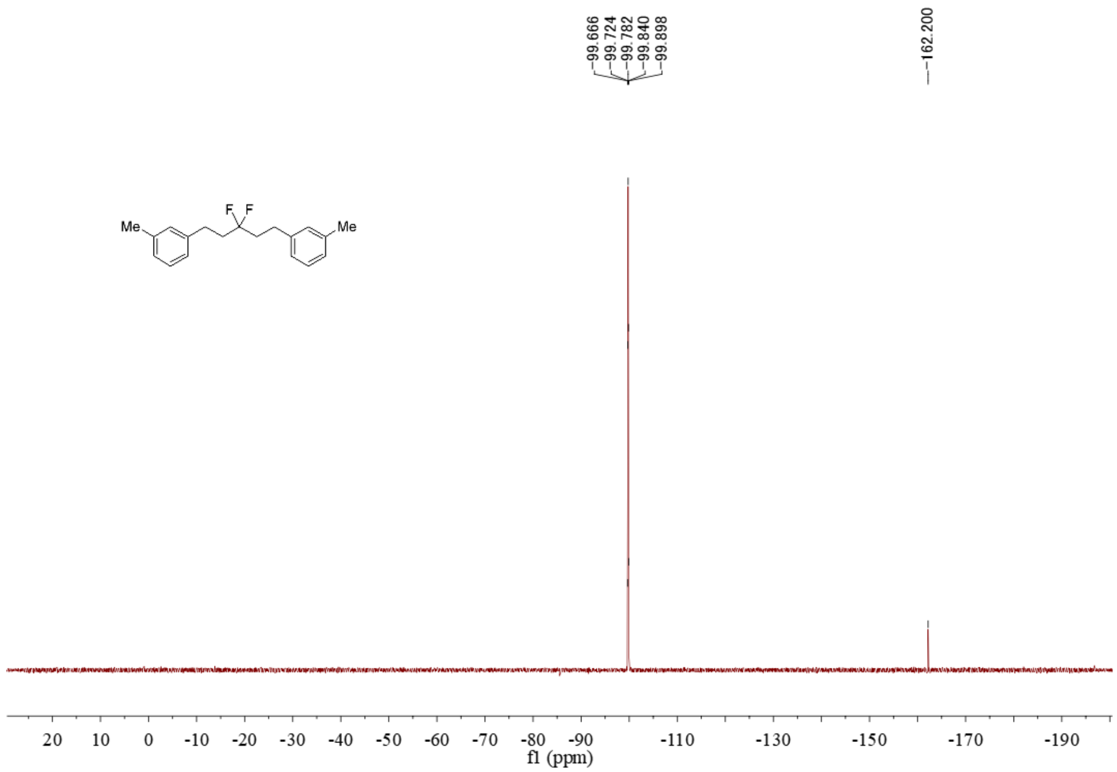

Figure S144. ^{1}H NMR spectrum of unknown *gem*-difluoride **1i**, related to **Figure 2**

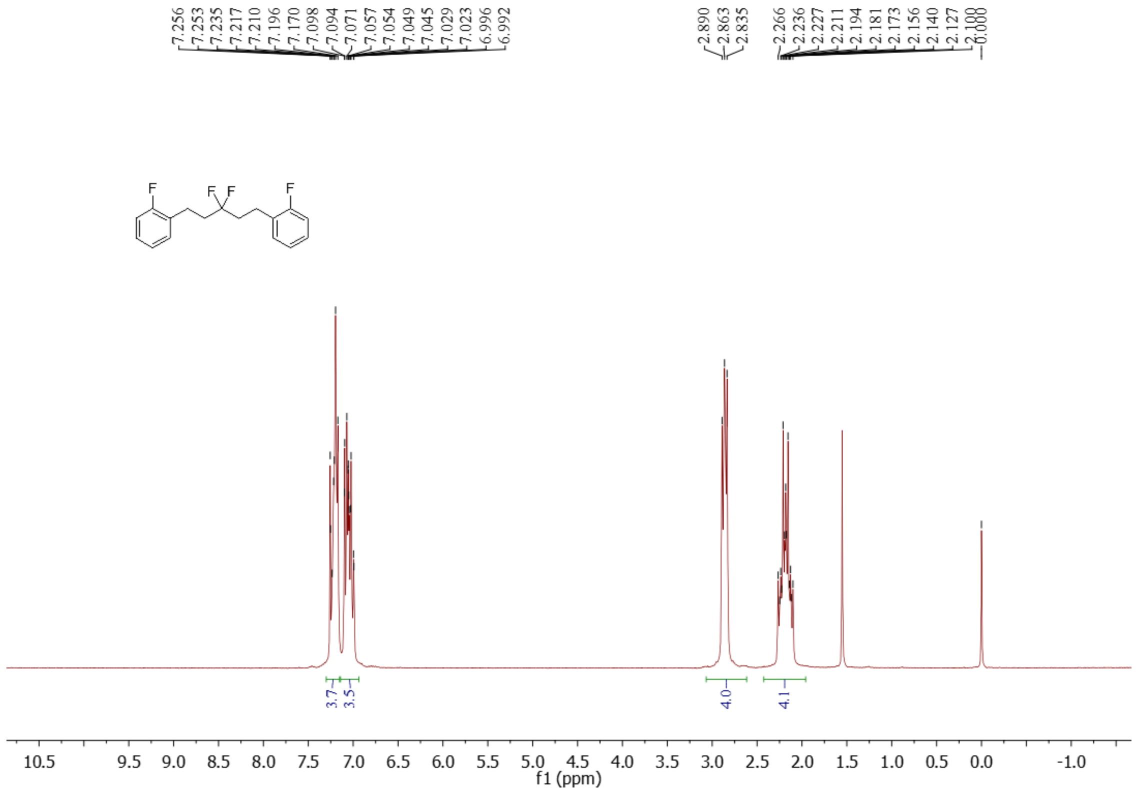

Figure S145. ^{13}C NMR spectrum of unknown *gem*-difluoride **1i**, related to **Figure 2**

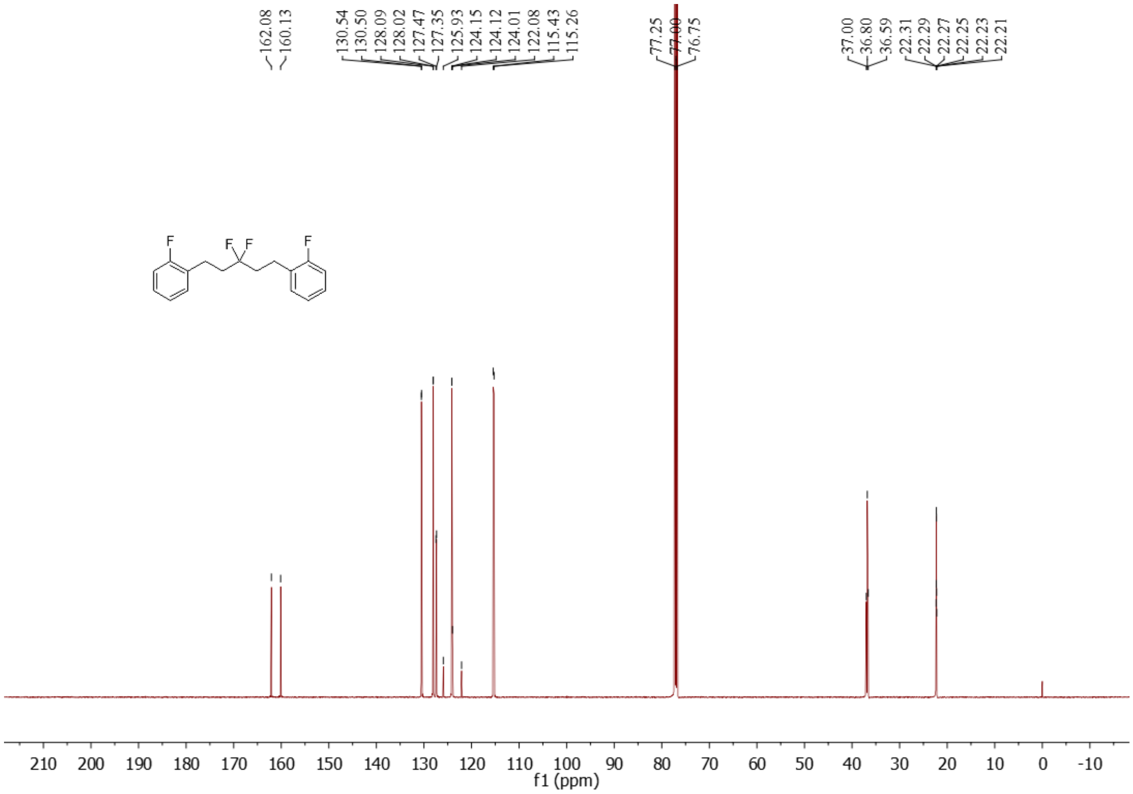

Figure S146. ^{19}F NMR spectrum of unknown *gem*-difluoride **1i**, related to **Figure 2**

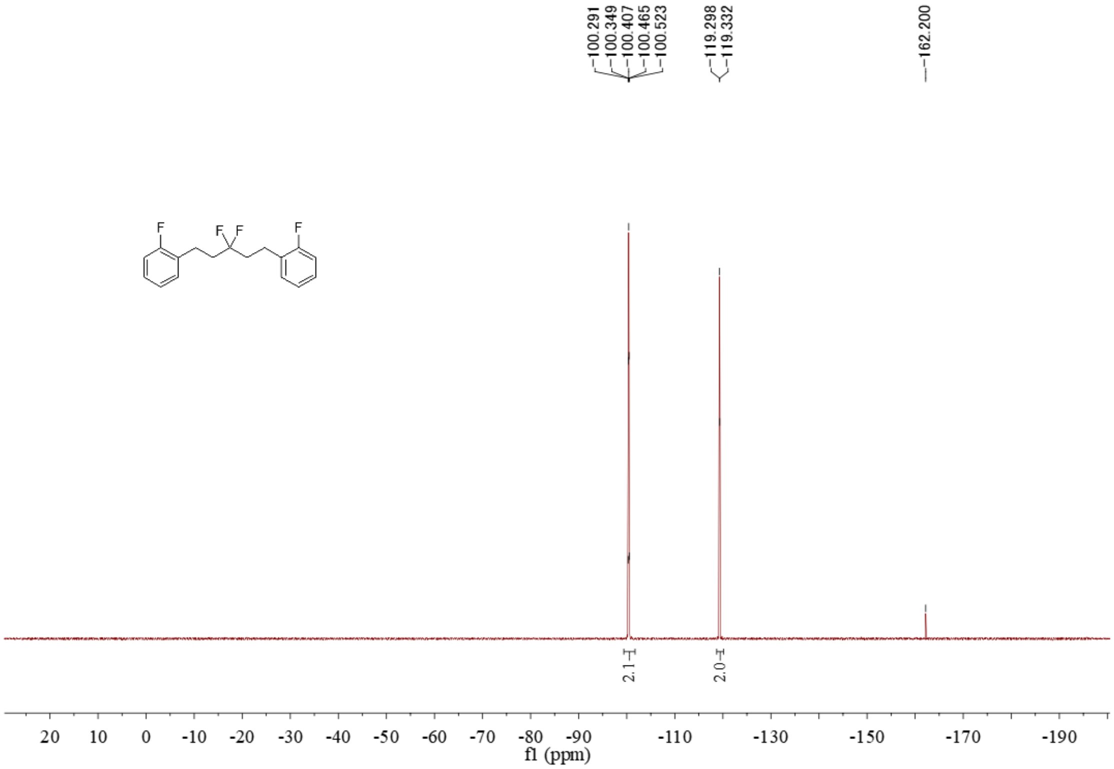

Figure S147. ^{1}H NMR spectrum of unknown *gem*-difluoride **1j**, related to **Figure 2**

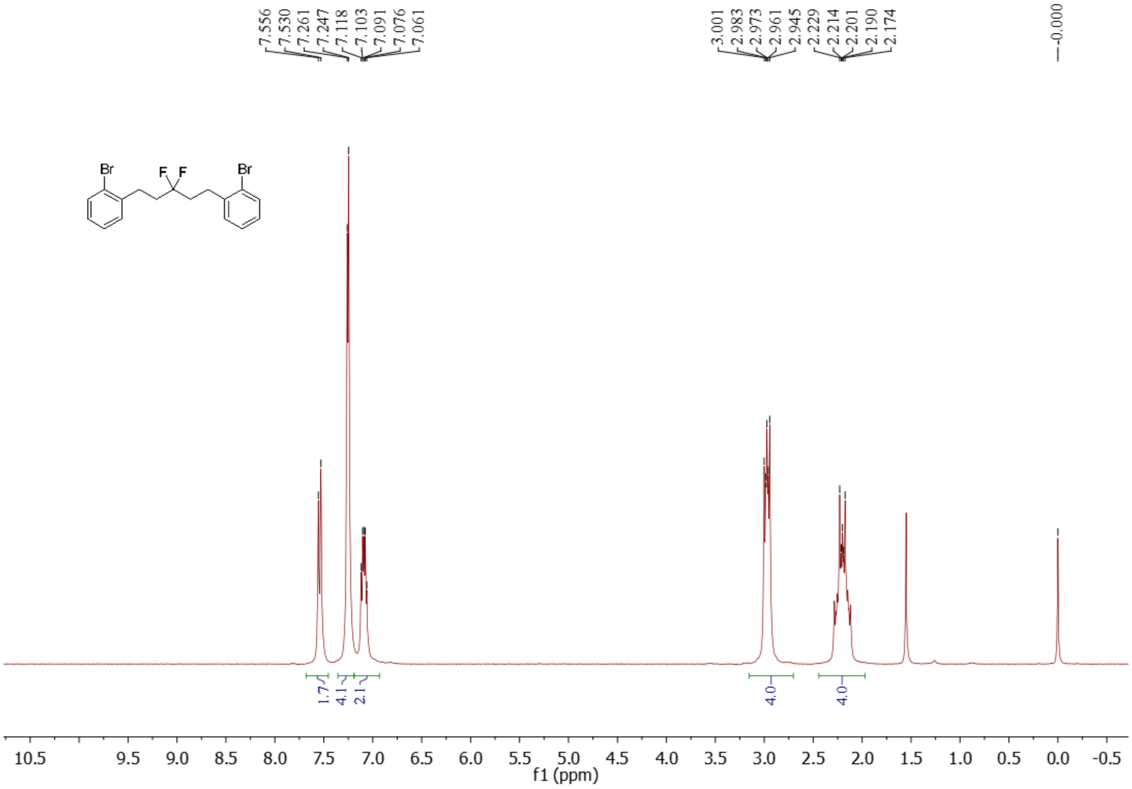

Figure S148. ^{13}C NMR spectrum of unknown *gem*-difluoride **1j**, related to **Figure 2**

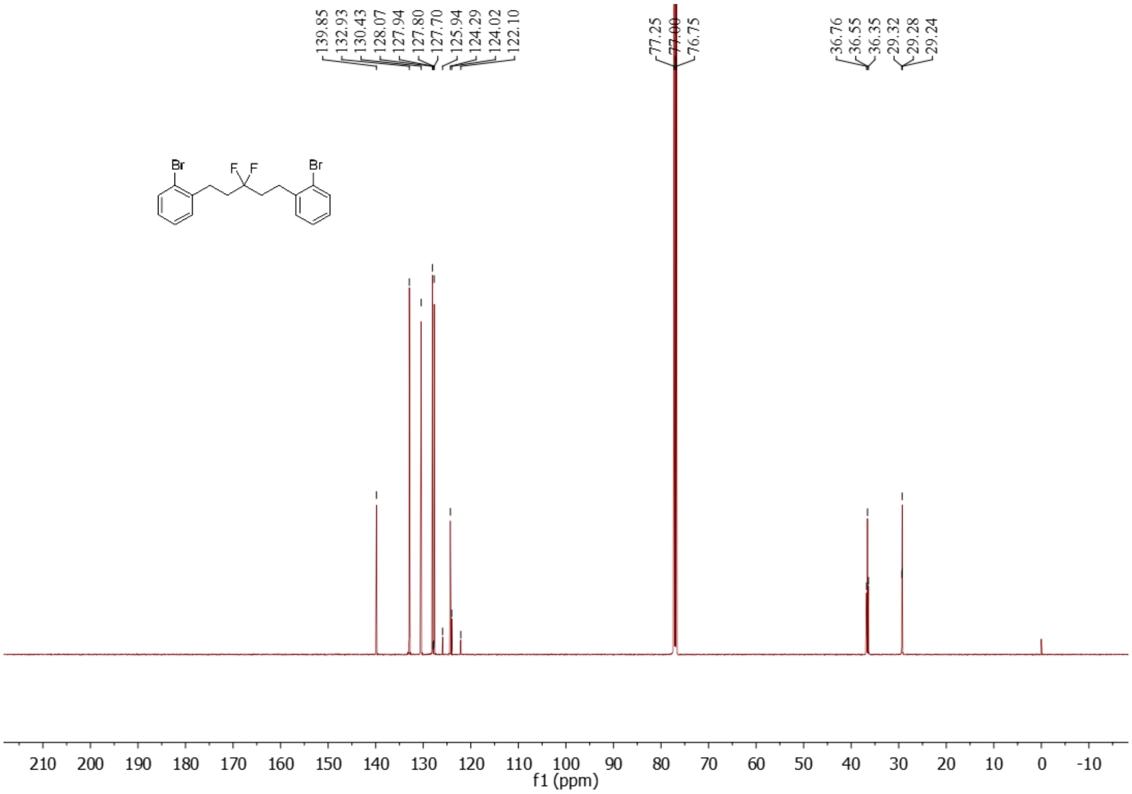

Figure S149. ^{19}F NMR spectrum of unknown *gem*-difluoride **1j**, related to **Figure 2**

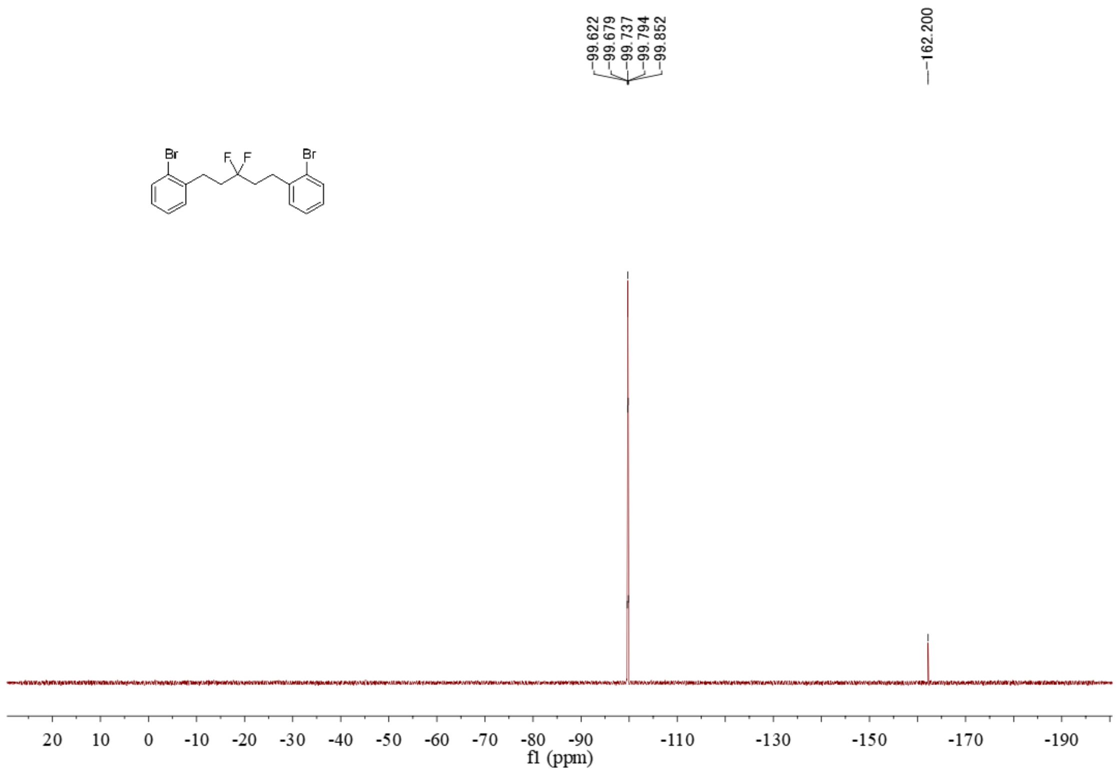

Figure S150. ^{1}H NMR spectrum of unknown *gem*-difluoride **1k**, related to **Figure 2**

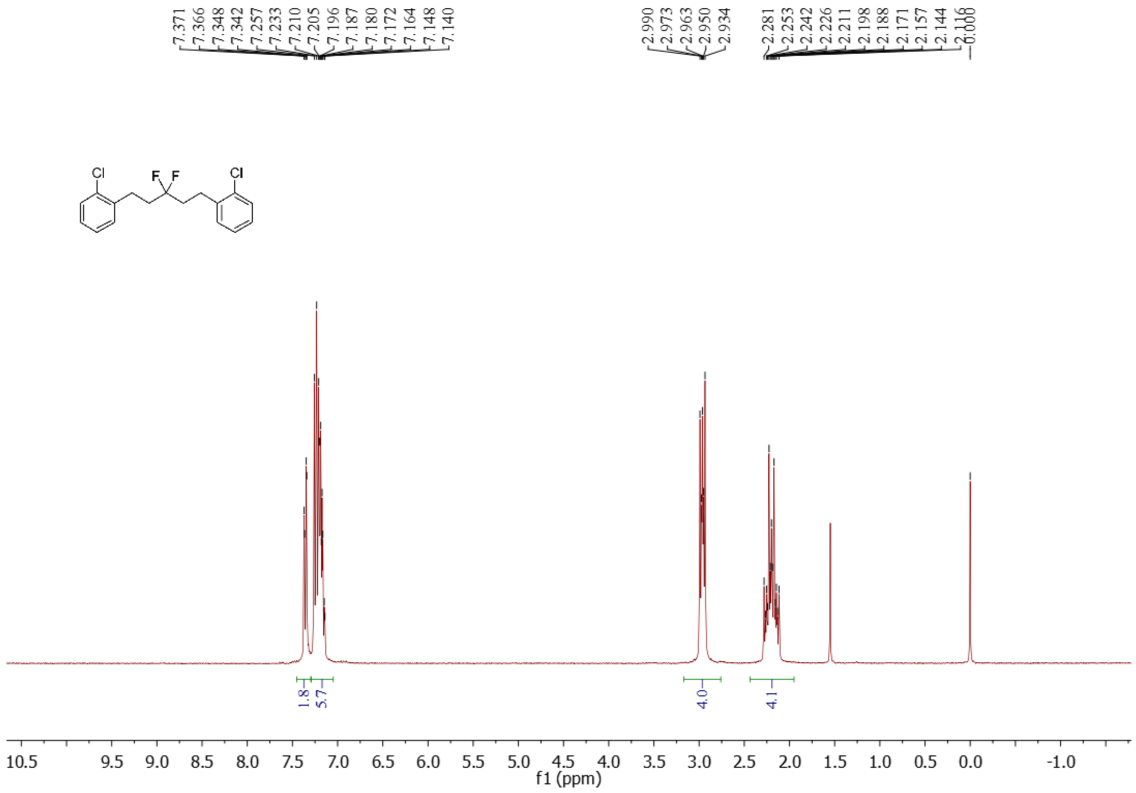

Figure S151. ^{13}C NMR spectrum of unknown *gem*-difluoride **1k**, related to **Figure 2**

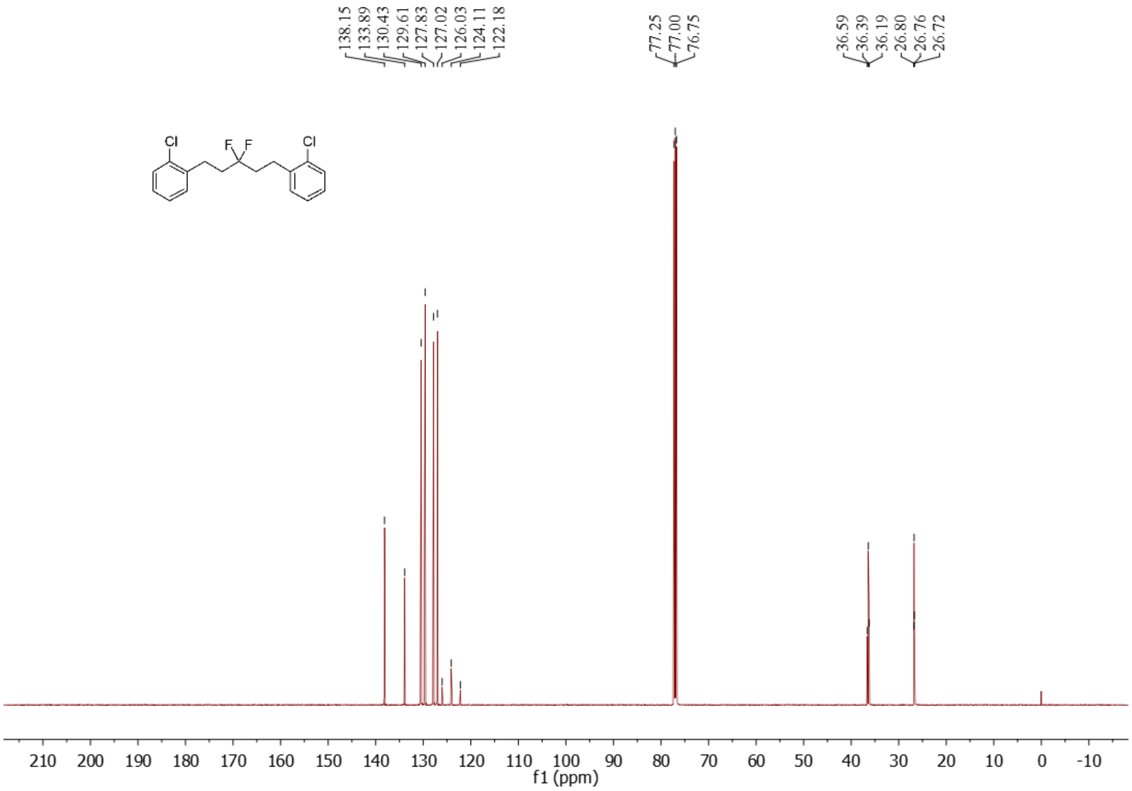

Figure S152. ^{19}F NMR spectrum of unknown *gem*-difluoride **1k**, related to **Figure 2**

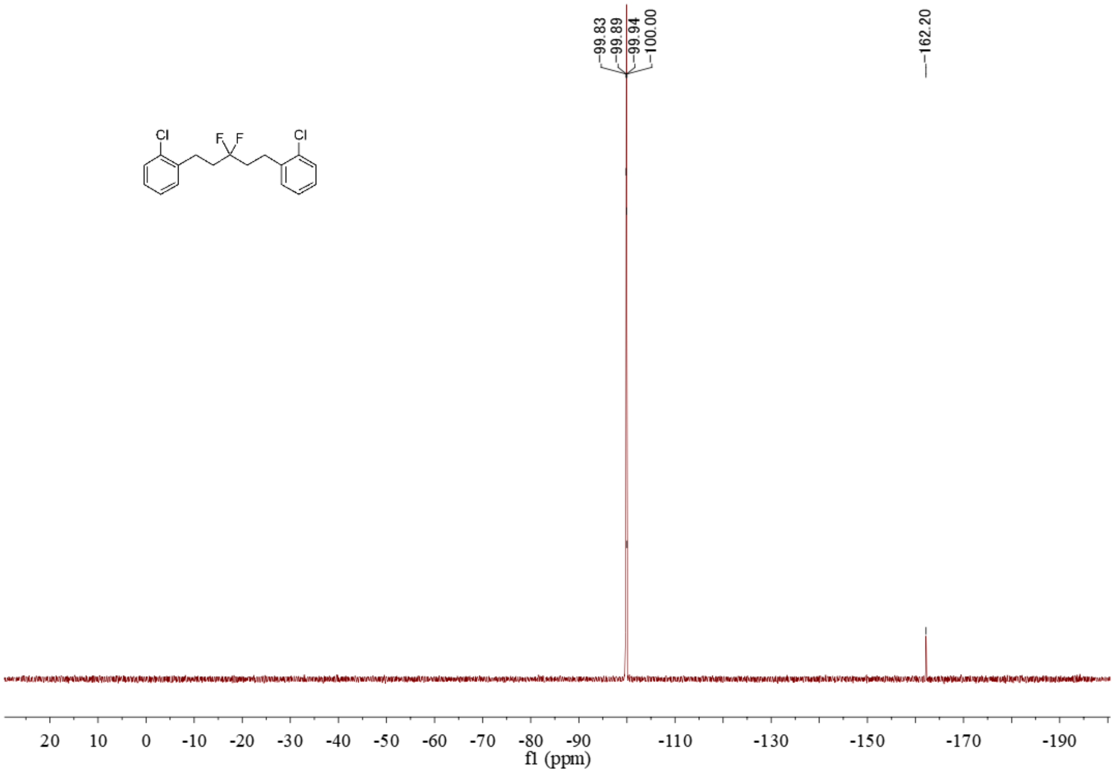

Figure S153. ^{1}H NMR spectrum of unknown *gem*-difluoride **1l**, related to **Figure 2**

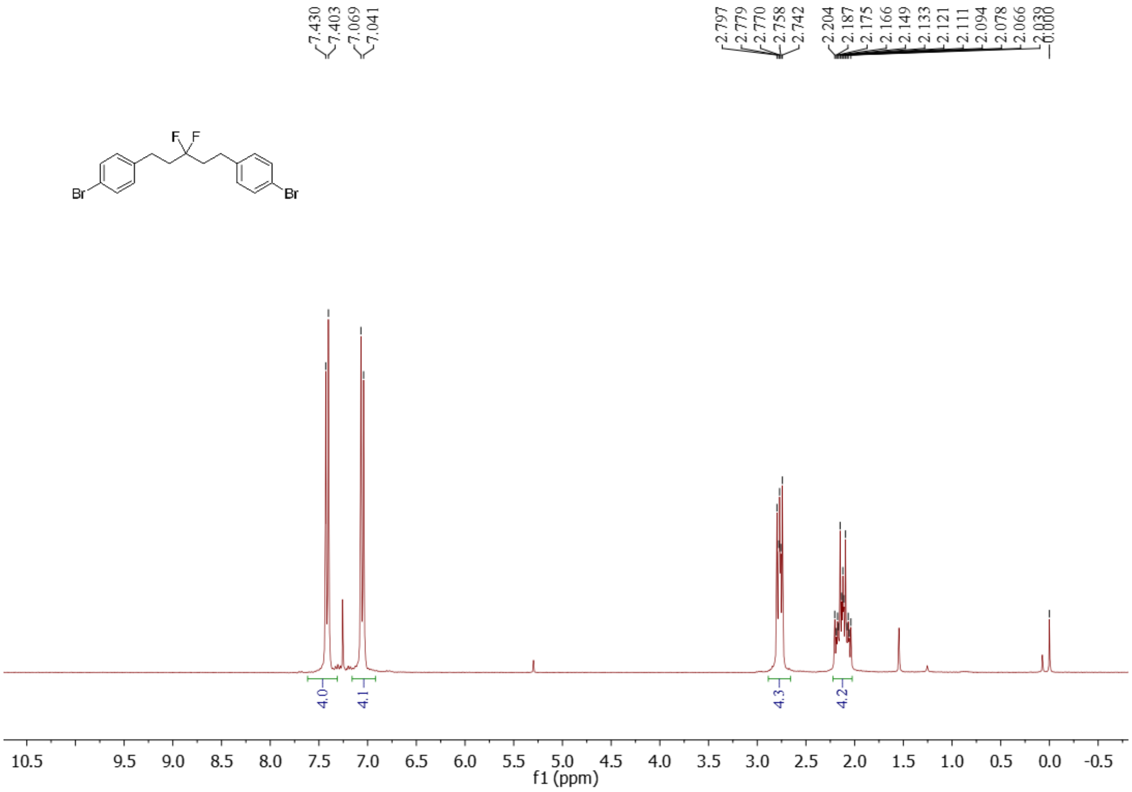

Figure S154. ^{13}C NMR spectrum of unknown *gem*-difluoride **1l**, related to **Figure 2**

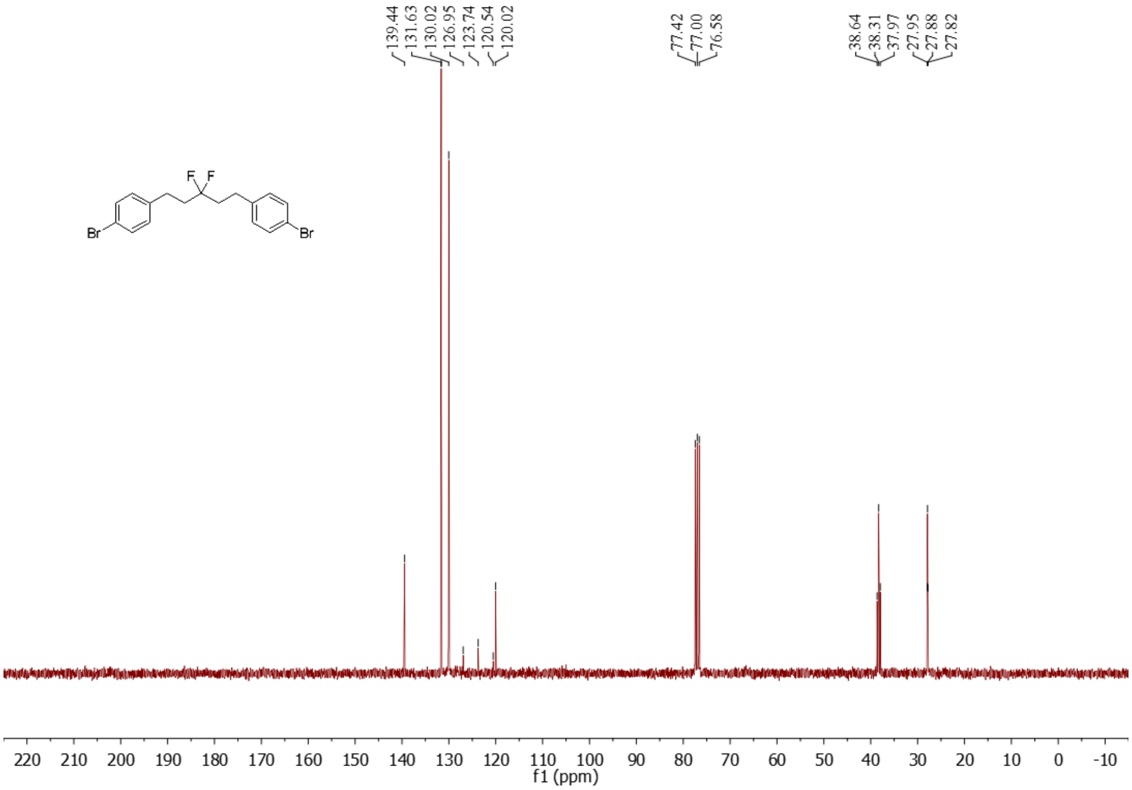

Figure S155. ^{19}F NMR spectrum of unknown *gem*-difluoride **1l**, related to **Figure 2**

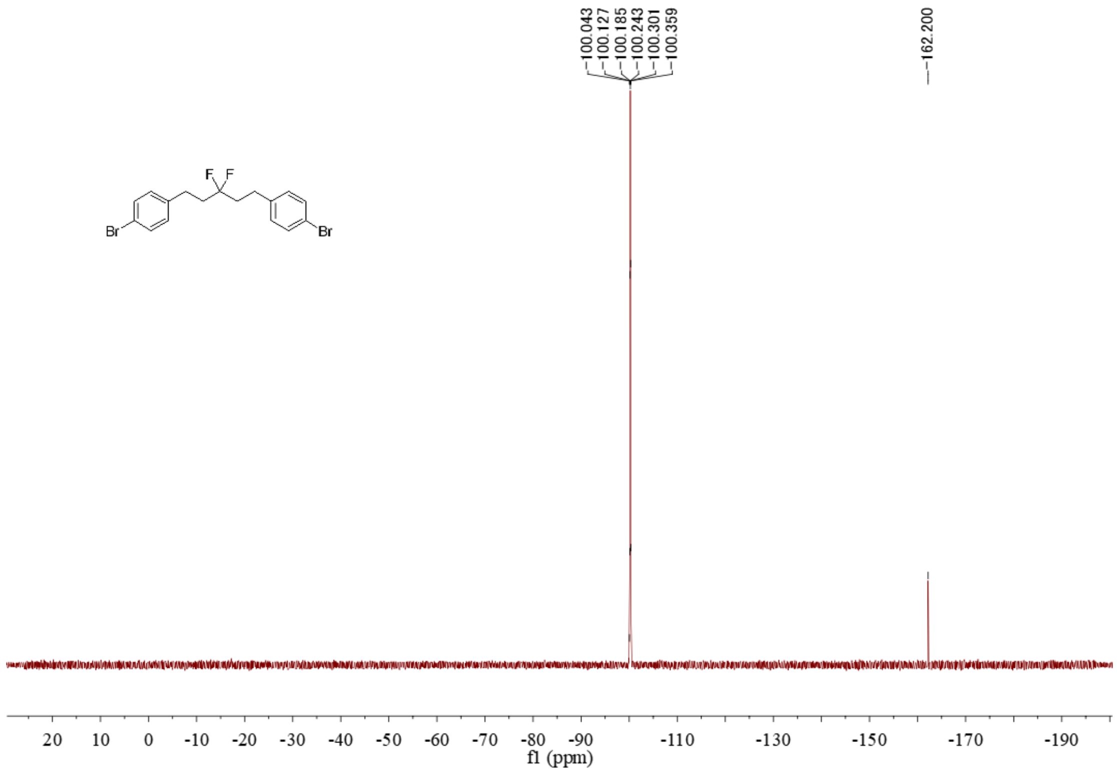

Figure S156. ^{1}H NMR spectrum of unknown *gem*-difluoride **1m**, related to **Figure 2**

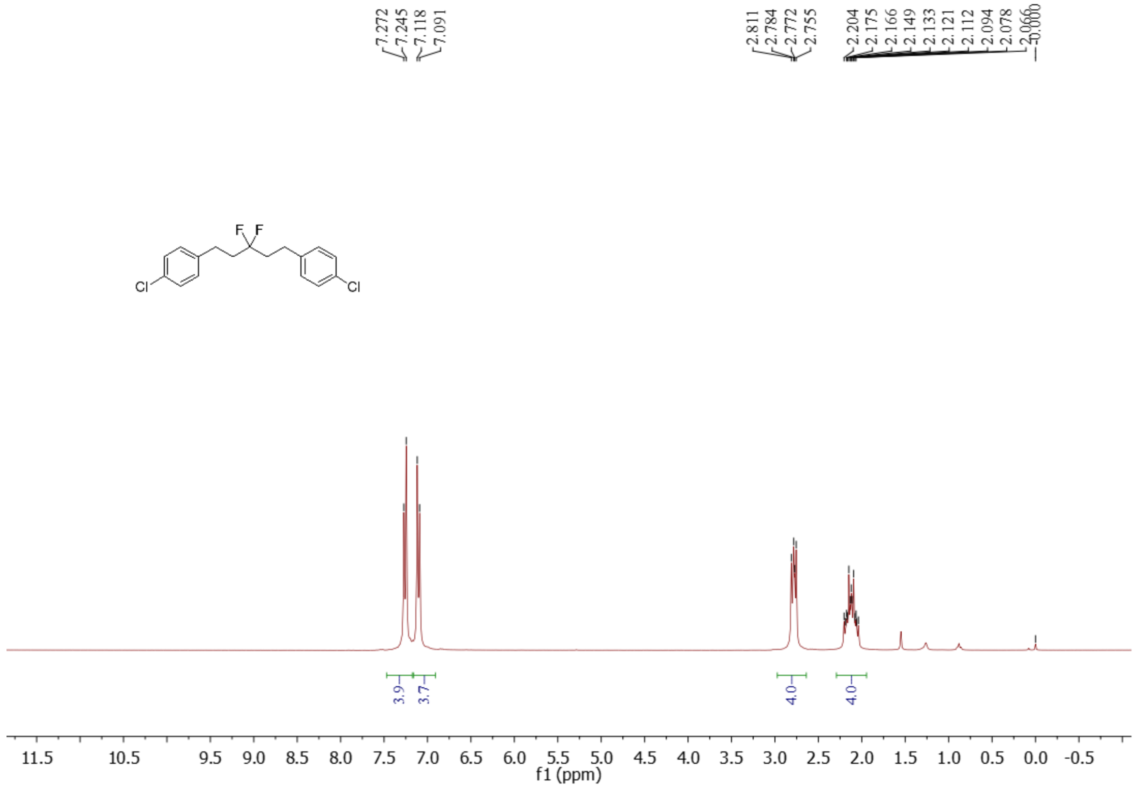

Figure S157. ^{13}C NMR spectrum of unknown *gem*-difluoride **1m**, related to **Figure 2**

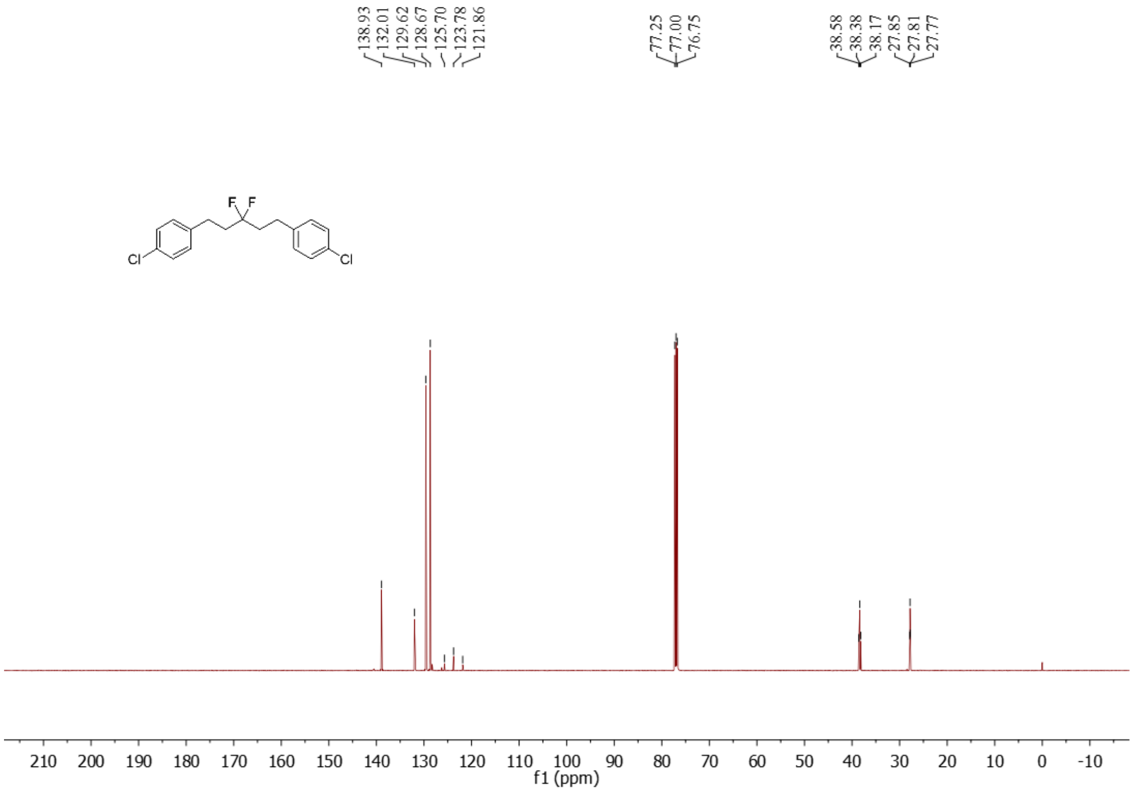

Figure S158. ^{19}F NMR spectrum of unknown *gem*-difluoride **1m**, related to **Figure 2**

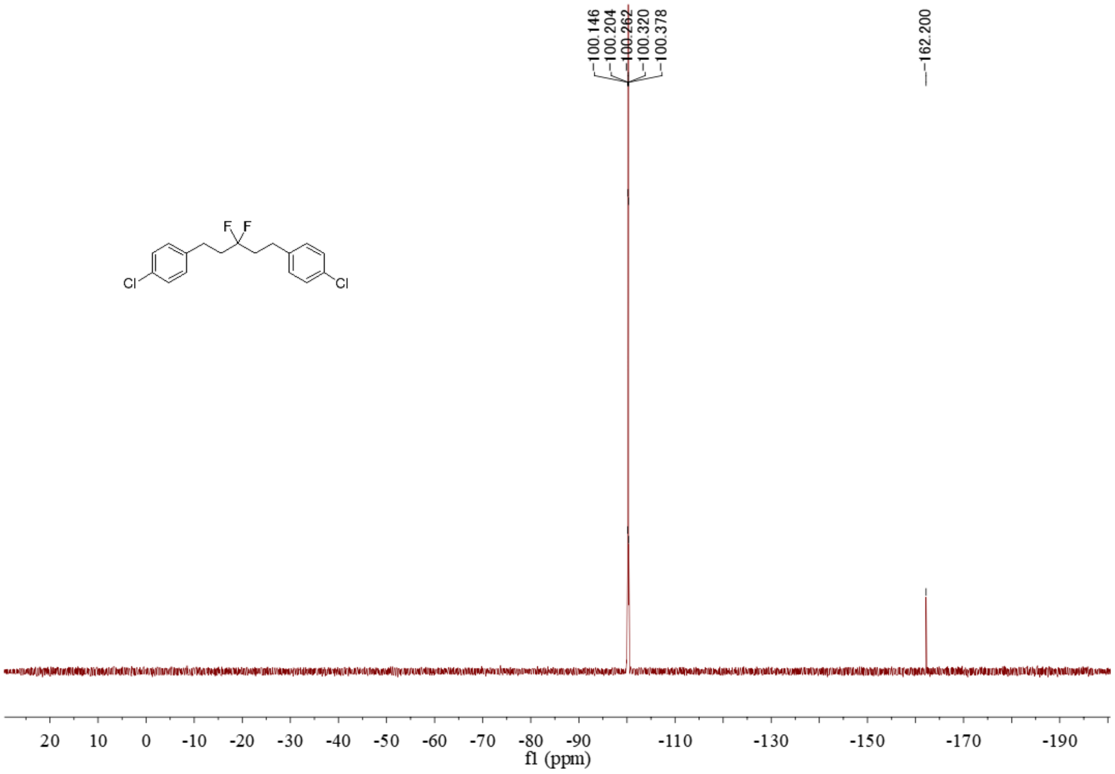

Figure S159. ^{1}H NMR spectrum of unknown *gem*-difluoride **1n**, related to **Figure 2**

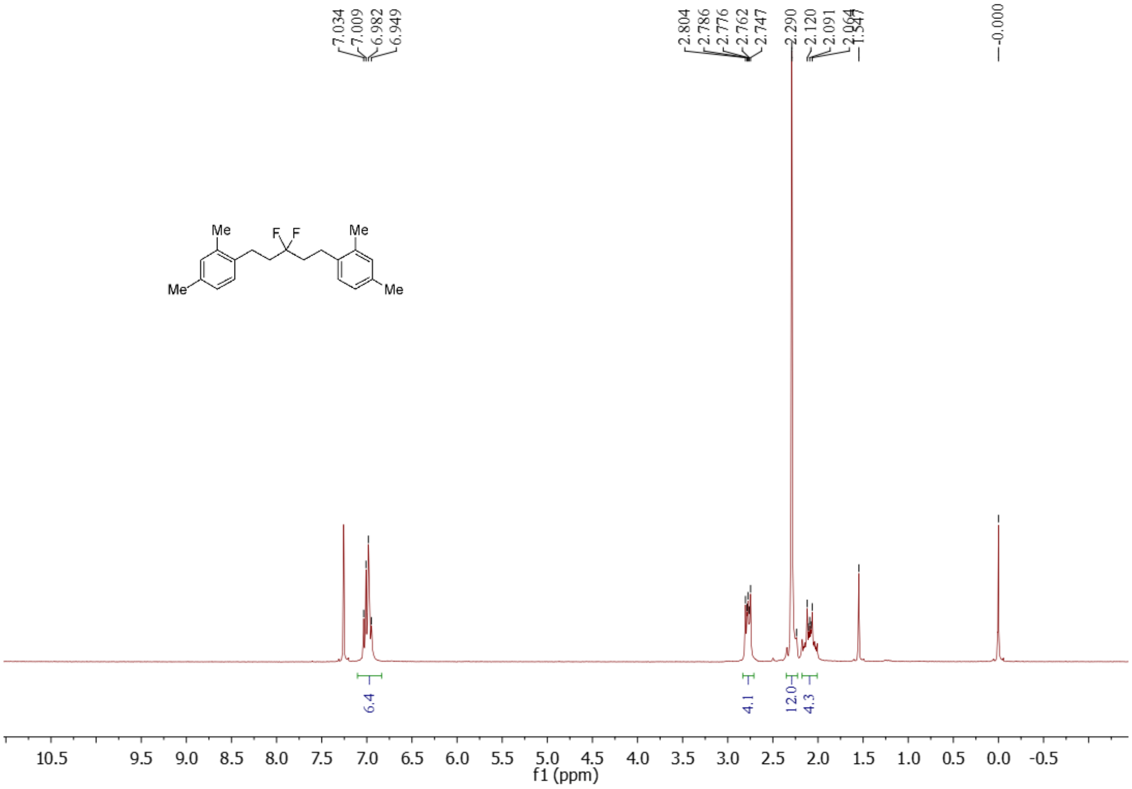

Figure S160. ^{13}C NMR spectrum of unknown *gem*-difluoride **1n**, related to **Figure 2**

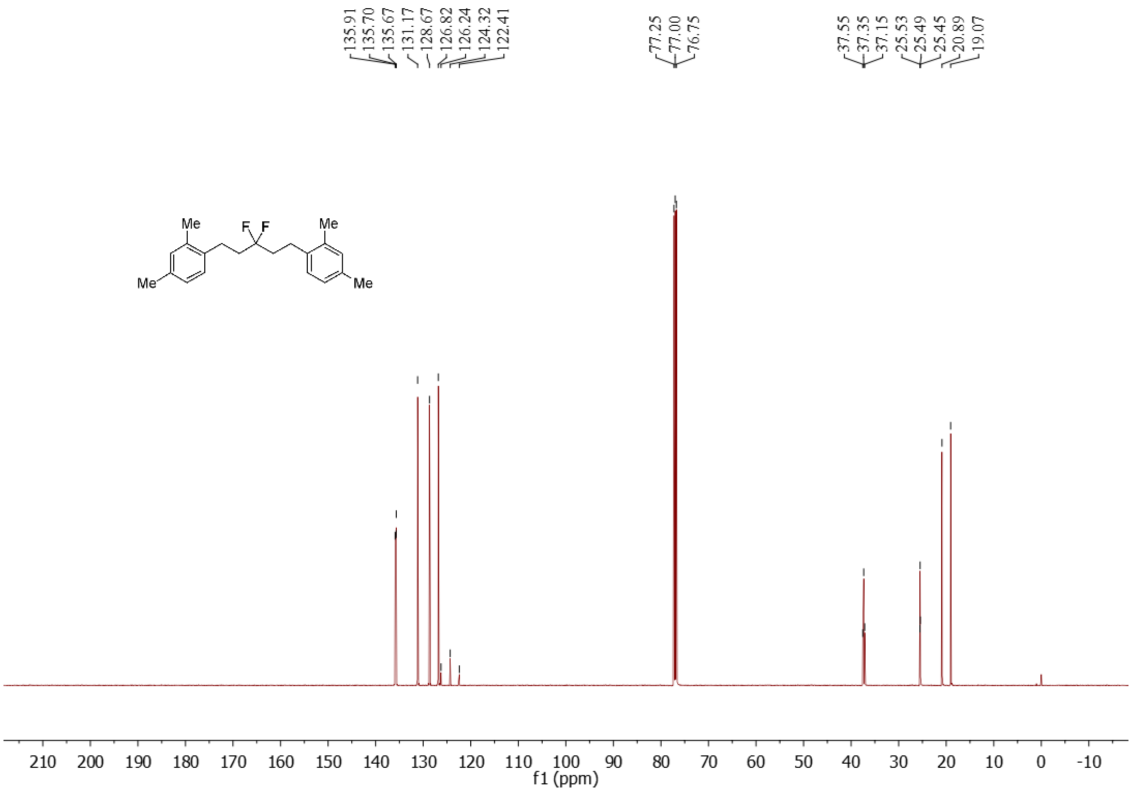

Figure S161. ^{19}F NMR spectrum of unknown *gem*-difluoride **1n**, related to **Figure 2**

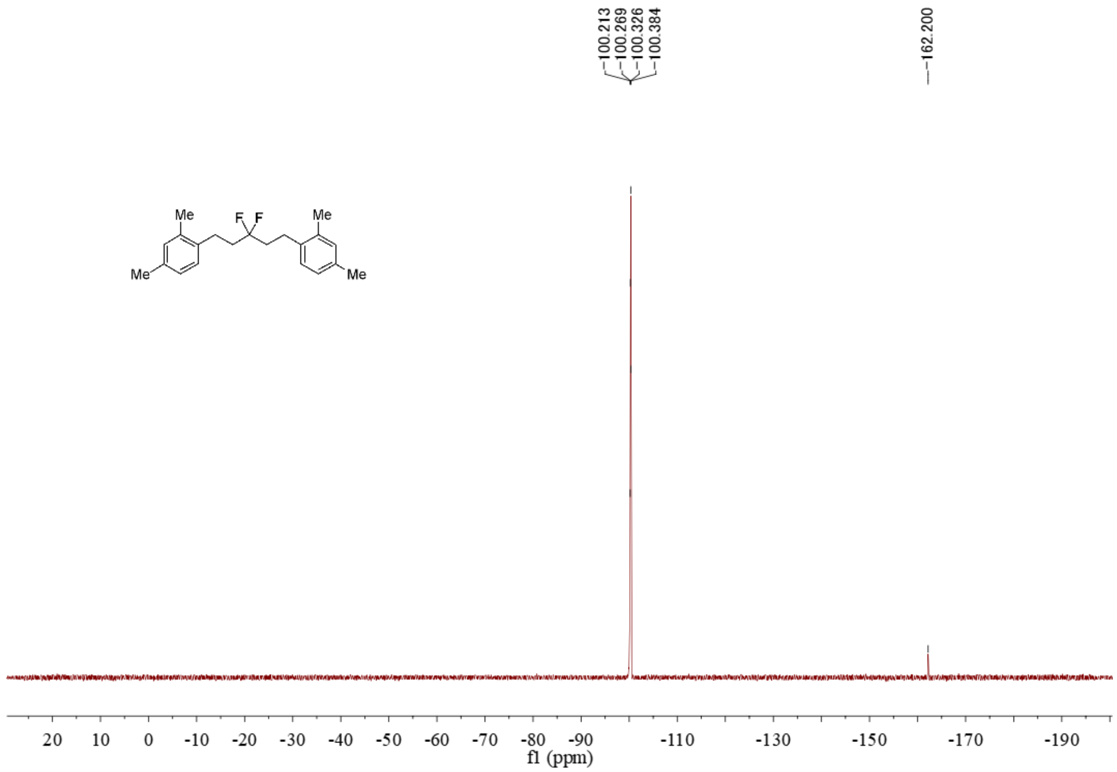

Figure S162. ^{1}H NMR spectrum of unknown *gem*-difluoride **1o**, related to **Figure 2**

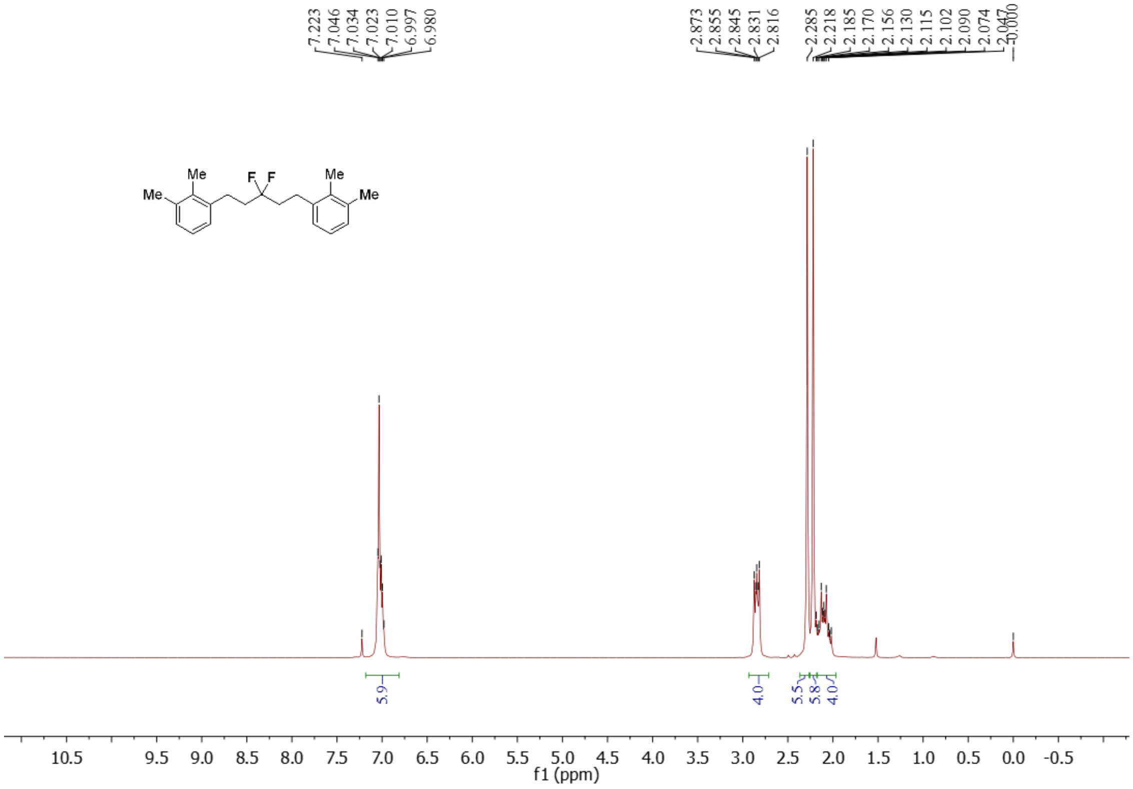

Figure S163. ^{13}C NMR spectrum of unknown *gem*-difluoride **1o**, related to **Figure 2**

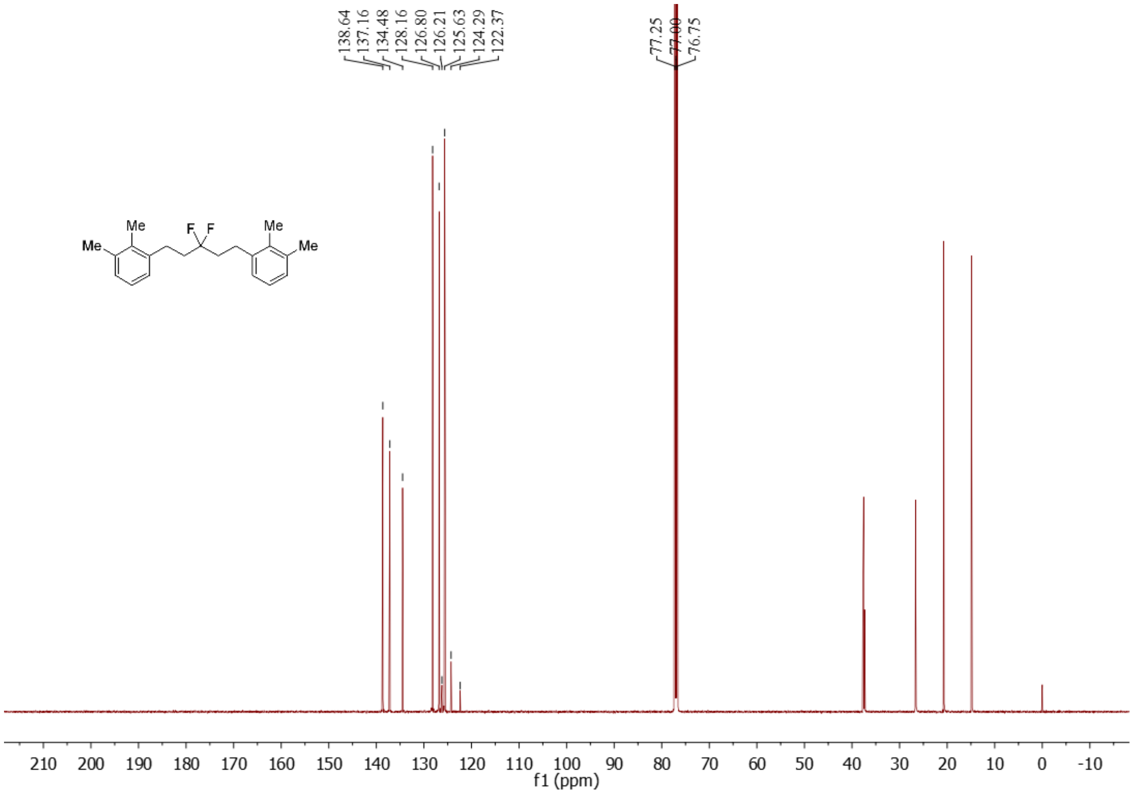

Figure S164. ^{19}F NMR spectrum of unknown *gem*-difluoride **1o**, related to **Figure 2**

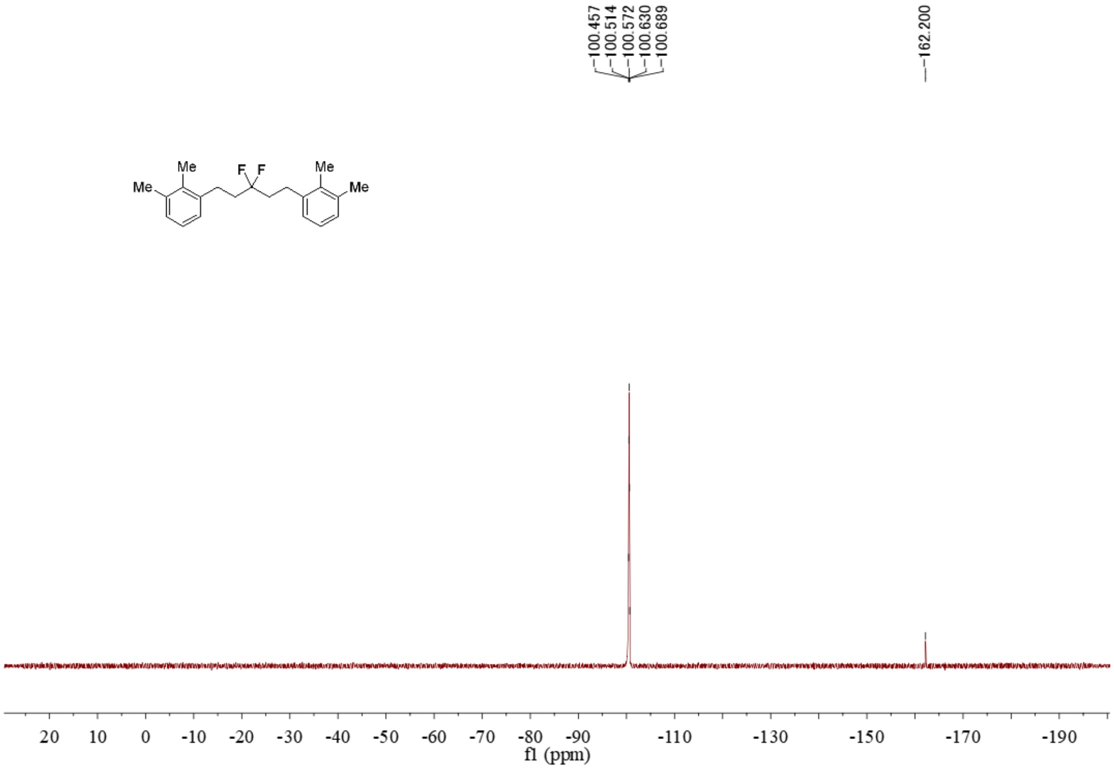

Figure S165. ^{1}H NMR spectrum of unknown *gem*-difluoride **1p**, related to **Figure 2**

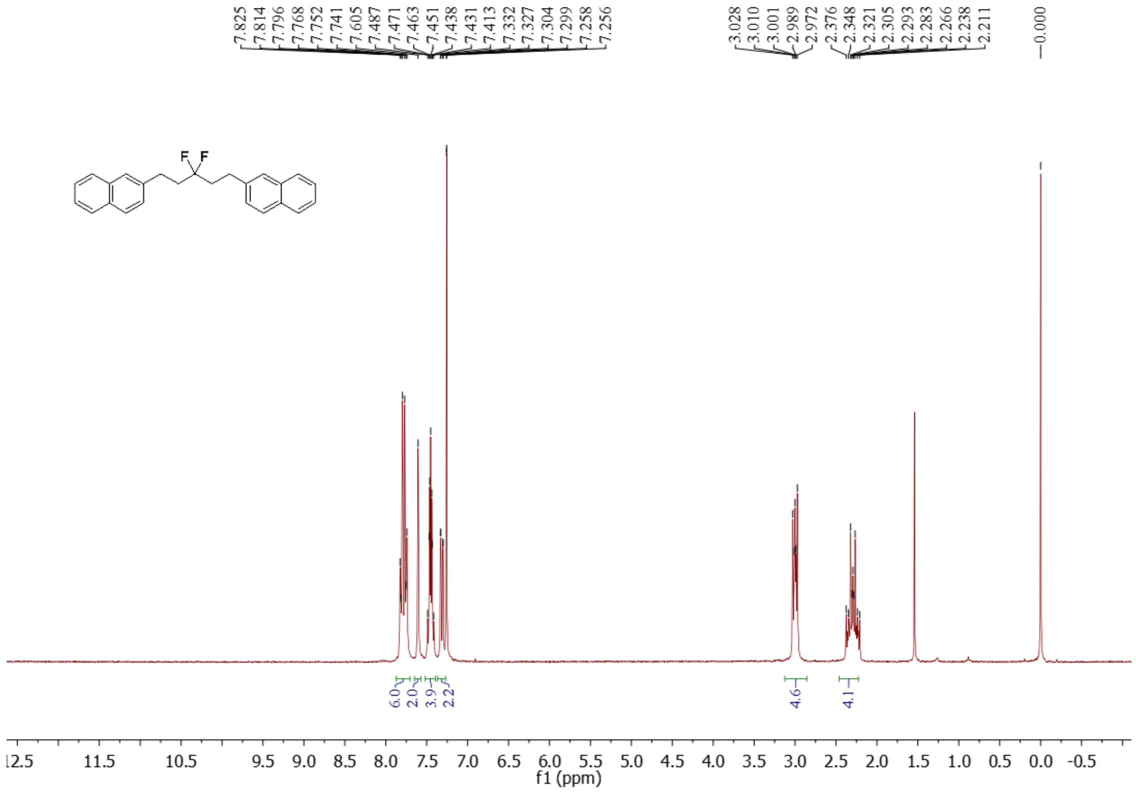

Figure S166. ^{13}C NMR spectrum of unknown *gem*-difluoride **1p**, related to **Figure 2**

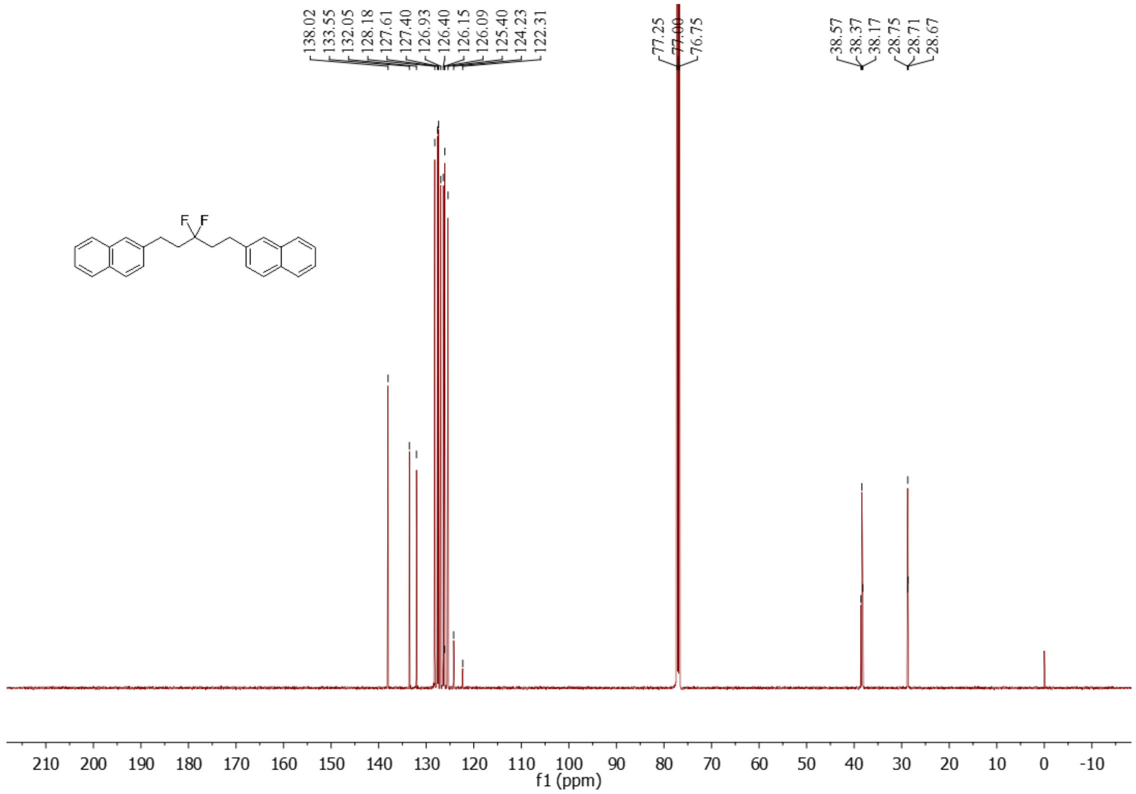

Figure S167. ^{19}F NMR spectrum of unknown *gem*-difluoride **1p**, related to **Figure 2**

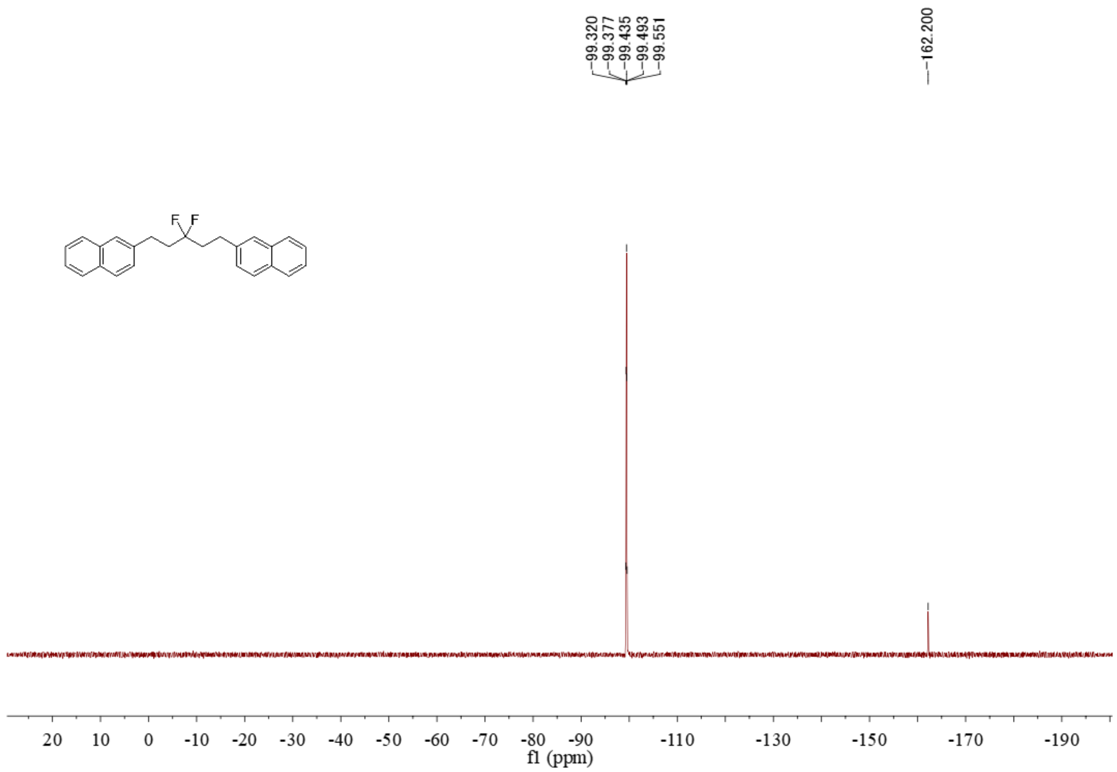

Figure S168. ^{1}H NMR spectrum of unknown *gem*-difluoride **1q**, related to **Figure 2**

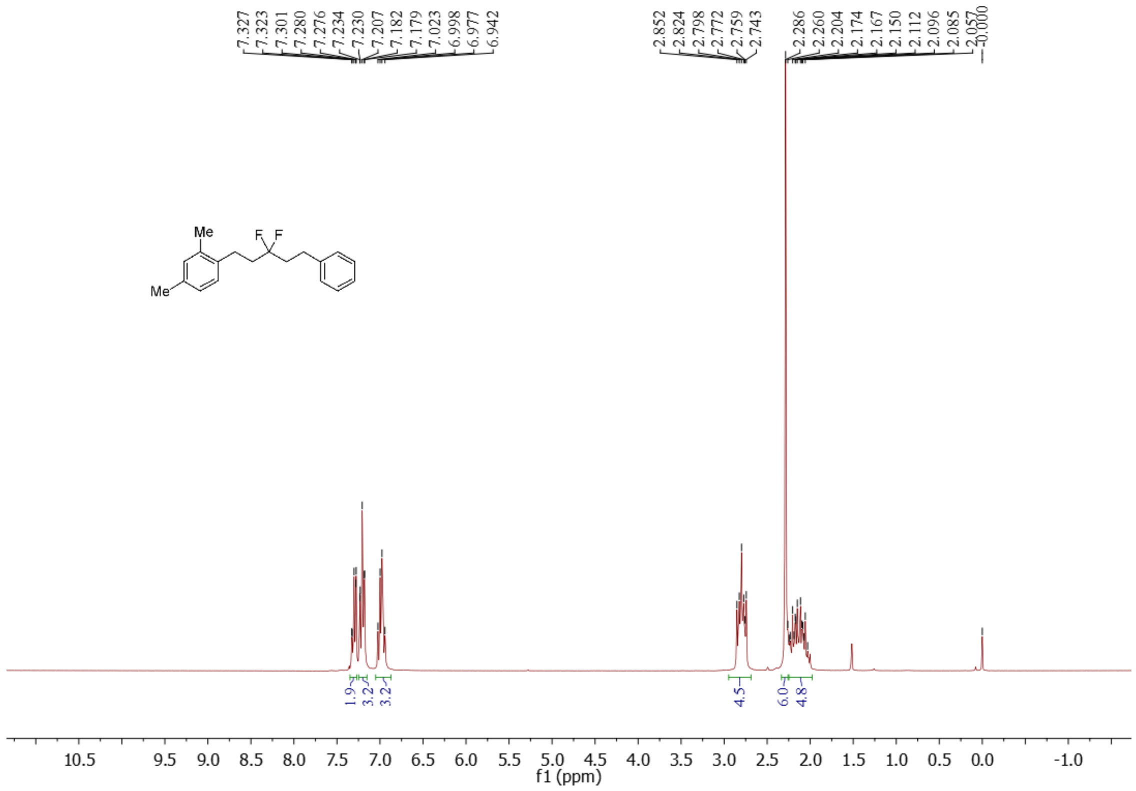

Figure S169. ^{13}C NMR spectrum of unknown *gem*-difluoride **1q**, related to **Figure 2**

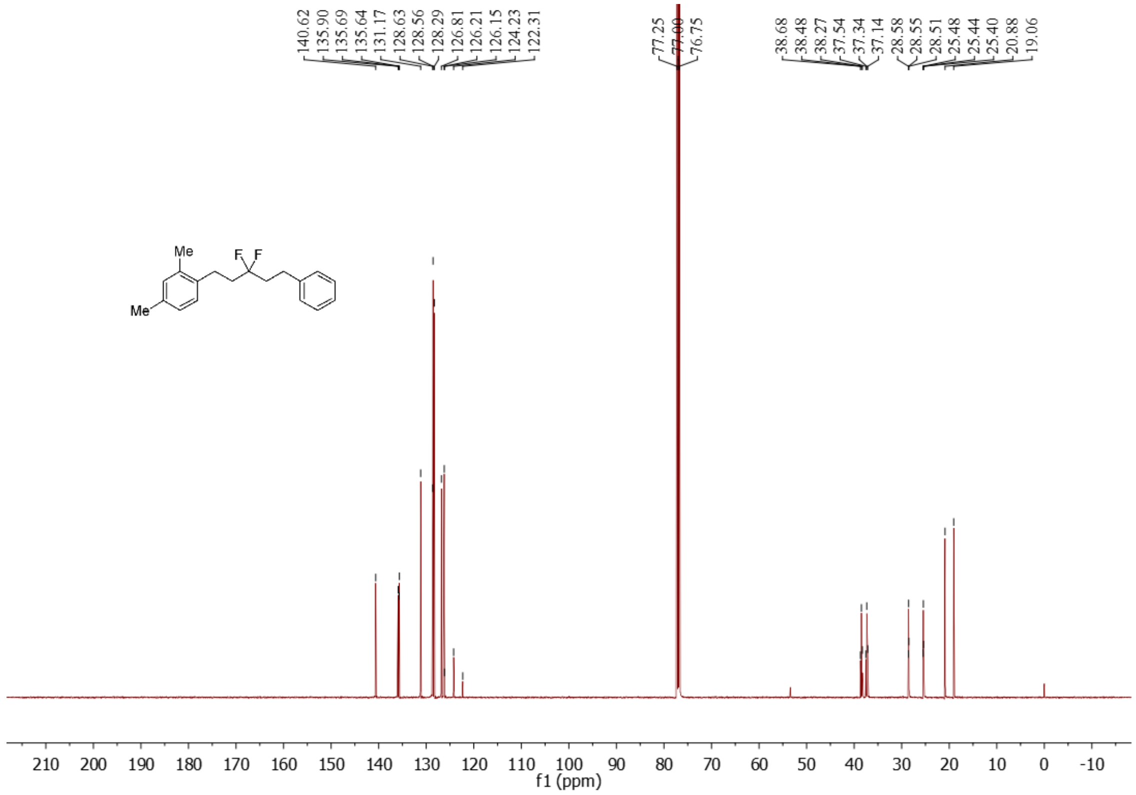

Figure S170. ^{19}F NMR spectrum of unknown *gem*-difluoride **1q**, related to **Figure 2**

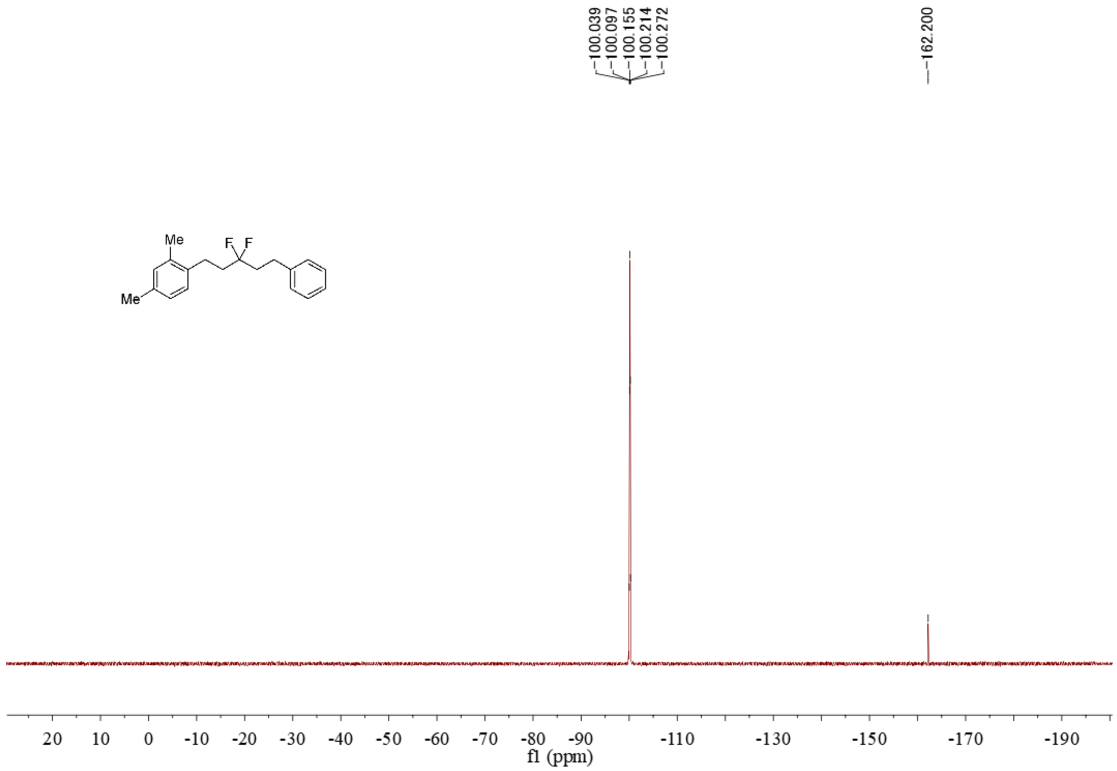

Figure S171. ¹H NMR spectrum of unknown *gem*-difluoride **1r**, related to **Figure 2**

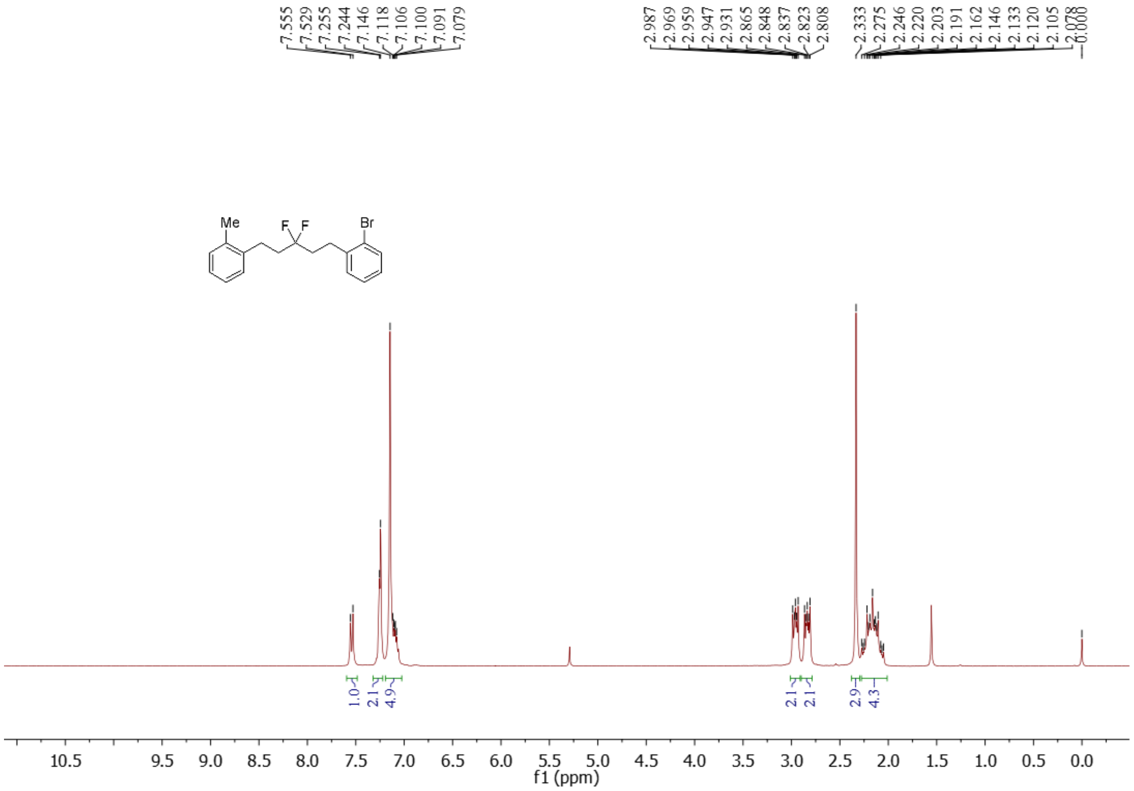

Figure S172. ¹³C NMR spectrum of unknown *gem*-difluoride **1r**, related to **Figure 2**

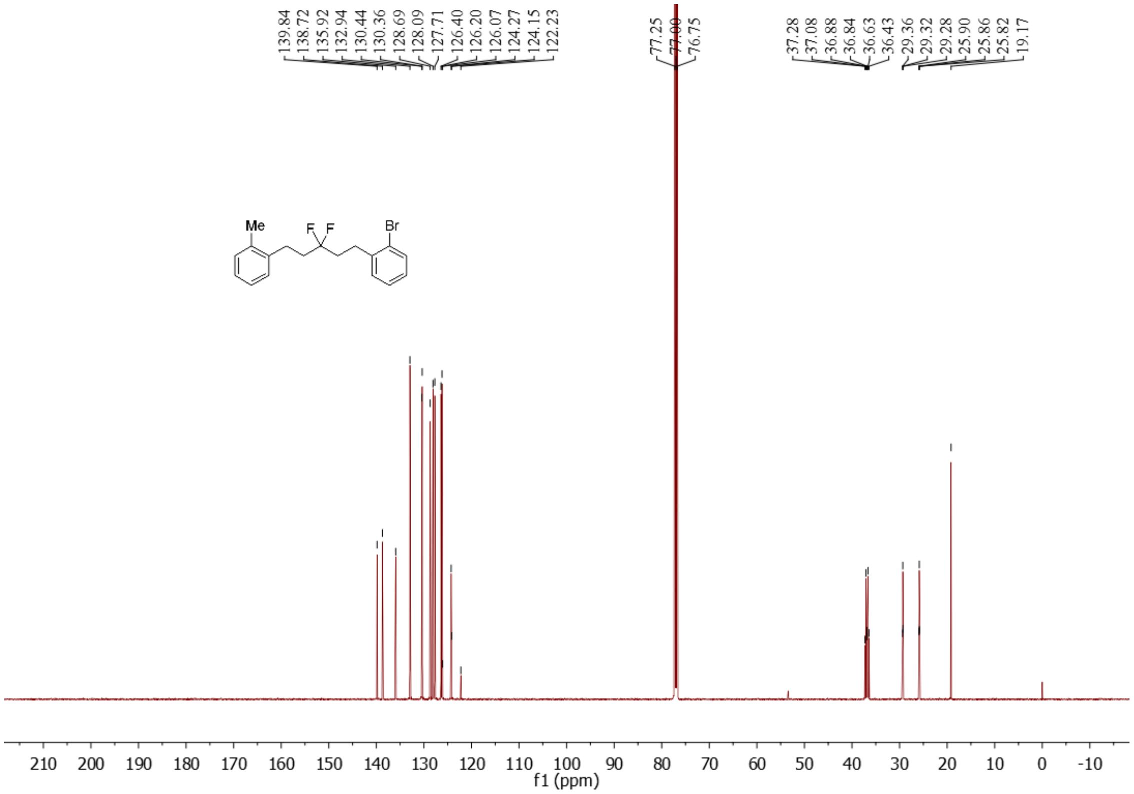

Figure S173. ^{19}F NMR spectrum of unknown *gem*-difluoride **1r**, related to **Figure 2**

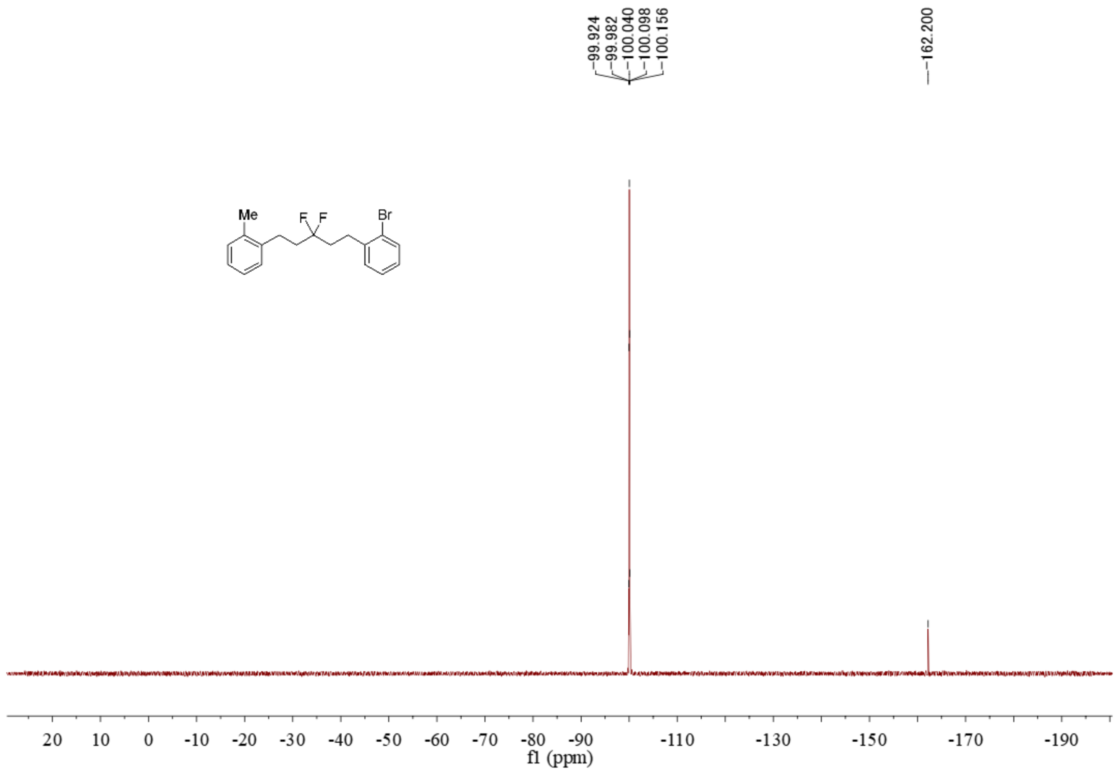

Figure S174. ^{1}H NMR spectrum of unknown *gem*-difluoride **1s**, related to **Figure 2**

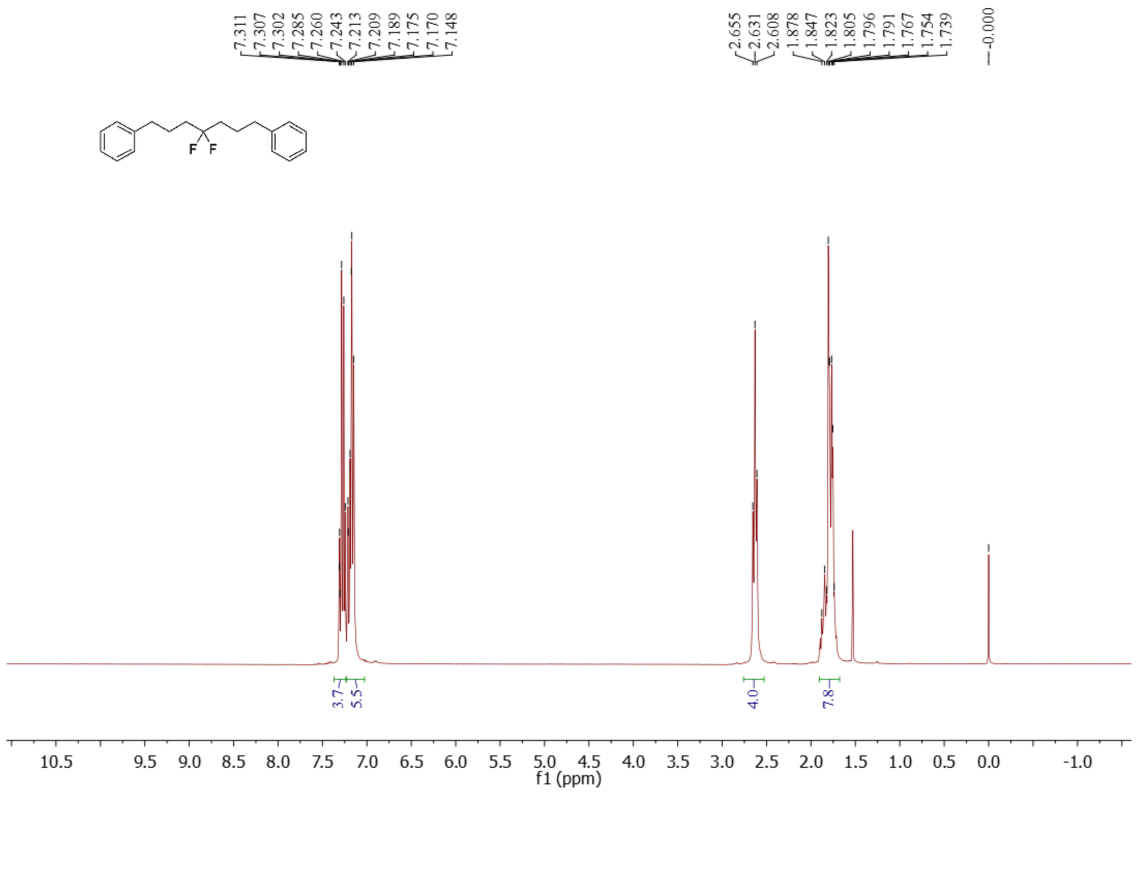

Figure S175. ^{13}C NMR spectrum of unknown *gem*-difluoride **1s**, related to **Figure 2**

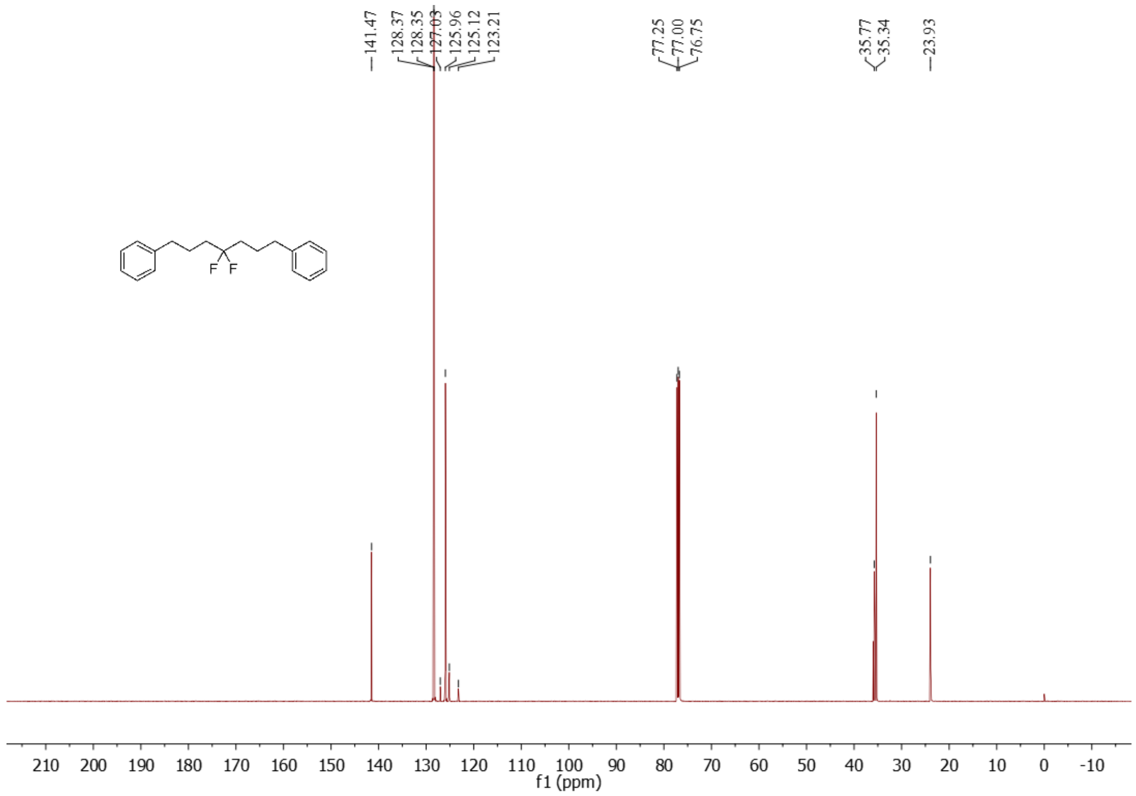

Figure S176. ^{19}F NMR spectrum of unknown *gem*-difluoride **1s**, related to **Figure 2**

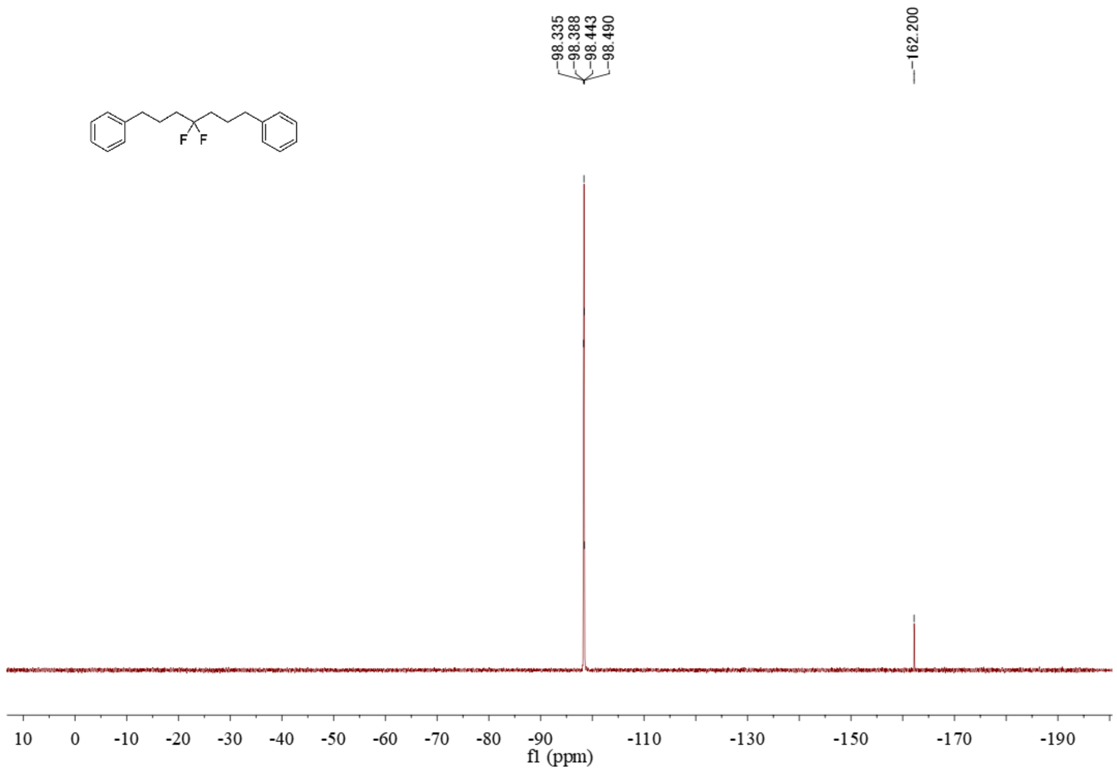

Figure S177. ^{1}H NMR spectrum of unknown *gem*-difluoride **1t**, related to **Figure 2**

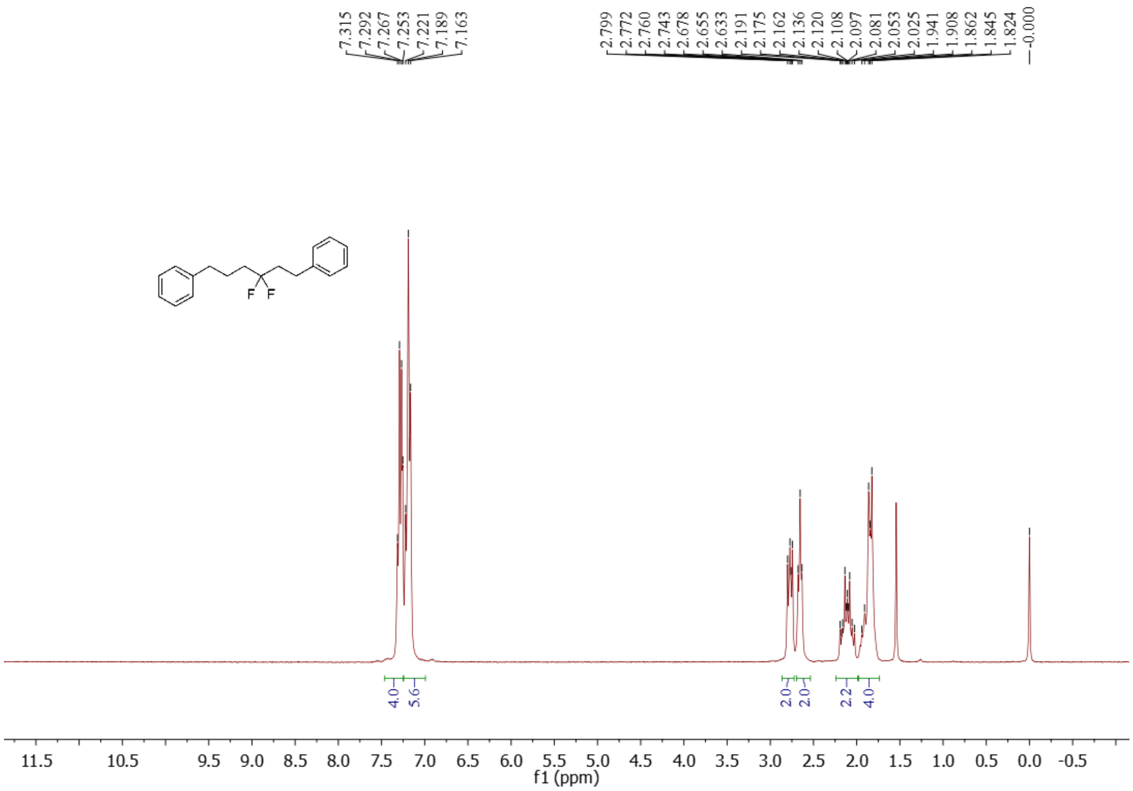

Figure S178. ^{13}C NMR spectrum of unknown *gem*-difluoride **1t**, related to **Figure 2**

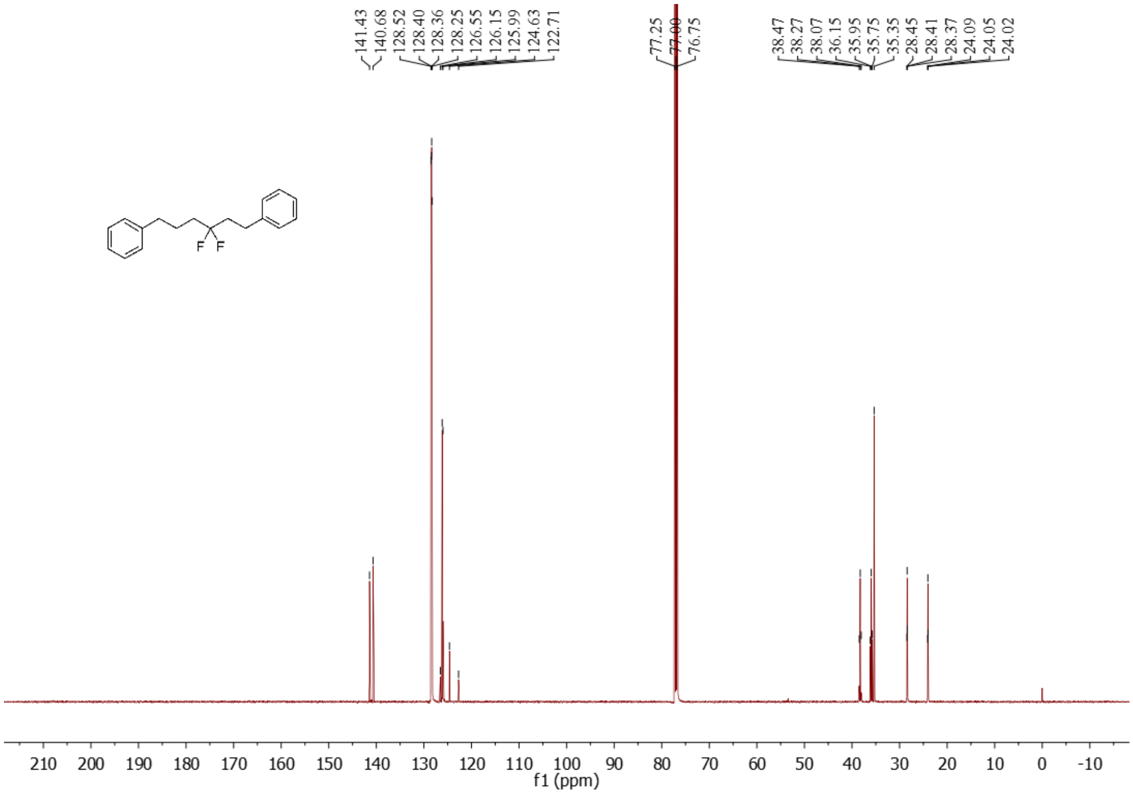

Figure S179. ^{19}F NMR spectrum of unknown *gem*-difluoride **1t**, related to **Figure 2**

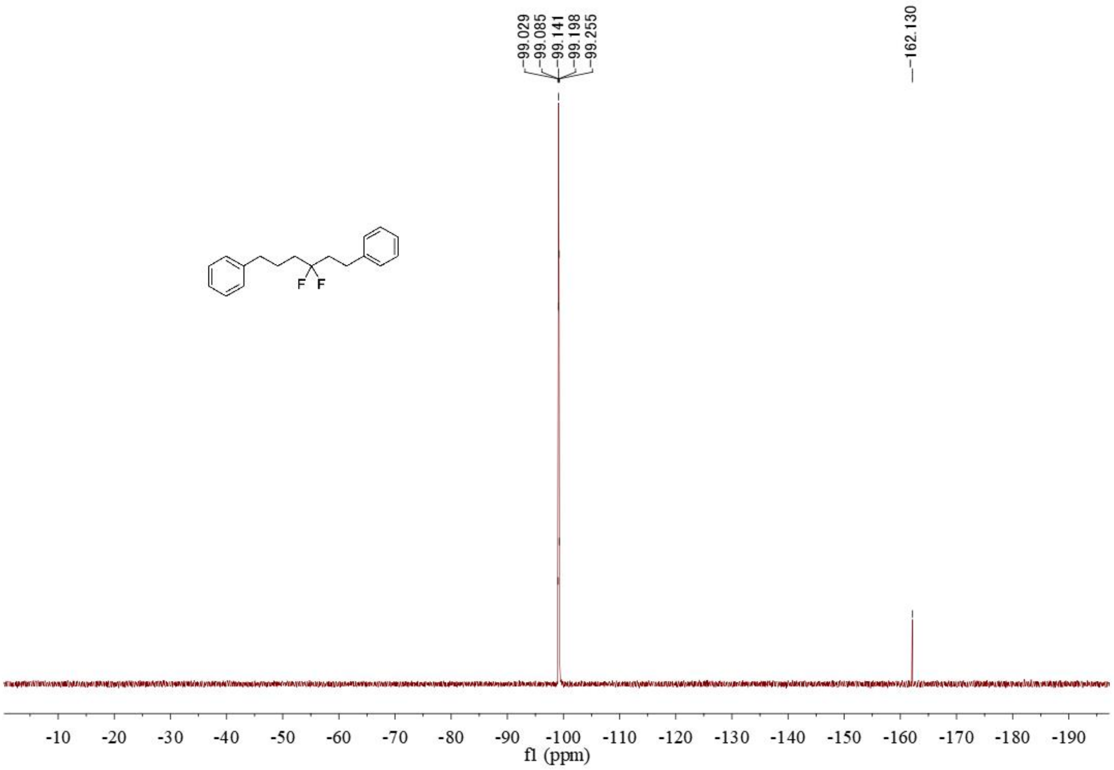

Figure S180. ^{1}H NMR spectrum of unknown *gem*-difluoride **1u**, related to **Figure 2**

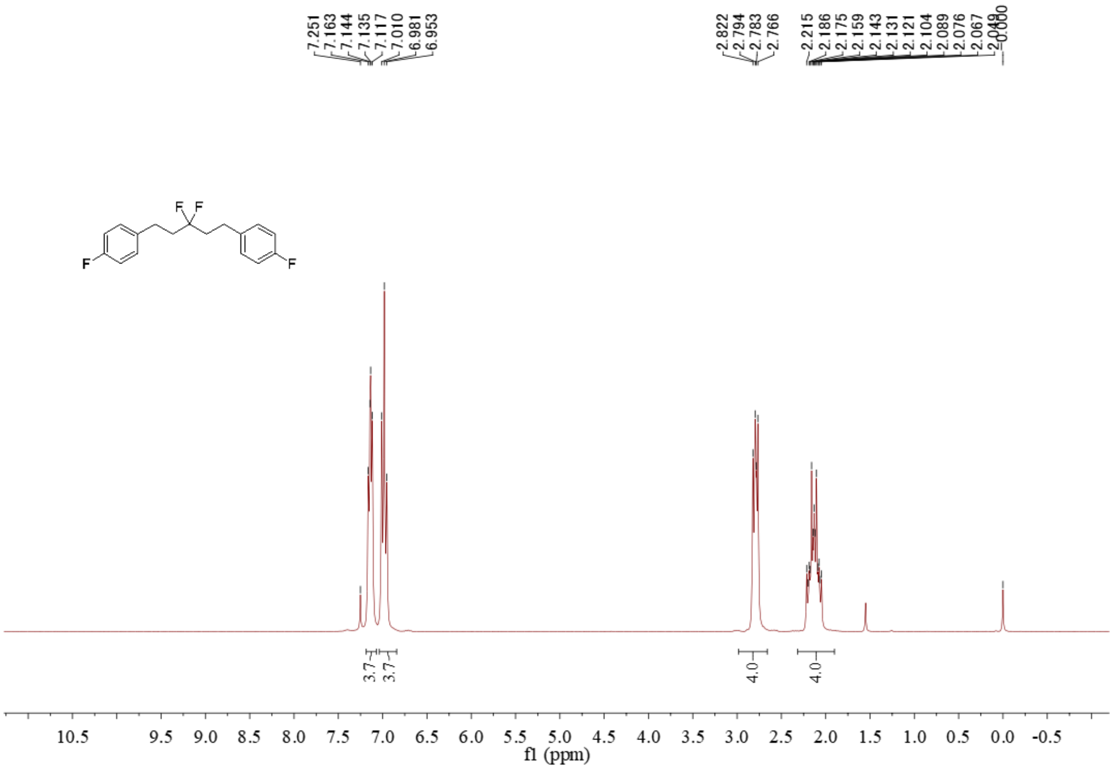

Figure S181. ^{13}C NMR spectrum of unknown *gem*-difluoride **1u**, related to **Figure 2**

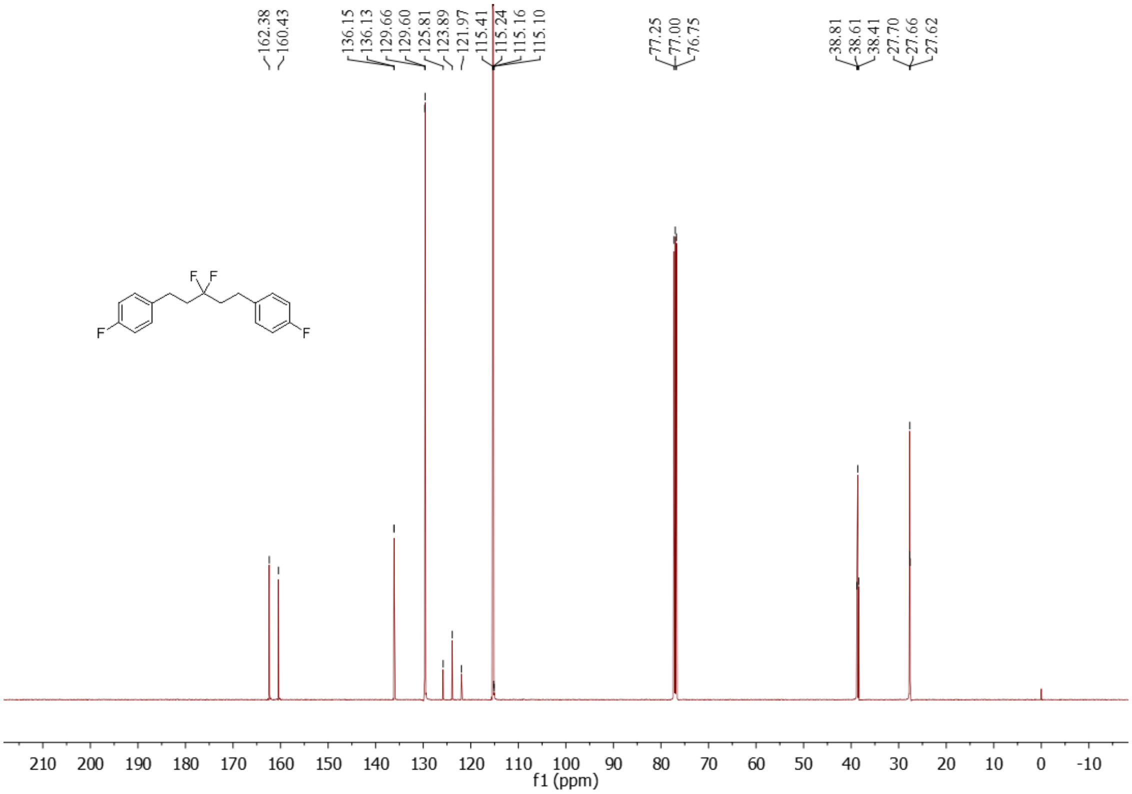

Figure S182. ^{19}F NMR spectrum of unknown *gem*-difluoride **1u**, related to **Figure 2**

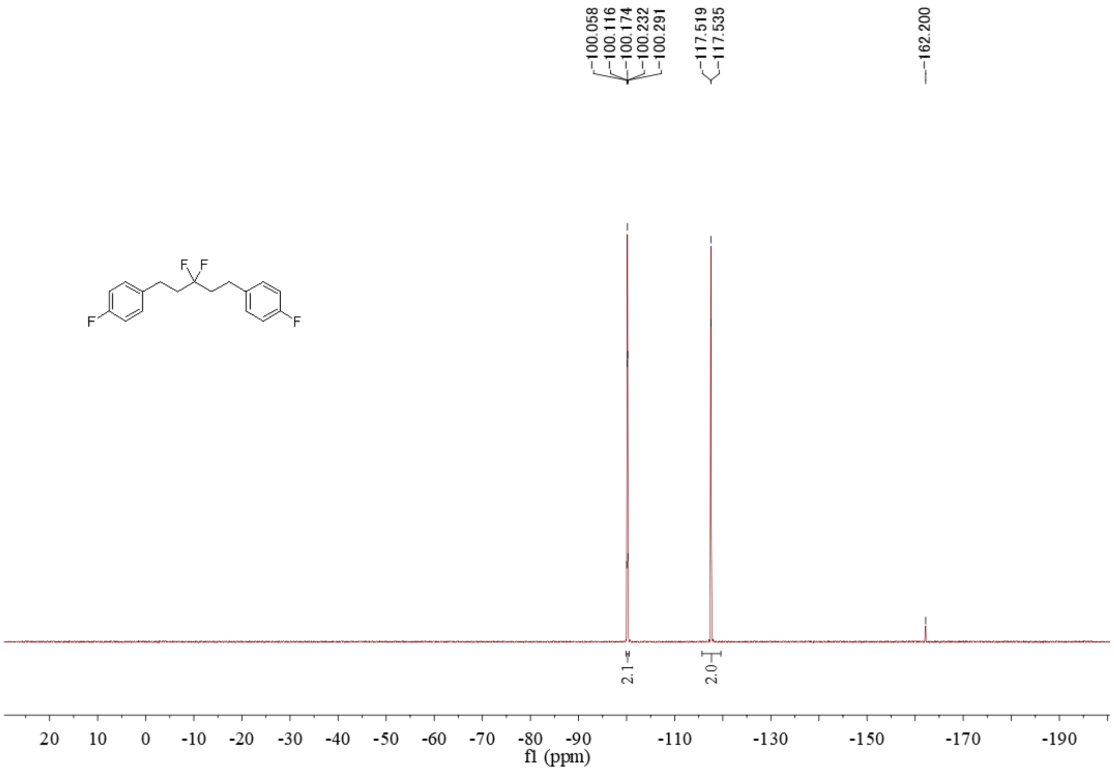

Figure S183. ^{1}H NMR spectrum of unknown *gem*-difluoride **1cc**, related to **Figure 2**

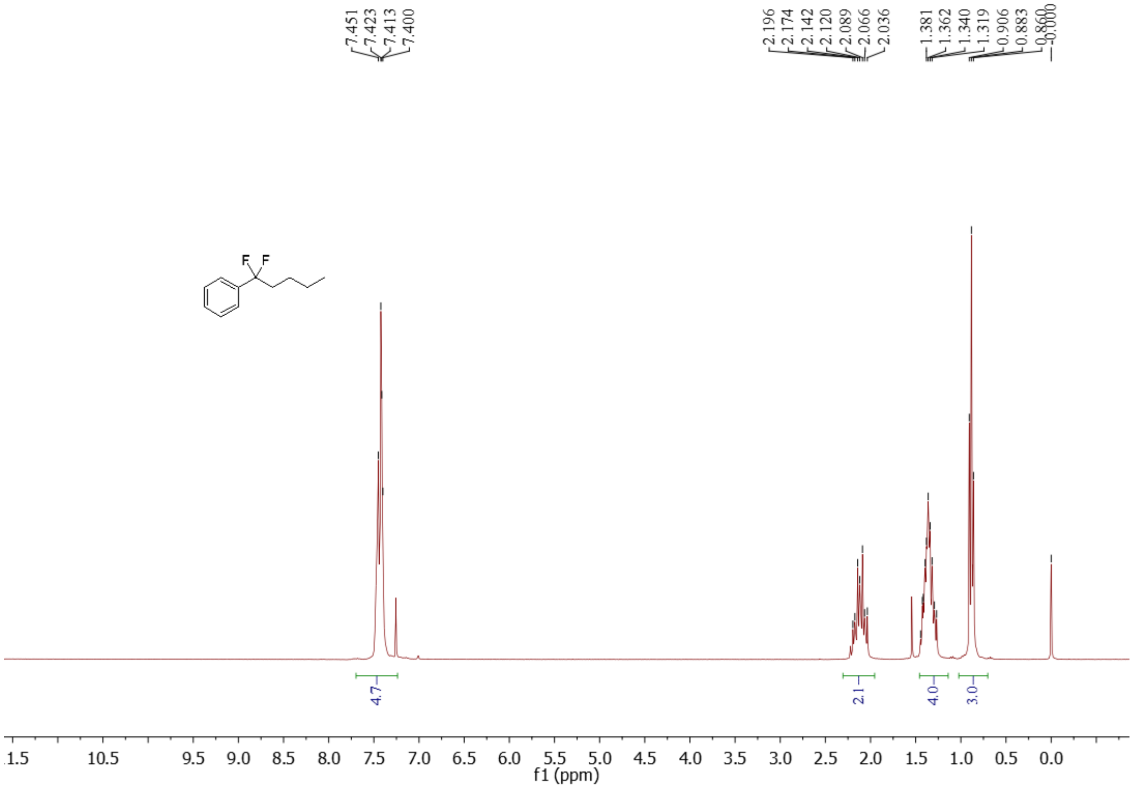

Figure S184. ^{13}C NMR spectrum of unknown *gem*-difluoride **1cc**, related to **Figure 2**

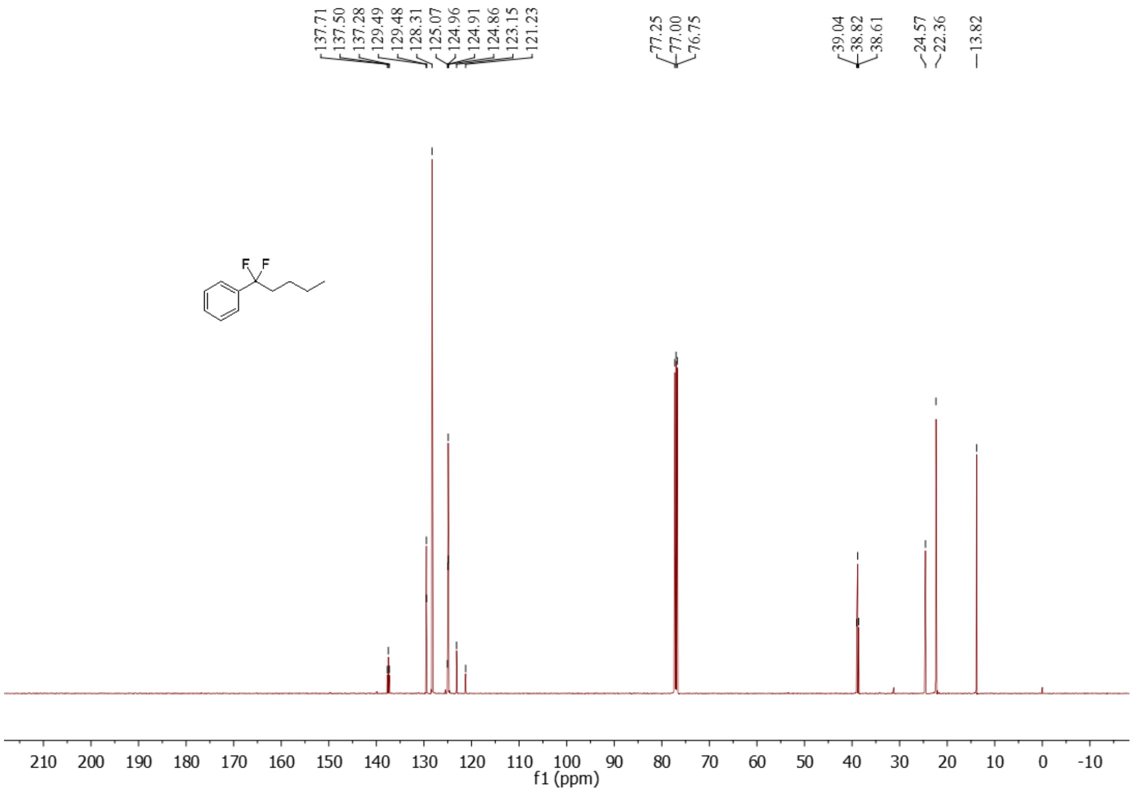

Figure S185. ^{19}F NMR spectrum of unknown *gem*-difluoride **1cc**, related to **Figure 2**

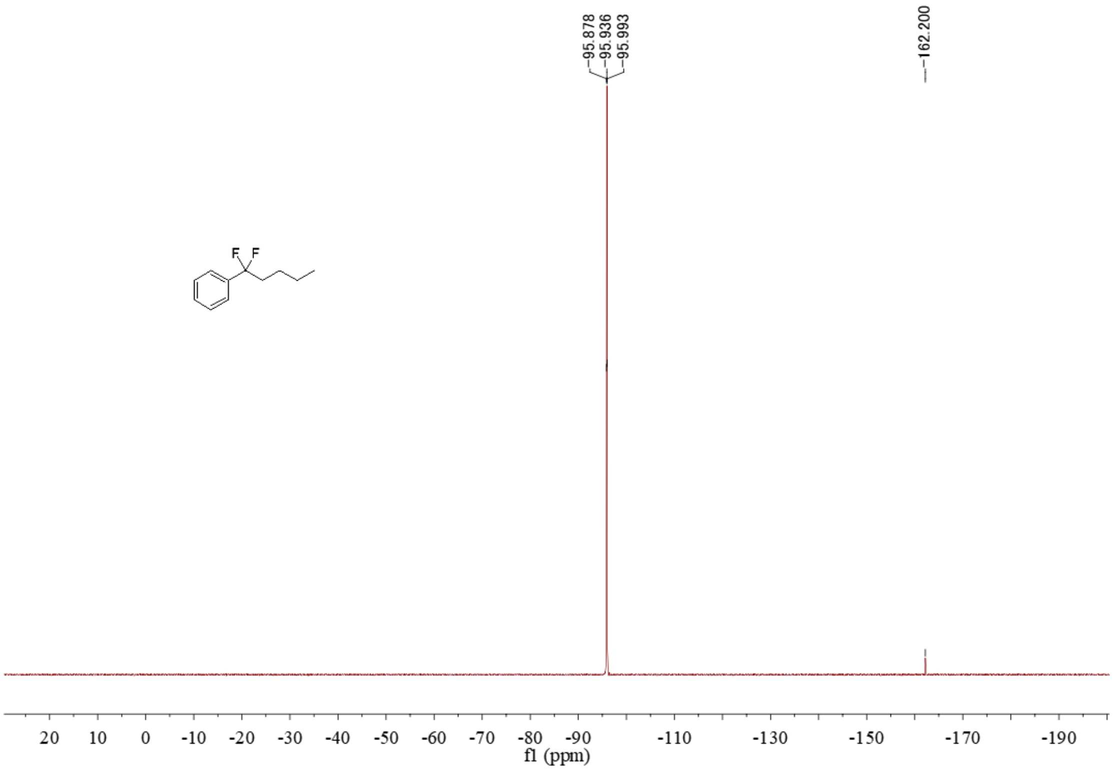

Figure S186. ^{1}H NMR spectrum of unknown *gem*-difluoride **1ee**, related to **Figure 2**

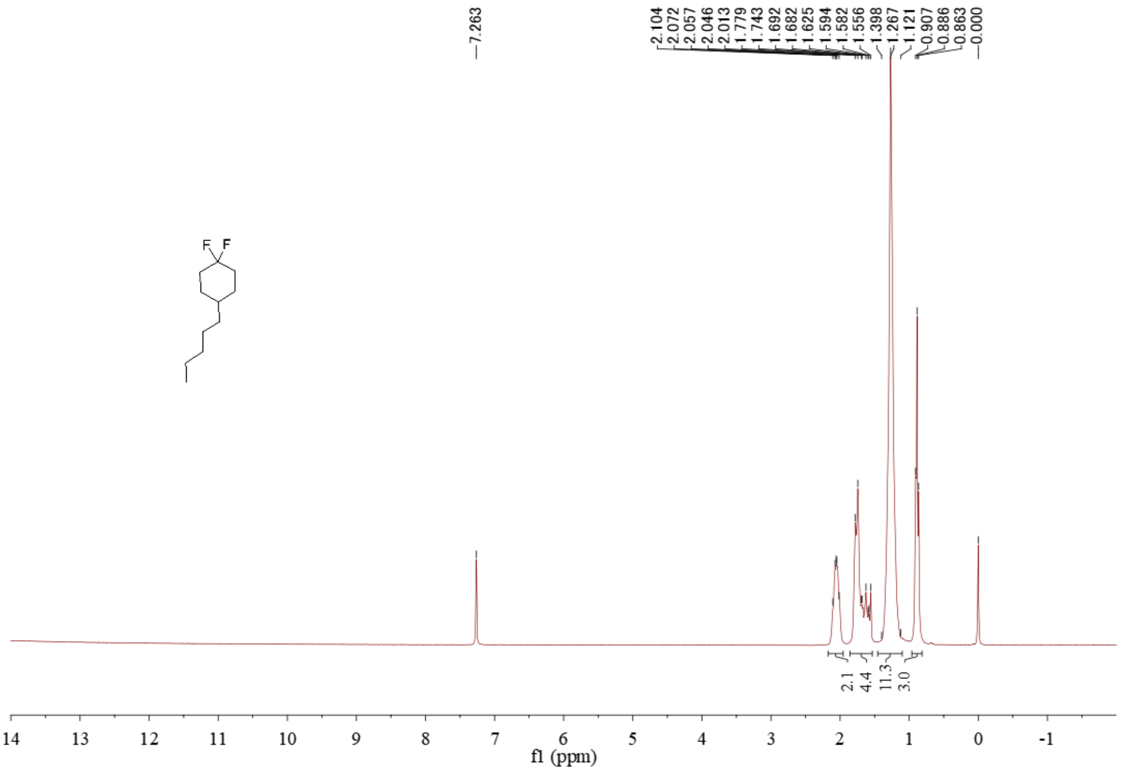

Figure S187. ^{13}C NMR spectrum of unknown *gem*-difluoride **1ee**, related to **Figure 2**

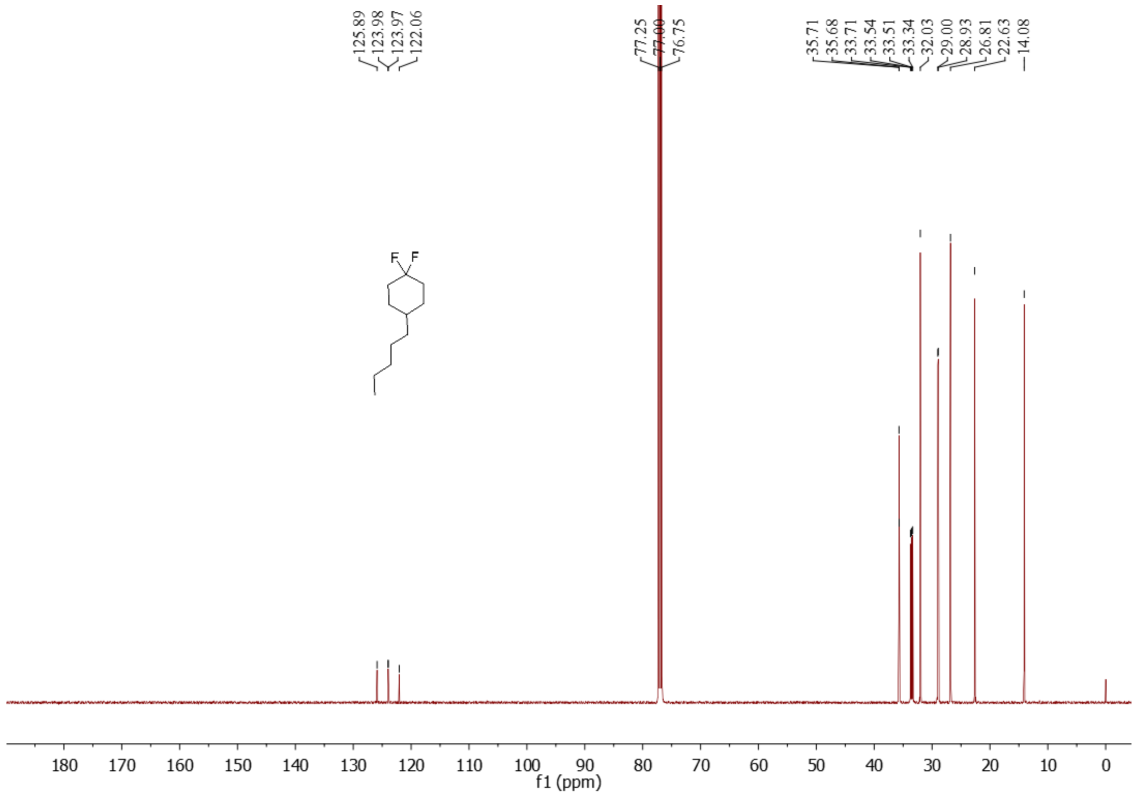

Figure S188. ^{19}F NMR spectrum of unknown *gem*-difluoride **1ee**, related to **Figure 2**

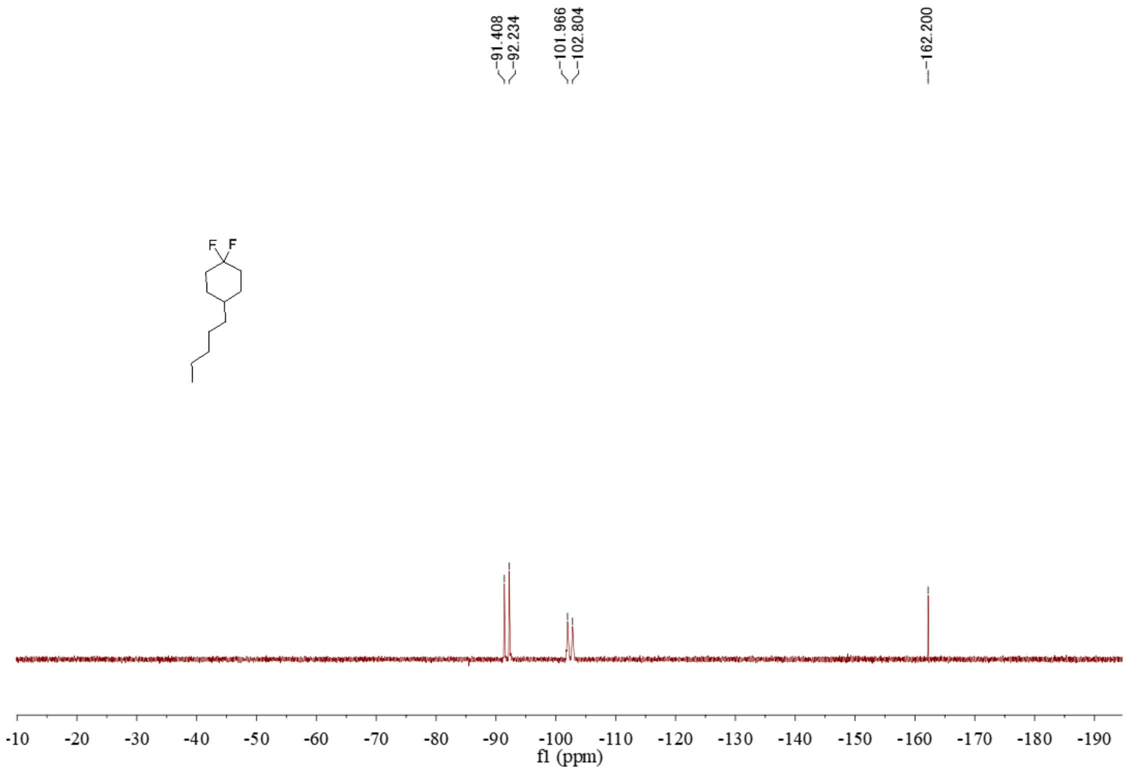

Figure S189. ^{1}H NMR spectrum of unknown *gem*-difluoride **1hh**, related to **Figure 2**

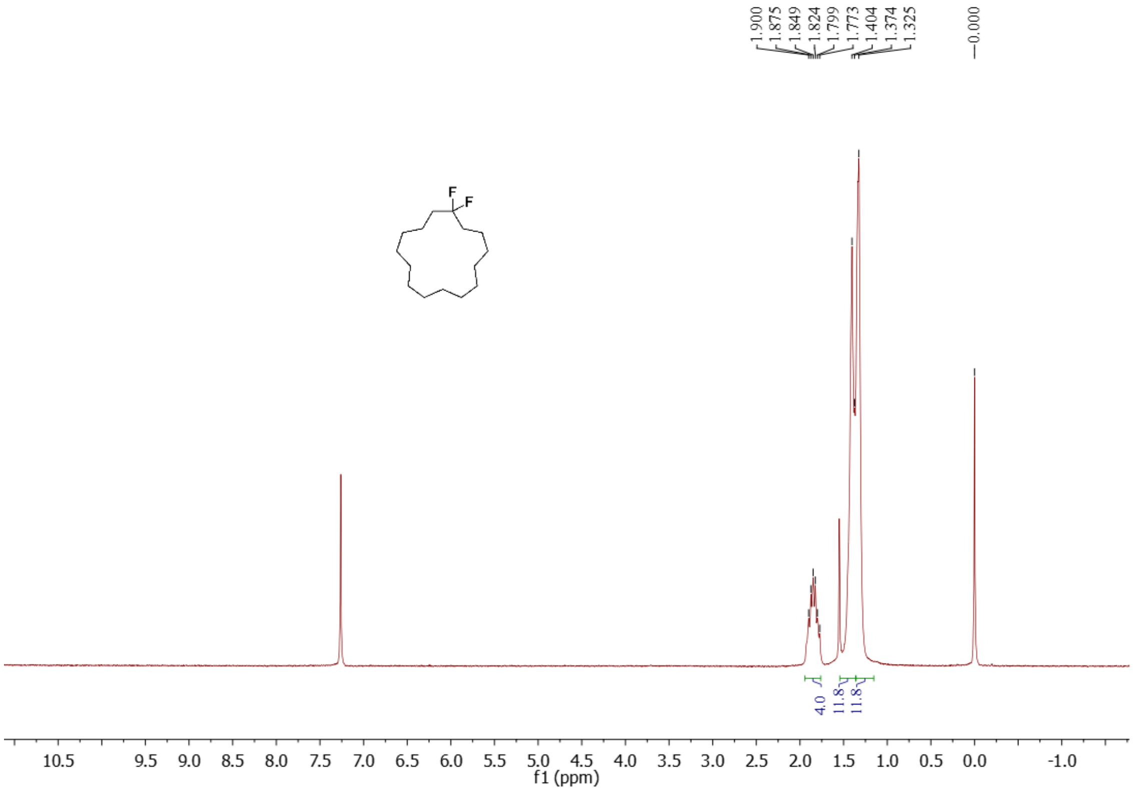

Figure S190. ^{13}C NMR spectrum of unknown *gem*-difluoride **1hh**, related to **Figure 2**

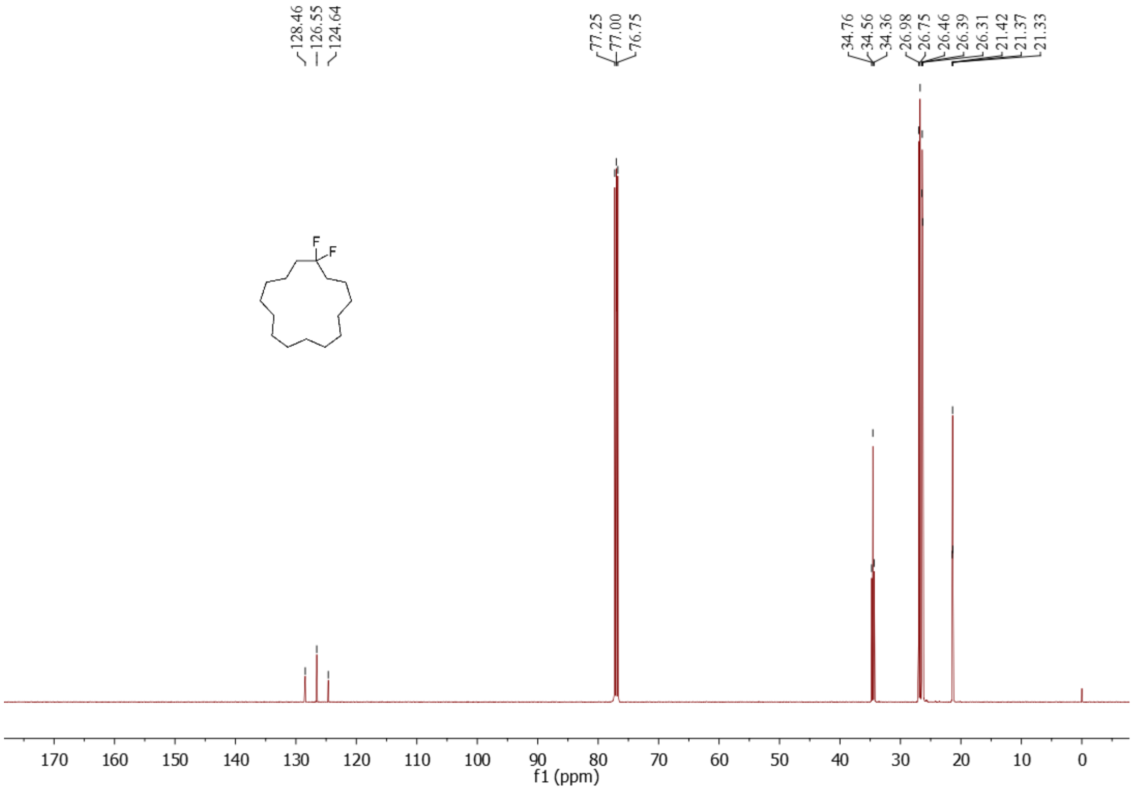

Figure S191. ^{19}F NMR spectrum of unknown *gem*-difluoride **1hh**, related to **Figure 2**

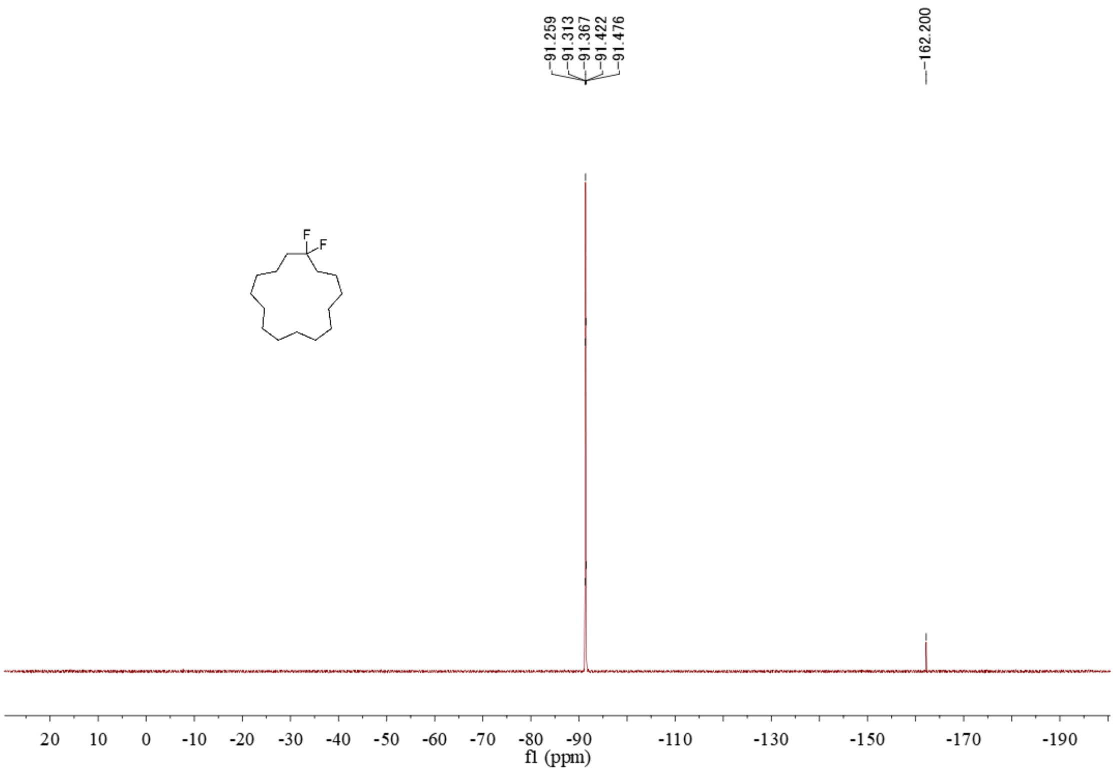

Figure S192. ^{1}H NMR spectrum of unknown secondary monofluoride **4a**, related to **Figure 3**

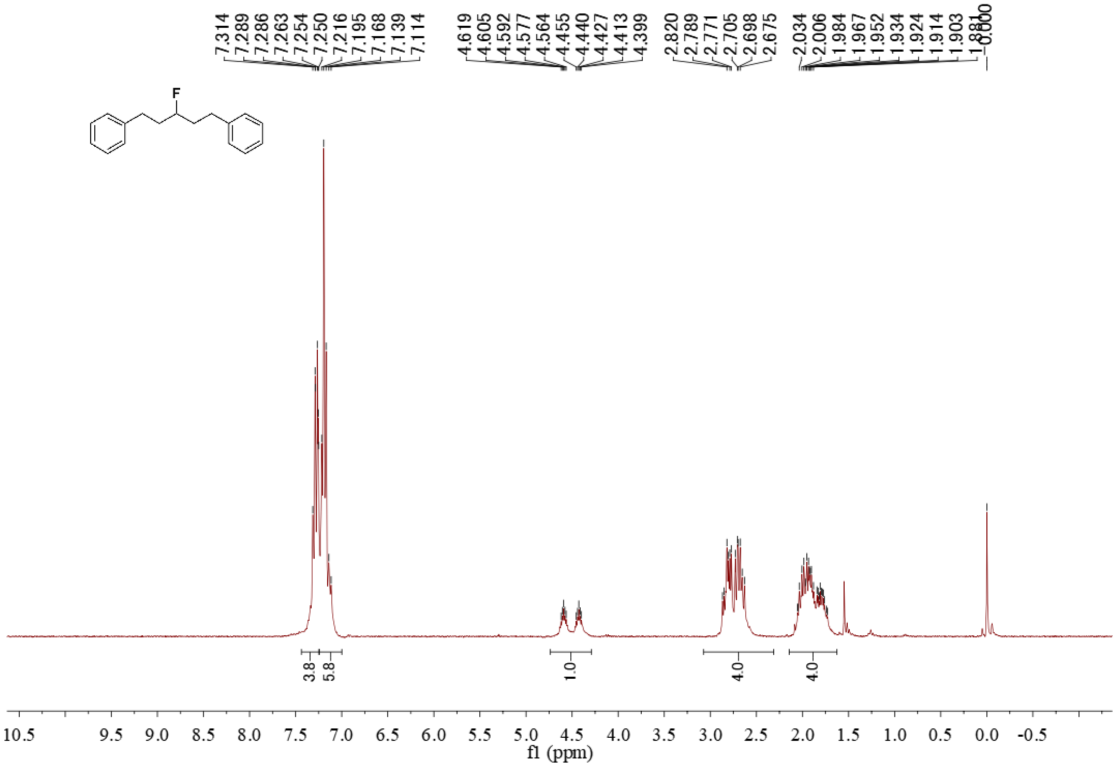

Figure S193. ^{13}C NMR spectrum of unknown secondary monofluoride **4a**, related to **Figure 3**

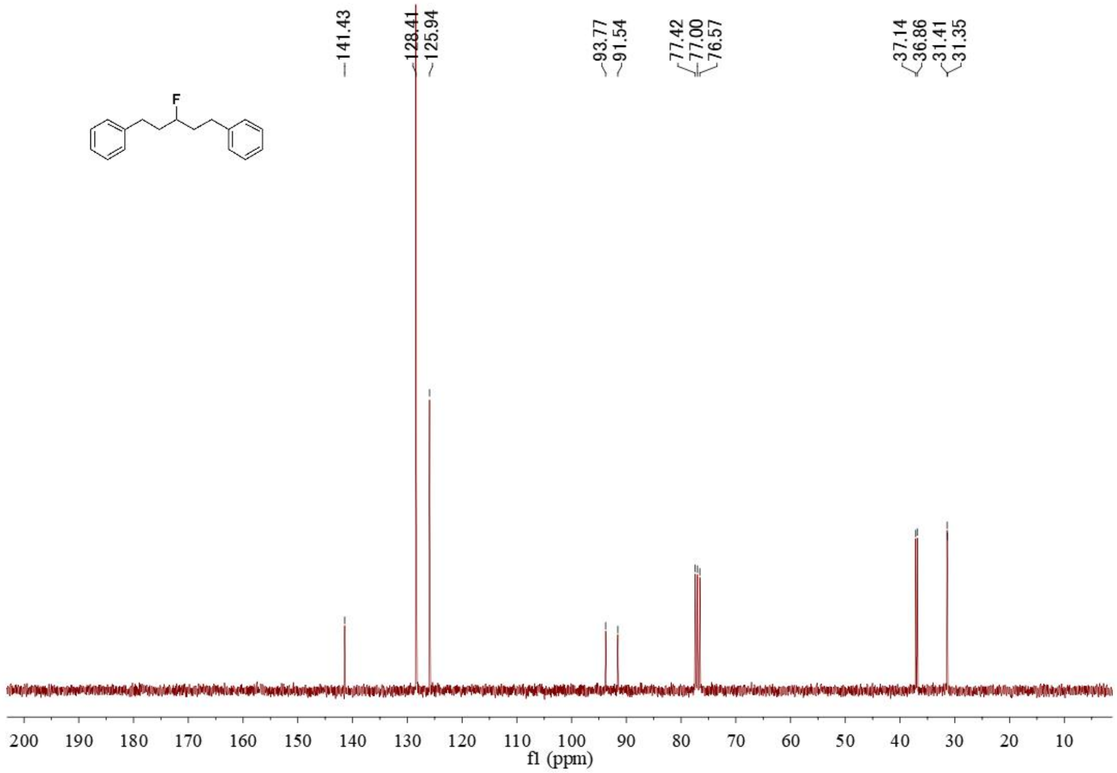

Figure S194. ^{19}F NMR spectrum of unknown secondary monofluoride **4a**, related to **Figure 3**

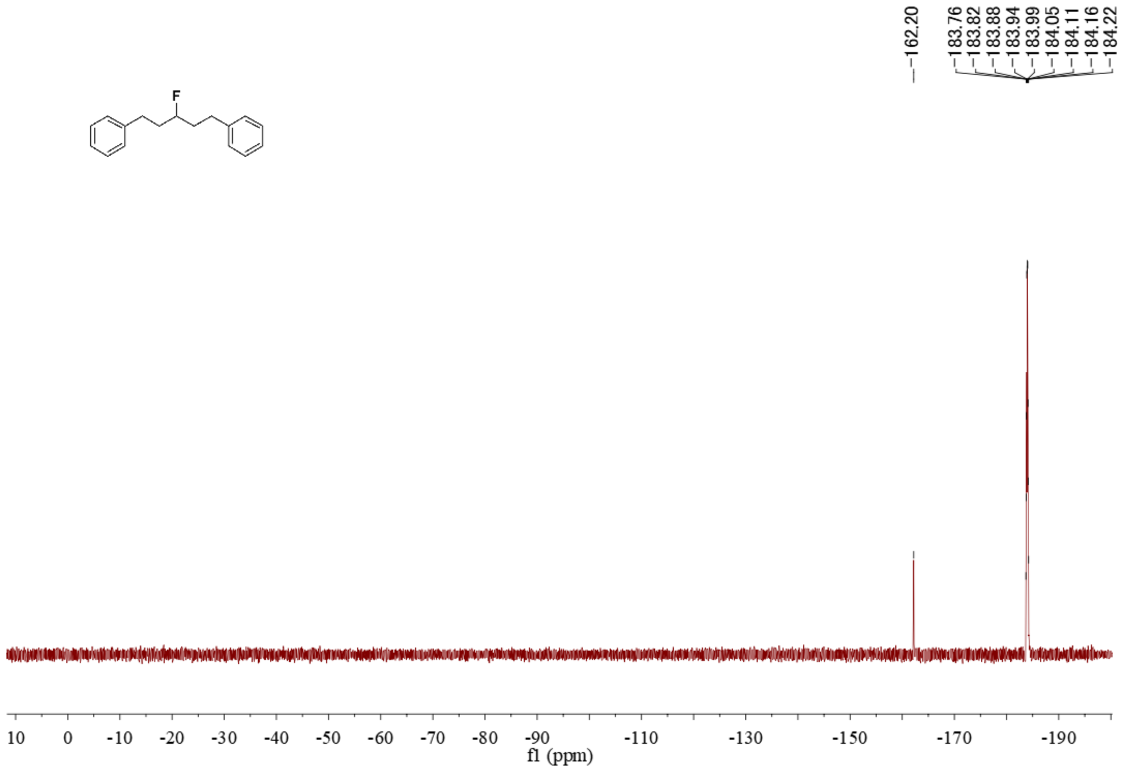

Figure S195. ^{1}H NMR spectrum of unknown secondary monofluoride **4b**, related to **Figure 3**

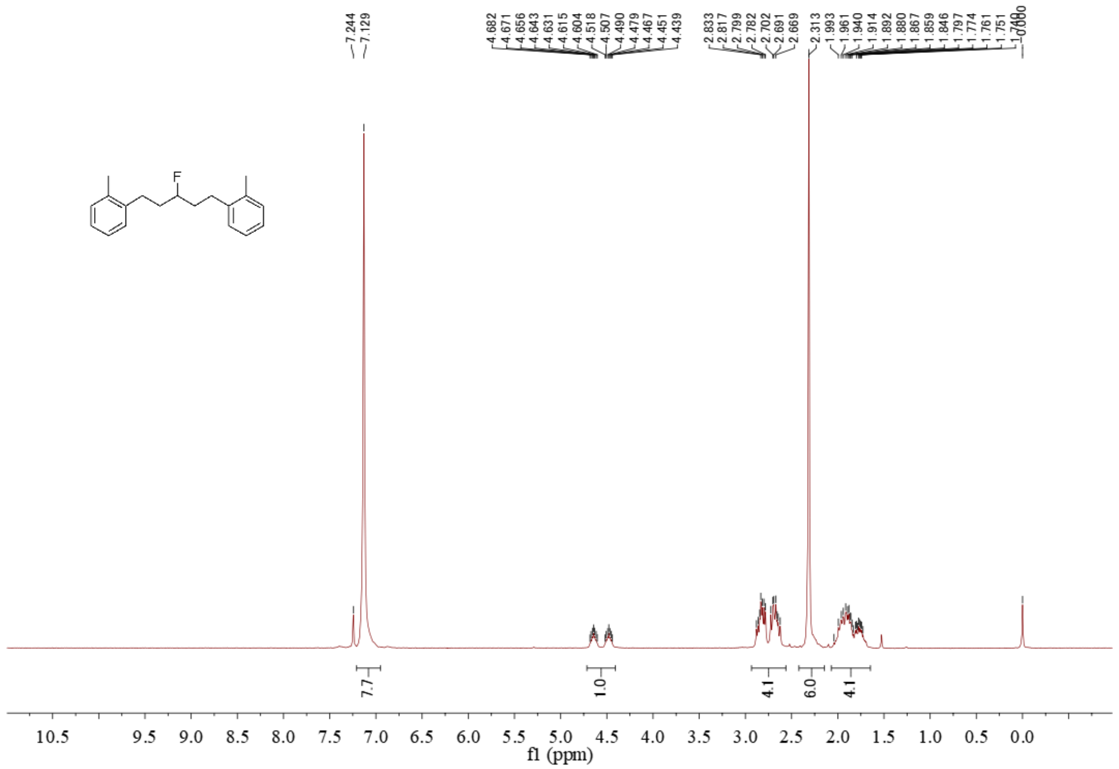

Figure S196. ^{13}C NMR spectrum of unknown secondary monofluoride **4b**, related to **Figure 3**

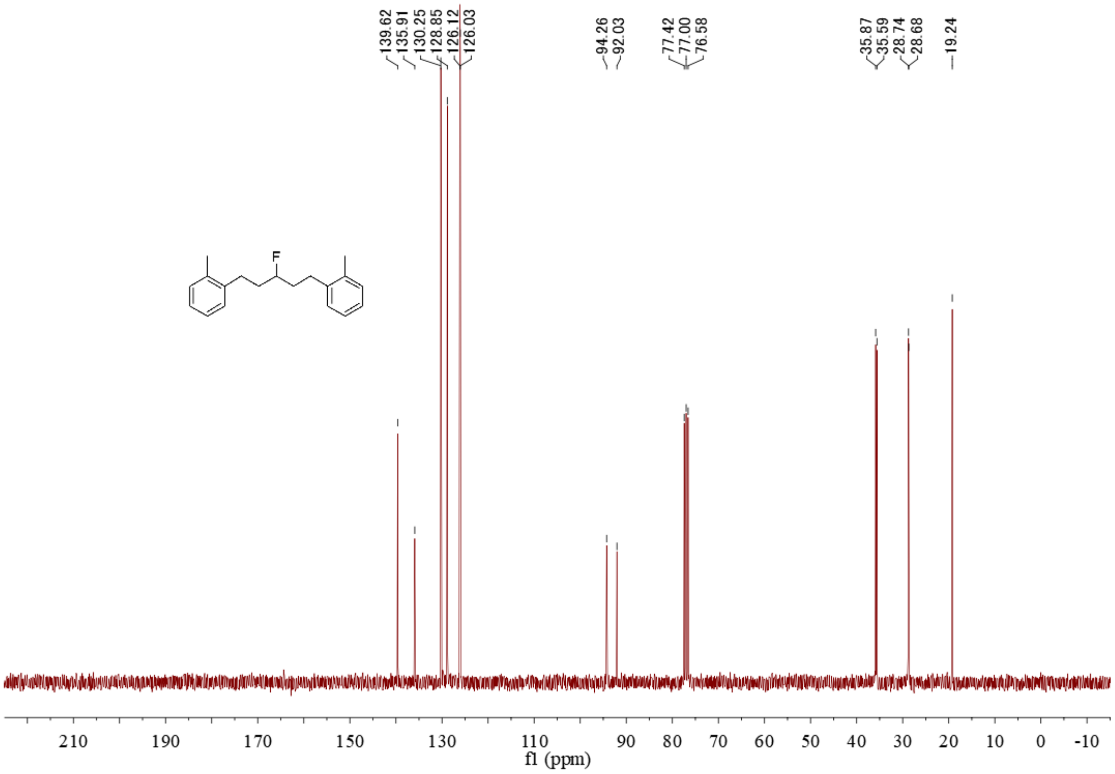

Figure S197. ^{19}F NMR spectrum of unknown secondary monofluoride **4b**, related to **Figure 3**

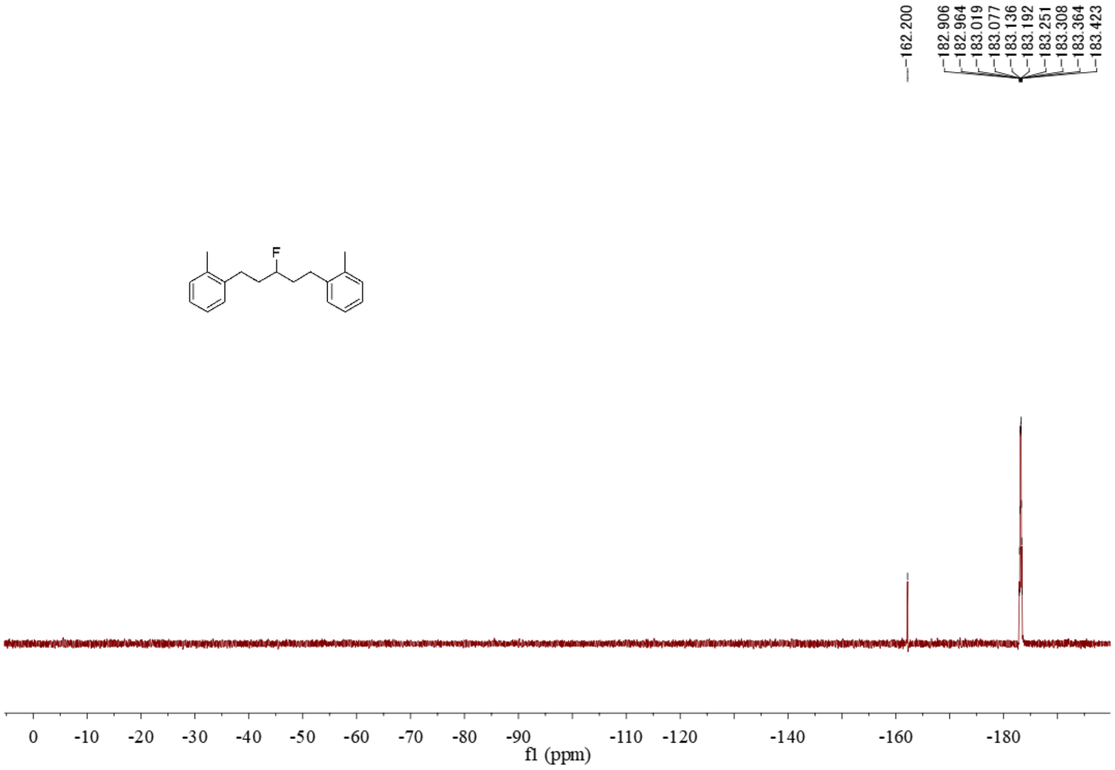

Figure S198. ^{1}H NMR spectrum of unknown secondary monofluoride **4c**, related to **Figure 3**

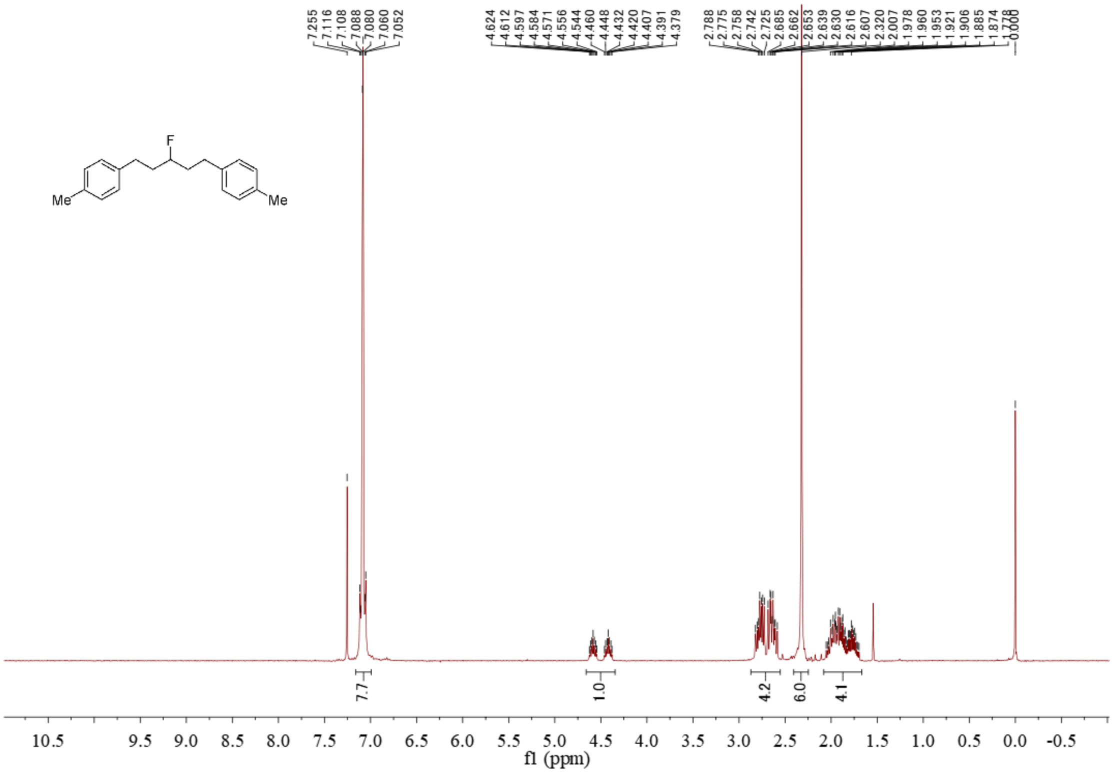

Figure S199. ^{13}C NMR spectrum of unknown secondary monofluoride **4c**, related to **Figure 3**

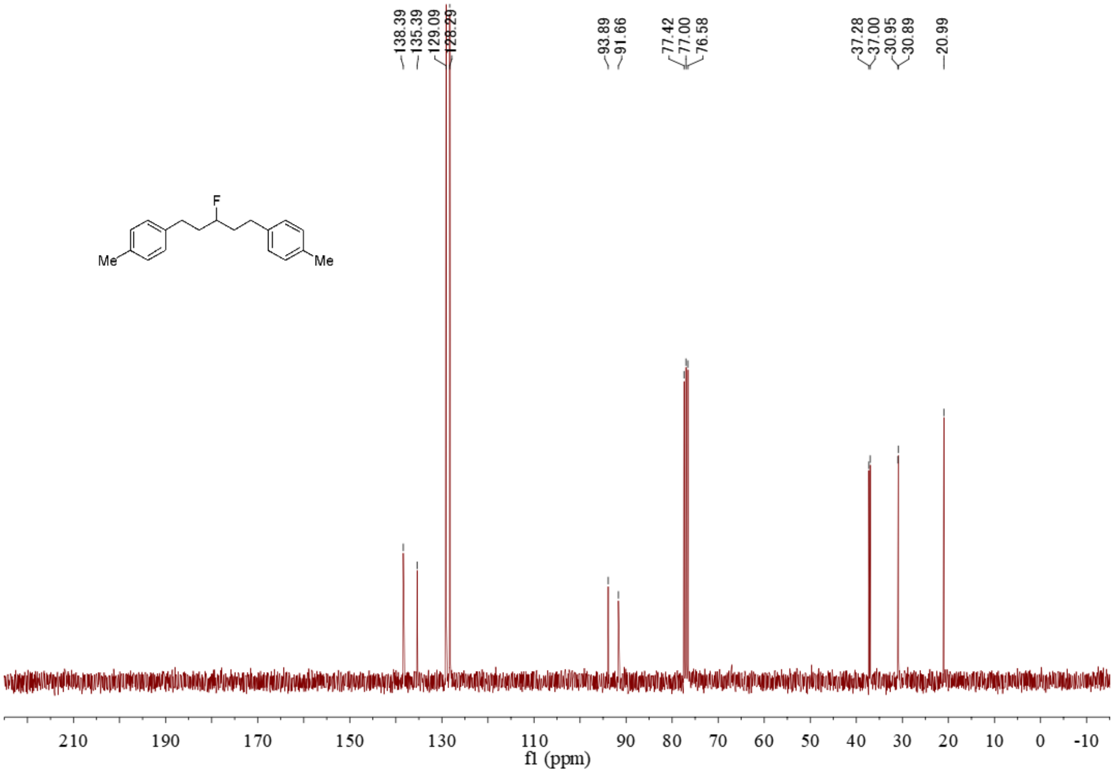

Figure S200. ^{19}F NMR spectrum of unknown secondary monofluoride **4c**, related to **Figure 3**

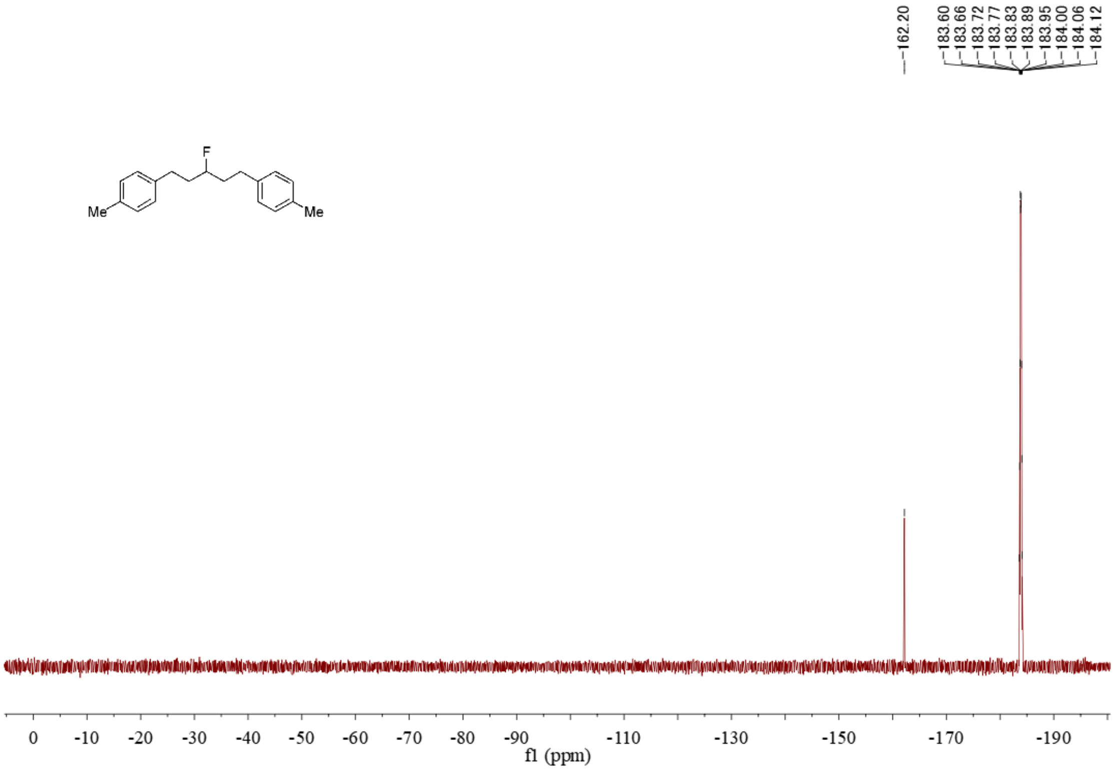

Figure S201. ^{1}H NMR spectrum of unknown secondary monofluoride **4d**, related to **Figure 3**

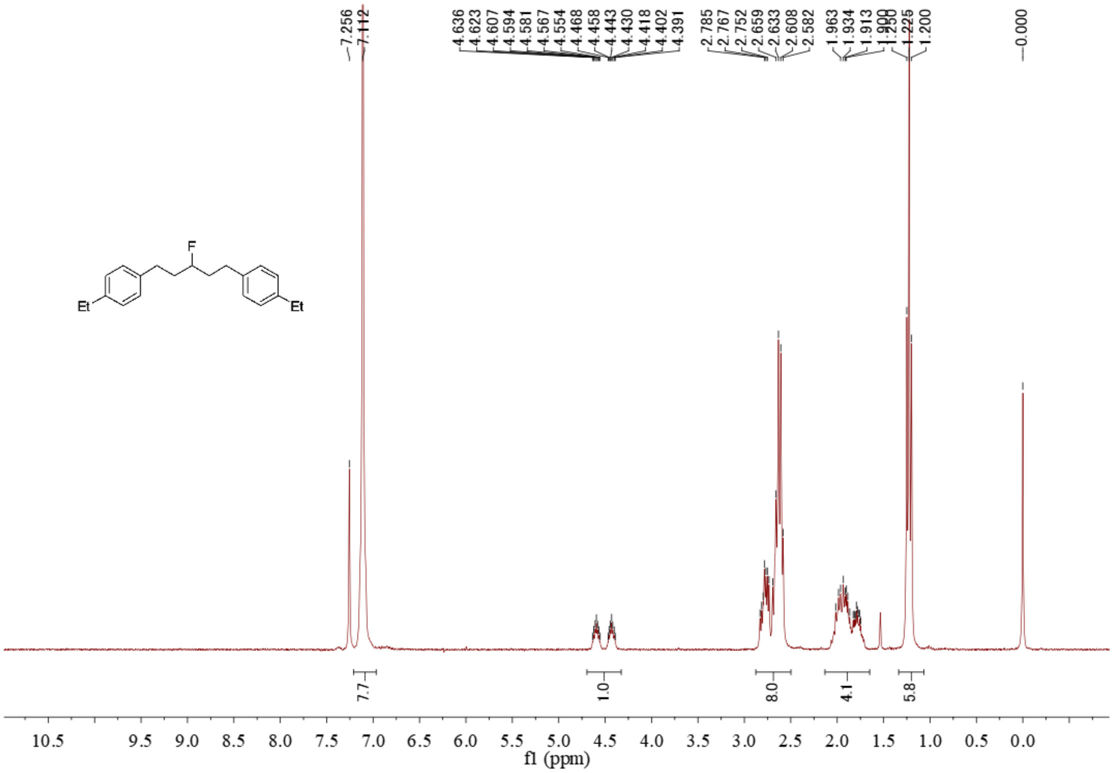

Figure S202. ^{13}C NMR spectrum of unknown secondary monofluoride **4d**, related to **Figure 3**

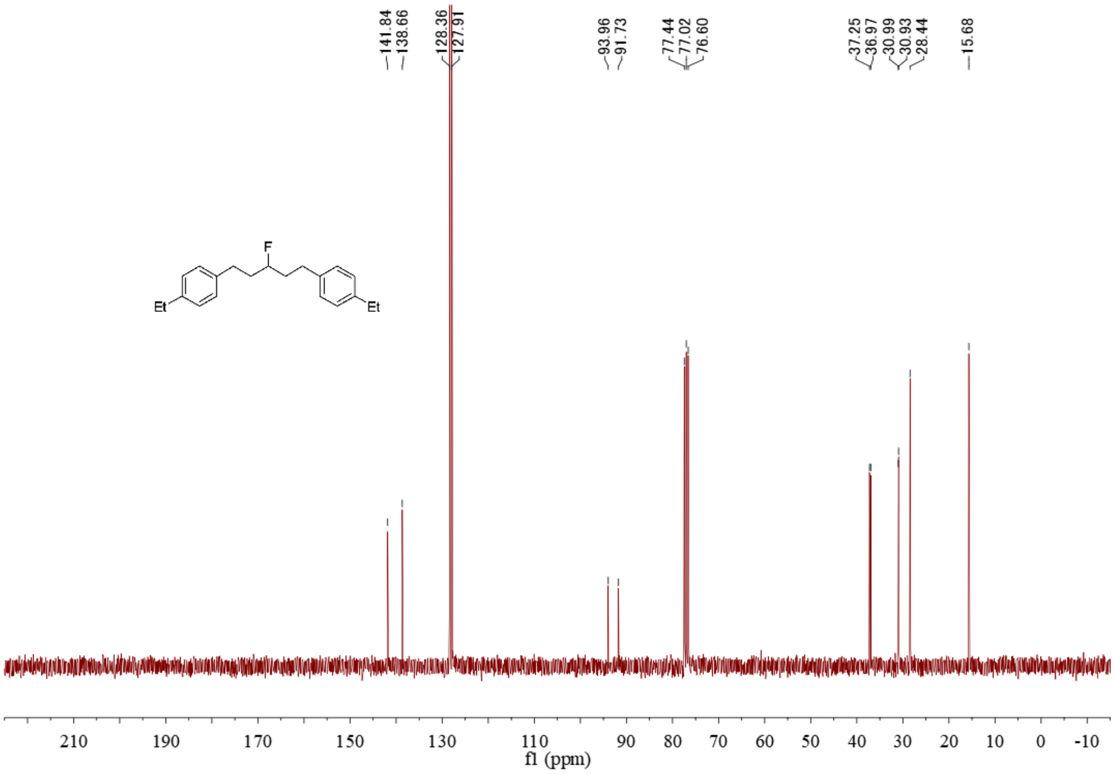

Figure S203. ^{19}F NMR spectrum of unknown secondary monofluoride **4d**, related to **Figure 3**

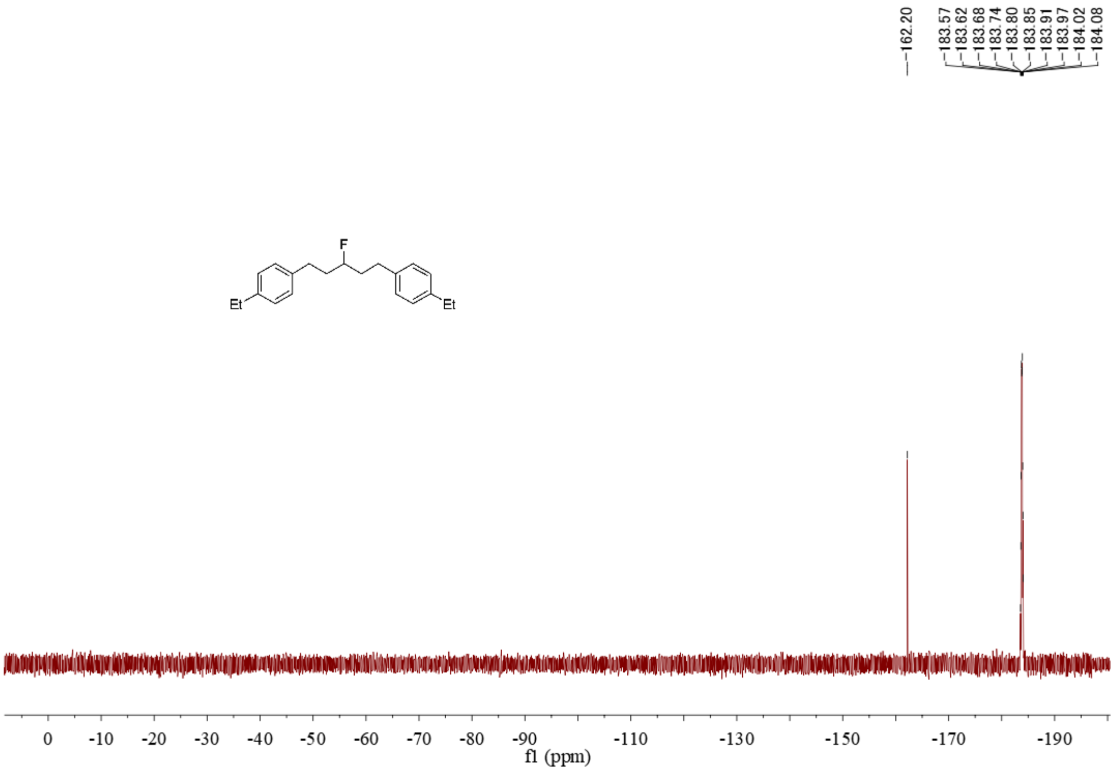

Figure S204. ^{1}H NMR spectrum of unknown secondary monofluoride **4e**, related to **Figure 3**

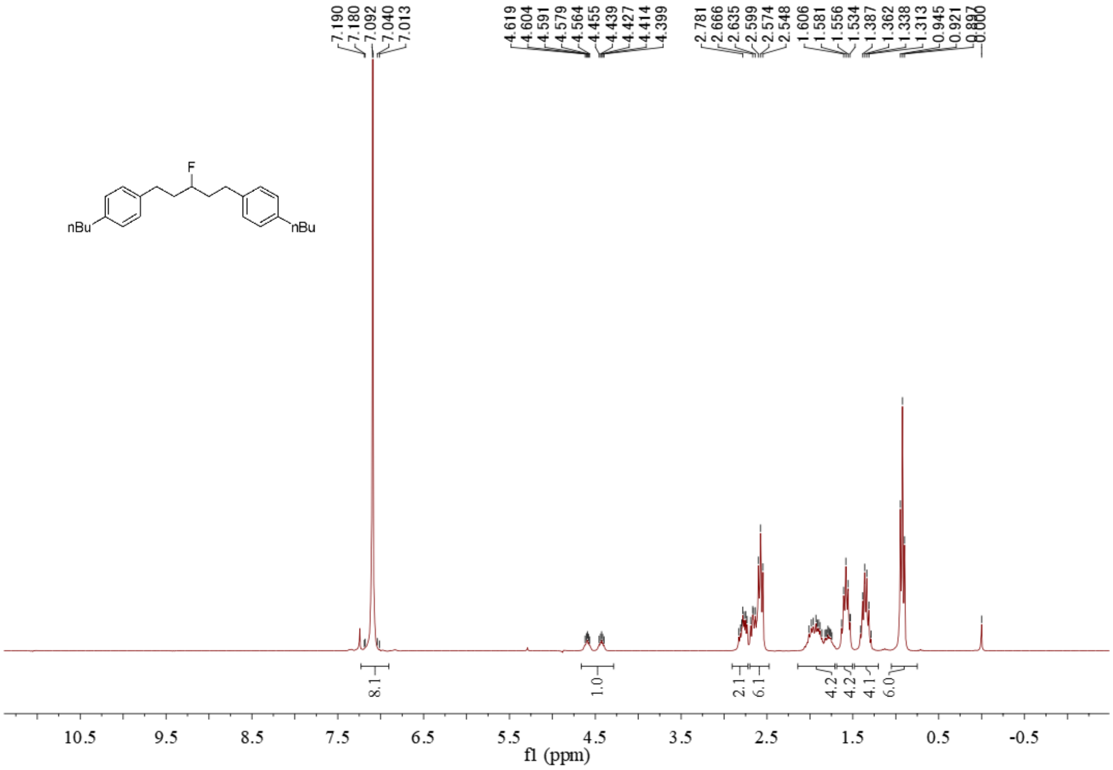

Figure S205. ^{13}C NMR spectrum of unknown secondary monofluoride **4e**, related to **Figure 3**

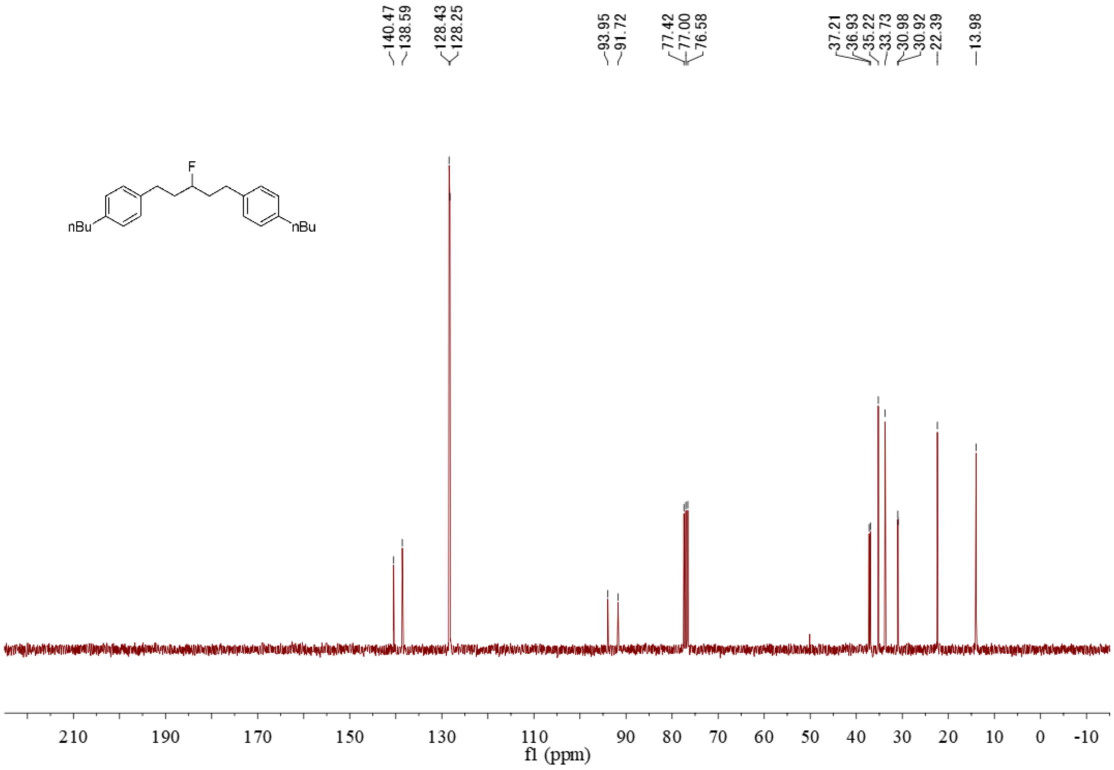

Figure S206 ^{19}F NMR spectrum of unknown secondary monofluoride **4e**, related to **Figure 3**

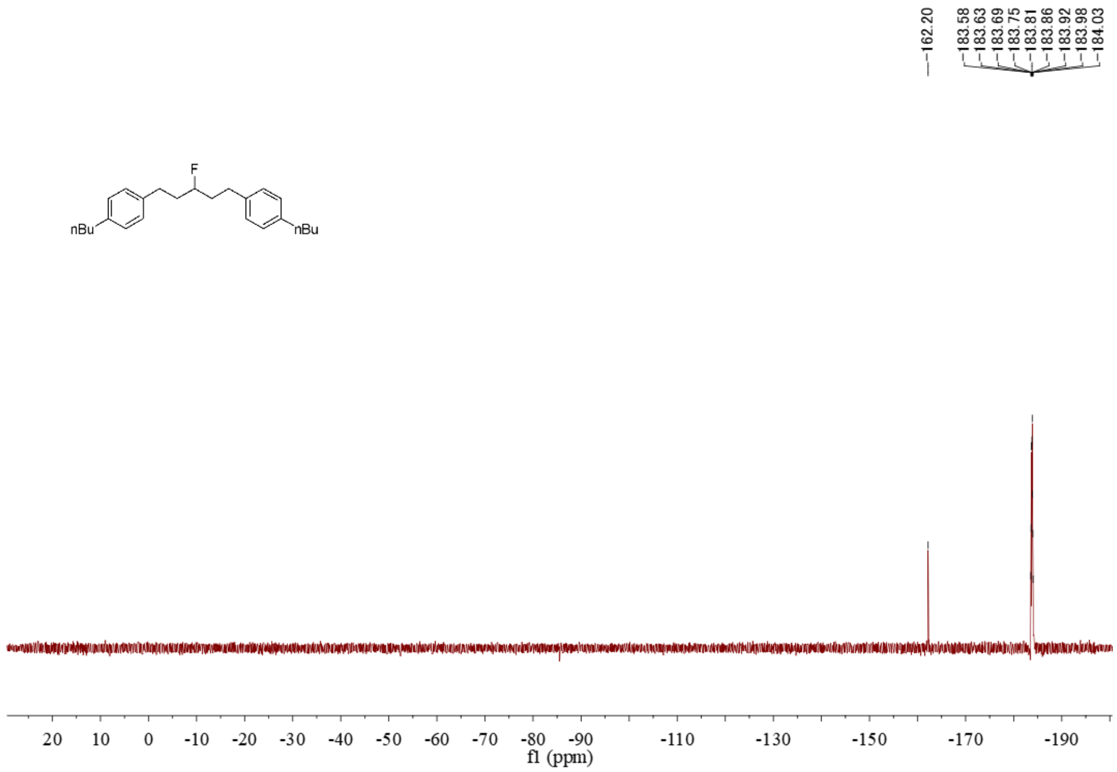

Figure S207. ^{1}H NMR spectrum of unknown secondary monofluoride **4f**, related to **Figure 3**

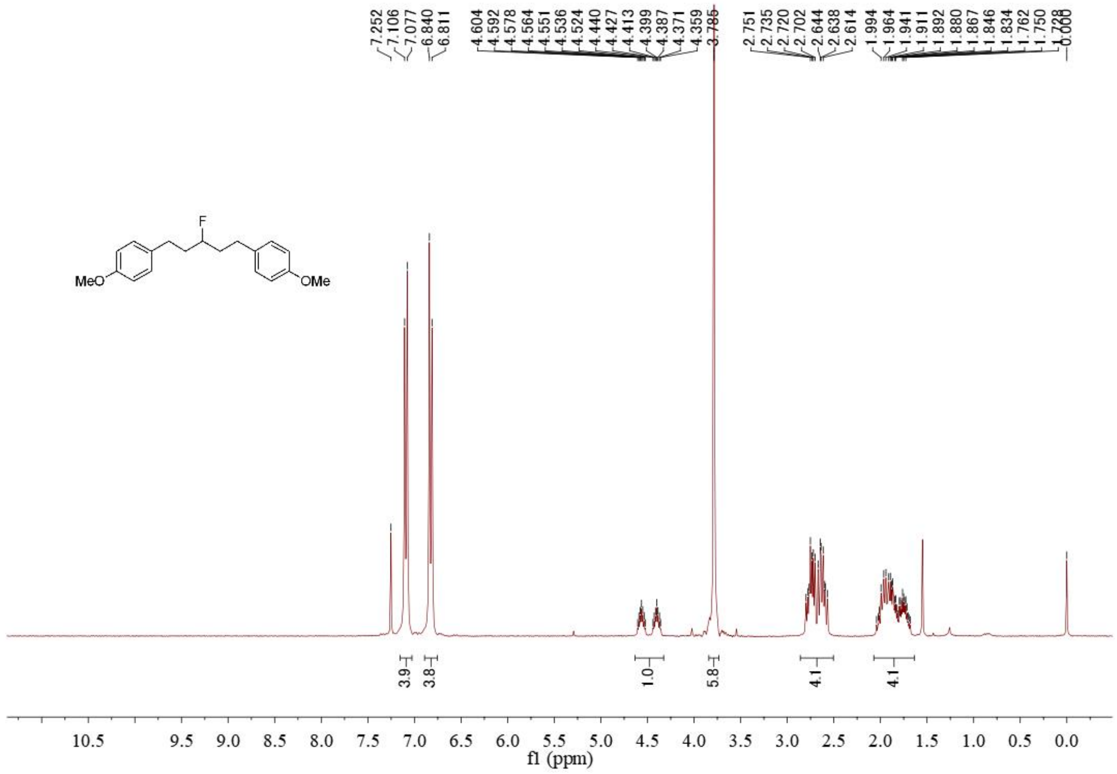

Figure S208. ^{13}C NMR spectrum of unknown secondary monofluoride **4f**, related to **Figure 3**

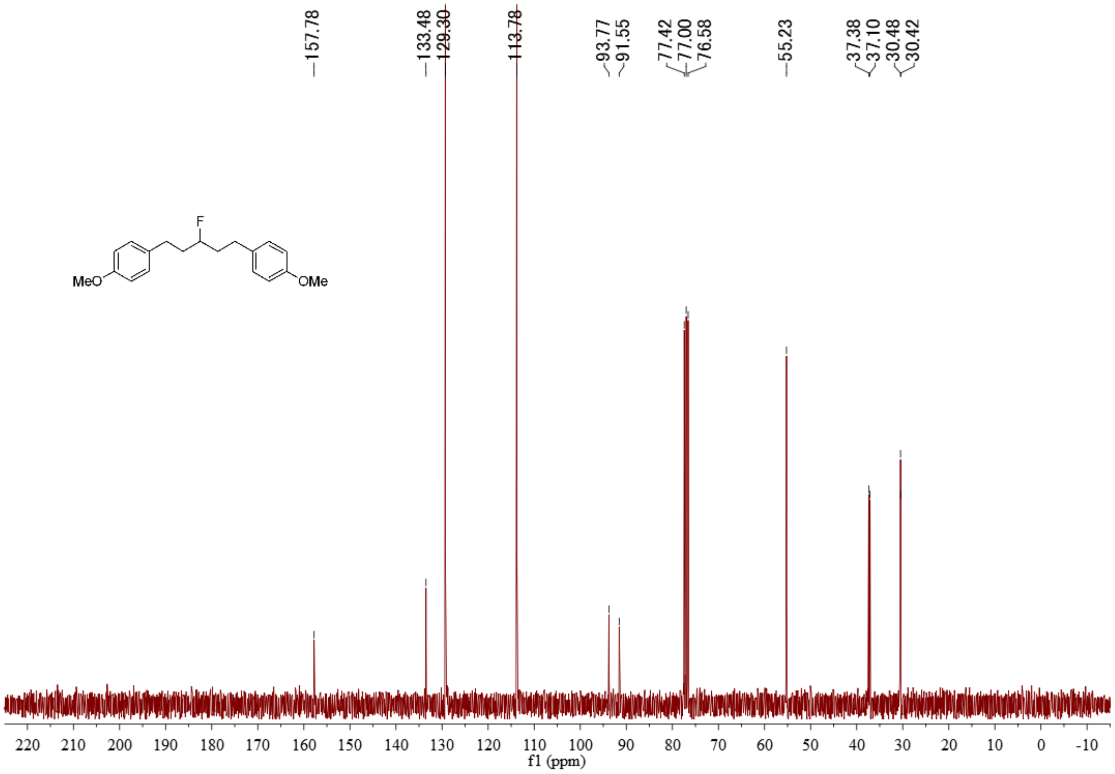

Figure S209. ^{19}F NMR spectrum of unknown secondary monofluoride **4f**, related to **Figure 3**

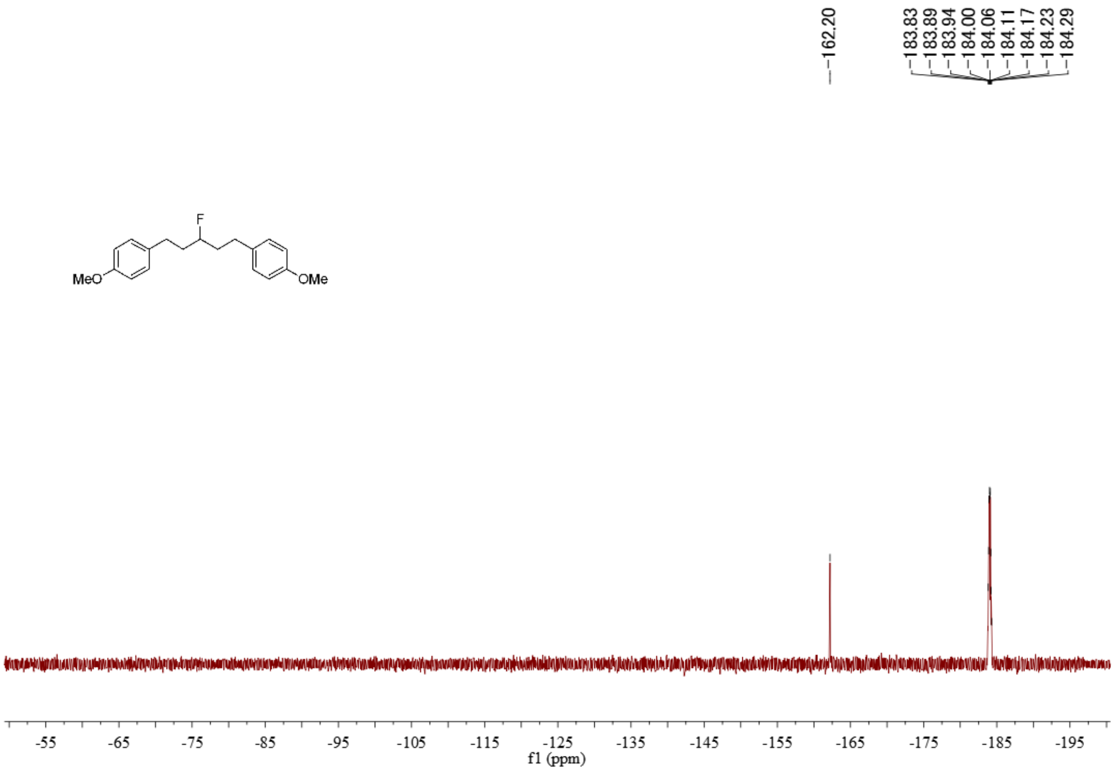

Figure S210. ^{1}H NMR spectrum of unknown secondary monofluoride **4g**, related to **Figure 3**

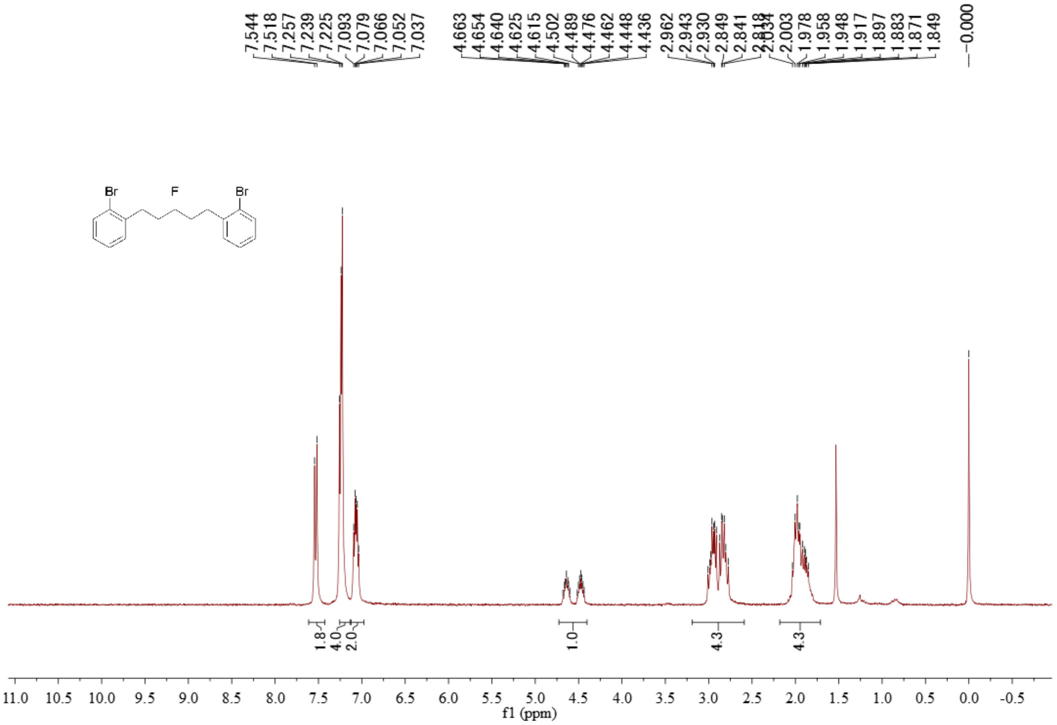

Figure S211. ^{13}C NMR spectrum of unknown secondary monofluoride **4g**, related to **Figure 3**

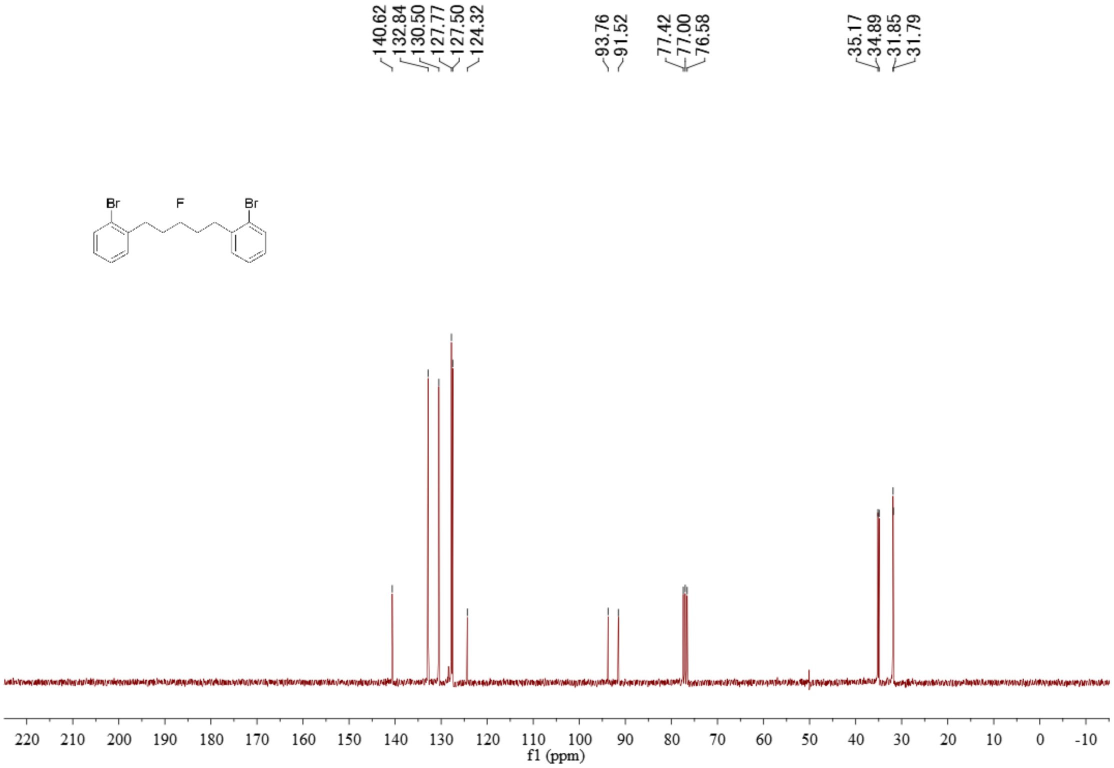

Figure S212. ^{19}F NMR spectrum of unknown secondary monofluoride **4g**, related to **Figure 3**

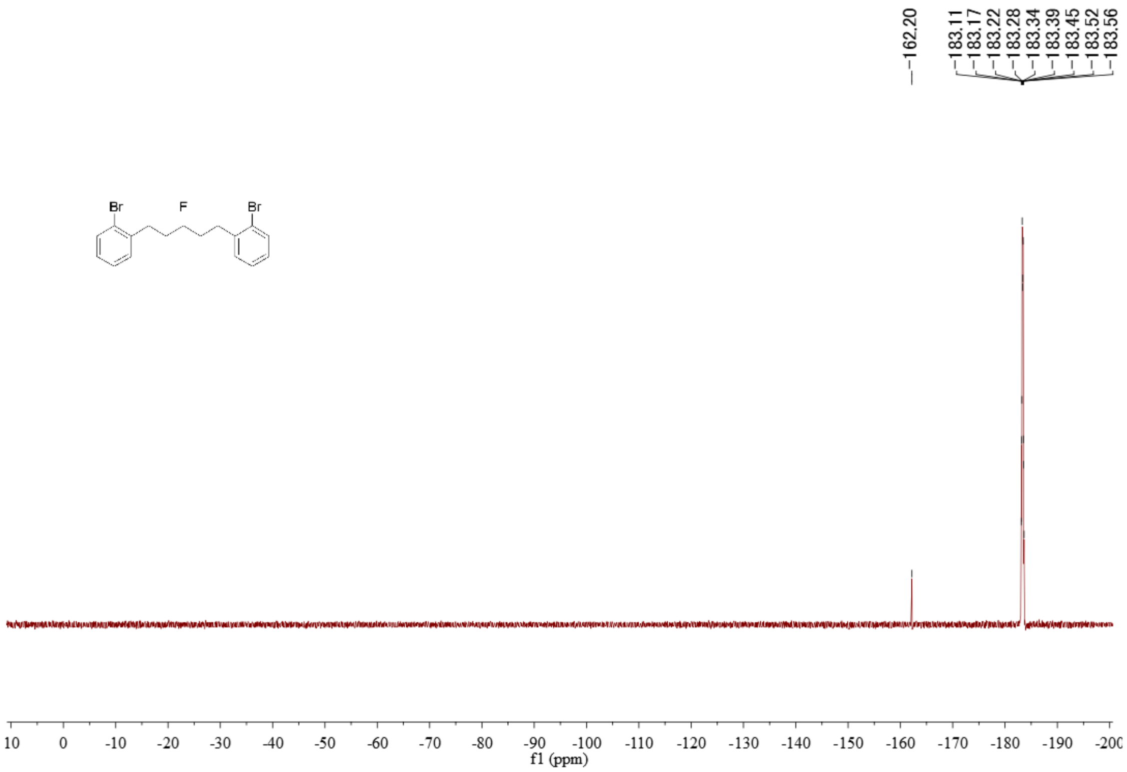

Figure S213. ^{1}H NMR spectrum of unknown secondary monofluoride **4h**, related to **Figure 3**

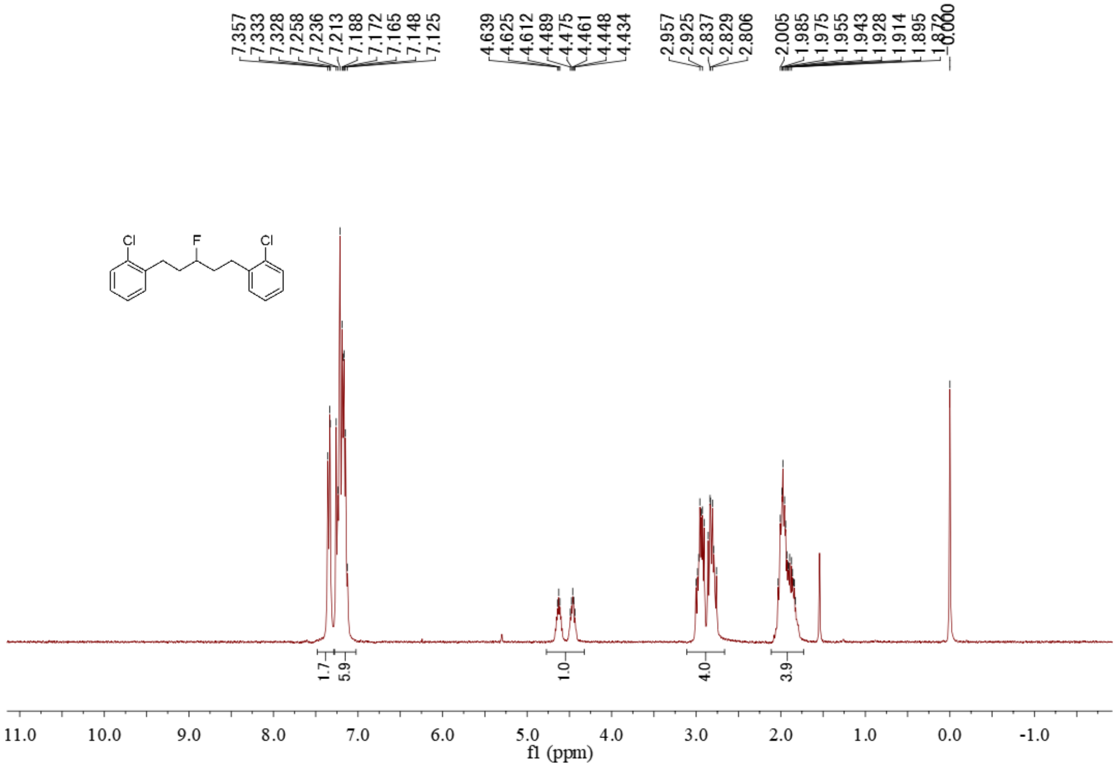

Figure S214. ^{13}C NMR spectrum of unknown secondary monofluoride **4h**, related to **Figure 3**

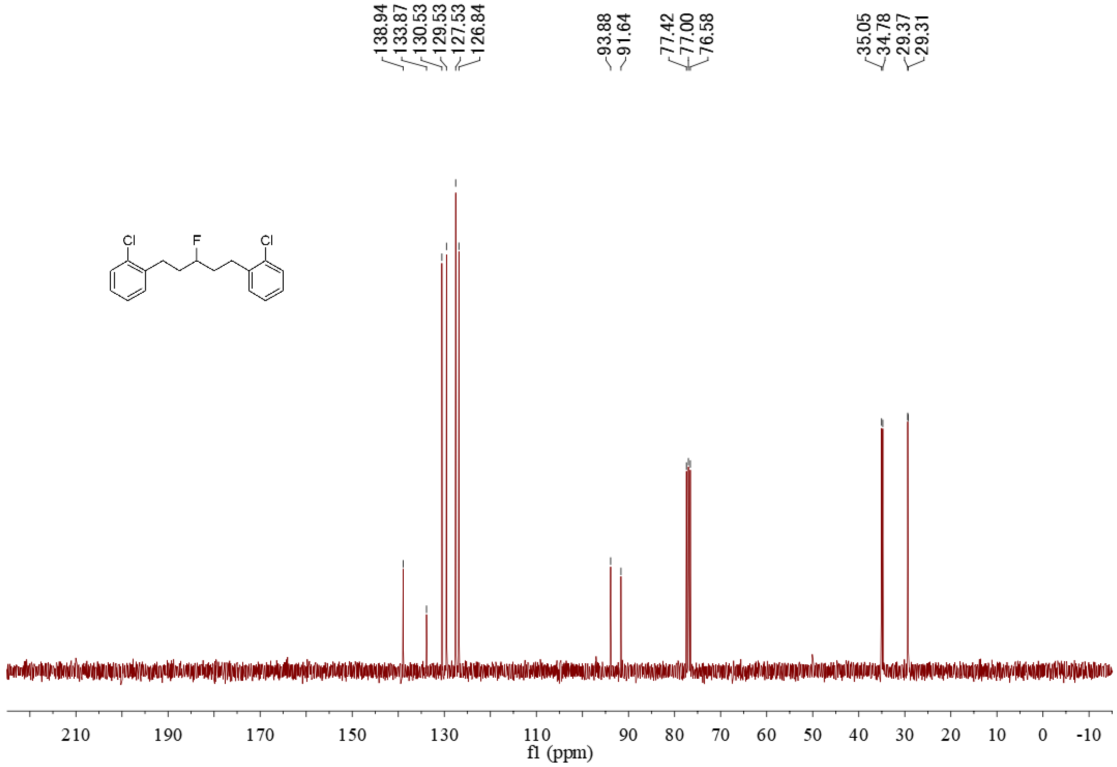

Figure S215. ^{19}F NMR spectrum of unknown secondary monofluoride **4h**, related to **Figure 3**

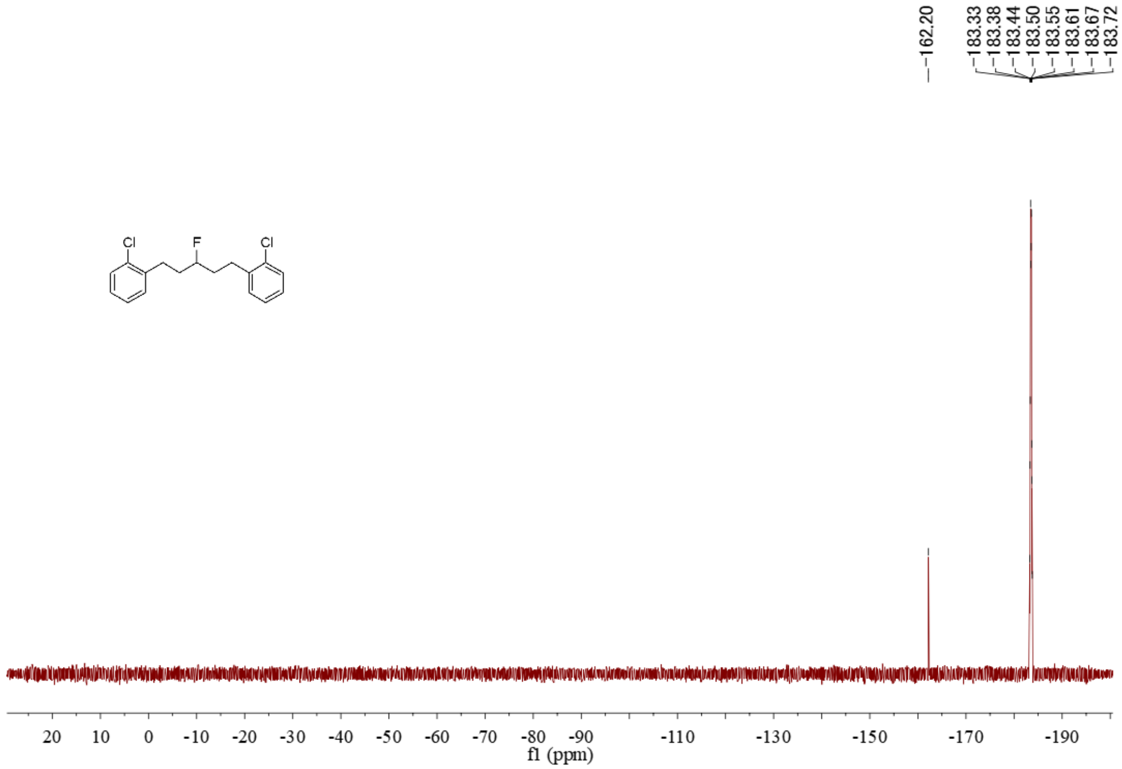

Figure S216. ^{1}H NMR spectrum of unknown secondary monofluoride **4i**, related to **Figure 3**

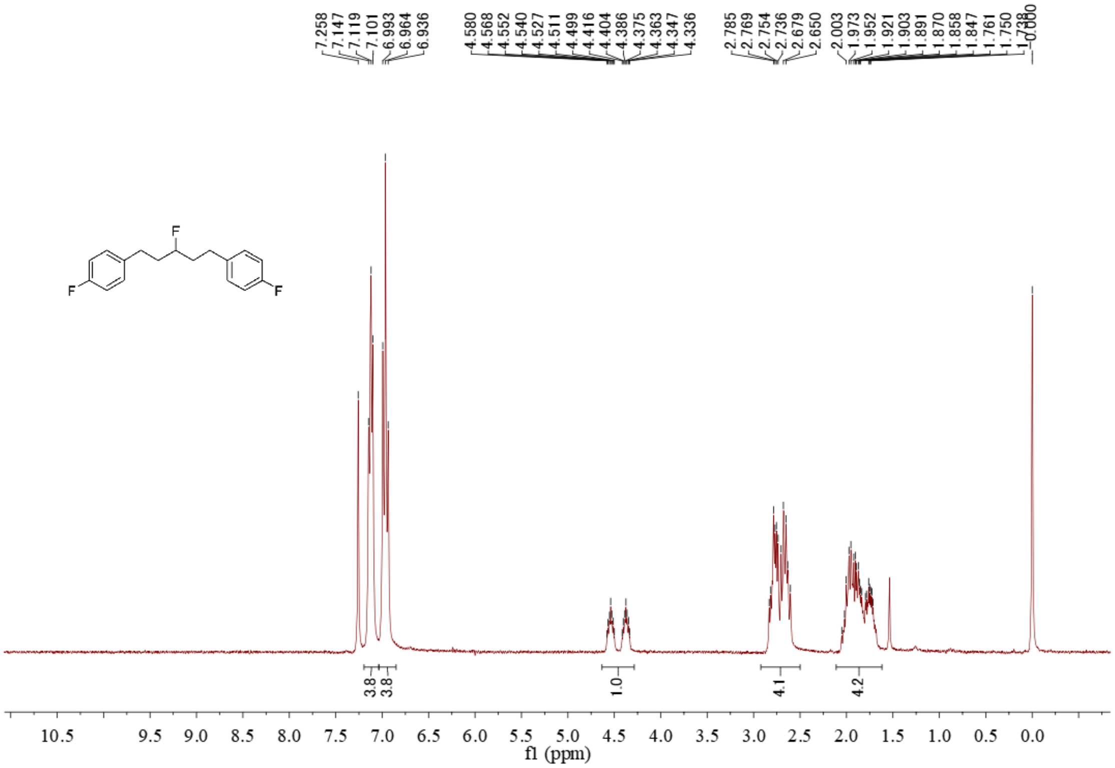

Figure S217. ^{13}C NMR spectrum of unknown secondary monofluoride **4i**, related to **Figure 3**

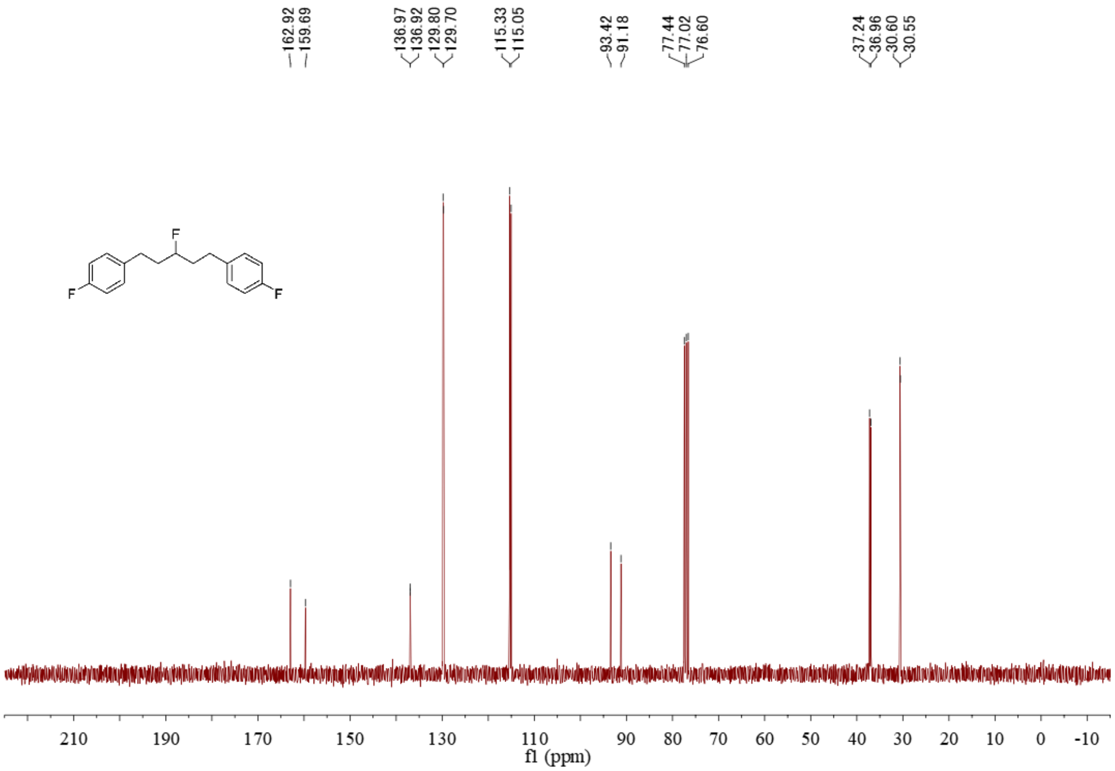

Figure S218. ^{19}F NMR spectrum of unknown secondary monofluoride **4i**, related to **Figure 3**

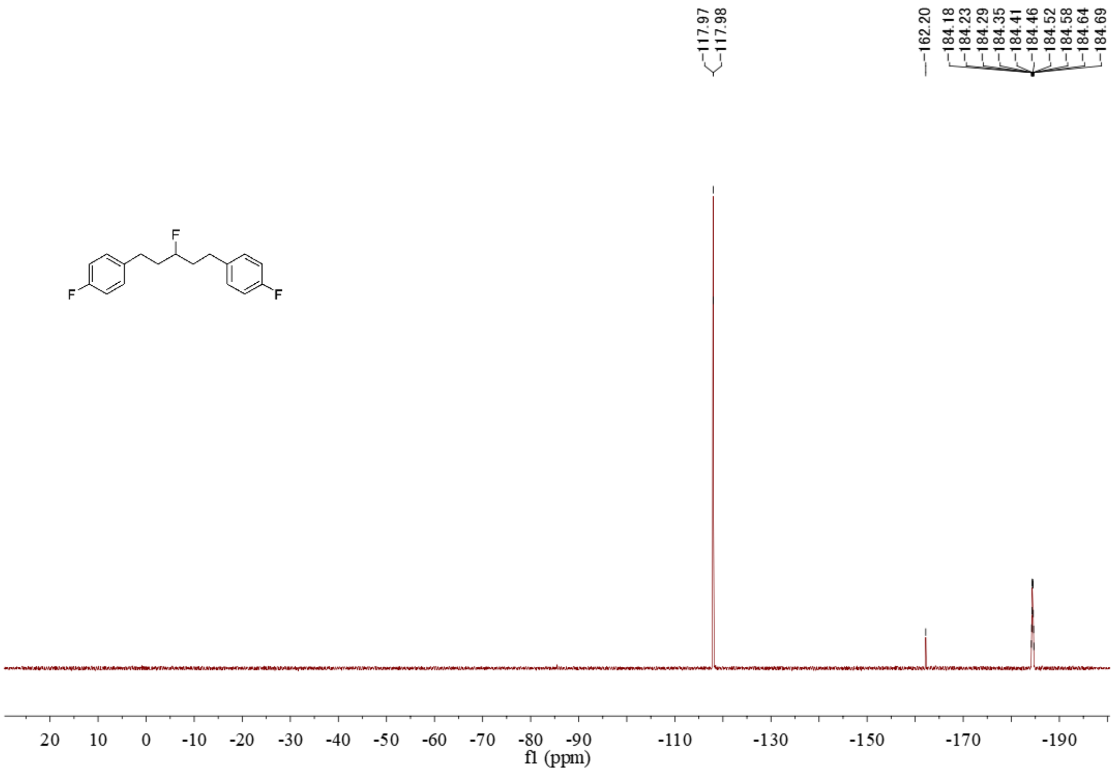

Figure S219. ^{1}H NMR spectrum of unknown secondary monofluoride **4j**, related to **Figure 3**

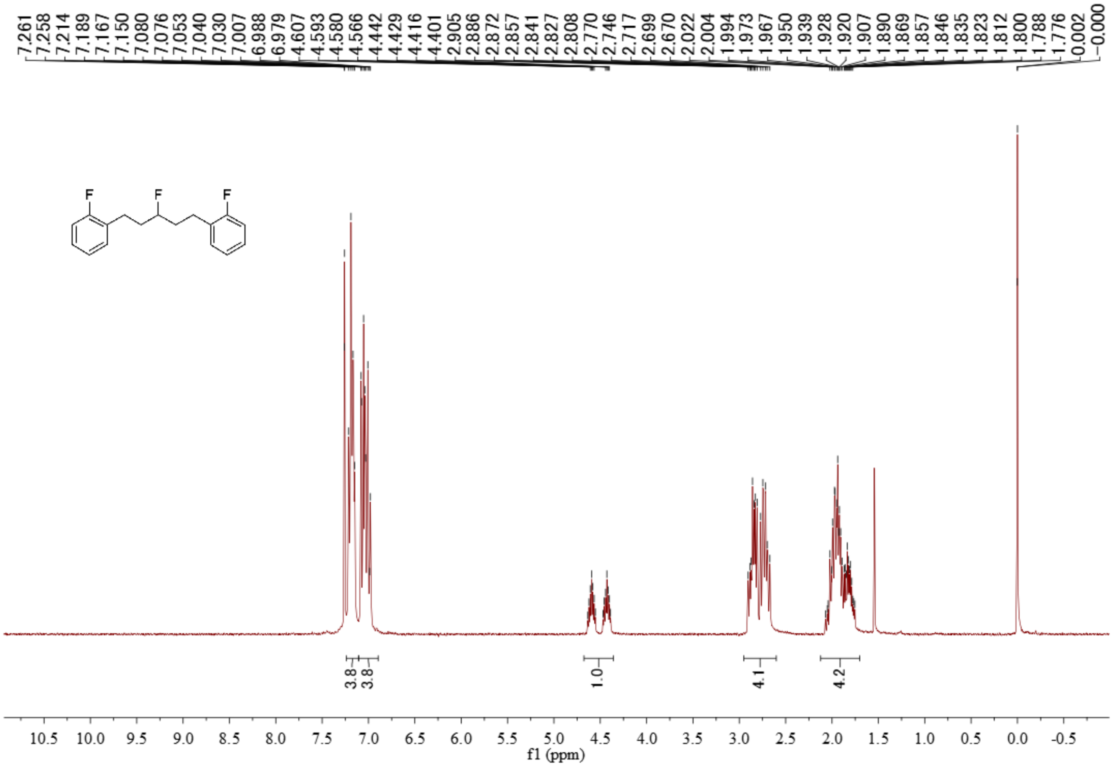

Figure S220. ^{13}C NMR spectrum of unknown secondary monofluoride **4j**, related to **Figure 3**

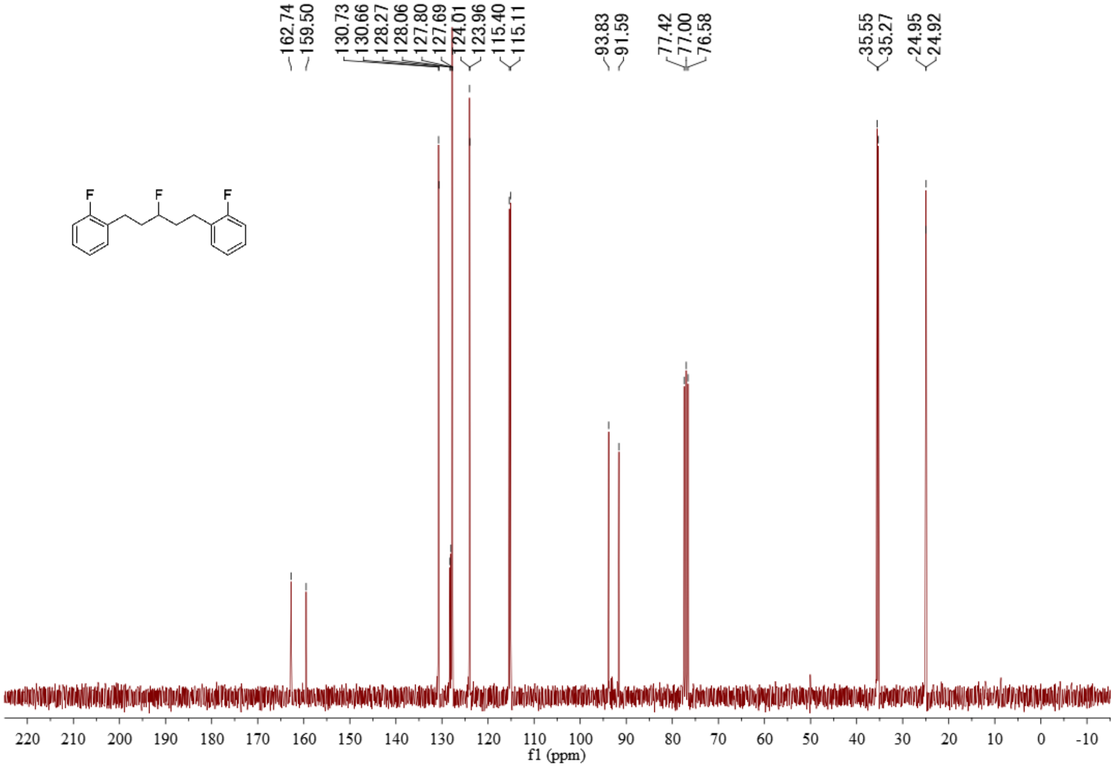

Figure S221. ^{19}F NMR spectrum of unknown secondary monofluoride **4j**, related to **Figure 3**

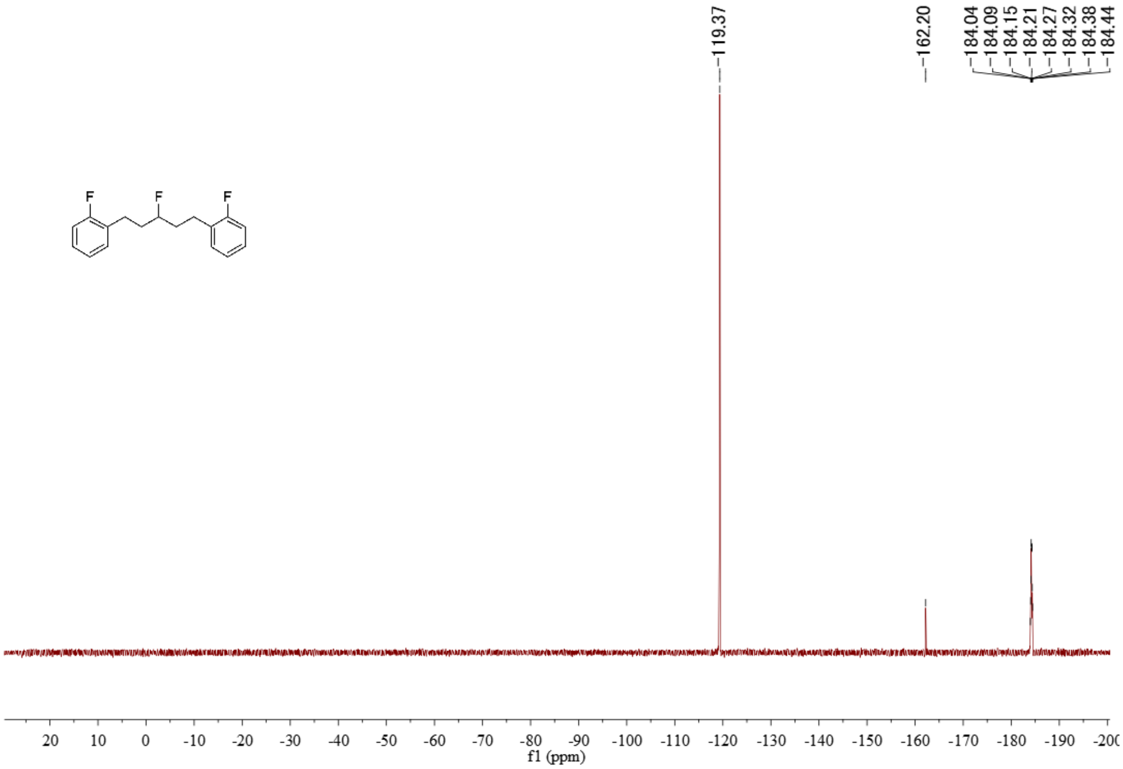

Figure S222. ^{1}H NMR spectrum of unknown secondary monofluoride **4k**, related to **Figure 3**

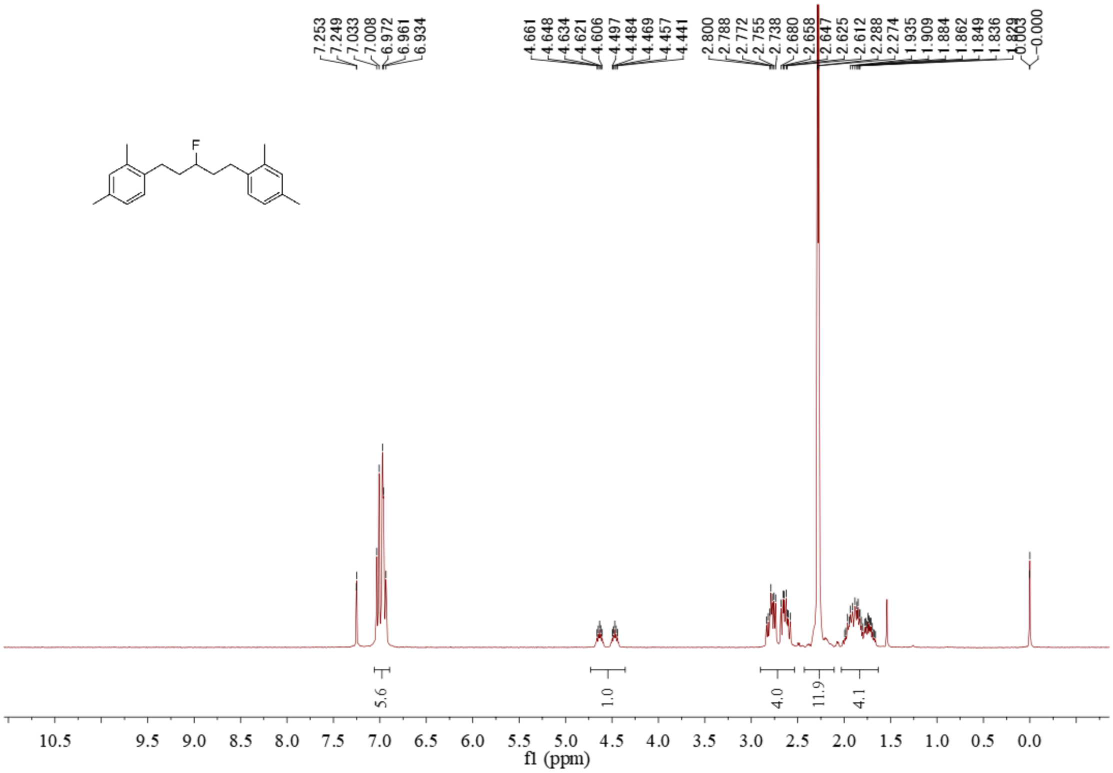

Figure S223. ^{13}C NMR spectrum of unknown secondary monofluoride **4k**, related to **Figure 3**

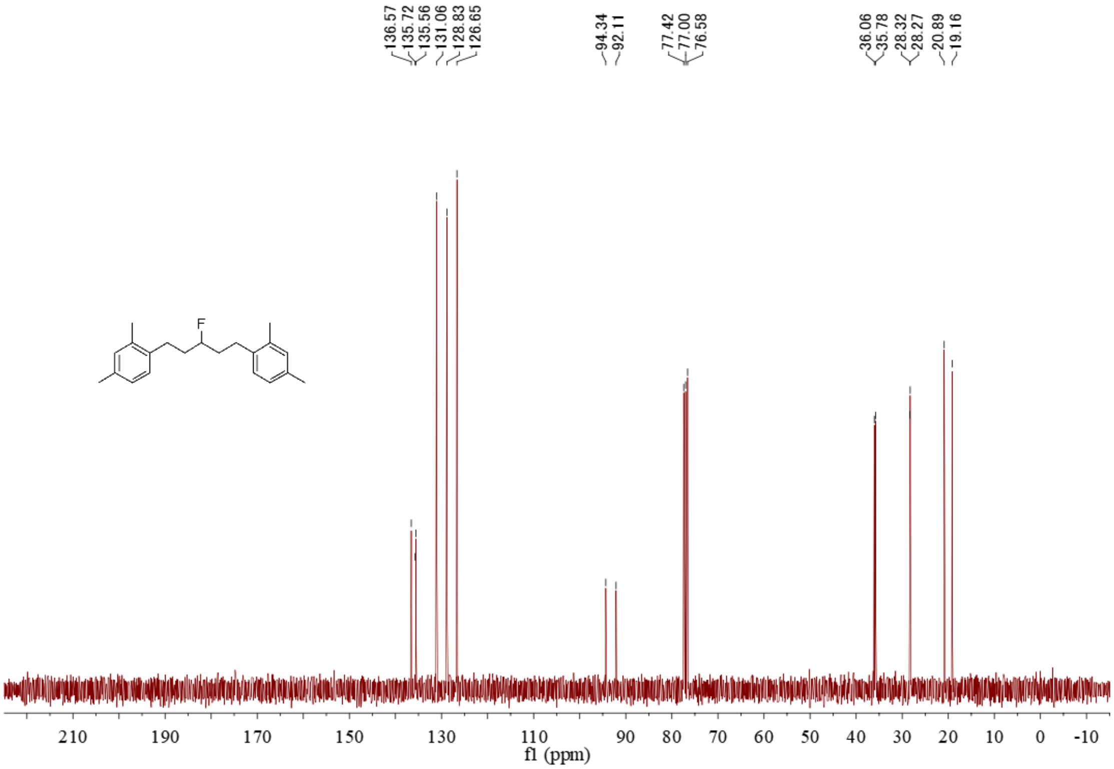

Figure S224. ^{19}F NMR spectrum of unknown secondary monofluoride **4k**, related to **Figure 3**

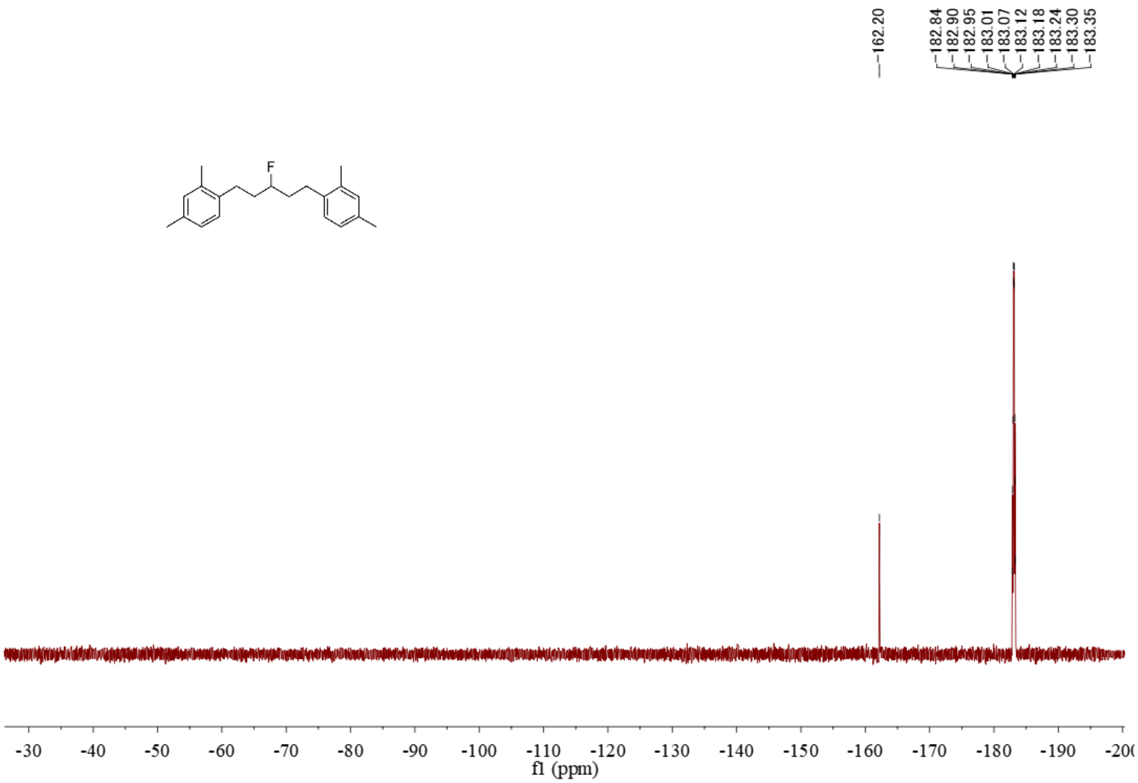

Figure S225. ^{1}H NMR spectrum of unknown secondary monofluoride **4l**, related to **Figure 3**

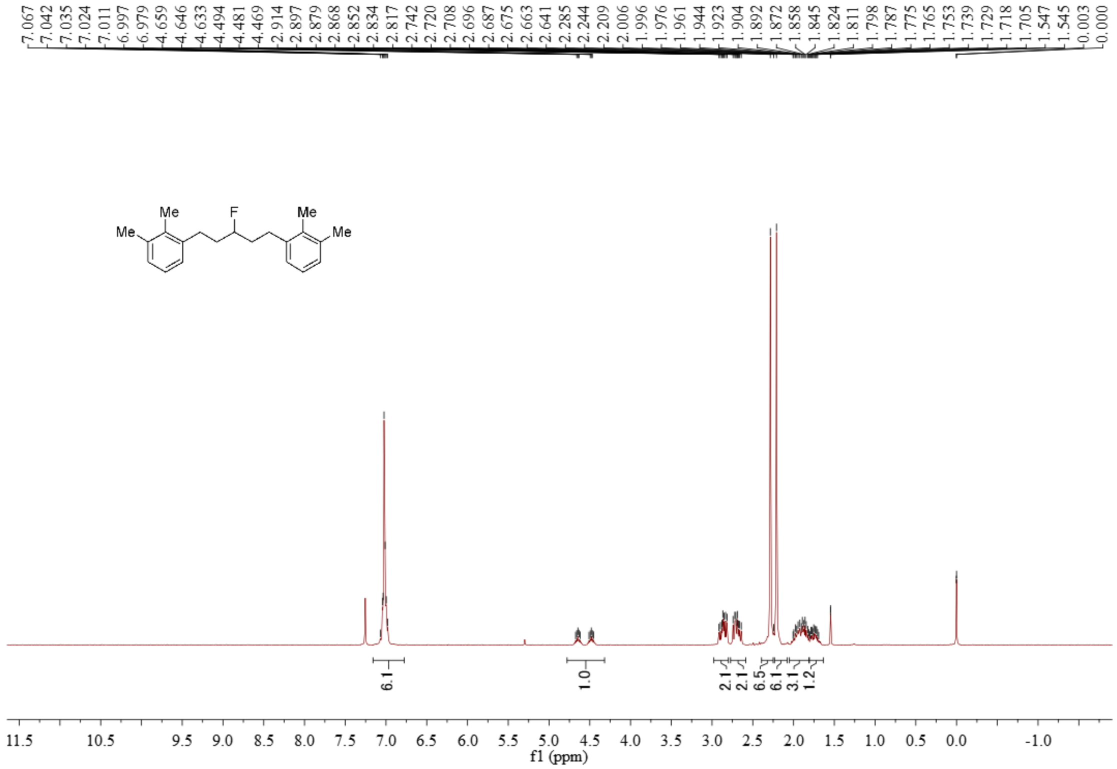

Figure S226. ^{13}C NMR spectrum of unknown secondary monofluoride **4l**, related to **Figure 3**

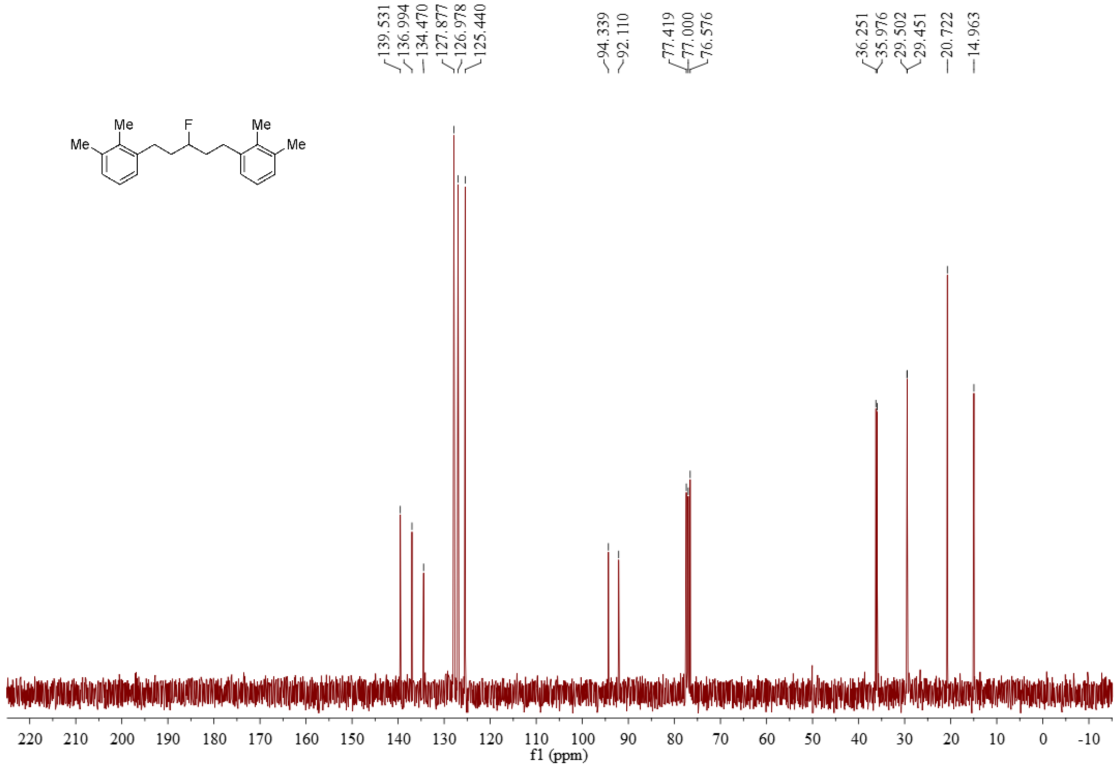

Figure S227. ^{19}F NMR spectrum of unknown secondary monofluoride **4l**, related to **Figure 3**

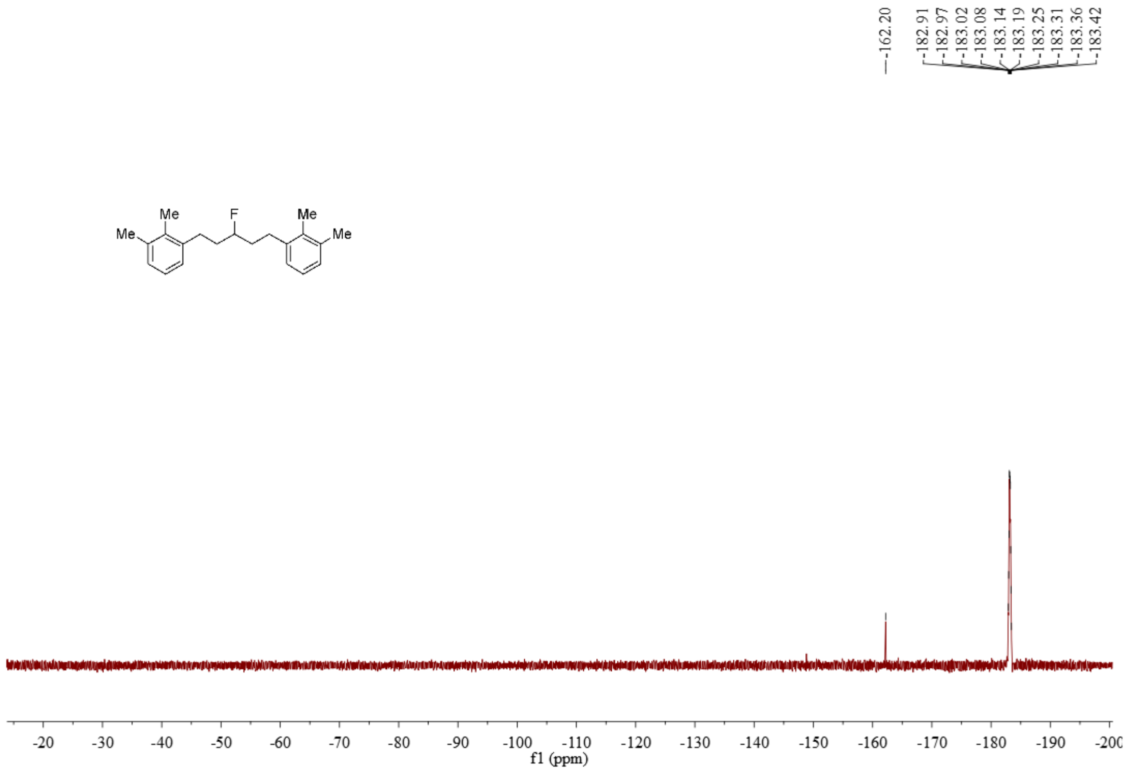

Figure S228. ^{1}H NMR spectrum of unknown secondary monofluoride **4m**, related to **Figure 3**

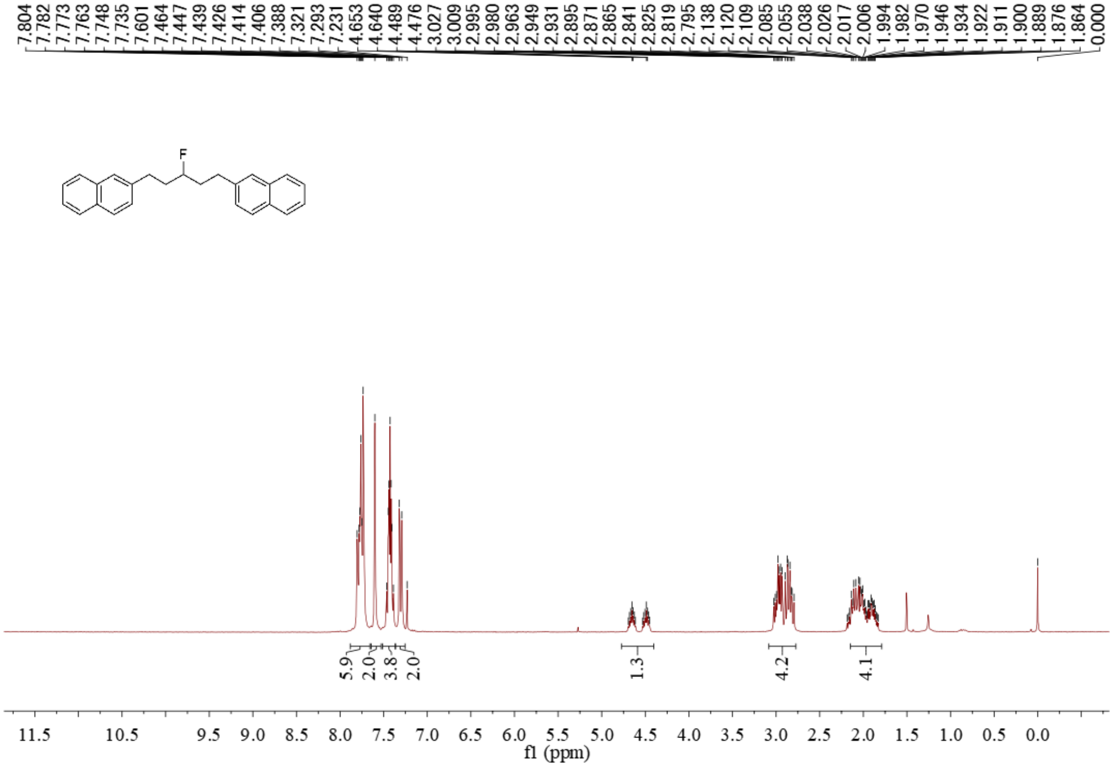

Figure S229. ^{13}C NMR spectrum of unknown secondary monofluoride **4m**, related to **Figure 3**

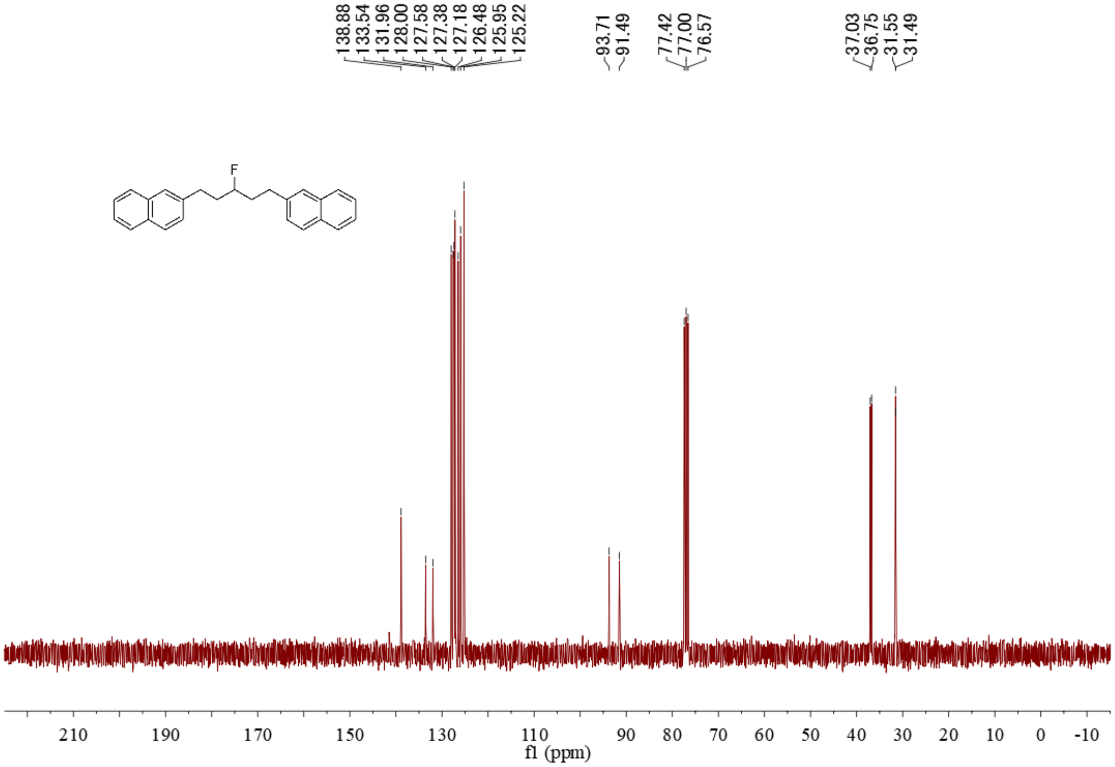

Figure S230. ^{19}F NMR spectrum of unknown secondary monofluoride **4m**, related to **Figure 3**

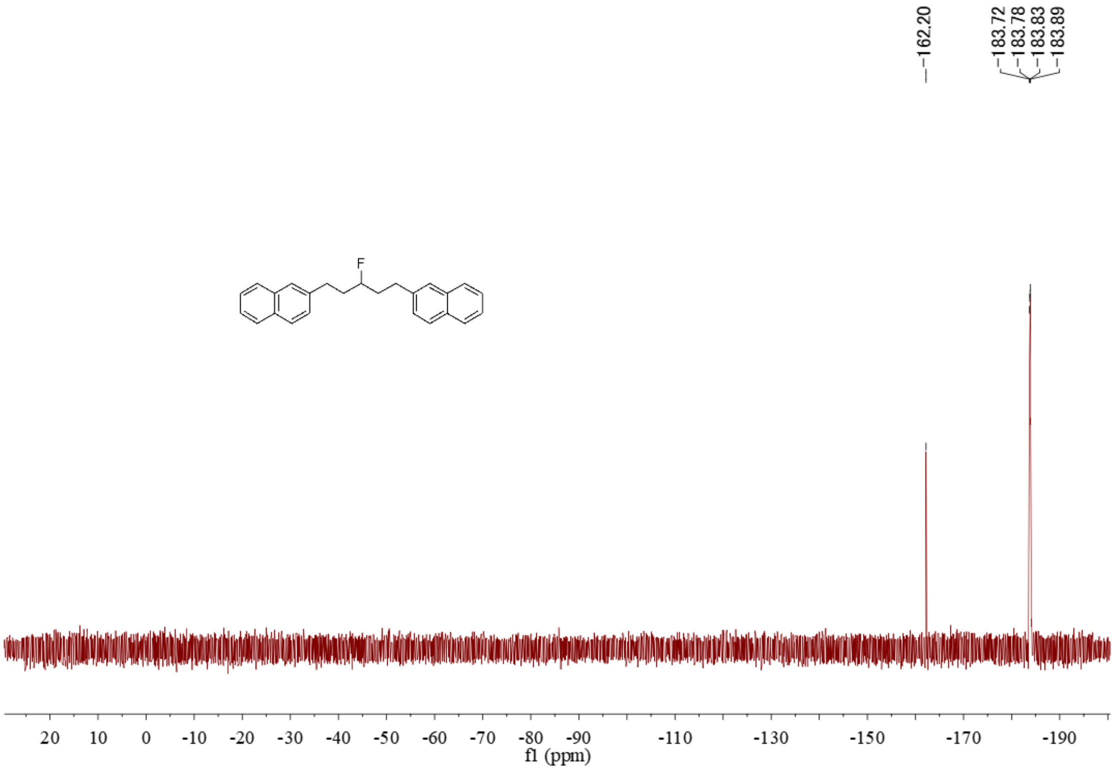

Figure S231. ^{1}H NMR spectrum of unknown secondary monofluoride **4n**, related to **Figure 3**

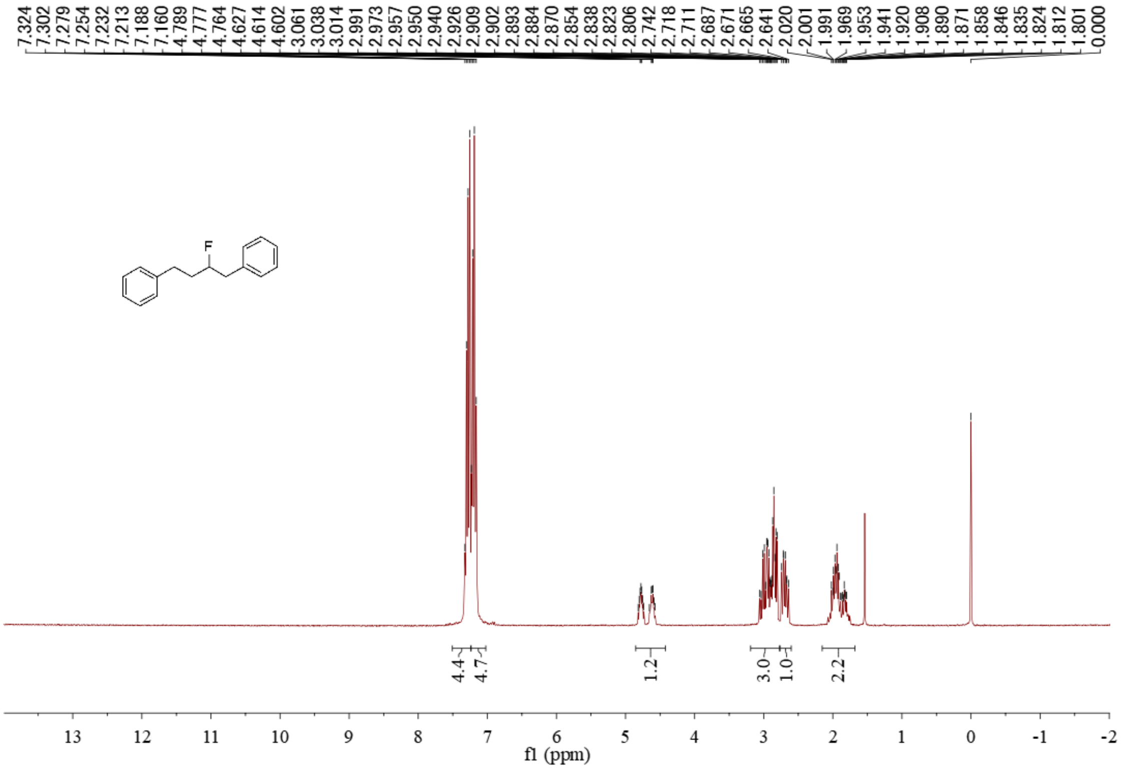

Figure S232. ^{13}C NMR spectrum of unknown secondary monofluoride **4n**, related to **Figure 3**

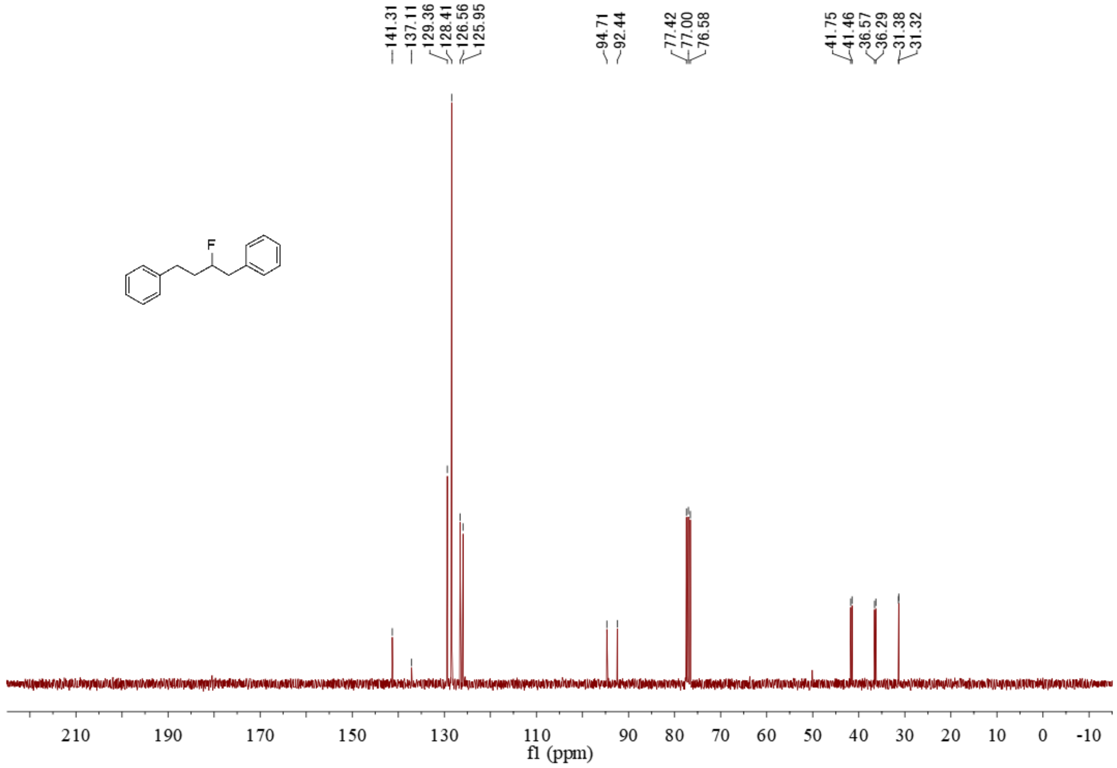

Figure S233. ^{19}F NMR spectrum of unknown secondary monofluoride **4n**, related to **Figure 3**

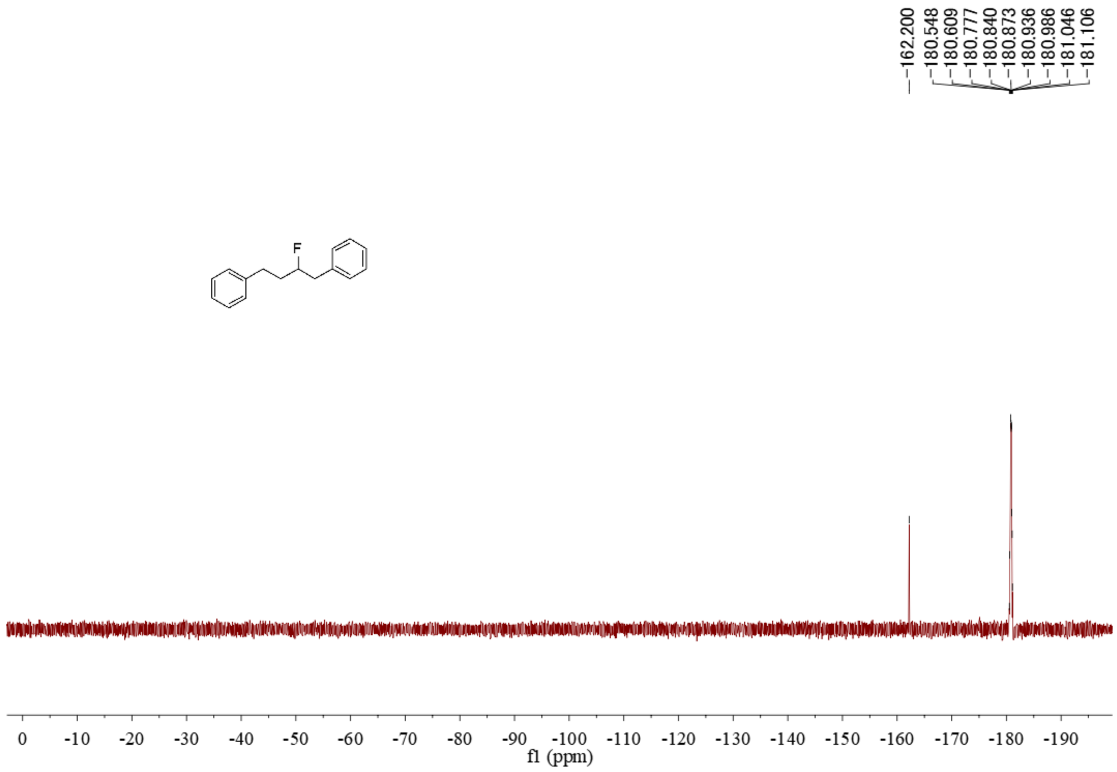

Figure S234. ^{1}H NMR spectrum of unknown secondary monofluoride **4p**, related to **Figure 3**

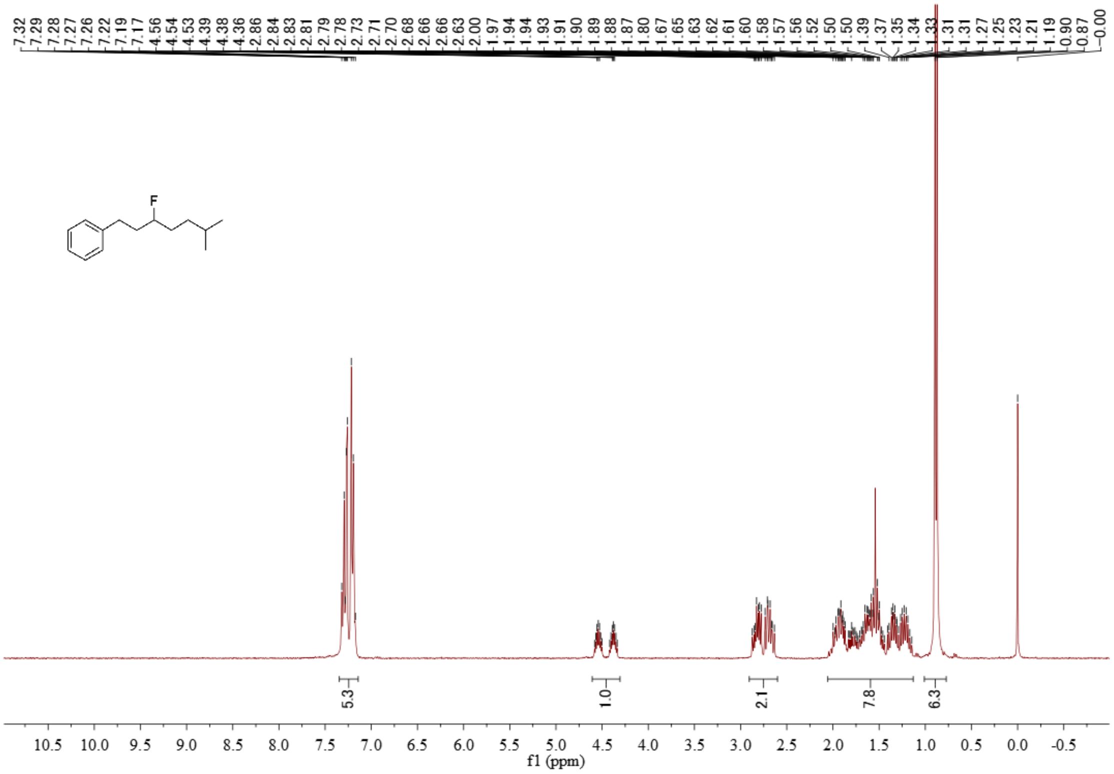

Figure S235. ^{13}C NMR spectrum of unknown secondary monofluoride **4p**, related to **Figure 3**

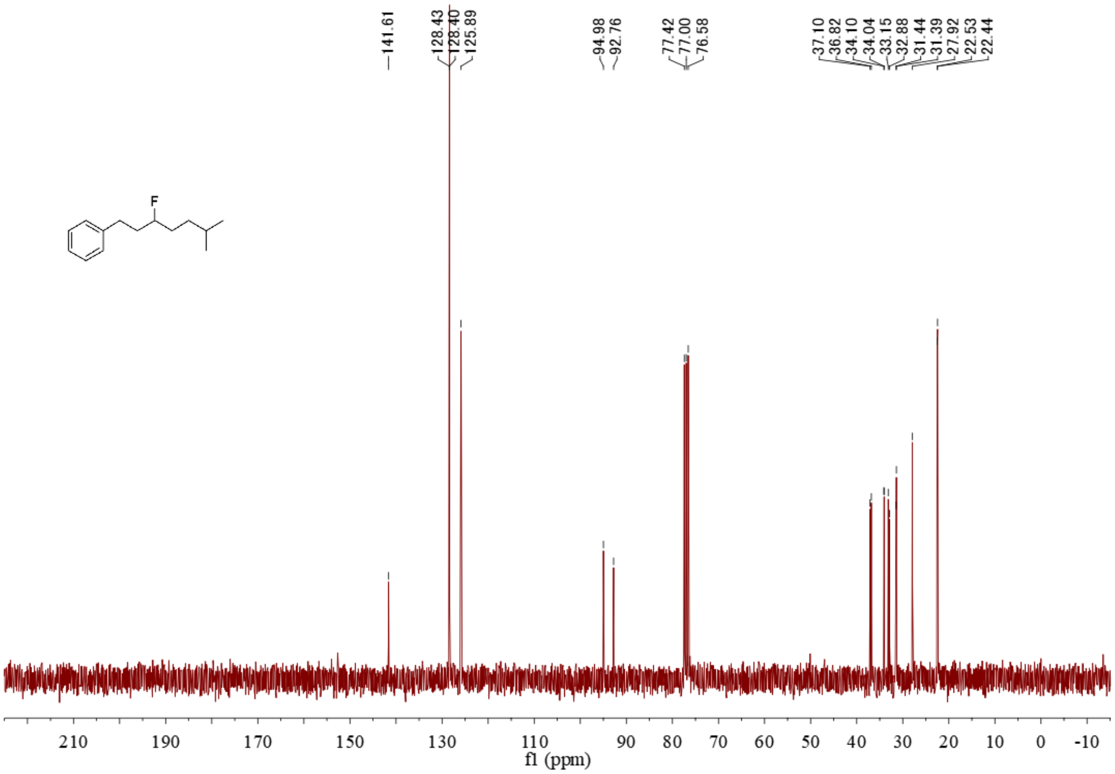

Figure S236. ^{19}F NMR spectrum of unknown secondary monofluoride **4p**, related to **Figure 3**

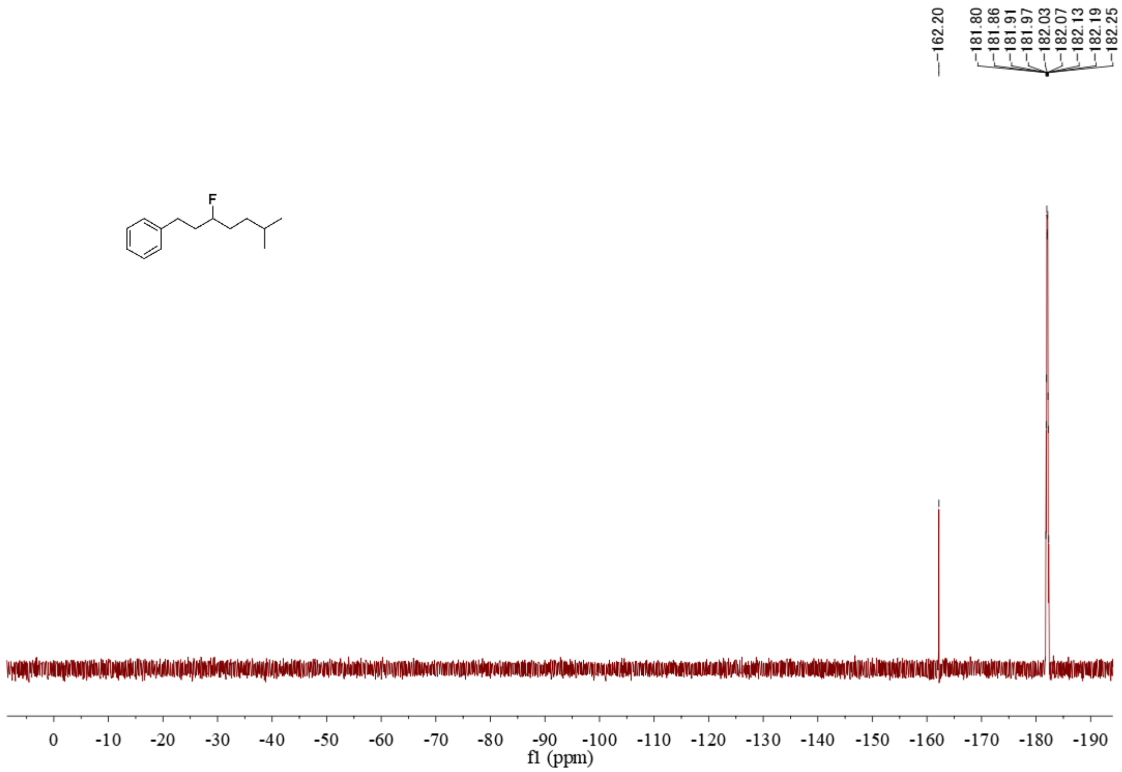

Figure S237. ^{1}H NMR spectrum of unknown secondary monofluoride **4r**, related to **Figure 3**

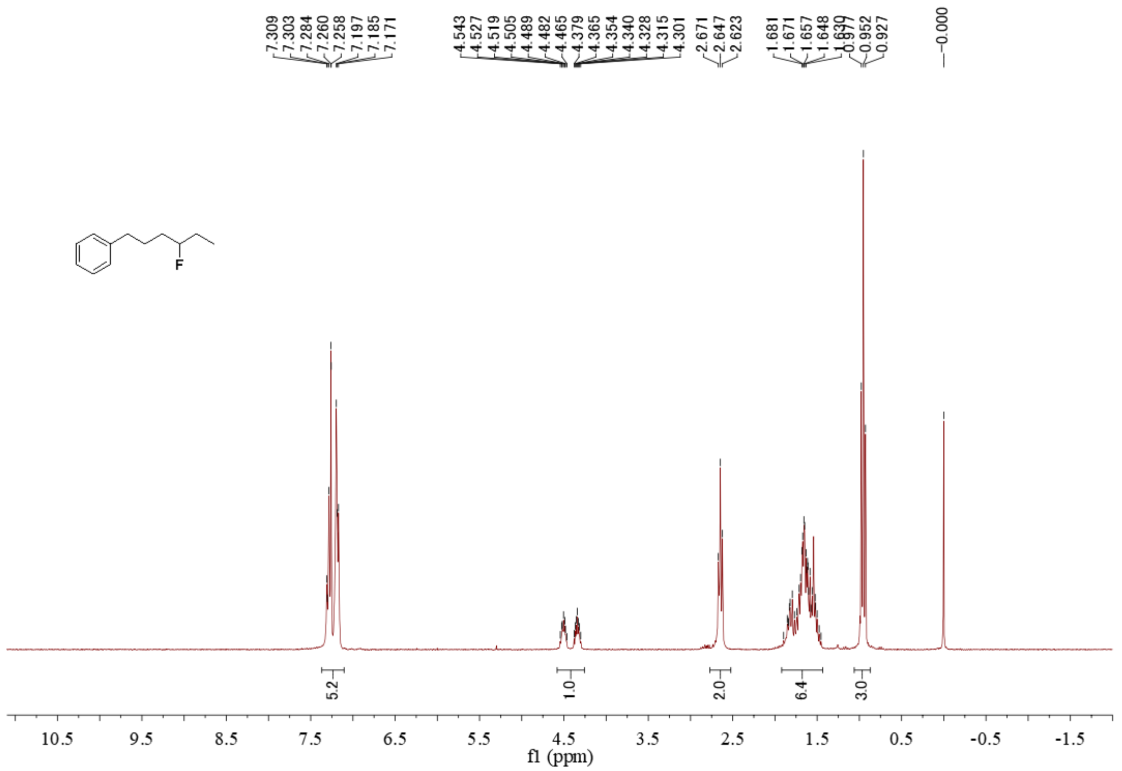

Figure S238. ^{13}C NMR spectrum of unknown secondary monofluoride **4r**, related to **Figure 3**

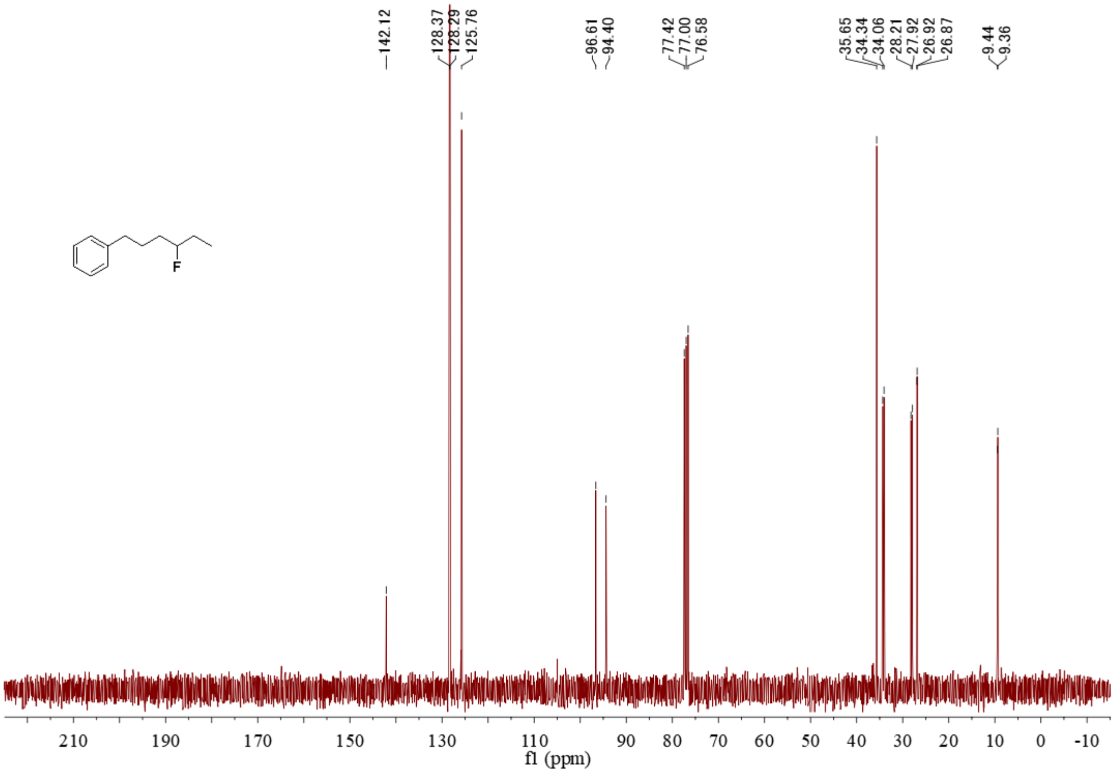

Figure S239. ^{19}F NMR spectrum of unknown secondary monofluoride **4r**, related to **Figure 3**

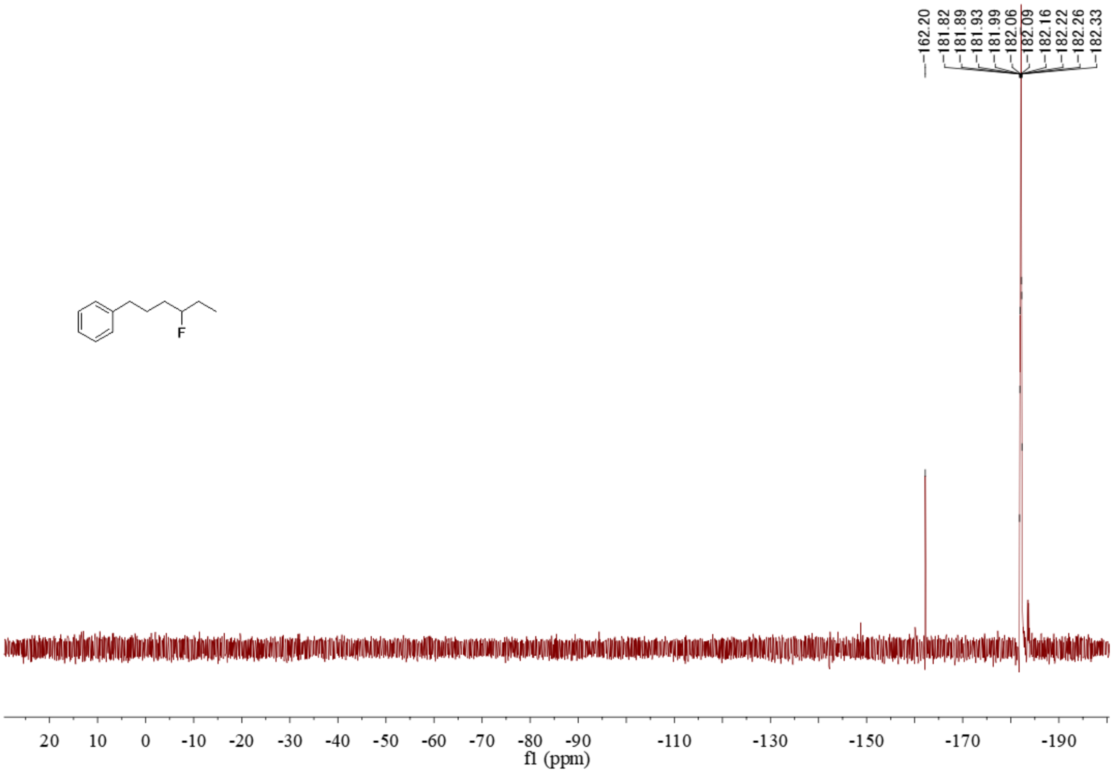

Figure S240. ^{1}H NMR spectrum of unknown secondary monofluoride **4s**, related to **Figure 3**

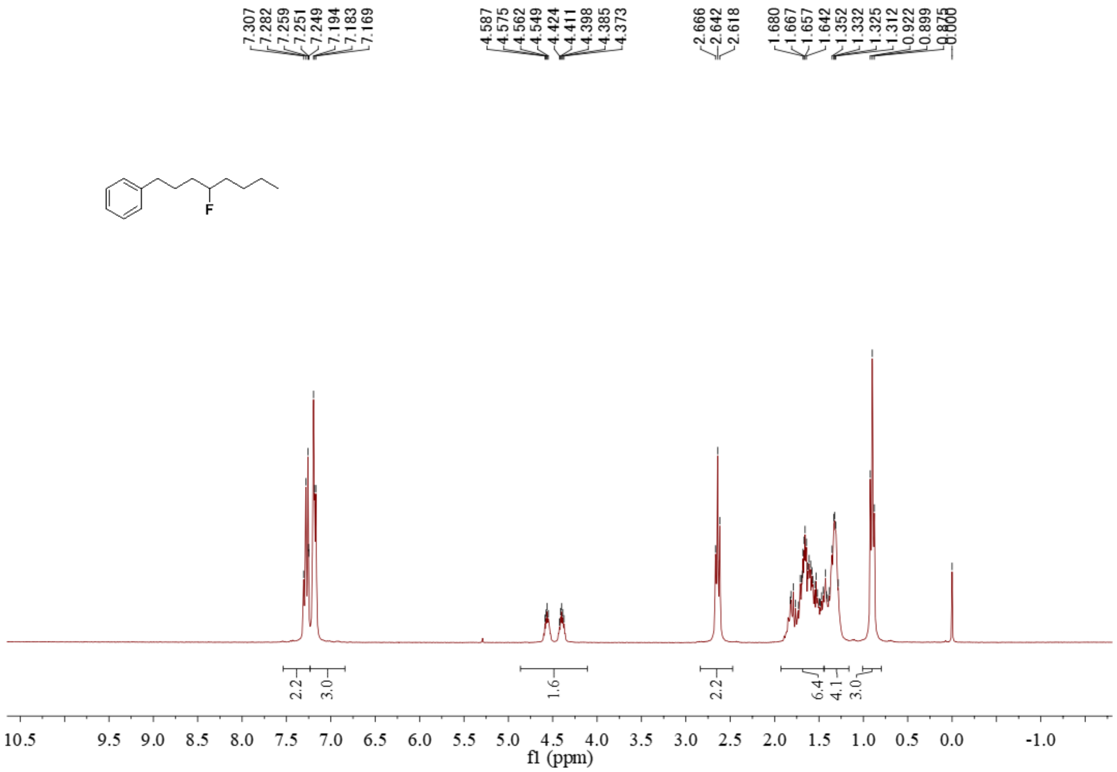

Figure S241. ^{13}C NMR spectrum of unknown secondary monofluoride **4s**, related to **Figure 3**

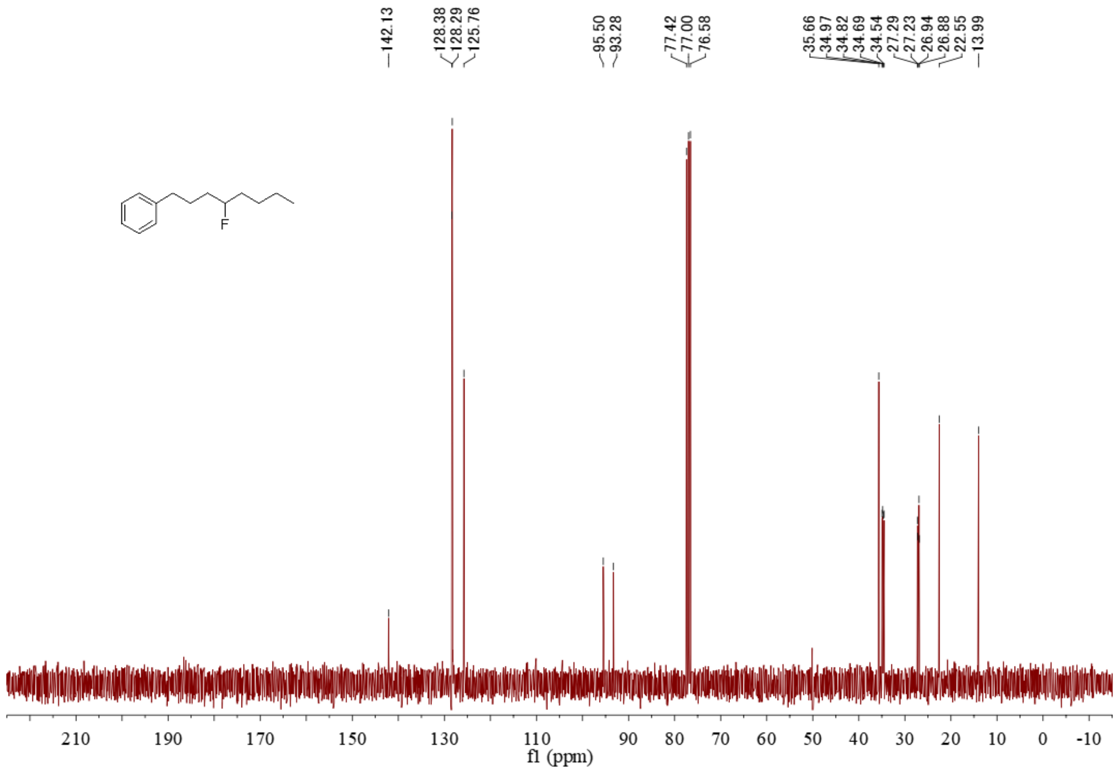

Figure S242. ^{19}F NMR spectrum of unknown secondary monofluoride **4s**, related to **Figure 3**

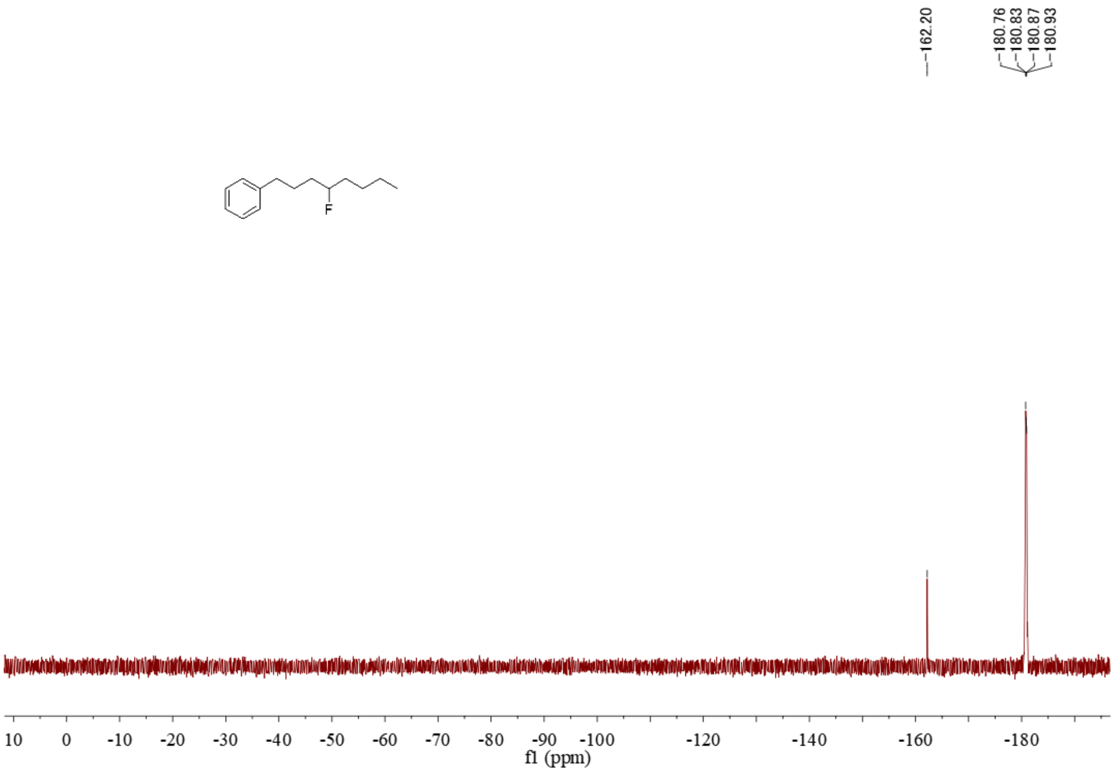

Figure S243. ^{1}H-NMR spectra copy of crude reaction mixture: Using $(CF_3)_2CHOH$ as purchased without precaution to exclude moisture, related to **Table 1** (entry 7)

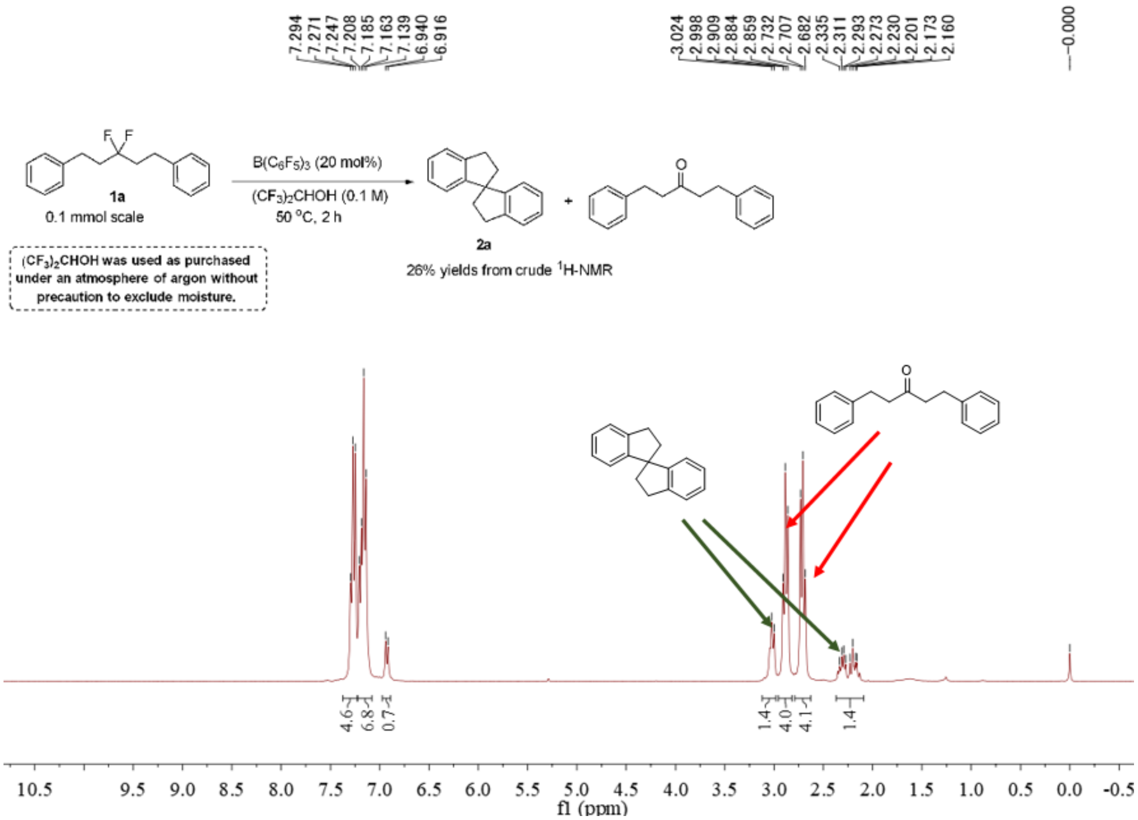

Figure S244. ^{1}H-NMR spectrum of **1v**, related to **Table 2**

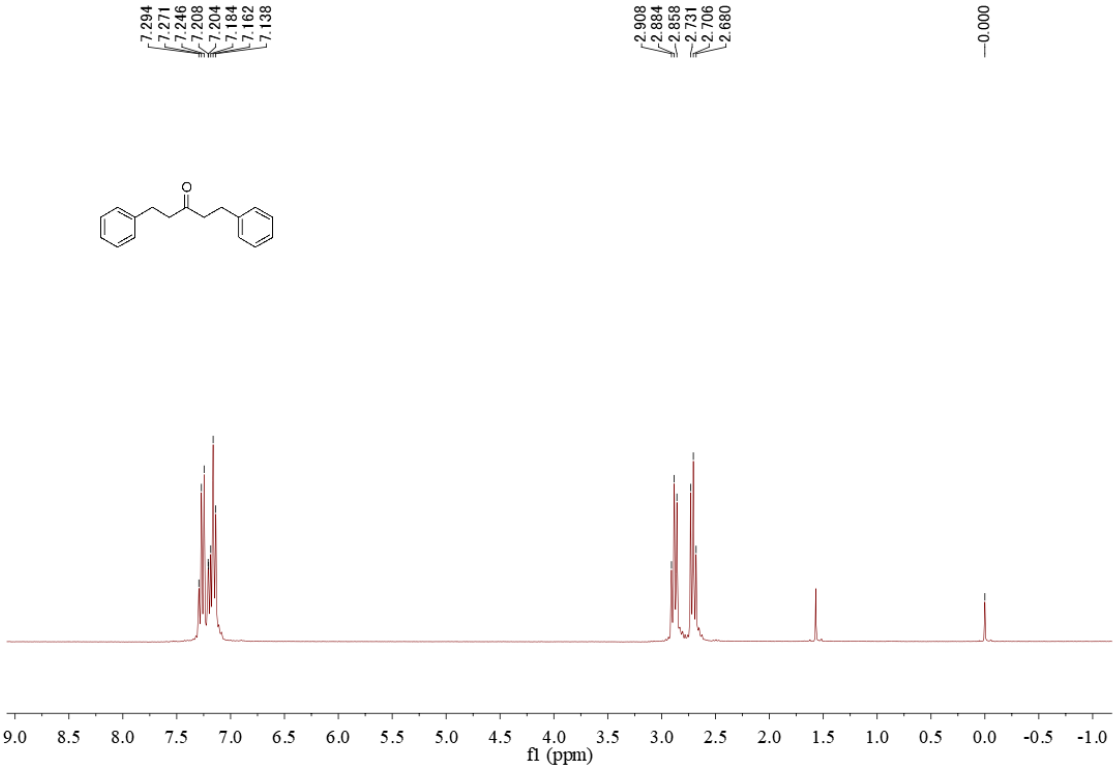

Figure S245. ^{1}H-NMR spectrum of **1w**, related to **Table 2**

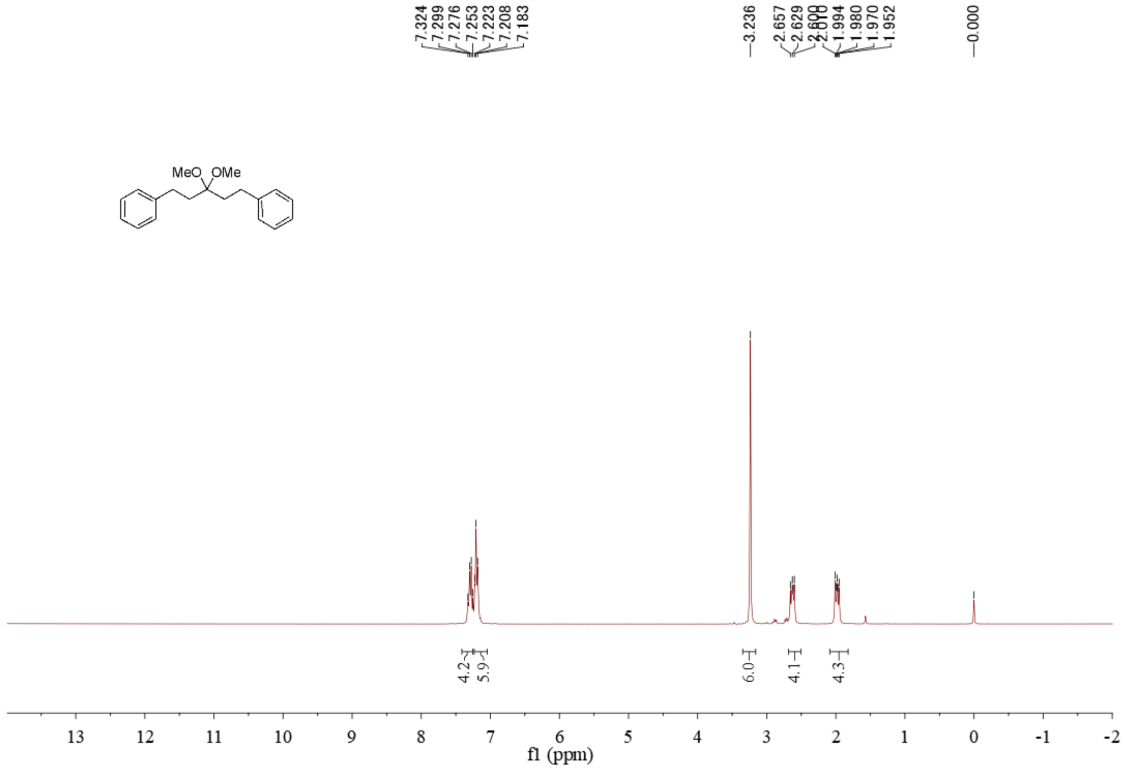

Figure S246. ^{1}H-NMR spectrum of **1x**, related to **Table 2**

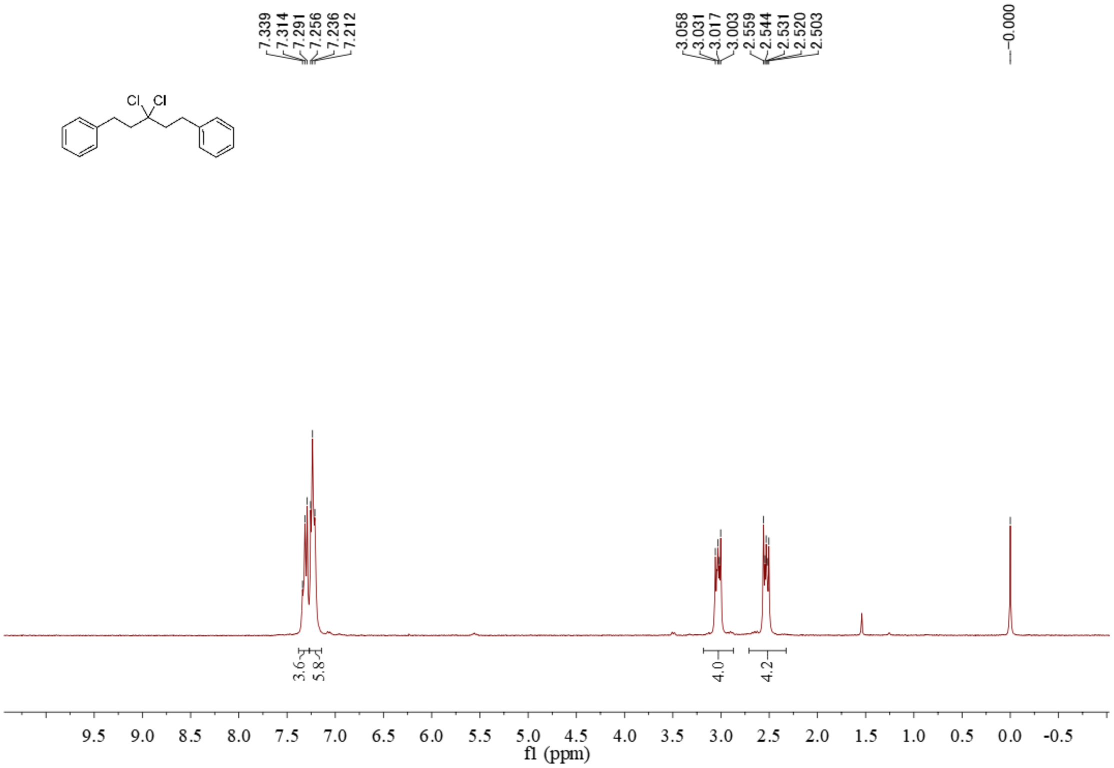

Figure S247. ^{1}H-NMR spectrum of **1y**, related to **Table 2**

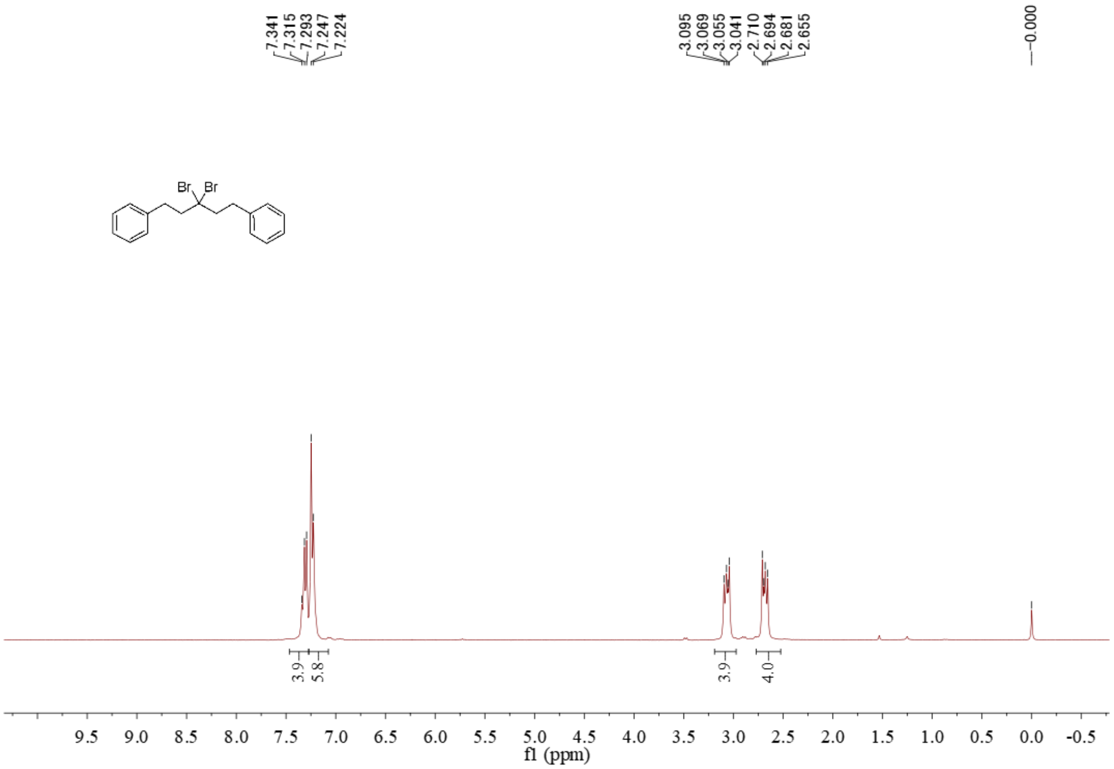

Figure S248. ^{1}H-NMR spectrum of **3x**, related to **Table 2**

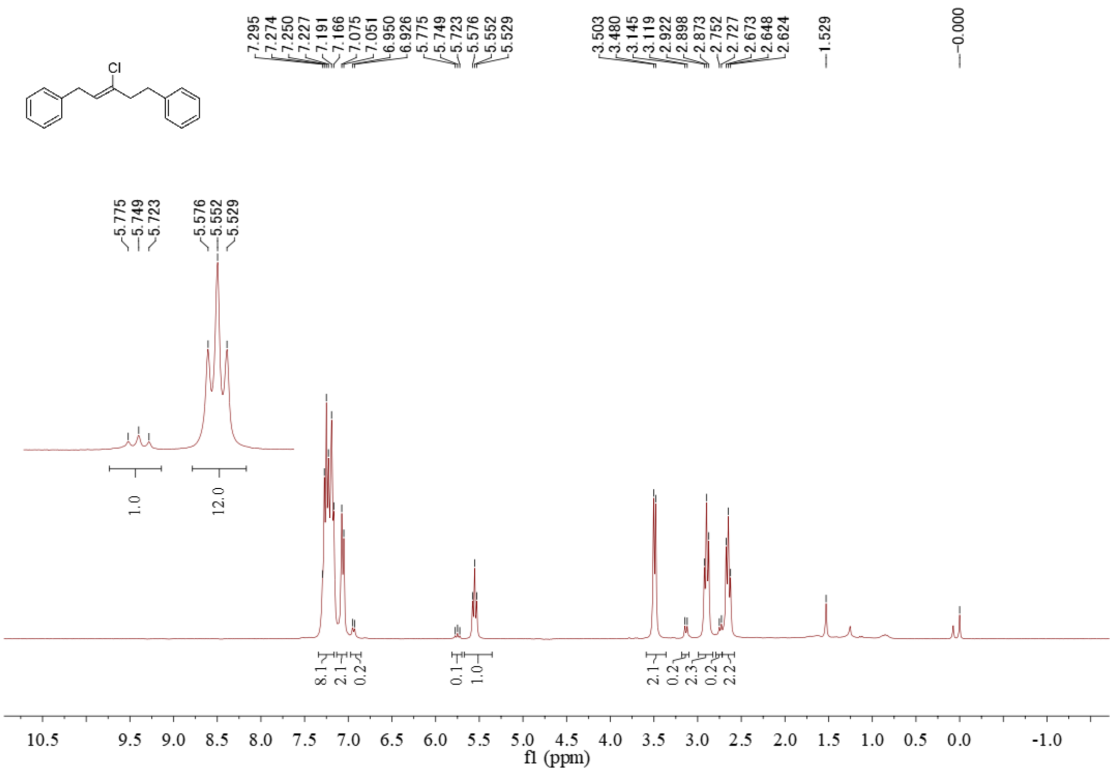

Figure S249. ^{1}H-NMR spectrum of **3y**, related to **Table 2**

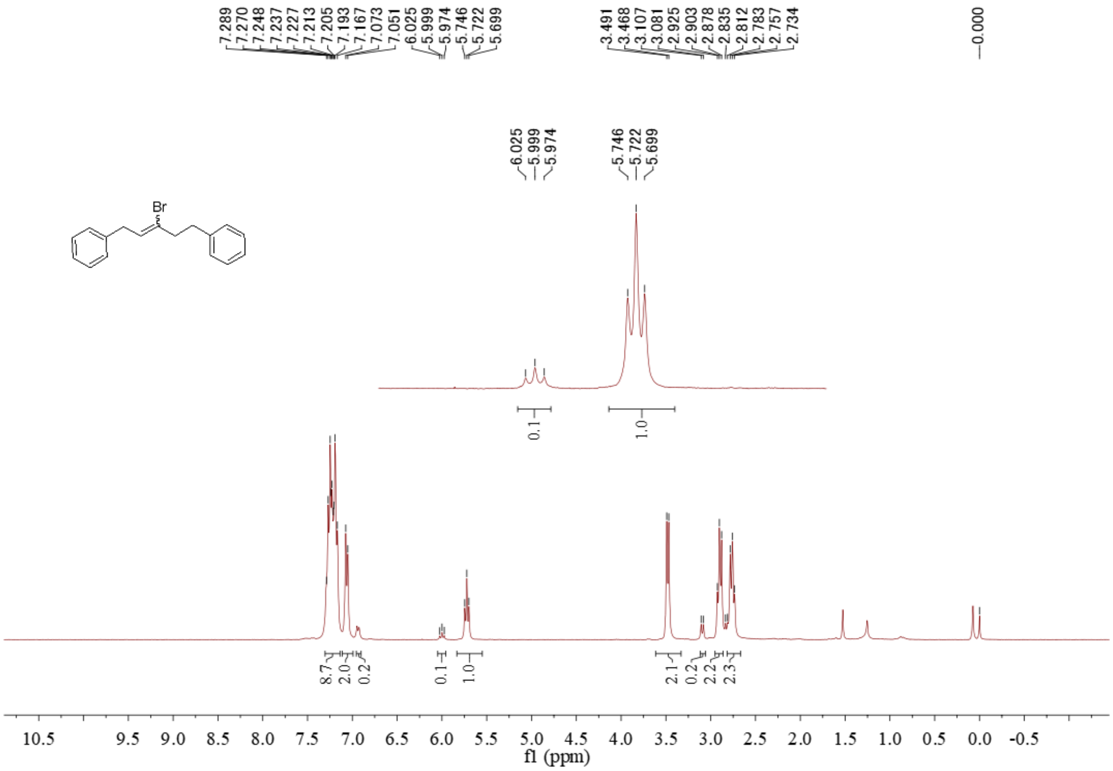

Figure S250. ^{13}C-NMR spectrum of **3y**, related to **Table 2**

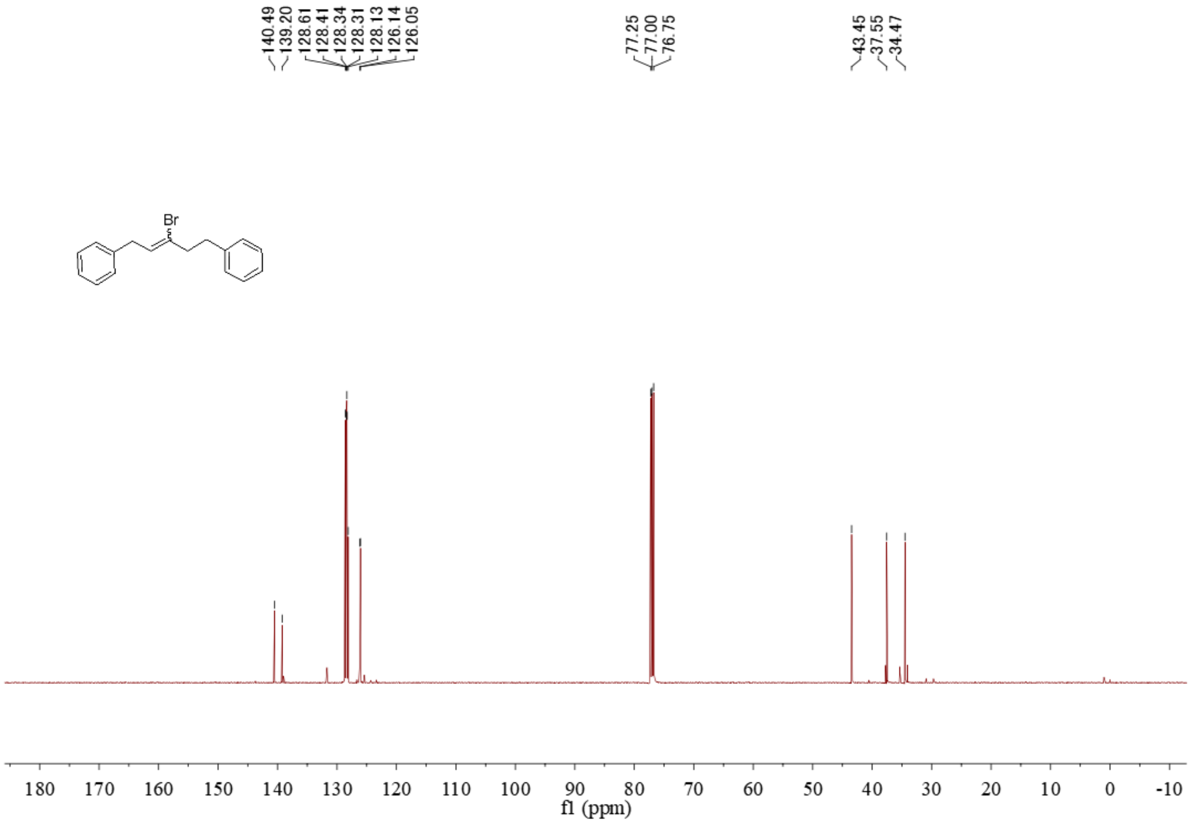

Figure S251. ^{1}H-NMR spectrum of crude reaction mixture of (3,3-dichloropentane-1,5-diyl)dibenzene (**1x**) and $(CF_3)_2CHOH$, related to **Table 2.**

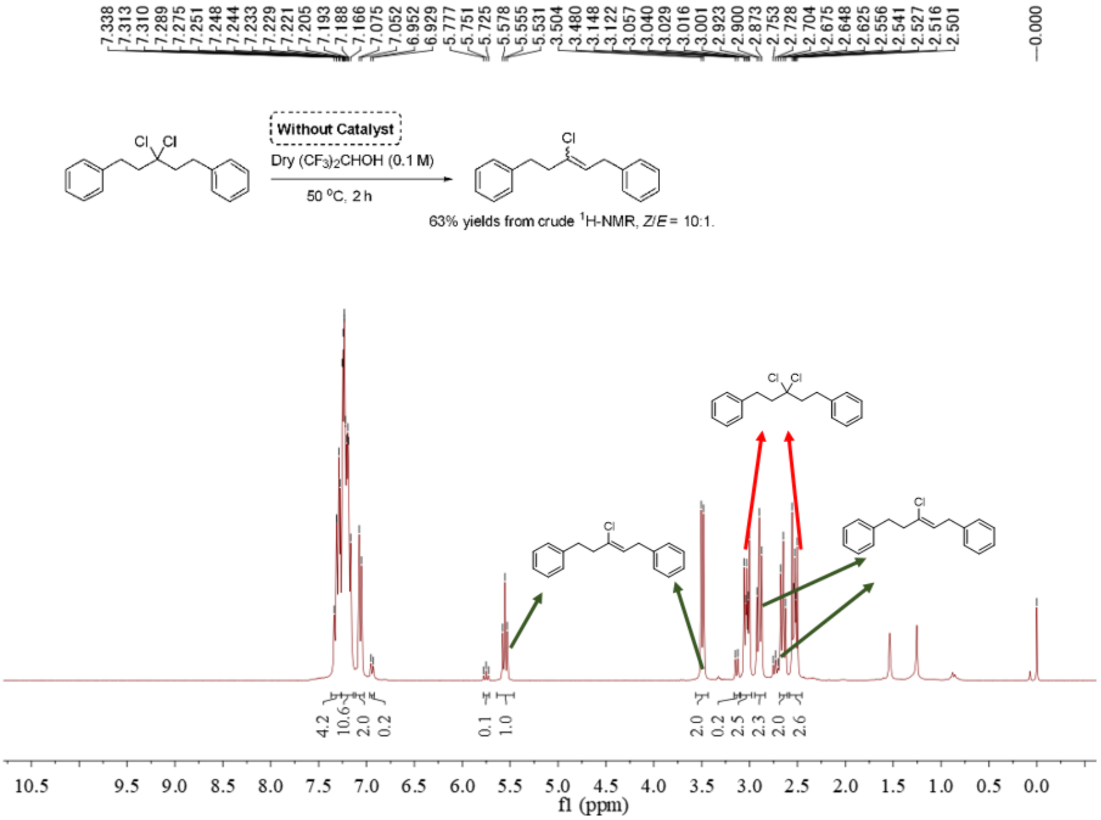

Figure S252. ^{1}H-NMR spectrum of crude reaction mixture of (3,3-dibromopentane-1,5-diyl)dibenzene (**1y**) and $(CF_3)_2CHOH$, related to **Table 2.**

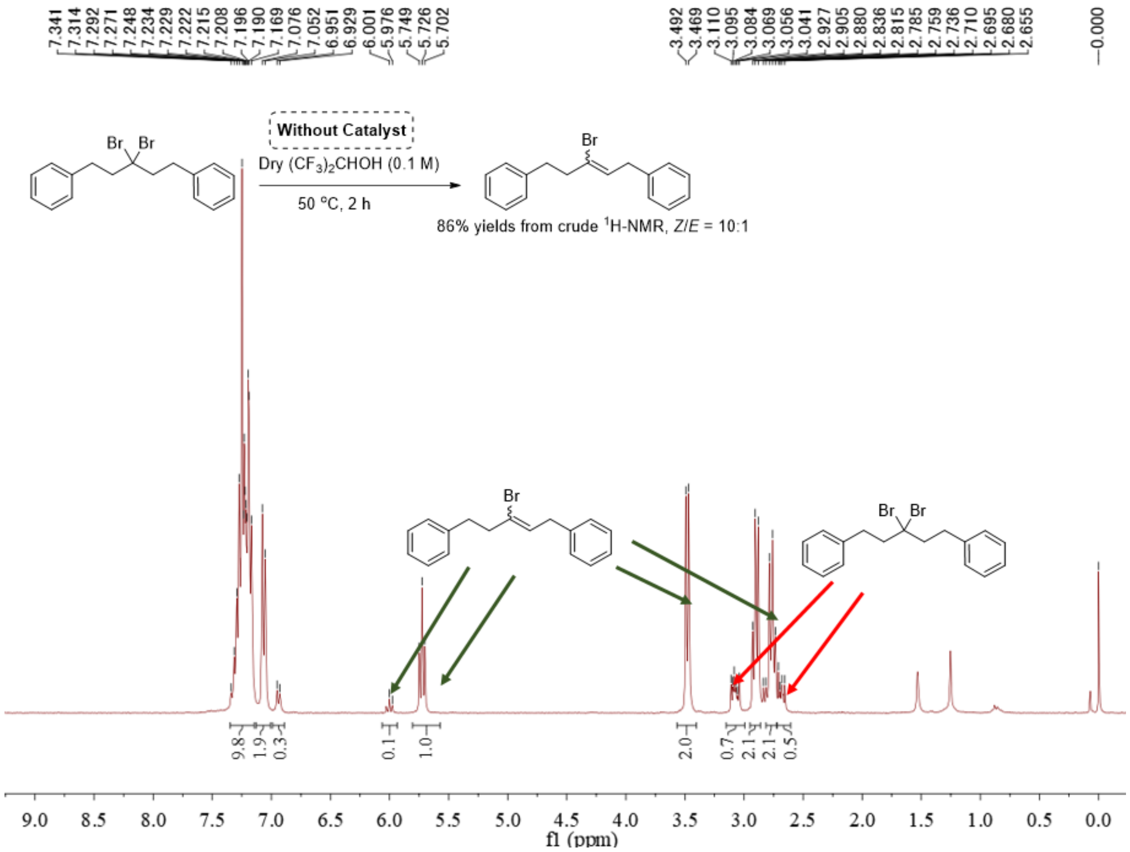

Figure S253 ^{1}H-NMR spectra copy of crude reaction mixture of (3,3-dibromopentane-1,5-diyl)dibenzene(**1y**)/B(C_6F_5)$_3$/*p*-$C_6H_4F_2$, related to **Table 2.**

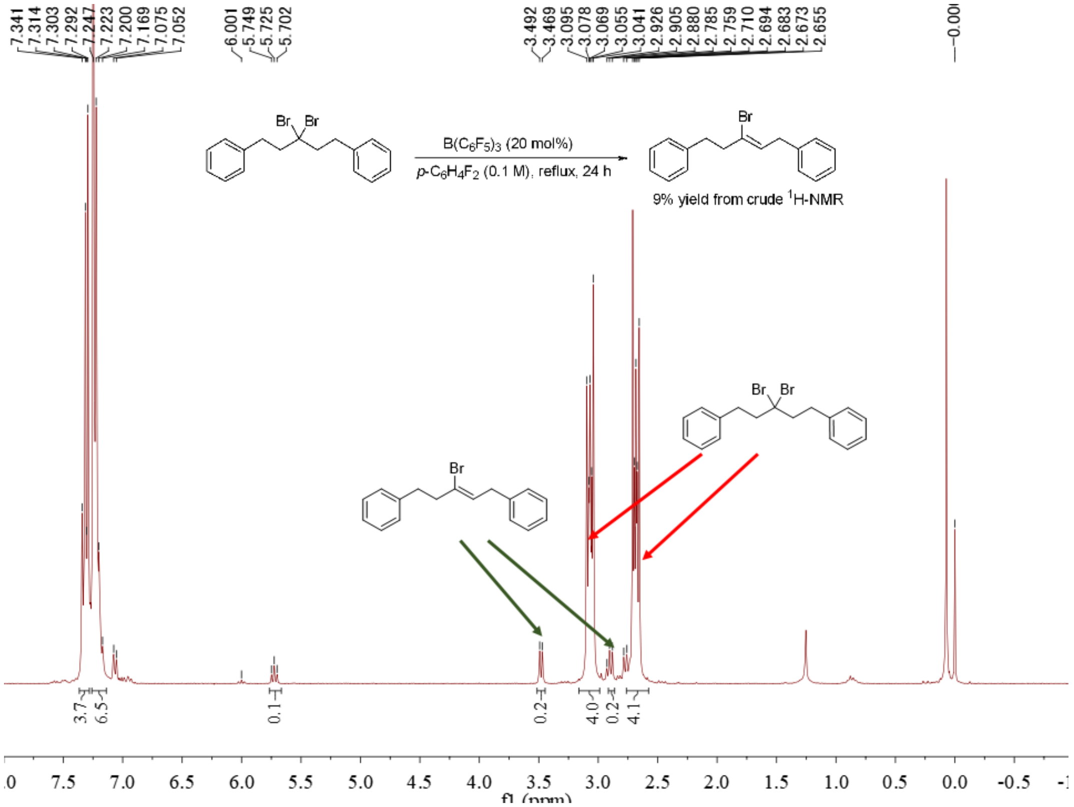

Supplemental Table

Table S1. Optimization of $B(C_6F_5)_3$ induced defluorinative Friedel-Crafts cyclization, related to **Table 1**.

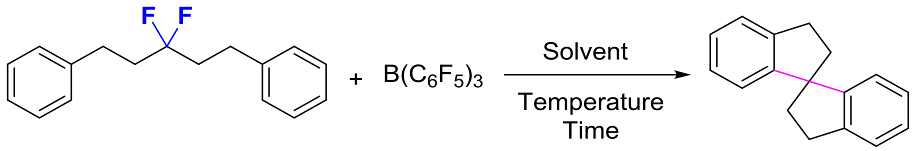

Entry	$B(C_6F_5)_3$ (equiv)	Solvent	Concentration	Temperature (°C)	Time (h)	Yields (%)
1	2.2	CH_2Cl_2	0.1 M	RT	30	85
2	1.1	CH_2Cl_2	0.1 M	RT	30	31
3	0.2	CH_2Cl_2	0.1 M	RT	30	Trace
4	0.2	CH_2Cl_2	0.2 M	100[a]	2	Trace
5	0.2	CH_2Cl_2	2.0 M	100[a]	2	Trace
6	0.5	$MeNO_2$	2.0 M	RT	30	Trace
7	0.2	$(CF_3)_2CHOH$	2.0 M	100[a]	2	54
8	0.2	$(CF_3)_2CHOH$	0.25 M	100[a]	2	53
9	0.1	$(CF_3)_2CHOH$	0.25 M	100[a]	2	41
10	0.05	$(CF_3)_2CHOH$	0.25 M	100[a]	2	25
11	---	$(CF_3)_2CHOH$	0.25 M	100[a]	2	NR
12	0.2	$(CF_3)_2CHOH$/ DCM (1:9)	0.25 M	100[a]	2	Trace
13	0.2	$(CF_3)_2CHOH$	0.125 M	100[a]	2	71
14	0.2	$(CF_3)_2CHOH$ H_2O (2.2 equiv)	0.125 M	100[a]	2	0[b]
15	0.2	$(CF_3)_2CHOH$	0.125 M	50	2	75
16	0.2	$(CF_3)_2CHOH$	0.1 M	50	2	77
17	0.2	$(CF_3)_2CHOH$	0.1 M	RT	17	28
18	0.1	$(CF_3)_2CHOH$	0.1 M	50	20	16
19	0.2	$(CF_3)_2CHOH$	0.05 M	50	2	84
20	0.2	$(CF_3)_2CHOH$	0.05 M	50[c]	2	83
21	0.2	$(CF_3)_2CHOH$[d]	0.05 M	50	2	27[e]
22	0.2	$(CF_3)_2CHOH$ H_2O (2.2 equiv)	0.05 M	50	12	0[b]
23	---	$(CF_3)_2CHOH$	0.05 M	50	2	NR
24	0.2	Solkane-365	0.05 M	50	2	NR
25	0.2	iPrOH	0.05 M	50	2	NR
26	0.2	1,4-dioxane	0.05 M	50	2	NR
27	0.2	CF_3CH_2OH	0.05 M	50	2	NR
28	0.2	$(CF_3)_2PhOH$	0.05 M	100	2	NR

[a]Sealed tube. [b] The hydrolysis product 1,5-diphenylpentan-3-one was obtained in quantitative yield. [c]The reaction was conducted under microwave conditions. [d]$(CF_3)_2CHOH$ was used as purchased, without any precaution to exclude moisture. [e]1,5-diphenylpentan-3-one was observed as major product.

Table S2. Optimization of conditions for the synthesis of monofluoroalkenes, related to **Table 1.**

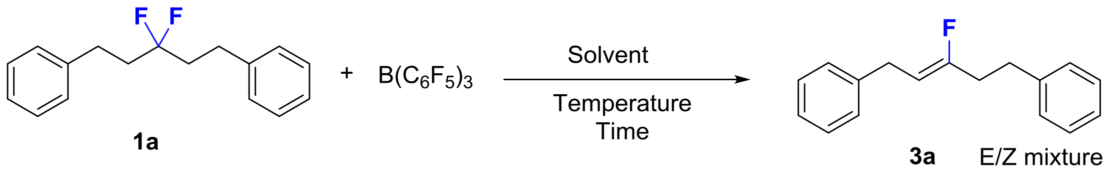

Entry	$B(C_6F_5)_3$ (equiv)	Solvent	Concentration	Temperature (°C)	Time (h)	Yield[a] (%)	*Z*/*E*[a]
1	0.2	*o*-$C_6H_4Cl_2$	0.1 M	100	3	45	5.9:1
2	0.2	*o*-$C_6H_4Cl_2$	0.1 M	160	3	70	6.9:1
3	0.1	*o*-$C_6H_4Cl_2$	0.1 M	160	3	30	6.3:1
4	0.2	*o*-$C_6H_4Cl_2$	0.1 M	160	6	64	6.2:1
5	---	*o*-$C_6H_4Cl_2$	0.1 M	160	3	NR	---
6	0.2	*o*-$C_6H_4Cl_2$	0.25 M	180[b]	3	67	5.9:1
7	0.2	*o*-$C_6H_4Cl_2$	0.25 M	160	3	52	6.3:1
8	0.1	*o*-$C_6H_4Cl_2$	0.25 M	160	3	43	7.5:1
9	0.2	*o*-$C_6H_4Cl_2$	0.05 M	160	3	71	5.6:1
10	0.2	*o*-$C_6H_4Cl_2$	0.1 M	220[c]	3	81	7.3:1
11	0.2	*m*-$C_6H_4Cl_2$	0.1 M	160	3	13	---
12	0.2	Nitrobenzene	0.1 M	160	3	23	6.5:1
14	0.2	DMF	0.1 M	reflux	3	NR	---
15	0.2	DMSO	0.1 M	160	3	NR	---
16	0.2	*o*-$C_6H_4F_2$	0.1 M	reflux	3	75	6.9:1
17	0.2	*o*-$C_6H_4F_2$	0.1 M	reflux	24	87	7.1:1

[a]Determined by ^{19}F NMR analysis using $PhCF_3$ as the internal standard. [b]The reaction was conducted under microwave conditions. [c] Sealed tube.

Transparent Methods

General information

All reactions were performed in oven-dried and flame-dried glassware (10 mL) under a positive pressure of argon atmosphere unless mentioned otherwise. Solvents were transferred *via* syringe and were introduced into the reaction vessels through a rubber septum. All of the reactions were monitored by thin-layer chromatography (TLC) carried out on 0.25 mm Merck silica gel (60-F254). The TLC plates were visualized with UV light and 7% phosphomolybdic acid or $KMnO_4$ in ethanol/heat. Column chromatography was carried out on a column packed with silica gel (60N spherical neutral size 63-210 μm). The ^{1}H-NMR (300 MHz), ^{19}F-NMR (282 MHz), ^{13}C-NMR (125 MHz or 75 MHz) spectra for solution in $CDCl_3$ were recorded on a Buruker Avance 500, a Varian Mercury 300 spectrometers. Chemical shifts (δ) are expressed in ppm downfield from internal TMS (δ = 0.00) for ^{1}H-NMR. C_6F_6 [δ = −162.2 ($CDCl_3$)] was used as an internal standard for ^{19}F-NMR. Mass spectra were recorded on a SHIMAZU LCMS-2010EV (ESI-MS and APCI-MS) and SHIMADZU GCMS-QP5050A (EI-MS) using GC capillary column HYDRODEX-β-TBDAc (length: 25 m, i.d.: 0.25 mm). Helium was used as a carrier gas. Initial temperature: 50 ^{o}C, increase temperature at a rate: 40 ^{o}C/min until final temperature (230 ^{o}C), hold temperature for 15 min at 230 ^{o}C. Solvent delay: 3.0 minutes.High resolution mass spectrometry (HRMS) was recorded on a Waters, GCT Premier (EI-MS) with a TOF analyzer. Infrared spectra were recorded on a JASCO FT/IR-4100 spectrometer. Melting points were recorded on a BUCHI M-565.

Super dehydrated solvents such as CH_2Cl_2, 1,4-dioxane and 1,2-dichlorobenznene (water max 0.001%) were purchased from Wako Pure Chemical Industries, Ltd. and used under argon atmosphere. 1,4-difluorobenzene and 1,1,1,3,3,3-hexafluoropropan-2-ol was purchased from Tokyo Chemical Industry Co., Ltd., and were dried and distilled from 4Å molecule sieves under argon atmosphere, and were stored in glove box. Tis(pentafluorophenyl)borane was purchased from Tokyo Chemical Industry Co., Ltd. (>98.0%, stored under Ar), and was used and stored in glove box with argon atmosphere.

Experimental Procedures

The preparation of spirobiindanes 2a-2t, related to Figure 2.

General procedure for the intramolecular Friedel-Craft reaction of *gem*-difluoroalkanes: In a flame-dried test tube (10 mL), *gem*-difluoroalkanes **1** (0.1 mmol) were added to a solution of $B(C_6F_5)_3$ (20 mol%) in dry HFIP (2.0 mL) at room temperature in a glovebox filled with argon. Subsequently, the tube was sealed with a rubber septum, removed from the glovebox and stirred at 50 ^{o}C for 2-4 h under a positive pressure of argon with a balloon. The resulting mixture was allowed to cool to room temperature and washed with water, extracted with CH_2Cl_2, dried over Na_2SO_4, filtered, and then concentrated *in vacuo*. The residue was purified by column chromatography on silica gel using *n*-hexane as the eluent to afford the desired spirobiindanes **2a**-**2t** in good yields.

2,2',3,3'-Tetrahydro-1,1'-spirobi[indene] **2a**

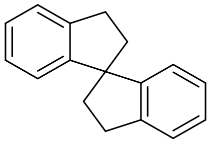

(3,3-Difluoropentane-1,5-diyl)dibenzene **1a** (26.0 mg, 0.1 mmol) was added to a solution of tris(pentafluorophenyl)borane (10.1 mg, 20 mol%) in dry $(CF_3)_2CHOH$ (2.0 ml). And the resulting mixture was stirred at 50 ^{o}C for 2 h under argon atmosphere. The purification by column chromatography on silica gel (*n*-hexane) to give **2a** (18.8 mg, 84%) as a colorless oil. ^{1}H NMR ($CDCl_3$, 300 MHz) δ 7.34–7.24 (m, 2H), 7.24–7.08 (m, 4H), 6.99–6.86 (m, 2H), 3.10–2.94 (m, 4H), 2.34-2.23 (m, 2H), 2.25–2.10 (m, 2H). ^{13}C NMR (126 MHz, $CDCl_3$) δ 150.4, 143.7, 126.65, 126.63, 124.3, 123.4, 60.7, 40.5, 30.8. MS (EI, *m*/*z*) 220 $[M]^+$

6,6'-Dimethyl-2,2',3,3'-tetrahydro-1,1'-spirobi[indene] **2b**

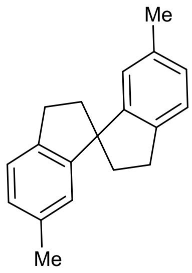

4,4'-(3,3-Difluoropentane-1,5-diyl)bis(methylbenzene) **1b** (28.9 mg, 0.1 mmol) was added to a solution of tris(pentafluorophenyl)borane (10.1 mg, 20 mol%) in dry $(CF_3)_2CHOH$ (2.0 ml). And the resulting mixture was stirred at 50 ^{o}C for 2 h under argon atmosphere. The purification by column chromatography on silica gel (*n*-hexane) to give **2a** (17.3 mg, 69%) as a white solid, mp = 84–86 ^{o}C. ^{1}H NMR (300 MHz, $CDCl_3$) δ 7.19 (d, *J* = 7.6 Hz, 2H), 7.08–6.98 (m, 2H), 6.78 (s, 2H), 2.99 (dd, *J* = 8.0, 6.0 Hz, 4H), 2.39–2.16 (m, 8H), 2.23–2.08 (m, 2H). ^{13}C NMR (126 MHz, $CDCl_3$) δ 150.6, 140.7, 136.2, 127.5, 124.1, 124.0, 60.6, 40.8, 30.5, 21.3. IR (KBr): 2929, 2852, 1490, 1459, 1380, 809 cm^{-1}. HRMS (EI) calcd. for $C_{19}H_{20}^+$ $[M^+]$: 248.1565 found

248.1573.

6,6'-Dimethoxy-2,2',3,3'-tetrahydro-1,1'-spirobi[indene] **2c**

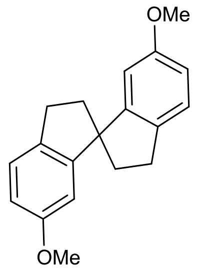

4,4'-(3,3-Difluoropentane-1,5-diyl)bis(methoxybenzene) **1c** (32.0 mg, 0.1 mmol) was added to a solution of tris(pentafluorophenyl)borane (10.1 mg, 20 mol%) in dry $(CF_3)_2CHOH$ (2.0 ml). And the resulting mixture was stirred at 50 °C for 2 h under argon atmosphere. The purification by column chromatography on silica gel (*n*-hexane) to give **2c** (9.7 mg, 27%) as a white solid, mp = 129–131 °C. ^{1}H NMR (300 MHz, $CDCl_3$) δ 7.17 (d, *J* = 8.2 Hz, 2H), 6.75 (dd, *J* = 8.2, 2.5 Hz, 2H), 6.49 (d, *J* = 2.4 Hz, 2H), 3.72 (s, 6H), 2.98–2.83 (m, 4H), 2.34–2.21 (m, 2H), 2.24–1.95 (m, 2H). ^{13}C NMR (126 MHz, $CDCl_3$) δ 159.0, 151.7, 135.7, 124.8, 112.8, 108.7, 61.2, 55.4, 40.9, 30.1. IR (KBr): 2937, 2832, 1614, 1479, 1364, 1284, 821 cm^{-1}. MS (EI, *m*/*z*) 280 [M$^+$]. HRMS (EI) calcd. for $C_{19}H_{20}O_2^+$ [M$^+$]: 280.1463 found 280.1466.

6,6'-Diethyl-2,2',3,3'-tetrahydro-1,1'-spirobi[indene] **2d**

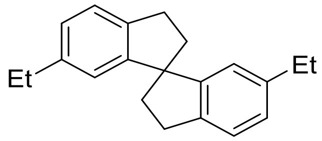

4,4'-(3,3-Difluoropentane-1,5-diyl)bis(ethylbenzene) **1d** (31.5 mg, 0.1 mmol) was added to a solution of tris(pentafluorophenyl)borane (10.1 mg, 20 mol%) in dry $(CF_3)_2CHOH$ (2.0 ml). And the resulting mixture was stirred at 50 °C for 2 h under argon atmosphere. The purification by column chromatography on silica gel (*n*-hexane) to give **2d** (17.6 mg, 62%) as a colorless oil. ^{1}H NMR (300 MHz, $CDCl_3$) δ 7.23–7.16 (m, 2H), 7.04 (d, *J* = 7.6 Hz, 2H), 6.79 (s, 2H), 2.98–2.86 (m, 4H), 2.57 (q, *J* = 7.5 Hz, 4H), 2.31–2.03 (m, 4H), 1.17 (t, *J* = 7.6 Hz, 6H). ^{13}C NMR (126 MHz, $CDCl_3$) δ 150.6, 142.8, 141.1, 126.2, 124.0, 122.9, 60.6, 40.7, 30.4, 28.8, 15.9. IR (KBr): 2960, 2933, 2852, 1482, 1463, 1373, 885, 813 cm^{-1}. MS (EI, *m*/*z*) 276 [M$^+$]. HRMS (EI) calcd. for $C_{21}H_{24}^+$ [M$^+$]: 276.1878 found 276.1884.

6,6'-Dibutyl-2,2',3,3'-tetrahydro-1,1'-spirobi[indene] **2e**

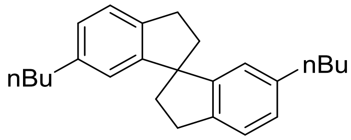

4,4'-(3,3-Difluoropentane-1,5-diyl)bis(butylbenzene) **1e** (37.2 mg, 0.1 mmol) was added to a solution of

tris(pentafluorophenyl)borane (10.1 mg, 20 mol%) in dry $(CF_3)_2CHOH$ (2.0 ml). And the resulting mixture was stirred at 50 °C for 2 h under argon atmosphere. The purification by column chromatography on silica gel (*n*-hexane) to give **2e** (19.7 mg, 59%) as a colorless oil. ^{1}H NMR (300 MHz, $CDCl_3$) δ 7.19 (d, *J* = 7.6 Hz, 2H), 7.05–6.96 (m, 2H), 6.77 (s, 2H), 2.99–2.86 (m, 4H), 2.54–2.46 (m, 4H), 2.37–2.24 (m, 2H), 2.21–2.12 (m, 2H), 1.53–1.44 (m, 4H), 1.30 (dq, *J* = 14.5, 7.2 Hz, 4H), 0.88 (t, *J* = 7.3 Hz, 6H). ^{13}C NMR (126 MHz, $CDCl_3$) δ 150.6, 141.5, 141.1, 126.8, 123.9, 123.5, 60.6, 40.8, 35.6, 34.0, 30.5, 22.5, 13.9. IR (KBr): 2948, 2925, 2860, 1606, 1488, 1454, 1378, 829, 732 cm^{-1}. MS (EI, *m/z*) 332 [M$^+$]. HRMS (EI) calcd. for $C_{25}H_{32}^+$ [M$^+$]: 332.2504 found 332.2519

4,4'-Dimethyl-2,2',3,3'-tetrahydro-1,1'-spirobi[indene] **2f**

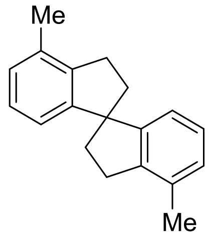

2,2'-(3,3-Difluoropentane-1,5-diyl)bis(methylbenzene) **1f** (28.8 mg, 0.1 mmol) was added to a solution of tris(pentafluorophenyl)borane (10.1 mg, 20 mol%) in dry $(CF_3)_2CHOH$ (2.0 ml). And the resulting mixture was stirred at 50 °C for 2 h under argon atmosphere. The purification by column chromatography on silica gel (*n*-hexane) to give **2f** (22.8 mg, 90%) as a white solid, mp = 89–90 °C. ^{1}H NMR (300 MHz, $CDCl_3$) δ 7.11–6.99 (m, 4H), 6.76 (d, *J* = 7.2 Hz, 2H), 2.95–2.89 (m, 4H), 2.34–2.25 (m, 8H), 2.22–2.04 (m, 2H). ^{13}C NMR (126 MHz, $CDCl_3$) δ 150.3, 142.5, 133.5, 127.4, 126.8, 120.7, 61.1, 40.3, 29.3, 19.1. IR (KBr): 2937, 2848, 1590, 1494, 1376, 782, 765 cm^{-1}. HRMS (EI) calcd. for $C_{19}H_{20}^+$ [M$^+$]: 248.1565 found 248.1567.

4,4'-Dimethoxy-2,2',3,3'-tetrahydro-1,1'-spirobi[indene] **2g**

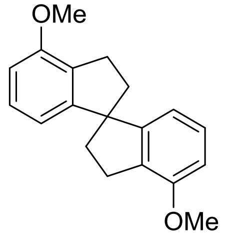

2,2'-(3,3-Difluoropentane-1,5-diyl)bis(methoxybenzene) **1g** (32.0 mg, 0.1 mmol) was added to a solution of tris(pentafluorophenyl)borane (10.1 mg, 20 mol%) in dry $(CF_3)_2CHOH$ (2.0 ml). And the resulting mixture was stirred at 50 °C for 2 h under argon atmosphere. The purification by column chromatography on silica gel (*n*-hexane) to give **2g** (10.5 mg, 37%) as a white solid, mp = 107-109 °C. ^{1}H NMR (300 MHz, $CDCl_3$) δ 7.13 (t, *J* = 7.8 Hz, 2H), 6.71 (d, *J* = 8.1 Hz, 2H), 6.57 (d, *J* = 7.5 Hz, 2H), 3.87 (s, 6H), 2.99–2.81 (m, 4H), 2.30–2.21 (m, 4H). ^{13}C NMR (126 MHz, $CDCl_3$) δ 155.7, 152.3, 131.1, 128.2, 115.8, 108.1, 61.7, 55.2, 40.4, 27.4. IR (KBr): 2952, 2840, 1687, 1463, 1315, 1255, 775 cm^{-1}. HRMS (EI) calcd. for $C_{19}H_{20}O_2^+$ [M$^+$]: 280.1463 found 280.1460.

5,5'-Dimethyl-2,2',3,3'-tetrahydro-1,1'-spirobi[indene] **2h**

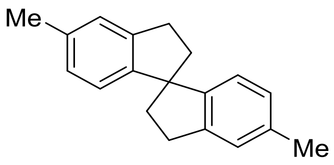

3,3'-(3,3-Difluoropentane-1,5-diyl)bis(methylbenzene) **1h** (28.8mg, 0.1 mmol) was added to a solution of tris(pentafluorophenyl)borane (10.1 mg, 20 mol%) in dry $(CF_3)_2CHOH$ (2.0 ml). And the resulting mixture was stirred at 50 °C for 2 h under argon atmosphere. The purification by column chromatography on silica gel (*n*-hexane) to give **2h** (19.6 mg, 78%) as a colorless oil. The isolated **2h** was obtained with impure isomers (ratio about 9:1, based on integrals of methyl peak in ^{1}H-NMR, and GC-MS analysis). ^{1}H NMR (300 MHz, $CDCl_3$) δ 7.10 (s, 2H), 6.96 (d, *J* = 7.7 Hz, 2H), 6.82 (d, *J* = 7.7 Hz, 2H), 2.99–2.87 (m, 4H), 2.33–2.23 (m, 8H), 2.22–2.16 (m, 2H). ^{13}C NMR (126 MHz, $CDCl_3$) δ 147.6, 143.9, 136.2, 127.4, 125.0, 123.0, 59.9, 40.7, 30.7, 21.2. IR (KBr): 3004, 2940, 2948, 1610, 1490, 1448, 1376, 809, 771 cm^{-1}. MS (EI, *m*/*z*) 248 [M$^+$]. HRMS (EI) calcd. for $C_{19}H_{20}^+$ [M$^+$]:248.1565 found 248. 1577.

4,4'-Difluoro-2,2',3,3'-tetrahydro-1,1'-spirobi[indene] **2i**

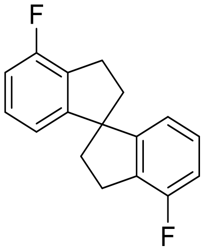

2,2'-(3,3-Difluoropentane-1,5-diyl)bis(fluorobenzene) **1i** (29.6 mg, 0.1 mmol) was added to a solution of tris(pentafluorophenyl)borane (10.1 mg, 20 mol%) in dry $(CF_3)_2CHOH$ (2.0 ml). And the resulting mixture was stirred at 50 °C for 2 h under argon atmosphere. The purification by column chromatography on silica gel (*n*-hexane) to give **2i** (14.9 mg, 57%) as a white solid, mp = 96–97 °C. ^{1}H NMR (300 MHz, $CDCl_3$) δ 7.15–7.08 (m, 2H), 6.96–6.86 (m, 2H), 6.70 (d, *J* = 7.5 Hz, 2H), 3.13–2.97 (m, 4H), 2.35 (ddd, *J* = 11.6, 7.5, 2.0 Hz, 2H), 2.26–2.16 (m, 2H). ^{13}C NMR (126 MHz, $CDCl_3$) δ 159.19 (d, *J* = 246.6 Hz), 153.50 (d, *J* = 5.6 Hz), 129.55 (d, *J* = 18.3 Hz), 128.75 (d, *J* = 6.9 Hz), 118.96 (d, *J* = 3.3 Hz), 113.43 (d, *J* = 20.6 Hz), 61.6, 40.5, 26.70. IR (KBr): 2944, 1614, 1585, 1455, 1241 cm^{-1}. HRMS (EI) calcd. for $C_{17}H_{14}F_2^+$ [M$^+$]: 256.1064 found 256.1057.

4,4'-Dibromo-2,2',3,3'-tetrahydro-1,1'-spirobi[indene] **2j**

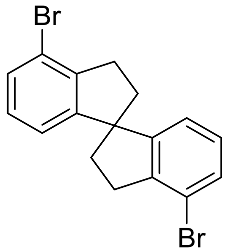

2,2'-(3,3-Difluoropentane-1,5-diyl)bis(bromobenzene) **1j** (41.8 mg, 0.1 mmol) was added to a solution of tris(pentafluorophenyl)borane (10.1 mg, 20 mol%) in dry $(CF_3)_2CHOH$ (2.0 ml). And the resulting mixture was stirred at 50 °C for 2 h under argon atmosphere. The purification by column chromatography on silica gel (*n*-hexane) to give **2j** (30.1 mg, 79%) as a white solid 100-102 °C. ^{1}H NMR (300 MHz, $CDCl_3$) δ 7.37–7.31 (m, 2H), 7.03 (t, *J* = 7.7 Hz, 2H), 6.85 (d, *J* = 7.5 Hz, 2H), 3.07–2.99 (m, 4H), 2.33–2.25 (m, 2H), 2.24–2.14 (m, 2H). ^{13}C NMR (126 MHz, $CDCl_3$) δ 151.9, 143.9, 130.1, 128.7, 122.2, 119.9, 63.2, 39.8, 32.3. IR (KBr): 2940, 1565, 1442, 1307, 775, 678 cm^{-1}. MS (EI, *m*/*z*) 375 [M$^+$]. HRMS (EI) calcd. for $C_{17}H_{14}Br_2^+$ [M$^+$]: 375.9462 found 375. 9452

4,4'-Dichloro-2,2',3,3'-tetrahydro-1,1'-spirobi[indene] **2k**

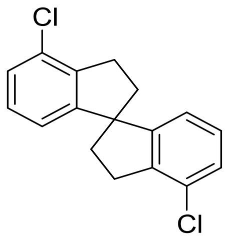

2,2'-(3,3-Difluoropentane-1,5-diyl)bis(chlorobenzene) **1k** (32.9 mg, 0.1 mmol) was added to a solution of tris(pentafluorophenyl)borane (10.1 mg, 20 mol%) in dry $(CF_3)_2CHOH$ (2.0 ml). And the resulting mixture was stirred at 50 °C for 2 h under argon atmosphere. The purification by column chromatography on silica gel (*n*-hexane) to give **2k** (23.3 mg, 77%) as a white solid, mp = 116-118 °C. ^{1}H NMR (300 MHz, $CDCl_3$) δ 7.19 (d, *J* = 7.9 Hz, 2H), 7.09 (t, *J* = 7.6 Hz, 2H), 6.80 (d, *J* = 7.4 Hz, 2H), 3.15–2.96 (m, 4H), 2.34 (ddd, *J* = 11.7, 7.3, 4.2 Hz, 2H), 2.26–2.17 (m, 2H). ^{13}C NMR (126 MHz, $CDCl_3$) δ 151.9, 141.7, 130.6, 128.4, 126.9, 121.6, 62.6, 39.9, 30.1. IR (KBr): 2937, 2844, 1590, 1459, 1415, 1099, 817, 725 cm^{-1}. HRMS (EI) calcd. for $C_{17}H_{14}Cl_2^+$ [M$^+$]: 288.0473 found 288.0481.

6,6'-Dibromo-2,2',3,3'-tetrahydro-1,1'-spirobi[indene] **2l**

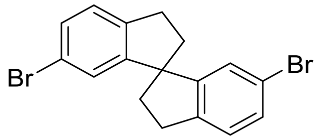

4,4'-(3,3-Difluoropentane-1,5-diyl)bis(bromobenzene) **1l** (41.9 mg, 0.1 mmol) was added to a solution of tris(pentafluorophenyl)borane (10.1 mg, 20 mol%) in dry $(CF_3)_2CHOH$ (2.0 ml). And the resulting mixture

was stirred at 50 °C for 2 h under argon atmosphere. The purification by column chromatography on silica gel (*n*-hexane) to give **2l** (24.4 mg, 64%) as a white solid, mp = 142–144 °C. ^{1}H NMR (300 MHz, $CDCl_3$) δ 7.32 (dd, *J* = 8.0, 1.8 Hz, 2H), 7.15 (d, *J* = 8.0 Hz, 2H), 7.02 (d, *J* = 1.5 Hz, 2H), 2.96 (dd, *J* = 8.2, 6.0 Hz, 4H), 2.32–2.23 (m, 2H), 2.24–2.16 (m, 2H). ^{13}C NMR (126 MHz, $CDCl_3$) δ 152.0, 142.5, 129.9, 126.4, 126.0, 120.4, 60.8, 40.6, 30.3. IR (KBr): 2944, 2840, 1583, 1479, 1396, 1064, 809, 638 cm^{-1}. HRMS (EI) calcd. for Chemical Formula: $C_{17}H_{14}Br_2^+$ [M$^+$]: 375.9462 found 375.9454.

6,6'-Dichloro-2,2',3,3'-tetrahydro-1,1'-spirobi[indene] **2m**

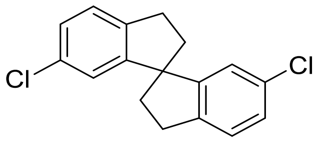

4,4'-(3,3-Difluoropentane-1,5-diyl)bis(chlorobenzene) **1m** (32.9 mg, 0.1 mmol) was added to a solution of tris(pentafluorophenyl)borane (10.1 mg, 20 mol%) in dry $(CF_3)_2CHOH$ (2.0 ml). And the resulting mixture was stirred at 50 °C for 2 h under argon atmosphere. The purification by column chromatography on silica gel (*n*-hexane) to give **2m** (18.9 mg, 65%) as a white solid, mp = 116–118 °C. ^{1}H NMR (300 MHz, $CDCl_3$) δ 7.25–7.14 (m, 4H), 6.87 (d, *J* = 1.3 Hz, 2H), 2.97 (dd, *J* = 8.4, 5.9 Hz, 4H), 2.35–2.14 (m, 4H). ^{13}C NMR (126 MHz, $CDCl_3$) δ 151.6, 141.9, 132.4, 127.1, 125.5, 123.5, 60.8, 40.6, 30.2. IR (KBr): 2937, 2844, 1590, 1415, 1459, 1099, 877, 725 cm^{-1}. MS (EI, *m/z*) 288 [M$^+$]. HRMS (EI) calcd. for Chemical Formula: $C_{17}H_{14}Cl_2^+$ [M$^+$]: 288.0473 found 288.0476.

4,4',6,6'-Tetramethyl-2,2',3,3'-tetrahydro-1,1'-spirobi[indene] **2n**

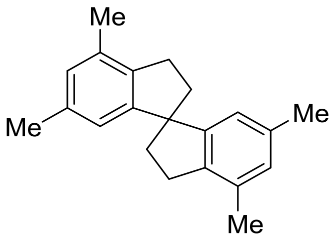

4,4'-(3,3-Difluoropentane-1,5-diyl)bis(1,3-dimethylbenzene) **1n** (31.6 mg, 0.1 mmol) was added to a solution of tris(pentafluorophenyl)borane (10.1 mg, 20 mol%) in dry $(CF_3)_2CHOH$ (2.0 ml). And the resulting mixture was stirred at 50 °C for 2 h under argon atmosphere. The purification by column chromatography on silica gel (*n*-hexane) to give **2n** (26.5 mg, 95%) as a white solid, mp = 149–150 °C. ^{1}H NMR (300 MHz, $CDCl_3$) δ 6.84 (s, 2H), 6.59 (s, 2H), 2.87 (t, *J* = 7.6 Hz, 4H), 2.28 (s, 6H), 2.25–1.94 (m, 10H). ^{13}C NMR (126 MHz, $CDCl_3$) δ 150.6, 139.5, 136.4, 133.2, 128.4, 121.3, 60.9, 40.6, 28.9, 21.2, 19.0. IR (KBr): 2917, 2848, 1594, 1448, 1471, 1376 cm^{-1}. MS (EI, *m/z*) 276 [M]$^+$. HRMS (EI) calcd. for $C_{21}H_{24}^+$ [M$^+$]: 276.1878 found 276.1886.

4,4',5,5'-Tetramethyl-2,2',3,3'-tetrahydro-1,1'-spirobi[indene] **2o**

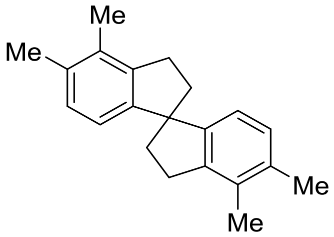

3,3'-(3,3-Difluoropentane-1,5-diyl)bis(1,2-dimethylbenzene) **1o** (31.6 mg, 0.1 mmol) was added to a solution of tris(pentafluorophenyl)borane (10.1 mg, 20 mol%) in dry $(CF_3)_2CHOH$ (2.0 ml). And the resulting mixture was stirred at 50 ^oC for 2 h under argon atmosphere. The purification by column chromatography on silica gel (*n*-hexane) to give **2o** (23.7 mg, 85%) as a white solid, mp = 140–141 ^oC. ^1H NMR (300 MHz, $CDCl_3$) δ 6.95 (d, *J* = 7.6 Hz, 2H), 6.69 (d, *J* = 7.6 Hz, 2H), 2.93 (dd, *J* = 11.6, 6.0 Hz, 4H), 2.25–2.24 (m, 8H), 2.22 (s, 6H), 2.19–2.10 (m, 2H). ^{13}C NMR (126 MHz, $CDCl_3$) δ 148.1, 142.7, 134.6, 132.1, 128.4, 120.4, 61.1, 40.6, 29.7, 19.6, 15.9. IR (KBr): 2996, 2933, 2857, 1605, 1475, 1452, 1373 cm^{-1}. HRMS (EI) calcd. for $C_{21}H_{24}^+$ $[M^+]$: 276.1878 found 276.1879.

2,2',3,3'-Tetrahydro-1,1'-spirobi[cyclopenta[b]naphthalene] **2p**

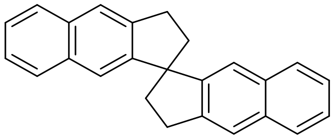

2,2'-(3,3-Difluoropentane-1,5-diyl)dinaphthalene **1p** (36.0 mg, 0.1 mmol) was added to a solution of tris(pentafluorophenyl)borane (10.1 mg, 20 mol%) in dry $(CF_3)_2CHOH$ (2.0 ml). And the resulting mixture was stirred at 50 ^oC for 2 h under argon atmosphere. The purification by column chromatography on silica gel (*n*-hexane) to give **2l** (28.6 mg, 84%) as a white solid. M.p 49-51 ^oC ^1H NMR (300 MHz, $CDCl_3$) δ 7.82–7.75 (m, 4H), 7.65 (d, *J* = 7.8 Hz, 2H), 7.38–7.25 (m, 6H), 3.24–3.14 (m, 4H), 2.47–2.33 (m, 4H). ^{13}C NMR (126 MHz, $CDCl_3$) δ 149.8, 143.0, 133.2, 133.1, 127.8, 127.4, 125.1, 124.9, 122.3, 121.6, 59.9, 41.3, 30.5. IR (KBr): 2933, 2848, 1598, 1448, 1259, 750 cm^{-1}. MS (EI, m/z) 320 $[M^+]$ HRMS (EI) calcd. for $C_{25}H_{20}^+$ $[M^+]$: 320.1565 found 320.1568.

4,6-Dimethyl-2,2',3,3'-tetrahydro-1,1'-spirobi[indene] **2q**

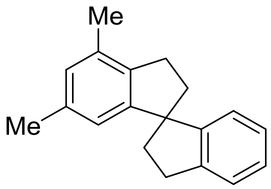

1-(3,3-Difluoro-5-phenylpentyl)-2,4-dimethylbenzene **1q** (28.8 mg, 0.1 mmol) was added to a solution of tris(pentafluorophenyl)borane (10.1 mg, 20 mol%) in dry $(CF_3)_2CHOH$ (2.0 ml). And the resulting mixture

was stirred at 50 °C for 2 h under argon atmosphere. The purification by column chromatography on silica gel (*n*-hexane) to give **2q** (10.8 mg, 42%) as a colorless oil. ^{1}H NMR (300 MHz, $CDCl_3$) δ 7.28 (d, *J* = 6.6 Hz, 1H), 7.25–7.14 (m, 2H), 6.95 (d, *J* = 6.9 Hz, 1H), 6.85 (s, 1H), 6.58 (s, 1H), 3.03–2.85 (m, 4H), 2.36–2.24 (m, 5H), 2.23 (s, 3H), 2.20–2.12 (m, 2H). ^{13}C NMR (126 MHz, $CDCl_3$) δ 150.7, 150.4, 143.8, 139.6, 136.6, 133.3, 128.6, 126.6, 126.5, 124.3, 123.5, 121.3, 60.9, 40.7, 40.4, 30.9, 29.1, 21.2, 19.1. IR (KBr): 2937, 2852, 1610, 1479, 1463, 850, 754, 730 cm^{-1}. MS (EI, m/z) 248 [M$^+$]. HRMS (EI) calcd. for $C_{19}H_{20}^+$ [M$^+$]: 248.1565 found 248.1567.

4-Bromo-4'-methyl-2,2',3,3'-tetrahydro-1,1'-spirobi[indene] **2r**

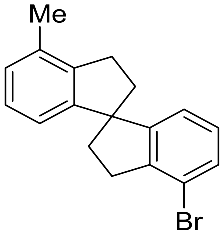

1-Bromo-2-(3,3-difluoro-5-(o-tolyl)pentyl)benzene **1r** (35.3 mg, 0.1 mmol) was added to a solution of tris(pentafluorophenyl)borane (10.1 mg, 20 mol%) in dry $(CF_3)_2CHOH$ (2.0 ml). And the resulting mixture was stirred at 50 °C for 2 h under argon atmosphere. The purification by column chromatography on silica gel (*n*-hexane) to give **2r** (24.3 mg, 69%) as a white solid, mp = 83–85 °C. ^{1}H NMR (300 MHz, $CDCl_3$) δ 7.34 (d, *J* = 7.8 Hz, 1H), 7.10–7.03 (m, 3H), 6.85 (d, *J* = 7.4 Hz, 1H), 6.76 (d, *J* = 7.1 Hz, 1H), 3.06–2.98 (m, 2H), 2.98–2.90 (m, 2H), 2.32–2.21 (m, 5H), 2.26–2.16 (m, 2H). ^{13}C NMR (126 MHz, $CDCl_3$) δ 152.7, 149.6, 143.9, 142.3, 133.7, 129.6, 128.5, 127.7, 127.0, 122.3, 120.6, 119.8, 62.2, 40.3, 39.5, 32.2, 29.3, 19.1. IR (KBr): 3075, 2932, 2848, 1598, 1486, 1438, 750, 615 cm^{-1}. HRMS (EI) calcd. for $C_{18}H_{17}Br^+$ [M$^+$]: 312.0514 found 312.0517.

3,3',4,4'-Tetrahydro-2H,2'H-1,1'-spirobi[naphthalene] **2s**

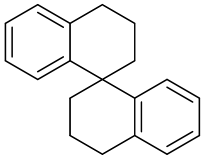

(4,4-Difluoroheptane-1,7-diyl)dibenzene **1s** (28.8 mg, 0.1 mmol) was added to a solution of tris(pentafluorophenyl)borane (10.1 mg, 20 mol%) in dry $(CF_3)_2CHOH$ (2.0 ml). And the resulting mixture was stirred at 50 °C for 2 h under argon atmosphere. The purification by column chromatography on silica gel (*n*-hexane) to give **2s** (22.7 mg, 90%) as a white solid, mp = 56–58 °C. ^{1}H NMR (300 MHz, $CDCl_3$) δ 7.12–6.99 (m, 6H), 6.77 (d, *J* = 7.5 Hz, 2H), 2.96–2.87 (m, 4H), 2.16–2.10 (m, 2H), 1.94–1.83 (m, 6H). ^{13}C NMR (126 MHz, $CDCl_3$) δ 146.7, 137.1, 130.2, 128.5, 125.7, 125.2, 42.9, 38.8, 30.3, 19.5. IR (KBr): 2952, 2852, 1579, 1479, 1448 cm^{-1}. HRMS (EI) calcd. for $C_{19}H_{20}^+$ [M$^+$]: 248.1565 found 248.1571.

2,3,3',4'-Tetrahydro-2'H-spiro[indene-1,1'-naphthalene] **2t**

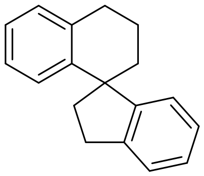

(3,3-Difluorohexane-1,6-diyl)dibenzene **1t** (27.4 mg, 0.1 mmol) was added to a solution of tris(pentafluorophenyl)borane (10.1 mg, 20 mol%) in dry $(CF_3)_2CHOH$ (2.0 ml). And the resulting mixture was stirred at 50 ºC for 2 h under argon atmosphere. The purification by column chromatography on silica gel (*n*-hexane) to give **2t** (21.7 mg, 88%) as a colorless oil. ^1H NMR (300 MHz, $CDCl_3$) δ 7.26 (t, *J* = 6.9 Hz, 2H), 7.08–6.99 (m, 4H), 6.84 (dd, *J* = 14.5, 7.3 Hz, 2H), 2.94–2.84 (m, 4H), 2.36–2.25 (m, 2H), 1.95–1.84 (m, 4H). ^{13}C NMR (126 MHz, $CDCl_3$) δ 152.9, 144.2, 143.6, 137.1, 129.0, 128.6, 126.6, 126.4, 125.8, 125.6, 124.2, 124.2, 52.4, 43.1, 36.2, 30.2, 30.1, 20.6. IR (KBr): 2937, 2840, 1592, 1563, 1450, 1307 cm^{-1}. HRMS (EI) calcd. for $C_{18}H_{18}^+$ [M^+]: 234.1409 found 234.1411.

General procedure for the preparation of monofluoroalkene 3, related to Figure 2.

In a flame-dried test tube, *gem*-difluoroalkanes **1** (0.1 mmol) were added to a solution of $B(C_6F_5)_3$ (20 mol%) in dry 1,4-difluorobenzene (1.0 mL) at room temperature in a glovebox filled with argon. Subsequently, the tube was sealed with a rubber septum, removed from the glovebox and heated to reflux for 24-48 h under a positive pressure of argon with a balloon. The resulting mixture was allowed to cool to room temperature and washed with water, extracted with CH_2Cl_2, dried over Na_2SO_4, filtered, and then concentrated *in vacuo*. The residue was purified by column chromatography on silica gel using *n*-hexane as the eluent to give the desired monofluoroalkene **3**.

(3-Fluoropent-2-ene-1,5-diyl)dibenzene **3a**

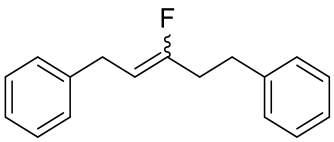

(3,3-Difluoropentane-1,5-diyl)dibenzene **1a** (26.0 mg, 0.1 mmol) was added to a solution of tris(pentafluorophenyl)borane (10.1 mg, 20 mol%) in dry 1,4-difluorobenzene (1.0 ml). And the resulting mixtuure was refluxed for 24 h under argon atmosphere. The purification by column chromatography on silica gel (*n*-hexane) to give **2a** (20.3 mg, 84%) as a colorless oil. The ratio for *Z*/*E* isomers (7.1:1) was determined by ^{19}F-NMR. (*Z*)-**3a**: ^1H NMR (300 MHz, $CDCl_3$) δ 7.28–7.15 (m, 8H), 7.12 (d, *J* = 6.8 Hz, 2H), 4.68 (dt, *J* = 36.8, 7.6 Hz, 1H), 3.41 (d, *J* = 7.5 Hz, 2H), 2.87–2.81 (m, 2H), 2.53 (dt, *J* = 16.2, 6.1 Hz, 2H); ^{13}C NMR (126 MHz, $CDCl_3$) δ 159.0 (d, *J* = 254.5 Hz), 140.7, 140.5 (d, *J* = 1.7 Hz), 128.4, 128.4, 128.3, 128.2, 126.1, 125.9, 104.6 (d, *J* = 15.2 Hz), 33.9 (d, *J* = 27.5 Hz), 32.5 (d, *J* = 1.0 Hz), 29.8 (d, *J* = 5.9 Hz); ^{19}F NMR (282 MHz, $CDCl_3$) δ -110.7 (dt, *J* = 36.2, 17.5 Hz, 1F). IR (KBr): 3087, 3023, 2933, 2852, 1710, 1610, 1486, 1452, 1068, 943 cm^{-1}. MS (EI, *m/z*) 240 $[M]^+$. HRMS (EI) calcd. for $C_{17}H_{17}F^+$ [M^+]: 240.1314,

found 240.1325.

4,4'-(3-Fluoropent-2-ene-1,5-diyl)bis(butylbenzene) **3e**

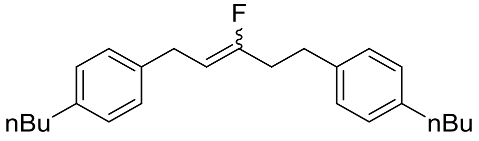

4,4'-(3,3-Difluoropentane-1,5-diyl)bis(butylbenzene) **1e** (37.2, 0.1 mmol) was added to a solution of tris(pentafluorophenyl)borane (10.1 mg, 20 mol%) in dry 1,4-difluorobenzene (1.0 ml). And the resulting mixtuure was refluxed for 24 h under argon atmosphere. The purification by column chromatography on silica gel (*n*-hexane) to give **3e** (24.5 mg, 69%) as a colorless oil. The ratio for *Z*/*E* isomers (10.0:1) was determined by ^{19}F-NMR. (*Z*)-**3e**: ^{1}H NMR (300 MHz, $CDCl_3$) δ 7.18–6.97 (m, 8H), 4.66 (dt, *J* = 36.9, 7.5 Hz, 1H), 3.36 (d, *J* = 7.4 Hz, 2H), 2.80 (t, *J* = 7.7 Hz, 2H), 2.65–2.54 (m, 6H), 1.58–1.51 (m, 4H), 1.35 (dd, *J* = 14.6, 7.3 Hz, 4H), 0.92 (t, *J* = 7.2 Hz, 6H). ^{13}C NMR (126 MHz, $CDCl_3$) δ 159.02 (d, *J* = 254.2 Hz), 140.6, 140.5, 137.9, 137.74 (d, *J* = 1.5 Hz), 128.41, 128.40, 128.3, 128.1, 104.77 (d, *J* = 15.2 Hz), 35.3, 35.2, 34.07 (d, *J* = 27.4 Hz), 33.75 (d, *J* = 2.3 Hz), 32.1, 29.4, 29.3, 22.42 , 22.40, 14.00, 13.98. ^{19}F NMR (282 MHz, $CDCl_3$) δ -111.3 (dt, *J* = 36.9, 17.2 Hz, 1F). IR (KBr): 3012, 2956, 2925, 2857, 1511, 1452, 1378, 1112, 798 cm^{-1}. HRMS (EI) calcd. for $C_{25}H_{33}F^{+}$ [M^{+}]: 352.2566, found 352.2569.

2,2'-(3-Fluoropent-2-ene-1,5-diyl)bis(methylbenzene) **3f**

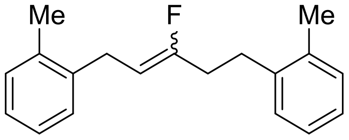

2,2'-(3,3-Difluoropentane-1,5-diyl)bis(methylbenzene) **1f** (28.8, 0.1 mmol) was added to a solution of tris(pentafluorophenyl)borane (10.1 mg, 20 mol%) in dry 1,4-difluorobenzene (1.0 ml). And the resulting mixtuure was refluxed for 24 h under argon atmosphere. The purification by column chromatography on silica gel (*n*-hexane) to give **3f** (17.3 mg, 60%) as a colorless oil. The ratio for *Z*/*E* isomers (9.1:1) was determined by ^{19}F-NMR. (*Z*)-**3f**: ^{1}H NMR (300 MHz, $CDCl_3$) δ 7.23–7.06 (m, 8H), 4.64 (dt, *J* = 36.9, 7.4 Hz, 1H), 3.39 (d, *J* = 7.4 Hz, 2H), 2.84–2.77 (m, 2H), 2.49–2.38 (m, 2H), 2.32 (s, 3H), 2.30 (s, 3H); ^{13}C NMR (126 MHz, $CDCl_3$) δ 159.12 (d, *J* = 254.6 Hz), 138.9, 138.70 (d, *J* = 1.5 Hz), 136.2, 135.9, 130.2, 130.1, 128.8, 128.6, 126.32 (d, *J* = 4.5 Hz), 126.2, 126.07, 126.05, 103.9 (d, *J* = 15.2 Hz), 32.9 (d, *J* = 27.6 Hz), 30.00 (d, *J* = 14.6 Hz), 27.7 (d, *J* = 5.8 Hz), 19.3, 19.2; ^{19}F NMR (282 MHz, $CDCl_3$) δ -110.17 (dt, *J* = 36.2, 17.8 Hz, 1F). IR (KBr): 3056, 2921, 2877, 1710, 1594, 14886, 1255, 1145, 1101, 738 cm^{-1}. MS (EI, *m/z*) 268 $[M]^{+}$. HRMS (EI) calcd. for $C_{19}H_{21}F^{+}$ [M^{+}]: 268.1627, found 268.1633.

2,2'-(3-Fluoropent-2-ene-1,5-diyl)bis(methoxybenzene) **3g**

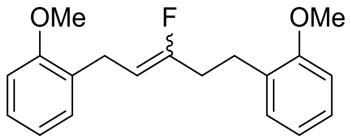

2,2'-(3,3-Difluoropentane-1,5-diyl)bis(methoxybenzene) **1g** (32.0, 0.1 mmol) was added to a solution of tris(pentafluorophenyl)borane (10.1 mg, 20 mol%) in dry 1,4-difluorobenzene (1.0 ml). And the resulting mixtuure was refluxed for 24 h under argon atmosphere. The purification by column chromatography on silica gel (*n*-hexane) to give **3g** (15.9 mg, 53%) as a colorless oil. The ratio for *Z*/*E* isomers (5.8:1) was determined by ^{19}F-NMR. (*Z*)-**3g**: ^{1}H NMR (300 MHz, $CDCl_3$) δ 7.26–7.16 (m, 4H), 6.94–6.72 (m, 4H), 4.69 (dt, *J* = 37.4, 7.5 Hz, 1H), 3.82 (s, 3H), 3.80 (s, 3H), 3.38 (d, *J* = 7.4 Hz, 2H), 2.85–2.79 (m, 2H), 2.51–2.40 (m, 2H). ^{13}C NMR (126 MHz, $CDCl_3$) δ 159.69 (d, *J* = 254.1 Hz), 157.4, 157.1, 130.0, 129.3, 129.2, 128.9, 127.3, 127.0, 120.39, 120.32, 110.13, 110.03, 103.41 (d, *J* = 15.0 Hz), 55.26, 55.16, 32.22 (d, *J* = 27.4 Hz), 27.44 (d, *J* = 1.4 Hz), 24.01 (d, *J* = 6.3 Hz). ^{19}F NMR (282 MHz, $CDCl_3$) δ -110.32 (dt, *J* = 37.4, 17.3 Hz, 1F). IR (KBr): 3019, 2952, 2832, 1702, 1602, 1486, 1459, 1243, 1108, 1025 cm^{-1}. HRMS (EI) calcd. for $C_{19}H_{21}FO_2^+$ [M^+]: 300.1526, found 300.1532.

4,4'-(3-Fluoropent-2-ene-1,5-diyl)bis(1,3-dimethylbenzene) **3n**

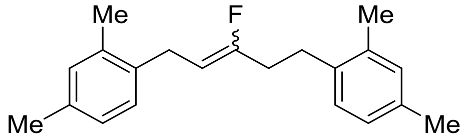

4,4'-(3,3-Difluoropentane-1,5-diyl)bis(1,3-dimethylbenzene) **1n** (31.6, 0.1 mmol) was added to a solution of tris(pentafluorophenyl)borane (10.1 mg, 20 mol%) in dry 1,4-difluorobenzene (1.0 ml). And the resulting mixtuure was refluxed for 24 h under argon atmosphere. The purification by column chromatography on silica gel (*n*-hexane) to give **3g** (15.0 mg, 50%) as a colorless oil. The ratio for *Z*/*E* isomers (9.1:1) was determined by ^{19}F-NMR. (*Z*)-**3n**: ^{1}H NMR (300 MHz, $CDCl_3$) δ 7.03–6.84 (m, 6H), 4.60 (dt, *J* = 37.1, 7.4 Hz, 1H), 3.34 (d, *J* = 7.4 Hz, 2H), 2.81–2.74 (m, 2H), 2.45–2.34 (m, 2H), 2.28 (s, 6H), 2.26 (s, 3H), 2.24 (s, 3H). ^{13}C NMR (126 MHz, $CDCl_3$) δ 159.01 (d, *J* = 254.3 Hz), 136.0, 135.9, 135.68, 135.66, 135.64, 135.63, 131.0, 130.9, 128.7, 128.5, 126.6, 126.5, 103.99 (d, *J* = 15.2 Hz), 32.95 (d, *J* = 27.5 Hz), 29.6, 27.25 (d, *J* = 5.8 Hz), 20.90, 20.89, 19.22, 19.13. ^{19}F NMR (282 MHz, $CDCl_3$) δ -110.37 (dt, *J* = 37.0, 17.7 Hz, 1F). IR (KBr): 3004, 2917, 2869, 1698, 1610, 1496, 1375, 1268, 1089 cm^{-1}. HRMS (EI) calcd. for $C_{21}H_{25}F^+$ [M^+]: 296.1940, found 296.1954.

3,3'-(3-Fluoropent-2-ene-1,5-diyl)bis(1,2-dimethylbenzene) **3o**

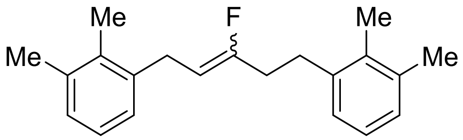

3,3'-(3,3-Difluoropentane-1,5-diyl)bis(1,2-dimethylbenzene) **1o** (31.6, 0.1 mmol) was added to a solution of tris(pentafluorophenyl)borane (10.1 mg, 20 mol%) in dry 1,4-difluorobenzene (1.0 ml). And the resulting mixtuure was refluxed for 24 h under argon atmosphere. The purification by column chromatography on silica gel (*n*-hexane) to give **3o** (20.5 mg, 70%) as a colorless oil. The ratio for *Z*/*E* isomers (8.7:1) was determined by ^{19}F-NMR. (*Z*)-**3o**: ^{1}H NMR (300 MHz, $CDCl_3$) δ 7.02–6.94 (m, 6H), 4.62 (dt, *J* = 37.1, 7.4 Hz, 1H), 3.41 (d, *J* = 7.3 Hz, 2H), 2.86–2.81 (m, 2H), 2.49–2.39 (m, 2H), 2.28 (s, 3H), 2.27 (s, 3H), 2.19 (s, 3H), 2.18 (s, 3H). ^{13}C NMR (126 MHz, $CDCl_3$) δ 158.8 (d, *J* = 254.3 Hz), 138.8, 138.63 (d, *J* = 1.5 Hz), 136.9, 136.84, 134.81, 134.4, 127.99, 127.98, 126.9, 126.6, 125.44, 125.43, 104.14 (d, *J* = 15.1 Hz), 33.16 (d, *J* = 27.5 Hz), 30.8, 28.40 (d, *J* = 5.6 Hz), 20.7, 20.6, 14.9. ^{19}F NMR (282 MHz, $CDCl_3$) δ -109.43 (dt, *J* = 37.1, 17.7 Hz, 1F). IR (KBr): 3016, 2917, 1702, 1583, 1452, 1382, 1132, 732, 779 cm^{-1}. MS (EI, *m/z*) 296 $[M]^+$. HRMS (EI) calcd. for $C_{21}H_{25}F^+$ $[M^+]$: 296.1940, found 296.1949.

2,2'-(3-Fluoropent-2-ene-1,5-diyl)bis(bromobenzene) **3j**

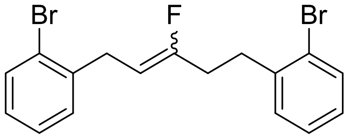

2,2'-(3,3-Difluoropentane-1,5-diyl)bis(bromobenzene) **1j** (41.5, 0.1 mmol) was added to a solution of tris(pentafluorophenyl)borane (10.1 mg, 20 mol%) in dry 1,4-difluorobenzene (1.0 ml). And the resulting mixtuure was refluxed for 48 h under argon atmosphere. The purification by column chromatography on silica gel (*n*-hexane) to give **3j** (15.1 mg, 38%) as a colorless oil. The ratio for *Z*/*E* isomers (25:1) was determined by ^{19}F-NMR. (*Z*)-**3j**: ^{1}H NMR (300 MHz, $CDCl_3$) δ 7.52 (dd, *J* = 7.8, 4.5 Hz, 2H), 7.24–7.02 (m, 6H), 4.69 (dt, *J* = 36.6, 7.5 Hz, 1H), 3.49 (d, *J* = 7.5 Hz, 2H), 3.06–2.93 (m, 2H), 2.62–2.46 (m, 2H); ^{13}C NMR (126 MHz, $CDCl_3$) δ 159.24 (d, *J* = 255.9 Hz), 139.8, 139.78 (d, *J* = 1.8 Hz), 132.8, 132.6, 130.7, 130.1, 127.9, 127.7, 127.4, 124.34, 124.29, 103.31 (d, *J* = 14.8 Hz), 33.1, 32.21 (d, *J* = 27.4 Hz), 30.4 (d, *J* = 5.9 Hz); ^{19}F NMR (282 MHz, $CDCl_3$) δ -108.96 (dt, *J* = 36.4, 18.1 Hz). IR (KBr): 3056, 2925, 1714, 1558, 1463, 1438, 1153, 1022, 754, 659 cm^{-1}. HRMS (EI) calcd. for $C_{17}H_{15}Br_2F^+$ $[M^+]$: 395.9525, found 395. 9547.

2,2'-(3-Fluoropent-2-ene-1,5-diyl)bis(chlorobenzene) **3k**

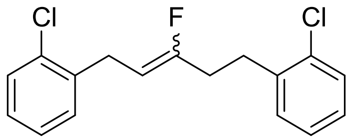

2,2'-(3,3-Difluoropentane-1,5-diyl)bis(chlorobenzene) **1k** (32.9 mg, 0.1 mmol) was added to a solution of tris(pentafluorophenyl)borane (10.1 mg, 20 mol%) in dry 1,4-difluorobenzene (1.0 ml). And the resulting mixtuure was refluxed for 48 h under argon atmosphere. The purification by column chromatography on silica gel (*n*-hexane) to give **3k** (18.1 mg, 55%) as a colorless oil. The ratio for *Z*/*E* isomers (8.6:1) was determined by ^{19}F-NMR. (*Z*)-**3k**: ^{1}H NMR (300 MHz, $CDCl_3$) δ 7.42–7.26 (m, 2H), 7.23–7.04 (m, 6H), 4.68 (dt, *J* = 36.5, 7.6 Hz, 1H), 3.49 (d, *J* = 7.5 Hz, 2H), 2.99–2.85 (m, 2H), 2.52 (dt, *J* = 17.9, 7.6 Hz, 2H). ^{13}C NMR (126 MHz, $CDCl_3$) δ 159.34 (d, *J* = 255.8 Hz), 138.16, 138.11, 138.0, 133.87, 133.85, 130.7, 130.0, 129.5, 129.3, 127.7, 127.4, 126.8, 103.18 (d, *J* = 14.8 Hz), 32.07 (d, *J* = 27.4 Hz), 30.5, 27.72 (d, *J* = 6.1 Hz). ^{19}F NMR (282 MHz, $CDCl_3$) δ -109.22 (dt, *J* = 36.4, 18.1 Hz, 1F). IR (KBr): 3072, 2911, 1706, 1565, 1463, 1438, 1141, 1041, 757 cm^{-1}. HRMS (EI) calcd. for $C_{17}H_{15}Cl_2F^+$ [M^+]: 308.0535, found 308.0558.

4,4'-(3-Fluoropentane-1,5-diyl)bis(fluorobenzene) **3u**

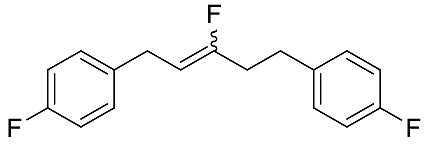

4,4'-(3,3-Difluoropentane-1,5-diyl)bis(fluorobenzene) **1u** (29.6 mg, 0.1 mmol) was added to a solution of tris(pentafluorophenyl)borane (10.1 mg, 20 mol%) in dry 1,4-difluorobenzene (1.0 ml). And the resulting mixtuure was refluxed for 48 h under argon atmosphere. The purification by column chromatography on silica gel (*n*-hexane) to give **3u** (14.9 mg, 52%) as a colorless oil. The ratio for *Z*/*E* isomers (11.1:1) was determined by ^{19}F-NMR. (*Z*)-**3u**: ^{1}H NMR (300 MHz, $CDCl_3$) δ 7.17–7.07 (m, 2H), 7.02–6.86 (m, 6H), 4.60 (dt, *J* = 36.6, 7.7 Hz, 1H), 3.33 (d, *J* = 7.6 Hz, 2H), 2.82 (dd, *J* = 14.6, 7.3 Hz, 2H), 2.52–2.44 (m, 2H). ^{13}C NMR (126 MHz, $CDCl_3$) δ 161.43 (d, *J* = 243.8 Hz), 161.32 (d, *J* = 243.6 Hz), 158.78 (d, *J* = 254.9 Hz), 136.22, 136.19, 136.07 (d, *J* = 3.0 Hz), 136.06 (d, *J* = 2.9 Hz), 129.75 (d, *J* = 41.2 Hz), 129.69 (d, *J* = 41.2 Hz), 115.2, 115.1, 115.08, 115.00, 104.95 (d, *J* = 15.1 Hz), 34.05 (d, *J* = 27.4 Hz), 31.6, 28.95 (d, *J* = 6.0 Hz). ^{19}F NMR (282 MHz, $CDCl_3$) δ -110.75 (dt, *J* = 36.6, 17.7 Hz, 1F), -117.18–-117.34 (m, 1F), -117.43–-117.68 (m, 1F). IR (KBr): 3045, 2929, 2857, 1710, 1606, 1519, 1430, 1153, 1089, 806 cm^{-1}. HRMS (EI) calcd. for $C_{17}H_{15}F_3^+$ [M^+]: 276.1126, found 276.1139.

(2-Fluoroprop-1-ene-1,3-diyl)dibenzene **3aa** (Nahra et al., 2015)

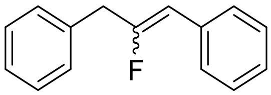

(2,2-Difluoropropane-1,3-diyl)dibenzene **1aa** (23.2 mg, 0.1 mmol) was added to a solution of tris(pentafluorophenyl)borane (10.1 mg, 20 mol%) in dry 1,4-difluorobenzene (1.0 ml). And the resulting mixtuure was refluxed for 24 h under argon atmosphere. The purification by column chromatography on silica gel (*n*-hexane) to give **3aa** (11.0 mg, 50%) as a colorless oil. The ratio for Z/E isomers (16.6:1) was determined by ^{19}F-NMR. (*Z*)-**3aa**: ^{1}H NMR (300 MHz, $CDCl_3$) δ 7.55–7.40 (m, 2H), 7.37–7.17 (m, 8H), 5.52 (d, *J* = 38.8 Hz, 1H), 3.65 (d, *J* = 17.0 Hz, 2H); ^{19}F NMR (282 MHz, $CDCl_3$) δ -100.15 (dt, *J* = 38.7, 17.0 Hz); MS (EI, *m/z*) 212 $[M]^+$

(*Z*)-(1-Fluoroprop-1-ene-1,3-diyl)dibenzene **3bb** (Yang et al., 2013)

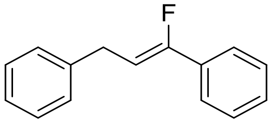

(1,1-Difluoropropane-1,3-diyl)dibenzene **1bb** (23.2 mg, 0.1 mmol) was added to a solution of tris(pentafluorophenyl)borane (10.1 mg, 20 mol%) in dry 1,4-difluorobenzene (1.0 ml). And the resulting mixtuure was refluxed for 24 h under argon atmosphere. The purification by column chromatography on silica gel (*n*-hexane) to give *Z*-**3bb** (8.8 mg, 41%) as a colorless oil. ^{1}H NMR (300 MHz, $CDCl_3$) δ 7.54–7.44 (m, 2H), 7.37–7.19 (m, 8H), 5.60 (dt, *J* = 36.4, 7.7 Hz, 1H), 3.65 (d, *J* = 7.7 Hz, 2H); ^{19}F NMR (282 MHz, $CDCl_3$) δ -121.09 (d, *J* = 36.4 Hz, 1F); MS (EI, *m/z*) 212 $[M]^+$

(*Z*)-(1-Fluoropent-1-en-1-yl)benzene **3cc** (Zhang et al., 2009)

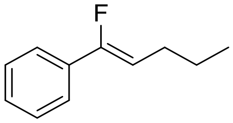

(1,1-Difluoropentyl)benzene **1cc** (18.4 mg, 0.1mmol) was added to a solution of tris(pentafluorophenyl)borane (10.1 mg, 20 mol%) in dry 1,4-difluorobenzene (1.0 ml). And the resulting mixtuure was refluxed for 24 h under argon atmosphere. The purification by column chromatography on silica gel (*n*-hexane) to give *Z*-**3cc** (4.2 mg, 25%) as a colorless oil. ^{1}H NMR (300 MHz, $CDCl_3$) δ 7.50 (dd, *J* = 8.2, 1.4 Hz, 2H), 7.47–7.29 (m, 3H), 5.40 (dt, *J* = 37.6, 7.6 Hz, 1H), 2.27–2.20 (m, 2H), 1.56–1.47 (m, 2H), 0.97 (t, *J* = 7.4 Hz, 3H); ^{19}F NMR (282 MHz, $CDCl_3$) δ -121.38 (d, *J* = 37.6 Hz); MS (EI, *m/z*) 164 $[M]^+$

4-Fluoro-1,2,3,6-tetrahydro-1,1'-biphenyl **3dd** (Vandamme and Paquin, 2017)

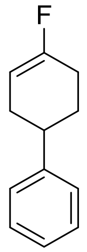

(4,4-Difluorocyclohexyl)benzene **3dd** (19.8 mg, 0.1 mmol) was added to a solution of tris(pentafluorophenyl)borane (10.1 mg, 20 mol%) in dry 1,4-difluorobenzene (1.0 ml). And the resulting mixtuure was refluxed for 24 h under argon atmosphere. The purification by column chromatography on silica gel (*n*-hexane) to give *Z*-**3dd** (15.6 mg, 82%) as a colorless oil. ^{1}H NMR (300 MHz, $CDCl_3$) δ 7.38–7.28 (m, 2H), 7.28–7.16 (m, 3H), 5.30–5.23 (m, 1H), 2.84–2.74 (m, 1H), 2.29–2.21 (m, 4H), 2.02–1.89 (m, 2H); ^{19}F NMR (282 MHz, $CDCl_3$) δ -103.35–-103.72 (m, 1F); MS (EI, *m/z*) 176 $[M]^+$

1-Fluoro-4-pentylcyclohex-1-ene **3ee** (Vandamme et al., 2017)

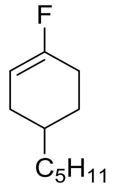

1,1-Difluoro-4-pentylcyclohexane **1ee** (19.0 mg, 0.1 mmol) was added to a solution of tris(pentafluorophenyl)borane (10.1 mg, 20 mol%) in dry 1,4-difluorobenzene (1.0 ml). And the resulting mixtuure was refluxed for 24 h under argon atmosphere. The purification by column chromatography on silica gel (*n*-hexane) to give *Z*-**3ee** (11.1 mg, 64%) as a colorless oil. ^{1}H NMR (300 MHz, $CDCl_3$) δ 5.17–5.10 (m, 1H), 2.23–2.09 (m, 3H), 1.84–1.80 (m, 1H), 1.71–1.62 (m, 1H), 1.52–1.46 (m, 1H), 1.40–1.20 (m, 9H), 0.88 (t, *J* = 6.8 Hz, 3H); ^{19}F NMR (282 MHz, $CDCl_3$)) δ -103.59–-103.77 (m, 1F); MS (EI, *m/z*) 170 $[M]^+$

1-Fluoro-2-phenylcyclohept-1-ene **3ff**

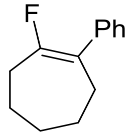

1,1-Difluoro-2-phenylcycloheptane **1ff** (21.0 mg, 0.1 mmol) was added to a solution of tris(pentafluorophenyl)borane (10.1 mg, 20 mol%) in dry 1,4-difluorobenzene (1.0 ml). And the resulting mixtuure was refluxed for 48 h under argon atmosphere. The purification by column chromatography on silica gel (*n*-hexane) to give **3ff** with 1-fluoro-7-phenylcyclohept-1-ene **3ff'** in a 3.3:1 ratio, (9.4 mg, 45%) as a colorless oil. 1-fluoro-2-phenylcyclohept-1-ene **3ff**: ^{1}H NMR (300 MHz, $CDCl_3$) δ 7.42–7.26 (m, 5H), 2.63–2.48 (m, 2H), 2.49–2.36 (m, 2H), 1.86–1.70 (m, 6H). ^{13}C NMR (126 MHz, $CDCl_3$) δ 159.30 (d, *J* = 258.1 Hz), 139.5, 127.97, 127.91, 126.33, 118.73 (d, *J* = 11.5 Hz), 31.79 (d, *J* = 29.6 Hz), 31.54 (d, *J* = 6.3 Hz),

31.2, 26.97 (d, J = 1.6 Hz), 24.67 (d, J = 3.3 Hz). ^{19}F NMR (282 MHz, $CDCl_3$) δ -94.81 (t, J = 17.0 Hz, 1F). 1-fluoro-7-phenylcyclohept-1-ene **3ff'**: ^{1}H NMR (300 MHz, $CDCl_3$) δ 7.25–7.14 (m, 5H), 5.59 (dt, J = 23.9, 6.4 Hz, 1H), 3.86–3.75 (m, 1H), 2.20–2.11 (m, 2H), 2.07–1.92 (m, 2H), 1.63–1.43 (m, 4H). ^{13}C NMR (126 MHz, $CDCl_3$) δ 162.30 (d, J = 246.3 Hz), 141.05 (d, J = 1.4 Hz), 128.4, 127.7, 126.4, 108.24 (d, J = 23.3 Hz), 47.96 (d, J = 28.0 Hz), 32.28 (d, J = 9.3 Hz), 27.1, 24.1, 22.22 (d, J = 11.4 Hz). ^{19}F NMR (282 MHz, $CDCl_3$) δ -94.26 (dd, J = 23.8, 13.0 Hz, 1F). IR (KBr): 3016, 2933, 2861, 1681, 1594, 1490, 1442, 1351, 1176, 1022, 750, 698 cm^{-1}. HRMS (EI) calcd. for $C_{13}H_{15}F^+$ [M^+]: 190.1158, found 190.1168.

1-Fluorocyclododec-1-ene **3gg**

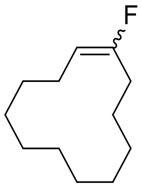

1,1-Difluorocyclododecane **1gg** (20.4 mg, 0.1 mmol) was added to a solution of tris(pentafluorophenyl)borane (10.1 mg, 20 mol%) in dry 1,4-difluorobenzene (1.0 ml). And the resulting mixtuure was refluxed for 48 h under argon atmosphere. The purification by column chromatography on silica gel (*n*-hexane) to give **3gg** (14.9 mg, 71%) as a colorless oil. The ratio for *Z*/*E* isomers (3.3:1) was determined by ^{19}F-NMR. (*Z*)-**3gg**: ^{1}H NMR (300 MHz, $CDCl_3$) δ 4.55 (dt, J = 37.8, 7.8 Hz, 1H), 2.28–2.19 (m, 1H), 2.19–2.11 (m, 3H), 1.39–1.26 (m, J = 12.0 Hz, 16H). ^{13}C NMR (126 MHz, $CDCl_3$) δ 159.1 (d, J = 252.5 Hz), 107.2 (d, J = 15.9 Hz), 31.7 (d, J = 28.4 Hz), 26.2 (d, J = 1.7 Hz), 25.9, 25.7, 25.2, 24.6, 24.65, 24.61, 22.9, 22.87 (d, J = 4.4 Hz). ^{19}F NMR (282 MHz, $CDCl_3$) δ -112.85 (dt, J = 37.8, 21.6 Hz, 1F). (*E*)-**3gg**: ^{1}H NMR (300 MHz, CDC_3) δ 4.96 (dt, J = 23.4, 8.2 Hz, 1H), 2.35–2.30 (m, 2H), 2.02–1.95 (m, 2H), 1.65–1.41 (m, 16H). ^{13}C NMR (126 MHz, $CDCl_3$) δ 160.0 (d, J = 244.9 Hz), 106.7 (d, J = 21.7 Hz), 27.1 (d, J = 2.1 Hz), 26.9 (d, J = 22.7 Hz), 24.6, 24.4, 24.2, 23.9, 23.55, 22.58(d, J = 9.4 Hz), 22.13, 21.93. ^{19}F NMR (282 MHz, $CDCl_3$) δ -106.15–-106.71 (m, 1F). IR (KBr): 2921, 2857, 1695, 1452, 1068 cm^{-1}. HRMS (EI) calcd. for $C_{12}H_{21}F^+$ [M^+]: 184.1627, found 184.1646.

1-Fluorocyclopentadec-1-ene **3hh**

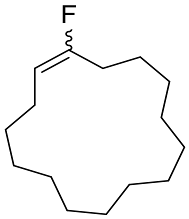

1,1-Difluorocyclopentadecane **1hh** (24.6, 0.1 mmol) was added to a solution of tris(pentafluorophenyl)borane (10.1 mg, 20 mol%) in dry 1,4-difluorobenzene (1.0 ml). And the resulting mixtuure was refluxed for 48 h under argon atmosphere. The ^{19}F-NMR showed that the *Z*/*E* ratio was 3:1. Then the purification by column chromatography on silica gel (*n*-hexane) to give **3hh** (18.8 mg, 80% yield)

as a colorless oil. The ratio for *Z*/*E* isomers (3.0:1) was determined by ^{19}F-NMR. (*Z*)**-3hh**: ^{1}H NMR (300 MHz, $CDCl_3$) δ 4.45 (dt, *J* = 38.6, 7.3 Hz, 1H), 2.26–2.17 (m, 1H), 2.20–1.93 (m, 3H), 1.53–1.45 (m, 3H), 1.44–1.30 (m, 19H); ^{13}C NMR (126 MHz, $CDCl_3$) δ 159.30 (d, *J* = 252.9 Hz), 105.96 (d, *J* = 16.2 Hz), 31.43 (d, *J* = 28.0 Hz), 28.55 (d, *J* = 1.4 Hz), 27.2, 27.1, 27.0, 26.96, 26.90, 26.89, 26.87, 26.8, 25.6, 25.1, 22.7 (d, *J* = 4.7 Hz); ^{19}F NMR (282 MHz, $CDCl_3$) δ -111.35 (dt, *J* = 38.7, 19.4 Hz, 1F). IR (KBr): 2925, 2861, 1706, 1448, 1340 cm^{-1}. MS (EI, *m/z*) 226 $[M]^+$. HRMS (EI) calcd. for $C_{15}H_{27}F^+$ $[M^+]$: 226.2097, found 226.2088.

General procedure for Friedel-Crafts reaction of secondary monofluoroalkanes 4, related to Figure 3.

In a flame-dried test tube, monofluoroalkanes **4a-4s** (0.1 mmol) were added to a solution of $B(C_6F_5)_3$ (2 mol%) in dry HFIP (2.0 mL) at room temperature in a glovebox filled with argon. Subsequently, the tube was sealed with a rubber septum, removed from the glovebox and stirred at 50 ºC for 2-4 h under a positive pressure of argon with a balloon. The resulting mixture was allowed to cool to room temperature and washed with water, extracted with CH_2Cl_2, dried over Na_2SO_4, filtered and then concentrated *in vacuo*. The residue was purified by column chromatography on silica gel using *n*-hexane as the eluent to give the desired substituted indane derivatives **5a-5s.**

1-Phenethyl-2,3-dihydro-1*H*-indene **5a** (Khalaf and Roberts, 1972)

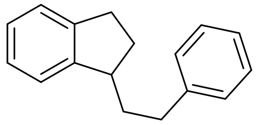

(3-Fluoropentane-1,5-diyl)dibenzene 4**a** (24.2 mg, 0.1 mmol) was added to a solution of tris(pentafluorophenyl)borane (1.0 mg, 2 mol%) in dry $(CF_3)_2$CHOH (2.0 ml). And the resulting mixtuure was stirred at 50 ºC for 2 h under argon atmosphere. The purification by column chromatography on silica gel (*n*-hexane) to give **5a** (20.5 mg, 91%) as a colorless oil. ^{1}H NMR (300 MHz, $CDCl_3$) δ 7.40–7.28 (m, 2H), 7.28–7.18 (m, 4H), 7.18–7.07 (m, 3H), 3.15–3.04 (m, 2H), 2.78–2.67 (m, 2H), 1.91–1.80 (m, 1H), 1.74–1.60 (m, 4H). ^{13}C NMR (126 MHz, $CDCl_3$) δ 140.9, 140.5, 137.0, 129.2, 129.1, 128.8, 128.2, 125.9, 125.6, 125.5, 43.3, 39.5, 29.7, 26.4, 19.1. MS -EI: 222.

4-Methyl-1-(2-methylphenethyl)-2,3-dihydro-1*H*-indene **5b**

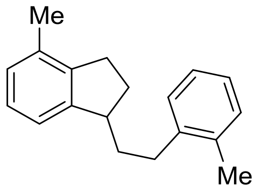

2,2'-(3-Fluoropentane-1,5-diyl)bis(methylbenzene) **4b** (27.0 mg, 0.1 mmol) was added to a solution of tris(pentafluorophenyl)borane (1.0 mg, 2 mol%) in dry $(CF_3)_2$CHOH (2.0 ml). And the resulting mixtuure was stirred at 50 ºC for 5 h under argon atmosphere. The purification by column chromatography on silica gel

(*n*-hexane) to give **5a** (22.5 mg, 85%) as a colorless oil. ^{1}H NMR (300 MHz, $CDCl_3$) δ 7.20–7.09 (m, 4H), 7.11–6.99 (m, 3H), 3.08–3.00 (m, 2H), 2.81–2.69 (m, 2H), 2.60–2.55 (m, 1H), 2.37 (s, 3H), 2.24 (s, 3H), 1.96–1.88 (m, 1H), 1.88–1.74 (m, 1H), 1.72–1.64 (m, 2H). ^{13}C NMR (75 MHz, $CDCl_3$) δ 140.7, 139.2, 136.5, 136.3, 135.4, 130.3, 130.2, 127.2, 126.7, 126.0, 125.6, 125.1, 40.5, 38.3, 26.8, 25.7, 19.7, 19.6, 18.9. IR(KBr): 3016, 2857, 2933, 1587, 1490, 1455, 1371, 1033, 782, 740 cm^{-1}. MS-EI: 250. HRMS (EI) calcd. for $C_{19}H_{22}^{+}$ [M^{+}]: 250.1722, found 250.1720.

6-Methyl-1-(4-methylphenethyl)-2,3-dihydro-1*H*-indene **5c**

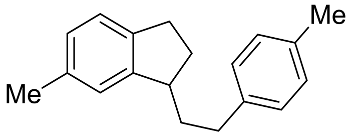

4,4'-(3-Fluoropentane-1,5-diyl)bis(methylbenzene) **4c** (27 mg, 0.1 mmol) was added to a solution of tris(pentafluorophenyl)borane (1.0 mg, 2 mol%) in dry $(CF_3)_2CHOH$ (2.0 ml). And the resulting mixtuure was stirred at 50 °C for 5 h under argon atmosphere. The purification by column chromatography on silica gel (*n*-hexane) to give **5c** (22.9 mg, 90%) as a colorless oil. ^{1}H NMR (300 MHz, $CDCl_3$) δ 7.18–7.03 (m, 5H), 7.03–6.94 (m, 2H), 3.07 (dd, *J* = 13.2, 4.3 Hz, 1H), 2.99 (dt, *J* = 14.8, 4.7 Hz, 1H), 2.75–2.62 (m, 2H), 2.55–2.49 (m, 1H), 2.34 (s, 3H), 2.31 (s, 3H), 1.92–1.77 (m, 1H), 1.73–1.56 (m, 3H). ^{13}C NMR (75 MHz, $CDCl_3$) δ 140.5, 138.0, 135.3, 134.8, 133.9, 129.4, 129.09, 129.04, 128.95, 126.56, 42.96, 39.59, 29.37, 26.33, 21.11, 21.07, 19.28. IR(KBr): 3012, 2857, 2937, 1614, 1498, 1442, 802 cm^{-1}. MS-EI: 250, HRMS (EI) calcd. for $C_{19}H_{22}^{+}$ [M^{+}]: 250.1722, found 250.1715.

6-Ethyl-1-(4-ethylphenethyl)-2,3-dihydro-1*H*-indene **5d**

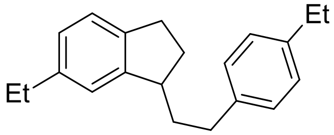

4,4'-(3-Fluoropentane-1,5-diyl)bis(ethylbenzene) **4d** (29.8 mg, 0.1 mmol) was added to a solution of tris(pentafluorophenyl)borane (1.0 mg, 2 mol%) in dry $(CF_3)_2CHOH$ (2.0 ml). And the resulting mixtuure was stirred at 50 °C for 3 h under argon atmosphere. The purification by column chromatography on silica gel (*n*-hexane) to give **5c** (26.6 mg, 93%) as a colorless oil. ^{1}H NMR (300 MHz, $CDCl_3$) δ 7.22–7.06 (m, 4H), 7.08–6.95 (m, 3H), 3.09–3.00 (m, 2H), 2.72–2.55 (m, 7H), 1.92–1.80 (m, 1H), 1.77–1.58 (m, 3H), 1.29–1.17 (m, 6H). ^{13}C NMR (75 MHz, $cdcl_3$) δ 141.7, 141.3, 140.4, 138.2, 134.2, 129.1, 129.0, 128.3, 127.7, 125.3, 43.0, 39.6, 29.4, 28.5, 28.5, 26.5, 19.2, 15.8, 15.7. IR(KBr): 3012, 2933, 2865, 1614, 1508, 1452, 1052, 835, 809 cm^{-1}. EI-MS: 278. HRMS (EI) calcd. for $C_{21}H_{26}^{+}$ [M^{+}]: 278.2035, found 278.2036.

6-Butyl-1-(4-butylphenethyl)-2,3-dihydro-1*H*-indene **5e**

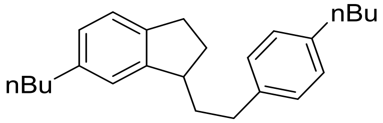

4,4'-(3-Fluoropentane-1,5-diyl)bis(butylbenzene) **4e** (35.4 mg, 0.1 mmol) was added to a solution of tris(pentafluorophenyl)borane (1.0 mg, 2 mol%) in dry $(CF_3)_2CHOH$ (2.0 ml). And the resulting mixtuure was stirred at 50 °C for 5 h under argon atmosphere. The purification by column chromatography on silica gel (*n*-hexane) to give **5e** (28.8 mg, 86%) as a colorless oil. ^{1}H NMR (300 MHz, $CDCl_3$) δ 7.13-7.10 (m, 4H), 7.04-6.98 (m, 3H), 3.13–2.95 (m, 2H), 2.78–2.49 (m, 7H), 1.93–1.81 (m, 1H), 1.74–1.51 (m, 7H), 1.43–1.27 (m, 4H), 0.98-0.85 (m, 6H). ^{13}C NMR (126 MHz, $CDCl_3$) δ 140.4, 140.3, 140.1, 138.2, 134.2, 129.1, 128.9, 128.8, 128.3, 125.9, 43.1, 39.7, 35.5, 35.3, 35.2, 33.9, 33.8, 29.4, 26.6, 22.5, 22.4, 19.2, 14.1; IR (KBr): 3008, 2937, 2857, 1610, 1508, 1455, 1375, 806, 838 cm^{-1}. MS (EI, *m/z*) 334 $[M]^+$. HRMS (EI) calcd. for $C_{25}H_{34}^+$ $[M^+]$: 334.2661, found 334.2663.

6-Methoxy-1-(4-methoxyphenethyl)-2,3-dihydro-1*H*-indene **5f**

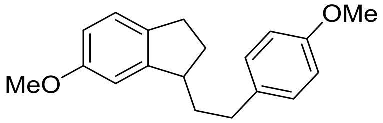

4,4'-(3-Fluoropentane-1,5-diyl)bis(methoxybenzene) **4f** (30.2 mg, 0.1 mmol) was added to a solution of tris(pentafluorophenyl)borane (1.0 mg, 2 mol%) in dry $(CF_3)_2CHOH$ (2.0 ml). And the resulting mixtuure was stirred at 50 °C for 5 h under argon atmosphere. The purification by column chromatography on silica gel (*n*-hexane) to give **5f** (12.4 mg, 44%) as a colorless oil. ^{1}H NMR (300 MHz, $CDCl_3$) δ 7.13 (d, *J* = 8.5 Hz, 2H), 7.04–7.01 (m, 1H), 6.86 (d, *J* = 8.5 Hz, 2H), 6.74–6.69 (m, 2H), 3.80 (s, 3H), 3.76 (s, 3H), 3.09–2.95 (m, 2H), 2.73–2.67 (m, 2H), 1.87–1.82 (m, 1H), 1.68–1.59 (m, 4H); ^{13}C NMR (126 MHz, $CDCl_3$) δ 157.8, 157.2, 141.6, 133.0, 130.1, 129.9, 129.1, 113.7, 113.6, 111.9, 55.3, 55.2, 42.4, 39.9, 28.9, 26.5, 19.4. IR (KBr): 3004, 2915, 2840, 1610, 1579, 1519, 1243, 1033, 846, 794 cm^{-1}. MS-EI: 282. HRMS (EI) calcd. for $C_{19}H_{22}O_2^+$ $[M^+]$: 282.1620, found 282.1622.

4-Bromo-1-(2-bromophenethyl)-2,3-dihydro-1*H*-indene **5g**

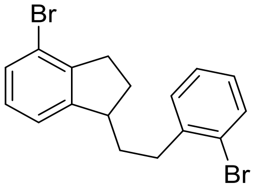

2,2'-(3-Fluoropentane-1,5-diyl)bis(bromobenzene) **4g** (40.0 mg, 0.1 mmol) was added to a solution of tris(pentafluorophenyl)borane (1.0 mg, 2 mol%) in dry $(CF_3)_2CHOH$ (2.0 ml). And the resulting mixtuure was stirred at 50 °C for 5 h under argon atmosphere. The purification by column chromatography on silica gel (*n*-hexane) to give **5g** (32.9 mg, 80%) as a colorless oil. ^{1}H NMR (300 MHz, $CDCl_3$) δ 7.58 (d, *J* = 7.9 Hz, 1H), 7.42 (d, *J* = 7.8 Hz, 1H), 7.29–7.18 (m, 2H), 7.09–7.04 (m, 1H), 7.00 (t, *J* = 7.8 Hz, 1H), 3.32–3.14 (m,

2H), 3.04–2.82 (m, 2H), 2.73–2.57 (m, 1H), 2.06–1.90 (m, 1H), 1.88–1.74 (m, 1H), 1.74–1.55 (m, 2H); ^{13}C NMR (126 MHz, $CDCl_3$) δ 142.9, 139.7 136.3, 133.0, 131.9, 130.1, 128.3, 127.9, 127.2, 126.7, 125.7, 124.9, 43.3, 37.7, 30.4, 25.4, 18.7. IR (KBr): 3056, 2933, 2873, 1554, 1434, 1135, 1037, 808, 777, 719 cm^{-1}. HRMS (EI) calcd. for $C_{17}H_{16}Br_2^+$ [M$^+$]: 377.9619, found 377.9622.

4-Chloro-1-(2-chlorophenethyl)-2,3-dihydro-1*H*-indene **5h**

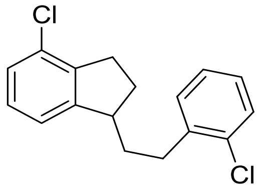

2,2'-(3-Fluoropentane-1,5-diyl)bis(chlorobenzene) **4h** (31.1 mg, 0.1 mmol) was added to a solution of tris(pentafluorophenyl)borane (1.0 mg, 2 mol%) in dry $(CF_3)_2CHOH$ (2.0 ml). And the resulting mixtuure was stirred at 50 °C for 5 h under argon atmosphere. The purification by column chromatography on silica gel (*n*-hexane) to give **5h** (21.8 mg, 75%) as a colorless oil. ^{1}H NMR (300 MHz, $CDCl_3$) δ 7.44–7.35 (m, 1H), 7.28–7.12 (m, 5H), 7.07 (t, *J* = 7.7 Hz, 1H), 3.28–3.14 (m, 2H), 2.98–2.79 (m, 2H), 2.70–2.60(m, 1H), 1.99–1.77 (m, 2H), 1.70–1.58 (m, 2H); ^{13}C NMR (126 MHz, $CDCl_3$) δ 142.7 138.1, 134.8, 134.4, 134.3, 131.8, 129.7, 127.7, 127.6, 126.7, 126.5, 126.2, 40.9, 37.7, 27.4, 25.4, 18.4; IR (KBr): 3056, 2933, 2873, 1594, 1563, 1444, 1143, 1051, 773, 682 cm^{-1}. MS (EI, *m/z*) 290 [M]$^+$. HRMS (EI) calcd. for $C_{17}H_{16}Cl_2^+$ [M$^+$]: 290.0629, found 290.0642.

6-Fluoro-1-(4-fluorophenethyl)-2,3-dihydro-1*H*-indene **5i**

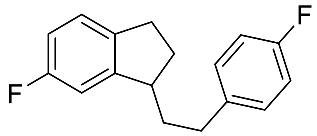

4,4'-(3-Fluoropentane-1,5-diyl)bis(fluorobenzene) **4i** (27.8 mg, 0.1 mmol) was added to a solution of tris(pentafluorophenyl)borane (1.0 mg, 2 mol%) in dry $(CF_3)_2CHOH$ (2.0 ml). And the resulting mixtuure was stirred at 50 °C for 5 h under argon atmosphere. The purification by column chromatography on silica gel (*n*-hexane) to give **5i** (17.6 mg, 68%) as a colorless oil. ^{1}H NMR (300 MHz, $CDCl_3$) δ 7.21–7.09 (m, 2H), 7.08–6.94 (m, 3H), 6.87–6.75 (m, 2H), 3.08–2.95 (m, 2H), 2.76–2.65 (m, 2H), 1.93–1.76 (m, 1H), 1.73–1.56 (m, 4H). ^{13}C NMR (75 MHz, $CDCl_3$) δ 161.4 (d, *J* = 243.8 Hz), 160.8 (d, *J* = 242.8 Hz), 142.1 (d, *J* = 6.5 Hz), 136.1 (d, *J* = 3.3 Hz), 132.5 (d, *J* = 2.9 Hz), 130.49 (d, *J* = 7.7 Hz), 130.3, 115.1 (d, *J* = 21.1 Hz), 114.8 (d, *J* = 21.1 Hz), 112.9 (d, *J* = 21.1 Hz), 42.29, 39.7, 29.0, 26.3, 19.3. IR (KBr): 3041, 2925, 2869, 1602, 1511, 1459, 1153, 1128, 813, 730 cm^{-1}. MS-EI: 258. HRMS (EI) calcd. for $C_{17}H_{16}F_2^+$ [M$^+$]: 258.1220, found 258.1225.

4-Fluoro-1-(2-fluorophenethyl)-2,3-dihydro-1*H*-indene **5j**

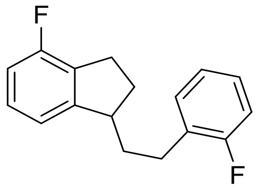

2,2'-(3-Fluoropentane-1,5-diyl)bis(fluorobenzene) **4j** (27.8 mg, 0.1 mmol) was added to a solution of tris(pentafluorophenyl)borane (1.0 mg, 2 mol%) in dry $(CF_3)_2CHOH$ (2.0 ml). And the resulting mixtuure was stirred at 50 °C for 5 h under argon atmosphere. The purification by column chromatography on silica gel (*n*-hexane) to give **5j** (17.6 mg, 68%) as a colorless oil. ^1H NMR (300 MHz, $CDCl_3$) δ 7.21–7.11 (m, 2H), 7.10–6.99 (m, 4H), 6.85 (t, *J* = 8.6 Hz, 1H), 3.17–3.04 (m, 2H), 2.91–2.75 (m, 2H), 2.65–2.56 (m, 1H), 1.95–1.83 (m, 1H), 1.82–1.74 (m, 1H), 1.63–1.54 (m, 2H). ^{13}C NMR (75 MHz, $CDCl_3$) δ 161.4 (d, *J* = 244.8 Hz), 160.7 (d, *J* = 243.3 Hz), 142.8 (d, *J* = 4.7 Hz), 131.5 (d, *J* = 5.1 Hz), 127.8 (d, *J* = 8.1 Hz), 127.5 (d, *J* = 16.0 Hz), 126.1 (d, *J* = 8.9 Hz), 124.6, 124.3 (d, *J* = 3.1 Hz), 123.83 (d, *J* = 3.5 Hz), 115.3 (d, *J* = 22.4 Hz), 111.9 (d, *J* = 22.1 Hz), 38.0, 36.3, 25.8, 22.06 (d, *J* = 4.3 Hz), 17.8. IR (KBr): 3031, 2933, 2857, 1579, 1498, 1457, 1234, 879, 773, 755 cm^{-1}. HRMS (EI) calcd. for $C_{17}H_{16}F_2^+$ [M^+]: 258.1220, found 258.1225.

1-(2,4-Dimethylphenethyl)-4,6-dimethyl-2,3-dihydro-1*H*-indene **5k**

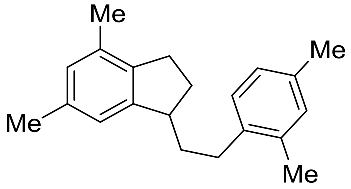

4,4'-(3-Fluoropentane-1,5-diyl)bis(1,3-dimethylbenzene) **4k** (29.8 mg, 0.1 mmol) was added to a solution of tris(pentafluorophenyl)borane (1.0 mg, 2 mol%) in dry $(CF_3)_2CHOH$ (2.0 ml). And the resulting mixtuure was stirred at 50 °C for 5 h under argon atmosphere. The purification by column chromatography on silica gel (*n*-hexane) to give **5j** (14.1 mg, 50%) as a colorless oil. ^1H NMR (300 MHz, $CDCl_3$) δ 7.06 (d, *J* = 7.6 Hz, 1H), 7.01 (s, 1H), 6.98 (d, *J* = 7.6 Hz, 1H), 6.90 (s, 1H), 6.87 (s, 1H), 3.08–2.95 (m, 2H), 2.73–2.65 (m, 2H), 2.57–2.45 (m, 1H), 2.36 (s, 3H), 2.32 (s, 3H), 2.29 (s, 3H), 2.21 (s, 3H), 2.03–1.87 (m, 1H), 1.85–1.71 (m, 1H), 1.70–1.57 (m, 2H). ^{13}C NMR (126 MHz, $CDCl_3$) δ 140.7, 136.3, 136.2, 136.1, 135.5, 134.4, 132.3, 131.1, 130.1, 128.2, 127.1, 126.2, 40.1, 38.3, 26.6, 25.5, 20.96, 20.93, 19.68, 19.63, 18.9. IR (KBr): 3004, 2925, 2861, 1612, 1500, 1452, 1027, 852, 813 cm^{-1}. MS-EI: 278. HRMS (EI) calcd. for $C_{21}H_{26}^+$ [M^+]: 278.2035, found 278.2041.

1-(3,4-Dimethylphenethyl)-4,5-dimethyl-2,3-dihydro-1*H*-indene **5l**

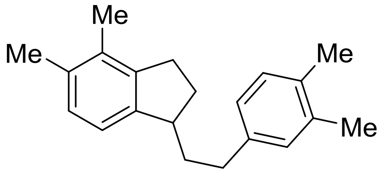

3,3'-(3-Fluoropentane-1,5-diyl)bis(1,2-dimethylbenzene) **4l** (29.8 mg, 0.1 mmol) was added to a solution of tris(pentafluorophenyl)borane (1.0 mg, 2 mol%) in dry $(CF_3)_2CHOH$ (2.0 ml). And the resulting mixture was stirred at 50 °C for 5 h under argon atmosphere. The purification by column chromatography on silica gel (*n*-hexane) to give **5j** (25.2 mg, 67%) as a white solid, mp = 97-98 °C. ^1H NMR (300 MHz, $CDCl_3$) δ 7.08–7.02 (m, 3H), 7.02–6.96 (m, 2H), 3.13 (dd, *J* = 13.5, 4.2 Hz, 1H), 3.02 (dd, *J* = 10.3, 4.8 Hz, 1H), 2.80–2.72 (m, 2H), 2.66–2.50 (m, 1H), 2.31 (s, 6H), 2.28 (s, 3H), 2.15 (s, 3H), 2.01–1.89 (m, 1H), 1.86–1.72 (m, 1H), 1.67–1.55 (m, 2H). ^{13}C NMR (126 MHz, $CDCl_3$) δ 139.1, 138.6, 136.9, 135.2, 134.8, 134.7, 133.7, 128.3, 127.7, 127.1, 126.1, 125.0, 41.2, 38.4, 27.5, 25.4, 20.8, 20.5, 19.1, 15.3, 15.1. IR (KBr): 3016, 2937, 2861, 1590, 1471, 1378, 777, 725 cm^{-1}. HRMS (EI) calcd. for $C_{21}H_{26}^+$ [M^+]: 278.2035, found 278.2059.

1-(2-(Naphthalen-2-yl)ethyl)-2,3-dihydro-1*H*-cyclopenta[b]naphthalene **5m**

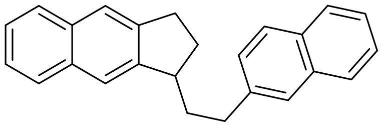

2,2'-(3-Fluoropentane-1,5-diyl)dinaphthalene 4m (34.2 mg, 0.1 mmol) was added to a solution of tris(pentafluorophenyl)borane (1.0 mg, 2 mol%) in dry $(CF_3)_2CHOH$ (2.0 ml). And the resulting mixture was stirred at 50 °C for 5 h under argon atmosphere. The purification by column chromatography on silica gel (*n*-hexane) to give **5m** (25.7 mg, 79%) as a sticky semi-solid. ^1H NMR (300 MHz, $CDCl_3$) δ 8.31 (d, *J* = 8.5 Hz, 1H), 7.94–7.80 (m, 4H), 7.77 (s, 1H), 7.70–7.61 (m, 1H), 7.61–7.53 (m, 2H), 7.52–7.40 (m, 3H), 7.28–7.20 (m, 1H), 3.91 (d, *J* = 11.5 Hz, 1H), 3.41 (d, *J* = 14.2 Hz, 1H), 3.09–2.87 (m, 3H), 2.24–2.06 (m, 1H), 2.02–1.78 (m, 2H), 1.75–1.58 (m, 1H). ^{13}C NMR (126 MHz, $CDCl_3$) δ 138.7, 135.2, 133.9, 133.5, 132.6, 132.0, 131.6, 128.9, 128.3, 128.0, 127.6, 127.5, 127.4, 127.2, 126.3, 126.0, 125.9, 125.2, 124.6, 122.7, 40.5, 35.0, 30.1, 24.4, 17.3. IR (KBr): 3052, 3012, 2925, 2865, 1673, 1600, 1513, 1450, 1373, 1268, 850, 738 cm^{-1}. HRMS (EI) calcd. for $C_{25}H_{22}^+$ [M^+]: 322.1722, found 322.1718.

1-Benzyl-2,3-dihydro-1*H*-indene **5n** (Adamczyk et al., 1984)

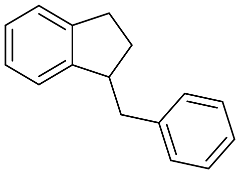

(2-Fluorobutane-1,4-diyl)dibenzene **4n** (22.8 mg, 0.1 mmol) was added to a solution of tris(pentafluorophenyl)borane (1.0 mg, 2 mol%) in dry $(CF_3)_2CHOH$ (2.0 ml). And the resulting mixture was

stirred at 50 °C for 2 h under argon atmosphere. The purification by column chromatography on silica gel (*n*-hexane) to give **5m** (20.0 mg, 91%) as a colorless oil. ^{1}H NMR (300 MHz, $CDCl_3$) δ 7.36–7.24 (m, 5H), 7.22–7.10 (m, 4H), 3.14–2.81 (m, 5H), 2.19–2.08 (m, 1H), 2.03–1.82 (m, 1H); ^{13}C NMR (126 MHz, $CDCl_3$) δ 146.6, 136.6, 136.2, 129.0, 128.9, 128.4, 126.8, 126.1, 125.7, 125.6, 40.7, 37.7, 30.3, 29.7. MS (EI, *m/z*) 208 $[M]^+$

1-Butyl-2,3-dihydro-1*H*-indene **5o** (Adamczyk et al., 1984)

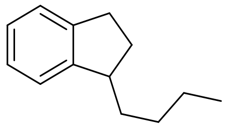

(3-Fluoroheptyl)benzene **4o** (19.4 mg, 0.1 mmol) was added to a solution of tris(pentafluorophenyl)borane (1.0 mg, 2 mol%) in dry $(CF_3)_2CHOH$ (2.0 ml). And the resulting mixture was stirred at 50 °C for 5 h under argon atmosphere. The purification by column chromatography on silica gel (*n*-hexane) to give **5o** (10.9 mg, 62%) as a colorless oil. ^{1}H NMR (300 MHz, $CDCl_3$) δ 7.24–7.05 (m, 4H), 2.87–2.68 (m, 3H), 1.97–1.80 (m, 2H), 1.75–1.61 (m, 3H), 1.58–1.28 (m, 3H), 0.95 (t, *J* = 7.6, 3H). MS (EI, *m/z*) 174 $[M]^+$

1-Isopentyl-2,3-dihydro-1*H*-indene **5p**

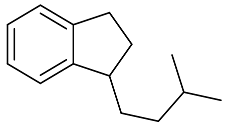

(3-Fluoro-6-methylheptyl)benzene **4p** (20.8 mg, 0.1 mmol) was added to a solution of tris(pentafluorophenyl)borane (1.0 mg, 2 mol%) in dry $(CF_3)_2CHOH$ (2.0 ml). And the resulting mixture was stirred at 50 °C for 2 h under argon atmosphere. The purification by column chromatography on silica gel (*n*-hexane) to give **5p** (7.5 mg, 39%) as a colorless oil. ^{1}H NMR (300 MHz, $CDCl_3$) δ 7.18–7.07 (m, 2H), 7.07–6.95 (m, 2H), 2.91–2.68 (m, 3H), 1.92–1.63(m, 5H), 1.58–1.37 (m, 2H), 0.97 (d, *J* = 6.5 Hz, 3H), 0.94 (d, *J* = 6.6 Hz, 3H); ^{13}C NMR (126 MHz, $CDCl_3$) δ 142.1, 136.9, 129.1, 128.6, 125.4, 125.3, 46.8, 35.1, 29.7, 27.1, 25.4, 23.9, 21.5, 19.3; IR (KBr): 3006, 2937, 2869, 1725, 1573, 1490, 1454, 1365, 748 cm^{-1}. MS (EI, *m/z*) 188 $[M]^+$. HRMS (EI) calcd. for $C_{14}H_{20}^+$ $[M^+]$: 188.1565 found 188.1564.

1-Phenyl-2,3-dihydro-1*H*-indene **5q** (Léonard and Chirik, 2018)

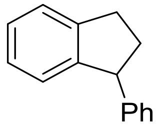

(1-Fluoropropane-1,3-diyl)dibenzene **4q** (21.4 mg, 0.1 mmol) was added to a solution of tris(pentafluorophenyl)borane (1.0 mg, 2 mol%) in dry $(CF_3)_2CHOH$ (2.0 ml). And the resulting mixture was stirred at 50 °C for 2 h under argon atmosphere. The purification by column chromatography on silica gel (*n*-hexane) to give **5q** (5.7 mg, 29%) as a colorless oil. Under the same condition in the absence of tris(pentafluorophenyl)borane, the desired **5q** was isolated in 46% yield (9.1 mg). ^{1}H NMR (300 MHz, $CDCl_3$) δ 7.33–7.16 (m, 8H), 6.96 (d, *J* = 7.3 Hz, 1H), 4.35 (t, *J* = 8.3 Hz, 1H), 3.05–2.97 (m, 2H), 2.66–2.56 (m,

1H), 2.15–1.99 (m, 1H); MS (EI, *m/z*) 194 $[M]^+$

1-Ethyl-1,2,3,4-tetrahydronaphthalene **5r** (Michelet et al., 2014)

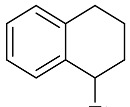

(4-Fluorohexyl)benzene **4r** (18.0 mg, 0.1 mmol) was added to a solution of tris(pentafluorophenyl)borane (1.0 mg, 2 mol%) in dry $(CF_3)_2CHOH$ (2.0 ml). And the resulting mixture was stirred at 50 ^oC for 2 h under argon atmosphere. The purification by column chromatography on silica gel (*n*-hexane) to give **5q** (13.4 mg, 82%) as a colorless oil. ^1H NMR (300 MHz, $CDCl_3$) δ 7.25–7.04 (m, 4H), 2.81–2.62 (m, 3H), 1.95–1.67 (m, 4H), 1.65–1.49 (m, 2H), 0.98 (t, J = 7.4 Hz, 3H); MS (EI, *m/z*) 160 $[M]^+$

1-Butyl-1,2,3,4-tetrahydronaphthalene **5s** (Adamczyk, et al., 1984)

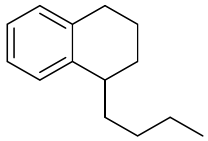

(4-Fluorooctyl)benzene **4s** (20.8 mg, 0.1 mmol) was added to a solution of tris(pentafluorophenyl)borane (1.0 mg, 2 mol%) in dry $(CF_3)_2CHOH$ (2.0 ml). And the resulting mixture was stirred at 50 ^oC for 2 h under argon atmosphere. The purification by column chromatography on silica gel (*n*-hexane) to give **5s** (16.1 mg, 85%) as a colorless oil. ^1H NMR (300 MHz, $CDCl_3$) δ 7.20–7.05 (m, 4H), 2.80–2.66 (m, 3H), 1.90–1.78 (m, 2H), 1.73–1.63 (m, 3H), 1.62–1.49 (m, 1H), 1.44–1.25 (m, 4H), 0.93 (t, J = 6.9 Hz, 3H); MS (EI, *m/z*) 188 $[M]^+$

Synthesis of unkown *gem*-difluorides 1b-1u, 1cc, 1ee and 1hh, related to Figure 2.

For the preparation of substrates **1b-1u**, to a solution of corresponding ketone (1.0 mmol) in dry1,2-dichloroethane at room temperature, was slowly added (diethylamino)sulfur trifluoride (DAST, 2.5 mmol). The resulting mixture was stirred at 60 ^oC, monitored by TLC and upon the completion of the reaction at the same temperature. After cooling to room temperature, the mixture was diluted with CH_2Cl_2, and then washed with water and brine. After drying over Na_2SO_4, the solvent was removed under reduced pressure, and the residue was subject to chromatography on silica gel (hexane/ CH_2Cl_2) to afford desired **1b-1u** in 28% to 57% yields, as shown in the following.

4,4'-(3,3-Difluoropentane-1,5-diyl)bis(methylbenzene) **1b**

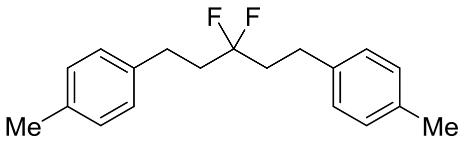

White solid, mp = 64–65 ^oC, 33% yield. ^1H NMR (300 MHz, $CDCl_3$) δ 7.20–6.84 (m, 8H), 2.84–2.68 (m, 4H),

2.32 (s, 6H), 2.23–2.04 (m, 4H). ^{13}C NMR (126 MHz, $CDCl_3$) δ 137.5, 135.7, 129.2, 128.2, 124.2 (t, *J* = 241.3 Hz), 38.6 (t, *J* = 25.2 Hz), 28.1 (t, *J* = 5.0 Hz), 21.1. ^{19}F NMR (282 MHz, $CDCl_3$) δ -99.18 (quintet, *J* = 16.4 Hz, 2F). IR (KBr): 3016, 2937, 2877, 1523, 1434, 1378, 1184, 1052, 908, 815, 742 cm^{-1}. HRMS (EI) calcd. for $C_{19}H_{22}F_2^+$ [M^+] 288.1690, found 288.1688.

4,4'-(3,3-Difluoropentane-1,5-diyl)bis(methoxybenzene) **1c**

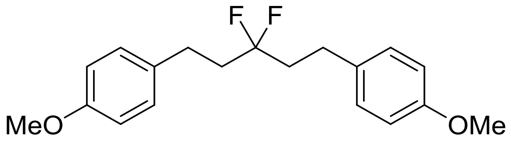

White solid, mp = 54–55 ºC, 28% yield. ^1H NMR (300 MHz, $CDCl_3$) δ 7.10 (d, *J* = 8.5 Hz, 4H), 6.84 (d, *J* = 8.6 Hz, 4H), 3.79 (s, 6H), 2.82–2.72 (m, 4H), 2.17–1.98 (m, 4H). ^{13}C NMR (126 MHz, $CDCl_3$) δ 157.9, 132.6, 129.1, 124.2 (t, *J* = 241.3 Hz), 113.9, 55.2, 38.6 (t, *J* = 25.2 Hz), 27.6 (t, *J* = 5.0 Hz). ^{19}F NMR (282 MHz, $CDCl_3$) δ -99.16 (quintet, *J* = 16.5 Hz, 2F). IR (KBr): 3008, 2937, 2877, 1523, 1434, 1378, 1184, 1052, 908, 815, 742 cm^{-1}. HRMS (EI) calcd. for $C_{19}H_{22}F_2O_2^+$ [M^+] 320.1588, found 320.1587.

4,4'-(3,3-Difluoropentane-1,5-diyl)bis(ethylbenzene) **1d**

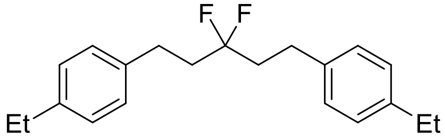

White solid, mp = 35–36 ºC, 33% yield. ^1H NMR (300 MHz, $CDCl_3$) δ 7.18–6.97 (m, 8H), 2.80–2.71 (m, 4H), 2.62 (q, *J* = 7.6 Hz, 4H), 2.29–2.12 (m, 4H), 1.22 (t, *J* = 7.6 Hz, 6H). ^{13}C NMR (126 MHz, $CDCl_3$) δ 142.1, 137.8, 128.2, 128.0, 124.2 (t, *J* = 241.3 Hz), 38.5 (t, *J* = 25.3 Hz), 28.4, 28.11 (t, *J* = 4.9 Hz), 15.6. ^{19}F NMR (282 MHz, $CDCl_3$) δ -99.09 (quintet, *J* = 16.4 Hz, 2F). IR(KBr): 3008, 2960, 2929, 2837, 1515, 1457, 1375, 1299, 1189, 1151, 1172, 813 cm^{-1}. HRMS (EI) calcd. for $C_{21}H_{26}F_2^+$ [M^+] 316.2003, found 316.2000.

4,4'-(3,3-Difluoropentane-1,5-diyl)bis(butylbenzene) **1e**

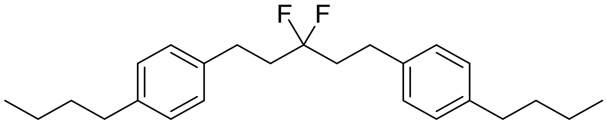

White solid, mp = 30–31 ºC, 36% yield. ^1H NMR (300 MHz, $CDCl_3$) δ 7.25–7.06 (m, 8H), 2.80–2.69 (m, 4H), 2.62–2.46 (m, 4H), 2.26–2.07 (m, 4H), 1.67–1.53 (m, 4H), 1.35 (dq, *J* = 14.5, 7.3 Hz, 4H), 0.92 (t, *J* = 7.3 Hz, 6H). ^{13}C NMR (126 MHz, $CDCl_3$) δ 140.8, 137.7, 128.5, 128.1, 124.27 (t, *J* = 241.3 Hz), 38.49 (t, *J* = 25.2 Hz), 35.2, 33.7, 28.11 (t, *J* = 4.6 Hz), 22.3, 13.9. ^{19}F NMR (282 MHz, $CDCl_3$) δ -99.07 (quintet, *J* = 16.4 Hz, 2F). IR (KBr): 3012, 2956, 2861, 1517, 1455, 1375, 1199, 1153, 1056, 813 cm^{-1}. HRMS (EI) calcd. for $C_{25}H_{34}F_2^+$ [M^+] 372.2629, found 372.2625.

2,2'-(3,3-Difluoropentane-1,5-diyl)bis(methylbenzene) **1f**

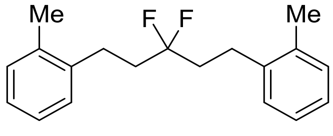

White solid, mp = 52–53 ºC, 32% yield. ^{1}H NMR (300 MHz, $CDCl_3$) δ 7.24–7.09 (m, 8H), 2.87–2.77 (m, 4H), 2.33 (s, 6H), 2.23–2.04 (m, 4H). ^{13}C NMR (126 MHz, $CDCl_3$) δ 138.7, 135.9, 130.3, 128.7, 126.4, 126.2, 124.2 (t, *J* = 241.5 Hz), 37.2 (t, *J* = 25.3 Hz), 25.8 (t, *J* = 5.0 Hz), 19.1. ^{19}F NMR (282 MHz, $CDCl_3$) δ -99.98 (quintet, *J* = 16.4 Hz, 2F). IR(KBr): 3019, 2937, 2869, 1494, 1461, 1380, 1299, 1199, 1157, 1064, 750 cm^{-1}. HRMS (EI) calcd. for $C_{19}H_{22}F_2^+$ [M^+] 288.1690, found 288.1692.

2,2'-(3,3-Difluoropentane-1,5-diyl)bis(methoxybenzene) **1g**

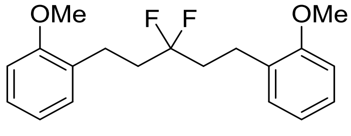

White solid, mp = 89–90 ºC, 45% yield. ^{1}H NMR (300 MHz, $CDCl_3$) δ 7.25–7.13 (m, 4H), 6.88 (dd, *J* = 15.3, 7.8 Hz, 4H), 3.83 (d, *J* = 0.8 Hz, 6H), 2.87–2.75 (m, 4H), 2.22–2.11 (m, 4H). ^{13}C NMR (126 MHz, $CDCl_3$) δ 157.4, 129.7, 129.1, 127.4, 125.1 (t, *J* = 241.0 Hz), 120.4, 110.1, 55.1, 36.2 (t, *J* = 25.2 Hz), 23.7 (t, *J* = 5.4 Hz). ^{19}F NMR (282 MHz, $CDCl_3$) δ -98.23 (quintet, *J* = 16.4 Hz, 2F). IR (KBr): 3019, 2960, 2940, 2844, 1598, 1492, 1457, 1448, 1367, 1243, 1151, 1108, 1052, 1022, 844 cm^{-1}.HRMS (EI) calcd. for $C_{19}H_{22}F_2O_2^+$ [M^+] 320.1588, found 320.1587.

3,3'-(3,3-Difluoropentane-1,5-diyl)bis(methylbenzene) **1h**

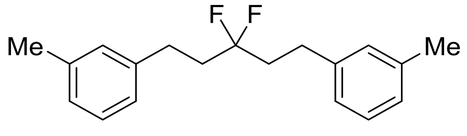

White solid, mp = 44–45ºC, 30% yield. ^{1}H NMR (300 MHz, $CDCl_3$) δ 7.23–7.12 (m, 2H), 7.09–6.94 (m, 6H), 2.85–2.73 (m, 4H), 2.33 (s, 6H), 2.28–2.07 (m, 4H). ^{13}C NMR (126 MHz, $CDCl_3$) δ 140.5, 138.1, 129.1, 128.4, 126.9, 125.2, 124.21 (t, *J* = 241.3 Hz), 38.49 (t, *J* = 25.3 Hz), 28.45 (t, *J* = 5.1 Hz), 21.3. ^{19}F NMR (282 MHz, $CDCl_3$) δ -99.24 (quintet, *J* = 16.3 Hz, 2F). IR(KBr): 3035, 2956, 2929, 1610, 1448, 1378, 1301, 1203, 1151, 1070, 779 cm^{-1}. HRMS (EI) calcd. for $C_{19}H_{22}F_2^+$ [M^+] 288.1690, found 288.1695.

2,2'-(3,3-Difluoropentane-1,5-diyl)bis(fluorobenzene) **1i**

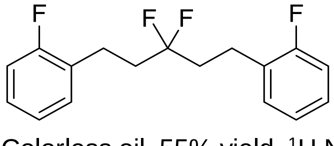

Colorless oil, 55% yield. ^{1}H NMR (300 MHz, $CDCl_3$) δ 7.25–7.14 (m, 4H), 7.12–6.99 (m, 4H), 2.92–2.79 (m, 4H), 2.28–2.12 (m, 4H). ^{13}C NMR (126 MHz, $CDCl_3$) δ 161.1 (d, *J* = 245.1 Hz), 130.5 (d, *J* = 4.9 Hz), 128.1 (d, *J* = 8.1 Hz), 127.4 (d, *J* = 15.6 Hz), 124.1 (d, *J* = 3.6 Hz), 124.0 (t, *J* = 241.6 Hz), 115.3 (d, *J* = 21.9 Hz), 36.8 (t, *J* = 25.2 Hz), 22.2 (td, *J* = 5.4, 2.8 Hz). ^{19}F NMR (282 MHz, $CDCl_3$) δ -99.87 (quintet, *J* = 16.4 Hz,

2F), -117.30–-119.91 (m, 2F). IR (KBr): 3052, 2937, 2869, 1589, 1494, 1454, 1228, 1195, 1060, 752 cm^{-1}. HRMS (EI) calcd. for $C_{17}H_{16}F_4^+$ [M^+] 296.1188, found 296.1194.

2,2'-(3,3-Difluoropentane-1,5-diyl)bis(bromobenzene) **1j**

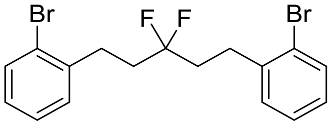

White solid, mp = 58–59 ^oC, 50% yield. ^1H NMR (300 MHz, $CDCl_3$) δ 7.54 (d, *J* = 7.9 Hz, 2H), 7.25 (d, *J* = 4.0 Hz, 4H), 7.09 (dt, *J* = 8.9, 4.4 Hz, 2H), 3.06–2.92 (m, 4H), 2.27–2.13 (m, 4H). ^{13}C NMR (126 MHz, $CDCl_3$) δ 139.8, 132.9, 130.4, 128.0, 127.7, 124.2, 124.0 (t, *J* = 241.9 Hz), 36.5 (t, *J* = 25.3 Hz), 29.2 (t, *J* = 5.3 Hz). ^{19}F NMR (282 MHz, $CDCl_3$) δ -99.18 (quintet, *J* = 16.3 Hz, 2F). IR(KBr): 3060, 2933, 1565, 1475, 1438, 1297, 1211, 1155, 1025, 744 cm^{-1}. HRMS (EI) calcd. for $C_{17}H_{16}Br_2F_2^+$ [M^+] 415.9587, found 415.9598.

2,2'-(3,3-Difluoropentane-1,5-diyl)bis(chlorobenzene) **1k**

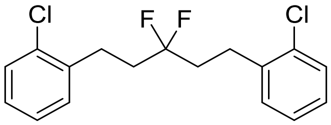

Colorless oil, 56% yield. ^1H NMR (300 MHz, $CDCl_3$) δ 7.36 (dd, *J* = 7.3, 1.6 Hz, 2H), 7.34–7.13 (m, 6H), 3.02–2.93 (m, 4H), 2.28–2.08 (m, 4H). ^{13}C NMR (126 MHz, $CDCl_3$) δ 138.1, 133.8, 130.4, 129.6, 127.8, 127.0, 124.1 (t, *J* = 241.8 Hz), 36.3 (t, *J* = 25.4 Hz), 26.7 (t, *J* = 5.3 Hz). ^{19}F NMR (282 MHz, $CDCl_3$) δ -99.35 (quintet, *J* = 16.3 Hz, 2F). IR(KBr): 3072, 2933, 2869, 1592, 1569, 1477, 1299, 1199, 1126, 1157, 1024, 759 cm^{-1}. HRMS (EI) calcd. for $C_{17}H_{16}Cl_2F_2^+$ [M^+] 328.0597, found 328.0604.

4,4'-(3,3-Difluoropentane-1,5-diyl)bis(bromobenzene) **1l**

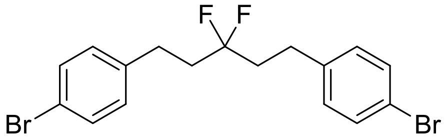

Yellow Solid, mp = 74–76 ^oC, 26% yield. ^1H NMR (300 MHz, $CDCl_3$) δ 7.42 (d, *J* = 8.3 Hz, 4H), 7.06 (d, *J* = 8.3 Hz, 4H), 2.83–2.64 (m, 4H), 2.27–2.03 (m, 4H). ^{13}C NMR (75 MHz, $CDCl_3$) δ 139.4, 131.6, 130.0, 123.7 (t, *J* = 241.7 Hz), 120.0, 38.3 (t, *J* = 25.3 Hz), 27.8 (t, *J* = 5.1 Hz). ^{19}F NMR (282 MHz, $CDCl_3$) δ -99.70 (quintet, *J* = 16.1 Hz, 2F). IR(KBr): 3025, 2971, 2925, 2867, 1492, 1455, 1402, 1267, 1193, 1068, 1010, 844, 736 cm^{-1}. HRMS (EI) calcd. for $C_{17}H_{16}Br_2F_2^+$ [M^+] 415.9587, found 415.9583.

4,4'-(3,3-Difluoropentane-1,5-diyl)bis(chlorobenzene) **1m**

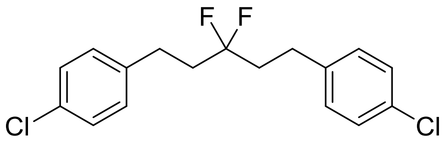

Yellow Solid, mp = 54–55 °C, 41% yield. ^{1}H NMR (300 MHz, $CDCl_3$) δ 7.26 (d, *J* = 8.4 Hz, 4H), 7.10 (d, *J* = 8.3 Hz, 4H), 2.86–2.72 (m, 4H), 2.24–1.99 (m, 4H). ^{13}C NMR (126 MHz, $CDCl_3$) δ 138.9, 132.0, 129.6, 128.6, 123.7 (t, *J* = 241.7 Hz), 38.38 (t, *J* = 25.3 Hz), 27.81 (t, *J* = 5.0 Hz). ^{19}F NMR (282 MHz, $CDCl_3$) δ -99.66 (quintet, *J* = 16.4 Hz, 2F). IR (KBr): 3027, 2937, 2889, 1490, 1455, 1407, 1384, 1159, 1095, 1014, 815, 757 cm^{-1}. HRMS (EI) calcd. for $C_{17}H_{16}Cl_2F_2^+$ [M$^+$] 328.0597, found 328.0606.

4,4'-(3,3-Difluoropentane-1,5-diyl)bis(1,3-dimethylbenzene) **1n**

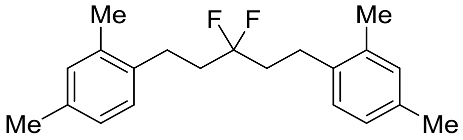

White Solid, mp = 66–68 °C, 29% yield. ^{1}H NMR (300 MHz, $CDCl_3$) δ 7.08–6.92 (m, 6H), 2.87–2.72 (m, 4H), 2.29 (s, 12H), 2.19–1.98 (m, 4H). ^{13}C NMR (126 MHz, $CDCl_3$) δ 135.9, 135.7, 135.6, 131.1, 128.6, 126.8, 124.3 (t, *J* = 241.4 Hz), 37.3 (t, *J* = 25.3 Hz), 25.4 (t, *J* = 5.0 Hz), 20.8, 19.1. ^{19}F NMR (282 MHz, $CDCl_3$) δ -99.82 (quintet, *J* = 16.5 Hz, 2F). IR(KBr): 3002, 2948, 2879, 1614, 1502, 1461, 1376, 1270, 1189, 1047, 840, 761 cm^{-1}. HRMS (EI) calcd. for $C_{21}H_{26}F_2^+$ [M$^+$] 316.2003, found 316.2013.

3,3'-(3,3-Difluoropentane-1,5-diyl)bis(1,2-dimethylbenzene) **1o**

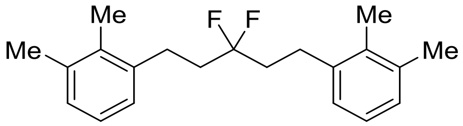

White Solid, mp = 76–77 °C, 41% yield. ^{1}H NMR (300 MHz, $CDCl_3$) δ 7.12–6.98 (m, 6H), 2.87–2.75 (m, 4H), 2.28 (s, 6H), 2.22 (s, 6H), 2.24–2.04 (m, 4H). ^{13}C NMR (126 MHz, $CDCl_3$) δ 138.6, 137.1, 134.4, 128.1, 126.8, 125.6, 124.29 (t, *J* = 241.4 Hz), 37.56 (t, *J* = 25.3 Hz), 26.65 (t, *J* = 4.9 Hz), 20.7, 14.9. ^{19}F NMR (282 MHz, $CDCl_3$) δ -99.97 (quintet, *J* = 16.5 Hz, 2F).
IR(KBr): 3001, 2948, 2892, 1585, 1467, 1440, 1386, 1186, 1031, 823, 773 cm^{-1}. HRMS (EI) calcd. for $C_{21}H_{26}F_2^+$ [M$^+$] 316.2003, found 316.1998.

2,2'-(3,3-Difluoropentane-1,5-diyl)dinaphthalene

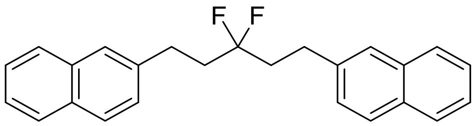

White Solid, mp = 110–112 °C, 48% yield. ^{1}H NMR (300 MHz, $CDCl_3$) δ 7.82–7.70 (m, 6H), 7.60 (s, 2H), 7.52–7.39 (m, 4H), 7.32 (dd, *J* = 8.4, 1.5 Hz, 2H), 3.09–2.97 (m, 4H), 2.50–2.21 (m, 4H). ^{13}C NMR (126

MHz, $CDCl_3$) δ 138.0, 133.5, 132.0, 128.1, 127.6, 127.4, 126.9, 126.4, 126.0, 125.4, 124.23 (t, *J* = 241.5 Hz), 38.37 (t, *J* = 25.3 Hz), 28.71 (t, *J* = 5.0 Hz). ^{19}F NMR (282 MHz, $cdcl_3$) δ -98.91 (quintet, *J* = 16.2 Hz, 2F). IR (KBr): 2937, 1598, 1508, 1458, 1365, 1295, 1155, 1102, 1066, 817 cm^{-1}. HRMS (EI) calcd. for $C_{25}H_{22}F_2^+$ [M^+] 360.1690, found 360.1696.

1-(3,3-Difluoro-5-phenylpentyl)-2,4-dimethylbenzene **1p**

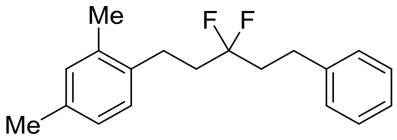

Colorless oil, 48% yield. ^{1}H NMR (300 MHz, $CDCl_3$) δ 7.34–7.26 (m, 2H), 7.25–7.12 (m, 3H), 7.07–6.94 (m, 3H), 2.85–2.64 (m, 4H), 2.29 (s, 6H), 2.22–1.97 (m, 4H). ^{13}C NMR (126 MHz, $CDCl_3$) δ 140.6, 135.9, 135.7, 135.6, 131.1, 128.6, 128.5, 128.2, 126.8, 126.2, 124.2 (t, *J* = 241.4 Hz), 38.4 (t, *J* = 25.3 Hz), 37.3 (t, *J* = 25.2 Hz), 28.5 (t, *J* = 5.0 Hz), 25.4 (t, *J* = 5.0 Hz), 20.8, 19.1. ^{19}F NMR (282 MHz, $CDCl_3$) δ -99.53 (m, 2F). IR(KBr): 3027, 2944, 2869, 1504, 1450, 1382, 1305, 1199, 1159, 811 cm^{-1}. HRMS (EI) calcd. for $C_{19}H_{22}F_2^+$ [M^+] 288.1690, found 288.1697.

1-Bromo-2-(3,3-difluoro-5-(o-tolyl)pentyl)benzene **1r**

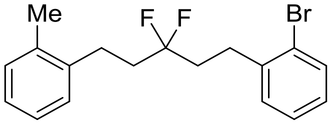

Yellow oil, 44% yield. ^{1}H NMR (300 MHz, $CDCl_3$) δ 7.54 (d, *J* = 7.8 Hz, 1H), 7.29–7.22 (m, 2H), 7.21–7.07 (m, 5H), 3.04–2.90 (m, 2H), 2.90–2.76 (m, 2H), 2.33 (s, 3H), 2.28–2.00 (m, 4H). ^{13}C NMR (126 MHz, $CDCl_3$) δ 139.8, 138.7, 135.9, 132.9, 130.4, 130.3, 128.6, 128.1, 127.7, 126.4, 126.2, 124.2, 124.1 (t, *J* = 241.6 Hz), 37.0 (t, *J* = 25.2 Hz), 36.6 (t, *J* = 25.4 Hz), 29.3 (t, *J* = 5.3 Hz), 25.8 (t, *J* = 5.1 Hz), 19.1. ^{19}F NMR (282 MHz, $cdcl_3$) δ -99.51 (quintet, *J* = 16.3 Hz, 2F). HRMS (EI) calcd. for $C_{18}H_{19}BrF_2^+$ [M^+] 352.0638, found 352.0639.

(4,4-Difluoroheptane-1,7-diyl)dibenzene **1s**

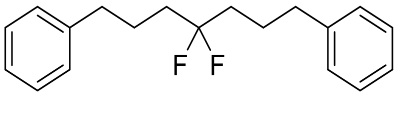

Yellow oil, 35% yield. ^{1}H NMR (300 MHz, $CDCl_3$) δ 7.34–7.23 (m, 4H), 7.23–7.10 (m, 6H), 2.65–2.50 (m, 4H), 1.95–1.63 (m, 8H). ^{13}C NMR (126 MHz, $CDCl_3$) δ 141.4, 128.3, 128.3, 125.9, 125.1 (t, *J* = 240.4 Hz), 35.7 (t, *J* = 25.5 Hz), 35.3, 23.9 (t, *J* = 4.5 Hz). ^{19}F NMR (282 MHz, $CDCl_3$) δ -97.85 (m, 2F).IR(KBr): 3023, 2952, 2857, 1604, 1492, 1454, 1322, 1091, 752 cm^{-1}. HRMS (EI) calcd. for $C_{19}H_{22}F_2^+$ [M^+] 288.1690, found 288.1692.

(3,3-Difluorohexane-1,6-diyl)dibenzene **1t**

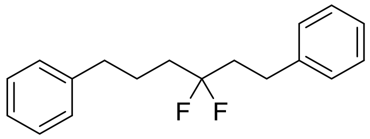

Colorless oil, 29% yield. ^{1}H NMR (300 MHz, $CDCl_3$) δ 7.37–7.24 (m, 4H), 7.24–7.08 (m, 6H), 2.85–2.72 (m, 2H), 2.71–2.58 (m, 2H), 2.25–2.00 (m, 2H), 1.98–1.70 (m, 4H). ^{13}C NMR (126 MHz, $CDCl_3$) δ 141.4, 140.6, 128.5, 128.4, 128.3, 128.2, 126.1, 125.9, 124.6 (t, *J* = 240.9 Hz), 38.2 (t, *J* = 25.5 Hz), 35.9 (t, *J* = 25.3 Hz), 35.3, 28.41 (t, *J* = 5.0 Hz), 24.05 (t, *J* = 4.5 Hz). ^{19}F NMR (282 MHz, $CDCl_3$) δ -98.67 (quintet, *J* = 16.1 Hz, 2F). IR(KBr): 3027, 2952, 2857, 1606, 1496, 1454, 1321, 1205, 1149, 746 cm^{-1}. HRMS (EI) calcd. for $C_{18}H_{20}F_2^+$ [M$^+$] 274.1533, found 274.1539.

4,4'-(3,3-Difluoropentane-1,5-diyl)bis(fluorobenzene) **1u**

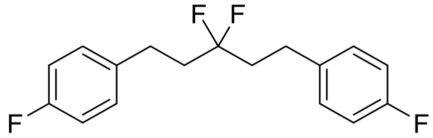

Colorless oil, 57% yield. ^{1}H NMR (300 MHz, $CDCl_3$) δ 7.24–7.07 (m, 4H), 7.08–6.95 (m, 4H), 2.87–2.71 (m, 4H), 2.28–2.05 (m, 4H). ^{13}C NMR (126 MHz, $CDCl_3$) δ 161.4 (d, *J* = 244.0 Hz), 136.1 (d, *J* = 3.2 Hz), 129.6 (d, *J* = 7.9 Hz), 123.8 (t, *J* = 241.5 Hz), 115.3 (d, *J* = 21.2 Hz), 38.6 (t, *J* = 25.3 Hz), 27.6 (t, *J* = 5.0 Hz). ^{19}F NMR (282 MHz, $CDCl_3$) δ -99.66 (qunitet, *J* = 16.4 Hz, 2F), -116.94–-117.21 (m, 1F). IR(KBr): 2877, 2929, 1608, 1517, 1454, 1311, 1228, 1157, 1054, 829 cm^{-1}. HRMS (EI) calcd. for $C_{17}H_{16}F_4^+$ [M$^+$] 296.1188, found 296.1191.

(1,1-Difluoropentyl)benzene **1cc**

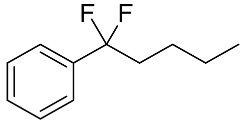

To a solution of 1-phenylpentan-1-one (1.0 mmol) in CH_2Cl_2 (1.0 mL) at room temperature, was slowly added 4-*tert*-butyl-2,6-dimethylphenylsulfur trifluoride (Fluolead, 2.0 mmol) and hydrogen fluoride pyridine (around 70% HF, 0.4 equiv). (Umemoto et al., 2010) The resulting mixture was stirred for 36 hours and was diluted with CH_2Cl_2, and then washed with saturated Na_2CO_3 aqueous solution and brine. After drying over Na_2SO_4, the solvent was removed under reduced pressure, and the residue was subject to chromatography on silica gel (hexane) to afford desired **3cc** in 80% yields, as a colorless liquid. ^{1}H NMR (300 MHz, $CDCl_3$) δ 7.45–7.30 (m, 5H), 2.25–2.03 (m, 2H), 1.48–1.31 (m, 4H), 0.88 (t, *J* = 7.0 Hz, 3H). ^{13}C NMR (126 MHz, $CDCl_3$) δ 137.5 (t, *J* = 26.7 Hz), 129.4 (d, *J* = 1.6 Hz), 128.3, 124.9 (t, *J* = 6.3 Hz), 123.1 (t, *J* = 241.9 Hz), 38.8 (t, *J* = 27.4 Hz), 24.5 (t, *J* = 4.0 Hz), 22.3, 13.8. ^{19}F NMR (282 MHz, $CDCl_3$) δ -95.44 (t, *J* = 16.2 Hz). HRMS (EI) calcd. for $C_{11}H_{14}F_2^+$ [M$^+$] 184.1064, found 184.1068.

1,1-Difluoro-4-pentylcyclohexane **1ee**

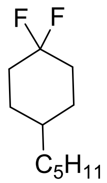

To a solution of 4-pentylcyclohexan-1-one (1.0 mmol) in dry CH_2Cl_2 at -40 ^oC, was slowly added DAST ((diethylamino)sulfur trifluoride, 2.0 mmol). The resulting mixture was slowly warmed to room temperature with 2-3 hours. And the reaction mixture was monitored by TLC and upon the completion of the reaction at the same temperature and was diluted with CH_2Cl_2, and then washed with water and brine. After drying over Na_2SO_4, the solvent was removed under reduced pressure, and the residue was subject to chromatography on silica gel (hexane/ CH_2Cl_2) to afford desired **3ee** in 72% yield, as a colorless liquid. ^1H NMR (300 MHz, $CDCl_3$) δ 2.19–1.95 (m, 2H), 1.77–1.54 (m, 4H), 1.35–1.10 (m, 11H), 0.88 (t, *J* = 6.6 Hz, 3H). ^{13}C NMR (126 MHz, $CDCl_3$) δ 123.9 (dd, *J* = 241.5, 239.6 Hz), 35.6 (d, *J* = 3.2 Hz), 33.6 (d, *J* = 22.2 Hz), 33.4 (d, *J* = 22.2 Hz), 32.0, 28.9 (d, *J* = 9.5 Hz), 26.8, 22.6, 14.1. ^{19}F NMR (282 MHz, $CDCl_3$) δ -91.32 (d, *J* = 233.2 Hz, 1F), -101.18–-102.71 (m, 1F). HRMS (EI) calcd. for $C_{11}H_{20}F^+$ $[M-F]^+$ 171.1544, found 171.1548.

1,1-Difluorocyclopentadecane **1hh**

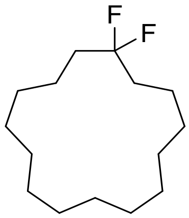

To a solution of cyclopentadecanone (1.0 mmol) in CH_2Cl_2 (1.0 mL) at room temperature, was slowly added 4-*tert*-butyl-2,6-dimethylphenylsulfur trifluoride (Fluolead, 2.0 mmol) and hydrogen fluoride pyridine (around 70% HF, 0.4 equiv). (Umemoto et al., 2010) The resulting mixture was stirred for 48 hours and was diluted with CH_2Cl_2, and then washed with saturated Na_2CO_3 aqueous solution and brine. After drying over Na_2SO_4, the solvent was removed under reduced pressure, and the residue was subject to chromatography on silica gel (hexane) to afford desired **3hh** in 66% yields, as a colorless semi-solid. ^1H NMR (300 MHz, $CDCl_3$) δ 1.93–1.79 (m, 4H), 1.53–1.37 (m, 12H), 1.37–1.24 (m, 12H). ^{13}C NMR (126 MHz, $CDCl_3$) δ 126.5 (t, *J* = 239.8 Hz), 34.5 (t, *J* = 25.5 Hz), 26.9, 26.7, 26.4, 26.3, 26.3, 21.3 (t, *J* = 5.5 Hz). ^{19}F NMR (282 MHz, $CDCl_3$) δ -90.88 (quintet, *J* = 15.5 Hz, 2F). IR(KBr): 2929, 2862, 1448, 1085, 1037 cm^{-1}. HRMS (EI) calcd. for $C_{15}H_{28}F^+$ $[M-F]^+$, 227.2170 found 227.2179

General procedure for preparation of aliphatic fluoride 4a-4s, related to Figure 3.

To a solution of aliphatic secondary alcohol (1.0 mmol) in dry CH_2Cl_2 at -78 °C, was slowly added (diethylamino)sulfur trifluoride (DAST, 1.3 mmol). The resulting mixture was slowly warmed to room temperature with 2-3 hours. And the reaction mixture was monitored by TLC and upon the completion of the reaction at the same temperature (around 2-3 hours) and was diluted with CH_2Cl_2, and then washed with water and brine. After drying over Na_2SO_4, the solvent was removed under reduced pressure, and the residue was subject to chromatography on silica gel (hexane/ CH_2Cl_2) to afford desired **4a-4s** in 41% to 90% yields as shown in the following.

(3-Fluoropentane-1,5-diyl)dibenzene **4a**

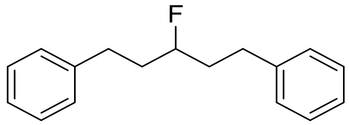

Colorless oil, 82% yield. ^{1}H NMR (300 MHz, $CDCl_3$) δ 7.40–7.24 (m, 4H), 7.23–7.02 (m, 6H), 4.61–4.39 (m, 1H, $^2J_{H\text{-}F}$ = 49.4 Hz), 2.87–2.62 (m, 4H), 2.02–1.76 (m, 4H). ^{13}C NMR (75 MHz, $CDCl_3$) δ 141.4, 128.4, 125.9, 92.7 (d, J = 168.2 Hz), 37.0 (d, J = 20.9 Hz), 31.4 (d, J = 4.3 Hz). ^{19}F NMR (282 MHz, $cdcl_3$) δ -183.61–-184.34 (m, 1F). IR (KBr): 3031, 2940, 2865, 1606, 1490, 1442, 1164, 1037, 754, 698 cm^{-1}. HRMS (EI) calcd. for $C_{17}H_{19}F^+$ [M$^+$]: 242.1471, found 242.1469.

2,2'-(3-Fluoropentane-1,5-diyl)bis(methylbenzene) **4b**

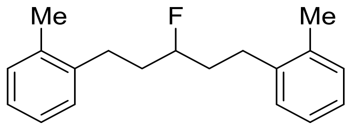

Colorless oil, 87% yield. ^{1}H NMR (300 MHz, $CDCl_3$) δ 7.22–7.01 (m, 8H), 4.62–4.37 (m, 1H, $^2J_{H\text{-}F}$ = 49.3 Hz), 2.99–2.75 (m, 2H), 2.75–2.55 (m, 2H), 2.31 (s, 6H), 2.06–1.74 (m, 4H). ^{13}C NMR (75 MHz, $CDCl_3$) δ 139.6, 135.8, 130.2, 128.8, 126.1, 126.0, 93.1 (d, J = 168.5 Hz), 35.7 (d, J = 21.0 Hz), 28.7 (d, J = 4.3 Hz), 19.2. ^{19}F NMR (282 MHz, $CDCl_3$) δ -181.50–-185.19 (m, 1F). IR (KBr): 3019, 2937, 2877, 1598, 1486, 1455, 1378, 1168, 1025, 738 cm^{-1}. HRMS (EI) calcd. for $C_{19}H_{23}F^+$ [M$^+$]: 270.1784 found 270.1783.

4,4'-(3-Fluoropentane-1,5-diyl)bis(methylbenzene) **4c**

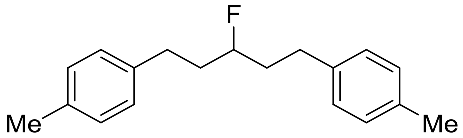

Colorless oil, 80% yield. ^{1}H NMR (300 MHz, $CDCl_3$) δ 7.18–6.96 (m, 8H), 4.62–4.37 (m, 1H, $^2J_{H\text{-}F}$ = 49.3 Hz), 2.87–2.70 (m, 2H), 2.69–2.56 (m, 2H), 2.32 (s, 6H), 2.12–1.77 (m, 4H). ^{13}C NMR (75 MHz, $CDCl_3$) δ 138.3, 135.3, 129.1, 128.3, 92.8 (d, J = 168.0 Hz), 37.1 (d, J = 20.9 Hz), 30.9 (d, J = 4.4 Hz), 20.9. ^{19}F NMR (282 MHz, $CDCl_3$) δ -177.86–-191.48 (m, 1F). IR (KBr): 3008, 2940, 2861, 1515, 1442, 1375, 1041, 892, 806 cm^{-1}. HRMS (EI) calcd. for $C_{19}H_{23}F^+$ [M$^+$]: 270.1784 found 270.1789.

4,4'-(3-Fluoropentane-1,5-diyl)bis(ethylbenzene) **4d**

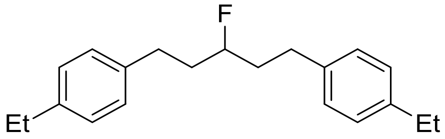

Colorless oil, 87% yield. ^{1}H NMR (300 MHz, $CDCl_3$) δ 7.20–6.98 (m, 8H), 4.68–4.39 (m, 1H, $^{2}J_{H\text{-}F}$ = 49.3 Hz), 2.92–2.58 (m, 8H), 2.16–1.79 (m, 4H), 1.23 (t, *J* = 7.6 Hz, 6H). ^{13}C NMR (75 MHz, $CDCl_3$) δ 141.8, 138.6, 128.3, 127.9, 92.8 (d, *J* = 168.0 Hz), 37.1 (d, *J* = 20.9 Hz), 30.9 (d, *J* = 4.4 Hz), 28.4, 15.7. ^{19}F NMR (282 MHz, $CDCl_3$) δ -178.72–-187.28 (m, 1F). IR (KBr): 3016, 2498, 2873, 1519, 1438, 1378, 1037, 898, 838 cm^{-1}. HRMS (EI) calcd. for $C_{21}H_{27}F^{+}$ [M^{+}]: 298.2097 found 298.2098.

4,4'-(3-Fluoropentane-1,5-diyl)bis(butylbenzene) **4e**

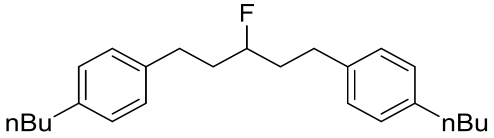

Colorless oil, 78% yield. ^{1}H NMR (300 MHz, $CDCl_3$) δ 7.16–6.97 (m, 8H), 4.62–4.39 (m, 1H, $^{2}J_{H\text{-}F}$ = 49.3 Hz), 2.86–2.71 (m, 2H), 2.69–2.49 (m, 6H), 2.01–1.86 (m, 4H), 1.66–1.53 (m, 4H), 1.43–1.21 (m, 4H), 0.92 (t, *J* = 7.3 Hz, 6H). ^{13}C NMR (75 MHz, $CDCl_3$) δ 140.5, 138.5, 128.4, 128.2, 92.8 (d, *J* = 168.0 Hz), 37.0 (d, *J* = 20.9 Hz), 35.2, 33.7, 30.9 (d, *J* = 4.5 Hz), 22.3, 13.9. ^{19}F NMR (282 MHz, $CDCl_3$) δ -172.31–-193.10 (m, 1F). IR (KBr): 3019, 2940, 2857, 1511, 1455, 1375, 1045, 902, 825 cm^{-1}. HRMS (EI) calcd. for $C_{25}H_{35}F^{+}$ [M^{+}]: 354.2723 found 354.2726.

4,4'-(3-Fluoropentane-1,5-diyl)bis(methoxybenzene) **4f**

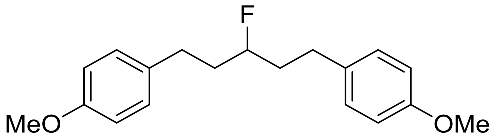

Colorless oi, 70% yield. ^{1}H NMR (300 MHz, $CDCl_3$) δ 7.09 (d, *J* = 8.5 Hz, 2H), 6.83 (d, *J* = 8.5 Hz, 2H), 4.60–4.35 (m, 1H, $^{2}J_{H\text{-}F}$ = 49.3 Hz), 3.79 (s, 6H), 2.75–2.61 (m, 4H), 1.99–1.67 (m, 4H). ^{13}C NMR (75 MHz, $CDCl_3$) δ 157.7, 133.4, 129.3, 113.7, 92.6 (d, *J* = 167.8 Hz), 55.2 (s), 37.2 (d, *J* = 20.8 Hz), 30.4 (d, *J* = 4.4 Hz). ^{19}F NMR (282 MHz, $CDCl_3$) δ -172.79–-190.80 (m, 1F). IR (KBr): 2940, 2836, 1610, 1587, 1523, 1459, 1303, 1240, 1172, 1033, 829 cm^{-1}. HRMS (EI) calcd. for $C_{19}H_{23}FO_2^{+}$ [M^{+}]: 302.1682 found 302.1687.

2,2'-(3-Fluoropentane-1,5-diyl)bis(bromobenzene) **4g**

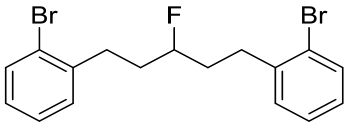

White solid, mp = 36–37 ^{o}C, 52% yield. ^{1}H NMR (300 MHz, $CDCl_3$) δ 7.53 (d, *J* = 7.9 Hz, 2H), 7.32–7.13

(m, 4H), 7.13–6.97 (m, 2H), 4.68–4.43 (m, 1H, $^2J_{H\text{-}F}$ = 49.3 Hz), 3.09–2.81 (m, 4H), 2.03–1.75 (m, 4H). ^{13}C NMR (75 MHz, $CDCl_3$) δ 140.6, 132.8, 131.8, 127.7, 127.5, 124.3, 92.6 (d, J = 169.1 Hz), 35.0 (d, J = 20.9 Hz), 31.8 (d, J = 4.5 Hz). ^{19}F NMR (282 MHz, $CDCl_3$) δ -174.86–-190.11 (m, 1F). IR (KBr): 3060, 2944, 2833, 1698, 1562, 1455, 1022, 881, 655 cm^{-1}. HRMS (EI) calcd. for $C_{17}H_{17}Br_2F^+$ [M$^+$]: 397.9681 found 397.9685.

2,2'-(3-Fluoropentane-1,5-diyl)bis(chlorobenzene) **4h**

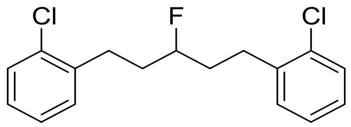

Colorless oil, 86% yield. ^{1}H NMR (300 MHz, $CDCl_3$) δ 7.48–7.29 (m, 2H), 7.28–7.02 (m, 6H), 4.63–4.41 (m, 1H, $^2J_{H\text{-}F}$ = 49.3 Hz), 3.03–2.95 (m, 4H), 2.02–1.74 (m, 4H). ^{13}C NMR (75 MHz, $CDCl_3$) δ 138.9, 133.8, 130.5, 129.5, 127.5, 126.8, 92.7 (d, J = 169.0 Hz), 34.9 (d, J = 20.9 Hz), 29.3 (d, J = 4.6 Hz). ^{19}F NMR (282 MHz, $CDCl_3$) δ -179.97–-189.12 (m, 1F). IR (KBr): 3068, 2933, 2869, 1575, 1448, 1475, 1378, 1134, 1045, 892, 750, 678 cm^{-1}. HRMS (EI) calcd. for $C_{17}H_{17}Cl_2F^+$ [M$^+$]: 310.0691 found 310.0696.

4,4'-(3-Fluoropentane-1,5-diyl)bis(fluorobenzene) **4i**

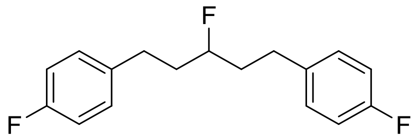

Colorless oil, 75% yield.^{1}H NMR (300 MHz, $CDCl_3$) δ 7.21–7.06 (m, 4H), 7.03–6.87 (m, 4H), 4.58–4.33 (m, 1H, $^2J_{H\text{-}F}$ = 49.1 Hz), 2.78–2.60 (m, 4H), 2.06–1.75 (m, 4H). ^{13}C NMR (75 MHz, $CDCl_3$) δ 161.31 (d, J = 243.6 Hz), 136.95 (d, J = 3.2 Hz), 129.75 (d, J = 7.8 Hz), 115.19 (d, J = 21.1 Hz), 92.30 (d, J = 168.5 Hz), 37.10 (d, J = 21.0 Hz), 30.57 (d, J = 4.4 Hz) ^{19}F NMR (282 MHz, $CDCl_3$) δ -117.85 (s, 2F), -180.69–-190.67 (m, 1F). IR (KBr): 3031, 2940, 2861, 1610, 1502, 1438, 1232, 1037, 829 cm^{-1}. HRMS (EI) calcd. for $C_{17}H_{17}F_3^+$ [M$^+$]: 278.1282 found 278.1282.

2,2'-(3-Fluoropentane-1,5-diyl)bis(fluorobenzene) 4**j**

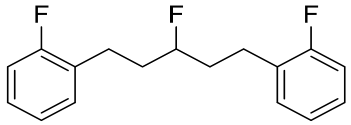

Colorless oil, 90% yield. ^{1}H NMR (300 MHz, $CDCl_3$) δ 7.24–7.12 (m, 4H), 7.11–6.97 (m, 4H), 4.61–4.40 (m, 1H, $^2J_{H\text{-}F}$ = 49.3 Hz), 2.90–2.67(m, 4H), 2.02–1.74 (m, 4H). ^{13}C NMR (75 MHz, $CDCl_3$) δ 161.1 (d, J = 244.8 Hz), 130.7 (d, J = 5.0 Hz), 128.1 (d, J = 15.8 Hz), 127.7 (d, J = 8.1 Hz), 123.9 (d, J = 3.5 Hz), 115.6 (d, J = 22.0 Hz), 92.7 (d, J = 168.7 Hz), 35.4 (d, J = 20.9 Hz), 24.9 (d, J = 2.5 Hz). ^{19}F NMR (282 MHz, $CDCl_3$) δ -119.37 (s, 2F), -179.94–-190.41 (m, 1F). IR (KBr): 2940, 2873, 1587, 1498, 1452, 1232, 1191, 1033, 750 cm^{-1}. HRMS (EI) calcd. for $C_{17}H_{17}F_3^+$ [M$^+$]: 278.1282 found 278.1288.

4,4'-(3-Fluoropentane-1,5-diyl)bis(1,3-dimethylbenzene) **4k**

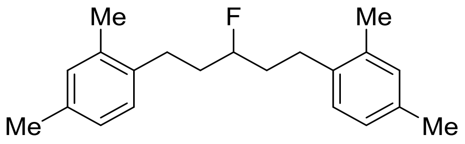

Semi-solid, 65% yield. ^1H NMR (300 MHz, $CDCl_3$) δ 7.07–6.91 (m, 6H), 4.66–4.41 (m, 1H, $^2J_{H\text{-}F}$ = 49.4 Hz), 2.83–2.72 (m, 2H), 2.70–2.56 (m, 2H), 2.29 (s, 6H), 2.27 (s, 6H), 1.99–1.67 (m, 4H). ^{13}C NMR (75 MHz, $CDCl_3$) δ 136.6, 135.7, 135.5, 131.1, 128.8, 126.6, 93.2 (d, *J* = 168.3 Hz), 35.9 (d, *J* = 21.0 Hz), 28.3 (d, *J* = 4.2 Hz), 20.9, 19.1. ^{19}F NMR (282 MHz, $CDCl_3$) δ -182.74–-183.43 (m, 1F). IR (KBr): 3008, 2952, 2819, 1606, 1515, 1448, 1375, 1037, 862, 813 cm^{-1}. HRMS (EI) calcd. for $C_{21}H_{27}F^+$ [M^+]: 298.2097 found 298.2094.

3,3'-(3-Fluoropentane-1,5-diyl)bis(1,2-dimethylbenzene) **4l**

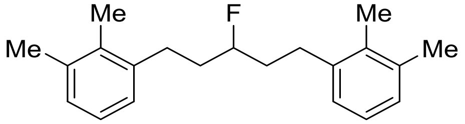

White solid, mp = 36–38 ^oC, 77% yield. ^1H NMR (300 MHz, $CDCl_3$) δ 7.06–6.83 (m, 6H), 4.66–4.44 (m, 1H, $^2J_{H\text{-}F}$ =49.1 Hz), 3.01–2.80 (m, 2H), 2.75–2.60 (m, 2H), 2.28 (s, 6H), 2.21 (s, 6H), 1.96–1.64 (m, 4H). ^{13}C NMR (75 MHz, $CDCl_3$) δ 139.5, 136.9, 134.4, 127.9, 126.9, 125.4, 93.2 (d, *J* = 168.2 Hz), 36.1 (d, *J* = 20.8 Hz), 29.4 (d, *J* = 3.8 Hz), 20.7, 14.9. ^{19}F NMR (282 MHz, $CDCl_3$) δ -173.12–-190.67 (m, 1F). IR (KBr): 3019, 2937, 1594, 1455, 1375, 1172, 1029, 889 cm^{-1}. HRMS (EI) calcd. for $C_{21}H_{27}F^+$ [M^+]: 298.2097 found 298.2096.

2,2'-(3-Fluoropentane-1,5-diyl)dinaphthalene **4m**

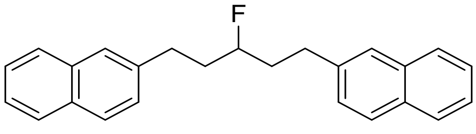

White solid, mp = 90–92 ^oC, 41% yield. ^1H NMR (300 MHz, $CDCl_3$) δ 7.89–7.68 (m, 6H), 7.60 (s, 2H), 7.49–7.37 (m, 4H), 7.31 (d, *J* = 8.4 Hz, 2H), 4.65–4.47 (m, 1H, $^2J_{H\text{-}F}$ = 49.3 Hz), 3.08–2.83 (m, 4H), 2.12–1.87 (m, 4H). ^{13}C NMR (75 MHz, $CDCl_3$) δ 138.8, 133.5, 131.9, 128.0, 127.5, 127.3, 127.1, 126.4, 125.9, 125.2, 92.6 (d, *J* = 168.2 Hz), 36.8 (d, *J* = 21.0 Hz), 31.5 (d, *J* = 4.3 Hz). ^{19}F NMR (282 MHz, $CDCl_3$) δ -182.84–-185.18 (m, 1F). IR (KBr): 3062, 2940, 1639, 1587, 1511, 1060, 862, 732 cm^{-1}. HRMS (EI) calcd. for $C_{25}H_{23}F^+$ [M^+]: 342.1784 found 342.1786.

(2-Fluorobutane-1,4-diyl)dibenzene **4n**

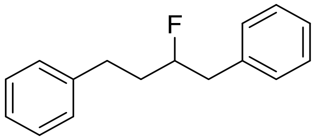

White solid, mp = 30–31 °C, 70% yield. ^{1}H NMR (300 MHz, $CDCl_3$) δ 7.34–7.23 (m, 4H), 7.24–7.11 (m, 6H), 4.87–4.60 (m, 1H, $^2J_{H\text{-}F}$ = 48.8 Hz), 3.06–2.80 (m, 3H), 2.76–2.59 (m, 1H), 2.02–1.80 (m, 2H). ^{13}C NMR (75 MHz, $CDCl_3$) δ 129.3, 128.4, 126.5, 125.9, 93.5 (d, J = 171.3 Hz), 41.6 (d, J = 21.4 Hz), 36.4 (d, J = 20.9 Hz), 31.3 (d, J = 4.2 Hz). ^{19}F NMR (282 MHz, $CDCl_3$) δ -180.42–-181.31 (m, 1F). IR (KBr): 3031, 2952, 2865, 1598, 1498, 1442, 1072, 838, 738, 694 cm^{-1}.HRMS (EI) calcd. for $C_{16}H_{17}F^+$ [M^+]: 228.1314 found 228.1317.

(3-Fluoro-6-methylheptyl)benzene **4p**

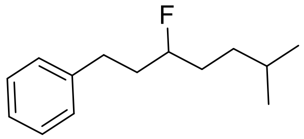

Colorless oil, 55% yield. ^{1}H NMR (300 MHz, $CDCl_3$) δ 7.32–7.03 (m, 5H), 4.58–4.34 (m, 1H, $^2J_{H\text{-}F}$ = 49.3 Hz), 2.87–2.59 (m, 2H), 2.06–1.75 (m, 2H), 1.72–1.44 (m, 3H), 1.42-1.15 (m, 2H), 0.90 (s, 3H), 0.87 (s, 3H). ^{13}C NMR (75 MHz, $CDCl_3$) δ 141.6, 128.43, 128.40, 125.8, 93.8 (d, J = 167.5 Hz), 36.9 (d, J = 21.1 Hz), 34.0 (d, J = 4.3 Hz), 33.0 (d, J = 20.7 Hz), 31.4 (d, J = 4.3 Hz), 27.9, 22.4 (d, J = 6.3 Hz). ^{19}F NMR (282 MHz, $CDCl_3$) δ -177.27–-184.97 (m, 1F). IR (KBr): 3023, 2944, 2864, 1590, 1494, 1463, 1382, 1060, 741, 698 cm^{-1}. HRMS (EI) calcd. for $C_{14}H_{21}F^+$ [M^+]: 208.1627 found 208.1635

(4-Fluorohexyl)benzene **4r**

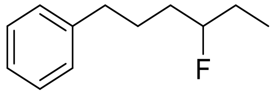

Colorless oil, 70% yield. ^{1}H NMR (300 MHz, $CDCl_3$) δ 7.33–7.24 (m, 2H), 7.23–7.13 (m, 3H), 4.59–4.28 (m, 1H, $^2J_{H\text{-}F}$ = 49.3 Hz), 2.65 (t, J = 7.3 Hz, 2H), 1.89–1.45 (m, 6H), 0.95 (t, J = 7.4 Hz, 3H). ^{13}C NMR (75 MHz, $CDCl_3$) δ 142.1, 128.3, 128.2, 125.7, 95.5 (d, J = 167.4 Hz), 35.6, 34.2 (d, J = 21.0 Hz), 28.0 (d, J = 21.5 Hz), 26.9 (d, J = 4.1 Hz), 9.4 (d, J = 5.8 Hz). ^{19}F NMR (282 MHz, $CDCl_3$) δ -181.54–-182.72 (m, 1F). IR(KBr): 3027, 2933, 2819, 1606, 1494, 1463, 1363, 1097, 944, 709 cm^{-1}. HRMS (EI) calcd. for $C_{12}H_{17}F^+$ [M^+]: 180.1314 found 180.1322.

(4-Fluorooctyl)benzene **4s**

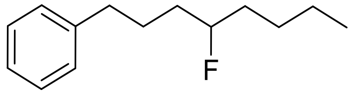

Colorless oil, 53% yield. ^{1}H NMR (300 MHz, $CDCl_3$) δ 7.33–7.24 (m, 2H), 7.22–7.15 (m, 3H), 4.58–4.37 (m, 1H, $^2J_{H\text{-}F}$ = 49.3 Hz), 2.64 (t, J = 7.3 Hz, 2H), 1.82–1.45 (m, 6H), 1.44–1.22 (m, 4H), 0.90 (t, J = 6.9 Hz, 3H). ^{13}C NMR (75 MHz, $CDCl_3$) δ 142.1, 128.3, 128.2, 125.7, 94.3 (d, J = 166.8 Hz), 35.6, 34.8 (d, J = 11.5 Hz), 34.6 (d, J = 11.7 Hz), 27.2 (d, J = 4.4 Hz), 26.9 (d, J = 4.2 Hz), 22.5, 13.9. ^{19}F NMR (282 MHz, $CDCl_3$) δ -180.40–181.34 (m, 1F). IR (KBr): 3019, 2937, 2865, 1602, 1494, 1448, 1378, 1022, 764, 690 cm^{-1}. HRMS

(EI) calcd. for $C_{14}H_{21}F^+$ [M^+]: 208.1627 found 208.1622

Synthesis of compound 1v-y, 3x and 3y, related to Table 2.

In Table 2, substrates such as 1,5-diphenylpentan-3-one (**1v**), (3,3-dimethoxypentane-1,5-diyl)dibenzene (**1w**), (3,3-dichloropentane-1,5-diyl)dibenzene (**1x**), (3,3-dibromopentane-1,5-diyl)dibenzene (**1y**) and elimination product (3-chloropent-2-ene-1,5-diyl)dibenzene (**3x**), were known compounds, and were synthesized followed literature report (Blümel et al., 2018; Takeda et al., 1997; Mukaiyama et al., 1973).

(3-bromopent-2-ene-1,5-diyl)dibenzene (**3y**) was new compound, as yellow oil. The *Z*/*E* ratio (10:1) was determined by ^{1}H-NMR. HRMS (EI) calcd. for $C_{17}H_{17}Br^+$ [M^+]: 300.0514, found 300.0518. ^{1}H NMR (300 MHz, $CDCl_3$) δ 7.32–7.15 (m, 8H), 7.06 (d, *J* = 6.8 Hz, 2H), 5.72 (t, *J* = 7.0 Hz, 1H), 3.48 (d, *J* = 6.9 Hz, 2H), 2.93–2.83 (m, 2H), 2.83–2.71 (m, 2H). ^{13}C NMR (126 MHz, $CDCl_3$) δ 140.4, 139.2, 128.6, 128.4, 128.34, 128.31, 128.13, 128.12, 126.1, 126.0, 43.4, 37.5, 34.4. IR (KBr): 3019, 2921, 2844, 1654, 1598, 1490, 1452, 1081, 686 cm^{-1}.

Supplemental References

Nahra, F., Patrick, S.R., Bello, D., Brill, M., Obled, A., Cordes, D.B., Slawin, A.M.Z., O'Hagan, D., and Nolan, S.P. (2015). Hydrofluorination of Alkynes Catalysed by Gold Bifluorides. ChemCatChem *7*, 240-244.

Yang, M.-H., Matikonda, S. S., and Altman, R. A. (2013). Preparation of Fluoroalkenes via the Shapiro Reaction: Direct Access to Fluorinated Peptidomimetics. Org. Lett. *15*, 3894-3897.

Zhang, W., Huang, W., and Hu, J. (2009). Highly Stereoselective Synthesis of Monofluoroalkenes from α-Fluorosulfoximines and Nitrones. Angew. Chem. Int. Ed. *48*, 9858-9861.

Vandamme, M., and Paquin, J.-F. (2017). Eliminative Deoxofluorination Using XtalFluor-E: A One-Step Synthesis of Monofluoroalkenes from Cyclohexanone Derivatives. Org. Lett. *19*, 3604-3607.

Khalaf, A. A., and Roberts, R. M. (1972). Friedel-Crafts cycloalkylations of certain mono- and diphenyl-substituted alcohols and alkyl chlorides. J. Org. Chem. *37*, 4227.

Adamczyk, M., Watt, D. S., and Netzel, D. A. (1984). Synthesis of biological markers in fossil fuels. 2. Synthesis and carbon-13 NMR studies of substituted indans and tetralins. J. Org. Chem. *49*, 4226-4237.

Léonard, N. G., and Chirik, P. J. (2018). Air-Stable α-Diimine Nickel Precatalysts for the Hydrogenation of Hindered, Unactivated Alkenes. ACS Catal. *8*, 342-348.

Michelet, B., Bour, C., and Gandon, V. (2014). Gallium-Assisted Transfer Hydrogenation of Alkenes. Chem. Eur. J. *20*, 14488-14492.

Umemoto, T., Singh, R. P., Xu, Y., and Saito, N. (2010). Discovery of 4-tert-Butyl-2,6-dimethylphenylsulfur Trifluoride as a Deoxofluorinating Agent with High Thermal Stability as Well as Unusual Resistance to Aqueous Hydrolysis, and Its Diverse Fluorination Capabilities Including Deoxofluoro-Arylsulfinylation with High Stereoselectivity. J. Am. Chem. Soc. *132*, 18199-18205.

Blümel, M., Nagasawa, S., Blackford, K., Hare, S.R., Tantillo, D.J., and Sarpong, R. (2018). Rearrangement of Hydroxylated Pinene Derivatives to Fenchone-Type Frameworks: Computational Evidence for Dynamically-Controlled Selectivity. J. Am. Chem. Soc. *140*, 9291-9298.

Takeda, T., Sasaki, R., Yamauchi, S., and Fujiwara, T. (1997). Transformation of ketones and aldehydes to gem-dihalides via hydrazones using copper(II) halides. Tetrahedron *53*, 557-566.

Mukaiyama, T., Imamoto, T., and Kobayashi, S. (1973). Convenient method for the hydrolysis of vinyl chlorides to ketones. Chem. Lett. *2*, 261-264.
